# Chitotriosidase - a putative biomarker for sporadic amyotrophic lateral sclerosis

**DOI:** 10.1186/1559-0275-10-19

**Published:** 2013-12-02

**Authors:** Anu Mary Varghese, Aparna Sharma, Poojashree Mishra, Kalyan Vijayalakshmi, Hindalahalli Chandregowda Harsha, Talakad N Sathyaprabha, Srinivas MM Bharath, Atchayaram Nalini, Phalguni Anand Alladi, Trichur R Raju

**Affiliations:** 1 Department of Neurophysiology, National Institute of Mental Health and Neuro Sciences, Hosur Road, Post Box no.: 2900, Bangalore 560 029, India; 2 Institute of Bioinformatics, Discoverer Building, International Tech Park, Whitefield, Bangalore 560 066, India; 3 Department of Neurochemistry, National Institute of Mental Health and Neuro Sciences, Hosur Road, Bangalore 560 029, India; 4 Department of Neurology, National Institute of Mental Health and Neuro Sciences, Hosur Road, Bangalore 560 029, India

**Keywords:** Proteomics, Cerebrospinal fluid, Sporadic amyotrophic lateral sclerosis

## Abstract

**Background:**

Potential biomarkers to aid diagnosis and therapy need to be identified for Amyotrophic Lateral Sclerosis, a progressive motor neuronal degenerative disorder. The present study was designed to identify the factor(s) which are differentially expressed in the cerebrospinal fluid (CSF) of patients with sporadic amyotrophic lateral sclerosis (SALS; ALS-CSF), and could be associated with the pathogenesis of this disease.

**Results:**

Quantitative mass spectrometry of ALS-CSF and control-CSF (from orthopaedic surgical patients undergoing spinal anaesthesia) samples showed upregulation of 31 proteins in the ALS-CSF, amongst which a ten-fold increase in the levels of chitotriosidase-1 (CHIT-1) was seen compared to the controls. A seventeen-fold increase in the CHIT-1 levels was detected by ELISA, while a ten-fold elevated enzyme activity was also observed. Both these results confirmed the finding of LC-MS/MS. CHIT-1 was found to be expressed by the Iba-1 immunopositive microglia.

**Conclusion:**

Elevated CHIT-1 levels in the ALS-CSF suggest a definitive role for the enzyme in the disease pathogenesis. Its synthesis and release from microglia into the CSF may be an aligned event of neurodegeneration. Thus, high levels of CHIT-1 signify enhanced microglial activity which may exacerbate the process of neurodegeneration. In view of the multifold increase observed in ALS-CSF, it can serve as a potential CSF biomarker for the diagnosis of SALS.

## Background

Selective loss of cortical and spinal motor neurons is the characteristic feature of Amyotrophic Lateral Sclerosis (ALS), an adult onset progressive fatal neurodegenerative disorder. Factors predisposing the most prominent form of this multifactorial disease viz. sporadic ALS (SALS) remain obscure due to the difficulties in developing animal models. Therefore, development of novel therapeutics is also severely hampered. This is also largely attributed to the lack of a ‘biomarker’ which can be objectively measured as an indicator of pathogenic processes and/or pharmacologic response to therapeutic interventions [1]. The discovery of ideal biomarkers may offer tools for rapid diagnosis, monitoring disease progression and provide insights into the pathophysiology of the disease; thereby broadening therapeutic options. Proximity to the central nervous system (CNS) renders Cerebrospinal Fluid (CSF) to be the ideal biofluid for detection of biomarkers in CNS pathologies. It is speculated that toxic agents which propagate the disease are synthesized in the affected areas, injure the neighboring cells, and are released into the extracellular space and CSF [2,3].

We have earlier shown that exposure of embryonic rat spinal cord cultures to ALS-CSF (*in-vitro*) and intrathecal injection of the same into neonatal rats (*in-vivo*) induced degenerative changes in motor neurons and showed the involvement of astrocytes [4-11]. Intracerebroventricular infusion of ALS-CSF in adult rats perturbed the cortical motor neuronal activity and was associated with poor motor performance [12]. Thus several studies, including ours, support the presence of toxic factor(s) in ALS-CSF and attribute them a role in eliciting its pathophysiology [3,13-15].

We undertook a study to identify the toxic factor(s) in ALS-CSF through proteomic analysis. Quantitative mass spectrometric analysis of ALS-CSF compared to age-matched controls showed upregulation of 31 proteins, amongst which Chitotriosidase-1 (CHIT-1) showed more than 10 fold increase. The biological significance of CHIT-1 expression is intriguing in view of the absence of its natural substrate chitin in human brain. Earlier studies have shown an increase in CSF CHIT-1 levels in multiple sclerosis (MS) and Alzheimer’s disease (AD) [16,17].

## Results

### Confirmation of toxicity of ALS-CSF samples

**Figure 1:**
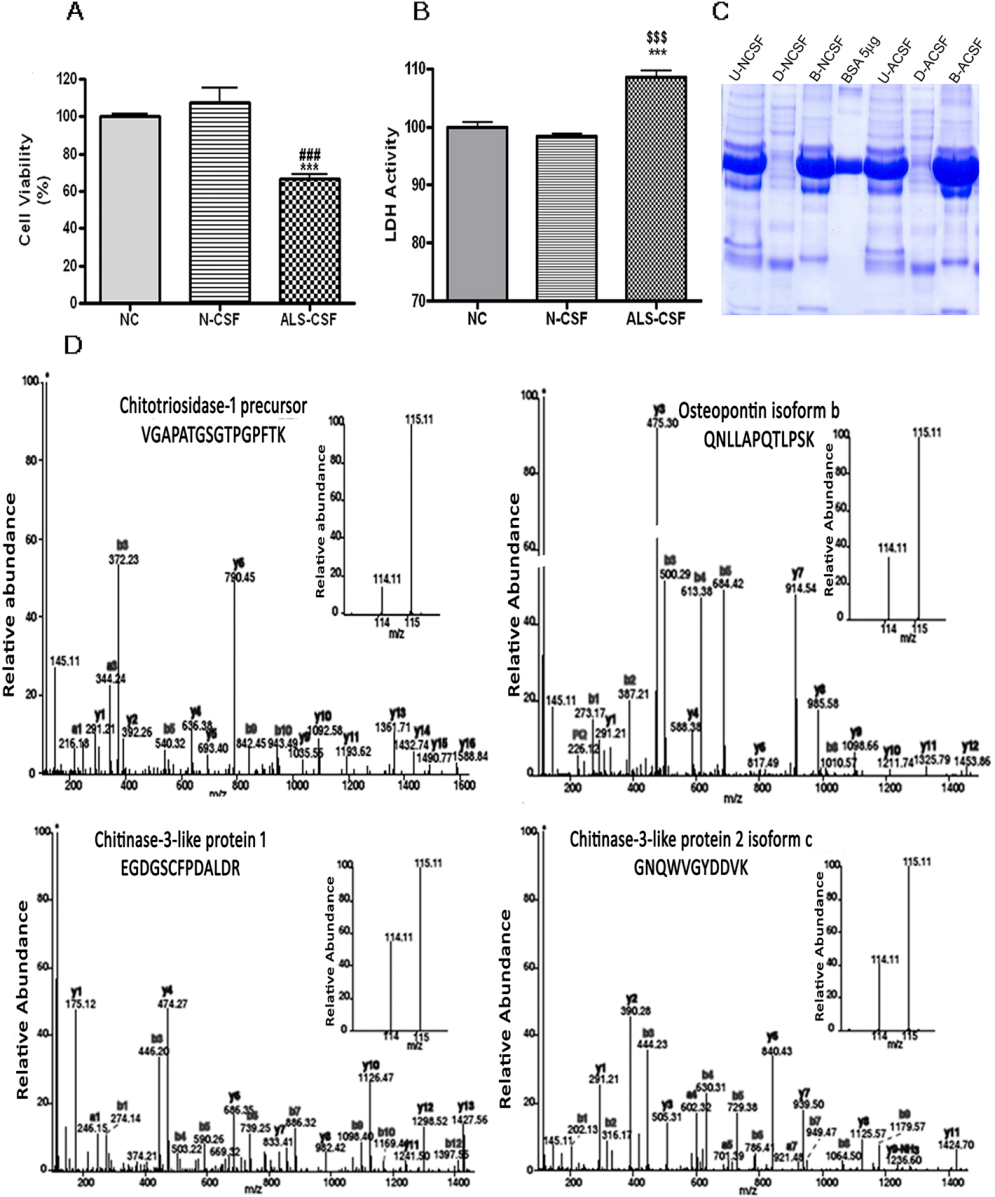
**Mass spectrometric analysis of ALS-CSF samples: Toxicity assays (A & B) were performed.** Histogram of MTT assay showing 40% reduction in the viability of NSC-34 cells upon exposure to 10%(v/v) ALS-CSF compared to the cells exposed to normal-CSF (###p < 0.001 vs. N-CSF) and 30% compared to normal controls (***p < 0.001 vs. NC; **A**). ALS-CSF caused significant increase in LDH activity when compared to control groups (***p < 0.001 vs. NC and ^$$$^p < 0.001 vs. N-CSF; **B**). Gel image representing depletion of abundant proteins prior to mass spectrometric analysis (**C**). Representative MS/MS spectra of the peptides of CHIT1, Osteopontin, CHI3L1 and CHI3L2 (**D**). Tests of significance was Student’s *t* test, One way Anova followed by Tukey’s post hoc analysis.

The toxicity of the ALS-CSF samples was confirmed prior to proteomic analysis. Exposure of NSC-34 cells to the CSF obtained from ALS patients (ALS-CSF) resulted in a dramatic decrease in their viability when compared to the control groups; i.e. the cells treated with CSF from normal individuals (N-CSF) or without CSF (NC) (***p < 0.001 vs NC; ###p < 0.001 vs N-CSF; Figure 1A). It also induced enhancement of LDH activity in ALS-CSF group (***p < 0.001 vs. NC and $$$p < 0.001 vs. N-CSF; Figure 1B).

### Proteomic analysis of control and ALS-CSF samples

Ten CSF samples each from controls and ALS were pooled, depleted of abundant proteins and electrophoresed on SDS-PAGE (Figure 1C). Total protein from control and ALS-CSF were subjected to tryptic digestion, followed by liquid chromatography-tandem mass spectrometry (LC-MS/MS) after labeling with isobaric tags for relative and absolute quantitation (iTRAQ). LC-MS/MS analysis identified 819 proteins using SEQUEST and Mascot (Additional file 1: Table S1). Approximately 31 proteins showed more than 1.5-fold increase, suggesting an up- regulation (Additional file 2: Table S2) and about 17 proteins were down-regulated (decrease of 0.5 fold or more) in ALS-CSF samples compared to the normal controls (Additional file 3: Table S3). Four of the prominently up-regulated proteins were CHIT-1 (10 fold), osteopontin isoform-b (3 fold), chitinase-3-like protein 2 (CHI3L2; 2 fold) and chitinase-3-like protein 1 (CHI3L1; 1.7 fold). Thus CHIT-1 showed the most dramatic increase (Table 1, Figure 1D).

**Table 1:** List of proteins upregulated in ALS-CSF

**S. No**	**Gene symbol**	**Protein name**	**Relative expression (ALS-CSF/N-CSF) fold change**
1	CHIT1	chitotriosidase-1 precursor [Homo sapiens]	10
2	SPP1	osteopontin isoform b precursor [Homo sapiens]	3
3	CHI3L2	chitinase-3-like protein 2 isoform c [Homo sapiens]	2
4	CHI3L1	chitinase-3-like protein 1 precursor [Homo sapiens]	1.7

### Validation of LC-MS/MS data by ELISA

**Figure 2:**
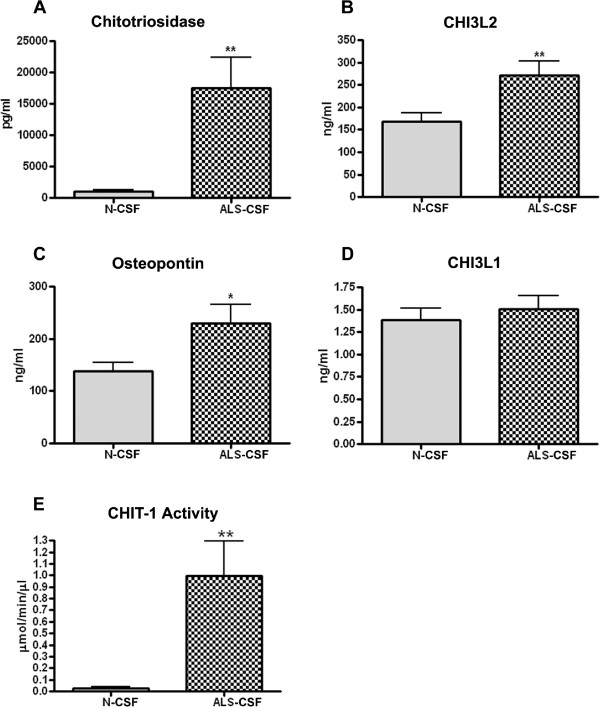
**Validation of upregulated proteins by ELISA and CHIT-1 activity assay.** Chitotriosidase was found to be 17 fold upregulated (**p < 0.01 vs. N-CSF; **A**) and chitinase 3- like-2 (CHI3L2) showed ~2 fold increase (**p < 0.01 vs. N-CSF; **B**) in ALS-CSF. Osteopontin showed ~ 1.9 fold upregulation (*p < 0.05 vs. N-CSF; **C**) whereas, upregulation in Chitinase 3- like-1 (CHI3L1) levels was statistically non- significant (**D**). Histogram showing CHIT-1 enzymatic activity in CSF samples. The activity was found to be 10 fold higher in ALS-CSF (**p < 0.001 vs. N-CSF; **E**).

Observations obtained by LC-MS/MS were confirmed by ELISA. The basal values of CHIT-1 in the control CSF ranged as wide as 80 – 1250 pg/ml, with a mean of 982 ± 245 (n = 11). In the ALS-CSF, the level of CHIT-1 ranged between 5000 – 54,000 pg/ml, with a mean of 17570 ± 4883 pg/ml (n = 16). The mean increase in the ALS-CSF was approximately 17 fold (**p < 0.01 vs N-CSF; Figure 2A). Amongst the tested CSF samples of ALS patients, 27% showed a 4-fold increase, whereas 73% showed an increase of 10-fold or more.

Similar to the LC-MS/MS data, CHI3L2 levels showed approximately 2-fold increase (**p < 0.01 v/s N-CSF; no. of samples: N-CSF =13, ALS-CSF = 16) while osteopontin levels showed 1.9-fold increase in ALS-CSF compared to the control CSF (*p < 0.05 v/s N-CSF; no. of samples: N-CSF =13, ALS-CSF = 16) (Figure 2B & C). Although the CHI3L1 level was also increased, the change was not statistically significant (Figure 2D).

### Validation of LC-MS/MS data by enzyme assay

The CHIT-1 in the CSF samples catalyzed the conversion of 4-methylumbelliferyl-β - d N, N’, N” –triacetylchitotriose to 4-methylumbelliferone confirming that the enzyme was biologically active. The CHIT-1 activity in the control CSF ranged between 0.0169 – 0.1856 μmol/min/μl (Mean: 0.02459 ± 0.01499; n = 13) whereas in the ALS-CSF it was 0.0809 – 4.1658 μmol/min/μl (Mean: 0.9932 ± 0.3023; n = 16). Thus the patient CSF samples revealed approximately a 10-fold higher enzymatic activity (**p < 0.001 vs N-CSF; Figure 2E).

### CHIT-1 expression in microglia

**Figure 3:**
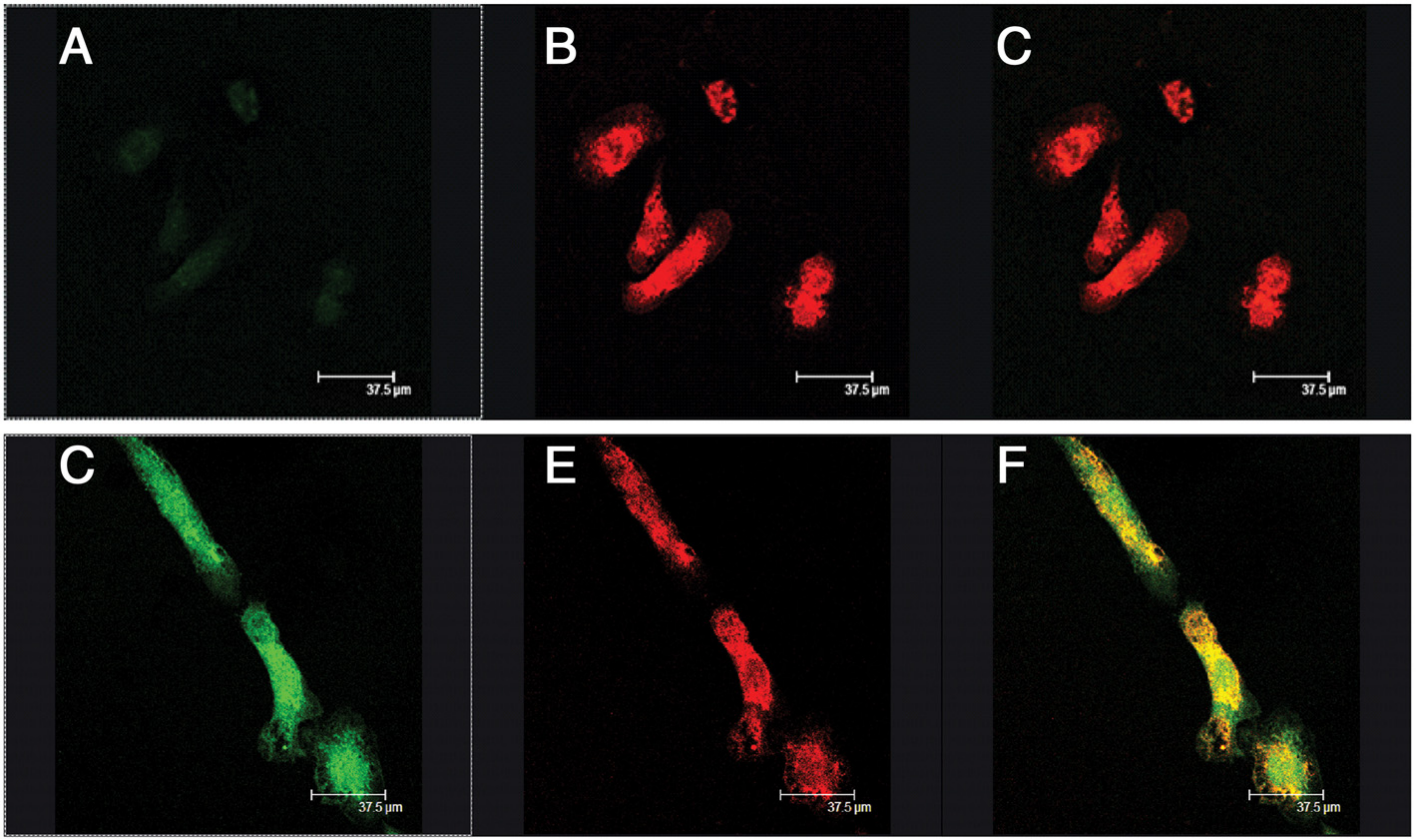
**Expression of CHIT-1 in microglial cultures.** Immunoflourescence photomicrographs of pure microglial cultures labeled with CHIT-1 **(green; A, D)** and Iba-1, a marker for microglia **(red; B, E)**. Note the increased expression of CHIT-1 in the cultures exposed to ALS-CSF **(D)** as compared to the normal control **(A)**. The merged images **(C and F)** depict the co-labeling of CHIT-1 with Iba-1 in normal control **(A)** and ALS group **(D)** respectively.

The Iba-1 immunoreactive pure microglial cultures when exposed to ALS-CSF, showed an elevated expression of CHIT-1 compared to control cultures (Figure 3).

## Discussion

This is the first report demonstrating an increase in the levels of four proteins namely, CHIT-1, osteopontin, CHI3L2 and CHI3L1 in the CSF of ALS patients using the novel and precise quantitative proteomics and ELISA. Similar patterns were observed with both LC-MS/MS of pooled CSF and ELISA of individual CSF. Amongst these the increase in CHIT-1 levels being most dramatic; further experiments were focused on this protein. A profound increase in CHIT-1 enzymatic activity was confirmed in ALS-CSF compared to normal CSF. Accordingly, it is likely to be an important biomarker which may help track the progression of the disease. CHIT-1 could have a role in causing toxicity propagated by the CSF, since the ALS-CSF samples used in the present study also contained neurodegenerative attributes as confirmed by MTT and LDH assays.

CHIT-1 is classically associated with lysosomal storage diseases like Gaucher’s disease, due to its increased levels in the patients’ CSF [18]. Chitin is the natural substrate of CHIT-1, but is thought to be absent in the human brain. However, it is intriguing to know that even in the absence of the substrate the enzyme is synthesized by microglia or infiltrating macrophages [19].

It is uncertain whether CHIT-1 activity directly affects the CNS or symbolizes an archaic macrophage response against chitin-containing pathogens [19,20]. Chitin is an insoluble N-acetylglucosamine polymer found in invertebrates and human parasites [21,22]. In its absence, alternative substances e.g. chitin-like glucosamine polymers may be its likely substrate. In post mortem brains of AD patients, presence of glucosamine polymers in amyloid plaques hints at its role in disease pathogenesis [16]. It is also suggested that chitin-like polysaccharides provide scaffolding for β-amyloid deposition thus facilitating the disease process [23]. Higher CHIT-1 expression in post mortem brains of AD patients [24] underlines the co-activation of macrophages and microglia in response to deposition of β-amyloid [25].

It is reported that higher CSF CHIT-1 levels in MS patients correlate well with the disease progression [26]. In the MS patients, the increase in CSF CHIT-1 levels was approximately two-fold, however we observed approximately 10-fold increase in CHIT-1 levels in the patient CSF. Although it is not justified to compare the differences in the patients’ CSF as their study was on MS whereas we studied ALS patients, it is also likely that the differences in fold levels of CSF CHIT-1 are indeed high in ALS compared to MS.

Contrary to AD, deposits of chitin-like substances were absent in MS. Although microglia and infiltrating macrophages driven innate immune response was a classical molecular feature of MS, the products of the latter processes viz. cytokines, ROS etc. were not substantial enough to establish any clinico-pathological co-relation [17]. However, Correale and Fiol reported that the enhanced level of CSF chitinases driven by IL-13 could contribute to neuroinflammation by increasing immune cell migration across the blood–brain barrier in the CNS [26].

The enhanced expression of CHIT-1, by microglia, possibly indicates a neuroinflammatory response. CHIT-1 is an index of the severity of inflammation alongside the release of pro-inflammatory cytokines like IL-16 and IL-18 [27]. In stroke, CHIT-1, TNF-α and other pro-inflammatory cytokines are accepted as markers of microglial activation, occurring independent of pre-existing inflammatory or infectious conditions in patients [28]. According to an alternative hypothesis, CHIT-1 is speculated to be neuroprotective, in view of the reduction in glucosamine aggregates following intrathecal CHIT-1 administration in MS [17]. Even in AD, enhanced levels of CHIT-1 activity in plasma were considered as the response of the activated microglia-macrophage complex to clear the pathogenic chitin-like substances [16,25]. Its role in ALS is yet to be deciphered.

## Conclusion

Although studies report higher CSF CHIT-1 levels in several neurological diseases including MS, AD and stroke, no study till date documents its elevated levels in ALS, where maximum increase was observed compared to other neurodegenerative diseases. The mechanisms of CHIT-1 induction in each of the neurological disorders appear to be unique to the disease. Collectively, our findings of steep increase in CSF CHIT-1 levels in SALS along with stable bioactivity render it a biomarker status and may find applications in developing therapeutic strategies for sporadic ALS.

## Materials and methods

### CSF sample collection

ALS-CSF samples from patients with a mean age of 47.38 ± 5.38 years and disease duration of 0.5 to 2.5 years were obtained. N-CSF samples were drawn from age-matched patients undergoing spinal anaesthesia for orthopaedic surgery but without any clinical history of neurological deficits (mean age 45.7 ± 7.04 years) (Tables 2 and Table 3). CSF samples were snap frozen in liquid nitrogen and stored at –80°C. Informed consent for CSF sample collection was obtained as per the institutional human ethics committee guidelines.

**Table 2:** Details of ALS-CSF

	**CSF for iTRAQ study**	**CSF for validation**		
Gender	Male – 7 (70%)	Female – 3 (30%)	Male – 10 (62.5%)	Female – 6 (37.5%)
Age at presentation (Mean ± SD)	47.40 ± 4.95 (38 – 54) Years	47.38 ± 5.38 (38 – 54) Years		
Age at onset	46 ± 5.05 (37 – 53) Years	46.28 ± 5.36 (37 – 53) Years		
Duration of illness (Mean ± SD)	15.9 ± 13.4 (4.0 – 48) months	14.19 ± 10.59 (4.0 – 48) months		
Onset Patter: Bulbar	1 (10%)	5 (31.25%)		
Limb onset	9 (90%)	11 (68.75%)		
Upper Limbs	9	11		
Lower Limbs	6	7		
Speech affected	80% (Mild – 30%, Moderate – 30%, Severe – 20%)	87.5% (Mild – 31.25%, Moderate – 31.25%, Severe – 25%)		
Dysphagia	80% (Mild – 30%, Moderate – 30%, Severe – 20%)	93.75% (Mild – 31.25%, Moderate – 18.75%, Severe -12.5%)		
Spasticity	9 cases, with evidence of pyramidal signs in the form of spasticity and exaggerated Deep Tendon Reflexes	11 cases, with evidence of pyramidal signs in the form of spasticity and exaggerated Deep Tendon Reflexes		

**Table 3:** Details of N-CSF

	**CSF for iTRAQ study**		**CSF for validation**	
Gender	Male – 8 (80%)	Female – 2 (20%)	Male – 11 (84.6%)	Female – 2 (15.4%)
Age (Mean ± SD in years)	47.3 ± 6.99 (39 – 60)		45.7 ± 7.04 (39 – 60)	
Patients undergoing orthopaedic surgery				

### Cell culture

We followed a modified protocol to establish pure microglial cultures from P0 Wistar rat pups [29]. Briefly, the spinal cords were dissected, freed of meninges and mechanically triturated in Dulbecco’s Modified Eagle Medium (DMEM) and propagated in DMEM with 10% FBS (GIBCO-BRL). The mixed glial cultures were allowed to attain 100% confluence. On 10th day *in-vitro* (DIV), the cultures were placed on incubated orbital shaker at 200 rpm for 3–4 hrs. The supernatant containing microglia was centrifuged at 1500 rpm for 5 min, and the pelleted cells were seeded onto poly-l-lysine coated coverslips at the density of 4.4×104 cells/ml. The cultures were maintained in DMEM with 10% FBS (GIBCO-BRL). The cells were then exposed to ALS- CSF or allowed to propagate under normal conditions.

NSC-34 cell line (Cedarlane Corporation, Canada) routinely maintained in DMEM with 10% FBS [30] was used to analyze the neurotoxic effects of individual ALS-CSF samples.

### Cell viability/death assays

NSC-34 cells seeded into 96 well plates (500 cells/well) were exposed to 10% v/v N-CSF or ALS-CSF on 5th DIV for 48 hrs, and then subjected to MTT assay (3-(4,5-Dimethylthiazol-2-yl)-2,5-diphenyltetrazolium bromide) and LDH assay [30].

### Mass spectrometry

#### ***Depletion of abundant proteins in CSF***

**Figure 4:**
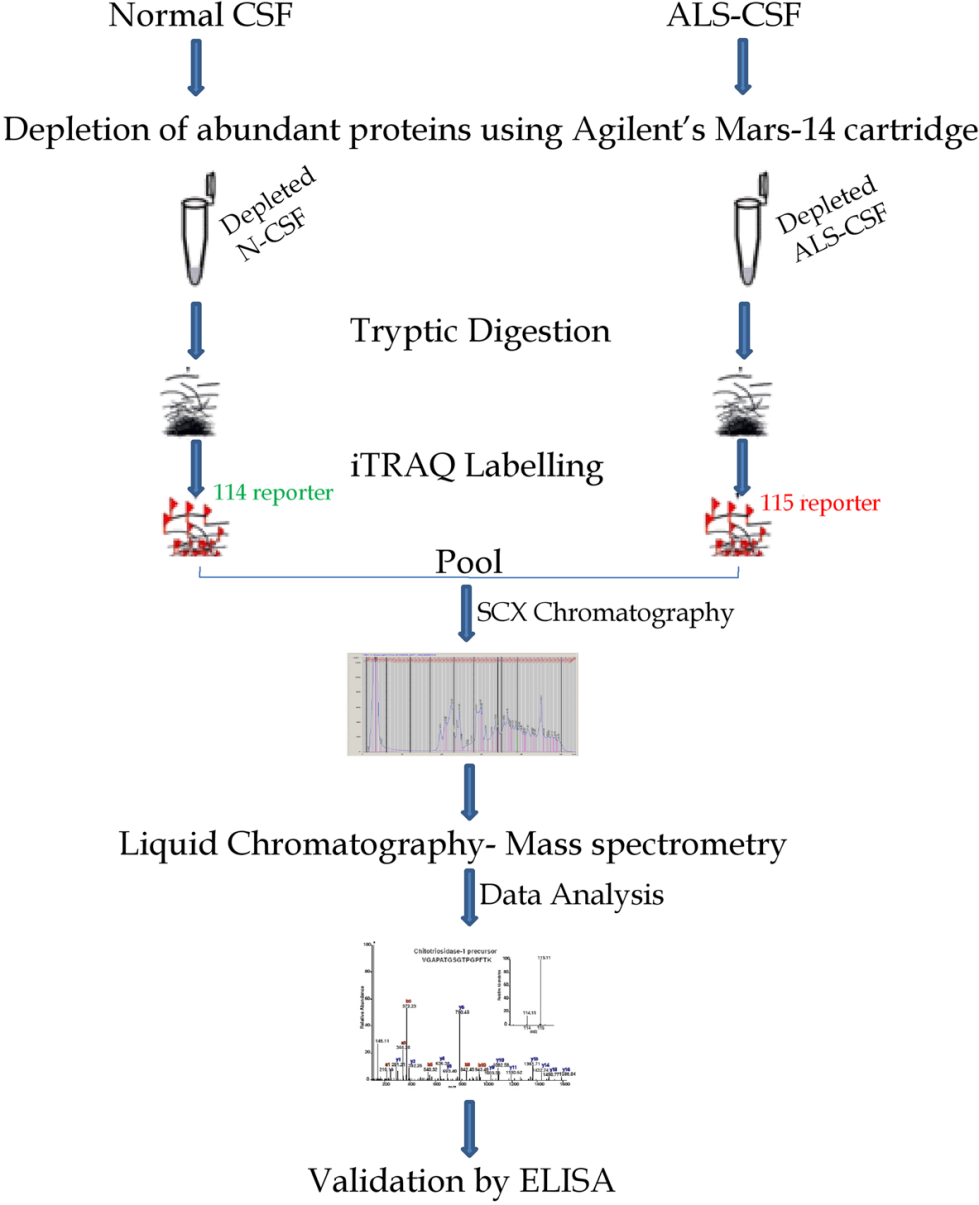
**A schematic representation of the procedural steps for mass spectrometric analysis.** Pooled CSF samples were subjected to mass spectrometry after sequential treatment procedures like, depletion of abundant proteins, tryptic digestion, iTRAQ labelling and SCX. Selected molecules were validated using ELISA.

CSF samples from controls (n = 10) and ALS patients (n = 10) were pooled individually and used for iTRAQ based quantitative proteomic comparison by mass spectrometry (Figure 4). Samples were centrifuged at 10,000 rpm for 10 min to remove cell debris. Agilent’s multiple affinity removal system (MARS-14), generally used for depletion of albumin, IgG, transferrin, heptoglobin, IgM, IgA, fibrinogen, alpha antitrypsin, apolipoprotein A1, alpha1 acid glycoprotein, alpha2 macroglobin, transthyretin, complement C3 and apolipoproteins was used to deplete the abundant proteins from CSF samples [31]. Briefly, MARS-14 cartridge was conditioned using 4 ml of Buffer-A (50 g, 1 min). CSF (300 μl) was loaded on to the cartridge and spun at 50 g for 1 min. Flow through was collected and reloaded on to the cartridge, incubated for 5 minutes at room temperature (RT) and spun at 50 g for 1 min. Flow through was re-collected and the bound proteins were eluted using 2.4 ml of Buffer-B. The cartridge was conditioned again using buffer-A and the process was repeated until the abundant proteins were depleted. The fractions were concentrated using Millipore filter (3 kDa). This step also ensures removal of salts contributed by the buffers used during depletion steps.

Protein estimation was carried out using Lowry’s method. 200 μg protein each from controls and ALS was used for proteomic comparison. Sample normalization was based on total protein amount which was further verified by SDS-PAGE.

#### ***iTRAQ labelling and strong cation exchange chromatography***

Individual depleted extract (125 μg) was treated with 2% SDS and reducing agent (2 μl; 60°C for 1 hr) and alkylated with 1 μl of cysteine blocking agent for 10 min at RT. Proteins were digested overnight at 37°C using sequencing grade trypsin at 1:20 (w/w) ratio. Thereafter, peptides from N-CSF and ALS-CSF were labeled using iTRAQ reagents yielding reporter ions of m/z114 and 115 respectively, for 2 hrs at RT. The reaction was quenched by adding 100 μl of water and the peptides from both N-CSF and ALS-CSF were pooled and fractionated using strong cation exchange (SCX) chromatography. The labeled samples were distributed into 19 fractions using SCX. The fractions were desalted using C18 zip tips, dried and reconstituted in 10 μl of 0.1% trifluoroacetic acid before mass spectrometry analysis [32].

#### ***LC-MS/MS analysis***

LC-MS/MS analysis of iTRAQ-labeled peptides was carried out on an LTQ-Orbitrap Velos mass spectrometer (Thermo Electron, Bremen, Germany) interfaced with Agilent’s 1200 series nanoflow liquid chromatography system (Agilent Technologies, Santa Clara, CA). Each sample was loaded on to the enrichment column (75 μm × 2 cm, 5 μm, 120 Å, Magic C18 AQ MichromBioresources) at a flow rate of 3 μl/min and then resolved on an analytical column (75 μm × 10 cm, 5 μm, 120 Å, Magic C18 AQ MichromBioresources) at a flow rate of 300 nl/min using a linear gradient of 10% - 40% solvent B (90% acetonitrile in 0.1% TFA) over a period of 70 min. The total run time per sample was 115 min. The resolved peptides from analytical column were delivered to LTQ Orbitrap Velos mass spectrometer through an emitter tip (8 μm, New Objective, Woburn, MA). LC-MS/MS data was acquired in a data dependent manner in FT- FT mode. MS spectra were acquired with a mass range of m/z 350 to 1800. Twenty most abundant precursor ions were selected for fragmentation from each MS scan. Data was acquired at MS resolution of 60,000 (m/z 400) and MS/MS resolution of 15,000. Precursor ion fragmentation was carried out using higher energy collision (HCD) mode with normalized collision energy of 41%. Monoisotopic precursor selection was enabled and the precursor ions that were selected for fragmentation were dynamically excluded for 30 sec [32].

#### ***Data analysis***

The MS data was analyzed using the Proteome Discoverer software (Thermo Scientific, version 1.2). The data was searched against human protein database (NCBI RefSeq 45) along with known contaminants using SEQUEST and MASCOT search algorithms. The parameters used for data analysis included trypsin as a protease (allowed one missed cleavage), iTRAQ labeling at N-terminus and lysine residues, and cysteine modification by methyl methanethiosulfonate (MMTS) as fixed modifications and oxidation of methionine as a variable modification. The precursor ion mass error tolerance was set to 20 ppm and product ion mass error tolerance was set to 0.1 Da. The peptide data was extracted using 1% FDR as a threshold. Relative abundance of proteins between N-CSF and ALS-CSF was determined by Proteome Discoverer based on difference in the peak intensity of reporter ions in the MS/MS spectra of each peptide that was ultimately used for quantifying the corresponding protein.

#### ***Enzyme linked immunosorbent assays (ELISA)***

N-CSF (n = 13) and ALS-CSF samples (n = 16) were subjected to ELISA based analysis using commercially available kits for CHIT-1 (MBL, Italy; CSF dilution factor (cdf): N-CSF: undiluted; ALS-CSF 1:20), CHI3L1 (Quidel, USA), CHI3L2 (USCN, China; cdf 1:1) and Osteopontin (R&D Systems; cdf 1:50). The protocol was followed as per the manufacturer’s instructions.

#### ***CHIT-1 activity assay***

4-methylumbelliferyl-β - d N, N’, N” –triacetylchitotriose (Sigma-Aldrich USA) was used as a substrate to assay the enzyme activity. CSF (2.5 g protein) was added to 150 μl of 22 μmol solution prepared in 0.5 M citrate-phosphate buffer (pH 5.2). Following incubation for 15 min at 37°C, the reaction was stopped using 100 μl of 0.5 mol Na_2_CO_3_ -NaHCO_3_ buffer (pH 10.7). The fluorescence was recorded at 365 nm excitation/450 nm emissions (Tecan 2500 flouorimeter, USA) and measured as micromoles of substrate hydrolysed/min/l.

#### ***Immunocytochemistry***

Fixed primary microglial cultures were stained with rabbit polyclonal anti-CHIT-1 antibody (1:1000, Santacruz, USA) for 24 hr after blocking with 3% bovine serum albumin (BSA) (Sigma–Aldrich, USA) and detected using FITC-conjugated anti-rabbit secondary antibody (1:200, Sigma–Aldrich, USA). The cultures were further incubated with a goat polyclonal anti Iba-1 antibody (1:400, Abcam, UK) for 24 hr and detected with CY3-conjugated anti-goat antibody (1:200, Sigma–Aldrich, USA). The staining was viewed using confocal microscopy (488 nm and 514 nm for FITC and Cy3, respectively; Leica-TCS-SL, Germany). The emission frequencies were segregated to avoid non-specific overlap of labelling [11].

### Statistical analysis

The data was statistically assessed for significance by either Student’s *t* test or one way ANOVA as applicable, followed by Tukey’s post hoc tests.

## Abbreviations

SALS: Sporadic amyotrophic lateral sclerosis; ALS-CSF: Cerebrospinal fluid from amyotrophic lateral sclerosis patients; N-CSF: CSF from patients undergoing orthopaedic surgery; CHIT-1: Chitotriosidase-1; LC-MS/MS: Liquid chromatography-tandem mass spectrometry; Iba-1: Ionised calcium binding adapter molecule-1; GFAP: Glial fibrillary acidic protein; NSC-34: Spinal cord motor neurons fused with neuroblastoma; iTRAQ: Isobaric tags for relative and absolute quantitation; MARS-14: Agilent’s multiple affinity removal system.

## Competing interests

The authors declare that they have no competing interests.

## Authors’ contributions

AMV collected samples, performed the experiments, analysed and wrote the manuscript. AS performed the experiments, analysed and wrote the manuscript. PM carried out microglial experiments, analysed and wrote the manuscript. VK analysed and wrote the manuscript. HHC supervised mass spectrometry, analysed and wrote the manuscript. TNS facilitated obtaining control CSF samples and critically evaluated manuscript. SB designed and supervised the experiments and critically reviewed the manuscript. NA enrolled patients with ALS, performed clinical evaluations and provided ALS-CSF. PAA designed, performed experiments, analyzed and wrote the manuscript. TRR conceptualized the project, obtained funding, supervised the study and critically reviewed the manuscript. All the authors read and approved the final manuscript.

## Supplementary Material

Additional file 1: Table S1 List of proteins identified in ALS-CSF. Description of Data: A list of the peptides and their fold changes of the 819 proteins identified in ALS-CSF using SEQUEST and Mascot. Click here for file

Additional file 2: Table S2 Up-regulated proteins in ALS-CSF. Description of data: Table showing 31 up-regulated proteins with more than 1.5-fold increase in ALS-CSF compared to normal CSF. Click here for file

Additional file 3: Table S3 Proteins down-regulated in ALS-CSF. Description of Data: List of 17 down-regulated proteins which showed a decrease of 0.5 fold or more, in ALS-CSF samples. Click here for file

## References

[B1] Wagner KR The need for biomarkers in amyotrophic lateral sclerosis drug development Neurology 2009 72 11 12 10.1212/01.wnl.0000338538.18938.9d 19122026

[B2] Shaw C What have cellular models taught us about ALS? Amyotroph Lateral Scler Other Motor Neuron Disord 2002 3 55 56 12215225 10.1080/146608202760196002

[B3] Anneser JM Chahli C Borasio GD Protective effect of metabotropic glutamate receptor inhibition on amyotrophic lateral sclerosis-cerebrospinal fluid toxicity in vitro Neuroscience 2006 141 1879 1886 10.1016/j.neuroscience.2006.05.044 16820266

[B4] Shahani N Nalini A Gourie-Devi M Raju TR Reactive astrogliosis in neonatal rat spinal cord after exposure to cerebrospinal fluid from patients with amyotrophic lateral sclerosis Exp Neurol 1998 149 295 298 10.1006/exnr.1997.6651 9454639

[B5] Shahani N Gourie-Devi M Nalini A Raju TR Cyclophosphamide attenuates the degenerative changes induced by CSF from patients with amyotrophic lateral sclerosis in the neonatal rat spinal cord J Neurol Sci 2001 185 109 118 10.1016/S0022-510X(01)00479-8 11311291

[B6] Shahani N Gourie-Devi M Nalini A Rammohan P Shobha K Harsha HN Raju TR: (-)-Deprenyl alleviates the degenerative changes induced in the neonatal rat spinal cord by CSF from amyotrophic lateral sclerosis patients Amyotroph Lateral Scler Other Motor Neuron Disord 2004 5 172 179 10.1080/14660820410017037 15512906

[B7] Shobha K Vijayalakshmi K Alladi PA Nalini A Sathyaprabha TN Raju TR Altered in-vitro and in-vivo expression of glial glutamate transporter-1 following exposure to cerebrospinal fluid of amyotrophic lateral sclerosis patients J Neurol Sci 2007 254 9 16 10.1016/j.jns.2006.12.004 17254611

[B8] Shobha K Alladi PA Nalini A Sathyaprabha TN Raju TR Exposure to CSF from sporadic amyotrophic lateral sclerosis patients induces morphological transformation of astroglia and enhances GFAP and S100beta expression Neurosci Lett 2010 473 56 61 10.1016/j.neulet.2010.02.022 20170712

[B9] Gunasekaran R Narayani RS Vijayalakshmi K Alladi PA Shobha K Nalini A Sathyaprabha TN Raju TR Exposure to cerebrospinal fluid of sporadic amyotrophic lateral sclerosis patients alters Nav1.6 and Kv1.6 channel expression in rat spinal motor neurons Brain Res 2009 1255 170 179 19109933 10.1016/j.brainres.2008.11.099

[B10] Deepa P Shahani N Alladi PA Vijayalakshmi K Sathyaprabha TN Nalini A Ravi V Raju TR Down regulation of trophic factors in neonatal rat spinal cord after administration of cerebrospinal fluid from sporadic amyotrophic lateral sclerosis patients J Neural Transm 2011 118 531 538 10.1007/s00702-010-0520-6 21069391

[B11] Vijayalakshmi K Alladi PA Ghosh S Prasanna VK Sagar BC Nalini A Sathyaprabha TN Raju TR Evidence of endoplasmic reticular stress in the spinal motor neurons exposed to CSF from sporadic amyotrophic lateral sclerosis patients Neurobiol Dis 2011 41 695 705 10.1016/j.nbd.2010.12.005 21168498

[B12] Sankaranarayani R Nalini A Rao Laxmi T Raju TR Altered neuronal activities in the motor cortex with impaired motor performance in adult rats observed after infusion of cerebrospinal fluid from amyotrophic lateral sclerosis patients Behav Brain Res 2010 206 109 119 10.1016/j.bbr.2009.09.009 19747511

[B13] Nagaraja TN Gourie-Devi M Nalini A Raju TR Neurofilament phosphorylation is enhanced in cultured chick spinal cord neurons exposed to cerebrospinal fluid from amyotrophic lateral sclerosis patients Acta Neuropathol 1994 88 349 352 10.1007/BF00310378 7839827

[B14] Sen I Nalini A Joshi NB Joshi PG Cerebrospinal fluid from amyotrophic lateral sclerosis patients preferentially elevates intracellular calcium and toxicity in motor neurons via AMPA/kainate receptor J Neurol Sci 2005 235 45 54 10.1016/j.jns.2005.03.049 15936037

[B15] Terro F Lesort M Viader F Ludolph A Hugon J Antioxidant drugs block in vitro the neurotoxicity of CSF from patients with amyotrophic lateral sclerosis Neuroreport 1996 7 1970 1972 8905705

[B16] Castellani RJ Siedlak SL Fortino AE Perry G Ghetti B Smith MA Chitin-like polysaccharides in Alzheimer’s disease brains Curr Alzheimer Res 2005 2 419 423 10.2174/156720505774330555 16248847

[B17] Sotgiu S Musumeci S Marconi S Gini B Bonetti B Different content of chitin-like polysaccharides in multiple sclerosis and Alzheimer’s disease brains J Neuroimmunol 2008 197 70 73 10.1016/j.jneuroim.2008.03.021 18485490

[B18] Aerts JM van Breemen MJ Bussink AP Ghauharali K Sprenger R Boot RG Groener JE Hollak CE Maas M Smit S Biomarkers for lysosomal storage disorders: identification and application as exemplified by chitotriosidase in Gaucher disease Acta Paediatr Suppl 2008 97 7 14 10.1111/j.1651-2227.2007.00641.x 18339181

[B19] Barone R Sotgiu S Musumeci S Plasma chitotriosidase in health and pathology Clin Lab 2007 53 321 333 17605408

[B20] Sotgiu S Arru G Soderstrom M Mameli G Serra C Dolei A Multiple sclerosis- associated retrovirus and optic neuritis Mult Scler 2006 12 357 359 10.1191/135248506ms1303sr 16764351

[B21] Renkema GH Boot RG Au FL Donker-Koopman WE Strijland A Muijsers AO Hrebicek M Aerts JM Chitotriosidase, a chitinase, and the 39-kDa human cartilage glycoprotein, a chitin-binding lectin, are homologues of family 18 glycosyl hydrolases secreted by human macrophages Eur J Biochem 1998 251 504 509 10.1046/j.1432-1327.1998.2510504.x 9492324

[B22] van Eijk M van Roomen CP Renkema GH Bussink AP Andrews L Blommaart EF Sugar A Verhoeven AJ Boot RG Aerts JM Characterization of human phagocyte-derived chitotriosidase, a component of innate immunity Int Immunol 2005 17 1505 1512 10.1093/intimm/dxh328 16214810

[B23] Castellani RJ Perry G Smith MA The role of novel chitin-like polysaccharides in Alzheimer disease Neurotox Res 2007 12 269 274 10.1007/BF03033910 18201954

[B24] Di Rosa M Dell’Ombra N Zambito AM Malaguarnera M Nicoletti F Malaguarnera L Chitotriosidase and inflammatory mediator levels in Alzheimer’s disease and cerebrovascular dementia Eur J Neurosci 2006 23 2648 2656 10.1111/j.1460-9568.2006.04780.x 16817867

[B25] Sotgiu S Piras MR Barone R Arru G Fois ML Rosati G Musumeci S Chitotriosidase and Alzheimer’s disease Curr Alzheimer Res 2007 4 295 296 10.2174/156720507781077232 17627486

[B26] Correale J Fiol M Chitinase effects on immune cell response in neuromyelitis optica and multiple sclerosis Mult Scler 2011 17 521 531 10.1177/1352458510392619 21159721

[B27] Di Rosa M Malaguarnera G De Gregorio C D’Amico F Mazzarino MC Malaguarnera L Modulation of chitotriosidase during macrophage differentiation Cell Biochem Biophys 2012 66 239 247 23152091 10.1007/s12013-012-9471-x

[B28] Sotgiu S Barone R Zanda B Arru G Fois ML Arru A Rosati G Marchetti B Musumeci S Chitotriosidase in patients with acute ischemic stroke Eur Neurol 2005 54 149 153 10.1159/000089935 16319488

[B29] Scorisa JM Duobles T Oliveira GP Maximino JR Chadi G The review of the methods to obtain non-neuronal cells to study glial influence on Amyotrophic Lateral Sclerosis pathophysiology at molecular level in vitro Acta Cir Bras 2010 25 281 289 20498942 10.1590/s0102-86502010000300011

[B30] Vijayalakshmi K Alladi PA Sathyaprabha TN Subramaniam JR Nalini A Raju TR Cerebrospinal fluid from sporadic amyotrophic lateral sclerosis patients induces degeneration of a cultured motor neuron cell line Brain Res 2009 1263 122 133 19368830 10.1016/j.brainres.2009.01.041

[B31] Xiang F Guo X Chen W Wang J Zhou T Huang F Cao C Chen X Proteomics analysis of human pericardial fluid Proteomics 2013 13 2692 2695 10.1002/pmic.201200317 23797974

[B32] Polisetty RV Gautam P Sharma R Harsha HC Nair SC Gupta MK Uppin MS Challa S Puligopu AK Ankathi P LC-MS/MS analysis of differentially expressed glioblastoma membrane proteome reveals altered calcium signaling and other protein groups of regulatory functions Mol Cell Proteomics 2012 11 1 15 10.1074/mcp.E112.019653 PMC3433906 22219345

