# Supplementary material for: Chitotriosidase - a putative biomarker for sporadic amyotrophic lateral sclerosis
Source: Clin Proteomics. 2013 Dec 2;10(1):19. doi: 10.1186/1559-0275-10-19 (PMC4220794; doi:10.1186/1559-0275-10-19)
Supplement: Additional file 1: Table S1 — List of proteins identified in ALS-CSF. Description of Data: A list of the peptides and their fold changes of the 819 proteins identified in ALS-CSF using SEQUEST and Mascot. [file 1559-0275-10-19-S1.pdf]

Accession	Gene Symbol	Protein name	Coverage	Unique Peptides	Peptides	PSMs	ALS/Control (Fold change)	MW [kDa]	Sequence	# PSMs	Modifications	ALS/Control (Fold change)	Xcorr (SEQUEST)	IonScore (MASCOT)	Charge	m/z
32171249	PTGDS	prostaglandin-H2 D-isomerase	69.47	12	12	419	0.932	21								
									AQGFTEDTIVFLPQTDK	40	N-Term(iTRAQ4plex); K17(iTRAQ4plex	0.965	7.23	99	2	1099.58
									SVVAPATDGGLNLTSTFLF	6	N-Term(iTRAQ4plex	0.973	5.85	91	2	1032.07
									TMLLQPAGSLGSYSYR	91	N-Term(iTRAQ4plex); M2(Oxidation	0.927	5.08	74	2	952.49
									TMLLQPAGSLGSYSYR	164	N-Term(iTRAQ4plex	0.931	5.05	89	2	944.49
									WFSAGLASNSSWLR	15	N-Term(iTRAQ4plex	0.956	4.88	94	2	863.45
									KAALSMCK	29	N-Term(iTRAQ4plex); K1(iTRAQ4plex); C7(Methylthio); K8(iTRAQ4plex)	0.89	4.55	55	3	443.92
									AALSMCK	36	N-Term(iTRAQ4plex); C6(Methylthio); K7(iTRAQ4plex)	0.859	3.33	53	2	529.28
									SPHWGSTYSVSVVETDYDQYALLYSQGS K	4	N-Term(iTRAQ4plex); K29(iTRAQ4plex	0.627		56		
									FTAFCK	28	N-Term(iTRAQ4plex); C5(Methylthio); K6(iTRAQ4plex)	1.078		41		
									MATLYSR	2	N-Term(iTRAQ4plex	0.907		38		
									GPGEDFR	2	N-Term(iTRAQ4plex	0.9		37		
									KNQCETR	1	N-Term(iTRAQ4plex); K1(iTRAQ4plex); C4(Methylthio)	1.191		33		
4507725	TTR	transthyretin precursor	68.71	7	7	339	0.941	15.9								
									ALGISPFHEHAEVVFTANDSGPF	66	N-Term(iTRAQ4plex	0.824	7.8	114	3	865.78
									GSPAINVAVHVFF	175	N-Term(iTRAQ4plex	1	6.02	94	3	504.29
									KAADDTWEPFASGK	5	N-Term(iTRAQ4plex); K1(iTRAQ4plex); K14(iTRAQ4plex)	1.001	5.97	87	2	978.02
									TSESGELHGLTTEEEFVEGIYK	8	N-Term(iTRAQ4plex); K22(iTRAQ4plex	0.927	5.81	90	3	915.13
									AADDTWEPFASGK	68	N-Term(iTRAQ4plex); K13(iTRAQ4plex	0.77	5.59	98	2	841.91
									RYTIAALLSPYSYSTTAVVTNPI	3	N-Term(iTRAQ4plex); K23(iTRAQ4plex	0.744		55		
									CPLMVK	14	N-Term(iTRAQ4plex); C1(Methylthio); K6(iTRAQ4plex)	0.81		42		
67191208	UBC	polyubiquitin-C	60.44	0	5	96		77								
									TITLEVEPSDTIENVK	20	N-Term(iTRAQ4plex); K16(iTRAQ4plex		5.99	89	2	1038.57
									LIFAGKQLEDGR	16	N-Term(iTRAQ4plex); K6(iTRAQ4plex		5	73	2	817.98
									TLSDYNIQK	22	N-Term(iTRAQ4plex); K9(iTRAQ4plex		4.71	67	2	685.38
									ESTLHLVLR	37	N-Term(iTRAQ4plex		4.63	58	2	606.36
									TLSDYNIQKESTLHLVLR	1	N-Term(iTRAQ4plex); K9(iTRAQ4plex			35		
11024714	UBB	polyubiquitin-B precursor	60.26	0	5	96		25.7								
									TITLEVEPSDTIENVK	20	N-Term(iTRAQ4plex); K16(iTRAQ4plex		5.99	89	2	1038.57
									LIFAGKQLEDGR	16	N-Term(iTRAQ4plex); K6(iTRAQ4plex		5	73	2	817.98
									TLSDYNIQK	22	N-Term(iTRAQ4plex); K9(iTRAQ4plex		4.71	67	2	685.38
									ESTLHLVLR	37	N-Term(iTRAQ4plex		4.63	58	2	606.36
									TLSDYNIQKESTLHLVLR	1	N-Term(iTRAQ4plex); K9(iTRAQ4plex			35		
4557325	APOE	apolipoprotein E precursor	59.31	16	16	404	0.86	36.1								
									AATVGSLAGQPLQER	62	N-Term(iTRAQ4plex	0.792	6.63	98	2	821.46
									VQAAVGTSAAPVPSDNI	43	N-Term(iTRAQ4plex	0.982	6.58	109	2	882.96
									SELEEQLTPVAEETR	74	N-Term(iTRAQ4plex	0.796	6.26	96	2	937.98
									VEQAVETEPEPELR	14	N-Term(iTRAQ4plex	1.103	5.81	94	2	885.45
									GEVQAMLGQSTEELR	27	N-Term(iTRAQ4plex	0.928	5.72	95	2	896.46
									SWFEPLVEDMQR	35	N-Term(iTRAQ4plex	0.861	5.06	66	2	840.91
									GEVQAMLGQSTEELR	3	N-Term(iTRAQ4plex); M6(Oxidation	0.833	4.8	83	2	904.45
									LGADMEDVCGR	49	N-Term(iTRAQ4plex); C9(Methylthio	0.831	4.38	67	2	678.30
									LEEQAQQIR	5	N-Term(iTRAQ4plex	0.83	4.15	49	2	629.85
									ALMDETMK	20	N-Term(iTRAQ4plex); K8(iTRAQ4plex	0.977	3.98	48	2	613.82
									SWFEPLVEDMQR	3	N-Term(iTRAQ4plex); M10(Oxidation	0.913	3.93	58	2	848.91
									QWAGLVEK	41	N-Term(iTRAQ4plex); K8(iTRAQ4plex	1.011	3.84	44	2	609.86
									LQAEAFQAR	5	N-Term(iTRAQ4plex	0.756	3.75	39	2	589.32
									LGPLVEQGR	10	N-Term(iTRAQ4plex	0.715	3.38	52	2	556.83
									LAVYQAGAR	3	N-Term(iTRAQ4plex	0.76	3.28	47	2	546.82
									LGADMEDVCGR	6	N-Term(iTRAQ4plex); M5(Oxidation); C9(Methylthio)	0.833	3.28	50	2	686.30
									WVQTLSEQVQEELLSSQVTQELR	1	N-Term(iTRAQ4plex	1.104		48		
									WELALGR	2	N-Term(iTRAQ4plex	0.881		42		
									EQVAEVR	1	N-Term(iTRAQ4plex	0.869		41		
61744426	KLK6	kallikrein-6 isoform B	59.12	0	6	176		15								
									DSCQGDSGGPLVCGDHLR	52	N-Term(iTRAQ4plex); C3(Methylthio); C13(Methylthio)		7.31	118	3	684.62
									EKPGVYTNVCR	24	N-Term(iTRAQ4plex); K2(iTRAQ4plex); C10(Methylthio)		5.31	75	3	533.95
									GLVSWGNIPCGSK	40	N-Term(iTRAQ4plex); C10(Methylthio); K13(iTRAQ4plex)		4.98	76	2	826.43
									LSELIQPLPLER	23	N-Term(iTRAQ4plex		4.76	63	2	776.47
									TADGDFPDTIQCAYIHLVSF	15	N-Term(iTRAQ4plex); C12(Methylthio		4.65	70	3	804.72
									YTNWIQK	22	N-Term(iTRAQ4plex); K7(iTRAQ4plex		3.56	51	2	620.84
71773110	APOA4	apolipoprotein A-IV precursor	51.26	19	19	231	0.966	45.3								
									LNHQLEGLTFQMK	18	N-Term(iTRAQ4plex); K13(iTRAQ4plex	1.024	6.82	80	3	616.34
									SLAELGGHLDQQVEEFR	19	N-Term(iTRAQ4plex	0.938	6.8	102	3	691.36
									RVEPYGENFNK	5	N-Term(iTRAQ4plex); K11(iTRAQ4plex	1.072	6.53	39	3	547.63
									LGEVNTYAGDLQK	11	N-Term(iTRAQ4plex); K13(iTRAQ4plex	0.97	6.4	84	2	848.46
									ENADSLQASLRPHADELK	5	N-Term(iTRAQ4plex); K18(iTRAQ4plex	1.04	5.76	36	4	571.31
									LGPHAGDVEGHLSFLEK	13	N-Term(iTRAQ4plex); K17(iTRAQ4plex	1.022	5.55	82	3	698.71
									LNHQLEGLTFQMK	3	N-Term(iTRAQ4plex); M12(Oxidation); K13(iTRAQ4plex)	0.886	5.33	47	3	621.67
									LVPFATELHER	15	N-Term(iTRAQ4plex	0.891	5.1	56	3	485.94
									IDQNVEELK	18	N-Term(iTRAQ4plex); K9(iTRAQ4plex	0.983	4.95	76	2	688.39
									ALVQQMEQLR	8	N-Term(iTRAQ4plex	0.993	4.49	58	2	680.38
									IDQTVEELR	22	N-Term(iTRAQ4plex	0.888	4.45	60	2	623.84
									VEPYGENFNK	8	N-Term(iTRAQ4plex); K10(iTRAQ4plex	1.051	4.29	75	2	742.88
									SLAPYAQDTQEK	15	N-Term(iTRAQ4plex); K12(iTRAQ4plex	0.941	4.23	81	2	819.94
									VNSFFSTFK	10	N-Term(iTRAQ4plex); K9(iTRAQ4plex	1.018	4.11	76	2	682.87
									LEPYADQLR	24	N-Term(iTRAQ4plex	1.057	4.01	53	2	624.84
									LTPYADEFK	28	N-Term(iTRAQ4plex); K9(iTRAQ4plex	0.812	3.94	76	2	686.37
									SELTQQLNALFQDK	2	N-Term(iTRAQ4plex); K14(iTRAQ4plex	0.901	3.61	49	2	962.03
									LLPHANEVSQK	3	N-Term(iTRAQ4plex); K11(iTRAQ4plex	0.999	3.48	59	3	508.64
									LAPLAEDVR	3	N-Term(iTRAQ4plex	0.96	3.45	36	2	564.33
									DKVNSFFSTFK	1	N-Term(iTRAQ4plex); K2(iTRAQ4plex); K11(iTRAQ4plex)	0.781		46		

166197660	C8G	complement component C8 gamma chain precursor	50.5	7	7	31	0.901	22.3								
									VQEAHLTEDQIFYFPK	11	N-Term(iTRAQ4plex); K16(iTRAQ4plex	0.838	6.33	61	3	751.73
									RPASPISTIQPK	6	N-Term(iTRAQ4plex); K12(iTRAQ4plex	0.791	4.94	42	3	528.32
									SLPVSDSVLSGFEQR	6	N-Term(iTRAQ4plex	0.934	4.69	86	2	882.97
									AEATTLHVAPQGTAMAVSTFI	4	N-Term(iTRAQ4plex	0.974	3.64	44	3	768.40
									KLDGICWQVR	2	N-Term(iTRAQ4plex); K1(iTRAQ4plex); C6(Methylthio)	1.052		77		
									YGFCEAADQFHVLDEVF	1	N-Term(iTRAQ4plex); C4(Methylthio	0.822		33		
4506155	KLK6	kallikrein-6 isoform A preproprotein	49.18	3	9	254	1.048	26.8								
									AVIHPDYDAASHDQDIMLLF	37	N-Term(iTRAQ4plex	1.019	9.25	94	3	808.74
									DSCQGDSGGPLVCGDHLR	52	N-Term(iTRAQ4plex); C3(Methylthio); C13(Methylthio)		7.31	118	3	684.62
									EKPGVYTNVCF	24	N-Term(iTRAQ4plex); K2(iTRAQ4plex); C10(Methylthio)		5.31	75	3	533.95
									KPNLQVFLGK	33	N-Term(iTRAQ4plex); K1(iTRAQ4plex); K10(iTRAQ4plex)	1.092	5.22	60	3	526.00
									AVIHPDYDAASHDQDIMLLF	7	N-Term(iTRAQ4plex); M17(Oxidation	1.021	5.15	73	3	814.07
									GLVSWGNIPCGSK	40	N-Term(iTRAQ4plex); C10(Methylthio); K13(iTRAQ4plex)		4.98	76	2	826.43
									LSELIQPLPLER	23	N-Term(iTRAQ4plex		4.76	63	2	776.47
									TADGDFPDTIQCAYIHLVSF	15	N-Term(iTRAQ4plex); C12(Methylthio		4.65	70	3	804.72
									YTNWIQK	22	N-Term(iTRAQ4plex); K7(iTRAQ4plex		3.56	51	2	620.84
									LVHGGPCDK	1	N-Term(iTRAQ4plex); C7(Methylthio); K9(iTRAQ4plex)	0.912		48		
4505621	PEBP1	phosphatidylethanolamine-binding protein 1	48.13	7	7	53	0.876	21								
									GNDISSGTVLSDYVGSGPPK	8	N-Term(iTRAQ4plex); K20(iTRAQ4plex	0.871	8.19	111	2	1119.58
									NRPTSISWDGLDSGK	15	N-Term(iTRAQ4plex); K15(iTRAQ4plex	0.872	5.27	74	3	641.01
									EWHHFLVVNMK	6	N-Term(iTRAQ4plex); K11(iTRAQ4plex	0.921	4.82	61	3	576.65
									YVWLVYEQDRPLK	5	N-Term(iTRAQ4plex); K13(iTRAQ4plex	0.807	4.5	58	3	666.37
									EWHHFLVVNMK	2	N-Term(iTRAQ4plex); M10(Oxidation); K11(iTRAQ4plex)	0.889	4.34	48	3	581.98
									LYTLVLTDPDAPSR	12	N-Term(iTRAQ4plex	0.844	4.3	77	2	852.97
									CDEPILSNR	3	N-Term(iTRAQ4plex); C1(Methylthio	0.97	3.43	34	2	618.80
									VLTPTQVK	2	N-Term(iTRAQ4plex); K8(iTRAQ4plex	0.919		58		
4502261	SERPINC1	antithrombin-III precursor	47.84	18	18	354	1.045	52.6								
									ADGESCSASMMYQEGK	25	N-Term(iTRAQ4plex); C6(Methylthio); K16(iTRAQ4plex)	1.111	7.25	140	2	1014.42
									AFLEVNEEGSEAAASTAVVIAGI	27	N-Term(iTRAQ4plex	0.73	6.28	90	3	812.43
									FATTFYQHLADSK	35	N-Term(iTRAQ4plex); K13(iTRAQ4plex	1.124	6.03	103	2	908.98
									DDLYVSDAFHK	44	N-Term(iTRAQ4plex); K11(iTRAQ4plex	0.993	6.03	85	3	533.27
									EQLQDMGLVDLFSPEK	11	N-Term(iTRAQ4plex); K16(iTRAQ4plex	1.08	6	96	2	1069.06
									VAEGTQVLELPFK	42	N-Term(iTRAQ4plex); K13(iTRAQ4plex	1.088	5.96	90	2	860.00
									GDDITMVLILPKPEK	13	N-Term(iTRAQ4plex); K12(iTRAQ4plex); K15(iTRAQ4plex)	0.945	5.9	72	2	1051.11
									TSDQIHFFFAK	32	N-Term(iTRAQ4plex); K11(iTRAQ4plex	1.111	5.69	74	2	814.94
									SKLPGIVAEGR	10	N-Term(iTRAQ4plex); K2(iTRAQ4plex	1.102	4.75	62	3	472.29
									NDNDNIFLSPLSISTAFAMTK	2	N-Term(iTRAQ4plex); K21(iTRAQ4plex	1.037	4.44	91	2	1294.17
									DIPMNPMCIYR	6	N-Term(iTRAQ4plex); C8(Methylthio	1.115	4.23	40	2	771.86
									EVPLNTIIFMGR	39	N-Term(iTRAQ4plex	1.041	4.1	73	2	767.43
									EQLQDMGLVDLFSPEK	3	N-Term(iTRAQ4plex); M6(Oxidation); K16(iTRAQ4plex)	0.963	3.97	54	2	1077.06
									ADGESCSASMMYQEGK	4	N-Term(iTRAQ4plex); C6(Methylthio); M10(Oxidation); K16(iTRAQ4plex)	0.927	3.89	78	2	1022.42
									ANRPFLVFIR	13	N-Term(iTRAQ4plex	0.985	3.67	42	3	459.62
									EVPLNTIIFMGR	3	N-Term(iTRAQ4plex); M10(Oxidation	0.992	3.63	37	2	775.44
									IEDGFSLK	36	N-Term(iTRAQ4plex); K8(iTRAQ4plex	1.075	3.59	50	2	598.84
									RVWELSK	3	N-Term(iTRAQ4plex); K7(iTRAQ4plex	0.968	3.52	48	3	402.58
									ATEDEGSEQKIPEATNF	1	N-Term(iTRAQ4plex); K10(iTRAQ4plex	1.071		53		
									LPGIVAEGR	4	N-Term(iTRAQ4plex	1.042		46		
									LQPLDFK	1	N-Term(iTRAQ4plex); K7(iTRAQ4plex	0.864		39		
4502027	ALB	serum albumin preproprotein	47.13	26	30	354	0.296	69.3								
									KVPQVSTPTLVEVSF	37	N-Term(iTRAQ4plex); K1(iTRAQ4plex		7.14	104	3	643.39
									RPCFSALEVDETYVPK	25	N-Term(iTRAQ4plex); C3(Methylthio); K16(iTRAQ4plex)	0.432	6.97	77	3	730.04
									AVMDDFAAFVEK	22	N-Term(iTRAQ4plex); K12(iTRAQ4plex	0.213	6.23	109	2	815.93
									SHCIAEVENDEMPADLPSLAADFVESK	2	N-Term(iTRAQ4plex); C3(Methylthio); K27(iTRAQ4plex)	0.279	6.04	81	3	1084.51
									AAFTECCQAADK	12	N-Term(iTRAQ4plex); C6(Methylthio); C7(Methylthio); K12(iTRAQ4plex)	0.281	5.69	103	2	819.36
									QNCELFEQLGEYK	6	N-Term(iTRAQ4plex); C3(Methylthio); K13(iTRAQ4plex)	0.281	5.62	91	2	967.97
									YICENQDSISSK	20	N-Term(iTRAQ4plex); C3(Methylthio); K12(iTRAQ4plex)	0.249	5.32	99	2	860.91
									LVNEVTEFAK	35	N-Term(iTRAQ4plex); K10(iTRAQ4plex	0.34	4.92	76	2	719.41
									VPQVSTPTLVEVSF	9	N-Term(iTRAQ4plex		4.89	69	2	828.48
									ETYGEMADCCAK	8	N-Term(iTRAQ4plex); C9(Methylthio); C10(Methylthio); K12(iTRAQ4plex)	0.452	4.88	89	2	850.84
									AVMDDFAAFVEK	2	N-Term(iTRAQ4plex); M3(Oxidation); K12(iTRAQ4plex)	0.516	4.48	107	2	823.93
									FKDLGEENFK	9	N-Term(iTRAQ4plex); K2(iTRAQ4plex); K10(iTRAQ4plex)	0.359	4.36	55	3	553.65
									CCTESLVNR	14	N-Term(iTRAQ4plex); C1(Methylthio); C2(Methylthio)		4.27	46	2	630.77
									KQTALVELVK	13	N-Term(iTRAQ4plex); K1(iTRAQ4plex); K10(iTRAQ4plex)	0.335	4.22	70	3	521.01
									SLHTLFGDK	10	N-Term(iTRAQ4plex); K9(iTRAQ4plex	0.337	4.13	53	2	653.38
									QTALVELVK	40	N-Term(iTRAQ4plex); K9(iTRAQ4plex	0.423	3.87	65	2	644.91
									ADDKETCFAEEGK	5	N-Term(iTRAQ4plex); K4(iTRAQ4plex); C7(Methylthio); K13(iTRAQ4plex)	0.225	3.84	70	3	640.98
									KYLYEIAR	6	N-Term(iTRAQ4plex); K1(iTRAQ4plex	0.276	3.83	49	3	448.61
									HPDYSVVLLLR	14	N-Term(iTRAQ4plex	0.455	3.8	64	3	485.95
									DLGEENFK	7	N-Term(iTRAQ4plex); K8(iTRAQ4plex	0.409	3.8	41	2	620.33
									FQNALLVR	13	N-Term(iTRAQ4plex	0.274	3.79	42	2	552.84
									TCVADESAENCDK	2	N-Term(iTRAQ4plex); C2(Methylthio); C11(Methylthio); K13(iTRAQ4plex)	0.243	3.7	62	2	882.87
									KLVAASQAALGL	2	N-Term(iTRAQ4plex); K1(iTRAQ4plex	0.984	3.58	38	2	715.45

									LVAASQAALGL	10	N-Term(iTRAQ4plex	0.227	3.57	83	2	579.36
									LCTVATLR	19	N-Term(iTRAQ4plex); C2(Methylthio	0.22	3.42	46	2	533.80
									LDELRDEGK	1	N-Term(iTRAQ4plex); K9(iTRAQ4plex	0.212		52		
									RHPDYSVVLLLR	1	N-Term(iTRAQ4plex			52		
									AEFAEVSK	2	N-Term(iTRAQ4plex); K8(iTRAQ4plex	0.209		47		
									TYETTLEK	1	N-Term(iTRAQ4plex); K8(iTRAQ4plex	0.262		43		
									LVTDLTK	3	N-Term(iTRAQ4plex); K7(iTRAQ4plex			40		
									HPYFYAPELLFFAK	4	N-Term(iTRAQ4plex); K14(iTRAQ4plex	0.339		38		
11321561	HPX	hemopexin precursor	46.75	17	17	489	1.024	51.6								
									CSPHLVLSALTSDNHGATYAFSGTHYWF	3	N-Term(iTRAQ4plex); C1(Methylthio	1.344	8.28	41	5	657.12
									WKNFPSPVDAAFR	7	N-Term(iTRAQ4plex); K2(iTRAQ4plex	1.111	6.55	62	3	608.33
									GECQAEGVLFFQGDR	56	N-Term(iTRAQ4plex); C3(Methylthio	1.018	6.27	98	2	923.43
									SGAQATWTELPWPHEK	52	N-Term(iTRAQ4plex); K16(iTRAQ4plex	0.941	6.22	72	3	709.37
									LLQDEFPGIPSPLDAAVECHR	43	N-Term(iTRAQ4plex); C19(Methylthio	1.155	5.7	66	3	833.09
									LYLVQGTQVYVFLTK	11	N-Term(iTRAQ4plex); K15(iTRAQ4plex	1.021	5.58	78	2	1030.61
									EWFWDLATGTMK	39	N-Term(iTRAQ4plex); K12(iTRAQ4plex	1.066	5.4	77	2	886.95
									GGYTLVSGYPK	42	N-Term(iTRAQ4plex); K11(iTRAQ4plex	1.183	5.29	77	2	715.40
									EVGTPHGIILDSVDAAFICPGSSF	22	N-Term(iTRAQ4plex); C19(Methylthio	0.966	5.05	116	3	877.77
									YYCFQGNQFLR	28	N-Term(iTRAQ4plex); C3(Methylthio	1.033	4.4	52	2	814.88
									VDGALCMEK	16	N-Term(iTRAQ4plex); C6(Methylthio); K9(iTRAQ4plex)	1.087	4.35	57	2	650.32
									NFPSPVDAAFR	68	N-Term(iTRAQ4plex	0.975	4.2	62	2	682.86
									EWFWDLATGTMK	7	N-Term(iTRAQ4plex); M11(Oxidation); K12(iTRAQ4plex)	1.222	3.95	60	2	894.95
									ALPQPQNVTSLLGCTH	3	N-Term(iTRAQ4plex); C14(Methylthio	1.178	3.9	72	2	934.98
									DYFMPCPGR	29	N-Term(iTRAQ4plex); C6(Methylthio	1.029	3.66	42	2	638.28
									RLWWLDLK	8	N-Term(iTRAQ4plex); K8(iTRAQ4plex	0.9	3.41	49	3	473.29
									VWVYPPEK	15	N-Term(iTRAQ4plex); K8(iTRAQ4plex	0.973	3.25	42	2	653.38
									LWWLDLK	19	N-Term(iTRAQ4plex); K7(iTRAQ4plex	1.081	3.21	40	2	631.38
									VDGALCMEK	11	N-Term(iTRAQ4plex); C6(Methylthio); M7(Oxidation); K9(iTRAQ4plex)	0.901	3.18	47	2	658.31
									DYFMPCPGR	10	N-Term(iTRAQ4plex); M4(Oxidation); C6(Methylthio)	0.947		41		
5453678	NPC2	epididymal secretory protein E1 precursor	45.7	5	5	44	1.159	16.6								
									AVVHGILMGVPVPFPIPEPDGCK	6	N-Term(iTRAQ4plex); C22(Methylthio); K23(iTRAQ4plex)	0.829	6.91	48	3	902.82
									LVVEWQLQDDK	15	N-Term(iTRAQ4plex); K11(iTRAQ4plex	1.361	5.25	87	2	830.96
									DCGSVDGVIK	16	N-Term(iTRAQ4plex); C2(Methylthio); K10(iTRAQ4plex)	1.188	4.61	62	2	663.84
									EVNVSPCPTQPCQLSK	2	N-Term(iTRAQ4plex); C7(Methylthio); C12(Methylthio); K16(iTRAQ4plex)	1.338	4.54	64	2	1055.51
									SGINCPIQK	5	N-Term(iTRAQ4plex); C5(Methylthio); K9(iTRAQ4plex)	1.015	4.13	43	2	647.35
91206462	SPP1	osteopontin isoform a precursor	45.54	1	10	144	1.758	35.4								
									DSYETSQLDDQSAETHSHK	15	N-Term(iTRAQ4plex); K19(iTRAQ4plex		9.51	110	3	822.71
									GKDSYETSQLDDQSAETHSHK	3	N-Term(iTRAQ4plex); K2(iTRAQ4plex); K21(iTRAQ4plex)		7.7	43	5	559.87
									ANDESNEHSDVIDSQELSK	25	N-Term(iTRAQ4plex); K19(iTRAQ4plex		7.4	98	3	802.38
									KANDESNEHSDVIDSQELSK	19	N-Term(iTRAQ4plex); K1(iTRAQ4plex); K20(iTRAQ4plex)		7.25	94	3	893.11
									YPDAVATWLNPDPSQK	8	N-Term(iTRAQ4plex); K16(iTRAQ4plex		5.82	73	2	1045.54
									AIPVAQDLNAPSDWDSR	32	N-Term(iTRAQ4plex		5.77	88	2	1000.00
									ISHELDSASSEVN	37	N-Term(iTRAQ4plex		4.77	55	2	766.37
									GDSVVYGLR	2	N-Term(iTRAQ4plex		3.86	39	2	555.30
									QNLLAPQNAVSSEETNDFK	2	N-Term(iTRAQ4plex); K19(iTRAQ4plex	1.758	3.68	75	2	1197.11
62739188	CFH	complement factor H isoform b precursor	45.43	0	16	72		51								
									GDAVCTESGWRPLPSCEEK	3	N-Term(iTRAQ4plex); C5(Methylthio); C16(Methylthio); K19(iTRAQ4plex)		6.06	36	3	815.37
									SIDVACHPGYALPK	6	N-Term(iTRAQ4plex); C6(Methylthio); K14(iTRAQ4plex)		6.04	44	2	902.97
									KGEWVALNPLR	12	N-Term(iTRAQ4plex); K1(iTRAQ4plex		5.42	69	3	524.32
									AQTTVTCMENGWSPTPR	2	N-Term(iTRAQ4plex); C7(Methylthio		4.88	55	2	1034.97
									SLGNVIMVCR	2	N-Term(iTRAQ4plex); C9(Methylthio		4.52	63	2	641.34
									CNMGYEYSER	9	N-Term(iTRAQ4plex); C1(Methylthio		4.42	48	2	721.29
									CTSTGWIPAPR	10	N-Term(iTRAQ4plex); C1(Methylthio		4.23	61	2	689.84
									GEWVALNPLR	6	N-Term(iTRAQ4plex		4.16	44	2	649.87
									TGDEITYQCR	2	N-Term(iTRAQ4plex); C9(Methylthio		4	35	2	688.31
									RPYFPVAVGK	13	N-Term(iTRAQ4plex); K10(iTRAQ4plex		3.95	58	3	474.62
									SPDVINGSPISQK	2	N-Term(iTRAQ4plex); K13(iTRAQ4plex		3.76	38	2	815.46
									FVCNSGYK	3	N-Term(iTRAQ4plex); C3(Methylthio); K8(iTRAQ4plex)		3.33	50	2	626.31
									CVEISCK	4	N-Term(iTRAQ4plex); C1(Methylthio); C6(Methylthio); K7(iTRAQ4plex)		3.23	44	2	581.28
									ECDTDGWTNDIPICEVVK	1	N-Term(iTRAQ4plex); C2(Methylthio); C14(Methylthio); K18(iTRAQ4plex)			48		
									RPCGHPGDTPFGTFTLTGGNVFEYGVK	1	N-Term(iTRAQ4plex); C3(Methylthio); K27(iTRAQ4plex)			38		
4759166	SPP1	osteopontin isoform b precursor	45.33	1	10	158	2.768	33.8								
									DSYETSQLDDQSAETHSHK	15	N-Term(iTRAQ4plex); K19(iTRAQ4plex		9.51	110	3	822.71
									GKDSYETSQLDDQSAETHSHK	3	N-Term(iTRAQ4plex); K2(iTRAQ4plex); K21(iTRAQ4plex)		7.7	43	5	559.87
									ANDESNEHSDVIDSQELSK	25	N-Term(iTRAQ4plex); K19(iTRAQ4plex		7.4	98	3	802.38
									KANDESNEHSDVIDSQELSK	19	N-Term(iTRAQ4plex); K1(iTRAQ4plex); K20(iTRAQ4plex)		7.25	94	3	893.11
									YPDAVATWLNPDPSQK	8	N-Term(iTRAQ4plex); K16(iTRAQ4plex		5.82	73	2	1045.54
									AIPVAQDLNAPSDWDSR	32	N-Term(iTRAQ4plex		5.77	88	2	1000.00
									QNLLAPQTLPSK	16	N-Term(iTRAQ4plex); K12(iTRAQ4plex	2.768	4.8	72	2	799.48
									ISHELDSASSEVN	37	N-Term(iTRAQ4plex		4.77	55	2	766.37
									GDSVVYGLR	2	N-Term(iTRAQ4plex		3.86	39	2	555.30
4502337	AZGP1	zinc-alpha-2-glycoprotein precursor	44.63	13	13	180	1.191	34.2								
									QDPPSVVVTSHQAPGEK	26	N-Term(iTRAQ4plex); K17(iTRAQ4plex	1.261	6.42	70	3	688.71
									AREDIFMETLK	14	N-Term(iTRAQ4plex); K11(iTRAQ4plex	1.2	6.08	51	3	547.64
									QKWEAEPVYVQR	4	N-Term(iTRAQ4plex); K2(iTRAQ4plex	1.253	5.4	63	2	911.00
									HVEDVPAFQALGSLNDLQFFR	9	N-Term(iTRAQ4plex	1.137	4.79	60	3	849.78
									EIPAWVPFDPAAQITK	9	N-Term(iTRAQ4plex); K16(iTRAQ4plex	1.123	4.71	60	2	1036.08
									AYLEEECPATLR	25	N-Term(iTRAQ4plex); C7(Methylthio	0.98	4.61	63	2	792.88
									QVEGMEDWK	21	N-Term(iTRAQ4plex); K9(iTRAQ4plex	1.439	4.5	55	2	705.35

									YSLTYIYTGLSK	7	N-Term(iTRAQ4plex); K12(iTRAQ4plex	1.322	4.33	80	2	848.98
									EDIFMETLK	12	N-Term(iTRAQ4plex); K9(iTRAQ4plex	1.209	4.22	56	2	707.38
									WEAEPVYVQR	24	N-Term(iTRAQ4plex	1.107	4.16	49	2	710.87
									CLAYDFYPGK	21	N-Term(iTRAQ4plex); C1(Methylthio); K10(iTRAQ4plex)	1.111	3.99	58	2	755.87
									DYIEFNK	6	N-Term(iTRAQ4plex); K7(iTRAQ4plex	1.527	3.43	40	2	608.83
									AREDIFMETLK	1	N-Term(iTRAQ4plex); M7(Oxidation); K11(iTRAQ4plex)	0.984		45		
									YYYDGKDYIEFNK	1	N-Term(iTRAQ4plex); K6(iTRAQ4plex); K13(iTRAQ4plex)	1.614		38		
4504619	IGFBP7	insulin-like growth factor-binding protein 7	44.33	8	8	97	0.937	29.1								
									AGAAAGGPGVSGVCVCK	5	N-Term(iTRAQ4plex); C14(Methylthio); C16(Methylthio); K17(iTRAQ4plex)	0.909	7.43	137	2	892.43
									ITVVDALHEIPVK	37	N-Term(iTRAQ4plex); K13(iTRAQ4plex	0.928	6.31	97	2	861.53
									HEVTGWVLVSPLSK	13	N-Term(iTRAQ4plex); K14(iTRAQ4plex	0.921	5.9	98	2	920.53
									EDAGEYECHASNSQGQASASAK	5	N-Term(iTRAQ4plex); C8(Methylthio); K22(iTRAQ4plex)	0.954	5.63	115	3	858.71
									GTCEQGPSIVTPPK	25	N-Term(iTRAQ4plex); C3(Methylthio); K14(iTRAQ4plex)	0.964	5.22	92	2	874.45
									GYCAPGMECVK	2	N-Term(iTRAQ4plex); C3(Methylthio); C9(Methylthio); K11(iTRAQ4plex)	0.874	4.54	49	2	769.33
									TELLPGDRDNLAIQTR	8	N-Term(iTRAQ4plex	1.025	4.38	48	2	978.54
									YPVCGSDGTTYPSGCQLR	2	N-Term(iTRAQ4plex); C4(Methylthio); C15(Methylthio)	1.045	3.33	65	2	1070.46
39725934	SERPINF1	pigment epithelium-derived factor precursor	44.26	15	15	378	0.76	46.3								
									ALYYDLISSPDIHGTYK	34	N-Term(iTRAQ4plex); K17(iTRAQ4plex	0.75	7.43	81	3	748.73
									KTSLEDFYLDEER	31	N-Term(iTRAQ4plex); K1(iTRAQ4plex	0.728	6.47	92	3	645.00
									SSTSPTTNVLLSPLSVATALSALSLGAEQR	3	N-Term(iTRAQ4plex	0.745	6	81	3	1039.24
									DTDTGALLFIGK	30	N-Term(iTRAQ4plex); K12(iTRAQ4plex	0.751	5.99	93	2	769.94
									LAAAVSNFGYDLYR	41	N-Term(iTRAQ4plex	0.796	5.7	87	2	852.45
									TSLEDFYLDEER	11	N-Term(iTRAQ4plex	0.758	5.6	72	2	830.90
									LQSLFDSPDFSK	48	N-Term(iTRAQ4plex); K12(iTRAQ4plex	0.734	5.4	113	2	836.45
									LDLQEINNWVQAQMK	17	N-Term(iTRAQ4plex); K15(iTRAQ4plex	0.738	5.32	88	2	1059.57
									ELLDTVTAPQK	35	N-Term(iTRAQ4plex); K11(iTRAQ4plex	0.8	5.01	80	2	751.94
									YGLDSDLSCK	38	N-Term(iTRAQ4plex); C9(Methylthio); K10(iTRAQ4plex)	0.769	4.8	63	2	717.84
									EIPDEISILLLGVAHFK	16	N-Term(iTRAQ4plex); K17(iTRAQ4plex	0.723	4.69	64	3	728.10
									TVQAVLTVPK	20	N-Term(iTRAQ4plex); K10(iTRAQ4plex	0.889	4.46	64	2	672.42
									LSYEGEVTK	24	N-Term(iTRAQ4plex); K9(iTRAQ4plex	0.904	4.4	65	2	657.36
									SSFVAPLEK	29	N-Term(iTRAQ4plex); K9(iTRAQ4plex	0.76	4.05	59	2	633.37
									GQWVTK	1	N-Term(iTRAQ4plex); K6(iTRAQ4plex	0.809		42		
4502805	CHGA	chromogranin-A preproprotein	43.98	12	12	146	0.728	50.7								
									SEALAVDGAGKPGAEEAQDPEGK	6	N-Term(iTRAQ4plex); K11(iTRAQ4plex); K23(iTRAQ4plex)	0.849	6.36	59	3	886.79
									HSGFEDELSEVLENQSSQAELK	10	N-Term(iTRAQ4plex); K22(iTRAQ4plex	0.661	6.15	78	3	922.13
									YPGPQAEGDSEGLSQGLVDR	48	N-Term(iTRAQ4plex	0.712	6.1	112	2	1110.04
									EEEEEMAVVPQGLFR	12	N-Term(iTRAQ4plex	0.844	5.98	59	2	953.97
									SGEATDGARPQALPEPMQESK	15	N-Term(iTRAQ4plex); K21(iTRAQ4plex	0.84	5.68	64	3	829.76
									ELQDLALQGAK	8	N-Term(iTRAQ4plex); K11(iTRAQ4plex	0.907	5.62	84	2	737.43
									GLSAEPGWQAK	4	N-Term(iTRAQ4plex); K11(iTRAQ4plex	1.062	5.49	77	2	716.39
									EDSLEAGLPLQVR	11	N-Term(iTRAQ4plex	0.85	5.35	75	2	785.93
									AEGNNQAPGEEEEEEEEATNTHPPASLPSQK	13	N-Term(iTRAQ4plex); K31(iTRAQ4plex	0.73	5.07	58	3	1203.22
									GEQEHSQQKEEEEEMAVVPQGLFR	7	N-Term(iTRAQ4plex); K9(iTRAQ4plex	0.721	4.63	66	3	1034.84
									RPEDQELESLSAIEAELEK	7	N-Term(iTRAQ4plex); K19(iTRAQ4plex	0.499		82		
									EEEEEMAVVPQGLFR	1	N-Term(iTRAQ4plex); M6(Oxidation	0.709		71		
									ILSILR	4	N-Term(iTRAQ4plex	0.701		35		
21071039	CNDP1	beta-Ala-His dipeptidase precursor	43.2	18	18	390	0.828	56.7								
									VFQYIDLHQDEFVQTLK	21	N-Term(iTRAQ4plex); K17(iTRAQ4plex	0.832	7.9	97	3	804.44
									SVVLIPLGAVDDGEHSQNEK	21	N-Term(iTRAQ4plex); K20(iTRAQ4plex	0.821	6.91	74	3	799.09
									GTVCFYGHLDVQPADR	72	N-Term(iTRAQ4plex); C4(Methylthio	0.76	6.83	87	3	656.65
									GNSYFMVEVK	22	N-Term(iTRAQ4plex); K10(iTRAQ4plex	0.794	5.58	71	2	731.38
									ALEQDLPVNIK	38	N-Term(iTRAQ4plex); K11(iTRAQ4plex	0.822	5.51	79	2	764.45
									GDGWLTDPYVLTEVDGK	11	N-Term(iTRAQ4plex); K17(iTRAQ4plex	0.673	5.43	87	2	1077.06
									EWVAIESDSVQPVPR	19	N-Term(iTRAQ4plex	0.841	5.4	79	2	928.49
									GPVLAWINAVSAFR	9	N-Term(iTRAQ4plex	0.635	5.4	72	2	822.97
									AIHLDLEEYR	40	N-Term(iTRAQ4plex	0.98	4.72	65	3	468.25
									TVFGTEPDMIR	22	N-Term(iTRAQ4plex	0.814	4.63	58	2	705.37
									GNSYFMVEVK	2	N-Term(iTRAQ4plex); M6(Oxidation); K10(iTRAQ4plex)	0.917	4.36	50	2	739.38
									MMAVAADTLQR	28	N-Term(iTRAQ4plex	0.878	4.35	57	2	675.85
									EEILMHLWR	13	N-Term(iTRAQ4plex	0.773	4.13	45	2	685.87
									MMAVAADTLQR	9	N-Term(iTRAQ4plex); M2(Oxidation	0.714	3.94	56	2	683.85
									MFQEIVHK	13	N-Term(iTRAQ4plex); K8(iTRAQ4plex	0.861	3.93	59	2	660.37
									TVFGTEPDMIR	5	N-Term(iTRAQ4plex); M9(Oxidation	0.845	3.72	45	2	713.36
									DGSTIPIAK	2	N-Term(iTRAQ4plex); K9(iTRAQ4plex	0.745	3.63	54	2	595.36
									WNYIEGTK	11	N-Term(iTRAQ4plex); K8(iTRAQ4plex	0.933	3.61	49	2	649.85
									EEILMHLWR	4	N-Term(iTRAQ4plex); M5(Oxidation	0.832	3.51	43	3	462.92
									HLEDVFSK	17	N-Term(iTRAQ4plex); K8(iTRAQ4plex	0.799	3.49	60	2	631.85
									YPSLSIHGIEGAFDEPGTK	4	N-Term(iTRAQ4plex); K19(iTRAQ4plex	0.732		44		
									FLFDTK	7	N-Term(iTRAQ4plex); K6(iTRAQ4plex	0.864		43		
4503107	CST3	cystatin-C precursor	42.47	6	6	333	1.176	15.8								
									TQPNLDNCPFHDQPHLK	92	N-Term(iTRAQ4plex); C8(Methylthio); K17(iTRAQ4plex)	1.117	8.52	87	4	585.29
									LVGGPMDASVEEEGVRR	18	N-Term(iTRAQ4plex	1.289	6.88	68	2	973.00
									LVGGPMDASVEEEGVR	88	N-Term(iTRAQ4plex	1.189	6.5	100	2	894.95
									LVGGPMDASVEEEGVRR	4	N-Term(iTRAQ4plex); M6(Oxidation	1.388	5.75	46	3	654.34
									LVGGPMDASVEEEGVR	44	N-Term(iTRAQ4plex); M6(Oxidation	1.169	5.71	86	2	902.95
									QIVAGVNYFLDVELGR	5	N-Term(iTRAQ4plex	0.971	5.64	108	2	969.03
									ALDFAVGEYNK	80	N-Term(iTRAQ4plex); K11(iTRAQ4plex	1.196	5.12	71	2	757.91
									KQIVAGVNYFLDVELGR	2	N-Term(iTRAQ4plex); K1(iTRAQ4plex	0.512		55		
4503635	F2	prothrombin preproprotein	42.28	20	20	122	1.498	70								
									ITDNMFCAGYKPDEGK	6	N-Term(iTRAQ4plex); C7(Methylthio); K11(iTRAQ4plex); K16(iTRAQ4plex)	1.718	6.56	69	3	756.37
									TATSEYQTFFNPR	16	N-Term(iTRAQ4plex	1.794	6.4	80	2	853.42
									RQECSIPVCGQDQVTVAMTPR	3	N-Term(iTRAQ4plex); C4(Methylthio); C9(Methylthio)	1.418	6.34	98	3	852.07

									ETAASLLQAGYK	14	N-Term(iTRAQ4plex); K12(iTRAQ4plex	1.502	6.14	104	2	770.43
									SPQELLCGASLISDR	3	N-Term(iTRAQ4plex); C7(Methylthio	1.274	5.04	63	2	889.95
									GDACEGDSGGPFVMK	6	N-Term(iTRAQ4plex); C4(Methylthio); K15(iTRAQ4plex)	1.49	5.03	83	2	902.40
									ELLESYIDGR	18	N-Term(iTRAQ4plex	1.179	4.57	56	2	669.85
									IVEGSDAEIGMSPWQVMLFR	4	N-Term(iTRAQ4plex	1.168	4.4	80	2	1205.11
									YGFYTHVFR	8	N-Term(iTRAQ4plex	1.558	4.01	43	2	667.34
									SGIECQLWR	5	N-Term(iTRAQ4plex); C5(Methylthio	1.498	3.98	52	2	641.31
									ETWTANVGK	3	N-Term(iTRAQ4plex); K9(iTRAQ4plex	1.496	3.9	78	2	647.36
									VTGWGNLK	21	N-Term(iTRAQ4plex); K8(iTRAQ4plex	2.12	3.68	54	2	581.84
									LAVTTHGLPCLAWASAQAK	2	N-Term(iTRAQ4plex); C10(Methylthio); K19(iTRAQ4plex)	1.403	3.39	67	3	758.08
									TFGSGEADCGLRPLFEK	2	N-Term(iTRAQ4plex); C9(Methylthio); K17(iTRAQ4plex)	1.658	3.36	44	3	721.03
									KSPQELLCGASLISDR	2	N-Term(iTRAQ4plex); K1(iTRAQ4plex); C8(Methylthio)	0.868		79		
									GQPSVLQVVNLPIVERPVCK	1	N-Term(iTRAQ4plex); C19(Methylthio); K20(iTRAQ4plex)	1.426		56		
									VIDQFGE	2	N-Term(iTRAQ4plex	1.301		42		
									ISMLEK	3	N-Term(iTRAQ4plex); K6(iTRAQ4plex	1.274		40		
									KPVAFSDYIHPVCLPDR	2	N-Term(iTRAQ4plex); K1(iTRAQ4plex); C13(Methylthio)	1.698		39		
									SEGSSVNLSPPLEQCVPDR	1	N-Term(iTRAQ4plex); C15(Methylthio	1.416		38		
91598939	SPP1	osteopontin isoform c precursor	42.16	1	9	136	1.739	32.3								
									DSYETSQLDDQSAETHSHK	15	N-Term(iTRAQ4plex); K19(iTRAQ4plex		9.51	110	3	822.71
									GKDSYETSQLDDQSAETHSHK	3	N-Term(iTRAQ4plex); K2(iTRAQ4plex); K21(iTRAQ4plex)		7.7	43	5	559.87
									ANDESNEHSDVIDSQELSK	25	N-Term(iTRAQ4plex); K19(iTRAQ4plex		7.4	98	3	802.38
									KANDESNEHSDVIDSQELSK	19	N-Term(iTRAQ4plex); K1(iTRAQ4plex); K20(iTRAQ4plex)		7.25	94	3	893.11
									AIPVAQDLNAPSDWDSR	32	N-Term(iTRAQ4plex		5.77	88	2	1000.00
									ISHELDSASSEVN	37	N-Term(iTRAQ4plex		4.77	55	2	766.37
									GDSVVYGLR	2	N-Term(iTRAQ4plex		3.86	39	2	555.30
									QNAVSSEETNDFK	2	N-Term(iTRAQ4plex); K13(iTRAQ4plex	1.739	3.7	67	2	878.93
156627579	CLEC3B	tetranectin precursor	42.08	7	7	82	0.954	22.5								
									LDTLAQEVALLK	23	N-Term(iTRAQ4plex); K12(iTRAQ4plex	0.934	5.78	95	2	801.49
									TFHEASEDCISR	13	N-Term(iTRAQ4plex); C9(Methylthio	0.946	5.61	68	3	528.90
									NWETEITAQPDGGK	13	N-Term(iTRAQ4plex); K14(iTRAQ4plex	0.965	5.33	75	2	917.46
									EQQALQTVCLK	16	N-Term(iTRAQ4plex); C9(Methylthio); K11(iTRAQ4plex)	1.065	5.33	81	2	797.93
									CFLAFTQTK	11	N-Term(iTRAQ4plex); C1(Methylthio); K9(iTRAQ4plex)	0.881	4.19	51	2	696.87
									GGTLGTPQTGSENDALYEYLR	4	N-Term(iTRAQ4plex	0.967	3.98	77	2	1193.59
									MFEELK	2	N-Term(iTRAQ4plex); K6(iTRAQ4plex	1.057		38		
32483410	GC	vitamin D-binding protein precursor	41.98	19	19	315	1.045	52.9								
									SCESNSPFPVHPGTAECCTK	3	N-Term(iTRAQ4plex); C2(Methylthio); C17(Methylthio); C18(Methylthio); K20(iTRAQ4plex)	1.093	6.74	67	3	840.69
									FPSGTFEQVSQLVK	15	N-Term(iTRAQ4plex); K14(iTRAQ4plex	1.029	6.47	117	2	928.02
									KFPSGTFEQVSQLVK	10	N-Term(iTRAQ4plex); K1(iTRAQ4plex); K15(iTRAQ4plex)	1.068	6.11	58	2	1064.11
									GQELCADYSENTFTEYK	11	N-Term(iTRAQ4plex); C5(Methylthio); K17(iTRAQ4plex)	1	5.91	86	2	1166.52
									VCSQYAAYGEK	48	N-Term(iTRAQ4plex); C2(Methylthio); K11(iTRAQ4plex)	1.063	5.6	75	2	776.87
									HQPQEFPTYVEPTNDEICEAFR	19	N-Term(iTRAQ4plex); C18(Methylthio	0.961	5.42	59	3	947.43
									KLCMAALK	24	N-Term(iTRAQ4plex); K1(iTRAQ4plex); C3(Methylthio); K8(iTRAQ4plex)	1.132	5.11	43	3	452.60
									EDFTSLSLVLYSR	14	N-Term(iTRAQ4plex	0.962	4.62	84	2	837.45
									HLSLLTTLSNR	11	N-Term(iTRAQ4plex	0.785	4.16	59	2	699.91
									ELSSFIDK	36	N-Term(iTRAQ4plex); K8(iTRAQ4plex	1.229	4.09	65	2	613.85
									RTHLPEVFLSK	2	N-Term(iTRAQ4plex); K11(iTRAQ4plex	1.384	4.08	37	3	538.99
									THLPEVFLSK	33	N-Term(iTRAQ4plex); K10(iTRAQ4plex	1.042	4.02	63	2	729.93
									LPDATPTELAK	13	N-Term(iTRAQ4plex); K11(iTRAQ4plex	0.731	3.99	63	2	722.42
									VPTADLEDVLPLAEDITNILSK	5	N-Term(iTRAQ4plex); K22(iTRAQ4plex	0.785	3.92	56	3	885.50
									LCMAALK	44	N-Term(iTRAQ4plex); C2(Methylthio); K7(iTRAQ4plex)	1.04	3.54	55	2	542.30
									LCDNLSTK	5	N-Term(iTRAQ4plex); C2(Methylthio); K8(iTRAQ4plex)	1.012	3.41	53	2	614.32
									FEDCCQEK	8	N-Term(iTRAQ4plex); C4(Methylthio); C5(Methylthio); K8(iTRAQ4plex)	1.096	3.2	39	2	691.27
									VLEPTLK	10	N-Term(iTRAQ4plex); K7(iTRAQ4plex	1.101	3.14	46	2	544.35
									EFSHLGK	4	N-Term(iTRAQ4plex); K7(iTRAQ4plex	1.087		44		
144226251	CHI3L1	chitinase-3-like protein 1 precursor	41.78	12	12	125	1.582	42.6								
									SFTLASSETGVGAPISGPGIPGR	8	N-Term(iTRAQ4plex	1.524	7.19	95	2	1151.61
									EGDGSCFPDALDR	17	N-Term(iTRAQ4plex); C6(Methylthio	1.611	5.72	69	2	786.33
									GNQWVGYDDQESVK	15	N-Term(iTRAQ4plex); K14(iTRAQ4plex	1.748	5.53	73	2	956.97
									FSNTDYAVGYMLR	4	N-Term(iTRAQ4plex	1.503	5.48	74	2	840.91
									EAGTLAYYEICDFLR	3	N-Term(iTRAQ4plex); C11(Methylthio	1.433	5.3	80	2	977.46
									THGFDGLDLAWLYPGR	4	N-Term(iTRAQ4plex	1.871	5.23	88	2	981.50
									VTIDSSYDIAK	21	N-Term(iTRAQ4plex); K11(iTRAQ4plex	1.778	5.16	89	2	750.41
									QLLLSAALSAGK	8	N-Term(iTRAQ4plex); K12(iTRAQ4plex	1.612	5.08	85	2	730.46
									ILGQQVPYATK	10	N-Term(iTRAQ4plex); K11(iTRAQ4plex	1.387	4.83	76	2	753.45
									TLLSVGGWNFGSQR	10	N-Term(iTRAQ4plex	1.402	4.78	67	2	833.44
									FPLTNAIK	13	N-Term(iTRAQ4plex); K8(iTRAQ4plex	1.426	3.32	66	2	596.37
									LVMGIPTFGR	12	N-Term(iTRAQ4plex	1.651	3.29	54	2	617.86
116812622	PEBP4	phosphatidylethanolamine-binding protein 4 precursor	41.41	7	7	46	1.16	25.7								
									IQGQELSAYQAPSPPAHSGFHR	7	N-Term(iTRAQ4plex	0.975	5.72	83	4	631.32
									ITSWMEPIVK	15	N-Term(iTRAQ4plex); K10(iTRAQ4plex	1.291	4.66	75	2	746.43
									FHLGEPEASTQFMTQNYQDSPTLQAPR	2	N-Term(iTRAQ4plex	1.05	3.64	46	3	1079.85
									HWLVTDIK	4	N-Term(iTRAQ4plex); K8(iTRAQ4plex	1.357	3.4	41	3	433.93
									VVPDCNNYR	2	N-Term(iTRAQ4plex); C5(Methylthio	1.265	3.33	34	2	635.30
									VISLLPK	14	N-Term(iTRAQ4plex); K7(iTRAQ4plex	1.052	3.2	38	2	529.36
									YQFFVYLQEGK	1	N-Term(iTRAQ4plex); K11(iTRAQ4plex	1.269		42		

									ITSWMEPIVK	1	N-Term(iTRAQ4plex); M5(Oxidation); K10(iTRAQ4plex)	1.365		39		
310125235	LOC100293534	PREDICTED: complement C4-B-like isoform 1	40.37	0	53	997		192.7								
									MRPSTDTITVMVENSHGLF	35	N-Term(iTRAQ4plex		8.46	106	4	572.80
									MRPSTDTITVMVENSHGLF	13	N-Term(iTRAQ4plex); M1(Oxidation		8.31	94	4	576.80
									YVSHFETEGPHVLLYFDSVPTSF	15	N-Term(iTRAQ4plex		7.3	71	3	942.14
									STQDTVIALDALSAYWIASHTTEEF	13	N-Term(iTRAQ4plex		7.07	112	3	974.83
									GPEVQLVAHSPWLK	39	N-Term(iTRAQ4plex); K14(iTRAQ4plex		7.02	78	3	617.02
									VTASDPLDTLGSEGALSPGGVASLLF	19	N-Term(iTRAQ4plex		7.01	119	2	1314.21
									KADGSYAAWLSR	27	N-Term(iTRAQ4plex); K1(iTRAQ4plex		6.77	75	3	538.29
									VLSLAQEQVGGSPEK	16	N-Term(iTRAQ4plex); K15(iTRAQ4plex		6.7	102	2	915.51
									LLLFSPSVVHLGVPLSVGVQLQDVPF	5	N-Term(iTRAQ4plex		6.68	79	3	971.91
									RGHLFLQTDQPIYNPGQR	7	N-Term(iTRAQ4plex		6.63	68	3	762.08
									GLEEELQFSLGSK	12	N-Term(iTRAQ4plex); K13(iTRAQ4plex		6.56	93	2	862.97
									GHLFLQTDQPIYNPGQR	58	N-Term(iTRAQ4plex		6.5	82	3	710.04
									AVGSGATFSHYYYMILSF	11	N-Term(iTRAQ4plex		6.17	71	3	723.03
									TTNIQGINLLFSSR	38	N-Term(iTRAQ4plex		5.93	89	2	854.48
									DFALLSLQVPLK	14	N-Term(iTRAQ4plex); K12(iTRAQ4plex		5.62	68	2	816.50
									EMSGSPASGIPVK	41	N-Term(iTRAQ4plex); K13(iTRAQ4plex		5.44	88	2	774.42
									VGLSGMAIADVTLLSGFHALF	4	N-Term(iTRAQ4plex		5.38	71	3	758.09
									GSFEFPVGDAVSK	26	N-Term(iTRAQ4plex); K13(iTRAQ4plex		5.37	88	2	814.43
									DDPDAPLQPVTPLQLFEGR	7	N-Term(iTRAQ4plex		5.3	73	2	1126.59
									ADGSYAAWLSR	16	N-Term(iTRAQ4plex		5.3	72	2	670.84
									EELVYELNPLDHR	66	N-Term(iTRAQ4plex		5.25	76	2	885.96
									KYVLPNFEVK	13	N-Term(iTRAQ4plex); K1(iTRAQ4plex); K10(iTRAQ4plex)		5.24	58	3	557.01
									AEMADQAAAWLTF	8	N-Term(iTRAQ4plex); M3(Oxidation		5.14	80	2	797.39
									AEMADQAAAWLTF	2	N-Term(iTRAQ4plex		5.12	80	2	789.40
									ALEILQEEDLIDEDDIPVR	25	N-Term(iTRAQ4plex		4.92	93	2	1185.12
									LNMGITDLQGLR	6	N-Term(iTRAQ4plex		4.87	61	2	737.91
									TEQWSTLPPETK	31	N-Term(iTRAQ4plex); K12(iTRAQ4plex		4.86	82	2	852.96
									LQETSNWLLSQQQADGSFQDLSPVIHF	8	N-Term(iTRAQ4plex		4.79	93	3	1081.22
									AEFQDALEK	38	N-Term(iTRAQ4plex); K9(iTRAQ4plex		4.78	68	2	669.86
									GLCVATPVQLF	25	N-Term(iTRAQ4plex); C3(Methylthio		4.74	65	2	673.87
									YIYGKPVQGVAYVF	3	N-Term(iTRAQ4plex); K5(iTRAQ4plex		4.71	58	2	951.05
									FGLLDEDGKK	24	N-Term(iTRAQ4plex); K9(iTRAQ4plex); K10(iTRAQ4plex)		4.6	70	2	777.45
									VDVQAGACEGK	16	N-Term(iTRAQ4plex); C8(Methylthio); K11(iTRAQ4plex)		4.6	73	2	705.85
									VGDTLNLNLR	33	N-Term(iTRAQ4plex		4.59	57	2	629.86
									EPFLSCCQFAESLR	2	N-Term(iTRAQ4plex); C6(Methylthio); C7(Methylthio)		4.48	69	2	933.42
									SFFPENWLWR	15	N-Term(iTRAQ4plex		4.34	58	2	763.39
									EMSGSPASGIPVK	2	N-Term(iTRAQ4plex); M2(Oxidation); K13(iTRAQ4plex)		4.27	57	2	782.42
									LELSVDGAK	29	N-Term(iTRAQ4plex); K9(iTRAQ4plex		4.23	68	2	610.35
									ITQVLHFTK	18	N-Term(iTRAQ4plex); K9(iTRAQ4plex		4.23	77	2	687.92
									VDFTLSSER	32	N-Term(iTRAQ4plex		4.17	56	2	599.31
									LNMGITDLQGLR	4	N-Term(iTRAQ4plex); M3(Oxidation		4.16	66	2	745.91
									VEYGFQVK	30	N-Term(iTRAQ4plex); K8(iTRAQ4plex		4.16	51	2	629.36
									CSVFYGAPSK	29	N-Term(iTRAQ4plex); C1(Methylthio); K10(iTRAQ4plex)		4.12	70	2	696.85
									TYNVLDMK	21	N-Term(iTRAQ4plex); K8(iTRAQ4plex		4.09	46	2	636.35
									SCGLHQLLR	17	N-Term(iTRAQ4plex); C2(Methylthio		3.95	53	2	608.82
									FGLLDEDGK	12	N-Term(iTRAQ4plex); K9(iTRAQ4plex		3.87	48	2	641.35
									LGQYASPTAK	3	N-Term(iTRAQ4plex); K10(iTRAQ4plex		3.86	79	2	662.38
									HLVPGAPFLLQALVR	9	N-Term(iTRAQ4plex		3.78	58	3	592.37
									YVLPNFEVK	20	N-Term(iTRAQ4plex); K9(iTRAQ4plex		3.74	65	2	698.91
									TLEIPGNSDPNMIPDGDFNSYVF	4	N-Term(iTRAQ4plex		3.53	40	3	899.10
									VHYTVCIWR	4	N-Term(iTRAQ4plex); C6(Methylthio		3.48	36	3	456.24
									VFALDQK	17	N-Term(iTRAQ4plex); K7(iTRAQ4plex		3.47	42	2	554.83
									ASSFLGEK	2	N-Term(iTRAQ4plex); K8(iTRAQ4plex		3.46	44	2	563.83
									GSSTWLTAFVLK	1	N-Term(iTRAQ4plex); K12(iTRAQ4plex			51		
									AVGSGATFSHYYYMILSF	1	N-Term(iTRAQ4plex); M14(Oxidation			42		
									VLQIEK	1	N-Term(iTRAQ4plex); K6(iTRAQ4plex			42		
									ECVGFEAVQEVPVGLVQPASATLYDYYI PER	1	N-Term(iTRAQ4plex); C2(Methylthio			31		
178557739	C4B	complement C4-B preproproteir	40.37	0	53	997		192.6								
									MRPSTDTITVMVENSHGLF	35	N-Term(iTRAQ4plex		8.46	106	4	572.80
									MRPSTDTITVMVENSHGLF	13	N-Term(iTRAQ4plex); M1(Oxidation		8.31	94	4	576.80
									YVSHFETEGPHVLLYFDSVPTSF	15	N-Term(iTRAQ4plex		7.3	71	3	942.14
									STQDTVIALDALSAYWIASHTTEEF	13	N-Term(iTRAQ4plex		7.07	112	3	974.83
									GPEVQLVAHSPWLK	39	N-Term(iTRAQ4plex); K14(iTRAQ4plex		7.02	78	3	617.02
									VTASDPLDTLGSEGALSPGGVASLLF	19	N-Term(iTRAQ4plex		7.01	119	2	1314.21
									KADGSYAAWLSR	27	N-Term(iTRAQ4plex); K1(iTRAQ4plex		6.77	75	3	538.29
									VLSLAQEQVGGSPEK	16	N-Term(iTRAQ4plex); K15(iTRAQ4plex		6.7	102	2	915.51
									LLLFSPSVVHLGVPLSVGVQLQDVPF	5	N-Term(iTRAQ4plex		6.68	79	3	971.91
									RGHLFLQTDQPIYNPGQR	7	N-Term(iTRAQ4plex		6.63	68	3	762.08
									GLEEELQFSLGSK	12	N-Term(iTRAQ4plex); K13(iTRAQ4plex		6.56	93	2	862.97
									GHLFLQTDQPIYNPGQR	58	N-Term(iTRAQ4plex		6.5	82	3	710.04
									AVGSGATFSHYYYMILSF	11	N-Term(iTRAQ4plex		6.17	71	3	723.03
									TTNIQGINLLFSSR	38	N-Term(iTRAQ4plex		5.93	89	2	854.48
									DFALLSLQVPLK	14	N-Term(iTRAQ4plex); K12(iTRAQ4plex		5.62	68	2	816.50
									EMSGSPASGIPVK	41	N-Term(iTRAQ4plex); K13(iTRAQ4plex		5.44	88	2	774.42
									VGLSGMAIADVTLLSGFHALF	4	N-Term(iTRAQ4plex		5.38	71	3	758.09
									GSFEFPVGDAVSK	26	N-Term(iTRAQ4plex); K13(iTRAQ4plex		5.37	88	2	814.43
									DDPDAPLQPVTPLQLFEGR	7	N-Term(iTRAQ4plex		5.3	73	2	1126.59
									ADGSYAAWLSR	16	N-Term(iTRAQ4plex		5.3	72	2	670.84
									EELVYELNPLDHR	66	N-Term(iTRAQ4plex		5.25	76	2	885.96
									KYVLPNFEVK	13	N-Term(iTRAQ4plex); K1(iTRAQ4plex); K10(iTRAQ4plex)		5.24	58	3	557.01
									AEMADQAAAWLTF	8	N-Term(iTRAQ4plex); M3(Oxidation		5.14	80	2	797.39
									AEMADQAAAWLTF	2	N-Term(iTRAQ4plex		5.12	80	2	789.40
									ALEILQEEDLIDEDDIPVR	25	N-Term(iTRAQ4plex		4.92	93	2	1185.12
									LNMGITDLQGLR	6	N-Term(iTRAQ4plex		4.87	61	2	737.91
									TEQWSTLPPETK	31	N-Term(iTRAQ4plex); K12(iTRAQ4plex		4.86	82	2	852.96
									LQETSNWLLSQQQADGSFQDLSPVIHF	8	N-Term(iTRAQ4plex		4.79	93	3	1081.22
									AEFQDALEK	38	N-Term(iTRAQ4plex); K9(iTRAQ4plex		4.78	68	2	669.86

									GLCVATPVQLR	25	N-Term(iTRAQ4plex); C3(Methylthio		4.74	65	2	673.87
									YIYGKPVQGVAYVR	3	N-Term(iTRAQ4plex); K5(iTRAQ4plex		4.71	58	2	951.05
									FGLLDEDGKK	24	N-Term(iTRAQ4plex); K9(iTRAQ4plex); K10(iTRAQ4plex)		4.6	70	2	777.45
									VDVQAGACEGK	16	N-Term(iTRAQ4plex); C8(Methylthio); K11(iTRAQ4plex)		4.6	73	2	705.85
									VGDTLNLNLR	33	N-Term(iTRAQ4plex		4.59	57	2	629.86
									EPFLSCCQFAESLR	2	N-Term(iTRAQ4plex); C6(Methylthio); C7(Methylthio)		4.48	69	2	933.42
									SFFPENWLWR	15	N-Term(iTRAQ4plex		4.34	58	2	763.39
									EMSGSPASGIPVK	2	N-Term(iTRAQ4plex); M2(Oxidation); K13(iTRAQ4plex)		4.27	57	2	782.42
									LELSVDGAK	29	N-Term(iTRAQ4plex); K9(iTRAQ4plex		4.23	68	2	610.35
									ITQVLHFTK	18	N-Term(iTRAQ4plex); K9(iTRAQ4plex		4.23	77	2	687.92
									VDFTLSSER	32	N-Term(iTRAQ4plex		4.17	56	2	599.31
									LNMGITDLQGLR	4	N-Term(iTRAQ4plex); M3(Oxidation		4.16	66	2	745.91
									VEYGFQVK	30	N-Term(iTRAQ4plex); K8(iTRAQ4plex		4.16	51	2	629.36
									CSVFYGAPSK	29	N-Term(iTRAQ4plex); C1(Methylthio); K10(iTRAQ4plex)		4.12	70	2	696.85
									TYNVLDMK	21	N-Term(iTRAQ4plex); K8(iTRAQ4plex		4.09	46	2	636.35
									SCGLHQLLR	17	N-Term(iTRAQ4plex); C2(Methylthio		3.95	53	2	608.82
									FGLLDEDGK	12	N-Term(iTRAQ4plex); K9(iTRAQ4plex		3.87	48	2	641.35
									LGQYASPTAK	3	N-Term(iTRAQ4plex); K10(iTRAQ4plex		3.86	79	2	662.38
									HLVPGAPFLLQALVR	9	N-Term(iTRAQ4plex		3.78	58	3	592.37
									YVLPNFEVK	20	N-Term(iTRAQ4plex); K9(iTRAQ4plex		3.74	65	2	698.91
									TLEIPGNSDPNMIPDGDFNSYVR	4	N-Term(iTRAQ4plex		3.53	40	3	899.10
									VHYTVCIWR	4	N-Term(iTRAQ4plex); C6(Methylthio		3.48	36	3	456.24
									VFALDQK	17	N-Term(iTRAQ4plex); K7(iTRAQ4plex		3.47	42	2	554.83
									ASSFLGEK	2	N-Term(iTRAQ4plex); K8(iTRAQ4plex		3.46	44	2	563.83
									GSSTWLTAFVLK	1	N-Term(iTRAQ4plex); K12(iTRAQ4plex			51		
									AVGSGATFSHYYYMILSR	1	N-Term(iTRAQ4plex); M14(Oxidation			42		
									VLQIEK	1	N-Term(iTRAQ4plex); K6(iTRAQ4plex			42		
									ECVGFEAVQEVPVGLVQPASATLYDYYI PER	1	N-Term(iTRAQ4plex); C2(Methylthio			31		
4506335	PVALB	parvalbumin alpha	40	5	5	18	1.117	12.1								
									AVGAFSATDSFDHK	2	N-Term(iTRAQ4plex); K14(iTRAQ4plex	1.158	5.75	96	2	870.94
									VFHMLDKDK	6	N-Term(iTRAQ4plex); K7(iTRAQ4plex); K9(iTRAQ4plex)	1.076	4.02	74	3	522.30
									FFQMVGLK	7	N-Term(iTRAQ4plex); K8(iTRAQ4plex	1.124	3.69	52	2	629.37
									VFHMLDK	2	N-Term(iTRAQ4plex); K7(iTRAQ4plex	1.119	3.35	49	3	393.22
									IGVDEFSTLVAES	1	N-Term(iTRAQ4plex	1.376		54		
310125237	LOC100293534	PREDICTED: complement C4-B-like isoform 2	39.69	0	51	989		187.6								
									MRPSTDTITVMVENSHGLR	35	N-Term(iTRAQ4plex		8.46	106	4	572.80
									MRPSTDTITVMVENSHGLR	13	N-Term(iTRAQ4plex); M1(Oxidation		8.31	94	4	576.80
									YVSHFETEGPHVLLYFDSVPTSR	15	N-Term(iTRAQ4plex		7.3	71	3	942.14
									STQDTVIALDALSAYWIASHTTEER	13	N-Term(iTRAQ4plex		7.07	112	3	974.83
									GPEVQLVAHSPWLK	39	N-Term(iTRAQ4plex); K14(iTRAQ4plex		7.02	78	3	617.02
									VTASDPLDTLGSEGALSPGGVASLLR	19	N-Term(iTRAQ4plex		7.01	119	2	1314.21
									KADGSYAAWLSR	27	N-Term(iTRAQ4plex); K1(iTRAQ4plex		6.77	75	3	538.29
									VLSLAQEQVGGSPEK	16	N-Term(iTRAQ4plex); K15(iTRAQ4plex		6.7	102	2	915.51
									LLLFSPSVVHLGVPLSVGVQLQDVPR	5	N-Term(iTRAQ4plex		6.68	79	3	971.91
									RGHLFLQTDQPIYNPGQR	7	N-Term(iTRAQ4plex		6.63	68	3	762.08
									GLEEELQFSLGSK	12	N-Term(iTRAQ4plex); K13(iTRAQ4plex		6.56	93	2	862.97
									GHLFLQTDQPIYNPGQR	58	N-Term(iTRAQ4plex		6.5	82	3	710.04
									AVGSGATFSHYYYMILSR	11	N-Term(iTRAQ4plex		6.17	71	3	723.03
									TTNIQGINLLFSSR	38	N-Term(iTRAQ4plex		5.93	89	2	854.48
									DFALLSLQVPLK	14	N-Term(iTRAQ4plex); K12(iTRAQ4plex		5.62	68	2	816.50
									EMSGSPASGIPVK	41	N-Term(iTRAQ4plex); K13(iTRAQ4plex		5.44	88	2	774.42
									GSFEFPVGDAVSK	26	N-Term(iTRAQ4plex); K13(iTRAQ4plex		5.37	88	2	814.43
									DDPDAPLQPVTPLQLFEGR	7	N-Term(iTRAQ4plex		5.3	73	2	1126.59
									ADGSYAAWLSR	16	N-Term(iTRAQ4plex		5.3	72	2	670.84
									EELVYELNPLDHR	66	N-Term(iTRAQ4plex		5.25	76	2	885.96
									KYVLPNFEVK	13	N-Term(iTRAQ4plex); K1(iTRAQ4plex); K10(iTRAQ4plex)		5.24	58	3	557.01
									AEMADQAAAWLTR	8	N-Term(iTRAQ4plex); M3(Oxidation		5.14	80	2	797.39
									AEMADQAAAWLTR	2	N-Term(iTRAQ4plex		5.12	80	2	789.40
									ALEILQEEDLIDEDDIPVR	25	N-Term(iTRAQ4plex		4.92	93	2	1185.12
									LNMGITDLQGLR	6	N-Term(iTRAQ4plex		4.87	61	2	737.91
									TEQWSTLPPETK	31	N-Term(iTRAQ4plex); K12(iTRAQ4plex		4.86	82	2	852.96
									LQETSNWLLSQQQADGSFQDLSPVIHR	8	N-Term(iTRAQ4plex		4.79	93	3	1081.22
									AEFQDALEK	38	N-Term(iTRAQ4plex); K9(iTRAQ4plex		4.78	68	2	669.86
									GLCVATPVQLR	25	N-Term(iTRAQ4plex); C3(Methylthio		4.74	65	2	673.87
									YIYGKPVQGVAYVR	3	N-Term(iTRAQ4plex); K5(iTRAQ4plex		4.71	58	2	951.05
									FGLLDEDGKK	24	N-Term(iTRAQ4plex); K9(iTRAQ4plex); K10(iTRAQ4plex)		4.6	70	2	777.45
									VDVQAGACEGK	16	N-Term(iTRAQ4plex); C8(Methylthio); K11(iTRAQ4plex)		4.6	73	2	705.85
									VGDTLNLNLR	33	N-Term(iTRAQ4plex		4.59	57	2	629.86
									EPFLSCCQFAESLR	2	N-Term(iTRAQ4plex); C6(Methylthio); C7(Methylthio)		4.48	69	2	933.42
									SFFPENWLWR	15	N-Term(iTRAQ4plex		4.34	58	2	763.39
									EMSGSPASGIPVK	2	N-Term(iTRAQ4plex); M2(Oxidation); K13(iTRAQ4plex)		4.27	57	2	782.42
									LELSVDGAK	29	N-Term(iTRAQ4plex); K9(iTRAQ4plex		4.23	68	2	610.35
									ITQVLHFTK	18	N-Term(iTRAQ4plex); K9(iTRAQ4plex		4.23	77	2	687.92
									VDFTLSSER	32	N-Term(iTRAQ4plex		4.17	56	2	599.31
									LNMGITDLQGLR	4	N-Term(iTRAQ4plex); M3(Oxidation		4.16	66	2	745.91
									VEYGFQVK	30	N-Term(iTRAQ4plex); K8(iTRAQ4plex		4.16	51	2	629.36
									CSVFYGAPSK	29	N-Term(iTRAQ4plex); C1(Methylthio); K10(iTRAQ4plex)		4.12	70	2	696.85
									TYNVLDMK	21	N-Term(iTRAQ4plex); K8(iTRAQ4plex		4.09	46	2	636.35
									SCGLHQLLR	17	N-Term(iTRAQ4plex); C2(Methylthio		3.95	53	2	608.82
									FGLLDEDGK	12	N-Term(iTRAQ4plex); K9(iTRAQ4plex		3.87	48	2	641.35
									LGQYASPTAK	3	N-Term(iTRAQ4plex); K10(iTRAQ4plex		3.86	79	2	662.38
									HLVPGAPFLLQALVR	9	N-Term(iTRAQ4plex		3.78	58	3	592.37
									YVLPNFEVK	20	N-Term(iTRAQ4plex); K9(iTRAQ4plex		3.74	65	2	698.91
									TLEIPGNSDPNMIPDGDFNSYVR	4	N-Term(iTRAQ4plex		3.53	40	3	899.10
									VFALDQK	17	N-Term(iTRAQ4plex); K7(iTRAQ4plex		3.47	42	2	554.83

									ASSFLGEK	2	N-Term(iTRAQ4plex); K8(iTRAQ4plex		3.46	44	2	563.83
									GSSTWLTAFVLK	1	N-Term(iTRAQ4plex); K12(iTRAQ4plex			51		
									AVGSGATFSHYYYMILSF	1	N-Term(iTRAQ4plex); M14(Oxidation			42		
									VLQIEK	1	N-Term(iTRAQ4plex); K6(iTRAQ4plex			42		
									ECVGFEAVQEVPVGLVQPASATLYDYYI PER	1	N-Term(iTRAQ4plex); C2(Methylthio			31		
67190748	C4A	complement C4-A preproproteir	39.68	2	52	984	0.302	192.7								
									MRPSTDTITVMVENSHGLF	35	N-Term(iTRAQ4plex		8.46	106	4	572.80
									MRPSTDTITVMVENSHGLF	13	N-Term(iTRAQ4plex); M1(Oxidation		8.31	94	4	576.80
									YVSHFETEGPHVLLYFDSVPTSF	15	N-Term(iTRAQ4plex		7.3	71	3	942.14
									STQDTVIALDALSAYWIASHTTEEF	13	N-Term(iTRAQ4plex		7.07	112	3	974.83
									GPEVQLVAHSPWLK	39	N-Term(iTRAQ4plex); K14(iTRAQ4plex		7.02	78	3	617.02
									VTASDPLDTLGSEGALSPGGVASLLF	19	N-Term(iTRAQ4plex		7.01	119	2	1314.21
									KADGSYAAWLSR	27	N-Term(iTRAQ4plex); K1(iTRAQ4plex		6.77	75	3	538.29
									VLSLAQEQVGGSPEK	16	N-Term(iTRAQ4plex); K15(iTRAQ4plex		6.7	102	2	915.51
									LLLFSPSVVHLGVPLSVGVQLQDVPF	5	N-Term(iTRAQ4plex		6.68	79	3	971.91
									RGHLFLQTDQPIYNPGQR	7	N-Term(iTRAQ4plex		6.63	68	3	762.08
									GLEEELQFSLGSK	12	N-Term(iTRAQ4plex); K13(iTRAQ4plex		6.56	93	2	862.97
									GHLFLQTDQPIYNPGQR	58	N-Term(iTRAQ4plex		6.5	82	3	710.04
									AVGSGATFSHYYYMILSF	11	N-Term(iTRAQ4plex		6.17	71	3	723.03
									TTNIQGINLLFSSR	38	N-Term(iTRAQ4plex		5.93	89	2	854.48
									DFALLSLQVPLK	14	N-Term(iTRAQ4plex); K12(iTRAQ4plex		5.62	68	2	816.50
									EMSGSPASGIPVK	41	N-Term(iTRAQ4plex); K13(iTRAQ4plex		5.44	88	2	774.42
									VGLSGMAIADVTLLSGFHALF	4	N-Term(iTRAQ4plex		5.38	71	3	758.09
									GSFEFPVGDAVSK	26	N-Term(iTRAQ4plex); K13(iTRAQ4plex		5.37	88	2	814.43
									AEMADQASAWLTR	4	N-Term(iTRAQ4plex	0.302	5.35	75	2	797.40
									DDPDAPLQPVTPLQLFEGR	7	N-Term(iTRAQ4plex		5.3	73	2	1126.59
									ADGSYAAWLSR	16	N-Term(iTRAQ4plex		5.3	72	2	670.84
									EELVYELNPLDHR	66	N-Term(iTRAQ4plex		5.25	76	2	885.96
									KYVLPNFEVK	13	N-Term(iTRAQ4plex); K1(iTRAQ4plex); K10(iTRAQ4plex)		5.24	58	3	557.01
									ALEILQEEDLIDEDDIPVR	25	N-Term(iTRAQ4plex		4.92	93	2	1185.12
									LNMGITDLQGLR	6	N-Term(iTRAQ4plex		4.87	61	2	737.91
									TEQWSTLPPETK	31	N-Term(iTRAQ4plex); K12(iTRAQ4plex		4.86	82	2	852.96
									AEFQDALEK	38	N-Term(iTRAQ4plex); K9(iTRAQ4plex		4.78	68	2	669.86
									GLCVATPVQLR	25	N-Term(iTRAQ4plex); C3(Methylthio		4.74	65	2	673.87
									YIYGKPVQGVAYVF	3	N-Term(iTRAQ4plex); K5(iTRAQ4plex		4.71	58	2	951.05
									FGLLDEDGKK	24	N-Term(iTRAQ4plex); K9(iTRAQ4plex); K10(iTRAQ4plex)		4.6	70	2	777.45
									VDVQAGACEGK	16	N-Term(iTRAQ4plex); C8(Methylthio); K11(iTRAQ4plex)		4.6	73	2	705.85
									VGDTLNLNLR	33	N-Term(iTRAQ4plex		4.59	57	2	629.86
									EPFLSCCQFAESLR	2	N-Term(iTRAQ4plex); C6(Methylthio); C7(Methylthio)		4.48	69	2	933.42
									SFFPENWLWR	15	N-Term(iTRAQ4plex		4.34	58	2	763.39
									EMSGSPASGIPVK	2	N-Term(iTRAQ4plex); M2(Oxidation); K13(iTRAQ4plex)		4.27	57	2	782.42
									LELSVDGAK	29	N-Term(iTRAQ4plex); K9(iTRAQ4plex		4.23	68	2	610.35
									ITQVLHFTK	18	N-Term(iTRAQ4plex); K9(iTRAQ4plex		4.23	77	2	687.92
									VDFTLSSER	32	N-Term(iTRAQ4plex		4.17	56	2	599.31
									LNMGITDLQGLR	4	N-Term(iTRAQ4plex); M3(Oxidation		4.16	66	2	745.91
									VEYGFQVK	30	N-Term(iTRAQ4plex); K8(iTRAQ4plex		4.16	51	2	629.36
									CSVFYGAPSK	29	N-Term(iTRAQ4plex); C1(Methylthio); K10(iTRAQ4plex)		4.12	70	2	696.85
									TYNVLDMK	21	N-Term(iTRAQ4plex); K8(iTRAQ4plex		4.09	46	2	636.35
									SCGLHQLLR	17	N-Term(iTRAQ4plex); C2(Methylthio		3.95	53	2	608.82
									FGLLDEDGK	12	N-Term(iTRAQ4plex); K9(iTRAQ4plex		3.87	48	2	641.35
									LGQYASPTAK	3	N-Term(iTRAQ4plex); K10(iTRAQ4plex		3.86	79	2	662.38
									HLVPGAPFLLQALVR	9	N-Term(iTRAQ4plex		3.78	58	3	592.37
									YVLPNFEVK	20	N-Term(iTRAQ4plex); K9(iTRAQ4plex		3.74	65	2	698.91
									LQETSNWLLSQQQADGSFQDPCPVLDR	2	N-Term(iTRAQ4plex); C22(Methylthio		3.56	42	3	1089.19
									TLEIPGNSDPNMIPDGDFNSYVF	4	N-Term(iTRAQ4plex		3.53	40	3	899.10
									VHYTVCIWR	4	N-Term(iTRAQ4plex); C6(Methylthio		3.48	36	3	456.24
									VFALDQK	17	N-Term(iTRAQ4plex); K7(iTRAQ4plex		3.47	42	2	554.83
									ANSFLGEK	2	N-Term(iTRAQ4plex); K8(iTRAQ4plex			47		
									AVGSGATFSHYYYMILSF	1	N-Term(iTRAQ4plex); M14(Oxidation			42		
									VLQIEK	1	N-Term(iTRAQ4plex); K6(iTRAQ4plex			42		
									ECVGFEAVQEVPVGLVQPASATLYDYYI PER	1	N-Term(iTRAQ4plex); C2(Methylthio			31		
221316599	CHGB	secretogranin-1 precursor	39.44	21	21	340	1.024	78.2								
									KELENLAAMDLELQK	7	N-Term(iTRAQ4plex); K1(iTRAQ4plex); K15(iTRAQ4plex)	1.001	7.49	84	3	726.41
									HLEEPGETQNAFLNEF	50	N-Term(iTRAQ4plex	1.006	7.21	98	3	676.67
									ELENLAAMDLELQK	52	N-Term(iTRAQ4plex); K14(iTRAQ4plex	1.267	7.19	94	2	953.02
									GHPQEESEESNVSMASLGEK	9	N-Term(iTRAQ4plex); K20(iTRAQ4plex	0.939	7.06	92	3	811.72
									SQEESEEGEEDATSEVDK	3	N-Term(iTRAQ4plex); K18(iTRAQ4plex	0.861	6.77	113	2	1143.50
									SQEESEEGEEDATSEVDKR	6	N-Term(iTRAQ4plex); K18(iTRAQ4plex	0.772	6.23	51	3	814.71
									GEAGAPGEEDIQGPTK	47	N-Term(iTRAQ4plex); K16(iTRAQ4plex	0.93	6.02	106	2	922.47
									NYLNYGEEGAPGK	24	N-Term(iTRAQ4plex); K13(iTRAQ4plex	1.299	5.69	68	2	850.43
									ASEEEPEYGEEIK	39	N-Term(iTRAQ4plex); K13(iTRAQ4plex	0.939	5.61	98	2	899.43
									GYPGVQAPEDLEWER	24	N-Term(iTRAQ4plex	1.055	5.6	82	2	945.46
									WQQQGDLQDTK	26	N-Term(iTRAQ4plex); K11(iTRAQ4plex	0.949	5.48	81	2	817.93
									VAQLDQLLHYR	4	N-Term(iTRAQ4plex	1.221	5.3	47	3	500.62
									ADEPQWSLYPSDSQVSEEVK	6	N-Term(iTRAQ4plex); K20(iTRAQ4plex	0.969	4.97	84	2	1291.63
									ADQTVLTEDEK	7	N-Term(iTRAQ4plex); K11(iTRAQ4plex	1.026	4.81	70	2	768.91
									SAEFPDFYDSEEPVSTHQEAENEKDF	9	N-Term(iTRAQ4plex); K24(iTRAQ4plex	1.631	4.55	56	3	1115.50
									SSAPPITPECR	2	N-Term(iTRAQ4plex); C10(Methylthio	1.172	4.26	60	2	674.33
									SAEFPDFYDSEEPVSTHQEAENEK	3	N-Term(iTRAQ4plex); K24(iTRAQ4plex	2.107	4.1	69	3	1025.13
									NYPSLELDK	15	N-Term(iTRAQ4plex); K9(iTRAQ4plex	1.329	3.82	59	2	683.87
									SETHAAGHSQEK	2	N-Term(iTRAQ4plex); K12(iTRAQ4plex	1.072	3.72	40	4	393.20
									ELENLAAMDLELQK	2	N-Term(iTRAQ4plex); M8(Oxidation); K14(iTRAQ4plex)	1.149		77		
									GHPQEESEESNVSMASLGEK	1	N-Term(iTRAQ4plex); M14(Oxidation); K20(iTRAQ4plex)	1.079		66		
									MAHGYGEESEEER	1	N-Term(iTRAQ4plex	1.025		48		
									SQREDEEEEEGENYQK	1	N-Term(iTRAQ4plex); K16(iTRAQ4plex	1.163		35		
11128019	CYCS	cytochrome c	38.1	4	4	23	0.885	11.7								

									TGPNLHGLFGR	9	N-Term(iTRAQ4plex	0.887	4.48	67	2	656.87
									ADLIAYLK	10	N-Term(iTRAQ4plex); K8(iTRAQ4plex	0.89	3.62	59	2	597.87
									MIFVGIK	3	N-Term(iTRAQ4plex); K7(iTRAQ4plex	0.847	3.2	43	2	548.34
									TGQAPGYSYTAANK	1	N-Term(iTRAQ4plex); K14(iTRAQ4plex	0.97		60		
50659080	SERPINA3	alpha-1-antichymotrypsin precursor	38.06	14	14	450	1.016	47.6								
									LYGSEAFATDFQDSAAAK	31	N-Term(iTRAQ4plex); K18(iTRAQ4plex	1.093	8.8	102	2	1090.54
									DEELSCTVVELK	40	N-Term(iTRAQ4plex); C6(Methylthio); K12(iTRAQ4plex)	0.985	6.08	93	2	849.93
									WEMPFDPQDTHQSR	68	N-Term(iTRAQ4plex	0.989	6	75	3	639.96
									NLAVSQVVHK	42	N-Term(iTRAQ4plex); K10(iTRAQ4plex	0.97	5.67	75	3	461.62
									WEMPFDPQDTHQSR	23	N-Term(iTRAQ4plex); M3(Oxidation	1.003	5.4	73	3	645.29
									MEEVEAMLLPETLK	25	N-Term(iTRAQ4plex); K14(iTRAQ4plex	1.017	4.9	79	2	961.02
									KLINDYVK	23	N-Term(iTRAQ4plex); K1(iTRAQ4plex); K8(iTRAQ4plex)	1.055	4.82	51	3	475.63
									MEEVEAMLLPETLK	2	N-Term(iTRAQ4plex); M1(Oxidation); M7(Oxidation); K14(iTRAQ4plex)	1.054	4.55	67	2	977.01
									ADLSGITGAR	20	N-Term(iTRAQ4plex	0.965	4.25	69	2	552.81
									ITLLSALVETR	59	N-Term(iTRAQ4plex	1.056	4.19	72	2	680.42
									MEEVEAMLLPETLK	11	N-Term(iTRAQ4plex); M1(Oxidation); K14(iTRAQ4plex)	0.957	4.18	61	2	969.01
									EQLSLLDR	46	N-Term(iTRAQ4plex	1.064	3.53	46	2	559.32
									LINDYVK	26	N-Term(iTRAQ4plex); K7(iTRAQ4plex	1.037	3.35	52	2	576.85
									EIGELYLPK	16	N-Term(iTRAQ4plex); K9(iTRAQ4plex	1.086	3.21	49	2	675.39
									AVLDVFEEGTEASAATAVI	2	N-Term(iTRAQ4plex); K19(iTRAQ4plex	1.083		45		
									ITDLIK	15	N-Term(iTRAQ4plex); K6(iTRAQ4plex	0.895		44		
									FNRPFLMIIVPTDTQNIFFMSK	1	N-Term(iTRAQ4plex); K22(iTRAQ4plex	1.152		38		
4506773	S100A9	protein S100-A9	37.72	3	3	5	0.903	13.2								
									LGHPDTLNQGEFK	3	N-Term(iTRAQ4plex); K13(iTRAQ4plex	0.931	4.62	73	3	581.98
									NIETIINTFHQYSVK	1	N-Term(iTRAQ4plex); K15(iTRAQ4plex	0.917		54		
									VIEHIMEDLDTNADK	1	N-Term(iTRAQ4plex); K15(iTRAQ4plex	0.684		43		
189083772	GSN	gelsolin isoform b	37.35	1	22	168	0.748	80.6								
									AGALNSNDAFVLK	22	N-Term(iTRAQ4plex); K13(iTRAQ4plex		7	90	2	804.45
									DPDQTDGLGLSYLSSHIANVER	18	N-Term(iTRAQ4plex		6.86	116	3	844.42
									DSQEEEKTEALTSAK	8	N-Term(iTRAQ4plex); K7(iTRAQ4plex); K15(iTRAQ4plex)		6.62	85	2	1049.55
									VPFDAATLHTSTAMAAQHGMDDDGTGQ	3	N-Term(iTRAQ4plex); K28(iTRAQ4plex		6.16	34	4	791.13
									TPSAAYLWVGTGASEAEK	12	N-Term(iTRAQ4plex); K18(iTRAQ4plex		6.05	107	2	1063.56
									EVQGFESATFLGYFK	16	N-Term(iTRAQ4plex); K15(iTRAQ4plex		6	93	2	1006.03
									QTQVSVLPEGGETPLFK	10	N-Term(iTRAQ4plex); K17(iTRAQ4plex		5.4	68	2	1059.59
									SEDCFILDHGK	39	N-Term(iTRAQ4plex); C4(Methylthio); K11(iTRAQ4plex)		5.34	83	2	799.38
									VSNGAGTMSVSLVADENPFAQGALI	2	N-Term(iTRAQ4plex); K25(iTRAQ4plex		5.03	59	3	917.81
									AQPVQVAEGSEPDGFWEALGGK	8	N-Term(iTRAQ4plex); K22(iTRAQ4plex		4.69	88	2	1280.65
									TASDFITK	4	N-Term(iTRAQ4plex); K8(iTRAQ4plex		3.73	48	2	585.83
									AVEVLPK	4	N-Term(iTRAQ4plex); K7(iTRAQ4plex		3.15	45	2	522.34
									NWRDPDQTDGLGLSYLSSHIANVER	1	N-Term(iTRAQ4plex			60		
									VVEHPEFLK	1	N-Term(iTRAQ4plex); K9(iTRAQ4plex	0.748		55		
									HVVPNEVVVQR	1	N-Term(iTRAQ4plex			46		
									AMAELAA	1	N-Term(iTRAQ4plex			46		
									LFACSNK	1	N-Term(iTRAQ4plex); C4(Methylthio); K7(iTRAQ4plex)			39		
									TGAQELLR	1	N-Term(iTRAQ4plex			39		
									EPGLQIWR	12	N-Term(iTRAQ4plex			38		
									TPITVVK	2	N-Term(iTRAQ4plex); K7(iTRAQ4plex			38		
									AGKEPGLQIWR	1	N-Term(iTRAQ4plex); K3(iTRAQ4plex			37		
									YIETDPANR	1	N-Term(iTRAQ4plex			34		
20070228	NUCB1	nucleobindin-1 precursor	37.31	12	12	40	1.087	53.8								
									ELQQAVLHMEQR	13	N-Term(iTRAQ4plex	1.126	5.4	68	2	813.43
									ELQQAVLHMEQR	2	N-Term(iTRAQ4plex); M9(Oxidation	1.047	4.74	68	3	547.96
									YLESLGEEQR	2	N-Term(iTRAQ4plex	1.048	4.3	51	2	684.35
									VNVPGSQAQLK	2	N-Term(iTRAQ4plex); K11(iTRAQ4plex	1.247	4.2	83	2	714.92
									MDAEQDPNVQVDHLNLLK	4	N-Term(iTRAQ4plex); K18(iTRAQ4plex	1.3	4.2	65	3	789.75
									QFEHLDPQNQHTFEAR	2	N-Term(iTRAQ4plex	1.17	4.08	44	3	714.35
									FHPDTDDVPVPAPAGDQK	5	N-Term(iTRAQ4plex); K18(iTRAQ4plex	0.833	3.76	58	2	1097.55
									EFGDTGEGWETVEMHPAYTEEELR	2	N-Term(iTRAQ4plex	0.824	3.69	63	3	986.11
									DLAQYDAAHHEEFK	2	N-Term(iTRAQ4plex); K14(iTRAQ4plex	0.827	3.49	41	4	491.24
									EVWEELDGLDPNR	2	N-Term(iTRAQ4plex	0.867	3.23	48	2	858.42
									LVTLEEFLASTQR	2	N-Term(iTRAQ4plex	1.132		40		
									LPEVEVPQHL	1	N-Term(iTRAQ4plex	0.982		35		
									NEEDDMREMEEER	1	N-Term(iTRAQ4plex	1.059		27		
7019519	PCSK1N	proSAAS precursor	37.31	6	7	55	0.867	27.4								
									AADHDVGSELPPEGVLGALLR	4	N-Term(iTRAQ4plex	1.556	5.87	58	3	754.08
									NSDPALGLDDDPDAPAAQLAR	13	N-Term(iTRAQ4plex	0.803	5.67	110	2	1133.56
									ILAGSADSEGVAAPR	14	N-Term(iTRAQ4plex	0.891	5.57	96	2	779.42
									GEAAGAVQELAR	10	N-Term(iTRAQ4plex	0.761	5.4	86	2	658.36
									ALAHLLEAER	10	N-Term(iTRAQ4plex	0.848	4.06	53	2	633.87
									LETPAPQVPAR	2	N-Term(iTRAQ4plex	1.023	3.5	48	2	661.88
									VLAQLLR	2	N-Term(iTRAQ4plex			32		
66346706	NEGR1	neuronal growth regulator 1 precursor	37.29	9	9	52	0.925	38.7								
									TMQVHLTVQVPPK	15	N-Term(iTRAQ4plex); K13(iTRAQ4plex	0.967	6.03	60	3	589.35
									VVVNFAPTIQEIK	15	N-Term(iTRAQ4plex); K13(iTRAQ4plex	0.909	5.36	92	2	873.53
									DYSLQIQNVDVTDDGPYTCSVQTQHTPI	2	N-Term(iTRAQ4plex); C19(Methylthio	0.937	5.18	84	3	1124.19
									TMQVHLTVQVPPK	2	N-Term(iTRAQ4plex); M2(Oxidation); K13(iTRAQ4plex)	0.965	5.05	47	3	594.68
									CEGAGVPPPAFEWYK	7	N-Term(iTRAQ4plex); C1(Methylthio); K15(iTRAQ4plex)	0.971	4.62	55	2	992.98
									SSIIFAGGDK	4	N-Term(iTRAQ4plex); K10(iTRAQ4plex	1.035	4.12	54	2	641.87
									CYLEDGASK	2	N-Term(iTRAQ4plex); C1(Methylthio); K9(iTRAQ4plex)	0.946	3.59	44	2	660.32
									VSISTLNK	2	N-Term(iTRAQ4plex); K8(iTRAQ4plex	0.888	3.54	65	2	575.35
									DQAGEYECSAENDVSFPDVR	2	N-Term(iTRAQ4plex); C8(Methylthio	0.814		59		
									LFNGQGIIIQNFSTR	1	N-Term(iTRAQ4plex	0.776		45		
10863927	PPIA	peptidyl-prolyl cis-trans isomerase A	36.97	4	6	23	0.824	18								
									EGMNIVEAMER	4	N-Term(iTRAQ4plex	0.824	5.07	61	2	711.85
									IIPGFMCQGGDFTR	2	N-Term(iTRAQ4plex); C7(Methylthio		4.36	69	2	866.41
									VSFELFADK	5	N-Term(iTRAQ4plex); K9(iTRAQ4plex	0.693	4.07	59	2	672.38

									FEDENFILK	4	N-Term(iTRAQ4plex); K9(iTRAQ4plex	0.682	4	52	2	721.89
									KITIADCGQLE	4	N-Term(iTRAQ4plex); K1(iTRAQ4plex); C7(Methylthio)	0.865	3.69	65	2	762.91
									TEWLDGK	3	N-Term(iTRAQ4plex); K7(iTRAQ4plex		3.28	47	2	568.82
									EGMNIVEAMER	1	N-Term(iTRAQ4plex); M3(Oxidation	0.918		39		
28373119	CNTN1	contactin-1 isoform 2 precursor	36.94	0	29	210		111.8								
									TTKPYPADIVVQFK	11	N-Term(iTRAQ4plex); K3(iTRAQ4plex); K14(iTRAQ4plex)		7.4	65	3	680.41
									YVHKDETMSPSTAFQVK	4	N-Term(iTRAQ4plex); K4(iTRAQ4plex); K17(iTRAQ4plex)		7.23	79	3	800.76
									STEATLSFGYLDPFPPEERPEVR	6	N-Term(iTRAQ4plex		6.69	71	3	927.80
									YSMVGGNLVINNPDK	10	N-Term(iTRAQ4plex); K15(iTRAQ4plex		6.53	85	2	955.01
									DETMSPSTAFQVK	7	N-Term(iTRAQ4plex); K13(iTRAQ4plex		6.13	95	2	864.94
									WLLNEFPVFITMDK	4	N-Term(iTRAQ4plex); K14(iTRAQ4plex		5.86	78	2	1021.06
									YSMVGGNLVINNPDK	4	N-Term(iTRAQ4plex); M3(Oxidation); K15(iTRAQ4plex)		5.64	87	2	963.00
									VLEPMPSTAEISTSGAVLK	4	N-Term(iTRAQ4plex); K19(iTRAQ4plex		5.58	75	2	1109.62
									YWAAHDKEEANR	7	N-Term(iTRAQ4plex); K7(iTRAQ4plex		5.54	69	2	924.97
									TDGAAPNVAPSDVGGGGGR	19	N-Term(iTRAQ4plex		5.51	106	2	899.95
									NDGGIYTCFAENNR	6	N-Term(iTRAQ4plex); C8(Methylthio		5.2	82	2	882.39
									NFMLDSNGELLIR	6	N-Term(iTRAQ4plex		5	65	2	833.44
									ILALAPTFEMNPMK	9	N-Term(iTRAQ4plex); K14(iTRAQ4plex		4.85	61	2	932.52
									TDPPIIEGNMEAAR	16	N-Term(iTRAQ4plex		4.65	72	2	829.43
									AVDLIPWMEYEFR	6	N-Term(iTRAQ4plex		4.56	63	2	906.96
									ELTITWAPLSR	7	N-Term(iTRAQ4plex		4.55	58	2	715.91
									VIIECKPK	6	N-Term(iTRAQ4plex); C5(Methylthio); K6(iTRAQ4plex); K8(iTRAQ4plex		4.05	33	3	469.96
									KVLEPMPSTAEISTSGAVLK	4	N-Term(iTRAQ4plex); K1(iTRAQ4plex); K20(iTRAQ4plex)		3.99	50	3	830.82
									MNNGDVDLTSDR	2	N-Term(iTRAQ4plex		3.95	52	2	740.85
									DGEYVVEVR	4	N-Term(iTRAQ4plex		3.62	57	2	605.32
									ASPFPVYK	14	N-Term(iTRAQ4plex); K8(iTRAQ4plex		3.62	55	2	598.85
									TILSDDWK	16	N-Term(iTRAQ4plex); K8(iTRAQ4plex		3.47	42	2	633.35
									FIPLIPIPER	15	N-Term(iTRAQ4plex		3.42	46	2	669.92
									DAGIYYCLASNNYGMVR	2	N-Term(iTRAQ4plex); C7(Methylthio		3.31	48	2	1050.48
									ILALAPTFEMNPMK	3	N-Term(iTRAQ4plex); M10(Oxidation); K14(iTRAQ4plex)		3.24	54	2	940.52
									DETMSPSTAFQVK	1	N-Term(iTRAQ4plex); M4(Oxidation); K13(iTRAQ4plex)			72		
									ATSVALTWSR	2	N-Term(iTRAQ4plex			54		
									TDPPIIEGNMEAAR	3	N-Term(iTRAQ4plex); M10(Oxidation			51		
									NFMLDSNGELLIR	1	N-Term(iTRAQ4plex); M3(Oxidation			47		
									HSIEVPIPR	7	N-Term(iTRAQ4plex			44		
									GFGPIFEEQPINTIYPEESLEGK	1	N-Term(iTRAQ4plex); K23(iTRAQ4plex			42		
									VLSSSEISVHWEHVLEK	1	N-Term(iTRAQ4plex); K17(iTRAQ4plex			41		
									GMVLLCDPPYHFPDDLSYR	1	N-Term(iTRAQ4plex); C6(Methylthio			30		
28373117	CNTN1	contactin-1 isoform 1 precursor	36.54	0	29	210		113.2								
									TTKPYPADIVVQFK	11	N-Term(iTRAQ4plex); K3(iTRAQ4plex); K14(iTRAQ4plex)		7.4	65	3	680.41
									YVHKDETMSPSTAFQVK	4	N-Term(iTRAQ4plex); K4(iTRAQ4plex); K17(iTRAQ4plex)		7.23	79	3	800.76
									STEATLSFGYLDPFPPEERPEVR	6	N-Term(iTRAQ4plex		6.69	71	3	927.80
									YSMVGGNLVINNPDK	10	N-Term(iTRAQ4plex); K15(iTRAQ4plex		6.53	85	2	955.01
									DETMSPSTAFQVK	7	N-Term(iTRAQ4plex); K13(iTRAQ4plex		6.13	95	2	864.94
									WLLNEFPVFITMDK	4	N-Term(iTRAQ4plex); K14(iTRAQ4plex		5.86	78	2	1021.06
									YSMVGGNLVINNPDK	4	N-Term(iTRAQ4plex); M3(Oxidation); K15(iTRAQ4plex)		5.64	87	2	963.00
									VLEPMPSTAEISTSGAVLK	4	N-Term(iTRAQ4plex); K19(iTRAQ4plex		5.58	75	2	1109.62
									YWAAHDKEEANR	7	N-Term(iTRAQ4plex); K7(iTRAQ4plex		5.54	69	2	924.97
									TDGAAPNVAPSDVGGGGGR	19	N-Term(iTRAQ4plex		5.51	106	2	899.95
									NDGGIYTCFAENNR	6	N-Term(iTRAQ4plex); C8(Methylthio		5.2	82	2	882.39
									NFMLDSNGELLIR	6	N-Term(iTRAQ4plex		5	65	2	833.44
									ILALAPTFEMNPMK	9	N-Term(iTRAQ4plex); K14(iTRAQ4plex		4.85	61	2	932.52
									TDPPIIEGNMEAAR	16	N-Term(iTRAQ4plex		4.65	72	2	829.43
									AVDLIPWMEYEFR	6	N-Term(iTRAQ4plex		4.56	63	2	906.96
									ELTITWAPLSR	7	N-Term(iTRAQ4plex		4.55	58	2	715.91
									VIIECKPK	6	N-Term(iTRAQ4plex); C5(Methylthio); K6(iTRAQ4plex); K8(iTRAQ4plex		4.05	33	3	469.96
									KVLEPMPSTAEISTSGAVLK	4	N-Term(iTRAQ4plex); K1(iTRAQ4plex); K20(iTRAQ4plex)		3.99	50	3	830.82
									MNNGDVDLTSDR	2	N-Term(iTRAQ4plex		3.95	52	2	740.85
									DGEYVVEVR	4	N-Term(iTRAQ4plex		3.62	57	2	605.32
									ASPFPVYK	14	N-Term(iTRAQ4plex); K8(iTRAQ4plex		3.62	55	2	598.85
									TILSDDWK	16	N-Term(iTRAQ4plex); K8(iTRAQ4plex		3.47	42	2	633.35
									FIPLIPIPER	15	N-Term(iTRAQ4plex		3.42	46	2	669.92
									DAGIYYCLASNNYGMVR	2	N-Term(iTRAQ4plex); C7(Methylthio		3.31	48	2	1050.48
									ILALAPTFEMNPMK	3	N-Term(iTRAQ4plex); M10(Oxidation); K14(iTRAQ4plex)		3.24	54	2	940.52
									DETMSPSTAFQVK	1	N-Term(iTRAQ4plex); M4(Oxidation); K13(iTRAQ4plex)			72		
									ATSVALTWSR	2	N-Term(iTRAQ4plex			54		
									TDPPIIEGNMEAAR	3	N-Term(iTRAQ4plex); M10(Oxidation			51		
									NFMLDSNGELLIR	1	N-Term(iTRAQ4plex); M3(Oxidation			47		
									HSIEVPIPR	7	N-Term(iTRAQ4plex			44		
									GFGPIFEEQPINTIYPEESLEGK	1	N-Term(iTRAQ4plex); K23(iTRAQ4plex			42		
									VLSSSEISVHWEHVLEK	1	N-Term(iTRAQ4plex); K17(iTRAQ4plex			41		
									GMVLLCDPPYHFPDDLSYR	1	N-Term(iTRAQ4plex); C6(Methylthio			30		
310118062	LOC100509001	PREDICTED: complement C4-B-like isoform 2	36.52	0	8	108		40.8								
									YVSHFETEGPHVLLYFDSVPTSR	15	N-Term(iTRAQ4plex		7.3	71	3	942.14
									VGLSGMAIADVTLLSGFHALR	4	N-Term(iTRAQ4plex		5.38	71	3	758.09
									DDPDAPLQPVTPLQLFEGR	7	N-Term(iTRAQ4plex		5.3	73	2	1126.59
									ITQVLHFTK	18	N-Term(iTRAQ4plex); K9(iTRAQ4plex		4.23	77	2	687.92
									VEYGFQVK	30	N-Term(iTRAQ4plex); K8(iTRAQ4plex		4.16	51	2	629.36
									CSVFYGAPSK	29	N-Term(iTRAQ4plex); C1(Methylthio); K10(iTRAQ4plex)		4.12	70	2	696.85
									VHYTVCIWR	4	N-Term(iTRAQ4plex); C6(Methylthio		3.48	36	3	456.24
									ECVGFEAVQEVPVGLVQPASATLYDYYI PER	1	N-Term(iTRAQ4plex); C2(Methylthio			31		

167857790	ORM1	alpha-1-acid glycoprotein 1 precursor	36.32	5	6	21	0.285	23.5								
									NWGLSVYADKPETTK	3	N-Term(iTRAQ4plex); K10(iTRAQ4plex); K15(iTRAQ4plex)	0.285	4.51	38	3	714.40
									YVGGQEHFAHLLILR	3	N-Term(iTRAQ4plex	0.229	4.51	65	3	633.02
									TYMLAFDVNDEK	4	N-Term(iTRAQ4plex); K12(iTRAQ4plex	0.235	4.42	98	2	867.44
									SDVVYTDWK	8	N-Term(iTRAQ4plex); K9(iTRAQ4plex	0.36	3.89	71	2	700.87
									EQLGEFYEALDCLR	1	N-Term(iTRAQ4plex); C12(Methylthio	0.308		34		
190341024	SPARCL1	SPARC-like protein 1 precursor	35.99	16	16	150	0.984	75.2								
									NILMQLYEANSEHAGYLNEK	12	N-Term(iTRAQ4plex); K20(iTRAQ4plex	1.383	9.12	87	3	875.78
									DQGNQEQDPNISNGEEEEEKEPGEVGTHNDNQER	7	N-Term(iTRAQ4plex); K20(iTRAQ4plex	1.167	8.22	35	4	1035.71
									TVSEALLMEPTDDGNTTPR	18	N-Term(iTRAQ4plex	1.023	5.84	96	2	1096.04
									VLTHSELAPLR	45	N-Term(iTRAQ4plex	0.935	5.52	60	3	460.61
									TVSEALLMEPTDDGNTTPR	6	N-Term(iTRAQ4plex); M8(Oxidation	0.932	5.46	78	2	1104.04
									SIPTCTDFEVIQFPLR	2	N-Term(iTRAQ4plex); C5(Methylthio	0.977	5.38	73	2	1028.52
									GHQLQLDYFGACK	2	N-Term(iTRAQ4plex); C12(Methylthio); K13(iTRAQ4plex)	1.06	5.32	45	2	907.45
									LLAGDHPIDLLLR	15	N-Term(iTRAQ4plex	1.048	4.83	74	2	795.48
									KLSENTDFLAPGVSSFTDSNQQESITK	3	N-Term(iTRAQ4plex); K1(iTRAQ4plex); K27(iTRAQ4plex)	0.85	4.76	51	3	1125.91
									ASLVPMEHCITR	17	N-Term(iTRAQ4plex); C9(Methylthio	0.963	4.38	59	3	516.26
									VHAVDSCMSFQCK	2	N-Term(iTRAQ4plex); C7(Methylthio); C12(Methylthio); K13(iTRAQ4plex)	0.947	4.35	44	3	612.27
									HSASDDYFIPSQAFLEAER	5	N-Term(iTRAQ4plex	1.084	4.32	50	3	776.38
									FFEECDPNKDK	4	N-Term(iTRAQ4plex); C5(Methylthio); K9(iTRAQ4plex); K11(iTRAQ4plex)	1.037	4.18	53	2	925.45
									EWGHCFGIK	5	N-Term(iTRAQ4plex); C5(Methylthio); K9(iTRAQ4plex)	1.072	3.83	41	3	470.90
									FFEECDPNK	3	N-Term(iTRAQ4plex); C5(Methylthio); K9(iTRAQ4plex)	1.198	3.55	46	2	731.83
									HIQETEWQSQEGK	2	N-Term(iTRAQ4plex); K13(iTRAQ4plex	0.791	3.37	43	3	629.99
77539055	UBA52	ubiquitin-60S ribosomal protein L40 precursor	35.94	0	5	96		14.7								
									TITLEVEPSDTIENVK	20	N-Term(iTRAQ4plex); K16(iTRAQ4plex		5.99	89	2	1038.57
									LIFAGKQLEDGR	16	N-Term(iTRAQ4plex); K6(iTRAQ4plex		5	73	2	817.98
									TLSDYNIQK	22	N-Term(iTRAQ4plex); K9(iTRAQ4plex		4.71	67	2	685.38
									ESTLHLVLR	37	N-Term(iTRAQ4plex		4.63	58	2	606.36
									TLSDYNIQKESTLHLVLR	1	N-Term(iTRAQ4plex); K9(iTRAQ4plex			35		
310118060	LOC100509001	PREDICTED: complement C4-B-like isoform 1	35.86	0	12	164		59.3								
									YVSHFETEGPHVLLYFDSVPTSR	15	N-Term(iTRAQ4plex		7.3	71	3	942.14
									STQDTVIALDALSAYWIASHTTEER	13	N-Term(iTRAQ4plex		7.07	112	3	974.83
									GLEEELQFSLGSK	12	N-Term(iTRAQ4plex); K13(iTRAQ4plex		6.56	93	2	862.97
									VGLSGMAIADVTLLSGFHALR	4	N-Term(iTRAQ4plex		5.38	71	3	758.09
									DDPDAPLQPVTPLQLFEGR	7	N-Term(iTRAQ4plex		5.3	73	2	1126.59
									AEMADQAAAWLTR	8	N-Term(iTRAQ4plex); M3(Oxidation		5.14	80	2	797.39
									AEMADQAAAWLTR	2	N-Term(iTRAQ4plex		5.12	80	2	789.40
									ITQVLHFTK	18	N-Term(iTRAQ4plex); K9(iTRAQ4plex		4.23	77	2	687.92
									VEYGFQVK	30	N-Term(iTRAQ4plex); K8(iTRAQ4plex		4.16	51	2	629.36
									CSVFYGAPSK	29	N-Term(iTRAQ4plex); C1(Methylthio); K10(iTRAQ4plex)		4.12	70	2	696.85
									TYNVLDMK	21	N-Term(iTRAQ4plex); K8(iTRAQ4plex		4.09	46	2	636.35
									VHYTVCIWR	4	N-Term(iTRAQ4plex); C6(Methylthio		3.48	36	3	456.24
									ECVGFEAVQEVPVGLVQPASATLYDYYNPER	1	N-Term(iTRAQ4plex); C2(Methylthio			31		
4758950	PPIB	peptidyl-prolyl cis-trans isomerase B precursor	35.65	7	7	33	0.985	23.7								
									TVDNFVALATGEK	5	N-Term(iTRAQ4plex); K13(iTRAQ4plex	0.939	5.67	87	2	826.96
									IEVEKPFAIAK	2	N-Term(iTRAQ4plex); K5(iTRAQ4plex); K11(iTRAQ4plex)	1	5.27	56	2	839.01
									HYGPGWVSMANAGK	2	N-Term(iTRAQ4plex); K14(iTRAQ4plex	0.962	4.19	100	2	881.95
									VIFGLFGK	12	N-Term(iTRAQ4plex); K8(iTRAQ4plex	1.088	3.82	50	2	584.87
									VLEGMEVVR	4	N-Term(iTRAQ4plex	0.971	3.78	46	2	588.33
									DVIIADCGK	6	N-Term(iTRAQ4plex); C7(Methylthio); K9(iTRAQ4plex)	0.894	3.62	57	2	634.33
									DTNGSQFFITTVK	2	N-Term(iTRAQ4plex); K13(iTRAQ4plex	0.859	3.39	74	2	873.47
189083780	GSN	gelsolin isoform c	35.58	0	21	167		81.9								
									AGALNSNDAFVLK	22	N-Term(iTRAQ4plex); K13(iTRAQ4plex		7	90	2	804.45
									DPDQTDGLGLSYLSSHIANVER	18	N-Term(iTRAQ4plex		6.86	116	3	844.42
									DSQEEEKTEALTSAK	8	N-Term(iTRAQ4plex); K7(iTRAQ4plex); K15(iTRAQ4plex)		6.62	85	2	1049.55
									VPFDAATLHTSTAMAAQHGMDDDGTGQK	3	N-Term(iTRAQ4plex); K28(iTRAQ4plex		6.16	34	4	791.13
									TPSAAYLWVGTGASEAEK	12	N-Term(iTRAQ4plex); K18(iTRAQ4plex		6.05	107	2	1063.56
									EVQGFESATFLGYFK	16	N-Term(iTRAQ4plex); K15(iTRAQ4plex		6	93	2	1006.03
									QTQVSVLPEGGETPLFK	10	N-Term(iTRAQ4plex); K17(iTRAQ4plex		5.4	68	2	1059.59
									SEDCFILDHGK	39	N-Term(iTRAQ4plex); C4(Methylthio); K11(iTRAQ4plex)		5.34	83	2	799.38
									VSNGAGTMSVSLVADENPFAQGALK	2	N-Term(iTRAQ4plex); K25(iTRAQ4plex		5.03	59	3	917.81
									AQPVQVAEGSEPDGFWEALGGK	8	N-Term(iTRAQ4plex); K22(iTRAQ4plex		4.69	88	2	1280.65
									TASDFITK	4	N-Term(iTRAQ4plex); K8(iTRAQ4plex		3.73	48	2	585.83
									AVEVLPK	4	N-Term(iTRAQ4plex); K7(iTRAQ4plex		3.15	45	2	522.34
									NWRDPDQTDGLGLSYLSSHIANVER	1	N-Term(iTRAQ4plex			60		
									HVVPNEVVVQR	1	N-Term(iTRAQ4plex			46		
									AMAELAA	1	N-Term(iTRAQ4plex			46		
									LFACSNK	1	N-Term(iTRAQ4plex); C4(Methylthio); K7(iTRAQ4plex)			39		
									TGAQELLR	1	N-Term(iTRAQ4plex			39		
									EPGLQIWR	12	N-Term(iTRAQ4plex			38		
									TPITVVK	2	N-Term(iTRAQ4plex); K7(iTRAQ4plex			38		
									AGKEPGLQIWR	1	N-Term(iTRAQ4plex); K3(iTRAQ4plex			37		
									YIETDPANR	1	N-Term(iTRAQ4plex			34		
4557485	CP	ceruloplasmin precursor	35.49	28	28	408	0.95	122.1								
									GPEEEHLGILGPVIWAEVGDTIR	23	N-Term(iTRAQ4plex	0.971	7.7	104	3	877.81
									ELHHLQEQNVSNAFLDK	3	N-Term(iTRAQ4plex); K17(iTRAQ4plex	0.866	7.69	99	3	770.74
									AEEEHLGILGPQLHADVGDK	13	N-Term(iTRAQ4plex); K20(iTRAQ4plex	1.082	6.92	110	4	604.82
									VNKDDEEFIESNK	20	N-Term(iTRAQ4plex); K3(iTRAQ4plex); K13(iTRAQ4plex)	0.852	6.85	71	3	667.01
									LISVDTEHSNIYLQNGPDR	20	N-Term(iTRAQ4plex	0.941	6.68	95	2	1158.09
									NNEGTYYSPNYNPQSR	7	N-Term(iTRAQ4plex	1.003	6.62	86	2	1024.47
									SVPPSASHVAPTETFTYEWTVPK	13	N-Term(iTRAQ4plex); K23(iTRAQ4plex	1.12	6.53	81	3	940.49

									EVGPTNADPVCLAK	28	N-Term(iTRAQ4plex); C11(Methylthio); K14(iTRAQ4plex)	0.92	6.47	102	2	874.45
									DIASGLIGPLIICK	17	N-Term(iTRAQ4plex); C13(Methylthio); K14(iTRAQ4plex)	0.947	5.99	80	2	874.01
									MFTTAPDQVDKEDEDFQESNK	3	N-Term(iTRAQ4plex); M1(Oxidation); K11(iTRAQ4plex); K21(iTRAQ4plex)	0.941	5.21	59	4	731.35
									DLYSGLIGPLIVCR	8	N-Term(iTRAQ4plex); C13(Methylthio	0.857	5.21	78	2	854.97
									GVYSSDVFDIFPGTYQTLEMFPR	2	N-Term(iTRAQ4plex); M20(Oxidation	0.832	5.07	39	3	943.79
									KAEEEHLGILGPQLHADVGDK	2	N-Term(iTRAQ4plex); K1(iTRAQ4plex); K21(iTRAQ4plex)	1.311	5.07	75	3	896.83
									ALYLQYTDETFR	29	N-Term(iTRAQ4plex	0.943	5.06	67	2	832.43
									DDEEFIESNK	6	N-Term(iTRAQ4plex); K10(iTRAQ4plex	1.002	5	74	2	757.36
									IYHSHIDAPK	2	N-Term(iTRAQ4plex); K10(iTRAQ4plex	0.858	4.98	54	3	490.28
									QSEDSTFYLGER	44	N-Term(iTRAQ4plex	0.926	4.94	74	2	788.38
									GAYPLSIEPIGVR	45	N-Term(iTRAQ4plex	0.953	4.75	78	2	758.44
									DIFTGLIGPMK	29	N-Term(iTRAQ4plex); K11(iTRAQ4plex	0.973	4.75	84	2	740.43
									MYYSAVDPTK	39	N-Term(iTRAQ4plex); K10(iTRAQ4plex	0.895	4.65	62	2	731.87
									DIFTGLIGPMK	10	N-Term(iTRAQ4plex); M10(Oxidation); K11(iTRAQ4plex)	0.952	4.29	60	2	748.43
									EYTDASFTNR	3	N-Term(iTRAQ4plex	0.853	4.01	46	2	674.32
									MYYSAVDPTK	2	N-Term(iTRAQ4plex); M1(Oxidation); K10(iTRAQ4plex)	0.915	3.98	52	2	739.88
									VTFHNKGAYPLSIEPIGVR	4	N-Term(iTRAQ4plex); K6(iTRAQ4plex	1.415	3.85	65	3	796.12
									GEFYIGSK	25	N-Term(iTRAQ4plex); K8(iTRAQ4plex	0.954	3.75	54	2	594.83
									GVYSSDVFDIFPGTYQTLEMFPR	3	N-Term(iTRAQ4plex	1.133	3.23	73	2	1407.19
									TTIEKPVWLGFLGPIIK	3	N-Term(iTRAQ4plex); K5(iTRAQ4plex); K17(iTRAQ4plex)	0.983		58		
									ERGPEEEHLGILGPVIWAEVGDTIR	1	N-Term(iTRAQ4plex			34		
									MYSVNGYTFGSLPGLSMCAEDR	1	N-Term(iTRAQ4plex); C18(Methylthio	0.885		30		
									TYCSEPEK	1	N-Term(iTRAQ4plex); C3(Methylthio); K8(iTRAQ4plex)	1.136		28		
19557645	SCG3	secretogranin-3 isoform 1 precursor	35.26	7	15	104	0.996	53								
									FQDDPDGLHQLDGTPLTAEDIVHK	12	N-Term(iTRAQ4plex); K24(iTRAQ4plex	0.889	9.34	95	4	738.13
									GENDETVSNTLTLTNGLER	6	N-Term(iTRAQ4plex		7.02	84	2	1104.05
									ELSAERPLNEQIAEAEEDK	9	N-Term(iTRAQ4plex); K19(iTRAQ4plex	1.076	7.01	64	3	820.42
									KLIDDYDSTK	7	N-Term(iTRAQ4plex); K1(iTRAQ4plex); K10(iTRAQ4plex)	1.042	5.92	70	3	543.97
									VTPMAAIQDGLAK	8	N-Term(iTRAQ4plex); K13(iTRAQ4plex	0.996	5.66	109	2	801.96
									GILDKEEAEAIK	10	N-Term(iTRAQ4plex); K5(iTRAQ4plex); K12(iTRAQ4plex)		5.53	73	2	874.52
									EKETLITIMK	4	N-Term(iTRAQ4plex); K2(iTRAQ4plex); K10(iTRAQ4plex)		4.75	62	2	819.49
									GKTEAYLEAIR	2	N-Term(iTRAQ4plex); K2(iTRAQ4plex		4.65	43	2	769.94
									LNVEDVDSTK	9	N-Term(iTRAQ4plex); K10(iTRAQ4plex	1.217	4.61	77	2	704.38
									TEAYLEAIR	14	N-Term(iTRAQ4plex		4.37	48	2	605.34
									EANNYEEDPNKPTSWTENQAGK	2	N-Term(iTRAQ4plex); K11(iTRAQ4plex); K22(iTRAQ4plex)	0.712	4.16	32	3	985.47
									VTPMAAIQDGLAK	3	N-Term(iTRAQ4plex); M4(Oxidation); K13(iTRAQ4plex)	0.917	4.08	53	2	809.96
									LIDDYDSTK	4	N-Term(iTRAQ4plex); K9(iTRAQ4plex	0.963	3.89	64	2	679.36
									ETLITIMK	2	N-Term(iTRAQ4plex); K8(iTRAQ4plex		3.76	48	2	618.87
									TLIDFVK	11	N-Term(iTRAQ4plex); K7(iTRAQ4plex		3.24	54	2	562.35
									LFPAPSEK	1	N-Term(iTRAQ4plex); K8(iTRAQ4plex			40		
205277441	SERPINA7	thyroxine-binding globulin precurso	34.94	11	11	92	1.176	46.3								
									EGQMESVEAAMSSK	8	N-Term(iTRAQ4plex); K14(iTRAQ4plex	1.141	7.04	131	2	886.42
									GTEAAAVPEVELSDQPENTFLHPIIQIDR	3	N-Term(iTRAQ4plex	1.313	5.8	84	3	1111.91
									TEDSSSFLIDK	22	N-Term(iTRAQ4plex); K11(iTRAQ4plex	1.234	5.76	92	2	765.40
									MGIQHAYSENADFSGLTEDNGLK	2	N-Term(iTRAQ4plex); K23(iTRAQ4plex	1.106	5.36	85	3	929.11
									EGQMESVEAAMSSK	2	N-Term(iTRAQ4plex); M4(Oxidation); K14(iTRAQ4plex)	1.176	5.33	105	2	894.42
									NALALFVLPK	11	N-Term(iTRAQ4plex); K10(iTRAQ4plex	1.162	4.45	69	2	687.44
									MSSINADFAFNLYR	3	N-Term(iTRAQ4plex	1.071	4.4	74	2	896.94
									AQWANPFDPSK	8	N-Term(iTRAQ4plex); K11(iTRAQ4plex	1.332	4.36	66	2	774.90
									FTVETPDK	8	N-Term(iTRAQ4plex); K8(iTRAQ4plex	0.872	3.46	53	2	612.83
									GWVDLFVPK	11	N-Term(iTRAQ4plex); K9(iTRAQ4plex	1.693	3.25	62	2	674.90
									MGIQHAYSENADFSGLTEDNGLK	1	N-Term(iTRAQ4plex); M1(Oxidation); K23(iTRAQ4plex)	0.725		67		
									SILFLGK	12	N-Term(iTRAQ4plex); K7(iTRAQ4plex	1.124		48		
									SFMLLILER	1	N-Term(iTRAQ4plex	1.176		38		
4504067	GOT1	aspartate aminotransferase, cytoplasmic	34.87	11	11	40	0.662	46.2								
									NLDYVATSIHEAVTK	11	N-Term(iTRAQ4plex); K15(iTRAQ4plex	0.564	6.48	74	3	650.36
									VGGVQSLGGTGALR	4	N-Term(iTRAQ4plex	0.648	5.03	84	2	708.41
									QVEYLVNEK	4	N-Term(iTRAQ4plex); K9(iTRAQ4plex	0.605	4.48	57	2	705.39
									TDDCHPWVLPVVK	2	N-Term(iTRAQ4plex); C4(Methylthio); K13(iTRAQ4plex)	0.753	4.44	45	2	921.98
									LALGDDSPALK	2	N-Term(iTRAQ4plex); K11(iTRAQ4plex	0.834	3.97	50	2	694.41
									ITWSNPPAQGAR	4	N-Term(iTRAQ4plex	0.713	3.85	56	2	721.39
									IANDNSLNHEYLPILGLAEFR	2	N-Term(iTRAQ4plex	0.463	3.84	54	3	848.45
									INVSGLTTK	3	N-Term(iTRAQ4plex); K9(iTRAQ4plex	0.711	3.51	44	2	610.88
									VNLGVGAYR	5	N-Term(iTRAQ4plex	0.722	3.45	47	2	546.82
									TPGTWNHITDQIGMFSFTGLNPK	1	N-Term(iTRAQ4plex); K23(iTRAQ4plex	0.662		60		
									IGADFLAR	2	N-Term(iTRAQ4plex	0.61		40		
16306550	SELENBP1	selenium-binding protein 1	34.53	13	13	50	0.692	52.4								
									EPLGPALAHELR	17	N-Term(iTRAQ4plex	0.691	6.35	74	3	482.94
									VAGGPQMIQLSLDGK	2	N-Term(iTRAQ4plex); K15(iTRAQ4plex	0.683	6.19	87	2	901.51
									HEIVQTLSLK	4	N-Term(iTRAQ4plex); K10(iTRAQ4plex	0.741	4.54	54	2	728.44
									GGPVQVLEDEELK	2	N-Term(iTRAQ4plex); K13(iTRAQ4plex	0.476	4.38	68	2	850.98
									SPQYCQVIHR	8	N-Term(iTRAQ4plex); C5(Methylthio	0.69	4.36	68	3	474.24
									EGSVMLQVDVDTVK	2	N-Term(iTRAQ4plex); K14(iTRAQ4plex	0.839	4.09	92	2	904.50
									IYVVDVGSEPR	5	N-Term(iTRAQ4plex	0.713	3.95	52	2	689.38
									LNPNFLVDFGK	2	N-Term(iTRAQ4plex); K11(iTRAQ4plex	0.804	3.93	55	2	776.44
									LTGQLFLGGSIVK	2	N-Term(iTRAQ4plex); K13(iTRAQ4plex	0.723	3.45	77	2	811.00
									EEIVYLPCIYR	2	N-Term(iTRAQ4plex); C8(Methylthio	0.855	3.17	42	2	794.41
									LVLPSLISSR	2	N-Term(iTRAQ4plex	0.69		44		
									DGFNPADVEAGLYGSHLYVWDWQR	1	N-Term(iTRAQ4plex	0.585		40		
									DGLIPLEIR	1	N-Term(iTRAQ4plex	0.66		40		
157653329	PCOLCE	procollagen C-endopeptidase enhancer 1	34.08	11	11	79	0.929	47.9								

									TEESPSAPDAPTCPK	6	N-Term(iTRAQ4plex); C13(Methylthio); K15(iTRAQ4plex)	1.028	6.35	88	2	932.44
									YDSVSVFNGAVSDDSF	5	N-Term(iTRAQ4plex	1.07	6.32	112	2	931.44
									GVSYLLMGQVEENR	14	N-Term(iTRAQ4plex	0.864	5.89	82	2	869.95
									YDALEVFAGSGTSGQF	15	N-Term(iTRAQ4plex	0.9	5.31	85	2	901.44
									TGGLDLPSPPTGASLK	14	N-Term(iTRAQ4plex); K16(iTRAQ4plex	0.96	5.26	102	2	900.01
									VFDLELHPACR	10	N-Term(iTRAQ4plex); C10(Methylthio	0.912	4.68	54	2	745.37
									GESGYVASEGFPNLYPPNK	4	N-Term(iTRAQ4plex); K19(iTRAQ4plex	0.953	4.27	65	2	1157.58
									KGVSYLLMGQVEENR	2	N-Term(iTRAQ4plex); K1(iTRAQ4plex	0.93	3.93	43	3	671.03
									GVSYLLMGQVEENR	1	N-Term(iTRAQ4plex); M7(Oxidation	0.932		65		
									GFLLWYSGR	6	N-Term(iTRAQ4plex	0.941		55		
									GPVLPPESFVVLHRPNQDQILTNLSK	1	N-Term(iTRAQ4plex); K26(iTRAQ4plex	1.483		52		
221316614	ECM1	extracellular matrix protein 1 isoform 1 precursor	33.89	5	14	58	1	60.6								
									EVGPPLPQEAVPLQK	8	N-Term(iTRAQ4plex); K15(iTRAQ4plex		5.93	60	2	945.55
									QGETLNFLEIGYSR	3	N-Term(iTRAQ4plex		5.8	75	2	885.96
									ELPSLQHPNEQK	2	N-Term(iTRAQ4plex); K12(iTRAQ4plex		4.9	39	3	569.98
									FCEAEFSVK	2	N-Term(iTRAQ4plex); C2(Methylthio); K9(iTRAQ4plex)	1.083	4.54	59	2	697.34
									ELLALIQLER	8	N-Term(iTRAQ4plex	1.002	4.12	48	2	671.42
									VTPNLMGHLCGNQR	2	N-Term(iTRAQ4plex); C10(Methylthio		3.8	42	3	577.28
									LLPAQLPAEK	11	N-Term(iTRAQ4plex); K10(iTRAQ4plex		3.79	49	2	684.43
									LVWEEAMSR	10	N-Term(iTRAQ4plex	1.022	3.79	44	2	632.83
									NVALVSGDTENAK	2	N-Term(iTRAQ4plex); K13(iTRAQ4plex		3.54	75	2	803.44
									DILTIDIGR	5	N-Term(iTRAQ4plex		3.45	47	2	580.35
									NLPATDPLQR	2	N-Term(iTRAQ4plex	0.907	3.27	40	2	634.86
									QHVVYGPWNLPQSSYSHLTR	1	N-Term(iTRAQ4plex			48		
									QLRPEHFQEVGYAAPPSPPLSR	1	N-Term(iTRAQ4plex			42		
55743122	RBP4	retinol-binding protein 4 precursor	33.83	6	6	41	1.169	23								
									YWGVASFLQK	18	N-Term(iTRAQ4plex); K10(iTRAQ4plex	1.295	4.59	75	2	743.92
									FSGTWYAMAK	14	N-Term(iTRAQ4plex); K10(iTRAQ4plex	0.959	4.51	72	2	725.37
									LIVHNGYCDGR	3	N-Term(iTRAQ4plex); C8(Methylthio	1.112	4.19	34	3	479.57
									GNDDHWIVDTDYDTYAVQYSCR	2	N-Term(iTRAQ4plex); C21(Methylthio	1.087	4.18	83	3	942.74
									QEELCLAR	3	N-Term(iTRAQ4plex); C5(Methylthio	0.945	3.63	40	2	576.29
									VKENFDK	1	N-Term(iTRAQ4plex); K2(iTRAQ4plex); K7(iTRAQ4plex)	1.681		37		
4504165	GSN	gelsolin isoform a precursor	33.76	0	21	167		85.6								
									AGALNSNDAFVLK	22	N-Term(iTRAQ4plex); K13(iTRAQ4plex		7	90	2	804.45
									DPDQTDGLGLSYLSSHIANVER	18	N-Term(iTRAQ4plex		6.86	116	3	844.42
									DSQEEEKTEALTSAK	8	N-Term(iTRAQ4plex); K7(iTRAQ4plex); K15(iTRAQ4plex)		6.62	85	2	1049.55
									VPFDAATLHTSTAMAAQHGMDDDGTGQK	3	N-Term(iTRAQ4plex); K28(iTRAQ4plex		6.16	34	4	791.13
									TPSAAYLWVGTGASEAEK	12	N-Term(iTRAQ4plex); K18(iTRAQ4plex		6.05	107	2	1063.56
									EVQGFESATFLGYFK	16	N-Term(iTRAQ4plex); K15(iTRAQ4plex		6	93	2	1006.03
									QTQVSVLPEGGETPLFK	10	N-Term(iTRAQ4plex); K17(iTRAQ4plex		5.4	68	2	1059.59
									SEDCFILDHGK	39	N-Term(iTRAQ4plex); C4(Methylthio); K11(iTRAQ4plex)		5.34	83	2	799.38
									VSNGAGTMSVSLVADENPFAQGALK	2	N-Term(iTRAQ4plex); K25(iTRAQ4plex		5.03	59	3	917.81
									AQPVQVAEGSEPDGFWEALGGK	8	N-Term(iTRAQ4plex); K22(iTRAQ4plex		4.69	88	2	1280.65
									TASDFITK	4	N-Term(iTRAQ4plex); K8(iTRAQ4plex		3.73	48	2	585.83
									AVEVLPK	4	N-Term(iTRAQ4plex); K7(iTRAQ4plex		3.15	45	2	522.34
									NWRDPDQTDGLGLSYLSSHIANVER	1	N-Term(iTRAQ4plex			60		
									HVVPNEVVVQR	1	N-Term(iTRAQ4plex			46		
									AMAELAA	1	N-Term(iTRAQ4plex			46		
									LFACSNK	1	N-Term(iTRAQ4plex); C4(Methylthio); K7(iTRAQ4plex)			39		
									TGAQELLR	1	N-Term(iTRAQ4plex			39		
									EPGLQIWR	12	N-Term(iTRAQ4plex			38		
									TPITVVK	2	N-Term(iTRAQ4plex); K7(iTRAQ4plex			38		
									AGKEPGLQIWR	1	N-Term(iTRAQ4plex); K3(iTRAQ4plex			37		
									YIETDPANR	1	N-Term(iTRAQ4plex			34		
239758130	LOC100293534	PREDICTED: complement C4-B-like, partia	33.63	0	14	174		73.8								
									YVSHFETEGPHVLLYFDSVPTSR	15	N-Term(iTRAQ4plex		7.3	71	3	942.14
									STQDTVIALDALSAYWIASHTTEER	13	N-Term(iTRAQ4plex		7.07	112	3	974.83
									GLEEELQFSLGSK	12	N-Term(iTRAQ4plex); K13(iTRAQ4plex		6.56	93	2	862.97
									VGLSGMAIADVTLLSGFHALR	4	N-Term(iTRAQ4plex		5.38	71	3	758.09
									DDPDAPLQPVTPLQLFEGR	7	N-Term(iTRAQ4plex		5.3	73	2	1126.59
									AEMADQAAAWLTR	8	N-Term(iTRAQ4plex); M3(Oxidation		5.14	80	2	797.39
									AEMADQAAAWLTR	2	N-Term(iTRAQ4plex		5.12	80	2	789.40
									LQETSNWLLSQQQADGSFQDLSPVIHR	8	N-Term(iTRAQ4plex		4.79	93	3	1081.22
									ITQVLHFTK	18	N-Term(iTRAQ4plex); K9(iTRAQ4plex		4.23	77	2	687.92
									VEYGFQVK	30	N-Term(iTRAQ4plex); K8(iTRAQ4plex		4.16	51	2	629.36
									CSVFYGAPSK	29	N-Term(iTRAQ4plex); C1(Methylthio); K10(iTRAQ4plex)		4.12	70	2	696.85
									TYNVLDMK	21	N-Term(iTRAQ4plex); K8(iTRAQ4plex		4.09	46	2	636.35
									VHYTVCIWR	4	N-Term(iTRAQ4plex); C6(Methylthio		3.48	36	3	456.24
									ANSFLGEK	2	N-Term(iTRAQ4plex); K8(iTRAQ4plex			47		
									ECVGFEAVQEVPVGLVQPASATLYDYYNPER	1	N-Term(iTRAQ4plex); C2(Methylthio			31		
4504345	HBA2	hemoglobin subunit alpha	33.1	3	4	8	0.388	15.2								
									VGAHAGEYGAEALER	4	N-Term(iTRAQ4plex	0.509	5.5	76	2	837.43
									TYFPHFDLSHGSAQVK	2	N-Term(iTRAQ4plex); K16(iTRAQ4plex	0.365	4.94	48	3	708.04
									MFLSFPTTK	1	N-Term(iTRAQ4plex); K9(iTRAQ4plex	0.297		65		
									VDPVNFK	1	N-Term(iTRAQ4plex); K7(iTRAQ4plex			55		
32189392	PRDX2	peroxiredoxin-2 isoform a	32.83	4	7	15	0.864	21.9								
									ATAVVDGAFK	3	N-Term(iTRAQ4plex); K10(iTRAQ4plex		4.35	67	2	633.87
									QITVNDLPVGR	2	N-Term(iTRAQ4plex		4.31	52	2	678.39
									GLFIIDGK	4	N-Term(iTRAQ4plex); K8(iTRAQ4plex	0.78	3.3	47	2	575.86
									EGGLGPLNIPLLADVTR	2	N-Term(iTRAQ4plex	0.538	3.15	40	2	940.05
									KEGGLGPLNIPLLADVTR	2	N-Term(iTRAQ4plex); K1(iTRAQ4plex	1.166		44		
208973246	QDPR	dihydropteridine reductase	32.79	6	6	14	1.002	25.8								
									QSIWTSTISSHLATK	2	N-Term(iTRAQ4plex); K15(iTRAQ4plex	0.916	7.28	76	3	650.03
									MTDSFTEQADQVTAEVGK	2	N-Term(iTRAQ4plex); K18(iTRAQ4plex	0.932	7.26	101	2	1123.05
									AALDGTPGMIGYGMAK	2	N-Term(iTRAQ4plex); K16(iTRAQ4plex	0.984	6.16	115	2	920.99
									EGGLLTLAGAK	2	N-Term(iTRAQ4plex); K11(iTRAQ4plex	1.037	5.27	63	2	659.40
									GAVHQLCQSLAGK	4	N-Term(iTRAQ4plex); C7(Methylthio); K13(iTRAQ4plex)	1.041	5.08	90	2	823.44

									NCDLMWK	2	N-Term(iTRAQ4plex); C2(Methylthio); K7(iTRAQ4plex)	0.955		44		
4507645	TPI1	triosephosphate isomerase isoform 1	32.53	0	7	24		26.7								
									DCGATWVVLGHSER	6	N-Term(iTRAQ4plex); C2(Methylthio		5.75	88	3	573.94
									IIYGGSVTGATCK	2	N-Term(iTRAQ4plex); C12(Methylthio); K13(iTRAQ4plex)		5.38	82	2	802.43
									KFFVGGNWK	2	N-Term(iTRAQ4plex); K1(iTRAQ4plex); K9(iTRAQ4plex)		5.17	40	3	505.64
									VVLAYEPVWAIGTGK	3	N-Term(iTRAQ4plex); K15(iTRAQ4plex		4.26	62	2	946.05
									VPADTEVVCAPPTAYIDFAR	2	N-Term(iTRAQ4plex); C9(Methylthio		4.07	55	2	1163.07
									IAVAAQNCYK	6	N-Term(iTRAQ4plex); C8(Methylthio); K10(iTRAQ4plex)		3.78	62	2	707.87
									FFVGGNWK	3	N-Term(iTRAQ4plex); K8(iTRAQ4plex		3.19	47	2	621.85
4557871	TF	serotransferrin precursor	32.38	19	19	71	0.447	77								
									EDPQTFYYAVAVVK	3	N-Term(iTRAQ4plex); K14(iTRAQ4plex	0.544	6.05	89	2	959.52
									EFQLFSSPHGK	5	N-Term(iTRAQ4plex); K11(iTRAQ4plex	0.521	5.57	75	3	522.28
									SASDLTWDNLK	8	N-Term(iTRAQ4plex); K11(iTRAQ4plex	0.475	5.05	89	2	769.41
									KDSGFQMNQLR	4	N-Term(iTRAQ4plex); K1(iTRAQ4plex	0.719	4.84	44	3	537.96
									WCAVSEHEATK	3	N-Term(iTRAQ4plex); C2(Methylthio); K11(iTRAQ4plex)	0.578	4.84	65	2	797.89
									SVIPSDGPSVACVK	7	N-Term(iTRAQ4plex); C12(Methylthio); K14(iTRAQ4plex)	0.376	4.78	70	2	846.95
									MYLGYEYVTAIR	6	N-Term(iTRAQ4plex	0.383	4.69	53	2	811.92
									EDLIWELLNQAQEHFGK	5	N-Term(iTRAQ4plex); K17(iTRAQ4plex	0.294	4.64	72	3	786.75
									FDEFFSEGCAPGSK	4	N-Term(iTRAQ4plex); C9(Methylthio); K14(iTRAQ4plex)	0.485	4.31	69	2	927.93
									EGYYGYTGAFR	4	N-Term(iTRAQ4plex	0.556	4.28	51	2	714.34
									ADRDQYELLCLDNTR	3	N-Term(iTRAQ4plex); C10(Methylthio	0.584	4.26	39	3	672.32
									DGAGDVAFVK	4	N-Term(iTRAQ4plex); K10(iTRAQ4plex	0.399	3.94	64	2	633.85
									DCHLAQVPSHTVVAR	3	N-Term(iTRAQ4plex); C2(Methylthio	0.616	3.78	65	4	456.48
									WCALSHHER	2	N-Term(iTRAQ4plex); C2(Methylthio	0.406	3.52	46	3	443.54
									HSTIFENLANK	2	N-Term(iTRAQ4plex); K11(iTRAQ4plex	0.314	3.51	63	3	521.29
									DYELLCLDGTR	2	N-Term(iTRAQ4plex); C6(Methylthio	0.455	3.21	36	2	744.35
									CSTSSLLEACTFR	2	N-Term(iTRAQ4plex); C1(Methylthio); C10(Methylthio)	0.326		57		
									YLGEEYVK	1	N-Term(iTRAQ4plex); K8(iTRAQ4plex	0.326		47		
									ASYLDCIR	3	N-Term(iTRAQ4plex); C6(Methylthio	0.359		39		
50592994	TXN	thioredoxin	31.43	3	3	4	1.1	11.7								
									TAFQEALDAAGDK	2	N-Term(iTRAQ4plex); K13(iTRAQ4plex	1.1	4.31	107	2	812.92
									CMPTFQFFK	1	N-Term(iTRAQ4plex); C1(Methylthio); K9(iTRAQ4plex)	1.137		59		
									EKLEATINELV	1	N-Term(iTRAQ4plex); K2(iTRAQ4plex	1.1		46		
169218213	LOC100133511	PREDICTED: complement C3-like, partia	31.35	0	33	98		144.7								
									AGDFLEANYMNLQR	4	N-Term(iTRAQ4plex		5.88	56	2	893.44
									EVVADSVWVDVK	2	N-Term(iTRAQ4plex); K12(iTRAQ4plex		5.78	78	2	817.46
									SNLDEDIIAEENIVSR	6	N-Term(iTRAQ4plex		5.5	105	2	981.00
									VELLHNPAFCSLATTK	4	N-Term(iTRAQ4plex); C10(Methylthio); K16(iTRAQ4plex)		5.13	108	3	693.37
									KGYTQQLAFR	2	N-Term(iTRAQ4plex); K1(iTRAQ4plex		5.08	44	3	500.62
									DSCVGSLVVK	2	N-Term(iTRAQ4plex); C3(Methylthio); K10(iTRAQ4plex)		4.95	65	2	670.86
									TVMVNIENPEGIPVK	2	N-Term(iTRAQ4plex); K15(iTRAQ4plex		4.94	61	2	964.54
									IPIEDGSGEVVLSR	4	N-Term(iTRAQ4plex		4.88	73	2	807.95
									ILLQGTPVAQMTEDAVDAEF	3	N-Term(iTRAQ4plex		4.84	75	2	1151.10
									QPSSAFAAFVK	2	N-Term(iTRAQ4plex); K11(iTRAQ4plex		4.69	79	2	720.91
									GYTQQLAFR	4	N-Term(iTRAQ4plex		4.48	44	2	614.33
									EDIPPADLSDQVPDTESETR	10	N-Term(iTRAQ4plex		4.24	83	3	786.71
									TIYTPGSTVLYR	6	N-Term(iTRAQ4plex		4.23	58	2	757.92
									QPVPGQQMTLK	3	N-Term(iTRAQ4plex); K11(iTRAQ4plex		4.23	59	2	757.93
									SSLSVPYVIVPLK	2	N-Term(iTRAQ4plex); K13(iTRAQ4plex		4.15	51	2	845.53
									APSTWLTAYVVK	3	N-Term(iTRAQ4plex); K12(iTRAQ4plex		4.1	93	2	812.47
									DFDFVPPVVR	4	N-Term(iTRAQ4plex		3.74	45	2	667.87
									QGALELIK	3	N-Term(iTRAQ4plex); K8(iTRAQ4plex		3.56	39	2	580.37
									FYYIYNEK	5	N-Term(iTRAQ4plex); K8(iTRAQ4plex		3.47	43	2	714.38
									FISLGEACK	3	N-Term(iTRAQ4plex); C8(Methylthio); K9(iTRAQ4plex)		3.45	66	2	651.35
									EYVLPSFEVIVEPTEK	2	N-Term(iTRAQ4plex); K16(iTRAQ4plex		3.43	35	2	1084.09
									ISLPESLK	4	N-Term(iTRAQ4plex); K8(iTRAQ4plex		3.33	49	2	587.87
									LMNIFLK	4	N-Term(iTRAQ4plex); K7(iTRAQ4plex		3.31	41	2	583.87
									YYTYLIMNK	1	N-Term(iTRAQ4plex); K9(iTRAQ4plex			74		
									GLEVTITAR	3	N-Term(iTRAQ4plex			65		
									LVAYYTLIGASGQR	1	N-Term(iTRAQ4plex			63		
									QKPDGVFQEDAPVIHQEMIGGLF	1	N-Term(iTRAQ4plex); K2(iTRAQ4plex			57		
									AYYENSPQQVFSTEFEVK	1	N-Term(iTRAQ4plex); K18(iTRAQ4plex			46		
									SYTVAIAGYALAQMGF	1	N-Term(iTRAQ4plex			42		
									WLILEK	2	N-Term(iTRAQ4plex); K6(iTRAQ4plex			39		
									DYAGVFSDAGLTFTSSSGQQTAQF	1	N-Term(iTRAQ4plex			34		
4504349	HBB	hemoglobin subunit beta	31.29	2	4	25	0.359	16								
									VVAGVANALAHK	6	N-Term(iTRAQ4plex); K12(iTRAQ4plex		5.99	78	3	479.97
									EFTPPVQAAYQK	8	N-Term(iTRAQ4plex); K12(iTRAQ4plex	0.339	4.94	73	2	833.96
									VNVDEVGGEALGR	6	N-Term(iTRAQ4plex	0.428	4.53	73	2	729.89
									LHVDPENFR	5	N-Term(iTRAQ4plex		3.47	38	3	424.23
221316616	ECM1	extracellular matrix protein 1 isoform 2 precursor	31.08	0	9	35		46.1								
									EVGPPLPQEAVPLQK	8	N-Term(iTRAQ4plex); K15(iTRAQ4plex		5.93	60	2	945.55
									QGETLNFLEIGYSR	3	N-Term(iTRAQ4plex		5.8	75	2	885.96
									ELPSLQHPNEQK	2	N-Term(iTRAQ4plex); K12(iTRAQ4plex		4.9	39	3	569.98
									VTPNLMGHLCGNQR	2	N-Term(iTRAQ4plex); C10(Methylthio		3.8	42	3	577.28
									LLPAQLPAEK	11	N-Term(iTRAQ4plex); K10(iTRAQ4plex		3.79	49	2	684.43
									NVALVSGDTENAK	2	N-Term(iTRAQ4plex); K13(iTRAQ4plex		3.54	75	2	803.44
									DILTIDIGR	5	N-Term(iTRAQ4plex		3.45	47	2	580.35
									QHVVYGPWNLPQSSYSHLTF	1	N-Term(iTRAQ4plex			48		
									QLRPEHFQEVGYAAPPSPPLSF	1	N-Term(iTRAQ4plex			42		
21071030	A1BG	alpha-1B-glycoprotein precursor	30.91	11	11	228	1.035	54.2								
									TPGAAANLELIFVGPQHAGNYF	27	N-Term(iTRAQ4plex	1.041	8.37	80	3	814.10
									NGVAQEPVHLDSPAIK	24	N-Term(iTRAQ4plex); K16(iTRAQ4plex	1.169	6.82	103	2	982.05
									SLPAPWLSMAPVSWITPGLK	5	N-Term(iTRAQ4plex); K20(iTRAQ4plex	1.248	6.2	59	2	1220.19
									SGLSTGWTQLSK	27	N-Term(iTRAQ4plex); K12(iTRAQ4plex	0.991	6.01	76	2	776.93

									IFFHLNAVALGDGGHYTCF	4	N-Term(iTRAQ4plex); C18(Methylthio	1.022	4.97	78	3	761.04
									CEGPIPDVTFELLR	20	N-Term(iTRAQ4plex); C1(Methylthio	1.006	4.89	71	2	889.95
									ATWSGAVLAGR	48	N-Term(iTRAQ4plex	1.066	4.85	73	2	616.84
									LELHVDGPPPRPQLR	18	N-Term(iTRAQ4plex	1.026	4.15	61	3	623.36
									CLAPLEGAR	5	N-Term(iTRAQ4plex); C1(Methylthio	1.097	3.4	49	2	560.29
									LLELTGPK	26	N-Term(iTRAQ4plex); K8(iTRAQ4plex	0.978	3.33	42	2	579.86
									GVTFLLR	23	N-Term(iTRAQ4plex	1.03	3.28	46	2	475.30
									SLPAPWLSMAPVSWITPGLK	1	N-Term(iTRAQ4plex); M9(Oxidation); K20(iTRAQ4plex)	1.295		34		
42544239	CFD	complement factor D preproprotein	30.83	4	4	12	0.937	27								
									VQVLLGAHSLSQPEPSK	4	N-Term(iTRAQ4plex); K17(iTRAQ4plex	1.178	6.1	90	3	693.40
									RPDSLQHVLLPVLDR	6	N-Term(iTRAQ4plex	0.937	3.9	45	3	634.71
									AVPHPDSQPDTIDHDLLLLQLSEK	1	N-Term(iTRAQ4plex); K24(iTRAQ4plex	0.843		46		
									GDSGGPLVCGGVLEGVVTSGSF	1	N-Term(iTRAQ4plex); C9(Methylthio	0.733		45		
4885063	ALDOC	fructose-bisphosphate aldolase C	30.77	7	8	47	0.815	39.4								
									DNAGAATEEFIK	6	N-Term(iTRAQ4plex); K12(iTRAQ4plex	0.901	6.48	79	2	777.41
									GVVPLAGTDGETTTQGLDGLSEF	11	N-Term(iTRAQ4plex	0.733	5.98	100	3	806.42
									YTPEEIAMATVTALF	8	N-Term(iTRAQ4plex	0.76	5.38	77	2	905.48
									TPSALAILENANVLAF	2	N-Term(iTRAQ4plex	0.711	5.29	91	2	899.02
									LSQIGVENTEENRF	3	N-Term(iTRAQ4plex	0.715	4.81	51	2	894.97
									GILAADESVGSMAK	10	N-Term(iTRAQ4plex); K14(iTRAQ4plex	0.866	4.79	99	2	818.94
									ALQASALNAWR	5	N-Term(iTRAQ4plex	0.827	3.92	43	2	672.88
									VLAAVYK	2	N-Term(iTRAQ4plex); K7(iTRAQ4plex		3.15	41	2	526.34
4504893	KNG1	kininogen-1 isoform 2	30.68	0	13	125		47.9								
									DIPTNSPELEETLTHTITK	12	N-Term(iTRAQ4plex); K19(iTRAQ4plex		7.73	108	3	809.77
									YNSQNQSNNQFVLYF	9	N-Term(iTRAQ4plex		6.17	84	2	1009.99
									ESNEELTESCETK	2	N-Term(iTRAQ4plex); C10(Methylthio); K13(iTRAQ4plex)		5.95	82	2	916.91
									TVGSDTFYSFK	28	N-Term(iTRAQ4plex); K11(iTRAQ4plex		5.6	74	2	770.39
									AATGECTATVGK	12	N-Term(iTRAQ4plex); C6(Methylthio); K12(iTRAQ4plex)		5.53	88	2	721.87
									RPPGFSPFR	15	N-Term(iTRAQ4plex		4.84	74	3	402.23
									KYNSQNQSNNQFVLYF	3	N-Term(iTRAQ4plex); K1(iTRAQ4plex		4.72	78	3	764.40
									KYFIDFVAR	16	N-Term(iTRAQ4plex); K1(iTRAQ4plex		4.47	66	3	482.95
									TWQDCEYK	7	N-Term(iTRAQ4plex); C5(Methylthio); K8(iTRAQ4plex)		3.76	41	2	703.82
									ENFLFLTPDCK	5	N-Term(iTRAQ4plex); C10(Methylthio); K11(iTRAQ4plex)		3.74	63	2	830.92
									QVVAGLNFR	9	N-Term(iTRAQ4plex		3.66	44	2	574.34
									IASFSQNCDIYPGK	3	N-Term(iTRAQ4plex); C8(Methylthio); K14(iTRAQ4plex)		3.45	71	2	938.96
									YFIDFVAR	4	N-Term(iTRAQ4plex			37		
118582275	SOD3	extracellular superoxide dismutase [Cu-Zn] precursor	30.42	6	6	98	0.873	25.8								
									AGLAASLAGPHSIVGF	38	N-Term(iTRAQ4plex	0.991	8.37	115	3	540.98
									RDDDGALHAACQVQPSATLDAAQPF	2	N-Term(iTRAQ4plex); C11(Methylthio	0.895	5.27	113	3	932.78
									AVVVHAGEDDLGF	16	N-Term(iTRAQ4plex	0.678	4.94	77	2	741.40
									VTEIWQEVMQR	23	N-Term(iTRAQ4plex	0.948	4.67	65	2	781.91
									DDDGALHAACQVQPSATLDAAQPF	3	N-Term(iTRAQ4plex); C10(Methylthio	1.353	3.44	71	3	880.75
									VTGVVLFR	16	N-Term(iTRAQ4plex	0.64	3.22	43	2	517.83
5803023	LMAN2	vesicular integral-membrane protein VIP36 precursor	30.34	8	8	46	0.935	40.2								
									DNFHGLAIFLDTYPNDETTEF	4	N-Term(iTRAQ4plex	0.889	6.24	74	3	871.42
									LTVMTDLEDKNEWK	3	N-Term(iTRAQ4plex); K10(iTRAQ4plex); K14(iTRAQ4plex)	0.997	5.45	45	2	1077.58
									LFQLMVEHTPDEESIDWTK	2	N-Term(iTRAQ4plex); K19(iTRAQ4plex	0.76	4.92	90	3	869.44
									NLHGDGIALWYTR	7	N-Term(iTRAQ4plex	0.938	4.76	58	3	553.96
									DWEMHVHFK	15	N-Term(iTRAQ4plex); K9(iTRAQ4plex	0.984	4.38	52	4	379.95
									EGSIWNHQPCFLK	8	N-Term(iTRAQ4plex); C10(Methylthio); K13(iTRAQ4plex)	0.87	4.34	43	3	631.65
									LFQLMVEHTPDEESIDWTK	2	N-Term(iTRAQ4plex); M5(Oxidation); K19(iTRAQ4plex)	0.927	3.81	67	3	874.78
									LVPGPVFGSK	4	N-Term(iTRAQ4plex); K10(iTRAQ4plex	0.83	3.44	49	2	644.90
									IEPSVNFLK	1	N-Term(iTRAQ4plex); K9(iTRAQ4plex	1.184		55		
192447430	CA1	carbonic anhydrase 1	30.27	5	5	12	0.497	28.9								
									ESISVSSEQLAQFR	4	N-Term(iTRAQ4plex	0.559	5.1	90	2	862.95
									VLDALQAIK	4	N-Term(iTRAQ4plex); K9(iTRAQ4plex	0.523	4.62	68	2	629.91
									SLLSNVEGDNAVPMQHNNRPTQPLI	2	N-Term(iTRAQ4plex); K25(iTRAQ4plex	0.497	3.41	58	4	762.65
									ADGLAVIGVLMK	1	N-Term(iTRAQ4plex); K12(iTRAQ4plex	0.274		49		
									EIINVGHSFHVNFEDNDNF	1	N-Term(iTRAQ4plex	0.462		38		
73858566	SERPIND1	heparin cofactor 2 precursor	30.26	13	13	88	0.912	57								
									GGETAQSADPQWEQLNNK	3	N-Term(iTRAQ4plex); K18(iTRAQ4plex	0.978	6.51	78	2	1131.06
									GETHEQVHSILHFK	8	N-Term(iTRAQ4plex); K14(iTRAQ4plex	0.839	6.18	87	4	488.27
									YEITTIHNLFR	15	N-Term(iTRAQ4plex	0.803	4.72	55	3	517.62
									NYNLVESLK	14	N-Term(iTRAQ4plex); K9(iTRAQ4plex	1.003	4.47	63	2	684.39
									QFPILLDFK	11	N-Term(iTRAQ4plex); K9(iTRAQ4plex	0.884	3.87	64	2	704.93
									TSCLLFMGR	5	N-Term(iTRAQ4plex); C3(Methylthio	0.815	3.58	51	2	609.30
									IAIDLFK	22	N-Term(iTRAQ4plex); K7(iTRAQ4plex	1.022	3.28	55	2	554.36
									SVNDLYIQK	2	N-Term(iTRAQ4plex); K9(iTRAQ4plex	0.958	3.2	64	2	684.38
									TLEAQLTPR	2	N-Term(iTRAQ4plex	0.738		47		
									HQGTITVNEEGTQATTVTTVGFMPLSTQ R	1	N-Term(iTRAQ4plex			46		
									FPVEMTHNHNFF	1	N-Term(iTRAQ4plex	0.833		43		
									LNILNAK	3	N-Term(iTRAQ4plex); K7(iTRAQ4plex	0.918		37		
115298678	C3	complement C3 precursor	30.13	8	41	112	0.781	187								
									AGDFLEANYMNLQF	4	N-Term(iTRAQ4plex		5.88	56	2	893.44
									EVVADSVWVDVK	2	N-Term(iTRAQ4plex); K12(iTRAQ4plex		5.78	78	2	817.46
									ENEGFTVTAEGK	3	N-Term(iTRAQ4plex); K12(iTRAQ4plex	0.741	5.71	79	2	785.41
									SNLDEDIIAEENIVSR	6	N-Term(iTRAQ4plex		5.5	105	2	981.00
									VELLHNPAFCSLATTK	4	N-Term(iTRAQ4plex); C10(Methylthio); K16(iTRAQ4plex)		5.13	108	3	693.37
									KGYTQQLAFR	2	N-Term(iTRAQ4plex); K1(iTRAQ4plex		5.08	44	3	500.62
									DSCVGSLVVK	2	N-Term(iTRAQ4plex); C3(Methylthio); K10(iTRAQ4plex)		4.95	65	2	670.86
									TVMVNIENPEGIPVK	2	N-Term(iTRAQ4plex); K15(iTRAQ4plex		4.94	61	2	964.54
									IPIEDGSGEVVLSR	4	N-Term(iTRAQ4plex		4.88	73	2	807.95
									VYAYYNLEESCTR	2	N-Term(iTRAQ4plex); C11(Methylthio	0.734	4.85	58	2	900.91
									ILLQGTPVAQMTEDAVDAEF	3	N-Term(iTRAQ4plex		4.84	75	2	1151.10

									QPSSAFAAFVK	2	N-Term(iTRAQ4plex); K11(iTRAQ4plex		4.69	79	2	720.91
									GYTQQLAFR	4	N-Term(iTRAQ4plex		4.48	44	2	614.33
									VSHSEDDCLAFK	2	N-Term(iTRAQ4plex); C8(Methylthio); K12(iTRAQ4plex)	0.809	4.33	51	3	562.27
									EDIPPADLSDQVPDTESETR	10	N-Term(iTRAQ4plex		4.24	83	3	786.71
									TIYTPGSTVLYR	6	N-Term(iTRAQ4plex		4.23	58	2	757.92
									QPVPGQQMTLK	3	N-Term(iTRAQ4plex); K11(iTRAQ4plex		4.23	59	2	757.93
									SSLSVPYVIVPLK	2	N-Term(iTRAQ4plex); K13(iTRAQ4plex		4.15	51	2	845.53
									APSTWLTAYVVK	3	N-Term(iTRAQ4plex); K12(iTRAQ4plex		4.1	93	2	812.47
									DFDFVPPVVR	4	N-Term(iTRAQ4plex		3.74	45	2	667.87
									CAEENCFIQK	2	N-Term(iTRAQ4plex); C1(Methylthio); C6(Methylthio); K10(iTRAQ4plex)	0.775	3.72	68	2	782.84
									QGALELIK	3	N-Term(iTRAQ4plex); K8(iTRAQ4plex		3.56	39	2	580.37
									FYYIYNEK	5	N-Term(iTRAQ4plex); K8(iTRAQ4plex		3.47	43	2	714.38
									FISLGEACK	3	N-Term(iTRAQ4plex); C8(Methylthio); K9(iTRAQ4plex)		3.45	66	2	651.35
									EYVLPSFEVIVEPTEK	2	N-Term(iTRAQ4plex); K16(iTRAQ4plex		3.43	35	2	1084.09
									ISLPESLK	4	N-Term(iTRAQ4plex); K8(iTRAQ4plex		3.33	49	2	587.87
									LMNIFLK	4	N-Term(iTRAQ4plex); K7(iTRAQ4plex		3.31	41	2	583.87
									VLLDGVQNPR	2	N-Term(iTRAQ4plex	0.791	3.13	52	2	627.87
									YYTYLIMNK	1	N-Term(iTRAQ4plex); K9(iTRAQ4plex			74		
									GLEVTITAR	3	N-Term(iTRAQ4plex			65		
									LVAYYTLIGASGQR	1	N-Term(iTRAQ4plex			63		
									QKPDGVFQEDAPVIHQEMIGGLR	1	N-Term(iTRAQ4plex); K2(iTRAQ4plex			57		
									NTLIIYLDK	1	N-Term(iTRAQ4plex); K9(iTRAQ4plex	0.755		56		
									AYYENSPQQVFSTEFEVK	1	N-Term(iTRAQ4plex); K18(iTRAQ4plex			46		
									IHWESASLLR	1	N-Term(iTRAQ4plex	0.787		46		
									SYTVAIAGYALAQMGR	1	N-Term(iTRAQ4plex			42		
									WLILEK	2	N-Term(iTRAQ4plex); K6(iTRAQ4plex			39		
									DAPDHQELNLDVSLQLPSR	1	N-Term(iTRAQ4plex	0.794		37		
									DYAGVFSDAGLTFTSSSGQQTAQR	1	N-Term(iTRAQ4plex			34		
5231228	RNASET2	ribonuclease T2 precursor	30.08	6	6	14	1.054	29.5								
									HGTCAAQVDALNSQK	2	N-Term(iTRAQ4plex); C4(Methylthio); K15(iTRAQ4plex)	1.141	6.35	87	2	938.97
									SWPFNLEEIK	6	N-Term(iTRAQ4plex); K10(iTRAQ4plex	0.967	4.4	66	2	775.93
									DPPDYWTIHGLWPDK	2	N-Term(iTRAQ4plex); K15(iTRAQ4plex	1.104	4.13	35	3	710.03
									VCEDGPVFYPPPK	2	N-Term(iTRAQ4plex); C2(Methylthio); K13(iTRAQ4plex)	1.054	3.85	42	2	891.45
									ELDLNSVLLK	1	N-Term(iTRAQ4plex); K10(iTRAQ4plex	0.968		58		
									LIMVQHWPETVCEK	1	N-Term(iTRAQ4plex); C12(Methylthio); K14(iTRAQ4plex)	1.313		40		
45580688	C7	complement component C7 precursor	30.01	19	19	125	1.032	93.5								
									LIDQYGTHYLQSGSLGGEYR	12	N-Term(iTRAQ4plex	1.005	6.97	100	3	801.07
									YSAWAESVTNLPQVIK	3	N-Term(iTRAQ4plex); K16(iTRAQ4plex	1.178	6.17	58	2	1047.58
									LSGNVLSYTFQVK	6	N-Term(iTRAQ4plex); K13(iTRAQ4plex	1.214	5.74	75	2	872.50
									WLVGEMHCQK	9	N-Term(iTRAQ4plex); C8(Methylthio); K10(iTRAQ4plex)	1.068	5.38	48	3	522.26
									ELSHLPSLYDYSAYR	28	N-Term(iTRAQ4plex	0.959	5.23	72	3	653.33
									EQTMSECEAGALR	3	N-Term(iTRAQ4plex); C7(Methylthio	1.043	5.08	48	2	807.86
									DGFVQDEGTMFPVGK	6	N-Term(iTRAQ4plex); K15(iTRAQ4plex	1.188	5.08	84	2	957.98
									DSCTLPASAEK	10	N-Term(iTRAQ4plex); C3(Methylthio); K11(iTRAQ4plex)	1.085	4.84	72	2	728.36
									GGGAGFISGLSYLELDNPAGNKR	2	N-Term(iTRAQ4plex); K22(iTRAQ4plex	1.447	4.82	44	3	861.13
									LTPLYELVK	14	N-Term(iTRAQ4plex); K9(iTRAQ4plex	0.999	4.32	76	2	682.43
									VLFYVDSEK	14	N-Term(iTRAQ4plex); K9(iTRAQ4plex	1.086	4.21	53	2	694.39
									CFSGQCISK	2	N-Term(iTRAQ4plex); C1(Methylthio); C6(Methylthio); K9(iTRAQ4plex)	1.295	4.21	47	2	676.80
									RPSCDIDKPPPNIELTGNGYNELTGQFR	2	N-Term(iTRAQ4plex); C4(Methylthio); K8(iTRAQ4plex)	1.253	3.59	59	4	867.19
									DGFVQDEGTMFPVGK	2	N-Term(iTRAQ4plex); M10(Oxidation); K15(iTRAQ4plex)	1.226	3.49	71	2	965.97
									ILPLTVCK	5	N-Term(iTRAQ4plex); C7(Methylthio); K8(iTRAQ4plex)	0.965		55		
									EVPCASVK	3	N-Term(iTRAQ4plex); C4(Methylthio); K8(iTRAQ4plex)	1.01		49		
									NVVYTCNEGYSLIGNPVAR	1	N-Term(iTRAQ4plex); C6(Methylthio	1.057		41		
									WSPEMK	1	N-Term(iTRAQ4plex); K6(iTRAQ4plex	1.752		38		
4557417	CD14	monocyte differentiation antigen CD14 precursor	29.87	8	8	71	0.92	40.1								
									VLSIAQAHSPAFSCEQVR	4	N-Term(iTRAQ4plex); C14(Methylthio	0.822	6.5	64	3	711.69
									LTVGAAQVPAQLLVGALR	16	N-Term(iTRAQ4plex	0.93	5.68	114	2	961.09
									ITGTMPPLPLEATGLALSSLR	11	N-Term(iTRAQ4plex	1.18	5.31	84	2	1141.65
									GLMAALCPHK	9	N-Term(iTRAQ4plex); C7(Methylthio); K10(iTRAQ4plex)	1.118	4.9	71	2	687.88
									ITGTMPPLPLEATGLALSSLR	4	N-Term(iTRAQ4plex); M5(Oxidation	1.078	4.31	78	2	1149.65
									ELTLEDLK	17	N-Term(iTRAQ4plex); K8(iTRAQ4plex	0.851	4.09	59	2	624.87
									GLMAALCPHK	2	N-Term(iTRAQ4plex); M3(Oxidation); C7(Methylthio); K10(iTRAQ4plex)	0.956	3.82	40	3	464.25
									FPAIQNLALR	5	N-Term(iTRAQ4plex	0.848	3.24	36	2	643.89
									AFPALTSLDLSDNPGLGER	2	N-Term(iTRAQ4plex	0.749		56		
									VLDLSCNR	1	N-Term(iTRAQ4plex); C6(Methylthio	0.9		34		
4503009	CPE	carboxypeptidase E preproprotein	29.83	11	11	96	0.98	53.1								
									LQQEDGISFEYHR	9	N-Term(iTRAQ4plex	0.929	6.14	78	3	589.29
									SGSAHEYSSSPDDAIFQSLAR	8	N-Term(iTRAQ4plex	0.805	6	93	3	790.38
									LTASAPGYLAITK	9	N-Term(iTRAQ4plex); K13(iTRAQ4plex	0.938	5.55	86	2	797.48
									IHIMPSLNPDGFEK	12	N-Term(iTRAQ4plex); K14(iTRAQ4plex	0.968	4.99	105	3	629.34
									IHIMPSLNPDGFEK	4	N-Term(iTRAQ4plex); M4(Oxidation); K14(iTRAQ4plex)	1.008	4.95	54	3	634.67
									NSLISYLEQIHR	21	N-Term(iTRAQ4plex	1.016	4.85	73	2	808.95
									AYSSFNPAMSDPNRPPCR	9	N-Term(iTRAQ4plex); C17(Methylthio	1.078	4.42	54	3	734.00
									FPPEETLK	15	N-Term(iTRAQ4plex); K8(iTRAQ4plex	0.98		57		
									LLIPGNYK	5	N-Term(iTRAQ4plex); K8(iTRAQ4plex	0.953		50		
									KVAVPYSPAAGVDFELESFSER	1	N-Term(iTRAQ4plex); K1(iTRAQ4plex	1.268		49		
									AYSSFNPAMSDPNRPPCR	1	N-Term(iTRAQ4plex); M9(Oxidation); C17(Methylthio)	1.169		47		
									YIGNMHGNEAVGR	1	N-Term(iTRAQ4plex	0.747		46		
									VAVPYSPAAGVDFELESFSER	1	N-Term(iTRAQ4plex	0.911		46		
119395750	KRT1	keratin, type II cytoskeletal 1	29.81	14	17	96	1.882	66								

									FSSCGGGGGSFGAGGGFGSR	5	N-Term(iTRAQ4plex); C4(Methylthio	2.205	6.97	151	2	949.90
									FLEQQNQVLQTK	12	N-Term(iTRAQ4plex); K12(iTRAQ4plex		6.7	96	2	882.50
									SLNNQFASFIDK	11	N-Term(iTRAQ4plex); K12(iTRAQ4plex	2.047	6.4	85	2	836.45
									LNDLEDALQQAK	10	N-Term(iTRAQ4plex); K12(iTRAQ4plex	1.776	6.01	101	2	823.45
									QISNLQQSISDAEQR	5	N-Term(iTRAQ4plex	1.677	5.83	92	2	930.98
									SGGGFSSGSAGIINYQR	2	N-Term(iTRAQ4plex	1.06	5.82	116	2	901.45
									TNAENEFVTIK	13	N-Term(iTRAQ4plex); K11(iTRAQ4plex	1.733	5.2	82	2	777.42
									SKAEAESLYQSK	3	N-Term(iTRAQ4plex); K2(iTRAQ4plex); K12(iTRAQ4plex)	1.596	4.65	69	2	886.99
									YEELQITAGR	16	N-Term(iTRAQ4plex		4.29	67	2	662.36
									NMQDMVEDYR	5	N-Term(iTRAQ4plex	1.767	4.19	48	2	722.82
									AEAESLYQSK	2	N-Term(iTRAQ4plex); K10(iTRAQ4plex	1.974	3.91	72	2	707.38
									WELLQQVDTSTR	2	N-Term(iTRAQ4plex	2.292	3.78	68	2	810.43
									LALDIEIATYR	5	N-Term(iTRAQ4plex		3.43	51	2	711.41
									SLDLDSIIAEVK	4	N-Term(iTRAQ4plex); K12(iTRAQ4plex	1.936		78		
									THNLEPYFESFINNLR	2	N-Term(iTRAQ4plex	3.164		52		
									SISISVAR	2	N-Term(iTRAQ4plex	1.817		50		
									SLNNQFASFIDKVR	1	N-Term(iTRAQ4plex); K12(iTRAQ4plex	1.077		41		
42740907	CLU	clusterin isoform 2 preproprotein	29.62	0	14	382		52.5								
									QQTHMLDVMQDHFSR	12	N-Term(iTRAQ4plex); M5(Oxidation		8.02	79	3	678.32
									QQTHMLDVMQDHFSR	42	N-Term(iTRAQ4plex		7.4	77	3	672.99
									KTLLSNLEEAK	23	N-Term(iTRAQ4plex); K1(iTRAQ4plex); K11(iTRAQ4plex)		6.37	77	3	560.01
									VTTVASHTSDSDVPSGVTEVVV	16	N-Term(iTRAQ4plex); K23(iTRAQ4plex		6.34	96	3	868.13
									LFDSDPITVTVPVEVSR	56	N-Term(iTRAQ4plex		6.01	113	2	1009.55
									TLLSNLEEAK	45	N-Term(iTRAQ4plex); K10(iTRAQ4plex		5.21	79	2	703.41
									RELDESLQVAER	9	N-Term(iTRAQ4plex		5.19	66	2	794.92
									EIQNAVNGVK	4	N-Term(iTRAQ4plex); K10(iTRAQ4plex		5.15	76	2	680.39
									ASSIIDELFQDR	29	N-Term(iTRAQ4plex		5.05	89	2	769.41
									ELDESLQVAER	53	N-Term(iTRAQ4plex		4.85	68	2	716.87
									IDSLLENDR	35	N-Term(iTRAQ4plex		4.59	54	2	609.82
									TLLSNLEEAKK	2	N-Term(iTRAQ4plex); K10(iTRAQ4plex); K11(iTRAQ4plex)		3.89	55	2	839.52
									FMETVAEK	49	N-Term(iTRAQ4plex); K8(iTRAQ4plex		3.84	62	2	621.84
									KYNELLK	3	N-Term(iTRAQ4plex); K1(iTRAQ4plex); K7(iTRAQ4plex)		3.61	43	3	447.29
									FMETVAEK	2	N-Term(iTRAQ4plex); M2(Oxidation); K8(iTRAQ4plex)		3.18	50	2	629.83
91823602	ENPP2	ectonucleotide pyrophosphatase/phosphodiesterase family member 2 isoform 2 preproprotein	29.55	0	21	255		98.9								
									NGVNVISGPIFDYDYDGLHDTEDK	6	N-Term(iTRAQ4plex); K24(iTRAQ4plex		7.77	86	3	991.14
									AGTFFWSVVIPHER	25	N-Term(iTRAQ4plex		5.42	65	2	895.48
									CFFQGDHGFDNK	20	N-Term(iTRAQ4plex); C1(Methylthio); K12(iTRAQ4plex)		5.18	79	2	874.89
									YGPFGPEMTNPLR	33	N-Term(iTRAQ4plex		5.15	74	2	811.91
									DIEHLTSLDFFR	35	N-Term(iTRAQ4plex		4.85	79	2	818.93
									IVGQLMDGLK	19	N-Term(iTRAQ4plex); K10(iTRAQ4plex		4.63	72	2	681.41
									RVWNYFQR	8	N-Term(iTRAQ4plex		4.49	40	3	438.24
									RIEDIHLLVER	3	N-Term(iTRAQ4plex		4.42	60	2	768.95
									IEDIHLLVER	12	N-Term(iTRAQ4plex		4.41	64	2	690.90
									SYPEILTLK	13	N-Term(iTRAQ4plex); K9(iTRAQ4plex		4.27	64	2	676.41
									CVNVIFVGDHGMEDVTCDR	4	N-Term(iTRAQ4plex); C1(Methylthio); C17(Methylthio)		4.2	57	3	782.34
									WWGGQPLWITATK	15	N-Term(iTRAQ4plex); K13(iTRAQ4plex		3.88	84	2	916.51
									YGPFGPEMTNPLR	3	N-Term(iTRAQ4plex); M8(Oxidation		3.82	53	2	819.91
									KPLDVYK	3	N-Term(iTRAQ4plex); K1(iTRAQ4plex); K7(iTRAQ4plex)		3.58	46	3	432.28
									WVEELMK	36	N-Term(iTRAQ4plex); K7(iTRAQ4plex		3.49	52	2	611.84
									TYLHTYESEI	3	N-Term(iTRAQ4plex		3.41	33	2	700.35
									QMSYGFLFPPYLSSSPEAK	3	N-Term(iTRAQ4plex); K19(iTRAQ4plex		3.17	57	2	1219.12
									TEFLSNYLTNVDDITLVPGTLGR	3	N-Term(iTRAQ4plex			52		
									VMPNIEK	7	N-Term(iTRAQ4plex); K7(iTRAQ4plex			52		
									YDAFLVTNMVPMYPAFK	1	N-Term(iTRAQ4plex); K17(iTRAQ4plex			38		
									VWNYFQR	1	N-Term(iTRAQ4plex			37		
									QMSYGFLFPPYLSSSPEAK	1	N-Term(iTRAQ4plex); M2(Oxidation); K19(iTRAQ4plex)			34		
208022622	RPS27A	ubiquitin-40S ribosomal protein S27a precursor	29.49	0	5	96		18								
									TITLEVEPSDTIENVK	20	N-Term(iTRAQ4plex); K16(iTRAQ4plex		5.99	89	2	1038.57
									LIFAGKQLEDGR	16	N-Term(iTRAQ4plex); K6(iTRAQ4plex		5	73	2	817.98
									TLSDYNIQK	22	N-Term(iTRAQ4plex); K9(iTRAQ4plex		4.71	67	2	685.38
									ESTLHLVLR	37	N-Term(iTRAQ4plex		4.63	58	2	606.36
									TLSDYNIQKESTLHLVLR	1	N-Term(iTRAQ4plex); K9(iTRAQ4plex			35		
67782358	CFB	complement factor B preproprotein	28.93	21	21	288	0.798	85.5								
									HVIILMTDGLHNMGGDPITVIDEIR	2	N-Term(iTRAQ4plex); M13(Oxidation	0.778	7.07	72	3	973.85
									VSEADSSNADWVTK	16	N-Term(iTRAQ4plex); K14(iTRAQ4plex	0.844	6.5	115	2	898.95
									QLNEINYEDHK	17	N-Term(iTRAQ4plex); K11(iTRAQ4plex	0.786	6.11	78	3	564.30
									LPPTTTCQQQK	34	N-Term(iTRAQ4plex); C7(Methylthio); K11(iTRAQ4plex)	0.797	5.3	72	3	526.95
									HVIILMTDGLHNMGGDPITVIDEIR	2	N-Term(iTRAQ4plex	0.793	5.19	86	3	968.51
									DFHINLFQVLPWLK	9	N-Term(iTRAQ4plex); K14(iTRAQ4plex	0.817	5.11	43	3	686.73
									LEDSVTYHCSR	11	N-Term(iTRAQ4plex); C9(Methylthio	0.82	4.82	66	3	500.56
									VKDISEVVTPR	8	N-Term(iTRAQ4plex); K2(iTRAQ4plex	0.85	4.76	59	3	510.97
									YGLVTYATYPK	24	N-Term(iTRAQ4plex); K11(iTRAQ4plex	0.813	4.73	77	2	782.44
									ALFVSEEEKK	11	N-Term(iTRAQ4plex); K9(iTRAQ4plex); K10(iTRAQ4plex)	0.757	4.69	62	2	806.46
									EAGIPEFYDYDVALIK	19	N-Term(iTRAQ4plex); K16(iTRAQ4plex	0.802	4.62	60	2	1066.06
									KEAGIPEFYDYDVALIK	4	N-Term(iTRAQ4plex); K1(iTRAQ4plex); K17(iTRAQ4plex)	0.798	4.47	53	3	801.78
									ALFVSEEEK	10	N-Term(iTRAQ4plex); K9(iTRAQ4plex	0.905	4.39	65	2	670.37
									CLVNLIEK	32	N-Term(iTRAQ4plex); C1(Methylthio); K8(iTRAQ4plex)	0.732	4.13	53	2	633.37
									DISEVVTPR	27	N-Term(iTRAQ4plex	0.759	4.12	56	2	580.33
									YGQTIRPICLPCTEGTTR	2	N-Term(iTRAQ4plex); C9(Methylthio); C12(Methylthio)	0.73	4.1	40	3	749.03
									STGSWSTLK	2	N-Term(iTRAQ4plex); K9(iTRAQ4plex	0.746	4.07	60	2	627.85
									EELLPAQDIK	23	N-Term(iTRAQ4plex); K10(iTRAQ4plex	0.809	4.02	62	2	722.42

									FLCTGGVSPYADPNTCF	8	N-Term(iTRAQ4plex); C3(Methylthio); C16(Methylthio)	0.721	4.01	64	2	1018.95
									DLLYIGK	20	N-Term(iTRAQ4plex); K7(iTRAQ4plex	0.842	3.25	42	2	555.35
									EKLQDEDLGFL	6	N-Term(iTRAQ4plex); K2(iTRAQ4plex	0.776	3.15	59	2	797.94
									KEVYIK	1	N-Term(iTRAQ4plex); K1(iTRAQ4plex); K6(iTRAQ4plex)	0.76		31		
283806712	CLU	clusterin isoform 3	28.91	0	14	382		53.6								
									QQTHMLDVMQDHFSF	12	N-Term(iTRAQ4plex); M5(Oxidation		8.02	79	3	678.32
									QQTHMLDVMQDHFSF	42	N-Term(iTRAQ4plex		7.4	77	3	672.99
									KTLLSNLEEAK	23	N-Term(iTRAQ4plex); K1(iTRAQ4plex); K11(iTRAQ4plex)		6.37	77	3	560.01
									VTTVASHTSDSDVPSGVTEVVV	16	N-Term(iTRAQ4plex); K23(iTRAQ4plex		6.34	96	3	868.13
									LFDSDPITVTVPVEVSF	56	N-Term(iTRAQ4plex		6.01	113	2	1009.55
									TLLSNLEEAK	45	N-Term(iTRAQ4plex); K10(iTRAQ4plex		5.21	79	2	703.41
									RELDESLQVAER	9	N-Term(iTRAQ4plex		5.19	66	2	794.92
									EIQNAVNGVK	4	N-Term(iTRAQ4plex); K10(iTRAQ4plex		5.15	76	2	680.39
									ASSIIDELFQDR	29	N-Term(iTRAQ4plex		5.05	89	2	769.41
									ELDESLQVAER	53	N-Term(iTRAQ4plex		4.85	68	2	716.87
									IDSLLENDR	35	N-Term(iTRAQ4plex		4.59	54	2	609.82
									TLLSNLEEAKK	2	N-Term(iTRAQ4plex); K10(iTRAQ4plex); K11(iTRAQ4plex)		3.89	55	2	839.52
									FMETVAEK	49	N-Term(iTRAQ4plex); K8(iTRAQ4plex		3.84	62	2	621.84
									KYNELLK	3	N-Term(iTRAQ4plex); K1(iTRAQ4plex); K7(iTRAQ4plex)		3.61	43	3	447.29
									FMETVAEK	2	N-Term(iTRAQ4plex); M2(Oxidation); K8(iTRAQ4plex)		3.18	50	2	629.83
195947389	ENPP2	ectonucleotide pyrophosphatase/phosphodiesterase family member 2 isoform 3 preproprotein	28.72	0	21	255		101.9								
									NGVNVISGPIFDYDYDGLHDTEDK	6	N-Term(iTRAQ4plex); K24(iTRAQ4plex		7.77	86	3	991.14
									AGTFFWSVVIPHER	25	N-Term(iTRAQ4plex		5.42	65	2	895.48
									CFFQGDHGFDNK	20	N-Term(iTRAQ4plex); C1(Methylthio); K12(iTRAQ4plex)		5.18	79	2	874.89
									YGPFGPEMTNPLR	33	N-Term(iTRAQ4plex		5.15	74	2	811.91
									DIEHLTSLDFFR	35	N-Term(iTRAQ4plex		4.85	79	2	818.93
									IVGQLMDGLK	19	N-Term(iTRAQ4plex); K10(iTRAQ4plex		4.63	72	2	681.41
									RVWNYFQR	8	N-Term(iTRAQ4plex		4.49	40	3	438.24
									RIEDIHLLVER	3	N-Term(iTRAQ4plex		4.42	60	2	768.95
									IEDIHLLVER	12	N-Term(iTRAQ4plex		4.41	64	2	690.90
									SYPEILTLK	13	N-Term(iTRAQ4plex); K9(iTRAQ4plex		4.27	64	2	676.41
									CVNVIFVGDHGMEDVTCDF	4	N-Term(iTRAQ4plex); C1(Methylthio); C17(Methylthio)		4.2	57	3	782.34
									WWGGQPLWITATK	15	N-Term(iTRAQ4plex); K13(iTRAQ4plex		3.88	84	2	916.51
									YGPFGPEMTNPLR	3	N-Term(iTRAQ4plex); M8(Oxidation		3.82	53	2	819.91
									KPLDVYK	3	N-Term(iTRAQ4plex); K1(iTRAQ4plex); K7(iTRAQ4plex)		3.58	46	3	432.28
									WVEELMK	36	N-Term(iTRAQ4plex); K7(iTRAQ4plex		3.49	52	2	611.84
									TYLHTYESEI	3	N-Term(iTRAQ4plex		3.41	33	2	700.35
									QMSYGFLFPPYLSSSPEAK	3	N-Term(iTRAQ4plex); K19(iTRAQ4plex		3.17	57	2	1219.12
									TEFLSNYLTNVDDITLVPGTLGF	3	N-Term(iTRAQ4plex			52		
									VMPNIEK	7	N-Term(iTRAQ4plex); K7(iTRAQ4plex			52		
									YDAFLVTNMVPMYPAFK	1	N-Term(iTRAQ4plex); K17(iTRAQ4plex			38		
									VWNYFQR	1	N-Term(iTRAQ4plex			37		
									QMSYGFLFPPYLSSSPEAK	1	N-Term(iTRAQ4plex); M2(Oxidation); K19(iTRAQ4plex)			34		
62739186	CFH	complement factor H isoform a precursor	28.68	12	30	179	0.932	139								
									SSNLIILEEHLK	29	N-Term(iTRAQ4plex); K12(iTRAQ4plex	0.841	6.14	78	3	562.00
									GDAVCTESGWRPLPSCEEK	3	N-Term(iTRAQ4plex); C5(Methylthio); C16(Methylthio); K19(iTRAQ4plex)		6.06	36	3	815.37
									SIDVACHPGYALPK	6	N-Term(iTRAQ4plex); C6(Methylthio); K14(iTRAQ4plex)		6.04	44	2	902.97
									CFEGFGIDGPAIAK	4	N-Term(iTRAQ4plex); C1(Methylthio); K14(iTRAQ4plex)	1.024	6.01	81	2	879.95
									KGEWVALNPLR	12	N-Term(iTRAQ4plex); K1(iTRAQ4plex		5.42	69	3	524.32
									EIMENYNIALR	16	N-Term(iTRAQ4plex		5.1	60	2	755.40
									AQTTVTCMENGWSPTPF	2	N-Term(iTRAQ4plex); C7(Methylthio		4.88	55	2	1034.97
									DGWSAQPTCIK	7	N-Term(iTRAQ4plex); C9(Methylthio); K11(iTRAQ4plex)	1.08	4.8	56	2	770.38
									SLGNVIMVCR	2	N-Term(iTRAQ4plex); C9(Methylthio		4.52	63	2	641.34
									TGESVEFVCK	2	N-Term(iTRAQ4plex); C9(Methylthio); K10(iTRAQ4plex)	0.97	4.44	55	2	716.86
									CNMGYEYSER	9	N-Term(iTRAQ4plex); C1(Methylthio		4.42	48	2	721.29
									CTSTGWIPAPR	10	N-Term(iTRAQ4plex); C1(Methylthio		4.23	61	2	689.84
									EIMENYNIALR	4	N-Term(iTRAQ4plex); M3(Oxidation		4.22	62	2	763.40
									WSSPPQCEGLPCK	3	N-Term(iTRAQ4plex); C7(Methylthio); C12(Methylthio); K13(iTRAQ4plex)	0.754	4.16	62	2	906.42
									GEWVALNPLR	6	N-Term(iTRAQ4plex		4.16	44	2	649.87
									LSYTCEGGFR	5	N-Term(iTRAQ4plex); C5(Methylthio	0.831	4.08	35	2	661.81
									TGDEITYQCR	2	N-Term(iTRAQ4plex); C9(Methylthio		4	35	2	688.31
									RPYFPVAVGK	13	N-Term(iTRAQ4plex); K10(iTRAQ4plex		3.95	58	3	474.62
									SPDVINGSPISQK	2	N-Term(iTRAQ4plex); K13(iTRAQ4plex		3.76	38	2	815.46
									WQSIPLCVEK	2	N-Term(iTRAQ4plex); C7(Methylthio); K10(iTRAQ4plex)	0.934	3.73	52	2	768.91
									WSHPPSCIK	2	N-Term(iTRAQ4plex); C7(Methylthio); K9(iTRAQ4plex)	0.88	3.57	40	3	463.58
									IDVHLVPDR	7	N-Term(iTRAQ4plex	0.862	3.51	45	2	604.35
									NDFTWFK	15	N-Term(iTRAQ4plex); K7(iTRAQ4plex	0.987	3.48	41	2	623.33
									AGEQVTYTCATYYK	3	N-Term(iTRAQ4plex); C9(Methylthio); K14(iTRAQ4plex)	0.955	3.41	68	2	966.46
									FVCNSGYK	3	N-Term(iTRAQ4plex); C3(Methylthio); K8(iTRAQ4plex)		3.33	50	2	626.31
									CVEISCK	4	N-Term(iTRAQ4plex); C1(Methylthio); C6(Methylthio); K7(iTRAQ4plex)		3.23	44	2	581.28
									SCDIPVFMNAR	1	N-Term(iTRAQ4plex); C2(Methylthio	0.857		58		
									LEYPTCAK	2	N-Term(iTRAQ4plex); C6(Methylthio); K8(iTRAQ4plex)			50		

									ECDTDGWTNDIPICEVVK	1	N-Term(iTRAQ4plex); C2(Methylthio); C14(Methylthio); K18(iTRAQ4plex)			48		
									RPCGHPGDTPFGTFTLTGGNVFEYGVK	1	N-Term(iTRAQ4plex); C3(Methylthio); K27(iTRAQ4plex)			38		
44955885	MB	myoglobin	28.57	3	3	17	0.197	17.2								
									HPGDFGADAQGAMNK	6	N-Term(iTRAQ4plex); K15(iTRAQ4plex	0.201	5.93	80	3	601.96
									VEADIPGHGQEVLIR	8	N-Term(iTRAQ4plex	0.194	4.88	90	3	592.99
									HGATVLTALGGILK	3	N-Term(iTRAQ4plex); K14(iTRAQ4plex	0.248		81		
259089433	SCG3	secretogranin-3 isoform 2	28.39	0	8	50		26.9								
									GENDETVSNTLTLTNGLER	6	N-Term(iTRAQ4plex		7.02	84	2	1104.05
									GILDKEEAEAIK	10	N-Term(iTRAQ4plex); K5(iTRAQ4plex); K12(iTRAQ4plex)		5.53	73	2	874.52
									EKETLITIMK	4	N-Term(iTRAQ4plex); K2(iTRAQ4plex); K10(iTRAQ4plex)		4.75	62	2	819.49
									GKTEAYLEAIR	2	N-Term(iTRAQ4plex); K2(iTRAQ4plex		4.65	43	2	769.94
									TEAYLEAIR	14	N-Term(iTRAQ4plex		4.37	48	2	605.34
									ETLITIMK	2	N-Term(iTRAQ4plex); K8(iTRAQ4plex		3.76	48	2	618.87
									TLIDFVK	11	N-Term(iTRAQ4plex); K7(iTRAQ4plex		3.24	54	2	562.35
									LFPAPSEK	1	N-Term(iTRAQ4plex); K8(iTRAQ4plex			40		
262050546	KNG1	kininogen-1 isoform 3	28.39	0	11	111		43.8								
									DIPTNSPELEETLTHTITK	12	N-Term(iTRAQ4plex); K19(iTRAQ4plex		7.73	108	3	809.77
									YNSQNQSNNQFVLYR	9	N-Term(iTRAQ4plex		6.17	84	2	1009.99
									ESNEELTESCETK	2	N-Term(iTRAQ4plex); C10(Methylthio); K13(iTRAQ4plex)		5.95	82	2	916.91
									TVGSDTFYSFK	28	N-Term(iTRAQ4plex); K11(iTRAQ4plex		5.6	74	2	770.39
									AATGECTATVGK	12	N-Term(iTRAQ4plex); C6(Methylthio); K12(iTRAQ4plex)		5.53	88	2	721.87
									RPPGFSPFR	15	N-Term(iTRAQ4plex		4.84	74	3	402.23
									KYNSQNQSNNQFVLYR	3	N-Term(iTRAQ4plex); K1(iTRAQ4plex		4.72	78	3	764.40
									KYFIDFVAR	16	N-Term(iTRAQ4plex); K1(iTRAQ4plex		4.47	66	3	482.95
									TWQDCEYK	7	N-Term(iTRAQ4plex); C5(Methylthio); K8(iTRAQ4plex)		3.76	41	2	703.82
									IASFSQNCDIYPGK	3	N-Term(iTRAQ4plex); C8(Methylthio); K14(iTRAQ4plex)		3.45	71	2	938.96
									YFIDFVAR	4	N-Term(iTRAQ4plex			37		
21361302	SERPINA4	kallistatin precursor	28.34	11	11	51	0.975	48.5								
									VGSALFLSHNLK	9	N-Term(iTRAQ4plex); K12(iTRAQ4plex	0.944	6.08	81	2	787.47
									FSISGSYVLDQILPR	4	N-Term(iTRAQ4plex	0.968	4.34	88	2	920.01
									LGFTDLFSK	4	N-Term(iTRAQ4plex); K9(iTRAQ4plex	1.019	4.1	62	2	658.38
									ALWEKPFISSR	3	N-Term(iTRAQ4plex); K5(iTRAQ4plex	0.896	4.06	54	3	541.32
									IAPANADFAFR	5	N-Term(iTRAQ4plex	0.887	4.01	58	2	668.86
									FYYLIASETPGK	4	N-Term(iTRAQ4plex); K12(iTRAQ4plex	1.027	3.99	61	2	838.96
									IVDLVSELK	6	N-Term(iTRAQ4plex); K9(iTRAQ4plex	1.006	3.95	66	2	652.41
									WADLSGITK	10	N-Term(iTRAQ4plex); K9(iTRAQ4plex	1.007	3.89	65	2	639.86
									GDATVFFILPNQGK	4	N-Term(iTRAQ4plex); K14(iTRAQ4plex	0.903	3.61	60	2	898.00
226529917	TPI1	triosephosphate isomerase isoform 2	28.32	0	7	24		30.8								
									DCGATWVVLGHSER	6	N-Term(iTRAQ4plex); C2(Methylthio		5.75	88	3	573.94
									IIYGGSVTGATCK	2	N-Term(iTRAQ4plex); C12(Methylthio); K13(iTRAQ4plex)		5.38	82	2	802.43
									KFFVGGNWK	2	N-Term(iTRAQ4plex); K1(iTRAQ4plex); K9(iTRAQ4plex)		5.17	40	3	505.64
									VVLAYEPVWAIGTGK	3	N-Term(iTRAQ4plex); K15(iTRAQ4plex		4.26	62	2	946.05
									VPADTEVVCAPPTAYIDFAR	2	N-Term(iTRAQ4plex); C9(Methylthio		4.07	55	2	1163.07
									IAVAAQNCYK	6	N-Term(iTRAQ4plex); C8(Methylthio); K10(iTRAQ4plex)		3.78	62	2	707.87
									FFVGGNWK	3	N-Term(iTRAQ4plex); K8(iTRAQ4plex		3.19	47	2	621.85
38201686	RNASE1	ribonuclease pancreatic precursor	28.21	2	2	39	0.908	17.6								
									QHMDSDSSPSSSSTYCNQMMR	21	N-Term(iTRAQ4plex); C16(Methylthio	0.822	7.68	83	3	857.01
									QHMDSDSSPSSSSTYCNQMMR	16	N-Term(iTRAQ4plex); M3(Oxidation); C16(Methylthio)	1.044	6.11	79	3	862.34
									CKPVNTFVHEPLVDVQNVCFQEK	2	N-Term(iTRAQ4plex); C1(Methylthio); K2(iTRAQ4plex); C19(Methylthio); K23(iTRAQ4plex)	0.837	6.02	55	3	1066.54
262050538	ITIH4	inter-alpha-trypsin inhibitor heavy chain H4 isoform 2 precursor	27.89	0	19	93		99.8								
									NPLVWVHASPEHVVVTR	4	N-Term(iTRAQ4plex		7.7	64	3	695.39
									QLGLPGPPDVPDHAAYHPFR	8	N-Term(iTRAQ4plex		6.08	72	3	776.74
									SPEQQETVLDGNLIIR	6	N-Term(iTRAQ4plex		5.72	83	2	978.53
									GPDVLTATVSGK	2	N-Term(iTRAQ4plex); K12(iTRAQ4plex		5.62	81	2	716.92
									ANTVQEATFQMELPK	8	N-Term(iTRAQ4plex); K15(iTRAQ4plex		5.5	71	2	998.03
									NMEQFQVSVSVAPNAK	2	N-Term(iTRAQ4plex); K16(iTRAQ4plex		4.93	77	2	1019.04
									NGIDIYSLTVDSR	2	N-Term(iTRAQ4plex		4.85	67	2	798.92
									ETLFSVMPGLK	10	N-Term(iTRAQ4plex); K11(iTRAQ4plex		4.66	65	2	755.44
									TGLLLLSDPDK	8	N-Term(iTRAQ4plex); K11(iTRAQ4plex		4.64	68	2	730.44
									LGVYELLLK	13	N-Term(iTRAQ4plex); K9(iTRAQ4plex		4.32	52	2	668.43
									AGFSWIEVTFK	4	N-Term(iTRAQ4plex); K11(iTRAQ4plex		4.22	68	2	786.94
									NVVFVIDK	10	N-Term(iTRAQ4plex); K8(iTRAQ4plex		3.94	59	2	611.38
									YIFHNFMER	3	N-Term(iTRAQ4plex		3.88	35	2	700.85
									LALDNGGLAR	2	N-Term(iTRAQ4plex		3.67	48	2	572.33
									QGPVNLLSDPEQGVEVTGQYER	3	N-Term(iTRAQ4plex		3.63	49	2	1280.14
									ILDDLSPR	3	N-Term(iTRAQ4plex		3.14	35	2	536.81
									AEAQAQYSAAVAK	3	N-Term(iTRAQ4plex); K13(iTRAQ4plex		3.14	88	2	798.43
									FSSHVGGTLGQFYQEVLWGSPAASDDG R	1	N-Term(iTRAQ4plex			55		
									EKAEAQAQYSAAVAK	1	N-Term(iTRAQ4plex); K2(iTRAQ4plex); K15(iTRAQ4plex)			41		
4507171	SPARC	SPARC precursor	27.72	8	8	49	1.006	34.6								
									TFDSSCHFFATK	5	N-Term(iTRAQ4plex); C6(Methylthio); K12(iTRAQ4plex)	0.995	5.62	69	2	862.91
									LEAGDHPVELLAR	9	N-Term(iTRAQ4plex	0.996	4.99	63	3	521.96
									YIPPCLDSELTEFPLR	4	N-Term(iTRAQ4plex); C5(Methylthio	0.983	4.43	92	2	1042.02
									APLIPMEHCTTR	8	N-Term(iTRAQ4plex); C9(Methylthio	0.911	4.37	43	3	520.26
									LHLDYIGPCK	16	N-Term(iTRAQ4plex); C9(Methylthio); K10(iTRAQ4plex)	1.006	4.09	71	2	746.90
									NVLVTLYER	4	N-Term(iTRAQ4plex	1.072	3.61	37	2	625.87
									FFETCDLDNDK	1	N-Term(iTRAQ4plex); C5(Methylthio); K11(iTRAQ4plex)	1.076		57		
120433590	DBI	acyl-CoA-binding protein isoform 3	27.59	0	2	8		10								

									TKPSDEEMLFIYGHYK	2	N-Term(iTRAQ4plex); K2(iTRAQ4plex); K16(iTRAQ4plex)		6.37	55	3	797.42
									WDAWNELK	6	N-Term(iTRAQ4plex); K8(iTRAQ4plex		3.93	46	2	675.36
33286420	PKM	pyruvate kinase isozymes M1/M2 isoform M1	27.5	1	13	55	0.421	58								
									KGVNLPGAAVDLPAVSEK	4	N-Term(iTRAQ4plex); K1(iTRAQ4plex); K18(iTRAQ4plex)		5.68	69	3	733.11
									DPVQEAWAEDVDLR	2	N-Term(iTRAQ4plex		5.36	67	2	893.94
									RFDEILEASDGIMVAR	5	N-Term(iTRAQ4plex		5.1	91	2	983.52
									NTGIICTIGPASR	4	N-Term(iTRAQ4plex); C6(Methylthio		4.99	56	2	746.89
									ITLDNAYMEK	12	N-Term(iTRAQ4plex); K10(iTRAQ4plex		4.74	74	2	743.40
									GDLGIEIPAEK	9	N-Term(iTRAQ4plex); K11(iTRAQ4plex		4.18	56	2	715.41
									LDIDSPPITAR	3	N-Term(iTRAQ4plex		4.12	52	2	671.38
									VNFAMNVGK	2	N-Term(iTRAQ4plex); K9(iTRAQ4plex		3.93	48	2	634.36
									KLFEELVR	4	N-Term(iTRAQ4plex); K1(iTRAQ4plex	0.421	3.82	40	3	441.28
									CDENILWLDYK	1	N-Term(iTRAQ4plex); C1(Methylthio); K11(iTRAQ4plex)			55		
									GIFPVLCK	6	N-Term(iTRAQ4plex); C7(Methylthio); K8(iTRAQ4plex)			49		
									AGKPVICATQMLESMIK	2	N-Term(iTRAQ4plex); K3(iTRAQ4plex); C7(Methylthio); K17(iTRAQ4plex)			42		
									FDEILEASDGIMVAR	1	N-Term(iTRAQ4plex			41		
120433593	DBI	acyl-CoA-binding protein isoform 2	27.27	0	2	8		10.1								
									TKPSDEEMLFIYGHYK	2	N-Term(iTRAQ4plex); K2(iTRAQ4plex); K16(iTRAQ4plex)		6.37	55	3	797.42
									WDAWNELK	6	N-Term(iTRAQ4plex); K8(iTRAQ4plex		3.93	46	2	675.36
31542984	ITIH4	inter-alpha-trypsin inhibitor heavy chain H4 isoform 1 precursor	26.99	0	19	93		103.3								
									NPLVWVHASPEHVVVTR	4	N-Term(iTRAQ4plex		7.7	64	3	695.39
									QLGLPGPPDVPDHAAYHPFR	8	N-Term(iTRAQ4plex		6.08	72	3	776.74
									SPEQQETVLDGNLIIR	6	N-Term(iTRAQ4plex		5.72	83	2	978.53
									GPDVLTATVSGK	2	N-Term(iTRAQ4plex); K12(iTRAQ4plex		5.62	81	2	716.92
									ANTVQEATFQMELPK	8	N-Term(iTRAQ4plex); K15(iTRAQ4plex		5.5	71	2	998.03
									NMEQFQVSVSVAPNAK	2	N-Term(iTRAQ4plex); K16(iTRAQ4plex		4.93	77	2	1019.04
									NGIDIYSLTVDSR	2	N-Term(iTRAQ4plex		4.85	67	2	798.92
									ETLFSVMPGLK	10	N-Term(iTRAQ4plex); K11(iTRAQ4plex		4.66	65	2	755.44
									TGLLLLSDPDK	8	N-Term(iTRAQ4plex); K11(iTRAQ4plex		4.64	68	2	730.44
									LGVYELLLK	13	N-Term(iTRAQ4plex); K9(iTRAQ4plex		4.32	52	2	668.43
									AGFSWIEVTFK	4	N-Term(iTRAQ4plex); K11(iTRAQ4plex		4.22	68	2	786.94
									NVVFVIDK	10	N-Term(iTRAQ4plex); K8(iTRAQ4plex		3.94	59	2	611.38
									YIFHNFMER	3	N-Term(iTRAQ4plex		3.88	35	2	700.85
									LALDNGGLAR	2	N-Term(iTRAQ4plex		3.67	48	2	572.33
									QGPVNLLSDPEQGVEVTGQYER	3	N-Term(iTRAQ4plex		3.63	49	2	1280.14
									ILDDLSPR	3	N-Term(iTRAQ4plex		3.14	35	2	536.81
									AEAQAQYSAAVAK	3	N-Term(iTRAQ4plex); K13(iTRAQ4plex		3.14	88	2	798.43
									FSSHVGGTLGQFYQEVLWGSPAASDDGR	1	N-Term(iTRAQ4plex			55		
									EKAEAQAQYSAAVAK	1	N-Term(iTRAQ4plex); K2(iTRAQ4plex); K15(iTRAQ4plex)			41		
4757826	B2M	beta-2-microglobulin precursor	26.89	2	2	126	1.184	13.7								
									SNFLNCYVSGFHPSDIEVDLLK	11	N-Term(iTRAQ4plex); C6(Methylthio); K22(iTRAQ4plex)	1.284	5.57	92	3	944.47
									VEHSDLSFSK	115	N-Term(iTRAQ4plex); K10(iTRAQ4plex	1.183	4.74	84	3	479.59
4557287	AGT	angiotensinogen preproprotein	26.8	10	10	277	0.73	53.1								
									SLDFTELDVAAEK	28	N-Term(iTRAQ4plex); K13(iTRAQ4plex	0.758	6.73	96	2	863.46
									VLSALQAVQGLLVAQGR	37	N-Term(iTRAQ4plex	0.843	6.59	135	2	934.07
									ALQDQLVLVAAK	38	N-Term(iTRAQ4plex); K12(iTRAQ4plex	0.749	6.32	96	2	778.99
									QPFVQGLALYTPVVLPR	41	N-Term(iTRAQ4plex	0.698	6.2	88	2	1021.60
									AAMVGMLANFLGFR	9	N-Term(iTRAQ4plex	0.677	5.41	74	2	821.44
									AAMVGMLANFLGFR	14	N-Term(iTRAQ4plex); M3(Oxidation	0.7	4.9	70	2	829.44
									FMQAVTGWK	37	N-Term(iTRAQ4plex); K9(iTRAQ4plex	0.677	4.43	72	2	678.38
									DPTFIPAPIQAK	20	N-Term(iTRAQ4plex); K12(iTRAQ4plex	0.753	4.37	70	2	793.47
									LQAILGVPWK	32	N-Term(iTRAQ4plex); K10(iTRAQ4plex	0.729	4.32	82	2	706.95
									AAMVGMLANFLGFR	2	N-Term(iTRAQ4plex); M3(Oxidation); M6(Oxidation)	0.801	4.22	51	2	837.44
									FMQAVTGWK	12	N-Term(iTRAQ4plex); M2(Oxidation); K9(iTRAQ4plex)	0.738	3.83	71	2	686.37
									ADSQAQLLLSTVVGVFTAPGLHLK	6	N-Term(iTRAQ4plex); K24(iTRAQ4plex	1.044	3.68	56	3	918.54
									LQAILGVPWKDK	1	N-Term(iTRAQ4plex); K10(iTRAQ4plex); K12(iTRAQ4plex)	0.875		27		
4502067	AMBP	protein AMBP preproprotein	26.7	7	7	33	0.883	39								
									GVCEETSGAYEK	6	N-Term(iTRAQ4plex); C3(Methylthio); K12(iTRAQ4plex)	0.757	6.21	78	2	803.87
									TVAACNLPIVR	4	N-Term(iTRAQ4plex); C5(Methylthio	0.926	4.62	40	2	673.88
									EYCGVPGDGDEELLR	8	N-Term(iTRAQ4plex); C3(Methylthio	1.324	4.47	90	2	921.41
									GECVPGEQEPEPILIPR	5	N-Term(iTRAQ4plex); C3(Methylthio	0.775	4.04	52	2	1027.02
									VVAQGVGIPEDSIFTMADR	2	N-Term(iTRAQ4plex	0.675	3.95	48	2	1075.06
									ETLLQDFR	3	N-Term(iTRAQ4plex	0.835	3.52	38	2	583.32
									AFIQLWAFDAVK	3	N-Term(iTRAQ4plex); K12(iTRAQ4plex	1.2	3.22	65	2	848.99
									VVAQGVGIPEDSIFTMADR	2	N-Term(iTRAQ4plex); M16(Oxidation	0.661		45		
54112390	CACNA2D1	voltage-dependent calcium channel subunit alpha-2/delta-1	26.67	25	25	101	0.754	123.1								
									SGPGAYESGIMVSK	4	N-Term(iTRAQ4plex); K14(iTRAQ4plex	0.844	6.89	95	2	835.94
									VFTFSVGQHNYDR	7	N-Term(iTRAQ4plex	0.734	6.3	62	3	571.95
									TASGVNQLVDIYEK	8	N-Term(iTRAQ4plex); K14(iTRAQ4plex	0.868	6.21	87	2	913.00
									EDFASNEVVYYNAK	2	N-Term(iTRAQ4plex); K14(iTRAQ4plex	0.826	5.91	96	2	968.98
									IKPVFIEDANFGR	9	N-Term(iTRAQ4plex); K2(iTRAQ4plex	0.733	5.43	80	3	598.68
									YQDLYTVEPNNAR	6	N-Term(iTRAQ4plex	0.757	5.11	70	2	863.93
									LLKPAVVGIK	10	N-Term(iTRAQ4plex); K3(iTRAQ4plex); K10(iTRAQ4plex)	0.801	4.96	48	3	490.68
									YYPASPWVDNSR	4	N-Term(iTRAQ4plex	0.748	4.46	63	2	799.89
									KTPNNPSCNADLINR	2	N-Term(iTRAQ4plex); K1(iTRAQ4plex); C8(Methylthio)	0.8	4.38	49	3	664.34
									TPNNPSCNADLINR	4	N-Term(iTRAQ4plex); C7(Methylthio	0.696	4.29	81	2	859.91
									FFGEIDPSLMR	2	N-Term(iTRAQ4plex	0.615	4.08	39	2	728.38
									AVEIYIQGK	4	N-Term(iTRAQ4plex); K9(iTRAQ4plex	0.613	4.08	58	2	654.89
									GPIQWMACENK	2	N-Term(iTRAQ4plex); C8(Methylthio); K11(iTRAQ4plex)	0.688	4.02	48	2	805.89
									IIMLFTDGGEER	11	N-Term(iTRAQ4plex	0.717	3.97	53	2	762.90

									SYDYQSVCEPGAAPK	2	N-Term(iTRAQ4plex); C8(Methylthio); K15(iTRAQ4plex)	0.746	3.92	60	2	974.95
									GYYYEIPSIGAIR	4	N-Term(iTRAQ4plex	0.689	3.75	52	2	823.44
									EAGENWQENPETYEDSFYK	2	N-Term(iTRAQ4plex); K19(iTRAQ4plex	0.788	3.63	59	2	1312.59
									RPWYIQGAASPK	3	N-Term(iTRAQ4plex); K12(iTRAQ4plex	0.734	3.56	52	3	554.65
									AKLEETITQAR	2	N-Term(iTRAQ4plex); K2(iTRAQ4plex	0.939	3.43	40	3	516.64
									DYCNDLK	2	N-Term(iTRAQ4plex); C3(Methylthio); K7(iTRAQ4plex)	0.732	3.4	31	2	602.79
									FVVTDGGITR	5	N-Term(iTRAQ4plex	0.755	3.3	43	2	604.84
									DSETLKPDNFEESGYTFIAPR	1	N-Term(iTRAQ4plex); K6(iTRAQ4plex	0.688		55		
									NREEDPSLLWQVFGSATGLAR	2	N-Term(iTRAQ4plex	0.565		52		
									SGPGAYESGIMVSK	1	N-Term(iTRAQ4plex); M11(Oxidation); K14(iTRAQ4plex)	0.682		51		
									LEETITQAR	1	N-Term(iTRAQ4plex	0.74		39		
									RRPWYIQGAASPK	1	N-Term(iTRAQ4plex); K13(iTRAQ4plex	0.983		37		
42716297	CLU	clusterin isoform 1	26.55	0	14	382		57.8								
									QQTHMLDVMQDHFSR	12	N-Term(iTRAQ4plex); M5(Oxidation		8.02	79	3	678.32
									QQTHMLDVMQDHFSR	42	N-Term(iTRAQ4plex		7.4	77	3	672.99
									KTLLSNLEEAK	23	N-Term(iTRAQ4plex); K1(iTRAQ4plex); K11(iTRAQ4plex)		6.37	77	3	560.01
									VTTVASHTSDSDVPSGVTEVVV	16	N-Term(iTRAQ4plex); K23(iTRAQ4plex		6.34	96	3	868.13
									LFDSDPITVTVPVEVSR	56	N-Term(iTRAQ4plex		6.01	113	2	1009.55
									TLLSNLEEAK	45	N-Term(iTRAQ4plex); K10(iTRAQ4plex		5.21	79	2	703.41
									RELDESLQVAER	9	N-Term(iTRAQ4plex		5.19	66	2	794.92
									EIQNAVNGVK	4	N-Term(iTRAQ4plex); K10(iTRAQ4plex		5.15	76	2	680.39
									ASSIIDELFQDR	29	N-Term(iTRAQ4plex		5.05	89	2	769.41
									ELDESLQVAER	53	N-Term(iTRAQ4plex		4.85	68	2	716.87
									IDSLLENDR	35	N-Term(iTRAQ4plex		4.59	54	2	609.82
									TLLSNLEEAKK	2	N-Term(iTRAQ4plex); K10(iTRAQ4plex); K11(iTRAQ4plex)		3.89	55	2	839.52
									FMETVAEK	49	N-Term(iTRAQ4plex); K8(iTRAQ4plex		3.84	62	2	621.84
									KYNELLK	3	N-Term(iTRAQ4plex); K1(iTRAQ4plex); K7(iTRAQ4plex)		3.61	43	3	447.29
									FMETVAEK	2	N-Term(iTRAQ4plex); M2(Oxidation); K8(iTRAQ4plex)		3.18	50	2	629.83
91823274	ENPP2	ectonucleotide pyrophosphatase/phosphodiesterase family member 2 isoform 1 preproprotein	26.45	0	20	219		105.1								
									NGVNVISGPIFDYDYDGLHDTEDK	6	N-Term(iTRAQ4plex); K24(iTRAQ4plex		7.77	86	3	991.14
									AGTFFWSVVIPHER	25	N-Term(iTRAQ4plex		5.42	65	2	895.48
									CFFQGDHGFDNK	20	N-Term(iTRAQ4plex); C1(Methylthio); K12(iTRAQ4plex)		5.18	79	2	874.89
									DIEHLTSLDFFR	35	N-Term(iTRAQ4plex		4.85	79	2	818.93
									IVGQLMDGLK	19	N-Term(iTRAQ4plex); K10(iTRAQ4plex		4.63	72	2	681.41
									RVWNYFQR	8	N-Term(iTRAQ4plex		4.49	40	3	438.24
									RIEDIHLLVER	3	N-Term(iTRAQ4plex		4.42	60	2	768.95
									IEDIHLLVER	12	N-Term(iTRAQ4plex		4.41	64	2	690.90
									SYPEILTLK	13	N-Term(iTRAQ4plex); K9(iTRAQ4plex		4.27	64	2	676.41
									CVNVIFVGDHGMEDVTCDR	4	N-Term(iTRAQ4plex); C1(Methylthio); C17(Methylthio)		4.2	57	3	782.34
									WWGGQPLWITATK	15	N-Term(iTRAQ4plex); K13(iTRAQ4plex		3.88	84	2	916.51
									KPLDVYK	3	N-Term(iTRAQ4plex); K1(iTRAQ4plex); K7(iTRAQ4plex)		3.58	46	3	432.28
									WVEELMK	36	N-Term(iTRAQ4plex); K7(iTRAQ4plex		3.49	52	2	611.84
									TYLHTYESEI	3	N-Term(iTRAQ4plex		3.41	33	2	700.35
									QMSYGFLFPPYLSSSPEAK	3	N-Term(iTRAQ4plex); K19(iTRAQ4plex		3.17	57	2	1219.12
									TEFLSNYLTNVDDITLVPGTLGR	3	N-Term(iTRAQ4plex			52		
									VMPNIEK	7	N-Term(iTRAQ4plex); K7(iTRAQ4plex			52		
									YDAFLVTNMVPMYPAFK	1	N-Term(iTRAQ4plex); K17(iTRAQ4plex			38		
									VWNYFQR	1	N-Term(iTRAQ4plex			37		
									QMSYGFLFPPYLSSSPEAK	1	N-Term(iTRAQ4plex); M2(Oxidation); K19(iTRAQ4plex)			34		
302191651	NRCAM	neuronal cell adhesion molecule isoform F precursor	26.27	0	23	211		130.6								
									SLPSEASEQYLTK	16	N-Term(iTRAQ4plex); K13(iTRAQ4plex		6.31	89	2	870.97
									KIDGDTIIFSNVQER	20	N-Term(iTRAQ4plex); K1(iTRAQ4plex		6.06	80	3	675.04
									AETYEGVYQCTAR	24	N-Term(iTRAQ4plex); C10(Methylthio		5.75	82	2	840.88
									NALGAIHHTISVR	31	N-Term(iTRAQ4plex		5.59	79	2	766.94
									THGMLPGLEPFSHYTLNVR	2	N-Term(iTRAQ4plex		5.43	59	3	771.74
									ERPPTFLTPEGNASNK	2	N-Term(iTRAQ4plex); K16(iTRAQ4plex		5.41	41	3	682.70
									VFNTPEGVPSAPSSLK	15	N-Term(iTRAQ4plex); K16(iTRAQ4plex		5.34	97	2	959.53
									ISWLTNGVPIEIAPDDPSR	2	N-Term(iTRAQ4plex		5.3	56	2	1112.59
									TLQIIHVSEADSGNYQCIAK	2	N-Term(iTRAQ4plex); C17(Methylthio); K20(iTRAQ4plex)		5.21	78	3	842.10
									IDGDTIIFSNVQER	5	N-Term(iTRAQ4plex		5.14	80	2	875.96
									VSQGLNGDLYFSNVLPEDTR	2	N-Term(iTRAQ4plex		5.11	101	2	1184.60
									GKPPPSFSWTR	19	N-Term(iTRAQ4plex); K2(iTRAQ4plex		4.99	59	3	516.62
									SVQLSWTPGDDNNSPITK	6	N-Term(iTRAQ4plex); K18(iTRAQ4plex		4.96	83	2	1124.08
									VQALNDMGFAPEPAVVMGHSGEDLPMV PGNVR	2	N-Term(iTRAQ4plex); M17(Oxidation		4.87	61	3	1189.25
									GSMVSFECK	10	N-Term(iTRAQ4plex); C8(Methylthio); K9(iTRAQ4plex)		4.61	57	2	661.32
									ENIVIQCEAK	12	N-Term(iTRAQ4plex); C7(Methylthio); K10(iTRAQ4plex)		4.35	70	2	740.89
									VMAVNSIGK	4	N-Term(iTRAQ4plex); K9(iTRAQ4plex		4.26	56	2	603.86
									LSPYVNYSFR	17	N-Term(iTRAQ4plex		4.09	64	2	695.37
									YIVSGTPTFVPYLIK	2	N-Term(iTRAQ4plex); K15(iTRAQ4plex		3.56	55	2	993.58
									EDYICYAR	6	N-Term(iTRAQ4plex); C5(Methylthio		3.45	41	2	611.77
									ILTFQGSK	8	N-Term(iTRAQ4plex); K8(iTRAQ4plex		3.44	55	2	591.35
									DPTWIVK	2	N-Term(iTRAQ4plex); K7(iTRAQ4plex			40		
									NEVHLEIK	1	N-Term(iTRAQ4plex); K8(iTRAQ4plex			37		
									VQALNDMGFAPEPAVVMGHSGEDLPMV PGNVR	1	N-Term(iTRAQ4plex			32		
81158224	NRCAM	neuronal cell adhesion molecule isoform B precursor	26.2	0	23	211		131								
									SLPSEASEQYLTK	16	N-Term(iTRAQ4plex); K13(iTRAQ4plex		6.31	89	2	870.97
									KIDGDTIIFSNVQER	20	N-Term(iTRAQ4plex); K1(iTRAQ4plex		6.06	80	3	675.04
									AETYEGVYQCTAR	24	N-Term(iTRAQ4plex); C10(Methylthio		5.75	82	2	840.88

									NALGAIHHTISVF	31	N-Term(iTRAQ4plex		5.59	79	2	766.94
									THGMLPGLEPFSHYTLNVF	2	N-Term(iTRAQ4plex		5.43	59	3	771.74
									ERPPTFLTPEGNASNK	2	N-Term(iTRAQ4plex); K16(iTRAQ4plex		5.41	41	3	682.70
									VFNTPEGVPSAPSSLK	15	N-Term(iTRAQ4plex); K16(iTRAQ4plex		5.34	97	2	959.53
									ISWLTNGVPIEIAPDDPSR	2	N-Term(iTRAQ4plex		5.3	56	2	1112.59
									TLQIIHVSEADSGNYQCIAK	2	N-Term(iTRAQ4plex); C17(Methylthio); K20(iTRAQ4plex)		5.21	78	3	842.10
									IDGDTIIFSNVQER	5	N-Term(iTRAQ4plex		5.14	80	2	875.96
									VSQGLNGDLYFSNVLPEDTF	2	N-Term(iTRAQ4plex		5.11	101	2	1184.60
									GKPPPSFSWTR	19	N-Term(iTRAQ4plex); K2(iTRAQ4plex		4.99	59	3	516.62
									SVQLSWTPGDDNNSPITK	6	N-Term(iTRAQ4plex); K18(iTRAQ4plex		4.96	83	2	1124.08
									VQALNDMGFAPEPAVVMGHSGEDLPMV PGNVR	2	N-Term(iTRAQ4plex); M17(Oxidation		4.87	61	3	1189.25
									GSMVSFECK	10	N-Term(iTRAQ4plex); C8(Methylthio); K9(iTRAQ4plex)		4.61	57	2	661.32
									ENIVIQCEAK	12	N-Term(iTRAQ4plex); C7(Methylthio); K10(iTRAQ4plex)		4.35	70	2	740.89
									VMAVNSIGK	4	N-Term(iTRAQ4plex); K9(iTRAQ4plex		4.26	56	2	603.86
									LSPYVNYSFR	17	N-Term(iTRAQ4plex		4.09	64	2	695.37
									YIVSGTPTFVPYLIK	2	N-Term(iTRAQ4plex); K15(iTRAQ4plex		3.56	55	2	993.58
									EDYICYAR	6	N-Term(iTRAQ4plex); C5(Methylthio		3.45	41	2	611.77
									ILTFQGSK	8	N-Term(iTRAQ4plex); K8(iTRAQ4plex		3.44	55	2	591.35
									DPTWIVK	2	N-Term(iTRAQ4plex); K7(iTRAQ4plex			40		
									NEVHLEIK	1	N-Term(iTRAQ4plex); K8(iTRAQ4plex			37		
									VQALNDMGFAPEPAVVMGHSGEDLPMV PGNVR	1	N-Term(iTRAQ4plex			32		
47132549	FN1	fibronectin isoform 6 preproprotein	26.15	0	41	301		239.5								
									RPGGEPSPEGTTGQSYNQYSQF	18	N-Term(iTRAQ4plex		9.81	124	3	847.41
									GLKPGVVYEGQLISIQQYGHQEVTF	2	N-Term(iTRAQ4plex); K3(iTRAQ4plex		7.18	46	4	772.68
									SSPVVIDASTAIDAPSNLF	22	N-Term(iTRAQ4plex		6.66	112	2	1029.05
									GDSPASSKPISINYR	6	N-Term(iTRAQ4plex); K8(iTRAQ4plex		6.38	88	2	940.51
									WCGTTQNYDADQK	9	N-Term(iTRAQ4plex); C2(Methylthio); K13(iTRAQ4plex)		6.24	91	2	932.42
									GATYNIIVEALK	12	N-Term(iTRAQ4plex); K12(iTRAQ4plex		6.03	96	2	790.47
									TETITGFQVDAVPANGQTPIQF	6	N-Term(iTRAQ4plex		5.94	102	2	1244.15
									NLQPASEYTVSLVAIK	6	N-Term(iTRAQ4plex); K16(iTRAQ4plex		5.9	98	2	1011.08
									EESPLLIGQQSTVSDVPR	20	N-Term(iTRAQ4plex		5.76	111	2	1050.06
									GFNCESKPEAEETCFDK	4	N-Term(iTRAQ4plex); C4(Methylthio); K7(iTRAQ4plex); C14(Methylthio); K17(iTRAQ4plex)		5.41	72	3	820.03
									SYTITGLQPGTDYK	14	N-Term(iTRAQ4plex); K14(iTRAQ4plex		5.37	77	2	916.49
									EATIPGHLNSYTIK	7	N-Term(iTRAQ4plex); K14(iTRAQ4plex		5.33	79	3	611.34
									HTSVQTTSSGSGPFTDVF	6	N-Term(iTRAQ4plex		5.31	102	3	670.00
									VDVIPVNLPGEHGQF	31	N-Term(iTRAQ4plex		5.22	71	3	592.00
									TYHVGEQWQK	3	N-Term(iTRAQ4plex); K10(iTRAQ4plex		5.09	48	3	521.95
									DLQFVEVTDVK	8	N-Term(iTRAQ4plex); K11(iTRAQ4plex		4.99	75	2	790.94
									WCHDNGVNYK	2	N-Term(iTRAQ4plex); C2(Methylthio); K10(iTRAQ4plex)		4.96	31	3	523.91
									VPGTSTSATLTGLTF	6	N-Term(iTRAQ4plex		4.88	95	2	803.45
									NTFAEVTGLSPGVTYYFK	4	N-Term(iTRAQ4plex); K18(iTRAQ4plex		4.59	84	2	1141.60
									DDKESVPISDTIIPAVPPPTDLF	4	N-Term(iTRAQ4plex); K3(iTRAQ4plex		4.56	44	3	921.84
									VTDATETTITISWF	11	N-Term(iTRAQ4plex		4.53	82	2	869.45
									GNLLQCICTGNGR	2	N-Term(iTRAQ4plex); C6(Methylthio); C8(Methylthio)		4.53	67	2	792.86
									YEVSVYALK	8	N-Term(iTRAQ4plex); K9(iTRAQ4plex		4.44	64	2	680.39
									VTIMWTPPESAVTGYF	12	N-Term(iTRAQ4plex		4.3	67	2	976.51
									YSFCTDHTVLVQTF	5	N-Term(iTRAQ4plex); C4(Methylthio		4.23	80	2	930.45
									TFYSCTTEGR	5	N-Term(iTRAQ4plex); C5(Methylthio		4.08	48	2	677.80
									FTNIGPDTMR	12	N-Term(iTRAQ4plex		4.04	53	2	648.33
									HYQINQQWER	3	N-Term(iTRAQ4plex		3.95	59	3	515.93
									GEWTCIAYSQLR	4	N-Term(iTRAQ4plex); C5(Methylthio		3.88	45	2	808.89
									IYLYTLNDNAR	7	N-Term(iTRAQ4plex		3.81	60	2	750.40
									ESVPISDTIIPAVPPPTDLF	3	N-Term(iTRAQ4plex		3.79	63	2	1131.13
									CDPHEATCYDDGK	2	N-Term(iTRAQ4plex); C1(Methylthio); C8(Methylthio); K13(iTRAQ4plex)		3.59	61	2	917.36
									TEIDKPSQMQVTDVQDNSISVK	3	N-Term(iTRAQ4plex); K5(iTRAQ4plex); K22(iTRAQ4plex)		3.57	88	3	965.52
									VTIMWTPPESAVTGYF	2	N-Term(iTRAQ4plex); M4(Oxidation		3.55	38	2	984.51
									WLPSSSPVTGYR	11	N-Term(iTRAQ4plex		3.48	53	2	747.40
									ITGYIIK	8	N-Term(iTRAQ4plex); K7(iTRAQ4plex		3.16	40	2	548.35
									IGDTWSK	6	N-Term(iTRAQ4plex); K7(iTRAQ4plex			56		
									LTVGLTR	1	N-Term(iTRAQ4plex			34		
									TYLGNALVCTCYGGSF	1	N-Term(iTRAQ4plex); C9(Methylthio); C11(Methylthio)			31		
4505047	LUM	lumican precursor	26.04	8	8	84	0.942	38.4								
									LKEDAVSAAFK	15	N-Term(iTRAQ4plex); K2(iTRAQ4plex); K11(iTRAQ4plex)	0.909	6.11	83	3	537.65
									SLEDLQLTHNK	12	N-Term(iTRAQ4plex); K11(iTRAQ4plex	1.041	5.81	82	3	529.30
									SLEYLDLSFNQIAR	2	N-Term(iTRAQ4plex	0.626	4.59	73	2	906.99
									ISETSLPPDMYECLR	6	N-Term(iTRAQ4plex); C13(Methylthio	0.871	4.04	67	2	972.46
									SVPMVPPGIK	25	N-Term(iTRAQ4plex); K10(iTRAQ4plex	0.979	3.85	73	2	656.89
									ILGPLSYSK	14	N-Term(iTRAQ4plex); K9(iTRAQ4plex	0.967	3.75	54	2	633.39
									FNALQYLR	6	N-Term(iTRAQ4plex	0.897	3.62	42	2	584.83
									SVPMVPPGIK	2	N-Term(iTRAQ4plex); M4(Oxidation); K10(iTRAQ4plex)	0.984	3.51	46	2	664.90
									ISNIPDEYFK	2	N-Term(iTRAQ4plex); K10(iTRAQ4plex	1.752	3.24	41	2	757.41
302191649	NRCAM	neuronal cell adhesion molecule isoform E precursor	26.01	0	23	211		131.7								
									SLPSEASEQYLTK	16	N-Term(iTRAQ4plex); K13(iTRAQ4plex		6.31	89	2	870.97
									KIDGDTIIFSNVQER	20	N-Term(iTRAQ4plex); K1(iTRAQ4plex		6.06	80	3	675.04
									AETYEGVYQCTAF	24	N-Term(iTRAQ4plex); C10(Methylthio		5.75	82	2	840.88
									NALGAIHHTISVF	31	N-Term(iTRAQ4plex		5.59	79	2	766.94
									THGMLPGLEPFSHYTLNVF	2	N-Term(iTRAQ4plex		5.43	59	3	771.74
									ERPPTFLTPEGNASNK	2	N-Term(iTRAQ4plex); K16(iTRAQ4plex		5.41	41	3	682.70
									VFNTPEGVPSAPSSLK	15	N-Term(iTRAQ4plex); K16(iTRAQ4plex		5.34	97	2	959.53
									ISWLTNGVPIEIAPDDPSR	2	N-Term(iTRAQ4plex		5.3	56	2	1112.59
									TLQIIHVSEADSGNYQCIAK	2	N-Term(iTRAQ4plex); C17(Methylthio); K20(iTRAQ4plex)		5.21	78	3	842.10

									IDGDTIIFSNVQER	5	N-Term(iTRAQ4plex		5.14	80	2	875.96
									VSQGLNGDLYFSNVLPEDTF	2	N-Term(iTRAQ4plex		5.11	101	2	1184.60
									GKPPPSFSWTR	19	N-Term(iTRAQ4plex); K2(iTRAQ4plex		4.99	59	3	516.62
									SVQLSWTPGDDNNSPITK	6	N-Term(iTRAQ4plex); K18(iTRAQ4plex		4.96	83	2	1124.08
									VQALNDMGFAPEPAVVMGHSGEDLPMV PGNVR	2	N-Term(iTRAQ4plex); M17(Oxidation		4.87	61	3	1189.25
									GSMVSFECK	10	N-Term(iTRAQ4plex); C8(Methylthio); K9(iTRAQ4plex)		4.61	57	2	661.32
									ENIVIQCEAK	12	N-Term(iTRAQ4plex); C7(Methylthio); K10(iTRAQ4plex)		4.35	70	2	740.89
									VMAVNSIGK	4	N-Term(iTRAQ4plex); K9(iTRAQ4plex		4.26	56	2	603.86
									LSPYVNYSFR	17	N-Term(iTRAQ4plex		4.09	64	2	695.37
									YIVSGTPTFVPYLIK	2	N-Term(iTRAQ4plex); K15(iTRAQ4plex		3.56	55	2	993.58
									EDYICYAR	6	N-Term(iTRAQ4plex); C5(Methylthio		3.45	41	2	611.77
									ILTFQGSK	8	N-Term(iTRAQ4plex); K8(iTRAQ4plex		3.44	55	2	591.35
									DPTWIVK	2	N-Term(iTRAQ4plex); K7(iTRAQ4plex			40		
									NEVHLEIK	1	N-Term(iTRAQ4plex); K8(iTRAQ4plex			37		
									VQALNDMGFAPEPAVVMGHSGEDLPMV PGNVR	1	N-Term(iTRAQ4plex			32		
33286418	PKM	pyruvate kinase isozymes M1/M2 isoform M2	25.99	0	12	51		57.9								
									KGVNLPGAAVDLPAVSEK	4	N-Term(iTRAQ4plex); K1(iTRAQ4plex); K18(iTRAQ4plex)		5.68	69	3	733.11
									DPVQEAWAEDVDLR	2	N-Term(iTRAQ4plex		5.36	67	2	893.94
									RFDEILEASDGIMVAR	5	N-Term(iTRAQ4plex		5.1	91	2	983.52
									NTGIICTIGPASR	4	N-Term(iTRAQ4plex); C6(Methylthio		4.99	56	2	746.89
									ITLDNAYMEK	12	N-Term(iTRAQ4plex); K10(iTRAQ4plex		4.74	74	2	743.40
									GDLGIEIPAEK	9	N-Term(iTRAQ4plex); K11(iTRAQ4plex		4.18	56	2	715.41
									LDIDSPPITAR	3	N-Term(iTRAQ4plex		4.12	52	2	671.38
									VNFAMNVGK	2	N-Term(iTRAQ4plex); K9(iTRAQ4plex		3.93	48	2	634.36
									CDENILWLDYK	1	N-Term(iTRAQ4plex); C1(Methylthio); K11(iTRAQ4plex)			55		
									GIFPVLCK	6	N-Term(iTRAQ4plex); C7(Methylthio); K8(iTRAQ4plex)			49		
									AGKPVICATQMLESMIK	2	N-Term(iTRAQ4plex); K3(iTRAQ4plex); C7(Methylthio); K17(iTRAQ4plex)			42		
									FDEILEASDGIMVAR	1	N-Term(iTRAQ4plex			41		
4504981	LGALS1	galectin-1	25.93	3	3	10	1.118	14.7								
									DSNNLCLHFNPR	4	N-Term(iTRAQ4plex); C6(Methylthio	1.149	3.94	62	3	540.59
									SFVLNLGK	4	N-Term(iTRAQ4plex); K8(iTRAQ4plex	1.085	3.91	54	2	583.36
									FNAHGDANTIVCNSK	2	N-Term(iTRAQ4plex); C12(Methylthio); K15(iTRAQ4plex)	1.042	3.35	48	3	642.32
41393602	C1S	complement C1s subcomponent precursor	25.87	14	14	137	0.941	76.6								
									MLTPEHVFIHPGWK	23	N-Term(iTRAQ4plex); K14(iTRAQ4plex	0.946	7.28	66	3	660.70
									MLTPEHVFIHPGWK	4	N-Term(iTRAQ4plex); M1(Oxidation); K14(iTRAQ4plex)	1.032	6.83	55	4	499.77
									GDSGGAFAVQDPNDK	4	N-Term(iTRAQ4plex); K15(iTRAQ4plex	0.942	6.66	99	2	883.44
									SSNNPHSPIVEEFQVPYNK	9	N-Term(iTRAQ4plex); K19(iTRAQ4plex	0.9	6.17	64	3	825.43
									QFGPYCGHGFPGPLNIETK	4	N-Term(iTRAQ4plex); C6(Methylthio); K19(iTRAQ4plex)	0.887	5.73	66	3	799.40
									TNFDNDIALVR	17	N-Term(iTRAQ4plex	0.926	5.14	62	2	711.38
									EDTPNSVWEPAK	10	N-Term(iTRAQ4plex); K12(iTRAQ4plex	1.034	5.07	88	2	830.92
									EPTMYVGSTSVQTSR	15	N-Term(iTRAQ4plex	0.951	4.98	81	2	893.95
									VEDPESTLFGSVIR	7	N-Term(iTRAQ4plex	0.934	4.82	87	2	846.95
									SNALDIIFQTDLTGQK	6	N-Term(iTRAQ4plex); K16(iTRAQ4plex	0.743	4.65	74	2	1026.57
									IIGGSDADIK	6	N-Term(iTRAQ4plex); K10(iTRAQ4plex	0.906	4.48	69	2	638.88
									GFQVVVTLR	4	N-Term(iTRAQ4plex	0.895	4.1	61	2	581.86
									EPTMYVGSTSVQTSR	4	N-Term(iTRAQ4plex); M4(Oxidation	0.905	3.87	88	2	901.94
									YHGDPMPCPK	2	N-Term(iTRAQ4plex); C8(Methylthio); K10(iTRAQ4plex)	0.996	3.82	79	2	739.85
									NYVDWIMK	5	N-Term(iTRAQ4plex); K8(iTRAQ4plex	1.104	3.45	55	2	678.87
									LQVIFK	16	N-Term(iTRAQ4plex); K6(iTRAQ4plex	0.992		42		
									NYVDWIMK	1	N-Term(iTRAQ4plex); M7(Oxidation); K8(iTRAQ4plex)	1.254		36		
37577172	RNASE4	ribonuclease 4 precursor	25.85	3	3	5	0.876	16.8								
									FNTFIHEDIWNIR	2	N-Term(iTRAQ4plex	0.876	4.09	47	3	616.99
									YCNLMMQR	2	N-Term(iTRAQ4plex); C2(Methylthio	0.991	3.21	27	2	624.78
									VVIACEGNPQVPVHFDC	1	N-Term(iTRAQ4plex); C5(Methylthio	0.644		36		
33356541	PLTP	phospholipid transfer protein isoform b precursor	25.85	0	9	80		49.2								
									AGALQLLLVGDK	6	N-Term(iTRAQ4plex); K12(iTRAQ4plex		5.68	98	2	743.47
									DPVASTSNLDMDFR	11	N-Term(iTRAQ4plex		5.33	100	2	856.41
									DPVASTSNLDMDFR	3	N-Term(iTRAQ4plex); M11(Oxidation		4.27	81	2	864.41
									FLEQELETITIPDLR	2	N-Term(iTRAQ4plex		3.96	42	2	981.04
									SSVDELVGIDYSLMK	7	N-Term(iTRAQ4plex); K15(iTRAQ4plex		3.95	100	2	972.52
									VYDFLSTFITSGMR	4	N-Term(iTRAQ4plex		3.89	72	2	890.96
									VPHDLDMLLR	29	N-Term(iTRAQ4plex		3.86	66	3	451.59
									GAFFPLTER	13	N-Term(iTRAQ4plex		3.58	45	2	591.33
									AVEPQLQEEER	2	N-Term(iTRAQ4plex		3.14	61	2	736.38
									VPHDLDMLLR	2	N-Term(iTRAQ4plex); M7(Oxidation			56		
302191647	NRCAM	neuronal cell adhesion molecule isoform D precursor	25.6	0	23	211		133.7								
									SLPSEASEQYLTK	16	N-Term(iTRAQ4plex); K13(iTRAQ4plex		6.31	89	2	870.97
									KIDGDTIIFSNVQER	20	N-Term(iTRAQ4plex); K1(iTRAQ4plex		6.06	80	3	675.04
									AETYEGVYQCTAR	24	N-Term(iTRAQ4plex); C10(Methylthio		5.75	82	2	840.88
									NALGAIHHTISVR	31	N-Term(iTRAQ4plex		5.59	79	2	766.94
									THGMLPGLEPFSHYTLNVR	2	N-Term(iTRAQ4plex		5.43	59	3	771.74
									ERPPTFLTPEGNASNK	2	N-Term(iTRAQ4plex); K16(iTRAQ4plex		5.41	41	3	682.70
									VFNTPEGVPSAPSSLK	15	N-Term(iTRAQ4plex); K16(iTRAQ4plex		5.34	97	2	959.53
									ISWLTNGVPIEIAPDDPSR	2	N-Term(iTRAQ4plex		5.3	56	2	1112.59
									TLQIIHVSEADSGNYQCIAK	2	N-Term(iTRAQ4plex); C17(Methylthio); K20(iTRAQ4plex)		5.21	78	3	842.10
									IDGDTIIFSNVQER	5	N-Term(iTRAQ4plex		5.14	80	2	875.96
									VSQGLNGDLYFSNVLPEDTF	2	N-Term(iTRAQ4plex		5.11	101	2	1184.60
									GKPPPSFSWTR	19	N-Term(iTRAQ4plex); K2(iTRAQ4plex		4.99	59	3	516.62
									SVQLSWTPGDDNNSPITK	6	N-Term(iTRAQ4plex); K18(iTRAQ4plex		4.96	83	2	1124.08
									VQALNDMGFAPEPAVVMGHSGEDLPMV PGNVR	2	N-Term(iTRAQ4plex); M17(Oxidation		4.87	61	3	1189.25

									GSMVSFECK	10	N-Term(iTRAQ4plex); C8(Methylthio); K9(iTRAQ4plex)		4.61	57	2	661.32
									ENIVIQCEAK	12	N-Term(iTRAQ4plex); C7(Methylthio); K10(iTRAQ4plex)		4.35	70	2	740.89
									VMAVNSIGK	4	N-Term(iTRAQ4plex); K9(iTRAQ4plex		4.26	56	2	603.86
									LSPYVNYSFR	17	N-Term(iTRAQ4plex		4.09	64	2	695.37
									YIVSGTPTFVPYLIK	2	N-Term(iTRAQ4plex); K15(iTRAQ4plex		3.56	55	2	993.58
									EDYICYAR	6	N-Term(iTRAQ4plex); C5(Methylthio		3.45	41	2	611.77
									ILTFQGSK	8	N-Term(iTRAQ4plex); K8(iTRAQ4plex		3.44	55	2	591.35
									DPTWIVK	2	N-Term(iTRAQ4plex); K7(iTRAQ4plex			40		
									NEVHLEIK	1	N-Term(iTRAQ4plex); K8(iTRAQ4plex			37		
									VQALNDMGFAPEPAVVMGHSGEDLPMV PGNVR	1	N-Term(iTRAQ4plex			32		
47132555	FN1	fibronectin isoform 4 preproprotein	25.49	0	43	311		256.4								
									RPGGEPSPEGTTGQSYNQYSQR	18	N-Term(iTRAQ4plex		9.81	124	3	847.41
									GLKPGVVYEGQLISIQQYGHQEVTR	2	N-Term(iTRAQ4plex); K3(iTRAQ4plex		7.18	46	4	772.68
									SSPVVIDASTAIDAPSNLR	22	N-Term(iTRAQ4plex		6.66	112	2	1029.05
									GDSPASSKPISINYR	6	N-Term(iTRAQ4plex); K8(iTRAQ4plex		6.38	88	2	940.51
									WCGTTQNYDADQK	9	N-Term(iTRAQ4plex); C2(Methylthio); K13(iTRAQ4plex)		6.24	91	2	932.42
									GATYNIIVEALK	12	N-Term(iTRAQ4plex); K12(iTRAQ4plex		6.03	96	2	790.47
									TETITGFQVDAVPANGQTPIQR	6	N-Term(iTRAQ4plex		5.94	102	2	1244.15
									NLQPASEYTVSLVAIK	6	N-Term(iTRAQ4plex); K16(iTRAQ4plex		5.9	98	2	1011.08
									EESPLLIGQQSTVSDVPR	20	N-Term(iTRAQ4plex		5.76	111	2	1050.06
									GLAFTDVDVDSIK	7	N-Term(iTRAQ4plex); K13(iTRAQ4plex		5.58	85	2	834.46
									GFNCESKPEAEETCFDK	4	N-Term(iTRAQ4plex); C4(Methylthio); K7(iTRAQ4plex); C14(Methylthio); K17(iTRAQ4plex)		5.41	72	3	820.03
									SYTITGLQPGTDYK	14	N-Term(iTRAQ4plex); K14(iTRAQ4plex		5.37	77	2	916.49
									EATIPGHLNSYTIK	7	N-Term(iTRAQ4plex); K14(iTRAQ4plex		5.33	79	3	611.34
									HTSVQTTSSGSGPFTDVR	6	N-Term(iTRAQ4plex		5.31	102	3	670.00
									VDVIPVNLPGEHGQR	31	N-Term(iTRAQ4plex		5.22	71	3	592.00
									TYHVGEQWQK	3	N-Term(iTRAQ4plex); K10(iTRAQ4plex		5.09	48	3	521.95
									DLQFVEVTDVK	8	N-Term(iTRAQ4plex); K11(iTRAQ4plex		4.99	75	2	790.94
									WCHDNGVNYK	2	N-Term(iTRAQ4plex); C2(Methylthio); K10(iTRAQ4plex)		4.96	31	3	523.91
									VPGTSTSATLTGLTR	6	N-Term(iTRAQ4plex		4.88	95	2	803.45
									NTFAEVTGLSPGVTYYFK	4	N-Term(iTRAQ4plex); K18(iTRAQ4plex		4.59	84	2	1141.60
									DDKESVPISDTIIPAVPPPTDLR	4	N-Term(iTRAQ4plex); K3(iTRAQ4plex		4.56	44	3	921.84
									VTDATETTITISWR	11	N-Term(iTRAQ4plex		4.53	82	2	869.45
									GNLLQCICTGNGR	2	N-Term(iTRAQ4plex); C6(Methylthio); C8(Methylthio)		4.53	67	2	792.86
									YEVSVYALK	8	N-Term(iTRAQ4plex); K9(iTRAQ4plex		4.44	64	2	680.39
									IAWESPQGQVSR	3	N-Term(iTRAQ4plex		4.39	62	2	751.40
									VTIMWTPPESAVTGYR	12	N-Term(iTRAQ4plex		4.3	67	2	976.51
									YSFCTDHTVLVQTR	5	N-Term(iTRAQ4plex); C4(Methylthio		4.23	80	2	930.45
									TFYSCTTEGR	5	N-Term(iTRAQ4plex); C5(Methylthio		4.08	48	2	677.80
									FTNIGPDTMR	12	N-Term(iTRAQ4plex		4.04	53	2	648.33
									HYQINQQWER	3	N-Term(iTRAQ4plex		3.95	59	3	515.93
									GEWTCIAYSQLR	4	N-Term(iTRAQ4plex); C5(Methylthio		3.88	45	2	808.89
									IYLYTLNDNAR	7	N-Term(iTRAQ4plex		3.81	60	2	750.40
									ESVPISDTIIPAVPPPTDLR	3	N-Term(iTRAQ4plex		3.79	63	2	1131.13
									CDPHEATCYDDGK	2	N-Term(iTRAQ4plex); C1(Methylthio); C8(Methylthio); K13(iTRAQ4plex)		3.59	61	2	917.36
									TEIDKPSQMQVTDVQDNSISVK	3	N-Term(iTRAQ4plex); K5(iTRAQ4plex); K22(iTRAQ4plex)		3.57	88	3	965.52
									VTIMWTPPESAVTGYR	2	N-Term(iTRAQ4plex); M4(Oxidation		3.55	38	2	984.51
									WLPSSSPVTGYR	11	N-Term(iTRAQ4plex		3.48	53	2	747.40
									ITGYIIK	8	N-Term(iTRAQ4plex); K7(iTRAQ4plex		3.16	40	2	548.35
									IGDTWSK	6	N-Term(iTRAQ4plex); K7(iTRAQ4plex			56		
									LTVGLTR	1	N-Term(iTRAQ4plex			34		
									TYLGNALVCTCYGGSR	1	N-Term(iTRAQ4plex); C9(Methylthio); C11(Methylthio)			31		
59939900	OPCML	opioid-binding protein/cell adhesion molecule isoform b preproprotein	25.44	0	7	26		37.2								
									DQSGEYECSALNDVAAPDVR	3	N-Term(iTRAQ4plex); C8(Methylthio		5.09	100	2	1165.02
									ITVNYPPYISK	16	N-Term(iTRAQ4plex); K11(iTRAQ4plex		4.22	72	2	791.96
									STILYAGNDK	2	N-Term(iTRAQ4plex); K10(iTRAQ4plex		3.96	51	2	685.39
									LATGLDGMR	2	N-Term(iTRAQ4plex		3.43	45	2	539.29
									MSTLTFFNVSEK	1	N-Term(iTRAQ4plex); K12(iTRAQ4plex			58		
									EGQGFVSEDEYLEISDIK	1	N-Term(iTRAQ4plex); K18(iTRAQ4plex			46		
									WSIDPR	1	N-Term(iTRAQ4plex			34		
4503139	CTSB	cathepsin B preproprotein	25.37	7	7	37	0.945	37.8								
									GQDHCGIESEVVAGIPR	9	N-Term(iTRAQ4plex); C5(Methylthio	1.005	4.65	93	3	652.99
									ICEPGYSPTYK	4	N-Term(iTRAQ4plex); C2(Methylthio); K11(iTRAQ4plex)	0.944	4.64	58	2	796.39
									DIMAEIYK	17	N-Term(iTRAQ4plex); K8(iTRAQ4plex	0.901	4.14	55	2	635.85
									VMFTEDLK	2	N-Term(iTRAQ4plex); K8(iTRAQ4plex	1.52	3.79	60	2	635.84
									EQWPQCPTIK	3	N-Term(iTRAQ4plex); C6(Methylthio); K10(iTRAQ4plex)	1.247	3.6	50	2	782.40
									SRPSFHPLSDELVNYVNK	1	N-Term(iTRAQ4plex); K18(iTRAQ4plex	0.954		42		
									HYGYNSYSVSNSEK	1	N-Term(iTRAQ4plex); K14(iTRAQ4plex	0.662		40		
16933542	FN1	fibronectin isoform 3 preproprotein	25.22	0	43	311		259.1								
									RPGGEPSPEGTTGQSYNQYSQR	18	N-Term(iTRAQ4plex		9.81	124	3	847.41
									GLKPGVVYEGQLISIQQYGHQEVTR	2	N-Term(iTRAQ4plex); K3(iTRAQ4plex		7.18	46	4	772.68
									SSPVVIDASTAIDAPSNLR	22	N-Term(iTRAQ4plex		6.66	112	2	1029.05
									GDSPASSKPISINYR	6	N-Term(iTRAQ4plex); K8(iTRAQ4plex		6.38	88	2	940.51
									WCGTTQNYDADQK	9	N-Term(iTRAQ4plex); C2(Methylthio); K13(iTRAQ4plex)		6.24	91	2	932.42
									GATYNIIVEALK	12	N-Term(iTRAQ4plex); K12(iTRAQ4plex		6.03	96	2	790.47
									TETITGFQVDAVPANGQTPIQR	6	N-Term(iTRAQ4plex		5.94	102	2	1244.15
									NLQPASEYTVSLVAIK	6	N-Term(iTRAQ4plex); K16(iTRAQ4plex		5.9	98	2	1011.08
									EESPLLIGQQSTVSDVPR	20	N-Term(iTRAQ4plex		5.76	111	2	1050.06
									GLAFTDVDVDSIK	7	N-Term(iTRAQ4plex); K13(iTRAQ4plex		5.58	85	2	834.46
									GFNCESKPEAEETCFDK	4	N-Term(iTRAQ4plex); C4(Methylthio); K7(iTRAQ4plex); C14(Methylthio); K17(iTRAQ4plex)		5.41	72	3	820.03
									SYTITGLQPGTDYK	14	N-Term(iTRAQ4plex); K14(iTRAQ4plex		5.37	77	2	916.49

									EATIPGHLNSYTIK	7	N-Term(iTRAQ4plex); K14(iTRAQ4plex		5.33	79	3	611.34
									HTSVQTTSSGSGPFTDVF	6	N-Term(iTRAQ4plex		5.31	102	3	670.00
									VDVIPVNLPGEHGQF	31	N-Term(iTRAQ4plex		5.22	71	3	592.00
									TYHVGEQWQK	3	N-Term(iTRAQ4plex); K10(iTRAQ4plex		5.09	48	3	521.95
									DLQFVEVTDVK	8	N-Term(iTRAQ4plex); K11(iTRAQ4plex		4.99	75	2	790.94
									WCHDNGVNYK	2	N-Term(iTRAQ4plex); C2(Methylthio); K10(iTRAQ4plex)		4.96	31	3	523.91
									VPGTSTSATLTGLTF	6	N-Term(iTRAQ4plex		4.88	95	2	803.45
									NTFAEVTGLSPGVTYYFK	4	N-Term(iTRAQ4plex); K18(iTRAQ4plex		4.59	84	2	1141.60
									DDKESVPISDTIIPAVPPPTDLF	4	N-Term(iTRAQ4plex); K3(iTRAQ4plex		4.56	44	3	921.84
									VTDATETTITISWF	11	N-Term(iTRAQ4plex		4.53	82	2	869.45
									GNLLQCICTGNGF	2	N-Term(iTRAQ4plex); C6(Methylthio); C8(Methylthio)		4.53	67	2	792.86
									YEVSVYALK	8	N-Term(iTRAQ4plex); K9(iTRAQ4plex		4.44	64	2	680.39
									IAWESPQGQVSR	3	N-Term(iTRAQ4plex		4.39	62	2	751.40
									VTIMWTPPESAVTGYF	12	N-Term(iTRAQ4plex		4.3	67	2	976.51
									YSFCTDHTVLVQTF	5	N-Term(iTRAQ4plex); C4(Methylthio		4.23	80	2	930.45
									TFYSCTTEGR	5	N-Term(iTRAQ4plex); C5(Methylthio		4.08	48	2	677.80
									FTNIGPDTMR	12	N-Term(iTRAQ4plex		4.04	53	2	648.33
									HYQINQQWER	3	N-Term(iTRAQ4plex		3.95	59	3	515.93
									GEWTCIAYSQLR	4	N-Term(iTRAQ4plex); C5(Methylthio		3.88	45	2	808.89
									IYLYTLNDNAR	7	N-Term(iTRAQ4plex		3.81	60	2	750.40
									ESVPISDTIIPAVPPPTDLF	3	N-Term(iTRAQ4plex		3.79	63	2	1131.13
									CDPHEATCYDDGK	2	N-Term(iTRAQ4plex); C1(Methylthio); C8(Methylthio); K13(iTRAQ4plex)		3.59	61	2	917.36
									TEIDKPSQMQVTDVQDNSISVK	3	N-Term(iTRAQ4plex); K5(iTRAQ4plex); K22(iTRAQ4plex)		3.57	88	3	965.52
									VTIMWTPPESAVTGYF	2	N-Term(iTRAQ4plex); M4(Oxidation		3.55	38	2	984.51
									WLPSSSPVTGYR	11	N-Term(iTRAQ4plex		3.48	53	2	747.40
									ITGYIIK	8	N-Term(iTRAQ4plex); K7(iTRAQ4plex		3.16	40	2	548.35
									IGDTWSK	6	N-Term(iTRAQ4plex); K7(iTRAQ4plex			56		
									LTVGLTR	1	N-Term(iTRAQ4plex			34		
									TYLGNALVCTCYGGSF	1	N-Term(iTRAQ4plex); C9(Methylthio); C11(Methylthio)			31		
5453914	PLTP	phospholipid transfer protein isoform a precursor	25.15	1	10	86	0.784	54.7								
									AGALQLLLVGDK	6	N-Term(iTRAQ4plex); K12(iTRAQ4plex		5.68	98	2	743.47
									DPVASTSNLDMDFF	11	N-Term(iTRAQ4plex		5.33	100	2	856.41
									DPVASTSNLDMDFF	3	N-Term(iTRAQ4plex); M11(Oxidation		4.27	81	2	864.41
									MHAAFGGTFK	6	N-Term(iTRAQ4plex); K10(iTRAQ4plex	0.784	4.14	57	2	677.86
									FLEQELETITIPDLR	2	N-Term(iTRAQ4plex		3.96	42	2	981.04
									SSVDELVGIDYSLMK	7	N-Term(iTRAQ4plex); K15(iTRAQ4plex		3.95	100	2	972.52
									VYDFLSTFITSGMR	4	N-Term(iTRAQ4plex		3.89	72	2	890.96
									VPHDLDMLLR	29	N-Term(iTRAQ4plex		3.86	66	3	451.59
									GAFFPLTER	13	N-Term(iTRAQ4plex		3.58	45	2	591.33
									AVEPQLQEEER	2	N-Term(iTRAQ4plex		3.14	61	2	736.38
									VPHDLDMLLR	2	N-Term(iTRAQ4plex); M7(Oxidation			56		
71274107	MCAM	cell surface glycoprotein MUC18	25.08	11	11	54	0.979	71.6								
									GATLALTQVTPQDEF	12	N-Term(iTRAQ4plex	0.894	5.91	100	2	872.47
									GPVLQLHDLK	16	N-Term(iTRAQ4plex); K10(iTRAQ4plex	1.003	4.9	65	2	704.43
									VWLEVEPVGMLK	4	N-Term(iTRAQ4plex); K12(iTRAQ4plex	0.987	4.74	83	2	844.49
									EVTVPVFYPTEK	8	N-Term(iTRAQ4plex); K12(iTRAQ4plex	0.963	4.47	69	2	848.97
									EDKDAQFYCELNYR	2	N-Term(iTRAQ4plex); K3(iTRAQ4plex); C9(Methylthio)	0.968	3.95	64	3	710.00
									APEEPNIQVNPLGIPVNSK	2	N-Term(iTRAQ4plex); K19(iTRAQ4plex	0.997	3.51	72	2	1152.64
									EAEEETTNDNGVLVLEPAF	3	N-Term(iTRAQ4plex	0.906	3.22	99	2	1115.55
									IFLCQGK	2	N-Term(iTRAQ4plex); C4(Methylthio); K7(iTRAQ4plex)	0.917	3.15	37	2	571.82
									TQLVNVAIFGPPWMAFK	2	N-Term(iTRAQ4plex); K17(iTRAQ4plex	1.235		49		
									VHIQSSQTVESSGLYTLQSILK	2	N-Term(iTRAQ4plex); K22(iTRAQ4plex	1.034		46		
									CLADGNPPPHFSISK	1	N-Term(iTRAQ4plex); C1(Methylthio); K15(iTRAQ4plex)	1.355		38		
4505881	PLG	plasminogen isoform 1 precursor	25.06	13	16	111	1.057	90.5								
									VILGAHQEVNLEPHVQEIEVSF	22	N-Term(iTRAQ4plex	1	8.06	100	3	880.82
									ATTVTGTPCQDWAAQEPHF	15	N-Term(iTRAQ4plex); C9(Methylthio	1.144	6.55	75	3	753.68
									FSPATHPSEGLEENYCF	4	N-Term(iTRAQ4plex); C16(Methylthio	1.099	5.83	50	3	709.65
									EQQCVIMAENF	9	N-Term(iTRAQ4plex); C4(Methylthio		5.04	57	2	755.85
									NPDADKGPWCFTTDPSVF	2	N-Term(iTRAQ4plex); K6(iTRAQ4plex); C10(Methylthio)	0.875	4.81	35	3	780.70
									LSSPAVITDK	19	N-Term(iTRAQ4plex); K10(iTRAQ4plex	1.057	4.47	76	2	659.89
									EAQLPVIENK	2	N-Term(iTRAQ4plex); K10(iTRAQ4plex	1.224	3.85	61	2	714.92
									NPDGDVGGPWCYTTNPF	4	N-Term(iTRAQ4plex); C11(Methylthio	1.082	3.75	48	2	1019.95
									FVTWIEGVMR	7	N-Term(iTRAQ4plex	1.095	3.72	48	2	691.38
									DVVLFEK	17	N-Term(iTRAQ4plex); K7(iTRAQ4plex		3.4	42	2	569.34
									CTTPPPSSGPTYQCLK	1	N-Term(iTRAQ4plex); C1(Methylthio); C14(Methylthio); K16(iTRAQ4plex)	0.939		48		
									TPENFPCK	1	N-Term(iTRAQ4plex); C7(Methylthio); K8(iTRAQ4plex)	1.122		43		
									WEYCNLK	1	N-Term(iTRAQ4plex); C4(Methylthio); K7(iTRAQ4plex)	0.962		42		
									VYLSECK	1	N-Term(iTRAQ4plex); C6(Methylthio); K7(iTRAQ4plex)			36		
									WELCDIPR	5	N-Term(iTRAQ4plex); C4(Methylthio	0.986		35		
4503143	CTSD	cathepsin D preproprotein	25	8	8	78	1.009	44.5								
									TMSEVGGSVEDLIAK	6	N-Term(iTRAQ4plex); K15(iTRAQ4plex	1.378	6.96	139	2	912.49
									DPDAQPGGELMLGGTDSK	2	N-Term(iTRAQ4plex); K18(iTRAQ4plex	1.004	5.16	54	2	1038.52
									LVDQNIFSFYLSR	5	N-Term(iTRAQ4plex	0.873	4.78	78	2	873.47
									VSTLPAITLK	18	N-Term(iTRAQ4plex); K10(iTRAQ4plex	1.056	4.62	70	2	665.93
									ISVNNVLPVFDNLMQQK	5	N-Term(iTRAQ4plex); K17(iTRAQ4plex	0.953	4.45	57	2	1124.13
									FDGILGMAYPR	19	N-Term(iTRAQ4plex	0.988	4.24	59	2	692.36
									LSPEDYTLK	10	N-Term(iTRAQ4plex); K9(iTRAQ4plex	1.078	4.19	63	2	677.38
									QPGITFIAAK	11	N-Term(iTRAQ4plex); K10(iTRAQ4plex	0.906	3.83	59	2	667.41
									FDGILGMAYPR	1	N-Term(iTRAQ4plex); M7(Oxidation	0.992		44		
									TMSEVGGSVEDLIAK	1	N-Term(iTRAQ4plex); M2(Oxidation); K15(iTRAQ4plex)	1.119		40		
20270339	CANT1	soluble calcium-activated nucleotidase 1	24.94	8	8	16	0.876	44.8								
									AQEENTWFSYLK	2	N-Term(iTRAQ4plex); K12(iTRAQ4plex	1.144	5.22	69	2	902.47
									TGVVYQIEGSK	2	N-Term(iTRAQ4plex); K11(iTRAQ4plex	0.906	4.2	50	2	734.92

									IAVIADLDTESF	4	N-Term(iTRAQ4plex	0.819	3.98	53	2	723.90
									AEWLAVK	4	N-Term(iTRAQ4plex); K7(iTRAQ4plex	0.963	3.73	55	2	552.84
									GSVDHENWVSNYNALF	1	N-Term(iTRAQ4plex	0.847		51		
									AVPWVILSDGDGTVEK	1	N-Term(iTRAQ4plex); K16(iTRAQ4plex	0.833		47		
									FLLPETK	1	N-Term(iTRAQ4plex); K7(iTRAQ4plex	0.68		38		
4505505	OPCML	opioid-binding protein/cell adhesion molecule isoform a preproprotein	24.93	0	7	26		38								
									DQSGEYECSALNDVAAPDVF	3	N-Term(iTRAQ4plex); C8(Methylthio		5.09	100	2	1165.02
									ITVNYPPYISK	16	N-Term(iTRAQ4plex); K11(iTRAQ4plex		4.22	72	2	791.96
									STILYAGNDK	2	N-Term(iTRAQ4plex); K10(iTRAQ4plex		3.96	51	2	685.39
									LATGLDGMR	2	N-Term(iTRAQ4plex		3.43	45	2	539.29
									MSTLTFFNVSEK	1	N-Term(iTRAQ4plex); K12(iTRAQ4plex			58		
									EGQGFVSEDEYLEISDIK	1	N-Term(iTRAQ4plex); K18(iTRAQ4plex			46		
									WSIDPR	1	N-Term(iTRAQ4plex			34		
19923362	THY1	thy-1 membrane glycoprotein preproprotein	24.84	4	4	110	0.839	17.9								
									HVLFGTVGVPEHTYF	53	N-Term(iTRAQ4plex	0.836	6.63	84	3	619.34
									VTSLTACLVDQSLF	29	N-Term(iTRAQ4plex); C7(Methylthio	0.87	5.7	90	2	848.45
									KHVLFGTVGVPEHTYF	7	N-Term(iTRAQ4plex); K1(iTRAQ4plex	0.735	5.38	85	4	532.81
									VLYLSAFTSK	21	N-Term(iTRAQ4plex); K10(iTRAQ4plex	0.884	4.32	65	2	708.92
16418467	LRG1	leucine-rich alpha-2-glycoprotein precursor	24.78	6	6	65	0.7	38.2								
									TLDLGENQLETLPPDLLR	12	N-Term(iTRAQ4plex	0.64	6.51	100	2	1091.10
									ENQLEVLEVSWLHGLK	12	N-Term(iTRAQ4plex); K16(iTRAQ4plex	0.632	4.85	75	3	728.08
									DGFDISGNPWICDQNLSDLYR	2	N-Term(iTRAQ4plex); C12(Methylthio	0.677	4.71	109	2	1309.60
									VAAGAFQGLF	15	N-Term(iTRAQ4plex	0.69	4.38	50	2	567.33
									ALGHLDLSGNR	2	N-Term(iTRAQ4plex	0.772	4.07	36	3	432.91
									DLLLPQPDLR	22	N-Term(iTRAQ4plex	0.733	3.44	55	2	662.39
47132553	FN1	fibronectin isoform 5 preproprotein	24.78	0	41	301		252.7								
									RPGGEPSPEGTTGQSYNQYSQF	18	N-Term(iTRAQ4plex		9.81	124	3	847.41
									GLKPGVVYEGQLISIQQYGHQEVTF	2	N-Term(iTRAQ4plex); K3(iTRAQ4plex		7.18	46	4	772.68
									SSPVVIDASTAIDAPSNLF	22	N-Term(iTRAQ4plex		6.66	112	2	1029.05
									GDSPASSKPISINYR	6	N-Term(iTRAQ4plex); K8(iTRAQ4plex		6.38	88	2	940.51
									WCGTTQNYDADQK	9	N-Term(iTRAQ4plex); C2(Methylthio); K13(iTRAQ4plex)		6.24	91	2	932.42
									GATYNIIVEALK	12	N-Term(iTRAQ4plex); K12(iTRAQ4plex		6.03	96	2	790.47
									TETITGFQVDAVPANGQTPIQF	6	N-Term(iTRAQ4plex		5.94	102	2	1244.15
									NLQPASEYTVSLVAIK	6	N-Term(iTRAQ4plex); K16(iTRAQ4plex		5.9	98	2	1011.08
									EESPLLIGQQSTVSDVPR	20	N-Term(iTRAQ4plex		5.76	111	2	1050.06
									GFNCESKPEAEETCFDK	4	N-Term(iTRAQ4plex); C4(Methylthio); K7(iTRAQ4plex); C14(Methylthio); K17(iTRAQ4plex)		5.41	72	3	820.03
									SYTITGLQPGTDYK	14	N-Term(iTRAQ4plex); K14(iTRAQ4plex		5.37	77	2	916.49
									EATIPGHLNSYTIK	7	N-Term(iTRAQ4plex); K14(iTRAQ4plex		5.33	79	3	611.34
									HTSVQTTSSGSGPFTDVF	6	N-Term(iTRAQ4plex		5.31	102	3	670.00
									VDVIPVNLPGEHGQF	31	N-Term(iTRAQ4plex		5.22	71	3	592.00
									TYHVGEQWQK	3	N-Term(iTRAQ4plex); K10(iTRAQ4plex		5.09	48	3	521.95
									DLQFVEVTDVK	8	N-Term(iTRAQ4plex); K11(iTRAQ4plex		4.99	75	2	790.94
									WCHDNGVNYK	2	N-Term(iTRAQ4plex); C2(Methylthio); K10(iTRAQ4plex)		4.96	31	3	523.91
									VPGTSTSATLTGLTF	6	N-Term(iTRAQ4plex		4.88	95	2	803.45
									NTFAEVTGLSPGVTYYFK	4	N-Term(iTRAQ4plex); K18(iTRAQ4plex		4.59	84	2	1141.60
									DDKESVPISDTIIPAVPPPTDLF	4	N-Term(iTRAQ4plex); K3(iTRAQ4plex		4.56	44	3	921.84
									VTDATETTITISWF	11	N-Term(iTRAQ4plex		4.53	82	2	869.45
									GNLLQCICTGNGR	2	N-Term(iTRAQ4plex); C6(Methylthio); C8(Methylthio)		4.53	67	2	792.86
									YEVSVYALK	8	N-Term(iTRAQ4plex); K9(iTRAQ4plex		4.44	64	2	680.39
									VTIMWTPPESAVTGYF	12	N-Term(iTRAQ4plex		4.3	67	2	976.51
									YSFCTDHTVLVQTF	5	N-Term(iTRAQ4plex); C4(Methylthio		4.23	80	2	930.45
									TFYSCTTEGR	5	N-Term(iTRAQ4plex); C5(Methylthio		4.08	48	2	677.80
									FTNIGPDTMR	12	N-Term(iTRAQ4plex		4.04	53	2	648.33
									HYQINQQWER	3	N-Term(iTRAQ4plex		3.95	59	3	515.93
									GEWTCIAYSQLR	4	N-Term(iTRAQ4plex); C5(Methylthio		3.88	45	2	808.89
									IYLYTLNDNAR	7	N-Term(iTRAQ4plex		3.81	60	2	750.40
									ESVPISDTIIPAVPPPTDLF	3	N-Term(iTRAQ4plex		3.79	63	2	1131.13
									CDPHEATCYDDGK	2	N-Term(iTRAQ4plex); C1(Methylthio); C8(Methylthio); K13(iTRAQ4plex)		3.59	61	2	917.36
									TEIDKPSQMQVTDVQDNSISVK	3	N-Term(iTRAQ4plex); K5(iTRAQ4plex); K22(iTRAQ4plex)		3.57	88	3	965.52
									VTIMWTPPESAVTGYF	2	N-Term(iTRAQ4plex); M4(Oxidation		3.55	38	2	984.51
									WLPSSSPVTGYR	11	N-Term(iTRAQ4plex		3.48	53	2	747.40
									ITGYIIK	8	N-Term(iTRAQ4plex); K7(iTRAQ4plex		3.16	40	2	548.35
									IGDTWSK	6	N-Term(iTRAQ4plex); K7(iTRAQ4plex			56		
									LTVGLTR	1	N-Term(iTRAQ4plex			34		
									TYLGNALVCTCYGGSF	1	N-Term(iTRAQ4plex); C9(Methylthio); C11(Methylthio)			31		
295842534	DBI	acyl-CoA-binding protein isoform 6	24.74	0	2	8		11.1								
									TKPSDEEMLFIYGHYK	2	N-Term(iTRAQ4plex); K2(iTRAQ4plex); K16(iTRAQ4plex)		6.37	55	3	797.42
									WDAWNELK	6	N-Term(iTRAQ4plex); K8(iTRAQ4plex		3.93	46	2	675.36
189217853	MMP2	72 kDa type IV collagenase isoform b	24.59	0	12	43		68.8								
									FQGTSYDSCTTEGF	2	N-Term(iTRAQ4plex); C9(Methylthio		5.14	84	2	871.37
									CGNPDVANYNFFPF	2	N-Term(iTRAQ4plex); C1(Methylthio		4.99	68	2	902.41
									WEHGDGYPFDGK	7	N-Term(iTRAQ4plex); K12(iTRAQ4plex		4.74	72	2	848.41
									AFQVWSDVTPLR	8	N-Term(iTRAQ4plex		4.37	62	2	781.93
									IHDGEADIMINFGF	3	N-Term(iTRAQ4plex		4.11	44	2	866.43
									QDIVFDGIAQIR	5	N-Term(iTRAQ4plex		4.09	62	2	759.93
									WCGTTEDYDR	3	N-Term(iTRAQ4plex); C2(Methylthio		3.88	41	2	718.29
									ESCNLFVLK	2	N-Term(iTRAQ4plex); C3(Methylthio); K9(iTRAQ4plex)		3.7	43	2	693.87
									IIGYTPDLDPETVDDAFAF	2	N-Term(iTRAQ4plex		3.68	61	2	1126.57
									GEIFFFK	7	N-Term(iTRAQ4plex); K7(iTRAQ4plex			54		
									FFGLPQTGDLDQNTIETMF	1	N-Term(iTRAQ4plex			38		
									FPFLFNGK	1	N-Term(iTRAQ4plex); K8(iTRAQ4plex			36		
66347875	C1R	complement C1r subcomponent precursor	24.54	13	13	75	0.907	80.1								
									QDACQGDSGGVFAVF	8	N-Term(iTRAQ4plex); C4(Methylthio	0.867	6.08	85	2	850.39
									FCGQLGSPLGNPPGK	4	N-Term(iTRAQ4plex); C2(Methylthio); K15(iTRAQ4plex)	0.824	5.65	65	2	903.47
									MGNFPWQVFTNIHGF	2	N-Term(iTRAQ4plex); M1(Oxidation	1.349	5.17	42	3	655.33
									TLDEFTIIQNLQPQYQFR	3	N-Term(iTRAQ4plex	0.825	5.08	59	2	1199.63

									YTTTMGVNTYK	11	N-Term(iTRAQ4plex); K11(iTRAQ4plex	0.864	5.02	82	2	783.91
									LPVANPQACENWLR	4	N-Term(iTRAQ4plex); C9(Methylthio	0.922	4.94	54	2	900.95
									VLNYVDWIK	15	N-Term(iTRAQ4plex); K9(iTRAQ4plex	0.941	4.47	77	2	719.42
									ESEQGVYTCTAQGIWK	4	N-Term(iTRAQ4plex); C9(Methylthio); K16(iTRAQ4plex)	0.905	4.37	97	2	1067.51
									MGNFPWQVFTNIHGR	9	N-Term(iTRAQ4plex	0.943	3.86	45	3	650.00
									DYFIATCK	6	N-Term(iTRAQ4plex); C7(Methylthio); K8(iTRAQ4plex)	0.799	3.63	49	2	647.82
									NIGEFCGK	4	N-Term(iTRAQ4plex); C6(Methylthio); K8(iTRAQ4plex)	0.898	3.23	54	2	601.29
									QRPPDLDTSSNAVDLLFFTDESGDSR	2	N-Term(iTRAQ4plex	1.105		83		
									YTTTMGVNTYK	1	N-Term(iTRAQ4plex); M5(Oxidation); K11(iTRAQ4plex)	1.112		64		
4501987	AFM	afamin precursor	24.54	14	14	132	1.173	69								
									IAPQLSTEELVSLGEK	13	N-Term(iTRAQ4plex); K16(iTRAQ4plex	1.186	5.98	98	2	1001.57
									ESLLNHFLYEVAR	10	N-Term(iTRAQ4plex	1.198	5.33	87	2	867.97
									FTDSENVCQER	8	N-Term(iTRAQ4plex); C8(Methylthio	1.095	4.61	44	2	759.33
									DADPDTFFAK	21	N-Term(iTRAQ4plex); K10(iTRAQ4plex	1.167	4.55	92	2	707.86
									RPCFESLK	7	N-Term(iTRAQ4plex); C3(Methylthio); K8(iTRAQ4plex)	1.158	4.43	42	3	438.57
									TINPAVDHCCK	2	N-Term(iTRAQ4plex); C9(Methylthio); C10(Methylthio); K11(iTRAQ4plex)	1.173	4.26	73	2	790.87
									SDVGFLPPFPTLDPEEK	4	N-Term(iTRAQ4plex); K17(iTRAQ4plex	1.169	4.12	63	2	1088.57
									ICAMEGLPQK	11	N-Term(iTRAQ4plex); C2(Methylthio); K10(iTRAQ4plex)	1.229	4.05	65	2	712.37
									AIPVTQYLK	12	N-Term(iTRAQ4plex); K9(iTRAQ4plex	0.992	4.04	60	2	660.91
									LPNNVLQEK	10	N-Term(iTRAQ4plex); K9(iTRAQ4plex	1.039	3.73	40	2	671.90
									LCFFYNK	11	N-Term(iTRAQ4plex); C2(Methylthio); K7(iTRAQ4plex)	1.313	3.39	47	2	634.83
									FLVNLVK	21	N-Term(iTRAQ4plex); K7(iTRAQ4plex	1.202	3.32	49	2	560.87
									HPDLSIPELLR	1	N-Term(iTRAQ4plex	1.123		59		
									HVCGALLK	1	N-Term(iTRAQ4plex); C3(Methylthio); K8(iTRAQ4plex)	1.329		49		
18426911	SIRPA	tyrosine-protein phosphatase non-receptor type substrate 1 precursor	24.4	3	8	80	1.191	54.9								
									LTCQVEHDGQPAVSK	8	N-Term(iTRAQ4plex); C3(Methylthio); K15(iTRAQ4plex)		7.14	63	3	649.33
									CTATSLIPVGPIQWFR	2	N-Term(iTRAQ4plex); C1(Methylthio	1.099	4.57	58	2	990.02
									SVLVAAGETATLF	22	N-Term(iTRAQ4plex	1.191	4.51	74	2	716.42
									VPPTLEVTQQPVF	35	N-Term(iTRAQ4plex		4.47	91	3	536.65
									VTTVSDLTK	7	N-Term(iTRAQ4plex); K9(iTRAQ4plex		4.15	66	2	626.37
									ATPQHTVSFTCESHGFSPF	3	N-Term(iTRAQ4plex); C11(Methylthio		3.79	66	3	760.35
									EDVHSQVICEVAHVTLQGDPLF	2	N-Term(iTRAQ4plex); C9(Methylthio		3.53	64	3	879.11
									IGNITPADAGTYYCVK	1	N-Term(iTRAQ4plex); C14(Methylthio); K16(iTRAQ4plex)	1.692		39		
221316762	NTM	neurotrimin isoform 4	24.37	0	6	69		35								
									EQSGDYECSASNDVAAPVVF	8	N-Term(iTRAQ4plex); C8(Methylthio		6.76	101	2	1144.01
									AVGFVSEDEYLEIQGITR	9	N-Term(iTRAQ4plex		5.88	92	2	1085.56
									VHLIVQVSPK	18	N-Term(iTRAQ4plex); K10(iTRAQ4plex		5.09	79	3	469.97
									VTVNYPPYISEAK	29	N-Term(iTRAQ4plex); K13(iTRAQ4plex		4.81	76	2	884.99
									STILYAGNDK	2	N-Term(iTRAQ4plex); K10(iTRAQ4plex		3.96	51	2	685.39
									WCLDPR	3	N-Term(iTRAQ4plex); C2(Methylthio			34		
47777317	CRTAC1	cartilage acidic protein 1 precursor	24.36	12	12	94	0.74	71.4								
									GDGTFVDAAASAGVDDPHQHGI	9	N-Term(iTRAQ4plex	0.853	8.04	98	3	775.37
									NVASGEMNSVLEILYPF	4	N-Term(iTRAQ4plex	0.811	6.23	105	2	1018.54
									DEASSVEVTWPDGK	8	N-Term(iTRAQ4plex); K14(iTRAQ4plex	0.722	5.62	113	2	904.45
									GNQGFNNNWLR	11	N-Term(iTRAQ4plex	0.823	5.59	59	2	732.37
									REHGDPLIEELNPGDALEPEGR	2	N-Term(iTRAQ4plex	0.562	5.23	74	3	863.10
									EHGDPLIEELNPGDALEPEGR	2	N-Term(iTRAQ4plex	0.693	5.08	86	2	1216.10
									WEDILSDEVNVAR	8	N-Term(iTRAQ4plex	0.681	4.92	83	2	845.43
									VDIVYGNWNGPHR	8	N-Term(iTRAQ4plex	0.773	4.9	60	3	557.62
									IIDGGSGYLCEMEPVAHFGLGK	5	N-Term(iTRAQ4plex); C10(Methylthio); K22(iTRAQ4plex)	0.644	4.55	77	3	876.44
									GVASLFAGR	13	N-Term(iTRAQ4plex	0.725	4.08	65	2	511.30
									GVALADFNR	11	N-Term(iTRAQ4plex	0.808	4.03	51	2	553.81
									LVNIAVDER	13	N-Term(iTRAQ4plex	0.695	3.69	43	2	586.84
14916498	OGN	mimecan preproprotein	24.16	8	8	84	0.881	33.9								
									EKETVIIPNEK	11	N-Term(iTRAQ4plex); K2(iTRAQ4plex); K11(iTRAQ4plex)	1.019	4.81	58	2	866.51
									LEGNPIVLGK	19	N-Term(iTRAQ4plex); K10(iTRAQ4plex	0.865	4.5	80	2	664.42
									ETVIIPNEK	23	N-Term(iTRAQ4plex); K9(iTRAQ4plex	0.975	3.82	54	2	665.89
									DFADIPNLR	15	N-Term(iTRAQ4plex	0.87	3.72	47	2	602.83
									RLDFTGNLIEDIEDGTFSK	5	N-Term(iTRAQ4plex); K19(iTRAQ4plex	0.706	3.44	65	3	820.10
									HPNSFICLK	1	N-Term(iTRAQ4plex); C7(Methylthio); K9(iTRAQ4plex)	1.02		55		
									LTLFNAK	4	N-Term(iTRAQ4plex); K7(iTRAQ4plex	0.776		50		
									LPVLPPK	6	N-Term(iTRAQ4plex); K7(iTRAQ4plex	0.651		42		
4504237	SEMA7A	semaphorin-7A isoform 1 preproprotein	24.02	1	11	55	0.903	74.8								
									SVLQSINPAEPHK	10	N-Term(iTRAQ4plex); K13(iTRAQ4plex		6.05	79	3	569.99
									VDFGQTEPHTVLFHEPGSSSVWVGGF	3	N-Term(iTRAQ4plex		5.83	58	4	743.12
									CLPDQQPIPTETFQVADR	5	N-Term(iTRAQ4plex); C1(Methylthio		5.65	92	2	1124.55
									RDCENYITLLER	3	N-Term(iTRAQ4plex); C3(Methylthio	0.903	5.28	75	2	857.93
									AAAIQTMSLDAEF	8	N-Term(iTRAQ4plex		4.95	78	2	760.90
									MQASHGETFHVLYLTTDF	5	N-Term(iTRAQ4plex		4.38	66	3	750.71
									VYLFDFPEGK	10	N-Term(iTRAQ4plex); K10(iTRAQ4plex		4.08	65	2	751.91
									AMLVCSDAATNK	2	N-Term(iTRAQ4plex); C5(Methylthio); K12(iTRAQ4plex)		4.06	87	2	779.39
									VVEPGEQEHSFAFNIMEIQPFF	2	N-Term(iTRAQ4plex		3.91	43	3	916.79
									YYLSCPMESR	2	N-Term(iTRAQ4plex); C5(Methylthio		3.54	44	2	719.82
									WNTFLK	5	N-Term(iTRAQ4plex); K6(iTRAQ4plex			42		
40548389	DKK3	dickkopf-related protein 3 precursor	24	7	7	275	0.947	38.4								
									SAVEEMEAEEAAAK	56	N-Term(iTRAQ4plex); K14(iTRAQ4plex	0.907	6.94	113	2	876.94
									EVEELMEDTQHK	77	N-Term(iTRAQ4plex); K12(iTRAQ4plex	0.933	6.82	96	3	592.63
									EVEELMEDTQHK	27	N-Term(iTRAQ4plex); M6(Oxidation); K12(iTRAQ4plex)	0.911	6.44	76	3	597.96
									EVPDEYEVGSFMEEVR	43	N-Term(iTRAQ4plex	0.914	5.52	87	2	1029.97
									EVPDEYEVGSFMEEVR	9	N-Term(iTRAQ4plex); M12(Oxidation	0.96	5.46	67	3	692.32

									SAVEEMEAEEAAAK	4	N-Term(iTRAQ4plex); M6(Oxidation); K14(iTRAQ4plex)	0.722	5.36	90	2	884.93
									EPAAAAAALLGGEE	12	N-Term(iTRAQ4plex	1.505	4.53	72	2	763.92
									DQDGEILLPR	27	N-Term(iTRAQ4plex	0.947	4.47	65	2	650.35
									SLTEEMALR	18	N-Term(iTRAQ4plex	1.611	4	55	2	597.32
									QELEDLER	2	N-Term(iTRAQ4plex	1.016	3.55	44	2	588.31
195972866	KRT10	keratin, type I cytoskeletal 10	23.97	10	12	69	2.15	58.8								
									GSLGGGFSSGGFSGGSFSR	8	N-Term(iTRAQ4plex	1.059	8.08	120	2	926.44
									ALEESNYELEGK	14	N-Term(iTRAQ4plex); K12(iTRAQ4plex	2.284	6.52	102	2	835.43
									QSLEASLAETEGR	6	N-Term(iTRAQ4plex	2.905	5.29	89	2	767.90
									ELTTEIDNNIEQISSYK	2	N-Term(iTRAQ4plex); K17(iTRAQ4plex	3.044	4.96	91	2	1143.09
									SQYEQLAEQNR	4	N-Term(iTRAQ4plex	1.934	4.51	62	2	755.38
									LENEIQTYR	6	N-Term(iTRAQ4plex	2.207	4.51	44	2	655.35
									VLDELTLTK	12	N-Term(iTRAQ4plex); K9(iTRAQ4plex	2.205	4.25	66	2	660.40
									DAEAWFNEK	9	N-Term(iTRAQ4plex); K9(iTRAQ4plex		4.11	65	2	699.34
									LASYLDK	4	N-Term(iTRAQ4plex); K7(iTRAQ4plex		3.35	53	2	549.32
									AETECQNTEYQQLLDIK	2	N-Term(iTRAQ4plex); C5(Methylthio); K17(iTRAQ4plex)	2.854		58		
									IKEWYEK	1	N-Term(iTRAQ4plex); K2(iTRAQ4plex); K7(iTRAQ4plex)	1.453		42		
221316757	NTM	neurotrimin isoform 3	23.94	1	7	71	0.748	39.2								
									EQSGDYECSASNDVAAPVVF	8	N-Term(iTRAQ4plex); C8(Methylthio		6.76	101	2	1144.01
									AVGFVSEDEYLEIQGITR	9	N-Term(iTRAQ4plex		5.88	92	2	1085.56
									VHLIVQVSPK	18	N-Term(iTRAQ4plex); K10(iTRAQ4plex		5.09	79	3	469.97
									VTVNYPPYISEAK	29	N-Term(iTRAQ4plex); K13(iTRAQ4plex		4.81	76	2	884.99
									STILYAGNDK	2	N-Term(iTRAQ4plex); K10(iTRAQ4plex		3.96	51	2	685.39
									TTALTPWK	2	N-Term(iTRAQ4plex); K8(iTRAQ4plex	0.748	3.24	46	2	603.36
									WCLDPR	3	N-Term(iTRAQ4plex); C2(Methylthio			34		
11342666	MMP2	72 kDa type IV collagenase isoform a preproprotein	23.94	1	13	44	1.034	73.8								
									FQGTSYDSCTTEGR	2	N-Term(iTRAQ4plex); C9(Methylthio		5.14	84	2	871.37
									CGNPDVANYNFFPF	2	N-Term(iTRAQ4plex); C1(Methylthio		4.99	68	2	902.41
									WEHGDGYPFDGK	7	N-Term(iTRAQ4plex); K12(iTRAQ4plex		4.74	72	2	848.41
									AFQVWSDVTPLR	8	N-Term(iTRAQ4plex		4.37	62	2	781.93
									IHDGEADIMINFGR	3	N-Term(iTRAQ4plex		4.11	44	2	866.43
									QDIVFDGIAQIR	5	N-Term(iTRAQ4plex		4.09	62	2	759.93
									WCGTTEDYDR	3	N-Term(iTRAQ4plex); C2(Methylthio		3.88	41	2	718.29
									ESCNLFVLK	2	N-Term(iTRAQ4plex); C3(Methylthio); K9(iTRAQ4plex)		3.7	43	2	693.87
									IIGYTPDLDPETVDDAFAR	2	N-Term(iTRAQ4plex		3.68	61	2	1126.57
									GEIFFFK	7	N-Term(iTRAQ4plex); K7(iTRAQ4plex			54		
									FPGDVAPK	1	N-Term(iTRAQ4plex); K8(iTRAQ4plex	1.034		43		
									FFGLPQTGDLDQNTIETMR	1	N-Term(iTRAQ4plex			38		
									FPFLFNGK	1	N-Term(iTRAQ4plex); K8(iTRAQ4plex			36		
81158226	NRCAM	neuronal cell adhesion molecule isoform A precursor	23.77	0	23	211		143.8								
									SLPSEASEQYLTK	16	N-Term(iTRAQ4plex); K13(iTRAQ4plex		6.31	89	2	870.97
									KIDGDTIIFSNVQER	20	N-Term(iTRAQ4plex); K1(iTRAQ4plex		6.06	80	3	675.04
									AETYEGVYQCTAF	24	N-Term(iTRAQ4plex); C10(Methylthio		5.75	82	2	840.88
									NALGAIHHTISVF	31	N-Term(iTRAQ4plex		5.59	79	2	766.94
									THGMLPGLEPFSHYTLNVF	2	N-Term(iTRAQ4plex		5.43	59	3	771.74
									ERPPTFLTPEGNASNK	2	N-Term(iTRAQ4plex); K16(iTRAQ4plex		5.41	41	3	682.70
									VFNTPEGVPSAPSSLK	15	N-Term(iTRAQ4plex); K16(iTRAQ4plex		5.34	97	2	959.53
									ISWLTNGVPIEIAPDDPSR	2	N-Term(iTRAQ4plex		5.3	56	2	1112.59
									TLQIIHVSEADSGNYQCIAK	2	N-Term(iTRAQ4plex); C17(Methylthio); K20(iTRAQ4plex)		5.21	78	3	842.10
									IDGDTIIFSNVQER	5	N-Term(iTRAQ4plex		5.14	80	2	875.96
									VSQGLNGDLYFSNVLPEDTF	2	N-Term(iTRAQ4plex		5.11	101	2	1184.60
									GKPPPSFSWTR	19	N-Term(iTRAQ4plex); K2(iTRAQ4plex		4.99	59	3	516.62
									SVQLSWTPGDDNNSPITK	6	N-Term(iTRAQ4plex); K18(iTRAQ4plex		4.96	83	2	1124.08
									VQALNDMGFAPEPAVVMGHSGEDLPMV PGNVR	2	N-Term(iTRAQ4plex); M17(Oxidation		4.87	61	3	1189.25
									GSMVSFECK	10	N-Term(iTRAQ4plex); C8(Methylthio); K9(iTRAQ4plex)		4.61	57	2	661.32
									ENIVIQCEAK	12	N-Term(iTRAQ4plex); C7(Methylthio); K10(iTRAQ4plex)		4.35	70	2	740.89
									VMAVNSIGK	4	N-Term(iTRAQ4plex); K9(iTRAQ4plex		4.26	56	2	603.86
									LSPYVNYSFR	17	N-Term(iTRAQ4plex		4.09	64	2	695.37
									YIVSGTPTFVPYLIK	2	N-Term(iTRAQ4plex); K15(iTRAQ4plex		3.56	55	2	993.58
									EDYICYAR	6	N-Term(iTRAQ4plex); C5(Methylthio		3.45	41	2	611.77
									ILTFQGSK	8	N-Term(iTRAQ4plex); K8(iTRAQ4plex		3.44	55	2	591.35
									DPTWIVK	2	N-Term(iTRAQ4plex); K7(iTRAQ4plex			40		
									NEVHLEIK	1	N-Term(iTRAQ4plex); K8(iTRAQ4plex			37		
									VQALNDMGFAPEPAVVMGHSGEDLPMV PGNVR	1	N-Term(iTRAQ4plex			32		
4827022	CNTN2	contactin-2 precursor	23.75	19	19	93	0.994	113.3								
									TTGPGGDGIPAEVHIVF	4	N-Term(iTRAQ4plex	1.161	6.62	89	2	910.50
									RGDGPESLTALVYSAEEEPR	3	N-Term(iTRAQ4plex	0.956	6.22	80	3	774.06
									HFVSQTTGNLYIAF	5	N-Term(iTRAQ4plex	1.047	5.85	87	3	584.32
									HQLVGGNLVIMNPTK	5	N-Term(iTRAQ4plex); K15(iTRAQ4plex	0.915	5.75	81	3	637.04
									RPPGNISWTFSSSSLSIK	5	N-Term(iTRAQ4plex); K18(iTRAQ4plex	1.005	5.38	85	3	751.41
									ETIGDLTILNAQLR	8	N-Term(iTRAQ4plex	1.027	5.29	99	2	850.99
									IIVQAQPEWLK	10	N-Term(iTRAQ4plex); K11(iTRAQ4plex	1.119	4.94	75	2	806.99
									HQLVGGNLVIMNPTK	3	N-Term(iTRAQ4plex); M11(Oxidation); K15(iTRAQ4plex)	0.989	4.72	46	3	642.37
									VISDTEADIGSNLR	6	N-Term(iTRAQ4plex	1.054	4.71	95	2	817.43
									FAQLNLAAEDTF	12	N-Term(iTRAQ4plex	1.082	4.69	71	2	746.90
									VTVTPDGTLIIR	4	N-Term(iTRAQ4plex	0.963	4.01	41	2	714.93
									GGEILIPCQPR	4	N-Term(iTRAQ4plex); C8(Methylthio	0.983	4.01	46	2	686.86
									VEVLAGDLR	4	N-Term(iTRAQ4plex	1.024	3.52	43	2	558.33
									GPPGPPGGVVVR	4	N-Term(iTRAQ4plex	0.936	3.18	67	2	616.87
									YTCFAENFMGK	3	N-Term(iTRAQ4plex); C3(Methylthio); K11(iTRAQ4plex)	1.073	3.13	68	2	822.88
									FGFLQEFSK	3	N-Term(iTRAQ4plex); K9(iTRAQ4plex	0.965		63		
									DIGDTTIQLSWSR	1	N-Term(iTRAQ4plex	0.953		44		
									AVVLWSK	3	N-Term(iTRAQ4plex); K7(iTRAQ4plex	1.032		44		
									LFAPSIK	5	N-Term(iTRAQ4plex); K7(iTRAQ4plex	0.898		43		
									AQDAGVYQCLASNPVGTVVSI	1	N-Term(iTRAQ4plex); C9(Methylthio	0.945		42		

4503155	CTSL1	cathepsin L1 preproprotein	23.72	6	6	23	1.011	37.5								
									VFQEPLFYEAPR	7	N-Term(iTRAQ4plex	1.025	4.83	59	2	820.43
									MIELHNQEYR	2	N-Term(iTRAQ4plex	0.836	3.91	51	3	492.92
									LYGMNEEGWR	8	N-Term(iTRAQ4plex	0.994	3.91	51	2	699.83
									NSWGEEWGMGGYVK	2	N-Term(iTRAQ4plex); K14(iTRAQ4plex	0.881	3.9	65	2	944.45
									HSFTMAMNAFGDMTSEEFF	2	N-Term(iTRAQ4plex	1.64	3.42	46	3	785.01
									LYGMNEEGWR	1	N-Term(iTRAQ4plex); M4(Oxidation	1.045		58		
									NHCGIASAASYPT\	1	N-Term(iTRAQ4plex); C3(Methylthio	0.848		43		
67782338	APLP1	amyloid-like protein 1 isoform 1 precursor	23.66	1	11	173	1.026	72.2								
									QALNEHFQSILQTLEEQVSGEF	24	N-Term(iTRAQ4plex		9.08	99	3	900.80
									FQVHTHLQVIEEF	52	N-Term(iTRAQ4plex		6.41	83	3	594.00
									AALEGFLAALQADPPQAEF	12	N-Term(iTRAQ4plex		5.6	96	2	1056.57
									VEQATQAIPMEF	14	N-Term(iTRAQ4plex		5.33	72	2	758.90
									DADTPMTLPK	23	N-Term(iTRAQ4plex); K10(iTRAQ4plex	1.026	4.64	65	2	688.87
									VEQATQAIPMEF	4	N-Term(iTRAQ4plex); M10(Oxidation		4.42	57	2	766.90
									MNPLEQYER	17	N-Term(iTRAQ4plex		4.25	53	2	662.33
									QMYPELQIAR	5	N-Term(iTRAQ4plex		3.87	55	2	696.88
									MNPLEQYER	3	N-Term(iTRAQ4plex); M1(Oxidation		3.74	32	2	670.33
									VIALINDQR	15	N-Term(iTRAQ4plex		3.59	54	2	593.36
									EWAMADNQSK	2	N-Term(iTRAQ4plex); K10(iTRAQ4plex		3.55	44	2	734.37
									HYQHVAAVDPEK	1	N-Term(iTRAQ4plex); K12(iTRAQ4plex			38		
									HQEAQEACSSQGLILHGSGMLLPCGSDF	1	N-Term(iTRAQ4plex); C8(Methylthio); C24(Methylthio)			32		
115529482	NCAM1	neural cell adhesion molecule 1 isoform 2	23.66	1	17	173	0.894	94.5								
									CVVTGEDGSESEATVNVI	8	N-Term(iTRAQ4plex); C1(Methylthio); K18(iTRAQ4plex)		7.41	101	2	1079.52
									EASMEGIVTIVGLKPETTYAVF	8	N-Term(iTRAQ4plex); K14(iTRAQ4plex		6.49	80	3	884.82
									YIFSDDSSQLTIK	8	N-Term(iTRAQ4plex); K13(iTRAQ4plex		6.37	89	2	902.99
									DKDISWFSPNGEK	7	N-Term(iTRAQ4plex); K2(iTRAQ4plex); K13(iTRAQ4plex)		6.34	59	3	652.35
									LEGQMGEDGNSIK	14	N-Term(iTRAQ4plex); K13(iTRAQ4plex		6.08	87	2	833.42
									GLGEISAASEFK	22	N-Term(iTRAQ4plex); K12(iTRAQ4plex		5.79	86	2	748.92
									ALSSEWKPEIR	14	N-Term(iTRAQ4plex); K7(iTRAQ4plex		5.33	58	3	535.31
									DIQVIVNVPPTIQAF	37	N-Term(iTRAQ4plex		5.18	77	2	904.04
									FFLCQVAGDAK	16	N-Term(iTRAQ4plex); C4(Methylthio); K11(iTRAQ4plex)		4.93	87	2	766.90
									FIVLSNNYLQIR	9	N-Term(iTRAQ4plex		4.78	64	2	812.47
									LPSGSDHVMLK	13	N-Term(iTRAQ4plex); K11(iTRAQ4plex		4.66	52	3	491.28
									QETLDGHMVVF	2	N-Term(iTRAQ4plex	0.894	4.41	64	2	714.88
									LPSGSDHVMLK	2	N-Term(iTRAQ4plex); M9(Oxidation); K11(iTRAQ4plex)		4.28	38	3	496.61
									NDEAEYICIAENK	5	N-Term(iTRAQ4plex); C8(Methylthio); K13(iTRAQ4plex)		4.12	82	2	923.44
									DISWFSPNGEK	4	N-Term(iTRAQ4plex); K11(iTRAQ4plex		3.93	54	2	784.40
									VSSLTLK	1	N-Term(iTRAQ4plex); K7(iTRAQ4plex			43		
									GEINFK	2	N-Term(iTRAQ4plex); K6(iTRAQ4plex			43		
17136078	VGF	neurosecretory protein VGF precursor	23.58	10	10	96	0.761	67.2								
									VGEEDEEAAEAEAEAEEAEF	52	N-Term(iTRAQ4plex	0.723	8.43	107	2	1154.00
									NSEPQDEGELFQGVDPR	12	N-Term(iTRAQ4plex	0.815	5.58	66	2	1030.99
									MPDSGPLPETHK	10	N-Term(iTRAQ4plex); K12(iTRAQ4plex	0.858	5.45	59	3	532.95
									QNALLFAEEEDGEAGAEDKF	2	N-Term(iTRAQ4plex); K19(iTRAQ4plex	0.968	5.35	88	3	827.41
									LLQQGLAQVEAGR	6	N-Term(iTRAQ4plex	0.865	5	75	2	763.94
									AYQGVAAPFPK	2	N-Term(iTRAQ4plex); K11(iTRAQ4plex	0.87	4.48	48	2	718.91
									EPVAGDAVPGPK	4	N-Term(iTRAQ4plex); K12(iTRAQ4plex	0.753	4.05	55	2	712.90
									NAPPEPVPPPR	6	N-Term(iTRAQ4plex	0.703	3.35	65	2	657.87
									RPESALLGGSEAGER	1	N-Term(iTRAQ4plex	1.098		49		
									APLPPPAPSQFQAR	1	N-Term(iTRAQ4plex	0.671		39		
4885065	APLP1	amyloid-like protein 1 isoform 2 precursor	23.54	1	11	167	0.906	72.1								
									QALNEHFQSILQTLEEQVSGEF	24	N-Term(iTRAQ4plex		9.08	99	3	900.80
									FQVHTHLQVIEEF	52	N-Term(iTRAQ4plex		6.41	83	3	594.00
									AALEGFLAALQADPPQAEF	12	N-Term(iTRAQ4plex		5.6	96	2	1056.57
									VEQATQAIPMEF	14	N-Term(iTRAQ4plex		5.33	72	2	758.90
									VEQATQAIPMEF	4	N-Term(iTRAQ4plex); M10(Oxidation		4.42	57	2	766.90
									MNPLEQYER	17	N-Term(iTRAQ4plex		4.25	53	2	662.33
									QMYPELQIAR	5	N-Term(iTRAQ4plex		3.87	55	2	696.88
									DDTPMTLPK	17	N-Term(iTRAQ4plex); K9(iTRAQ4plex	0.906	3.84	54	2	653.35
									MNPLEQYER	3	N-Term(iTRAQ4plex); M1(Oxidation		3.74	32	2	670.33
									VIALINDQR	15	N-Term(iTRAQ4plex		3.59	54	2	593.36
									EWAMADNQSK	2	N-Term(iTRAQ4plex); K10(iTRAQ4plex		3.55	44	2	734.37
									HYQHVAAVDPEK	1	N-Term(iTRAQ4plex); K12(iTRAQ4plex			38		
									HQEAQEACSSQGLILHGSGMLLPCGSDF	1	N-Term(iTRAQ4plex); C8(Methylthio); C24(Methylthio)			32		
57242755	CLSTN1	calsyntenin-1 isoform 2	23.48	1	20	179	0.732	108.6								
									ATVHIQVNDVNEYAPVFI	25	N-Term(iTRAQ4plex); K18(iTRAQ4plex		7.63	83	3	778.09
									ISIKPTCTPGWQGWNNR	5	N-Term(iTRAQ4plex); K4(iTRAQ4plex); C7(Methylthio)		7.63	55	3	764.73
									AASEFESSEGVFLFPELR	19	N-Term(iTRAQ4plex		6.19	93	2	1080.05
									DYSFTIQAYDCGK	8	N-Term(iTRAQ4plex); C11(Methylthio); K13(iTRAQ4plex)		5.67	95	2	922.93
									IHGQNVPFDAVVVDI	19	N-Term(iTRAQ4plex); K15(iTRAQ4plex		5.55	84	2	963.54
									EGLDLQVLEDSGR	14	N-Term(iTRAQ4plex		5.15	84	2	787.91
									EKLDCELQK	7	N-Term(iTRAQ4plex); K2(iTRAQ4plex); C5(Methylthio); K9(iTRAQ4plex)		4.62	48	3	528.63
									LTVTAYDCGK	14	N-Term(iTRAQ4plex); C8(Methylthio); K10(iTRAQ4plex)		4.41	69	2	702.86
									IPDGVVSVSPK	2	N-Term(iTRAQ4plex); K11(iTRAQ4plex		4.39	77	2	693.42
									VIDCLYTCK	15	N-Term(iTRAQ4plex); C4(Methylthio); C8(Methylthio); K9(iTRAQ4plex)		4.03	58	2	719.35
									GVQIQAHPSQLVLTLEGEDLGELDK	3	N-Term(iTRAQ4plex); K25(iTRAQ4plex		3.85	67	3	993.21
									FAGEICGFK	25	N-Term(iTRAQ4plex); C6(Methylthio); K9(iTRAQ4plex)	0.732	3.8	65	2	653.33
									ETILCSSDK	4	N-Term(iTRAQ4plex); C5(Methylthio); K9(iTRAQ4plex)		3.79	52	2	665.34
									ATEDVLVK	2	N-Term(iTRAQ4plex); K8(iTRAQ4plex		3.77	50	2	581.85
									EPFTISVWMR	4	N-Term(iTRAQ4plex		3.73	53	2	705.37
									GNLAGLTLR	3	N-Term(iTRAQ4plex		3.58	52	2	529.83
									LDCELQK	3	N-Term(iTRAQ4plex); C3(Methylthio); K7(iTRAQ4plex)		3.14	40	2	591.81

									LICSELNGR	3	N-Term(iTRAQ4plex); C3(Methylthio			47		
57242757	CLSTN1	calsyntenin-1 isoform 1	23.45	1	20	156	0.885	109.7								
									ATVHIQVNDVNEYAPVFI	25	N-Term(iTRAQ4plex); K18(iTRAQ4plex		7.63	83	3	778.09
									ISIKPTCTPGWQGWNNR	5	N-Term(iTRAQ4plex); K4(iTRAQ4plex); C7(Methylthio)		7.63	55	3	764.73
									AASEFESSEGVFLFPELR	19	N-Term(iTRAQ4plex		6.19	93	2	1080.05
									DYSFTIQAYDCGK	8	N-Term(iTRAQ4plex); C11(Methylthio); K13(iTRAQ4plex)		5.67	95	2	922.93
									IHGQNVPFDAVVVDK	19	N-Term(iTRAQ4plex); K15(iTRAQ4plex		5.55	84	2	963.54
									EGLDLQVLEDSGR	14	N-Term(iTRAQ4plex		5.15	84	2	787.91
									EKLDCELQK	7	N-Term(iTRAQ4plex); K2(iTRAQ4plex); C5(Methylthio); K9(iTRAQ4plex)		4.62	48	3	528.63
									LTVTAYDCGK	14	N-Term(iTRAQ4plex); C8(Methylthio); K10(iTRAQ4plex)		4.41	69	2	702.86
									IPDGVVSVSPK	2	N-Term(iTRAQ4plex); K11(iTRAQ4plex		4.39	77	2	693.42
									VIDCLYTCK	15	N-Term(iTRAQ4plex); C4(Methylthio); C8(Methylthio); K9(iTRAQ4plex)		4.03	58	2	719.35
									GVQIQAHPSQLVLTLEGEDLGELDK	3	N-Term(iTRAQ4plex); K25(iTRAQ4plex		3.85	67	3	993.21
									ETILCSSDK	4	N-Term(iTRAQ4plex); C5(Methylthio); K9(iTRAQ4plex)		3.79	52	2	665.34
									ATEDVLVK	2	N-Term(iTRAQ4plex); K8(iTRAQ4plex		3.77	50	2	581.85
									EPFTISVWMR	4	N-Term(iTRAQ4plex		3.73	53	2	705.37
									GNLAGLTLR	3	N-Term(iTRAQ4plex		3.58	52	2	529.83
									FAESFEVTVTK	2	N-Term(iTRAQ4plex); K11(iTRAQ4plex	0.885	3.22	82	2	773.43
									LDCELQK	3	N-Term(iTRAQ4plex); C3(Methylthio); K7(iTRAQ4plex)		3.14	40	2	591.81
									LICSELNGR	3	N-Term(iTRAQ4plex); C3(Methylthio			47		
194018472	SERPINA5	plasma serine protease inhibitor	23.4	9	9	16	0.949	45.6								
									GTQEQDFYVTSETVVF	2	N-Term(iTRAQ4plex	0.83	6.98	67	2	1002.00
									GFQQLLQELNQPR	4	N-Term(iTRAQ4plex	0.953	5.29	73	2	857.98
									FSIEGSYQLEK	2	N-Term(iTRAQ4plex); K11(iTRAQ4plex	0.983	4.95	62	2	794.93
									AAAATGTIFTFF	2	N-Term(iTRAQ4plex	0.87	3.87	59	2	685.88
									TLYLADTFPTNFR	2	N-Term(iTRAQ4plex	0.885	3.67	48	2	851.95
									RDFTFDLYR	1	N-Term(iTRAQ4plex	1.118		44		
									MQQVENGLSEK	1	N-Term(iTRAQ4plex); K11(iTRAQ4plex	1.16		44		
									DFTFDLYR	1	N-Term(iTRAQ4plex	0.916		42		
									EDQYHYLLDR	1	N-Term(iTRAQ4plex	1.018		34		
4557032	LDHB	L-lactate dehydrogenase B chain	23.35	7	7	18	0.487	36.6								
									GEMMDLQHGSLFLQTPK	2	N-Term(iTRAQ4plex); K17(iTRAQ4plex	0.515	5.8	50	3	740.72
									SADTLWDIQK	2	N-Term(iTRAQ4plex); K10(iTRAQ4plex	0.494	4.39	54	2	732.90
									GLTSVINQK	2	N-Term(iTRAQ4plex); K9(iTRAQ4plex	0.728	4.23	47	2	624.39
									IVADKDYSVTANSK	2	N-Term(iTRAQ4plex); K5(iTRAQ4plex); K14(iTRAQ4plex)	0.413	4.21	58	3	648.37
									MVVESAYEVIK	2	N-Term(iTRAQ4plex); M1(Oxidation); K11(iTRAQ4plex)	0.492	3.8	87	2	786.44
									IVVVTAGVF	3	N-Term(iTRAQ4plex	0.494	3.18	41	2	529.35
									FIIPQIVK	5	N-Term(iTRAQ4plex); K8(iTRAQ4plex	0.427		54		
39995109	GM2A	ganglioside GM2 activator isoform 1 precursor	23.32	2	4	46	0.939	20.8								
									IESVLSSSGK	19	N-Term(iTRAQ4plex); K10(iTRAQ4plex	0.99	4.59	72	2	647.88
									SEFVVPDLELPSWLTTGNYR	3	N-Term(iTRAQ4plex	0.695	4.22	50	2	1234.14
									EVAGLWIK	16	N-Term(iTRAQ4plex); K8(iTRAQ4plex		4.17	54	2	602.37
									VDLVLEK	8	N-Term(iTRAQ4plex); K7(iTRAQ4plex		3.33	43	2	552.35
115529478	NCAM1	neural cell adhesion molecule 1 isoform 3	23.26	0	15	170		83.7								
									CVVTGEDGSESEATVNVK	8	N-Term(iTRAQ4plex); C1(Methylthio); K18(iTRAQ4plex)		7.41	101	2	1079.52
									EASMEGIVTIVGLKPETTYAVF	8	N-Term(iTRAQ4plex); K14(iTRAQ4plex		6.49	80	3	884.82
									YIFSDDSSQLTIK	8	N-Term(iTRAQ4plex); K13(iTRAQ4plex		6.37	89	2	902.99
									DKDISWFSPNGEK	7	N-Term(iTRAQ4plex); K2(iTRAQ4plex); K13(iTRAQ4plex)		6.34	59	3	652.35
									LEGQMGEDGNSIK	14	N-Term(iTRAQ4plex); K13(iTRAQ4plex		6.08	87	2	833.42
									GLGEISAASEFK	22	N-Term(iTRAQ4plex); K12(iTRAQ4plex		5.79	86	2	748.92
									ALSSEWKPEIR	14	N-Term(iTRAQ4plex); K7(iTRAQ4plex		5.33	58	3	535.31
									DIQVIVNVPPTIQAF	37	N-Term(iTRAQ4plex		5.18	77	2	904.04
									FFLCQVAGDAK	16	N-Term(iTRAQ4plex); C4(Methylthio); K11(iTRAQ4plex)		4.93	87	2	766.90
									FIVLSNNYLQIR	9	N-Term(iTRAQ4plex		4.78	64	2	812.47
									LPSGSDHVMLK	13	N-Term(iTRAQ4plex); K11(iTRAQ4plex		4.66	52	3	491.28
									LPSGSDHVMLK	2	N-Term(iTRAQ4plex); M9(Oxidation); K11(iTRAQ4plex)		4.28	38	3	496.61
									NDEAEYICIAENK	5	N-Term(iTRAQ4plex); C8(Methylthio); K13(iTRAQ4plex)		4.12	82	2	923.44
									DISWFSPNGEK	4	N-Term(iTRAQ4plex); K11(iTRAQ4plex		3.93	54	2	784.40
									VSSLTLK	1	N-Term(iTRAQ4plex); K7(iTRAQ4plex			43		
									GEINFK	2	N-Term(iTRAQ4plex); K6(iTRAQ4plex			43		
156523970	AHSG	alpha-2-HS-glycoprotein	23.16	7	7	185	1.416	39.3								
									TVVQPSVGAAAGPVVPPCPGI	20	N-Term(iTRAQ4plex); C18(Methylthio	1.473	7.04	89	2	1075.07
									HTFMGVVSLGSPSGEVSHPF	13	N-Term(iTRAQ4plex); M4(Oxidation	1.496	6.8	87	3	747.71
									EHAVEGDCDFQLLK	65	N-Term(iTRAQ4plex); C8(Methylthio); K14(iTRAQ4plex)	1.347	6.56	89	2	969.47
									HTFMGVVSLGSPSGEVSHPF	39	N-Term(iTRAQ4plex	1.416	6.49	92	3	742.38
									HTLNQIDEVK	5	N-Term(iTRAQ4plex); K10(iTRAQ4plex	1.641	4.75	81	2	742.92
									CNLLAEK	16	N-Term(iTRAQ4plex); C1(Methylthio); K7(iTRAQ4plex)	1.442	3.32	43	2	562.81
									FSVVYAK	25	N-Term(iTRAQ4plex); K7(iTRAQ4plex	1.41	3.14	56	2	551.32
									QYGFCK	2	N-Term(iTRAQ4plex); C5(Methylthio); K6(iTRAQ4plex)	1.062		30		
295849279	DBI	acyl-CoA-binding protein isoform 1	23.08	0	2	8		11.8								
									TKPSDEEMLFIYGHYK	2	N-Term(iTRAQ4plex); K2(iTRAQ4plex); K16(iTRAQ4plex)		6.37	55	3	797.42
									WDAWNELK	6	N-Term(iTRAQ4plex); K8(iTRAQ4plex		3.93	46	2	675.36
225543438	C2	complement C2 isoform 2 preproprotein	23.06	0	12	57		69.4								
									RNDYLDIYAIGVGK	2	N-Term(iTRAQ4plex); K14(iTRAQ4plex		5.35	60	2	943.02
									CSSNLVLTGSSER	9	N-Term(iTRAQ4plex); C1(Methylthio		4.78	83	2	771.87
									KNQGILEFYGDDIALLK	2	N-Term(iTRAQ4plex); K1(iTRAQ4plex); K17(iTRAQ4plex)		4.74	70	3	790.46
									GESGGAVFLER	2	N-Term(iTRAQ4plex		4.67	60	2	633.33
									AVISPGFDVFAK	6	N-Term(iTRAQ4plex); K12(iTRAQ4plex		4.33	101	2	769.94
									VLMSVLNDNSF	10	N-Term(iTRAQ4plex		4.05	64	2	696.38

									HAIILLTDGK	4	N-Term(iTRAQ4plex); K10(iTRAQ4plex		3.99	49	2	684.93
									ALHQVFEHMLDVSK	8	N-Term(iTRAQ4plex); K14(iTRAQ4plex		3.9	68	3	648.02
									DMTEVISSLENANYK	2	N-Term(iTRAQ4plex); K15(iTRAQ4plex		3.8	73	2	1001.51
									EILNINQK	6	N-Term(iTRAQ4plex); K8(iTRAQ4plex		3.66	46	2	630.38
									QHLGDVLNFLPL	4	N-Term(iTRAQ4plex		3.5	73	2	755.43
									LNINLK	2	N-Term(iTRAQ4plex); K6(iTRAQ4plex			41		
47132557	FN1	fibronectin isoform 1 preproprotein	23.05	0	41	304		272.2								
									RPGGEPSPEGTTGQSYNQYSQR	18	N-Term(iTRAQ4plex		9.81	124	3	847.41
									GLKPGVVYEGQLISIQQYGHQEVTR	2	N-Term(iTRAQ4plex); K3(iTRAQ4plex		7.18	46	4	772.68
									SSPVVIDASTAIDAPSNLR	22	N-Term(iTRAQ4plex		6.66	112	2	1029.05
									GDSPASSKPISINYR	6	N-Term(iTRAQ4plex); K8(iTRAQ4plex		6.38	88	2	940.51
									WCGTTQNYDADQK	9	N-Term(iTRAQ4plex); C2(Methylthio); K13(iTRAQ4plex)		6.24	91	2	932.42
									GATYNIIVEALK	12	N-Term(iTRAQ4plex); K12(iTRAQ4plex		6.03	96	2	790.47
									TETITGFQVDAVPANGQTPIQR	6	N-Term(iTRAQ4plex		5.94	102	2	1244.15
									NLQPASEYTVSLVAIK	6	N-Term(iTRAQ4plex); K16(iTRAQ4plex		5.9	98	2	1011.08
									EESPLLIGQQSTVSDVPR	20	N-Term(iTRAQ4plex		5.76	111	2	1050.06
									GLAFTDVDVDSIK	7	N-Term(iTRAQ4plex); K13(iTRAQ4plex		5.58	85	2	834.46
									GFNCESKPEAEETCFDK	4	N-Term(iTRAQ4plex); C4(Methylthio); K7(iTRAQ4plex); C14(Methylthio); K17(iTRAQ4plex)		5.41	72	3	820.03
									SYTITGLQPGTDYK	14	N-Term(iTRAQ4plex); K14(iTRAQ4plex		5.37	77	2	916.49
									EATIPGHLNSYTIK	7	N-Term(iTRAQ4plex); K14(iTRAQ4plex		5.33	79	3	611.34
									HTSVQTTSSGSGPFTDVR	6	N-Term(iTRAQ4plex		5.31	102	3	670.00
									VDVIPVNLPGEHGQR	31	N-Term(iTRAQ4plex		5.22	71	3	592.00
									TYHVGEQWQK	3	N-Term(iTRAQ4plex); K10(iTRAQ4plex		5.09	48	3	521.95
									DLQFVEVTDVK	8	N-Term(iTRAQ4plex); K11(iTRAQ4plex		4.99	75	2	790.94
									WCHDNGVNYK	2	N-Term(iTRAQ4plex); C2(Methylthio); K10(iTRAQ4plex)		4.96	31	3	523.91
									VPGTSTSATLTGLTR	6	N-Term(iTRAQ4plex		4.88	95	2	803.45
									NTFAEVTGLSPGVTYYFK	4	N-Term(iTRAQ4plex); K18(iTRAQ4plex		4.59	84	2	1141.60
									VTDATETTITISWR	11	N-Term(iTRAQ4plex		4.53	82	2	869.45
									GNLLQCICTGNGR	2	N-Term(iTRAQ4plex); C6(Methylthio); C8(Methylthio)		4.53	67	2	792.86
									YEVSVYALK	8	N-Term(iTRAQ4plex); K9(iTRAQ4plex		4.44	64	2	680.39
									IAWESPQGQVSR	3	N-Term(iTRAQ4plex		4.39	62	2	751.40
									VTIMWTPPESAVTGYR	12	N-Term(iTRAQ4plex		4.3	67	2	976.51
									YSFCTDHTVLVQTR	5	N-Term(iTRAQ4plex); C4(Methylthio		4.23	80	2	930.45
									TFYSCTTEGR	5	N-Term(iTRAQ4plex); C5(Methylthio		4.08	48	2	677.80
									FTNIGPDTMR	12	N-Term(iTRAQ4plex		4.04	53	2	648.33
									HYQINQQWER	3	N-Term(iTRAQ4plex		3.95	59	3	515.93
									GEWTCIAYSQLR	4	N-Term(iTRAQ4plex); C5(Methylthio		3.88	45	2	808.89
									IYLYTLNDNAR	7	N-Term(iTRAQ4plex		3.81	60	2	750.40
									CDPHEATCYDDGK	2	N-Term(iTRAQ4plex); C1(Methylthio); C8(Methylthio); K13(iTRAQ4plex)		3.59	61	2	917.36
									TEIDKPSQMQVTDVQDNSISVK	3	N-Term(iTRAQ4plex); K5(iTRAQ4plex); K22(iTRAQ4plex)		3.57	88	3	965.52
									VTIMWTPPESAVTGYR	2	N-Term(iTRAQ4plex); M4(Oxidation		3.55	38	2	984.51
									WLPSSSPVTGYR	11	N-Term(iTRAQ4plex		3.48	53	2	747.40
									ITGYIIK	8	N-Term(iTRAQ4plex); K7(iTRAQ4plex		3.16	40	2	548.35
									IGDTWSK	6	N-Term(iTRAQ4plex); K7(iTRAQ4plex			56		
									LTVGLTR	1	N-Term(iTRAQ4plex			34		
									TYLGNALVCTCYGGSR	1	N-Term(iTRAQ4plex); C9(Methylthio); C11(Methylthio)			31		
7661714	FAM3C	family with sequence similarity 3, member C precursor	22.91	4	4	9	0.773	24.7								
									ICLEDNVLMSGVK	2	N-Term(iTRAQ4plex); C2(Methylthio); K13(iTRAQ4plex)	0.872	6	102	2	877.96
									MASGAANVVGPK	2	N-Term(iTRAQ4plex); K12(iTRAQ4plex	1.039	3.27	89	2	695.40
									LIADLGSTSITNLGFR	4	N-Term(iTRAQ4plex	0.661	3.26	69	2	911.52
									MDASLGNLFAR	1	N-Term(iTRAQ4plex			37		
71067336	CD99L2	CD99 antigen-like protein 2 isoform 1 precursor	22.9	0	3	12		28								
									DLEDIVGGGEYKPDK	6	N-Term(iTRAQ4plex); K12(iTRAQ4plex); K15(iTRAQ4plex)		5.04	41	3	689.71
									APAKPPGSGLDLADALDDQDDGR	5	N-Term(iTRAQ4plex); K4(iTRAQ4plex		4.14	78	3	861.44
									APANTLGNDFDLADALDDRNDR	1	N-Term(iTRAQ4plex			39		
34734066	FBLN1	fibulin-1 isoform D precursor	22.9	6	12	106	0.875	77.2								
									SQETGDLDVGGLQETDK	9	N-Term(iTRAQ4plex); K17(iTRAQ4plex		6.58	93	2	1040.52
									IIEVEEEQEDPYLNDR	24	N-Term(iTRAQ4plex		6.37	91	3	712.35
									LEMNYVVGGVVSHR	15	N-Term(iTRAQ4plex	0.908	5.13	83	3	568.64
									AITPPHPASQANIIFDITEGNLR	3	N-Term(iTRAQ4plex	0.66	5.02	74	3	873.81
									EFTRPEEIIFLR	10	N-Term(iTRAQ4plex	0.849	4.92	49	3	565.32
									CVDVDECAPPAEPCGK	2	N-Term(iTRAQ4plex); C1(Methylthio); C7(Methylthio); C14(Methylthio); K16(iTRAQ4plex)		4.79	65	2	1029.92
									LEMNYVVGGVVSHR	4	N-Term(iTRAQ4plex); M3(Oxidation	0.871	4.34	53	3	573.97
									TGYYFDGISR	12	N-Term(iTRAQ4plex		4.28	44	2	661.83
									YMDGMTVGVVR	8	N-Term(iTRAQ4plex	0.903	4.24	56	2	686.35
									DCSLPYATESK	4	N-Term(iTRAQ4plex); C2(Methylthio); K11(iTRAQ4plex)		4.16	66	2	774.37
									MCVDVNECQR	9	N-Term(iTRAQ4plex); C2(Methylthio); C8(Methylthio)		3.69	55	2	716.79
									DSFDIIK	3	N-Term(iTRAQ4plex); K7(iTRAQ4plex	1.049	3.23	39	2	563.32
									YMDGMTVGVVR	2	N-Term(iTRAQ4plex); M5(Oxidation	0.848	3.18	32	2	694.35
45594240	LSAMP	limbic system-associated membrane protein preproprotein	22.78	5	5	92	0.892	37.4								
									VDVYDEGSYTCSVQTQHEPK	22	N-Term(iTRAQ4plex); C11(Methylthio); K20(iTRAQ4plex)	0.925	9.35	107	3	873.74
									EFEGEEEYLEILGITR	16	N-Term(iTRAQ4plex	0.972	7.2	92	2	1036.03
									CEASAVPAPDFEWYR	11	N-Term(iTRAQ4plex); C1(Methylthio	0.875	4.77	91	2	965.93
									VTVNYPPTITESK	33	N-Term(iTRAQ4plex); K13(iTRAQ4plex	0.883	4.52	100	2	868.99
									TSQVYLIVQVPPK	10	N-Term(iTRAQ4plex); K13(iTRAQ4plex	0.917	4.11	59	2	880.53
5729770	TPP1	tripeptidyl-peptidase 1 preproprotein	22.74	8	8	27	0.914	61.2								
									LYQQHGAGLFDVTR	6	N-Term(iTRAQ4plex	0.901	6.04	77	3	583.64
									LFGGNFAHQASVAR	5	N-Term(iTRAQ4plex	1.029	5.89	90	3	540.29
									QAELLLPGAEFHHYVGGPTETHVVR	4	N-Term(iTRAQ4plex	0.977	5.23	49	5	581.11
									WLLAAGAQK	5	N-Term(iTRAQ4plex); K9(iTRAQ4plex	1.156	3.84	45	2	623.39

									AYPDVAALSDGYWVVSNF	3	N-Term(iTRAQ4plex	0.669	3.23	44	2	1064.04
									TLPPGWVSLGR	2	N-Term(iTRAQ4plex	0.834	3.15	41	2	663.89
									SPHPYQLPQALAPHVDFVGGLHF	1	N-Term(iTRAQ4plex			33		
225637520	SEMA7A	semaphorin-7A isoform 2 preproprotein	22.7	0	10	52		73.3								
									SVLQSINPAEPHK	10	N-Term(iTRAQ4plex); K13(iTRAQ4plex		6.05	79	3	569.99
									VDFGQTEPHTVLFHEPGSSSVWVGGF	3	N-Term(iTRAQ4plex		5.83	58	4	743.12
									CLPDQQPIPTETFQVADF	5	N-Term(iTRAQ4plex); C1(Methylthio		5.65	92	2	1124.55
									AAAIQTMSLDAEF	8	N-Term(iTRAQ4plex		4.95	78	2	760.90
									MQASHGETFHVLYLTTDF	5	N-Term(iTRAQ4plex		4.38	66	3	750.71
									VYLFDFPEGK	10	N-Term(iTRAQ4plex); K10(iTRAQ4plex		4.08	65	2	751.91
									AMLVCSDAATNK	2	N-Term(iTRAQ4plex); C5(Methylthio); K12(iTRAQ4plex)		4.06	87	2	779.39
									VVEPGEQEHSFAFNIMEIQPFF	2	N-Term(iTRAQ4plex		3.91	43	3	916.79
									YYLSCPMESR	2	N-Term(iTRAQ4plex); C5(Methylthio		3.54	44	2	719.82
									WNTFLK	5	N-Term(iTRAQ4plex); K6(iTRAQ4plex			42		
21686977	CADM4	cell adhesion molecule 4 precursor	22.68	7	7	54	0.913	42.8								
									LHQYDGSIVVIQNPAF	16	N-Term(iTRAQ4plex	0.957	6.54	108	2	977.53
									QTQYVLDVQYSPTAF	6	N-Term(iTRAQ4plex	1	5.77	91	2	957.00
									EQAVEGGEVELSCLVPF	5	N-Term(iTRAQ4plex); C13(Methylthio	0.92	4.34	95	2	1003.00
									DDGGIIICEAQNQALPSGHSK	2	N-Term(iTRAQ4plex); C8(Methylthio); K21(iTRAQ4plex)	1.206	4.07	62	3	829.75
									FQLEEFSPR	20	N-Term(iTRAQ4plex	0.899	3.96	53	2	648.84
									VWSVASTVR	4	N-Term(iTRAQ4plex	0.874	3.56	52	2	574.83
									KDDGGIIICEAQNQALPSGHSK	1	N-Term(iTRAQ4plex); K1(iTRAQ4plex); C9(Methylthio); K22(iTRAQ4plex)	0.819		37		
94420689	NCAM1	neural cell adhesion molecule 1 isoform 1	22.64	0	16	171		93.3								
									CVVTGEDGSESEATVNVK	8	N-Term(iTRAQ4plex); C1(Methylthio); K18(iTRAQ4plex)		7.41	101	2	1079.52
									EASMEGIVTIVGLKPETTYAVF	8	N-Term(iTRAQ4plex); K14(iTRAQ4plex		6.49	80	3	884.82
									YIFSDDSSQLTIK	8	N-Term(iTRAQ4plex); K13(iTRAQ4plex		6.37	89	2	902.99
									DKDISWFSPNGEK	7	N-Term(iTRAQ4plex); K2(iTRAQ4plex); K13(iTRAQ4plex)		6.34	59	3	652.35
									LEGQMGEDGNSIK	14	N-Term(iTRAQ4plex); K13(iTRAQ4plex		6.08	87	2	833.42
									GLGEISAASEFK	22	N-Term(iTRAQ4plex); K12(iTRAQ4plex		5.79	86	2	748.92
									ALSSEWKPEIR	14	N-Term(iTRAQ4plex); K7(iTRAQ4plex		5.33	58	3	535.31
									DIQVIVNVPPTIQAF	37	N-Term(iTRAQ4plex		5.18	77	2	904.04
									FFLCQVAGDAK	16	N-Term(iTRAQ4plex); C4(Methylthio); K11(iTRAQ4plex)		4.93	87	2	766.90
									FIVLSNNYLQIR	9	N-Term(iTRAQ4plex		4.78	64	2	812.47
									LPSGSDHVMLK	13	N-Term(iTRAQ4plex); K11(iTRAQ4plex		4.66	52	3	491.28
									LPSGSDHVMLK	2	N-Term(iTRAQ4plex); M9(Oxidation); K11(iTRAQ4plex)		4.28	38	3	496.61
									NDEAEYICIAENK	5	N-Term(iTRAQ4plex); C8(Methylthio); K13(iTRAQ4plex)		4.12	82	2	923.44
									DISWFSPNGEK	4	N-Term(iTRAQ4plex); K11(iTRAQ4plex		3.93	54	2	784.40
									VSSLTLK	1	N-Term(iTRAQ4plex); K7(iTRAQ4plex			43		
									GEINFK	2	N-Term(iTRAQ4plex); K6(iTRAQ4plex			43		
310128730	LOC652797	PREDICTED: pyruvate kinase isozymes M1/M2-like isoform 2, partial	22.49	0	6	33		35.9								
									KGVNLPGAAVDLPAVSEK	4	N-Term(iTRAQ4plex); K1(iTRAQ4plex); K18(iTRAQ4plex)		5.68	69	3	733.11
									NTGIICTIGPASR	4	N-Term(iTRAQ4plex); C6(Methylthio		4.99	56	2	746.89
									ITLDNAYMEK	12	N-Term(iTRAQ4plex); K10(iTRAQ4plex		4.74	74	2	743.40
									GDLGIEIPAEK	9	N-Term(iTRAQ4plex); K11(iTRAQ4plex		4.18	56	2	715.41
									LDIDSPPITAR	3	N-Term(iTRAQ4plex		4.12	52	2	671.38
									CDENILWLDYK	1	N-Term(iTRAQ4plex); C1(Methylthio); K11(iTRAQ4plex)			55		
310128734	LOC652797	PREDICTED: pyruvate kinase isozymes M1/M2-like isoform 3	22.42	0	5	24		30.7								
									KGVNLPGAAVDLPAVSEK	4	N-Term(iTRAQ4plex); K1(iTRAQ4plex); K18(iTRAQ4plex)		5.68	69	3	733.11
									NTGIICTIGPASR	4	N-Term(iTRAQ4plex); C6(Methylthio		4.99	56	2	746.89
									ITLDNAYMEK	12	N-Term(iTRAQ4plex); K10(iTRAQ4plex		4.74	74	2	743.40
									LDIDSPPITAR	3	N-Term(iTRAQ4plex		4.12	52	2	671.38
									CDENILWLDYK	1	N-Term(iTRAQ4plex); C1(Methylthio); K11(iTRAQ4plex)			55		
7705413	NTM	neurotrimin isoform 1	22.38	0	6	69		37.9								
									EQSGDYECSASNDVAAPVVF	8	N-Term(iTRAQ4plex); C8(Methylthio		6.76	101	2	1144.01
									AVGFVSEDEYLEIQGITF	9	N-Term(iTRAQ4plex		5.88	92	2	1085.56
									VHLIVQVSPK	18	N-Term(iTRAQ4plex); K10(iTRAQ4plex		5.09	79	3	469.97
									VTVNYPPYISEAK	29	N-Term(iTRAQ4plex); K13(iTRAQ4plex		4.81	76	2	884.99
									STILYAGNDK	2	N-Term(iTRAQ4plex); K10(iTRAQ4plex		3.96	51	2	685.39
									WCLDPR	3	N-Term(iTRAQ4plex); C2(Methylthio			34		
115298665	NTM	neurotrimin isoform 2	22.38	0	6	69		37.9								
									EQSGDYECSASNDVAAPVVF	8	N-Term(iTRAQ4plex); C8(Methylthio		6.76	101	2	1144.01
									AVGFVSEDEYLEIQGITF	9	N-Term(iTRAQ4plex		5.88	92	2	1085.56
									VHLIVQVSPK	18	N-Term(iTRAQ4plex); K10(iTRAQ4plex		5.09	79	3	469.97
									VTVNYPPYISEAK	29	N-Term(iTRAQ4plex); K13(iTRAQ4plex		4.81	76	2	884.99
									STILYAGNDK	2	N-Term(iTRAQ4plex); K10(iTRAQ4plex		3.96	51	2	685.39
									WCLDPR	3	N-Term(iTRAQ4plex); C2(Methylthio			34		
225637524	SEMA7A	semaphorin-7A isoform 3	22.36	0	8	39		57								
									SVLQSINPAEPHK	10	N-Term(iTRAQ4plex); K13(iTRAQ4plex		6.05	79	3	569.99
									CLPDQQPIPTETFQVADF	5	N-Term(iTRAQ4plex); C1(Methylthio		5.65	92	2	1124.55
									AAAIQTMSLDAEF	8	N-Term(iTRAQ4plex		4.95	78	2	760.90
									MQASHGETFHVLYLTTDF	5	N-Term(iTRAQ4plex		4.38	66	3	750.71
									AMLVCSDAATNK	2	N-Term(iTRAQ4plex); C5(Methylthio); K12(iTRAQ4plex)		4.06	87	2	779.39
									VVEPGEQEHSFAFNIMEIQPFF	2	N-Term(iTRAQ4plex		3.91	43	3	916.79
									YYLSCPMESR	2	N-Term(iTRAQ4plex); C5(Methylthio		3.54	44	2	719.82
									WNTFLK	5	N-Term(iTRAQ4plex); K6(iTRAQ4plex			42		
50363217	SERPINA1	alpha-1-antitrypsin precursor	22.25	10	10	32	0.476	46.7								
									DTEEEDFHVDQVTTVK	2	N-Term(iTRAQ4plex); K16(iTRAQ4plex	0.313	4.83	64	3	727.36
									LSITGTYDLK	13	N-Term(iTRAQ4plex); K10(iTRAQ4plex	0.569	4.68	91	2	699.91
									SVLGQLGITK	5	N-Term(iTRAQ4plex); K10(iTRAQ4plex	0.522	3.9	54	2	652.41
									QINDYVEK	2	N-Term(iTRAQ4plex); K8(iTRAQ4plex	0.417	3.86	44	2	648.86
									LQHLENELTHDIITK	4	N-Term(iTRAQ4plex); K15(iTRAQ4plex	0.43	3.6	71	3	698.06
									LSSWVLLMK	1	N-Term(iTRAQ4plex); K9(iTRAQ4plex	0.327		46		
									IVDLVK	2	N-Term(iTRAQ4plex); K6(iTRAQ4plex	0.42		44		

									AVLTIDEK	1	N-Term(iTRAQ4plex); K8(iTRAQ4plex	0.593		40		
									KLSSWVLLMK	1	N-Term(iTRAQ4plex); K1(iTRAQ4plex); K10(iTRAQ4plex)	0.189		35		
73858564	SERPINA6	corticosteroid-binding globulin precursor	22.22	6	6	30	0.795	45.1								
									SETEIHQGFQHLHQLFAK	4	N-Term(iTRAQ4plex); K18(iTRAQ4plex	0.832	8.93	71	3	813.44
									EENFYVDETTVVK	11	N-Term(iTRAQ4plex); K13(iTRAQ4plex	0.762	6.27	82	2	930.98
									WSAGLTSSQVDLYIPK	2	N-Term(iTRAQ4plex); K16(iTRAQ4plex	0.848	5.77	79	2	1027.06
									GTWTQPFDLASTR	4	N-Term(iTRAQ4plex	0.813	5.22	60	2	812.42
									MNTVIAALSR	8	N-Term(iTRAQ4plex	0.791	4.12	65	2	610.35
									HYYESEVLAMNFQDWATASR	1	N-Term(iTRAQ4plex			38		
208431838	LY6H	lymphocyte antigen 6H isoform a	22.14	0	3	13		14.7								
									QCQPSDTVCASVR	7	N-Term(iTRAQ4plex); C2(Methylthio); C9(Methylthio)		4.47	70	2	815.35
									MCASSCDFVK	4	N-Term(iTRAQ4plex); C2(Methylthio); C6(Methylthio); K10(iTRAQ4plex)		4.27	68	2	735.81
									VDVDCCEK	2	N-Term(iTRAQ4plex); C5(Methylthio); C6(Methylthio); K8(iTRAQ4plex)		3.21	39	2	645.78
148743776	VSTM2A	V-set and transmembrane domain-containing protein 2A	22.03	3	3	9	0.857	25.8								
									VTDANYGELQEHK	5	N-Term(iTRAQ4plex); K13(iTRAQ4plex	0.857	6.47	76	3	597.97
									MQAFEASPMWLQDMKPR	2	N-Term(iTRAQ4plex); K15(iTRAQ4plex	0.996	4	49	3	785.40
									GPEDLDPGAEGAGAQVELLPDR	2	N-Term(iTRAQ4plex	0.691	3.29	40	2	1175.59
4826904	SERPINI1	neuroserpin precursor	21.95	7	7	31	0.895	46.4								
									VMHPETMNTSGHDFEEL	4	N-Term(iTRAQ4plex	0.953	6.26	32	3	706.65
									AQLVEEWANSVK	4	N-Term(iTRAQ4plex); K12(iTRAQ4plex	0.913	5.51	85	2	831.46
									FTVEQEIDLK	7	N-Term(iTRAQ4plex); K10(iTRAQ4plex	0.805	4.67	75	2	755.43
									EFSNMVTAK	3	N-Term(iTRAQ4plex); K9(iTRAQ4plex	0.775	4.48	51	2	657.86
									TGTILFMGR	4	N-Term(iTRAQ4plex	0.885	3.68	48	2	570.32
									ALGITEIFIK	6	N-Term(iTRAQ4plex); K10(iTRAQ4plex	0.902	3.63	77	2	696.94
									VMHPETMNTSGHDFEEL	2	N-Term(iTRAQ4plex); M2(Oxidation	1.017	3.38	41	3	711.98
									YFNAAVNHVDFSQNVAVANYIN	1	N-Term(iTRAQ4plex); K23(iTRAQ4plex	1.076		37		
55925576	IGFBP2	insulin-like growth factor-binding protein 2 precursor	21.95	5	5	15	1.28	35.1								
									GDPECHLFYNEQQEAR	4	N-Term(iTRAQ4plex); C5(Methylthio	1.309	5.51	73	3	709.31
									LEGEACGVYTPR	5	N-Term(iTRAQ4plex); C6(Methylthio	1.039	4.89	56	2	742.85
									LAACGPPPVAPPAAVAAVAGGA	4	N-Term(iTRAQ4plex); C4(Methylthio	1.685	4.37	45	2	1087.59
									GECWCVNPNTGK	1	N-Term(iTRAQ4plex); C3(Methylthio); C5(Methylthio); K12(iTRAQ4plex)	1.281		54		
214010185	APLP2	amyloid-like protein 2 isoform 4	21.84	0	11	33		59.1								
									SQVMTHLHVIEER	5	N-Term(iTRAQ4plex		6.14	86	4	431.48
									QTLIQHFQAMVK	4	N-Term(iTRAQ4plex); K12(iTRAQ4plex		6.02	54	2	866.49
									KEWEEAELQAK	2	N-Term(iTRAQ4plex); K1(iTRAQ4plex); K11(iTRAQ4plex)		5.79	51	3	598.33
									LNMHVNIQTGK	4	N-Term(iTRAQ4plex); K11(iTRAQ4plex		5.31	67	3	514.96
									EWEEAELQAK	2	N-Term(iTRAQ4plex); K10(iTRAQ4plex		5.14	55	2	760.90
									VEAMLNDR	2	N-Term(iTRAQ4plex		3.42	43	2	546.28
									EMIFNAER	2	N-Term(iTRAQ4plex		3.38	43	2	577.29
									VSIDNWCR	4	N-Term(iTRAQ4plex); C7(Methylthio		3.26	41	2	591.78
									ADMDQFTASISETPVDVR	1	N-Term(iTRAQ4plex			59		
									MALENYLAALQSDPPRPHR	2	N-Term(iTRAQ4plex			52		
									FVTPFK	5	N-Term(iTRAQ4plex); K6(iTRAQ4plex			43		
119392081	CFI	complement factor I preproprotein	21.78	12	12	50	0.979	65.7								
									IIFHENYNAGTYQNDIALIEMK	2	N-Term(iTRAQ4plex); K22(iTRAQ4plex	1.226	5.62	90	3	962.50
									GLETSLAECTFTK	3	N-Term(iTRAQ4plex); C9(Methylthio); K13(iTRAQ4plex)	0.916	5.6	79	2	867.44
									AQLGDLPWQVAIK	9	N-Term(iTRAQ4plex); K13(iTRAQ4plex	1.062	5.52	94	2	864.01
									VFSLQWGEVK	16	N-Term(iTRAQ4plex); K10(iTRAQ4plex	1.035	4.59	75	2	740.92
									SFPTYCQQK	2	N-Term(iTRAQ4plex); C6(Methylthio); K9(iTRAQ4plex)	0.858	4.22	44	2	718.35
									SLECLHPGTK	4	N-Term(iTRAQ4plex); C4(Methylthio); K10(iTRAQ4plex)	0.962	4.09	56	2	709.87
									IVIEYVDR	7	N-Term(iTRAQ4plex	1.036	3.35	37	2	575.83
									LSCGVK	1	N-Term(iTRAQ4plex); C3(Methylthio); K6(iTRAQ4plex)	1.467		43		
									TMGYQDFADVVCYTQK	1	N-Term(iTRAQ4plex); C12(Methylthio); K16(iTRAQ4plex)	0.897		43		
									FSVSLK	2	N-Term(iTRAQ4plex); K6(iTRAQ4plex	0.977		39		
									TMFICK	2	N-Term(iTRAQ4plex); C5(Methylthio); K6(iTRAQ4plex)	0.932		36		
									VFCQPWQR	1	N-Term(iTRAQ4plex); C3(Methylthio	1.142		33		
260064050	SERPINF2	alpha-2-antiplasmin isoform b precursor	21.78	0	7	52		47.9								
									LQQVLHAGSGPCLPHLLSR	2	N-Term(iTRAQ4plex); C12(Methylthio		8.2	90	3	739.40
									WFLLEQPEIQVAHFPFK	10	N-Term(iTRAQ4plex); K17(iTRAQ4plex		7.7	67	3	806.45
									LCQDLGPGAFR	18	N-Term(iTRAQ4plex); C2(Methylthio		4.7	64	2	683.84
									QEDDLANINQWVK	8	N-Term(iTRAQ4plex); K13(iTRAQ4plex		4.01	78	2	930.99
									DFLQSLK	11	N-Term(iTRAQ4plex); K7(iTRAQ4plex		3.54	46	2	569.84
									DSFHLDEQFTVPVEMMQAR	2	N-Term(iTRAQ4plex			50		
									LFGPDLK	1	N-Term(iTRAQ4plex); K7(iTRAQ4plex			35		
295849303	C2	complement C2 isoform 3	21.75	0	10	45		60.3								
									RNDYLDIYAIGVGK	2	N-Term(iTRAQ4plex); K14(iTRAQ4plex		5.35	60	2	943.02
									CSSNLVLTGSSER	9	N-Term(iTRAQ4plex); C1(Methylthio		4.78	83	2	771.87
									KNQGILEFYGDDIALLK	2	N-Term(iTRAQ4plex); K1(iTRAQ4plex); K17(iTRAQ4plex)		4.74	70	3	790.46
									GESGGAVFLER	2	N-Term(iTRAQ4plex		4.67	60	2	633.33
									AVISPGFDVFAK	6	N-Term(iTRAQ4plex); K12(iTRAQ4plex		4.33	101	2	769.94
									HAIILLTDGK	4	N-Term(iTRAQ4plex); K10(iTRAQ4plex		3.99	49	2	684.93
									ALHQVFEHMLDVSK	8	N-Term(iTRAQ4plex); K14(iTRAQ4plex		3.9	68	3	648.02
									EILNINQK	6	N-Term(iTRAQ4plex); K8(iTRAQ4plex		3.66	46	2	630.38
									QHLGDVLNFLPL	4	N-Term(iTRAQ4plex		3.5	73	2	755.43
									LNINLK	2	N-Term(iTRAQ4plex); K6(iTRAQ4plex			41		
115583663	SERPINF2	alpha-2-antiplasmin isoform a precursor	21.59	1	8	56	0.953	54.5								
									LQQVLHAGSGPCLPHLLSR	2	N-Term(iTRAQ4plex); C12(Methylthio		8.2	90	3	739.40
									WFLLEQPEIQVAHFPFK	10	N-Term(iTRAQ4plex); K17(iTRAQ4plex		7.7	67	3	806.45
									LGNQEPGGQTALK	4	N-Term(iTRAQ4plex); K13(iTRAQ4plex	0.953	5.6	60	2	800.95
									LCQDLGPGAFR	18	N-Term(iTRAQ4plex); C2(Methylthio		4.7	64	2	683.84
									QEDDLANINQWVK	8	N-Term(iTRAQ4plex); K13(iTRAQ4plex		4.01	78	2	930.99
									DFLQSLK	11	N-Term(iTRAQ4plex); K7(iTRAQ4plex		3.54	46	2	569.84
									DSFHLDEQFTVPVEMMQAR	2	N-Term(iTRAQ4plex			50		

									LFGPDLK	1	N-Term(iTRAQ4plex); K7(iTRAQ4plex			35		
47132620	KRT2	keratin, type II cytoskeletal 2 epidermal	21.28	9	13	53	2.405	65.4								
									FLEQQNQVLQTK	12	N-Term(iTRAQ4plex); K12(iTRAQ4plex		6.7	96	2	882.50
									TAAENDFVTLK	6	N-Term(iTRAQ4plex); K11(iTRAQ4plex	2.502	5.53	87	2	748.91
									NVQDAIADAEQR	10	N-Term(iTRAQ4plex	2.433	4.64	78	2	737.38
									DVDNAYMIK	4	N-Term(iTRAQ4plex); K9(iTRAQ4plex	2.051	4.64	62	2	678.85
									LNDLEEALQQAK	4	N-Term(iTRAQ4plex); K12(iTRAQ4plex	2.517	4.33	87	2	830.46
									YEELQVTVGR	4	N-Term(iTRAQ4plex	2.262	4.27	48	2	669.36
									VDPEIQNVK	2	N-Term(iTRAQ4plex); K9(iTRAQ4plex	1.834	4.25	44	2	665.38
									SKEEAEALYHSK	2	N-Term(iTRAQ4plex); K2(iTRAQ4plex); K12(iTRAQ4plex)	1.872	3.75	50	3	608.67
									STSSFSCLSR	2	N-Term(iTRAQ4plex); C7(Methylthio	3.557	3.62	56	2	632.79
									FASFIDK	4	N-Term(iTRAQ4plex); K7(iTRAQ4plex		3.19	53	2	558.31
									VDLLNQEIEFLK	1	N-Term(iTRAQ4plex); K12(iTRAQ4plex	2.835		73		
21070969	NRXN3	neurexin-3-beta isoform 1 precursor	21.21	11	16	71	0.856	117.2								
									QLAEMQNAAGVK	4	N-Term(iTRAQ4plex); K12(iTRAQ4plex	0.98	6.23	82	2	774.43
									EASILSYDGSMYMK	2	N-Term(iTRAQ4plex); K14(iTRAQ4plex	0.775	6.18	92	2	941.97
									GYIHYVFDLGNGPNVIK	2	N-Term(iTRAQ4plex); K17(iTRAQ4plex	0.799	6.14	77	3	732.07
									LEFHNIETGIMTEK	6	N-Term(iTRAQ4plex); K14(iTRAQ4plex	0.819	6.11	59	3	650.68
									GNSDRPLNDNQWHNVVITR	3	N-Term(iTRAQ4plex	0.833	6.11	64	3	793.74
									VLNMAAENNPNIK	8	N-Term(iTRAQ4plex); K13(iTRAQ4plex		5.72	100	2	858.47
									LTVDDDVAEGTMVGDHTR	8	N-Term(iTRAQ4plex	0.895	5.55	124	3	692.33
									IDSAPGLGDFLQLHIEQGK	4	N-Term(iTRAQ4plex); K19(iTRAQ4plex		5.37	85	3	776.10
									SGGLILYTWPANDRPSTR	3	N-Term(iTRAQ4plex		4.75	36	3	716.72
									AYGLLVATTSR	2	N-Term(iTRAQ4plex	0.853	4.71	36	2	648.37
									LTVDDDVAEGTMVGDHTR	2	N-Term(iTRAQ4plex); M12(Oxidation	0.997	4.68	69	3	697.67
									ANDGEWYHVDIQR	2	N-Term(iTRAQ4plex	0.887	4.19	47	2	873.92
									NIIADPVTFK	10	N-Term(iTRAQ4plex); K10(iTRAQ4plex		4.03	60	2	703.42
									LEFHNIETGIMTEK	2	N-Term(iTRAQ4plex); M11(Oxidation); K14(iTRAQ4plex)	1.115	3.95	59	3	656.01
									LPDLINDALHR	9	N-Term(iTRAQ4plex	0.808	3.76	55	3	474.27
									GDLYMAGLAQGMYSNLPK	1	N-Term(iTRAQ4plex); K18(iTRAQ4plex	0.943		69		
									LAVGFSTTVK	1	N-Term(iTRAQ4plex); K10(iTRAQ4plex			39		
5802984	B3GNT1	N-acetyllactosaminide beta-1,3-N-acetylglucosaminyltransferase	21.2	7	7	105	0.866	47.1								
									TALASGGVLDASGDYR	40	N-Term(iTRAQ4plex	0.911	6.42	103	2	848.94
									WEGPLSVSVFAATK	6	N-Term(iTRAQ4plex); K14(iTRAQ4plex	0.898	5.92	104	2	890.50
									VAMHLVCPSR	31	N-Term(iTRAQ4plex); C7(Methylthio	0.854	4.37	55	2	651.83
									SCQEVFDK	13	N-Term(iTRAQ4plex); C2(Methylthio); K8(iTRAQ4plex)	0.844	4.06	53	2	645.31
									EMLDQSNQWGGTALVVPAFEIR	3	N-Term(iTRAQ4plex	0.721	3.48	75	2	1303.17
									EPGEFALLR	8	N-Term(iTRAQ4plex	0.835	3.38	37	2	588.33
									VAMHLVCPSR	2	N-Term(iTRAQ4plex); M3(Oxidation); C7(Methylthio)	0.864	3.36	47	3	440.22
									YEAAVPDPR	2	N-Term(iTRAQ4plex	0.804		41		
68533260	CHI3L2	chitinase-3-like protein 2 isoform c	20.9	0	5	11		34.6								
									GNQWVGYDDVK	2	N-Term(iTRAQ4plex); K11(iTRAQ4plex		4.77	63	2	784.90
									QMIDNSYQVEK	4	N-Term(iTRAQ4plex); K11(iTRAQ4plex		4.41	51	2	821.92
									SCNQGPYPLVQAVK	2	N-Term(iTRAQ4plex); C2(Methylthio); K14(iTRAQ4plex)		4.4	66	2	919.49
									LLLTAGVSAGR	2	N-Term(iTRAQ4plex		3.6	55	2	601.37
									GPSSYYNVEYAVGYWIHK	1	N-Term(iTRAQ4plex); K18(iTRAQ4plex			47		
237858682	NFASC	neurofascin isoform 6 precursor	20.88	0	13	44		68.9								
									EDDSLTIFGVAER	4	N-Term(iTRAQ4plex		5.45	81	2	798.41
									SGGRPEEYEGEYQCFAR	2	N-Term(iTRAQ4plex); C14(Methylthio		5.29	35	3	723.32
									KEDDSLTIFGVAER	2	N-Term(iTRAQ4plex); K1(iTRAQ4plex		5.08	56	3	623.34
									AAPYWLDEPK	9	N-Term(iTRAQ4plex); K10(iTRAQ4plex		4.68	76	2	739.40
									LDCPFFGSPIPTLR	2	N-Term(iTRAQ4plex); C3(Methylthio		4.57	53	2	876.95
									GTTVQLECR	2	N-Term(iTRAQ4plex); C8(Methylthio		4.52	46	2	598.80
									DDEPLYIGNR	2	N-Term(iTRAQ4plex		4.14	34	2	668.34
									EVAGDTIIFR	2	N-Term(iTRAQ4plex		4.13	55	2	632.86
									NLILAPGEDGR	4	N-Term(iTRAQ4plex		3.79	50	2	649.86
									GNPAPSFHWTR	2	N-Term(iTRAQ4plex		3.37	38	2	707.36
									SGTLVIDFR	2	N-Term(iTRAQ4plex		3.22	41	2	576.33
									LTVSWLK	8	N-Term(iTRAQ4plex); K7(iTRAQ4plex			53		
									FENFNK	3	N-Term(iTRAQ4plex); K6(iTRAQ4plex			37		
6006001	GPX3	glutathione peroxidase 3 precursor	20.8	5	5	46	0.755	25.5								
									QEPGENSEILPTLK	13	N-Term(iTRAQ4plex); K14(iTRAQ4plex	0.771	4.91	76	2	922.01
									FLVGPDGIPIMR	13	N-Term(iTRAQ4plex	0.743	4.04	52	2	729.92
									LFWEPMK	5	N-Term(iTRAQ4plex); K7(iTRAQ4plex	0.795	3.25	40	2	619.85
									MDILSYMR	1	N-Term(iTRAQ4plex); M1(Oxidation	0.742		46		
									MDILSYMR	5	N-Term(iTRAQ4plex	0.745		45		
									FYTFLK	8	N-Term(iTRAQ4plex); K6(iTRAQ4plex	0.806		41		
237858680	NFASC	neurofascin isoform 5 precursor	20.68	0	13	44		69.5								
									EDDSLTIFGVAER	4	N-Term(iTRAQ4plex		5.45	81	2	798.41
									SGGRPEEYEGEYQCFAR	2	N-Term(iTRAQ4plex); C14(Methylthio		5.29	35	3	723.32
									KEDDSLTIFGVAER	2	N-Term(iTRAQ4plex); K1(iTRAQ4plex		5.08	56	3	623.34
									AAPYWLDEPK	9	N-Term(iTRAQ4plex); K10(iTRAQ4plex		4.68	76	2	739.40
									LDCPFFGSPIPTLR	2	N-Term(iTRAQ4plex); C3(Methylthio		4.57	53	2	876.95
									GTTVQLECR	2	N-Term(iTRAQ4plex); C8(Methylthio		4.52	46	2	598.80
									DDEPLYIGNR	2	N-Term(iTRAQ4plex		4.14	34	2	668.34
									EVAGDTIIFR	2	N-Term(iTRAQ4plex		4.13	55	2	632.86
									NLILAPGEDGR	4	N-Term(iTRAQ4plex		3.79	50	2	649.86
									GNPAPSFHWTR	2	N-Term(iTRAQ4plex		3.37	38	2	707.36
									SGTLVIDFR	2	N-Term(iTRAQ4plex		3.22	41	2	576.33
									LTVSWLK	8	N-Term(iTRAQ4plex); K7(iTRAQ4plex			53		
									FENFNK	3	N-Term(iTRAQ4plex); K6(iTRAQ4plex			37		
4557305	ALDOA	fructose-bisphosphate aldolase A	20.6	5	6	24	0.621	39.4								
									FSHEEIAMATVTALR	9	N-Term(iTRAQ4plex	0.63	6.38	77	3	607.32
									IGEHTPSALAIMENANVLAR	2	N-Term(iTRAQ4plex	1.006	4.76	49	3	751.07
									ADDGRPFPQVIK	4	N-Term(iTRAQ4plex); K12(iTRAQ4plex	0.671	4.57	50	2	815.96
									FSHEEIAMATVTALR	2	N-Term(iTRAQ4plex); M8(Oxidation	0.817	4.02	67	3	612.65
									QLLLTADDR	2	N-Term(iTRAQ4plex	0.501	3.46	48	2	594.84
									VLAAVYK	2	N-Term(iTRAQ4plex); K7(iTRAQ4plex		3.15	41	2	526.34
									PYQYPALTPEQK	3	N-Term(iTRAQ4plex); K12(iTRAQ4plex	0.564		60		
40068512	FUCA2	plasma alpha-L-fucosidase precursor	20.56	10	10	33	0.96	54								
									WPTSGQLFLGHPK	8	N-Term(iTRAQ4plex); K13(iTRAQ4plex	0.844	6.28	70	3	586.00
									FDPTWESLDAR	5	N-Term(iTRAQ4plex	0.989	3.79	61	2	740.87

									YEDFGPLFTAK	5	N-Term(iTRAQ4plex); K11(iTRAQ4plex	1.028	3.75	71	2	788.42
									WENCMTIDK	2	N-Term(iTRAQ4plex); C4(Methylthio); K9(iTRAQ4plex)	0.831	3.55	54	2	737.34
									LVYAIFLK	4	N-Term(iTRAQ4plex); K8(iTRAQ4plex	0.89	3.25	53	2	627.91
									QLPAWFDQAK	1	N-Term(iTRAQ4plex); K10(iTRAQ4plex	0.929		64		
									WGAGSICK	2	N-Term(iTRAQ4plex); C7(Methylthio); K8(iTRAQ4plex)	0.97		53		
									YIVLTSK	3	N-Term(iTRAQ4plex); K7(iTRAQ4plex	0.933		42		
									YVEFMK	2	N-Term(iTRAQ4plex); K6(iTRAQ4plex	1.117		38		
89886217	CBLN3	cerebellin-3 precursor	20.49	3	3	15	1.021	21.5								
									AAAGGPGGAALGEAPPGF	10	N-Term(iTRAQ4plex	1.007	6.77	136	2	810.93
									EAATSSVLLPLDPGDR	2	N-Term(iTRAQ4plex	1.018		55		
156231037	KNG1	kininogen-1 isoform 1	20.34	0	13	125		71.9								
									DIPTNSPELEETLTHTITK	12	N-Term(iTRAQ4plex); K19(iTRAQ4plex		7.73	108	3	809.77
									YNSQNQSNNQFVLYF	9	N-Term(iTRAQ4plex		6.17	84	2	1009.99
									ESNEELTESCETK	2	N-Term(iTRAQ4plex); C10(Methylthio); K13(iTRAQ4plex)		5.95	82	2	916.91
									TVGSDTFYSFK	28	N-Term(iTRAQ4plex); K11(iTRAQ4plex		5.6	74	2	770.39
									AATGECTATVGK	12	N-Term(iTRAQ4plex); C6(Methylthio); K12(iTRAQ4plex)		5.53	88	2	721.87
									RPPGFSPFR	15	N-Term(iTRAQ4plex		4.84	74	3	402.23
									KYNSQNQSNNQFVLYF	3	N-Term(iTRAQ4plex); K1(iTRAQ4plex		4.72	78	3	764.40
									KYFIDFVAR	16	N-Term(iTRAQ4plex); K1(iTRAQ4plex		4.47	66	3	482.95
									TWQDCEYK	7	N-Term(iTRAQ4plex); C5(Methylthio); K8(iTRAQ4plex)		3.76	41	2	703.82
									ENFLFLTPDCK	5	N-Term(iTRAQ4plex); C10(Methylthio); K11(iTRAQ4plex)		3.74	63	2	830.92
									QVVAGLNFR	9	N-Term(iTRAQ4plex		3.66	44	2	574.34
									IASFSQNCDIYPGK	3	N-Term(iTRAQ4plex); C8(Methylthio); K14(iTRAQ4plex)		3.45	71	2	938.96
									YFIDFVAR	4	N-Term(iTRAQ4plex			37		
4557373	BTD	biotinidase precursor	20.26	8	8	42	0.937	61.1								
									HVVYPTAWMNQLPLLAAIEIQK	2	N-Term(iTRAQ4plex); K22(iTRAQ4plex	0.97	5.66	53	3	941.87
									TSIYPFLDFMPSPQVVR	6	N-Term(iTRAQ4plex	0.914	5.4	59	2	1071.06
									GDMFLVANLGTK	2	N-Term(iTRAQ4plex); K12(iTRAQ4plex	1.13	5.21	63	2	777.44
									LSSGLVTAALYGR	5	N-Term(iTRAQ4plex	1.013	5.1	88	2	726.42
									SHLIIAQVAK	7	N-Term(iTRAQ4plex); K10(iTRAQ4plex	1.017	4.65	70	3	456.63
									VDLITFDTPFAGR	10	N-Term(iTRAQ4plex	0.922	4.58	69	2	798.43
									WNPCLEPHR	7	N-Term(iTRAQ4plex); C4(Methylthio	0.888	3.64	49	3	447.88
									HNLYFEAAFDVPLK	3	N-Term(iTRAQ4plex); K14(iTRAQ4plex	0.851	3.19	52	2	976.53
156616294	PGLYRP2	N-acetylmuramoyl-L-alanine amidase precursor	20.14	7	7	34	1.18	62.2								
									HTASAWLMSAPNSGPHNF	2	N-Term(iTRAQ4plex	1.235	6.56	63	3	693.34
									AGLLRPDYALLGHR	4	N-Term(iTRAQ4plex	1.128	6.03	39	3	566.00
									GSQTQSHPDLGTEGCWDQLSAPR	4	N-Term(iTRAQ4plex); C15(Methylthio	1.204	5.65	83	3	887.41
									GCPDVQASLPDAK	4	N-Term(iTRAQ4plex); C2(Methylthio); K13(iTRAQ4plex)	1.047	5.41	93	2	817.91
									TDCPGDALFDLLR	6	N-Term(iTRAQ4plex); C3(Methylthio	1.193	5.29	71	2	813.40
									EFTEAFLGCPAIHPR	13	N-Term(iTRAQ4plex); C9(Methylthio	1.232	4.9	85	2	939.47
									DGSPDVTTADIGANTPDATI	1	N-Term(iTRAQ4plex); K20(iTRAQ4plex	1.141		34		
16507237	HSPA5	78 kDa glucose-regulated protein precursor	20.03	9	10	21	0.973	72.3								
									TWNDPSVQQDIK	4	N-Term(iTRAQ4plex); K12(iTRAQ4plex	0.967	5.16	69	2	859.95
									NQLTSNPENTVFDAK	2	N-Term(iTRAQ4plex); K15(iTRAQ4plex	1.046	4.97	74	2	983.51
									IEWLESHQDADIEDFK	3	N-Term(iTRAQ4plex); K16(iTRAQ4plex	1.204	4.36	57	3	755.04
									ITPSYVAFTPEGER	2	N-Term(iTRAQ4plex	0.973	4.1	65	2	855.94
									KSDIDEIVLVGGSTR	2	N-Term(iTRAQ4plex); K1(iTRAQ4plex	0.939	3.83	68	2	939.04
									TFAPEEISAMVLTK	2	N-Term(iTRAQ4plex); K14(iTRAQ4plex	0.797	3.62	91	2	913.01
									FEELNMDLFR	2	N-Term(iTRAQ4plex	0.836	3.56	42	2	729.37
									IINEPTAAAIAYGLDK	2	N-Term(iTRAQ4plex); K16(iTRAQ4plex		3.25	60	2	974.56
									VMEHFIK	1	N-Term(iTRAQ4plex); K7(iTRAQ4plex	1.366		48		
									DAGTIAGLNVMF	1	N-Term(iTRAQ4plex	0.847		38		
17402888	NPTXR	neuronal pentraxin receptor	20	9	9	47	0.712	52.8								
									LVEAFGGATK	10	N-Term(iTRAQ4plex); K10(iTRAQ4plex	0.795	4.14	53	2	640.88
									EELLLLQSTAEQLR	3	N-Term(iTRAQ4plex	0.894	3.92	53	2	894.01
									IDRLEQELPAR	5	N-Term(iTRAQ4plex	0.763	3.64	45	3	495.28
									VAQLPLSLK	13	N-Term(iTRAQ4plex); K9(iTRAQ4plex	0.691	3.48	43	2	628.92
									ELDVLQGR	4	N-Term(iTRAQ4plex	0.808	3.2	43	2	537.31
									VAELEHGSSAYSPPDAFK	1	N-Term(iTRAQ4plex); K18(iTRAQ4plex	0.805		54		
									MDQLEGQLLAQVLALEK	1	N-Term(iTRAQ4plex); K17(iTRAQ4plex	0.912		47		
									ISIPIR	9	N-Term(iTRAQ4plex	0.669		35		
									AAFDVCK	1	N-Term(iTRAQ4plex); C6(Methylthio); K7(iTRAQ4plex)	0.83		34		
4557321	APOA1	apolipoprotein A-I preproprotein	19.85	5	5	8	0.512	30.8								
									VQPYLDDFQK	3	N-Term(iTRAQ4plex); K10(iTRAQ4plex	0.67	4.16	52	2	770.92
									LLDNWDSVTSTFSK	1	N-Term(iTRAQ4plex); K14(iTRAQ4plex	0.384		53		
									LSPLGEEMR	1	N-Term(iTRAQ4plex	0.435		46		
									WQEEMELYR	2	N-Term(iTRAQ4plex	0.478		44		
									THLAPYSDELR	1	N-Term(iTRAQ4plex	0.601		34		
73858568	SERPING1	plasma protease C1 inhibitor precursor	19.8	9	9	41	0.866	55.1								
									VTTSQDMLSIMEK	10	N-Term(iTRAQ4plex); K13(iTRAQ4plex	0.84	6.16	109	2	885.97
									GVTSVSQIFHSPDLAIF	9	N-Term(iTRAQ4plex	0.958	5.76	117	2	986.04
									DFTCVHQALK	2	N-Term(iTRAQ4plex); C4(Methylthio); K10(iTRAQ4plex)	0.88	4.7	69	2	748.39
									TNLESILSYPK	2	N-Term(iTRAQ4plex); K11(iTRAQ4plex	0.94	4.47	68	2	776.95
									LEDMEQALSPSVFK	5	N-Term(iTRAQ4plex); K14(iTRAQ4plex	0.681	4.18	86	2	941.50
									FQPTLLTLPR	4	N-Term(iTRAQ4plex	0.818	3.94	57	2	665.40
									LLDSLPSDTR	4	N-Term(iTRAQ4plex	0.721	3.64	51	2	630.85
									MEPFHFK	3	N-Term(iTRAQ4plex); K7(iTRAQ4plex	0.902	3.15	36	2	612.33
									VTTSQDMLSIMEK	1	N-Term(iTRAQ4plex); M7(Oxidation); K13(iTRAQ4plex)	0.75		57		
									FPVFMGR	1	N-Term(iTRAQ4plex	0.728		35		
47132547	FN1	fibronectin isoform 7 preproprotein	19.79	0	10	45		73.6								
									WCGTTQNYDADQK	9	N-Term(iTRAQ4plex); C2(Methylthio); K13(iTRAQ4plex)		6.24	91	2	932.42
									GFNCESKPEAEETCFDK	4	N-Term(iTRAQ4plex); C4(Methylthio); K7(iTRAQ4plex); C14(Methylthio); K17(iTRAQ4plex)		5.41	72	3	820.03
									HTSVQTTSSGSGPFTDVF	6	N-Term(iTRAQ4plex		5.31	102	3	670.00

									GNLLQCICTGNGR	2	N-Term(iTRAQ4plex); C6(Methylthio); C8(Methylthio)		4.53	67	2	792.86
									YSFCTDHTVLVQTF	5	N-Term(iTRAQ4plex); C4(Methylthio		4.23	80	2	930.45
									TFYSCTTEGR	5	N-Term(iTRAQ4plex); C5(Methylthio		4.08	48	2	677.80
									HYQINQQWER	3	N-Term(iTRAQ4plex		3.95	59	3	515.93
									GEWTCIAYSQLR	4	N-Term(iTRAQ4plex); C5(Methylthio		3.88	45	2	808.89
									IGDTWSK	6	N-Term(iTRAQ4plex); K7(iTRAQ4plex			56		
									TYLGNALVCTCYGGSF	1	N-Term(iTRAQ4plex); C9(Methylthio); C11(Methylthio)			31		
14149712	C1QTNF5	complement C1q tumor necrosis factor-related protein 5 precursor	19.75	3	3	12	0.986	25.3								
									VLVNEQGHYDAVTGK	6	N-Term(iTRAQ4plex); K15(iTRAQ4plex	1.046	6.34	92	3	640.02
									GEAGPAGPTGPAGECSVPPF	3	N-Term(iTRAQ4plex); C15(Methylthio	0.898	3.78	64	2	998.97
									VPPPSDAPLPFDR	3	N-Term(iTRAQ4plex	0.962	3.37	59	2	776.42
7706387	CPQ	plasma glutamate carboxypeptidase precursor	19.7	8	8	26	0.976	51.9								
									TYPDTDSFNTVAEITGSK	2	N-Term(iTRAQ4plex); K18(iTRAQ4plex	1.144	7.34	89	2	1117.56
									AIQIMYQNLQQDGLEK	3	N-Term(iTRAQ4plex); K16(iTRAQ4plex	1.206	4.68	63	2	1090.59
									IVVYNQPYINYSR	2	N-Term(iTRAQ4plex	0.89	4.66	52	2	886.98
									GEESAVMLEPR	2	N-Term(iTRAQ4plex	0.98	3.94	37	2	681.34
									LALLVDTVGPR	7	N-Term(iTRAQ4plex	0.931	3.71	66	2	649.41
									VGALASLIR	5	N-Term(iTRAQ4plex	0.936	3.63	51	2	522.34
									TQGAVEAAKVGALASLIF	2	N-Term(iTRAQ4plex); K9(iTRAQ4plex	0.666	3.52	63	3	681.74
									IVIQLK	3	N-Term(iTRAQ4plex); K6(iTRAQ4plex	1.041		43		
6912328	DDAH1	N(G),N(G)-dimethylarginine dimethylaminohydrolase 1 isoform 1	19.65	2	4	11	0.802	31.1								
									DENATLDGGDVLFTGF	4	N-Term(iTRAQ4plex		5.12	105	2	912.45
									QHQLYVGVLGSK	2	N-Term(iTRAQ4plex); K12(iTRAQ4plex	0.896	5.02	49	3	539.65
									ALPESLGQHALR	4	N-Term(iTRAQ4plex	0.724	4.53	67	3	479.28
									DYAVSTVPVADGLHLK	1	N-Term(iTRAQ4plex); K16(iTRAQ4plex			42		
209529703	SIRPB1	signal-regulatory protein beta-1 isoform 3 precursor	19.6	0	5	55		43.3								
									LTCQVEHDGQPAVSK	8	N-Term(iTRAQ4plex); C3(Methylthio); K15(iTRAQ4plex)		7.14	63	3	649.33
									VPPTLEVTQQPVF	35	N-Term(iTRAQ4plex		4.47	91	3	536.65
									VTTVSDLTK	7	N-Term(iTRAQ4plex); K9(iTRAQ4plex		4.15	66	2	626.37
									ATPQHTVSFTCESHGFSPF	3	N-Term(iTRAQ4plex); C11(Methylthio		3.79	66	3	760.35
									EDVHSQVICEVAHVTLQGDPLF	2	N-Term(iTRAQ4plex); C9(Methylthio		3.53	64	3	879.11
21735625	YWHAZ	14-3-3 protein zeta/delta	19.59	3	4	6	0.939	27.7								
									DSTLIMQLLR	2	N-Term(iTRAQ4plex		4.44	67	2	667.39
									SVTEQGAELSNEEF	2	N-Term(iTRAQ4plex	0.941	4.14	72	2	846.91
									FLIPNASQAESK	1	N-Term(iTRAQ4plex); K12(iTRAQ4plex	0.939		51		
68163411	ALCAM	CD166 antigen precursor	19.55	9	9	32	0.913	65.1								
									ESLTLIVEGKPQIK	2	N-Term(iTRAQ4plex); K10(iTRAQ4plex); K14(iTRAQ4plex)	0.899	5.64	27	3	663.08
									YEKPDGSPVFIAFR	4	N-Term(iTRAQ4plex); K3(iTRAQ4plex	0.963	5.1	54	2	957.52
									SVQYDDVPEYK	7	N-Term(iTRAQ4plex); K11(iTRAQ4plex	0.866	5.01	58	2	815.91
									ALFLETEQLK	7	N-Term(iTRAQ4plex); K10(iTRAQ4plex	0.954	4.53	65	2	740.44
									LDVPQNLMFGK	7	N-Term(iTRAQ4plex); K11(iTRAQ4plex	0.945	4.32	73	2	775.44
									QIGDALPVSCTISASR	2	N-Term(iTRAQ4plex); C10(Methylthio	0.845	4.2	63	2	904.47
									EMDPVTQLYTMTSTLEYK	1	N-Term(iTRAQ4plex); K18(iTRAQ4plex	1.021		45		
									VLHPLEGAVVIIFK	1	N-Term(iTRAQ4plex); K14(iTRAQ4plex	1.007		44		
									CSLIDK	1	N-Term(iTRAQ4plex); C1(Methylthio); K6(iTRAQ4plex)	0.918		42		
4502147	APLP2	amyloid-like protein 2 isoform 1	19.53	2	15	48	1.066	86.9								
									GSGVGEQDGGLIGAEEK	8	N-Term(iTRAQ4plex); K17(iTRAQ4plex	1.091	7.76	111	2	945.99
									SQVMTHLHVIEEF	5	N-Term(iTRAQ4plex		6.14	86	4	431.48
									QTLIQHFQAMVK	4	N-Term(iTRAQ4plex); K12(iTRAQ4plex		6.02	54	2	866.49
									KEWEEAELQAK	2	N-Term(iTRAQ4plex); K1(iTRAQ4plex); K11(iTRAQ4plex)		5.79	51	3	598.33
									LNMHVNIQTGK	4	N-Term(iTRAQ4plex); K11(iTRAQ4plex		5.31	67	3	514.96
									KGSGVGEQDGGLIGAEEK	2	N-Term(iTRAQ4plex); K1(iTRAQ4plex); K18(iTRAQ4plex)	0.883	5.22	71	3	721.73
									EWEEAELQAK	2	N-Term(iTRAQ4plex); K10(iTRAQ4plex		5.14	55	2	760.90
									VEAMLNDR	2	N-Term(iTRAQ4plex		3.42	43	2	546.28
									EMIFNAER	2	N-Term(iTRAQ4plex		3.38	43	2	577.29
									VSIDNWCR	4	N-Term(iTRAQ4plex); C7(Methylthio		3.26	41	2	591.78
									FIYGGCGGNR	2	N-Term(iTRAQ4plex); C6(Methylthio		3.14	32	2	617.28
									ADMDQFTASISETPVDVF	1	N-Term(iTRAQ4plex			59		
									MALENYLAALQSDPPRPHF	2	N-Term(iTRAQ4plex			52		
									WYFDLSK	3	N-Term(iTRAQ4plex); K7(iTRAQ4plex			52		
									FVTPFK	5	N-Term(iTRAQ4plex); K6(iTRAQ4plex			43		
187608363	CADM3	cell adhesion molecule 3 isoform 2	19.35	0	5	25		43.3								
									DHEDSSLQWSNPAQQTLYFGEK	4	N-Term(iTRAQ4plex); K22(iTRAQ4plex		5.09	74	3	956.79
									EDDGASIVCSVNHESLK	4	N-Term(iTRAQ4plex); C9(Methylthio); K17(iTRAQ4plex)		4.6	87	2	1069.01
									GNPVPQQYLWEK	10	N-Term(iTRAQ4plex); K12(iTRAQ4plex		4.58	73	2	873.97
									SLVTVLGIPQKPIITGYK	2	N-Term(iTRAQ4plex); K11(iTRAQ4plex); K18(iTRAQ4plex)		3.69	45	3	787.16
									LLLHCEGR	5	N-Term(iTRAQ4plex); C5(Methylthio			54		
221139785	SCG5	neuroendocrine protein 7B2 isoform 1	19.34	1	3	12	0.979	23.7								
									TADDGCLENTPDTAEFSF	2	N-Term(iTRAQ4plex); C6(Methylthio	0.979	5.43	90	2	1066.46
									SVPHFSDEDKDPE	9	N-Term(iTRAQ4plex); K10(iTRAQ4plex		5.1	51	3	597.29
									SVNPYLQGQR	1	N-Term(iTRAQ4plex			51		
194440697	LY6H	lymphocyte antigen 6H isoform b	19.25	0	3	13		17								
									QCQPSDTVCASVF	7	N-Term(iTRAQ4plex); C2(Methylthio); C9(Methylthio)		4.47	70	2	815.35
									MCASSCDFVK	4	N-Term(iTRAQ4plex); C2(Methylthio); C6(Methylthio); K10(iTRAQ4plex)		4.27	68	2	735.81
									VDVDCCEK	2	N-Term(iTRAQ4plex); C5(Methylthio); C6(Methylthio); K8(iTRAQ4plex)		3.21	39	2	645.78
41406057	APP	amyloid beta A4 protein isoform c precursor	19.14	0	10	96		78.6								
									YLETPGDENEHAHFQK	24	N-Term(iTRAQ4plex); K16(iTRAQ4plex		8.08	101	3	735.03
									ISYGNDALMPSLTETK	14	N-Term(iTRAQ4plex); K16(iTRAQ4plex		6.8	87	2	1014.53
									EQNYSDDVLANMISEPF	4	N-Term(iTRAQ4plex		5.55	96	2	1063.00
									VESLEQEAANEF	37	N-Term(iTRAQ4plex		5.46	76	2	759.88
									LVFFAEDVGSNK	2	N-Term(iTRAQ4plex); K12(iTRAQ4plex		4.68	73	2	807.45
									GLTTRPGSGLTNIK	2	N-Term(iTRAQ4plex); K14(iTRAQ4plex		4.53	39	2	852.01
									THPHFVIPYF	6	N-Term(iTRAQ4plex		4.38	48	3	470.93

									STNLHDYGMLLPCGIDK	2	N-Term(iTRAQ4plex); M9(Oxidation); C13(Methylthio); K17(iTRAQ4plex)		3.77	46	3	743.03
									VEAMLNDR	2	N-Term(iTRAQ4plex		3.42	43	2	546.28
									ISYGNDALMPSLTETK	2	N-Term(iTRAQ4plex); M9(Oxidation); K16(iTRAQ4plex)		3.36	68	2	1022.52
									LNMHMNVQNGK	1	N-Term(iTRAQ4plex); K11(iTRAQ4plex			57		
55956899	KRT9	keratin, type I cytoskeletal 9	19.1	10	11	32	1.288	62								
									VQALEEANNDLENK	5	N-Term(iTRAQ4plex); K14(iTRAQ4plex	1.611	5.4	94	2	937.99
									QVLDNLTMEK	3	N-Term(iTRAQ4plex); K10(iTRAQ4plex	1.407	4.89	63	2	739.91
									EIETYHNLLEGGQEDFESSGAGK	2	N-Term(iTRAQ4plex); K23(iTRAQ4plex	3.393	4.68	108	3	933.45
									QEYEQLIAK	2	N-Term(iTRAQ4plex); K9(iTRAQ4plex	1.074	4.22	52	2	705.40
									QVLDNLTMEK	4	N-Term(iTRAQ4plex); M8(Oxidation); K10(iTRAQ4plex)	1.207	4.07	48	2	747.91
									QGVDADINGLR	2	N-Term(iTRAQ4plex	1.288	4.07	65	2	651.35
									DQIVDLTVGNNK	2	N-Term(iTRAQ4plex); K12(iTRAQ4plex	1.832	3.84	72	2	802.45
									TLLDIDNTR	3	N-Term(iTRAQ4plex	1.147	3.45	42	2	602.84
									LASYLDK	4	N-Term(iTRAQ4plex); K7(iTRAQ4plex		3.35	53	2	549.32
									IQDWYDK	2	N-Term(iTRAQ4plex); K7(iTRAQ4plex	1.123	3.27	35	2	628.33
									IKFEMEQNLR	1	N-Term(iTRAQ4plex); K2(iTRAQ4plex); M5(Oxidation)	0.733		36		
									MTLDDFR	1	N-Term(iTRAQ4plex	1.499		35		
32455266	PRDX1	peroxiredoxin-1	19.1	2	4	12	0.877	22.1								
									QITVNDLPVGR	2	N-Term(iTRAQ4plex		4.31	52	2	678.39
									ATAVMPDGQFK	6	N-Term(iTRAQ4plex); K11(iTRAQ4plex	0.901	4.06	72	2	726.89
									DISLSDYK	2	N-Term(iTRAQ4plex); K8(iTRAQ4plex	0.854	3.88	49	2	614.84
									GLFIIDDK	2	N-Term(iTRAQ4plex); K8(iTRAQ4plex			55		
209915570	APP	amyloid beta A4 protein isoform e precursor	19.09	0	9	95		72.5								
									YLETPGDENEHAHFQK	24	N-Term(iTRAQ4plex); K16(iTRAQ4plex		8.08	101	3	735.03
									ISYGNDALMPSLTETK	14	N-Term(iTRAQ4plex); K16(iTRAQ4plex		6.8	87	2	1014.53
									EQNYSDDVLANMISEPR	4	N-Term(iTRAQ4plex		5.55	96	2	1063.00
									VESLEQEAANER	37	N-Term(iTRAQ4plex		5.46	76	2	759.88
									LVFFAEDVGSNK	2	N-Term(iTRAQ4plex); K12(iTRAQ4plex		4.68	73	2	807.45
									GLTTRPGSGLTNIK	2	N-Term(iTRAQ4plex); K14(iTRAQ4plex		4.53	39	2	852.01
									THPHFVIPYR	6	N-Term(iTRAQ4plex		4.38	48	3	470.93
									STNLHDYGMLLPCGIDK	2	N-Term(iTRAQ4plex); M9(Oxidation); C13(Methylthio); K17(iTRAQ4plex)		3.77	46	3	743.03
									VEAMLNDR	2	N-Term(iTRAQ4plex		3.42	43	2	546.28
									ISYGNDALMPSLTETK	2	N-Term(iTRAQ4plex); M9(Oxidation); K16(iTRAQ4plex)		3.36	68	2	1022.52
27894376	CHL1	neural cell adhesion molecule L1-like protein precursor	19.04	20	20	177	0.738	136.6								
									VDKDTATLSWGLPK	4	N-Term(iTRAQ4plex); K3(iTRAQ4plex); K14(iTRAQ4plex)	0.635	6.23	56	3	655.05
									LLLPPTESGSESSITILK	8	N-Term(iTRAQ4plex); K18(iTRAQ4plex	0.805	6.02	56	3	725.09
									VNGSPVDNHPFAGDVVFPR	7	N-Term(iTRAQ4plex	0.816	5.24	73	3	723.37
									VMTPAVYAPYDVK	22	N-Term(iTRAQ4plex); K13(iTRAQ4plex	0.79	5.22	99	2	871.48
									DTATLSWGLPK	6	N-Term(iTRAQ4plex); K11(iTRAQ4plex	0.805	5.2	83	2	738.92
									GDLYFANVEEK	28	N-Term(iTRAQ4plex); K11(iTRAQ4plex	0.807	5.06	75	2	786.91
									GNPEPTFSWTK	6	N-Term(iTRAQ4plex); K11(iTRAQ4plex	0.747	4.83	76	2	776.41
									VMTPAVYAPYDVK	4	N-Term(iTRAQ4plex); M2(Oxidation); K13(iTRAQ4plex)	0.754	4.72	84	2	879.47
									GYQINWWK	15	N-Term(iTRAQ4plex); K8(iTRAQ4plex	0.721	4.54	45	2	691.87
									SMEQNGPGLEYR	4	N-Term(iTRAQ4plex	0.7	4.11	70	2	762.87
									TAVTANLDIR	13	N-Term(iTRAQ4plex	0.724	4	60	2	609.35
									KTTVILPLAPFVR	5	N-Term(iTRAQ4plex); K1(iTRAQ4plex	0.738	3.98	49	3	581.71
									VTWSTVPK	10	N-Term(iTRAQ4plex); K8(iTRAQ4plex	0.746	3.94	42	2	603.36
									TTVILPLAPFVR	12	N-Term(iTRAQ4plex	0.635	3.89	53	2	735.96
									IENVSYQDK	4	N-Term(iTRAQ4plex); K9(iTRAQ4plex	0.774	3.68	64	2	692.38
									NDYCCFAAFPR	3	N-Term(iTRAQ4plex); C4(Methylthio); C5(Methylthio)	0.75	3.64	57	2	771.81
									DGNPFYFTDHR	22	N-Term(iTRAQ4plex	0.772	3.64	72	2	756.85
									VIAVNEVGR	1	N-Term(iTRAQ4plex	0.66		48		
									EKIDPLEVEEGDPIVLPCNPPK	1	N-Term(iTRAQ4plex); K2(iTRAQ4plex); C18(Methylthio); K22(iTRAQ4plex)			39		
									EEPGRWEELTR	1	N-Term(iTRAQ4plex	0.674		38		
									CTASNFLGTATHDFHVIVEEPPR	1	N-Term(iTRAQ4plex); C1(Methylthio	0.72		34		
14550407	C2	complement C2 isoform 1 preproprotein	19.02	0	12	57		83.2								
									RNDYLDIYAIGVGK	2	N-Term(iTRAQ4plex); K14(iTRAQ4plex		5.35	60	2	943.02
									CSSNLVLTGSSER	9	N-Term(iTRAQ4plex); C1(Methylthio		4.78	83	2	771.87
									KNQGILEFYGDDIALLK	2	N-Term(iTRAQ4plex); K1(iTRAQ4plex); K17(iTRAQ4plex)		4.74	70	3	790.46
									GESGGAVFLER	2	N-Term(iTRAQ4plex		4.67	60	2	633.33
									AVISPGFDVFAK	6	N-Term(iTRAQ4plex); K12(iTRAQ4plex		4.33	101	2	769.94
									VLMSVLNDNSR	10	N-Term(iTRAQ4plex		4.05	64	2	696.38
									HAIILLTDGK	4	N-Term(iTRAQ4plex); K10(iTRAQ4plex		3.99	49	2	684.93
									ALHQVFEHMLDVSK	8	N-Term(iTRAQ4plex); K14(iTRAQ4plex		3.9	68	3	648.02
									DMTEVISSLENANYK	2	N-Term(iTRAQ4plex); K15(iTRAQ4plex		3.8	73	2	1001.51
									EILNINQK	6	N-Term(iTRAQ4plex); K8(iTRAQ4plex		3.66	46	2	630.38
									QHLGDVLNFLPL	4	N-Term(iTRAQ4plex		3.5	73	2	755.43
									LNINLK	2	N-Term(iTRAQ4plex); K6(iTRAQ4plex			41		
89903008	NFASC	neurofascin isoform 4 precursor	18.99	0	20	62		131.6								
									VIAINEVGSSHPSLPSER	4	N-Term(iTRAQ4plex		7.15	74	3	679.37
									EDDSLTIFGVAER	4	N-Term(iTRAQ4plex		5.45	81	2	798.41
									SGGRPEEYEGEYQCFAR	2	N-Term(iTRAQ4plex); C14(Methylthio		5.29	35	3	723.32
									KEDDSLTIFGVAER	2	N-Term(iTRAQ4plex); K1(iTRAQ4plex		5.08	56	3	623.34
									YVVGQTPVYVPYEIR	4	N-Term(iTRAQ4plex		4.91	65	2	964.03
									AAPYWLDEPK	9	N-Term(iTRAQ4plex); K10(iTRAQ4plex		4.68	76	2	739.40
									LDCPFFGSPIPTLR	2	N-Term(iTRAQ4plex); C3(Methylthio		4.57	53	2	876.95
									GTTVQLECR	2	N-Term(iTRAQ4plex); C8(Methylthio		4.52	46	2	598.80
									DDEPLYIGNR	2	N-Term(iTRAQ4plex		4.14	34	2	668.34
									EVAGDTIIFR	2	N-Term(iTRAQ4plex		4.13	55	2	632.86
									EFTTPEGVPSAPR	2	N-Term(iTRAQ4plex		4.1	65	2	766.40
									DLELTDLAER	5	N-Term(iTRAQ4plex		3.98	47	2	659.85
									NLILAPGEDGR	4	N-Term(iTRAQ4plex		3.79	50	2	649.86
									GNPAPSFHWTR	2	N-Term(iTRAQ4plex		3.37	38	2	707.36
									SGTLVIDFR	2	N-Term(iTRAQ4plex		3.22	41	2	576.33
									VYSDTVQGQLR	1	N-Term(iTRAQ4plex			61		
									GPEPESVIGYSGEDYPR	1	N-Term(iTRAQ4plex			56		

									LTVSWLK	8	N-Term(iTRAQ4plex); K7(iTRAQ4plex			53		
									FENFNK	3	N-Term(iTRAQ4plex); K6(iTRAQ4plex			37		
4506917	SCG5	neuroendocrine protein 7B2 isoform 2	18.96	1	3	12	0.897	23.6								
									SVPHFSDEDKDPE	9	N-Term(iTRAQ4plex); K10(iTRAQ4plex		5.1	51	3	597.29
									SVNPYLQGQR	1	N-Term(iTRAQ4plex			51		
									TDDGCLENTPDTAEFSF	2	N-Term(iTRAQ4plex); C5(Methylthio	0.897		49		
237858679	NFASC	neurofascin isoform 3 precursor	18.91	0	20	62		132								
									VIAINEVGSSHPSLPSEF	4	N-Term(iTRAQ4plex		7.15	74	3	679.37
									EDDSLTIFGVAER	4	N-Term(iTRAQ4plex		5.45	81	2	798.41
									SGGRPEEYEGEYQCFAR	2	N-Term(iTRAQ4plex); C14(Methylthio		5.29	35	3	723.32
									KEDDSLTIFGVAER	2	N-Term(iTRAQ4plex); K1(iTRAQ4plex		5.08	56	3	623.34
									YVVGQTPVYVPYEIF	4	N-Term(iTRAQ4plex		4.91	65	2	964.03
									AAPYWLDEPK	9	N-Term(iTRAQ4plex); K10(iTRAQ4plex		4.68	76	2	739.40
									LDCPFFGSPIPTLR	2	N-Term(iTRAQ4plex); C3(Methylthio		4.57	53	2	876.95
									GTTVQLECR	2	N-Term(iTRAQ4plex); C8(Methylthio		4.52	46	2	598.80
									DDEPLYIGNR	2	N-Term(iTRAQ4plex		4.14	34	2	668.34
									EVAGDTIIFR	2	N-Term(iTRAQ4plex		4.13	55	2	632.86
									EFTTPEGVPSAPR	2	N-Term(iTRAQ4plex		4.1	65	2	766.40
									DLELTDLAER	5	N-Term(iTRAQ4plex		3.98	47	2	659.85
									NLILAPGEDGR	4	N-Term(iTRAQ4plex		3.79	50	2	649.86
									GNPAPSFHWTR	2	N-Term(iTRAQ4plex		3.37	38	2	707.36
									SGTLVIDFR	2	N-Term(iTRAQ4plex		3.22	41	2	576.33
									VYSDTVQGQLR	1	N-Term(iTRAQ4plex			61		
									GPEPESVIGYSGEDYPR	1	N-Term(iTRAQ4plex			56		
									LTVSWLK	8	N-Term(iTRAQ4plex); K7(iTRAQ4plex			53		
									FENFNK	3	N-Term(iTRAQ4plex); K6(iTRAQ4plex			37		
41406055	APP	amyloid beta A4 protein isoform b precursor	18.91	0	11	97		84.8								
									YLETPGDENEHAHFQK	24	N-Term(iTRAQ4plex); K16(iTRAQ4plex		8.08	101	3	735.03
									ISYGNDALMPSLTETK	14	N-Term(iTRAQ4plex); K16(iTRAQ4plex		6.8	87	2	1014.53
									EQNYSDDVLANMISEPF	4	N-Term(iTRAQ4plex		5.55	96	2	1063.00
									VESLEQEAANEF	37	N-Term(iTRAQ4plex		5.46	76	2	759.88
									LVFFAEDVGSNK	2	N-Term(iTRAQ4plex); K12(iTRAQ4plex		4.68	73	2	807.45
									GLTTRPGSGLTNIK	2	N-Term(iTRAQ4plex); K14(iTRAQ4plex		4.53	39	2	852.01
									THPHFVIPYF	6	N-Term(iTRAQ4plex		4.38	48	3	470.93
									STNLHDYGMLLPCGIDK	2	N-Term(iTRAQ4plex); M9(Oxidation); C13(Methylthio); K17(iTRAQ4plex)		3.77	46	3	743.03
									VEAMLNDR	2	N-Term(iTRAQ4plex		3.42	43	2	546.28
									ISYGNDALMPSLTETK	2	N-Term(iTRAQ4plex); M9(Oxidation); K16(iTRAQ4plex)		3.36	68	2	1022.52
									WYFDVTEGK	1	N-Term(iTRAQ4plex); K9(iTRAQ4plex			62		
									LNMHMNVQNGK	1	N-Term(iTRAQ4plex); K11(iTRAQ4plex			57		
4826772	IGFALS	insulin-like growth factor-binding protein complex acid labile subunit isoform 2 precursor	18.84	0	9	21		66								
									DLHFLEELQLGHNR	2	N-Term(iTRAQ4plex		5.73	62	3	622.33
									VAGLLEDTFPGLLGLR	3	N-Term(iTRAQ4plex		4.81	85	2	908.03
									DFALQNPSAVPF	3	N-Term(iTRAQ4plex		4.61	63	2	729.90
									LAELPADALGPLQR	2	N-Term(iTRAQ4plex		3.27	76	2	804.47
									WLDLSHNR	3	N-Term(iTRAQ4plex			53		
									AFWLDVSHNR	4	N-Term(iTRAQ4plex			53		
									TFTPQPPGLER	2	N-Term(iTRAQ4plex			52		
									SFEGLGQLEVLTLDHNQLQEVK	1	N-Term(iTRAQ4plex); K22(iTRAQ4plex			35		
4502511	C9	complement component C9 precursor	18.78	9	11	80	0.979	63.1								
									TSNFNAAISLK	12	N-Term(iTRAQ4plex); K11(iTRAQ4plex	0.953	6.07	68	2	727.42
									TEHYEEQIEAFK	9	N-Term(iTRAQ4plex); K12(iTRAQ4plex	0.973	6.04	70	2	906.46
									DVVLTTTFVDDIK	6	N-Term(iTRAQ4plex); K13(iTRAQ4plex	1.017	5.4	94	2	877.49
									LSPIYNLVPVK	13	N-Term(iTRAQ4plex); K11(iTRAQ4plex	1.016	5.07	64	2	765.98
									AIEDYINEFSVR	13	N-Term(iTRAQ4plex	0.937	4.98	75	2	800.41
									RPWNVASLIYETK	14	N-Term(iTRAQ4plex); K13(iTRAQ4plex	1.14	4.59	59	3	622.36
									FEGIACEISK	6	N-Term(iTRAQ4plex); C6(Methylthio); K10(iTRAQ4plex)	1.009	4.23	69	2	715.87
									KYAFELK	3	N-Term(iTRAQ4plex); K1(iTRAQ4plex); K7(iTRAQ4plex)		4.1	49	3	444.28
									ALPTTYEK	1	N-Term(iTRAQ4plex); K8(iTRAQ4plex	0.713		48		
									YAFELK	2	N-Term(iTRAQ4plex); K6(iTRAQ4plex			42		
									CLCACPFK	1	N-Term(iTRAQ4plex); C1(Methylthio); C3(Methylthio); C5(Methylthio); K8(iTRAQ4plex)	1.123		24		
33519481	NCAM2	neural cell adhesion molecule 2 precursor	18.76	14	14	80	0.92	93								
									LTIYNANIEDAGIYR	14	N-Term(iTRAQ4plex	0.856	5.8	67	2	935.50
									MILEIAPTSDNDFGR	8	N-Term(iTRAQ4plex	0.968	5.39	97	2	911.96
									ASGSPEPAISWFR	4	N-Term(iTRAQ4plex	0.984	5.03	86	2	774.90
									GQTQEATVVLEIYQK	2	N-Term(iTRAQ4plex); K15(iTRAQ4plex	0.824	4.87	67	2	998.06
									SMYLDIEYAPK	11	N-Term(iTRAQ4plex); K11(iTRAQ4plex	0.92	4.72	74	2	809.43
									DKEDQWLEK	2	N-Term(iTRAQ4plex); K2(iTRAQ4plex); K9(iTRAQ4plex)	0.97	4.36	36	2	811.95
									SMYLDIEYAPK	2	N-Term(iTRAQ4plex); M2(Oxidation); K11(iTRAQ4plex)	0.953	4.31	58	2	817.43
									IIELSQTTAK	6	N-Term(iTRAQ4plex); K10(iTRAQ4plex	0.936	4.14	70	2	696.42
									EVASEIWK	8	N-Term(iTRAQ4plex); K8(iTRAQ4plex	0.833	4.12	52	2	625.35
									EVVSPQEFK	6	N-Term(iTRAQ4plex); K9(iTRAQ4plex	0.973	4.02	45	2	675.88
									QDDGGAPILEYIVK	5	N-Term(iTRAQ4plex); K14(iTRAQ4plex	1.034	3.92	82	2	903.50
									IEIFQTLPVR	5	N-Term(iTRAQ4plex	0.863	3.75	50	2	680.41
									NIINSDGGPYVCR	2	N-Term(iTRAQ4plex); C12(Methylthio	0.926	3.74	38	2	799.39
									GEEMTFSCR	1	N-Term(iTRAQ4plex); C8(Methylthio	0.873		38		
									LVLPAK	4	N-Term(iTRAQ4plex); K6(iTRAQ4plex	0.902		33		
29550838	GOLM1	Golgi membrane protein 1	18.7	6	6	16	0.978	45.3								
									EETNEIQVVNEEPQF	3	N-Term(iTRAQ4plex	0.948	5.74	88	2	979.48
									LQAAGLPHTEVPQGK	2	N-Term(iTRAQ4plex); K15(iTRAQ4plex	1.086	4.71	43	3	612.02
									DQLVIPDGQEEEQEAAGEGF	6	N-Term(iTRAQ4plex	0.797	4.28	72	2	1157.55
									NIDVFNVEDQKF	2	N-Term(iTRAQ4plex); K11(iTRAQ4plex	0.989	3.82	64	3	588.99
									DTINLLDQR	2	N-Term(iTRAQ4plex	0.998	3.62	53	2	616.34
									QVEKEETNEIQVVNEEPQF	1	N-Term(iTRAQ4plex); K4(iTRAQ4plex	1.136		38		
237858677	NFASC	neurofascin isoform 2 precursor	18.67	0	20	62		133.6								
									VIAINEVGSSHPSLPSEF	4	N-Term(iTRAQ4plex		7.15	74	3	679.37
									EDDSLTIFGVAER	4	N-Term(iTRAQ4plex		5.45	81	2	798.41
									SGGRPEEYEGEYQCFAR	2	N-Term(iTRAQ4plex); C14(Methylthio		5.29	35	3	723.32
									KEDDSLTIFGVAER	2	N-Term(iTRAQ4plex); K1(iTRAQ4plex		5.08	56	3	623.34
									YVVGQTPVYVPYEIF	4	N-Term(iTRAQ4plex		4.91	65	2	964.03

									AAPYWLDEPK	9	N-Term(iTRAQ4plex); K10(iTRAQ4plex		4.68	76	2	739.40
									LDCPFFGSPIPTLR	2	N-Term(iTRAQ4plex); C3(Methylthio		4.57	53	2	876.95
									GTTVQLECR	2	N-Term(iTRAQ4plex); C8(Methylthio		4.52	46	2	598.80
									DDEPLYIGNR	2	N-Term(iTRAQ4plex		4.14	34	2	668.34
									EVAGDTIIFR	2	N-Term(iTRAQ4plex		4.13	55	2	632.86
									EFTTPEGVPSAPR	2	N-Term(iTRAQ4plex		4.1	65	2	766.40
									DLELTDLAER	5	N-Term(iTRAQ4plex		3.98	47	2	659.85
									NLILAPGEDGR	4	N-Term(iTRAQ4plex		3.79	50	2	649.86
									GNPAPSFHWTR	2	N-Term(iTRAQ4plex		3.37	38	2	707.36
									SGTLVIDFR	2	N-Term(iTRAQ4plex		3.22	41	2	576.33
									VYSDTVQGQLR	1	N-Term(iTRAQ4plex			61		
									GPEPESVIGYSGEDYPR	1	N-Term(iTRAQ4plex			56		
									LTVSWLK	8	N-Term(iTRAQ4plex); K7(iTRAQ4plex			53		
									FENFNK	3	N-Term(iTRAQ4plex); K6(iTRAQ4plex			37		
4501885	ACTB	actin, cytoplasmic 1	18.67	0	6	26		41.7								
									VAPEEHPVLLTEAPLNPK	10	N-Term(iTRAQ4plex); K18(iTRAQ4plex		6.06	76	3	748.09
									EITALAPSTMK	3	N-Term(iTRAQ4plex); K11(iTRAQ4plex		4.19	66	2	725.42
									DLTDYLMK	5	N-Term(iTRAQ4plex); K8(iTRAQ4plex		3.98	48	2	643.85
									SYELPDGQVITIGNER	3	N-Term(iTRAQ4plex		3.27	49	2	968.00
									EITALAPSTMK	1	N-Term(iTRAQ4plex); M10(Oxidation); K11(iTRAQ4plex)			43		
									IWHHTFYNELR	3	N-Term(iTRAQ4plex			41		
									GILTLK	1	N-Term(iTRAQ4plex); K6(iTRAQ4plex			31		
4501887	ACTG1	actin, cytoplasmic 2	18.67	0	6	26		41.8								
									VAPEEHPVLLTEAPLNPK	10	N-Term(iTRAQ4plex); K18(iTRAQ4plex		6.06	76	3	748.09
									EITALAPSTMK	3	N-Term(iTRAQ4plex); K11(iTRAQ4plex		4.19	66	2	725.42
									DLTDYLMK	5	N-Term(iTRAQ4plex); K8(iTRAQ4plex		3.98	48	2	643.85
									SYELPDGQVITIGNER	3	N-Term(iTRAQ4plex		3.27	49	2	968.00
									EITALAPSTMK	1	N-Term(iTRAQ4plex); M10(Oxidation); K11(iTRAQ4plex)			43		
									IWHHTFYNELR	3	N-Term(iTRAQ4plex			41		
									GILTLK	1	N-Term(iTRAQ4plex); K6(iTRAQ4plex			31		
295842514	DBI	acyl-CoA-binding protein isoform 5	18.6	0	2	8		14.4								
									TKPSDEEMLFIYGHYK	2	N-Term(iTRAQ4plex); K2(iTRAQ4plex); K16(iTRAQ4plex)		6.37	55	3	797.42
									WDAWNELK	6	N-Term(iTRAQ4plex); K8(iTRAQ4plex		3.93	46	2	675.36
4502167	APP	amyloid beta A4 protein isoform a precursor	18.44	0	11	97		86.9								
									YLETPGDENEHAHFQK	24	N-Term(iTRAQ4plex); K16(iTRAQ4plex		8.08	101	3	735.03
									ISYGNDALMPSLTETK	14	N-Term(iTRAQ4plex); K16(iTRAQ4plex		6.8	87	2	1014.53
									EQNYSDDVLANMISEPR	4	N-Term(iTRAQ4plex		5.55	96	2	1063.00
									VESLEQEAANER	37	N-Term(iTRAQ4plex		5.46	76	2	759.88
									LVFFAEDVGSNK	2	N-Term(iTRAQ4plex); K12(iTRAQ4plex		4.68	73	2	807.45
									GLTTRPGSGLTNIK	2	N-Term(iTRAQ4plex); K14(iTRAQ4plex		4.53	39	2	852.01
									THPHFVIPYR	6	N-Term(iTRAQ4plex		4.38	48	3	470.93
									STNLHDYGMLLPCGIDK	2	N-Term(iTRAQ4plex); M9(Oxidation); C13(Methylthio); K17(iTRAQ4plex)		3.77	46	3	743.03
									VEAMLNDR	2	N-Term(iTRAQ4plex		3.42	43	2	546.28
									ISYGNDALMPSLTETK	2	N-Term(iTRAQ4plex); M9(Oxidation); K16(iTRAQ4plex)		3.36	68	2	1022.52
									WYFDVTEGK	1	N-Term(iTRAQ4plex); K9(iTRAQ4plex			62		
									LNMHMNVQNGK	1	N-Term(iTRAQ4plex); K11(iTRAQ4plex			57		
270132876	PLG	plasminogen isoform 2 precursor	18.38	0	3	27		15.4								
									EQQCVIMAENR	9	N-Term(iTRAQ4plex); C4(Methylthio		5.04	57	2	755.85
									DVVLFEK	17	N-Term(iTRAQ4plex); K7(iTRAQ4plex		3.4	42	2	569.34
									VYLSECK	1	N-Term(iTRAQ4plex); C6(Methylthio); K7(iTRAQ4plex)			36		
209915573	APP	amyloid beta A4 protein isoform f precursor	18.35	0	10	96		80.8								
									YLETPGDENEHAHFQK	24	N-Term(iTRAQ4plex); K16(iTRAQ4plex		8.08	101	3	735.03
									ISYGNDALMPSLTETK	14	N-Term(iTRAQ4plex); K16(iTRAQ4plex		6.8	87	2	1014.53
									EQNYSDDVLANMISEPR	4	N-Term(iTRAQ4plex		5.55	96	2	1063.00
									VESLEQEAANER	37	N-Term(iTRAQ4plex		5.46	76	2	759.88
									LVFFAEDVGSNK	2	N-Term(iTRAQ4plex); K12(iTRAQ4plex		4.68	73	2	807.45
									GLTTRPGSGLTNIK	2	N-Term(iTRAQ4plex); K14(iTRAQ4plex		4.53	39	2	852.01
									THPHFVIPYR	6	N-Term(iTRAQ4plex		4.38	48	3	470.93
									STNLHDYGMLLPCGIDK	2	N-Term(iTRAQ4plex); M9(Oxidation); C13(Methylthio); K17(iTRAQ4plex)		3.77	46	3	743.03
									VEAMLNDR	2	N-Term(iTRAQ4plex		3.42	43	2	546.28
									ISYGNDALMPSLTETK	2	N-Term(iTRAQ4plex); M9(Oxidation); K16(iTRAQ4plex)		3.36	68	2	1022.52
									WYFDVTEGK	1	N-Term(iTRAQ4plex); K9(iTRAQ4plex			62		
34734062	FBLN1	fibulin-1 isoform C precursor	18.3	2	9	82	0.793	74.4								
									SQETGDLDVGGLQETDK	9	N-Term(iTRAQ4plex); K17(iTRAQ4plex		6.58	93	2	1040.52
									IIEVEEEQEDPYLNDR	24	N-Term(iTRAQ4plex		6.37	91	3	712.35
									VSPHSGVVALTKPVPEPR	2	N-Term(iTRAQ4plex); K12(iTRAQ4plex	0.793	6.01	35	4	540.32
									ITYYHLSFPTNIQAPAVVFR	5	N-Term(iTRAQ4plex	0.745	4.88	67	3	827.79
									CVDVDECAPPAEPCGK	2	N-Term(iTRAQ4plex); C1(Methylthio); C7(Methylthio); C14(Methylthio); K16(iTRAQ4plex)		4.79	65	2	1029.92
									TGYYFDGISR	12	N-Term(iTRAQ4plex		4.28	44	2	661.83
									DCSLPYATESK	4	N-Term(iTRAQ4plex); C2(Methylthio); K11(iTRAQ4plex)		4.16	66	2	774.37
									MCVDVNECQR	9	N-Term(iTRAQ4plex); C2(Methylthio); C8(Methylthio)		3.69	55	2	716.79
									DIILTVK	34	N-Term(iTRAQ4plex); K7(iTRAQ4plex		3.58	46	2	545.36
86788015	EFEMP1	EGF-containing fibulin-like extracellular matrix protein 1 precursor	18.26	7	7	34	1.042	54.6								
									QTSPVSAMLVLVK	9	N-Term(iTRAQ4plex); K13(iTRAQ4plex	1.009	6.51	69	2	831.00
									NPCQDPYILTPENR	7	N-Term(iTRAQ4plex); C3(Methylthio	1.072	5.26	57	2	925.44
									QTSPVSAMLVLVK	2	N-Term(iTRAQ4plex); M8(Oxidation); K13(iTRAQ4plex)	1	5.16	54	2	839.00
									LNCEDIDECR	2	N-Term(iTRAQ4plex); C3(Methylthio); C9(Methylthio)	1.034	4.43	49	2	723.29
									ELPQSIVYK	8	N-Term(iTRAQ4plex); K9(iTRAQ4plex	1.152	4.06	64	2	682.91
									GSFACQCPPGYQK	4	N-Term(iTRAQ4plex); C5(Methylthio); C7(Methylthio); K13(iTRAQ4plex)	1.042		40		
									EHIVDLEMLTVSSIGTFR	1	N-Term(iTRAQ4plex			35		
									FSCMCPQGYQVVR	1	N-Term(iTRAQ4plex); C3(Methylthio); C5(Methylthio)	0.899		32		

29029552	CECR1	adenosine deaminase CECR1 isoform b	18.15	0	4	7		30.6								
									DVAVIAESIR	2	N-Term(iTRAQ4plex		4.24	41	2	608.85
									LPYFFHAGETDWQGTSIDR	2	N-Term(iTRAQ4plex		3.59	45	3	795.39
									FIADVATK	2	N-Term(iTRAQ4plex); K8(iTRAQ4plex		3.25	55	2	576.85
									FVETHPEFIGIK	1	N-Term(iTRAQ4plex); K12(iTRAQ4plex			39		
310128738	LOC652797	PREDICTED: pyruvate kinase isozymes M1/M2-like isoform 5, partial	18.14	0	3	17		23.5								
									KGVNLPGAAVDLPAVSEK	4	N-Term(iTRAQ4plex); K1(iTRAQ4plex); K18(iTRAQ4plex)		5.68	69	3	733.11
									ITLDNAYMEK	12	N-Term(iTRAQ4plex); K10(iTRAQ4plex		4.74	74	2	743.40
									CDENILWLDYK	1	N-Term(iTRAQ4plex); C1(Methylthio); K11(iTRAQ4plex)			55		
4885049	ACTC1	actin, alpha cardiac muscle 1 proprotein	18.04	0	6	20		42								
									EITALAPSTMK	3	N-Term(iTRAQ4plex); K11(iTRAQ4plex		4.19	66	2	725.42
									DLTDYLMK	5	N-Term(iTRAQ4plex); K8(iTRAQ4plex		3.98	48	2	643.85
									YPIEHGIITNWDDMEK	4	N-Term(iTRAQ4plex); K16(iTRAQ4plex		3.37	54	3	750.38
									SYELPDGQVITIGNER	3	N-Term(iTRAQ4plex		3.27	49	2	968.00
									EITALAPSTMK	1	N-Term(iTRAQ4plex); M10(Oxidation); K11(iTRAQ4plex)			43		
									IWHHTFYNELR	3	N-Term(iTRAQ4plex			41		
									GILTLK	1	N-Term(iTRAQ4plex); K6(iTRAQ4plex			31		
4501881	ACTA1	actin, alpha skeletal muscle	18.04	0	6	20		42								
									EITALAPSTMK	3	N-Term(iTRAQ4plex); K11(iTRAQ4plex		4.19	66	2	725.42
									DLTDYLMK	5	N-Term(iTRAQ4plex); K8(iTRAQ4plex		3.98	48	2	643.85
									YPIEHGIITNWDDMEK	4	N-Term(iTRAQ4plex); K16(iTRAQ4plex		3.37	54	3	750.38
									SYELPDGQVITIGNER	3	N-Term(iTRAQ4plex		3.27	49	2	968.00
									EITALAPSTMK	1	N-Term(iTRAQ4plex); M10(Oxidation); K11(iTRAQ4plex)			43		
									IWHHTFYNELR	3	N-Term(iTRAQ4plex			41		
									GILTLK	1	N-Term(iTRAQ4plex); K6(iTRAQ4plex			31		
7706244	CUTA	protein CutA isoform 2	17.95	0	3	15		16.8								
									IEEDSEVLMMIK	2	N-Term(iTRAQ4plex); K12(iTRAQ4plex		5.39	37	2	862.96
									TQSSLVPALTDFVR	8	N-Term(iTRAQ4plex		5.17	88	2	839.47
									GKIEEDSEVLMMIK	5	N-Term(iTRAQ4plex); K2(iTRAQ4plex); K14(iTRAQ4plex)		3.95	74	2	1027.56
11056046	CADM3	cell adhesion molecule 3 isoform 1	17.82	0	5	25		47								
									DHEDSSLQWSNPAQQTLYFGEK	4	N-Term(iTRAQ4plex); K22(iTRAQ4plex		5.09	74	3	956.79
									EDDGASIVCSVNHESLK	4	N-Term(iTRAQ4plex); C9(Methylthio); K17(iTRAQ4plex)		4.6	87	2	1069.01
									GNPVPQQYLWEK	10	N-Term(iTRAQ4plex); K12(iTRAQ4plex		4.58	73	2	873.97
									SLVTVLGIPQKPIITGYK	2	N-Term(iTRAQ4plex); K11(iTRAQ4plex); K18(iTRAQ4plex)		3.69	45	3	787.16
									LLLHCEGR	5	N-Term(iTRAQ4plex); C5(Methylthio			54		
4502931	CNTFR	ciliary neurotrophic factor receptor subunit alpha preproprotein	17.74	4	4	13	0.706	40.6								
									RLEVTWQTPSTWPDPESFPLK	2	N-Term(iTRAQ4plex); K21(iTRAQ4plex	0.748	4.39	60	3	934.83
									YMHLFSTIK	2	N-Term(iTRAQ4plex); K9(iTRAQ4plex	0.973	4.02	38	3	476.60
									HQVLLHVGLPPR	8	N-Term(iTRAQ4plex	0.666	3.74	50	4	378.23
									DNEIGTWSDWSVAAHATPWTEEPR	1	N-Term(iTRAQ4plex	0.467		58		
225579152	IGFALS	insulin-like growth factor-binding protein complex acid labile subunit isoform 1 precursor	17.73	0	9	21		70.2								
									DLHFLEELQLGHNR	2	N-Term(iTRAQ4plex		5.73	62	3	622.33
									VAGLLEDTFPGLLGLR	3	N-Term(iTRAQ4plex		4.81	85	2	908.03
									DFALQNPSAVPR	3	N-Term(iTRAQ4plex		4.61	63	2	729.90
									LAELPADALGPLQR	2	N-Term(iTRAQ4plex		3.27	76	2	804.47
									WLDLSHNR	3	N-Term(iTRAQ4plex			53		
									AFWLDVSHNR	4	N-Term(iTRAQ4plex			53		
									TFTPQPPGLER	2	N-Term(iTRAQ4plex			52		
									SFEGLGQLEVLTLDHNQLQEVK	1	N-Term(iTRAQ4plex); K22(iTRAQ4plex			35		
148664211	CADM1	cell adhesion molecule 1 isoform 2	17.63	0	5	33		45.6								
									SDDSVIQLLNPNR	16	N-Term(iTRAQ4plex		5.54	82	2	807.93
									CEASNIVGK	2	N-Term(iTRAQ4plex); C1(Methylthio); K9(iTRAQ4plex)		4.25	64	2	627.83
									NLMIDIQK	11	N-Term(iTRAQ4plex); K8(iTRAQ4plex		4.14	59	2	631.88
									NLMIDIQK	2	N-Term(iTRAQ4plex); M3(Oxidation); K8(iTRAQ4plex)		3.39	44	2	639.86
									EDDGVPVICQVEHPAVTGNLQTQR	1	N-Term(iTRAQ4plex); C9(Methylthio			34		
									SEVEEWSDMYTVTSQLMLK	1	N-Term(iTRAQ4plex); K19(iTRAQ4plex			33		
4557391	C8B	complement component C8 beta chain preproprotein	17.6	10	10	42	0.921	66.9								
									KPYNVESYTPQTQGK	6	N-Term(iTRAQ4plex); K1(iTRAQ4plex); K15(iTRAQ4plex)	0.942	6.21	57	3	724.73
									IPGIFELGISSQSDR	2	N-Term(iTRAQ4plex	0.788	4.46	80	2	881.98
									RLPLEYSYGEYR	2	N-Term(iTRAQ4plex	0.854	4.28	50	3	563.96
									SLMLHYEFLQR	3	N-Term(iTRAQ4plex	0.95	4.2	54	2	790.92
									EYESYSDFER	3	N-Term(iTRAQ4plex	0.877	3.96	71	2	734.82
									DTMVEDLVVLVR	3	N-Term(iTRAQ4plex	0.892	3.91	60	2	766.93
									QALEEFQK	3	N-Term(iTRAQ4plex); K8(iTRAQ4plex	0.853	3.88	51	2	640.85
									LPLEYSYGEYR	5	N-Term(iTRAQ4plex	0.966	3.56	53	2	767.39
									SLMLHYEFLQR	2	N-Term(iTRAQ4plex); M3(Oxidation	0.915	3.54	41	3	532.95
									SGFSFGFK	12	N-Term(iTRAQ4plex); K8(iTRAQ4plex	0.968	3.4	54	2	582.82
55770878	NPTX1	neuronal pentraxin-1 precursor	17.59	6	6	30	0.919	47.1								
									LTPGEVYNLATCSTK	6	N-Term(iTRAQ4plex); C12(Methylthio); K15(iTRAQ4plex)	1.005	4.99	72	2	966.00
									IETALTSLHQR	12	N-Term(iTRAQ4plex	0.918	4.96	69	3	471.60
									LENLEQYSR	7	N-Term(iTRAQ4plex	0.932	4.34	43	2	648.34
									CESQSTLDPGAGEAR	3	N-Term(iTRAQ4plex); C1(Methylthio	0.687	3.95	81	2	855.88
									TPAAETLSQLGQTLQSLK	1	N-Term(iTRAQ4plex); K18(iTRAQ4plex	1.149		41		
197313763	DDAH1	N(G),N(G)-dimethylarginine dimethylaminohydrolase 1 isoform 2	17.58	0	2	5		20.2								
									DENATLDGGDVLFTGR	4	N-Term(iTRAQ4plex		5.12	105	2	912.45
									DYAVSTVPVADGLHLK	1	N-Term(iTRAQ4plex); K16(iTRAQ4plex			42		
214010181	APLP2	amyloid-like protein 2 isoform 2	17.44	0	13	38		85.4								
									SQVMTHLHVIEER	5	N-Term(iTRAQ4plex		6.14	86	4	431.48
									QTLIQHFQAMVK	4	N-Term(iTRAQ4plex); K12(iTRAQ4plex		6.02	54	2	866.49
									KEWEEAELQAK	2	N-Term(iTRAQ4plex); K1(iTRAQ4plex); K11(iTRAQ4plex)		5.79	51	3	598.33
									LNMHVNIQTGK	4	N-Term(iTRAQ4plex); K11(iTRAQ4plex		5.31	67	3	514.96

									EWEEAELQAK	2	N-Term(iTRAQ4plex); K10(iTRAQ4plex		5.14	55	2	760.90
									VEAMLNDR	2	N-Term(iTRAQ4plex		3.42	43	2	546.28
									EMIFNAER	2	N-Term(iTRAQ4plex		3.38	43	2	577.29
									VSIDNWCR	4	N-Term(iTRAQ4plex); C7(Methylthio		3.26	41	2	591.78
									FIYGGCGGNR	2	N-Term(iTRAQ4plex); C6(Methylthio		3.14	32	2	617.28
									ADMDQFTASISETPVDVF	1	N-Term(iTRAQ4plex			59		
									MALENYLAALQSDPPRPHF	2	N-Term(iTRAQ4plex			52		
									WYFDLSK	3	N-Term(iTRAQ4plex); K7(iTRAQ4plex			52		
									FVTPFK	5	N-Term(iTRAQ4plex); K6(iTRAQ4plex			43		
153266841	APOH	beta-2-glycoprotein 1 precursor	17.39	5	5	15	0.997	38.3								
									TCPKPDDLPFSTVVPLK	5	N-Term(iTRAQ4plex); C2(Methylthio); K4(iTRAQ4plex); K17(iTRAQ4plex	1.071	5.56	71	3	779.10
									ATFGCHDGYSLDGPEEIECTK	4	N-Term(iTRAQ4plex); C5(Methylthio); C19(Methylthio); K21(iTRAQ4plex)	0.97	5.14	69	3	884.71
									FICPLTGLWPINTLK	2	N-Term(iTRAQ4plex); C3(Methylthio); K15(iTRAQ4plex)	1.322	3.96	38	2	1025.58
									VSFFCK	3	N-Term(iTRAQ4plex); C5(Methylthio); K6(iTRAQ4plex)	0.8		41		
									KFICPLTGLWPINTLK	1	N-Term(iTRAQ4plex); K1(iTRAQ4plex); C4(Methylthio); K16(iTRAQ4plex)			30		
41281670	CD99L2	CD99 antigen-like protein 2 isoform 2 precursor	17.37	0	2	7		22.8								
									DLEDIVGGGEYKPDK	6	N-Term(iTRAQ4plex); K12(iTRAQ4plex); K15(iTRAQ4plex)		5.04	41	3	689.71
									APANTLGNDFDLADALDDRNDF	1	N-Term(iTRAQ4plex			39		
209571507	PIK3IP1	phosphoinositide-3-kinase-interacting protein 1 isoform 2	17.34	0	2	10		18.5								
									GPWCYVSGEAGVPEK	6	N-Term(iTRAQ4plex); C4(Methylthio); K15(iTRAQ4plex)		5.42	68	2	956.96
									SEAAAVQPVIGISQF	4	N-Term(iTRAQ4plex		4.57	65	2	835.47
5031985	NUTF2	nuclear transport factor 2	17.32	1	2	3	0.649	14.5								
									NINDAWVCTNDMFF	2	N-Term(iTRAQ4plex); C8(Methylthio	0.649	4.57	68	2	944.91
310114265	LOC100506055	PREDICTED: hypothetical protein LOC100506055, partial	17.31	0	1	2		11								
									GPSSEGPEEEDGEGFSFK	2	N-Term(iTRAQ4plex); K18(iTRAQ4plex		3.24	67	2	1086.99
187828910	CD59	CD59 glycoprotein preproprotein	17.19	2	2	18	0.887	14.2								
									FEHCNFNDVTTF	17	N-Term(iTRAQ4plex); C4(Methylthio	0.886	5.41	64	3	558.25
									ENELTYYCCK	1	N-Term(iTRAQ4plex); C8(Methylthio); C9(Methylthio); K10(iTRAQ4plex)	0.939		27		
68533253	CHI3L2	chitinase-3-like protein 2 isoform b	17.11	0	5	11		42.5								
									GNQWVGYDDVK	2	N-Term(iTRAQ4plex); K11(iTRAQ4plex		4.77	63	2	784.90
									QMIDNSYQVEK	4	N-Term(iTRAQ4plex); K11(iTRAQ4plex		4.41	51	2	821.92
									SCNQGPYPLVQAVK	2	N-Term(iTRAQ4plex); C2(Methylthio); K14(iTRAQ4plex)		4.4	66	2	919.49
									LLLTAGVSAGR	2	N-Term(iTRAQ4plex		3.6	55	2	601.37
									GPSSYYNVEYAVGYWIHK	1	N-Term(iTRAQ4plex); K18(iTRAQ4plex			47		
261278346	CGREF1	cell growth regulator with EF hand domain protein 1 isoform a	16.98	4	4	34	0.824	33.4								
									ESLDPVQEPGGQAEADGDVPGPF	4	N-Term(iTRAQ4plex	0.669	6.49	68	3	822.07
									RESLDPVQEPGGQAEADGDVPGPF	7	N-Term(iTRAQ4plex	0.922	6.28	73	3	874.10
									HVEPGEPLAPSPQEPQAVGF	9	N-Term(iTRAQ4plex	0.895	6.17	69	3	747.06
									ELPGETLESK	14	N-Term(iTRAQ4plex); K10(iTRAQ4plex	0.815	4.21	65	2	695.89
54792071	SUMO2	small ubiquitin-related modifier 2 isoform b precursor	16.9	0	1	2		8.1								
									VAGQDGSVVQFK	2	N-Term(iTRAQ4plex); K12(iTRAQ4plex		5.61	70	2	761.93
4507149	SOD1	superoxide dismutase [Cu-Zn]	16.88	3	3	13	0.582	15.9								
									GDGPVQGIINFEQK	10	N-Term(iTRAQ4plex); K14(iTRAQ4plex	0.557	5.99	86	2	895.50
									AVCVLK	1	N-Term(iTRAQ4plex); C3(Methylthio); K6(iTRAQ4plex)	0.694		42		
									VWGSIK	2	N-Term(iTRAQ4plex); K6(iTRAQ4plex	0.622		39		
4504373	HEXB	beta-hexosaminidase subunit beta preproprotein	16.73	8	8	19	0.935	63.1								
									VEPLDFGGTQK	4	N-Term(iTRAQ4plex); K11(iTRAQ4plex	0.837	4.94	77	2	739.91
									GSYSLSHVYTPNDVF	3	N-Term(iTRAQ4plex	1.064	4.81	71	3	613.64
									VLPEFDTPGHTLSWGK	4	N-Term(iTRAQ4plex); K16(iTRAQ4plex	0.915	4.52	57	3	691.37
									WHHEPAEFQAK	3	N-Term(iTRAQ4plex); K11(iTRAQ4plex	0.745	4.14	47	3	556.63
									LAPGTIVEVWK	2	N-Term(iTRAQ4plex); K11(iTRAQ4plex	0.925	4.01	74	2	750.96
									VLDIIATINK	1	N-Term(iTRAQ4plex); K10(iTRAQ4plex	0.853		78		
									GSIVWQEVFDDK	1	N-Term(iTRAQ4plex); K12(iTRAQ4plex	1.118		46		
									GFGTDFK	1	N-Term(iTRAQ4plex); K7(iTRAQ4plex	1.118		38		
4503987	GGH	gamma-glutamyl hydrolase precurso	16.67	5	5	14	0.873	35.9								
									KPIIGILMQK	2	N-Term(iTRAQ4plex); K1(iTRAQ4plex); K10(iTRAQ4plex)	0.862	5.25	47	3	525.02
									NLDGISHAPNAVK	3	N-Term(iTRAQ4plex); K13(iTRAQ4plex	0.742	3.99	46	3	541.98
									FFNVLTTNTDGK	2	N-Term(iTRAQ4plex); K12(iTRAQ4plex	0.884	3.91	68	2	822.95
									IEFISTMEGYK	4	N-Term(iTRAQ4plex); K11(iTRAQ4plex	0.755	3.69	87	2	803.43
									DYEILFK	3	N-Term(iTRAQ4plex); K7(iTRAQ4plex	0.866	3.37	38	2	608.34
68533255	CHI3L2	chitinase-3-like protein 2 isoform a	16.67	0	5	11		43.5								
									GNQWVGYDDVK	2	N-Term(iTRAQ4plex); K11(iTRAQ4plex		4.77	63	2	784.90
									QMIDNSYQVEK	4	N-Term(iTRAQ4plex); K11(iTRAQ4plex		4.41	51	2	821.92
									SCNQGPYPLVQAVK	2	N-Term(iTRAQ4plex); C2(Methylthio); K14(iTRAQ4plex)		4.4	66	2	919.49
									LLLTAGVSAGR	2	N-Term(iTRAQ4plex		3.6	55	2	601.37
									GPSSYYNVEYAVGYWIHK	1	N-Term(iTRAQ4plex); K18(iTRAQ4plex			47		
189181666	HEXA	beta-hexosaminidase subunit alpha preproprotein	16.64	8	8	17	1.035	60.7								
									GSYNPVTHIYTAQDVK	2	N-Term(iTRAQ4plex); K16(iTRAQ4plex	0.976	6.69	73	3	694.37
									LTSDLTFAYER	2	N-Term(iTRAQ4plex	1.019	4.63	54	2	730.38
									ALLSAPWYLNR	2	N-Term(iTRAQ4plex	1.051	3.72	39	2	724.41
									EDIPVNYMK	4	N-Term(iTRAQ4plex); K9(iTRAQ4plex	1.064	3.68	42	2	698.87
									GLETFSQLVWK	2	N-Term(iTRAQ4plex); K11(iTRAQ4plex	0.85	3.37	52	2	798.46
									GYVVWQEVFDNK	2	N-Term(iTRAQ4plex); K12(iTRAQ4plex	1.013	3.27	57	2	886.47
									IQPDTIIQVWR	1	N-Term(iTRAQ4plex	0.901		44		
4757922	CBLN1	cerebellin-1 precursor	16.58	2	3	22	0.951	21.1								
									EAASNGVLIQMEK	4	N-Term(iTRAQ4plex); K13(iTRAQ4plex	0.867	5.16	92	2	839.46
									GIYSFNFHVVK	3	N-Term(iTRAQ4plex); K11(iTRAQ4plex	1.016	4.15	51	2	799.95
									GNLMGGWK	15	N-Term(iTRAQ4plex); K8(iTRAQ4plex		3.64	48	2	575.81
5031863	LGALS3BP	galectin-3-binding protein	16.58	9	9	75	1.056	65.3								

									ASHEEVEGLVEK	26	N-Term(iTRAQ4plex); K12(iTRAQ4plex	1.1	6.55	92	2	807.93
									YSSDYFQAPSDYR	11	N-Term(iTRAQ4plex	1.047	4.85	69	2	871.90
									ELSEALGQIFDSQR	7	N-Term(iTRAQ4plex	0.976	4.74	88	2	868.95
									RIDITLSSVK	5	N-Term(iTRAQ4plex); K10(iTRAQ4plex	1.163	4.58	64	3	473.97
									SDLAVPSELALLK	9	N-Term(iTRAQ4plex); K13(iTRAQ4plex	1.095	4.58	73	2	822.50
									AVDTWSWGER	5	N-Term(iTRAQ4plex	0.97	4.31	41	2	675.83
									IDITLSSVK	10	N-Term(iTRAQ4plex); K9(iTRAQ4plex	0.895	3.79	63	2	632.38
									IYTSPTWSAFVTDSSWSAR	1	N-Term(iTRAQ4plex	1.329		54		
									SLGWLK	1	N-Term(iTRAQ4plex); K6(iTRAQ4plex	1.531		40		
4826669	CDH15	cadherin-15 preproprotein	16.58	10	10	15	1.1	88.9								
									VSVQNEAPLQAAALF	2	N-Term(iTRAQ4plex	1.146	5.36	95	2	855.99
									VHVQDTNEPPVFQENPLF	2	N-Term(iTRAQ4plex	1.205	4.89	43	3	755.06
									VLEGAVPGTYVTF	2	N-Term(iTRAQ4plex	1.069	4.08	51	2	753.42
									AIVLAQDDASQPF	2	N-Term(iTRAQ4plex	0.892	3.51	76	2	764.42
									DLPGSPNWVAR	2	N-Term(iTRAQ4plex	1.125	3.36	65	2	678.36
									FTILEGDPDGQFTIR	1	N-Term(iTRAQ4plex	1.113		57		
									AEATDADDPETDNAALF	1	N-Term(iTRAQ4plex	1.086		46		
									LPYPLVQIK	1	N-Term(iTRAQ4plex); K9(iTRAQ4plex	0.956		42		
88853069	VTN	vitronectin precursor	16.53	6	6	175	0.852	54.3								
									DWHGVPGQVDAAMAGF	13	N-Term(iTRAQ4plex); M13(Oxidation	0.851	7.04	66	3	609.63
									DWHGVPGQVDAAMAGF	49	N-Term(iTRAQ4plex	0.844	5.97	82	3	604.30
									DVWGIEGPIDAAFTR	32	N-Term(iTRAQ4plex	0.826	5.21	92	2	895.97
									GQYCYELDEK	41	N-Term(iTRAQ4plex); C4(Methylthio); K10(iTRAQ4plex)	0.871	4.92	74	2	791.36
									FEDGVLDPDYPR	31	N-Term(iTRAQ4plex	0.877	4.53	59	2	783.88
									RVDTVDPPYPR	6	N-Term(iTRAQ4plex	0.783	3.63	40	3	486.93
									SIAQYWLGCPAPGHL	3	N-Term(iTRAQ4plex); C9(Methylthio	0.852	3.3	65	2	901.95
148664190	CADM1	cell adhesion molecule 1 isoform 1	16.52	0	5	33		48.5								
									SDDSVIQLLNPNR	16	N-Term(iTRAQ4plex		5.54	82	2	807.93
									CEASNIVGK	2	N-Term(iTRAQ4plex); C1(Methylthio); K9(iTRAQ4plex)		4.25	64	2	627.83
									NLMIDIQK	11	N-Term(iTRAQ4plex); K8(iTRAQ4plex		4.14	59	2	631.88
									NLMIDIQK	2	N-Term(iTRAQ4plex); M3(Oxidation); K8(iTRAQ4plex)		3.39	44	2	639.86
									EDDGVPVICQVEHPAVTGNLQTQI	1	N-Term(iTRAQ4plex); C9(Methylthio			34		
									SEVEEWSDMYTVTSQLMLK	1	N-Term(iTRAQ4plex); K19(iTRAQ4plex			33		
87196339	COL6A1	collagen alpha-1(VI) chain precursor	16.44	13	13	81	0.993	108.5								
									LSIIATDHTYR	11	N-Term(iTRAQ4plex	1.036	6.07	82	3	478.60
									TAEYDVAYGESHLFF	14	N-Term(iTRAQ4plex	0.986	5.83	71	3	634.64
									GLEQLLVGGSHLK	13	N-Term(iTRAQ4plex); K13(iTRAQ4plex	0.976	5.82	81	3	547.00
									YLIVVTDGHPLEGYK	4	N-Term(iTRAQ4plex); K15(iTRAQ4plex	1.068	5.29	67	3	664.71
									VFSVAITPDHLEPF	12	N-Term(iTRAQ4plex	1.074	5.22	65	3	575.65
									GTYTDCAIK	6	N-Term(iTRAQ4plex); C6(Methylthio); K9(iTRAQ4plex)	0.934	4.73	49	2	653.31
									LKPYGALVDK	4	N-Term(iTRAQ4plex); K2(iTRAQ4plex); K10(iTRAQ4plex)	1	4.11	41	3	512.66
									ENYAELLEDAFLK	2	N-Term(iTRAQ4plex); K13(iTRAQ4plex	1.123	3.86	59	2	921.99
									EPCGGLEDAVNEAK	3	N-Term(iTRAQ4plex); C3(Methylthio); K14(iTRAQ4plex)	0.779	3.77	63	2	883.42
									VAVVQYSGTGQQRPEF	2	N-Term(iTRAQ4plex	1.057	3.6	49	3	640.35
									IALVITDGR	7	N-Term(iTRAQ4plex	0.91	3.37	58	2	551.34
									GDEGEAGDPGDDNNDIAPF	2	N-Term(iTRAQ4plex	0.818		66		
4826898	PFN1	profilin-1	16.43	2	2	6	0.982	15								
									STGGAPTFNVTVTI	2	N-Term(iTRAQ4plex); K14(iTRAQ4plex	0.898	6.29	100	2	834.47
									CYEMASHLR	4	N-Term(iTRAQ4plex); C1(Methylthio	0.991	3.69	44	3	433.86
214010183	APLP2	amyloid-like protein 2 isoform 3	16.4	0	11	33		79.2								
									SQVMTHLHVIEEF	5	N-Term(iTRAQ4plex		6.14	86	4	431.48
									QTLIQHFQAMVK	4	N-Term(iTRAQ4plex); K12(iTRAQ4plex		6.02	54	2	866.49
									KEWEEAELQAK	2	N-Term(iTRAQ4plex); K1(iTRAQ4plex); K11(iTRAQ4plex)		5.79	51	3	598.33
									LNMHVNIQTGK	4	N-Term(iTRAQ4plex); K11(iTRAQ4plex		5.31	67	3	514.96
									EWEEAELQAK	2	N-Term(iTRAQ4plex); K10(iTRAQ4plex		5.14	55	2	760.90
									VEAMLNDR	2	N-Term(iTRAQ4plex		3.42	43	2	546.28
									EMIFNAER	2	N-Term(iTRAQ4plex		3.38	43	2	577.29
									VSIDNWCR	4	N-Term(iTRAQ4plex); C7(Methylthio		3.26	41	2	591.78
									ADMDQFTASISETPVDVF	1	N-Term(iTRAQ4plex			59		
									MALENYLAALQSDPPRPHF	2	N-Term(iTRAQ4plex			52		
									FVTPFK	5	N-Term(iTRAQ4plex); K6(iTRAQ4plex			43		
4504489	HRG	histidine-rich glycoprotein precursor	16.38	7	7	27	0.821	59.5								
									YKEENDDFASFR	4	N-Term(iTRAQ4plex); K2(iTRAQ4plex	0.822	4.96	50	3	603.63
									GGEGTGYFVDFSVR	2	N-Term(iTRAQ4plex	0.759	4.94	72	2	817.90
									ALDLINK	12	N-Term(iTRAQ4plex); K7(iTRAQ4plex	0.858	3.36	42	2	537.85
									DSPVLIDFFEDTER	6	N-Term(iTRAQ4plex	0.744	3.27	74	2	913.95
									KYWNDCEPPDSR	1	N-Term(iTRAQ4plex); K1(iTRAQ4plex); C6(Methylthio)	1.288		44		
									ADLFYDVEALDLESPK	1	N-Term(iTRAQ4plex); K16(iTRAQ4plex	0.536		36		
4557892	LCAT	phosphatidylcholine-sterol acyltransferase precursor	16.36	6	6	22	1.003	49.5								
									TYSVEYLDSSK	2	N-Term(iTRAQ4plex); K11(iTRAQ4plex	1.116	5.37	82	2	790.41
									SSGLVSNAPGVQIF	5	N-Term(iTRAQ4plex	0.932	5.35	82	2	764.93
									LEPGQQEEYYR	7	N-Term(iTRAQ4plex	1.03	5.01	58	2	778.38
									STELCGLWQGR	4	N-Term(iTRAQ4plex); C5(Methylthio	1.001	4.66	60	2	720.35
									ITTTSPWMFPSR	3	N-Term(iTRAQ4plex	1.055	4.39	68	2	784.41
									LDKPDVVNWMCYR	1	N-Term(iTRAQ4plex); K3(iTRAQ4plex); C11(Methylthio)	0.892		36		
295849266	DBI	acyl-CoA-binding protein isoform 4	16.22	0	2	8		16.5								
									TKPSDEEMLFIYGHYK	2	N-Term(iTRAQ4plex); K2(iTRAQ4plex); K16(iTRAQ4plex)		6.37	55	3	797.42
									WDAWNELK	6	N-Term(iTRAQ4plex); K8(iTRAQ4plex		3.93	46	2	675.36
237858675	NFASC	neurofascin isoform 1 precursor	16.21	1	18	60	1.003	137.5								
									VIAINEVGSSHPSLPSEF	4	N-Term(iTRAQ4plex		7.15	74	3	679.37
									EDDSLTIFGVAER	4	N-Term(iTRAQ4plex		5.45	81	2	798.41
									SGGRPEEYEGEYQCFAR	2	N-Term(iTRAQ4plex); C14(Methylthio		5.29	35	3	723.32
									KEDDSLTIFGVAER	2	N-Term(iTRAQ4plex); K1(iTRAQ4plex		5.08	56	3	623.34
									YVVGQTPVYVPYEIF	4	N-Term(iTRAQ4plex		4.91	65	2	964.03
									AAPYWLDEPK	9	N-Term(iTRAQ4plex); K10(iTRAQ4plex		4.68	76	2	739.40
									LDCPFFGSPIPTLR	2	N-Term(iTRAQ4plex); C3(Methylthio		4.57	53	2	876.95
									GTTVQLECR	2	N-Term(iTRAQ4plex); C8(Methylthio		4.52	46	2	598.80

									DDEPLYIGNR	2	N-Term(iTRAQ4plex		4.14	34	2	668.34
									EVAGDTIIFR	2	N-Term(iTRAQ4plex		4.13	55	2	632.86
									DLELTDLAER	5	N-Term(iTRAQ4plex		3.98	47	2	659.85
									NLILAPGEDGR	4	N-Term(iTRAQ4plex		3.79	50	2	649.86
									GPEPESVIGYSGEDLPSAPR	2	N-Term(iTRAQ4plex	1.003	3.62	51	2	1101.05
									GNPAPSFHWTR	2	N-Term(iTRAQ4plex		3.37	38	2	707.36
									SGTLVIDFR	2	N-Term(iTRAQ4plex		3.22	41	2	576.33
									LTVSWLK	8	N-Term(iTRAQ4plex); K7(iTRAQ4plex			53		
									FENFNK	3	N-Term(iTRAQ4plex); K6(iTRAQ4plex			37		
7706123	NRN1	neuritin precursor	16.2	2	2	7	0.795	15.3								
									LGDSMANYPQGLDDK	5	N-Term(iTRAQ4plex); K15(iTRAQ4plex	0.726	5.33	77	2	956.47
									GFSDCLLK	2	N-Term(iTRAQ4plex); C5(Methylthio); K8(iTRAQ4plex)	0.982	3.93	47	2	608.82
148225659	ENDOD1	endonuclease domain-containing 1 protein precursor	16.2	6	6	29	0.989	55								
									FFYAGTPPAGLAADSHVK	2	N-Term(iTRAQ4plex); K18(iTRAQ4plex	0.838	8.09	89	3	713.05
									QALNTDYLDSDYQR	8	N-Term(iTRAQ4plex	1.04	5.71	81	2	923.44
									ILEVVNQIQDEER	14	N-Term(iTRAQ4plex	0.994	5.54	86	2	864.97
									LVGEEEAGFGECDK	3	N-Term(iTRAQ4plex); C12(Methylthio); K14(iTRAQ4plex)	1.04	4.81	104	2	908.92
									DSDIIEDVMVK	1	N-Term(iTRAQ4plex); K11(iTRAQ4plex	0.75		70		
									WYVNLHSLMDR	1	N-Term(iTRAQ4plex	1.077		42		
4557323	APOC3	apolipoprotein C-III precursor	16.16	1	1	2	0.996	10.8								
									DALSSVQESQVAQQAR	2	N-Term(iTRAQ4plex	0.996	4.42	109	2	930.99
13775198	SH3BGRL3	SH3 domain-binding glutamic acid-rich-like protein 3	16.13	1	1	2	0.849	10.4								
									IQYQLVDISQDNALR	2	N-Term(iTRAQ4plex	0.849	4.13	83	2	960.52
58218968	CALM3	calmodulin	16.11	0	2	4		16.8								
									VFDKDGNGYISAAELR	2	N-Term(iTRAQ4plex); K4(iTRAQ4plex		5.84	70	3	681.70
									EAFSLFDK	2	N-Term(iTRAQ4plex); K8(iTRAQ4plex		4.19	47	2	622.84
4826655	CALB1	calbindin	16.09	4	4	9	0.982	30								
									YDTDHSGFIETEELK	2	N-Term(iTRAQ4plex); K15(iTRAQ4plex	0.951	4.99	61	3	691.34
									LAEYTDLMLK	2	N-Term(iTRAQ4plex); K10(iTRAQ4plex	1.234	4.54	62	2	742.92
									AGLELSPEMK	4	N-Term(iTRAQ4plex); K10(iTRAQ4plex	0.967	4.47	60	2	681.88
									SCEEFMK	1	N-Term(iTRAQ4plex); C2(Methylthio); K7(iTRAQ4plex)	0.982		35		
166064050	FOLR2	folate receptor beta precursor	16.08	3	4	5	0.929	29.3								
									GWDWTSGVNK	2	N-Term(iTRAQ4plex); K10(iTRAQ4plex	0.929	4.58	53	2	719.37
									TDLLNVCMDAK	1	N-Term(iTRAQ4plex); C7(Methylthio); K11(iTRAQ4plex)	1.122		57		
									LYNFNWDHCGK	1	N-Term(iTRAQ4plex); C9(Methylthio); K11(iTRAQ4plex)			35		
118442839	CFHR1	complement factor H-related protein 1 precursor	16.06	1	5	32	1.658	37.6								
									LQNNENNISCVER	4	N-Term(iTRAQ4plex); C10(Methylthio		5.89	84	2	861.90
									INHGILYDEEK	5	N-Term(iTRAQ4plex); K11(iTRAQ4plex		5.16	76	3	540.30
									EIMENYNIALR	16	N-Term(iTRAQ4plex		5.1	60	2	755.40
									EIMENYNIALR	4	N-Term(iTRAQ4plex); M3(Oxidation		4.22	62	2	763.40
									LEYPTCAK	2	N-Term(iTRAQ4plex); C6(Methylthio); K8(iTRAQ4plex)			50		
									TGESAEFVCK	1	N-Term(iTRAQ4plex); C9(Methylthio); K10(iTRAQ4plex)	1.658		48		
5031695	CFHR2	complement factor H-related protein 2 precursor	15.93	2	4	12	0.89	30.6								
									LQNNENNISCVER	4	N-Term(iTRAQ4plex); C10(Methylthio		5.89	84	2	861.90
									INHGILYDEEK	5	N-Term(iTRAQ4plex); K11(iTRAQ4plex		5.16	76	3	540.30
									LVYPSCEEK	2	N-Term(iTRAQ4plex); C6(Methylthio); K9(iTRAQ4plex)	0.886	3.46	50	2	701.35
									TGDIVEFVCK	1	N-Term(iTRAQ4plex); C9(Methylthio); K10(iTRAQ4plex)	0.894		57		
166235903	C1QC	complement C1q subcomponent subunit C precursor	15.92	3	3	6	0.706	25.8								
									TNQVNSGGVLLR	2	N-Term(iTRAQ4plex	0.704	3.91	37	2	701.40
									FQSVFTVTR	3	N-Term(iTRAQ4plex	0.73	3.89	44	2	614.84
									FNAVLTNPQGDYDTSTGK	1	N-Term(iTRAQ4plex); K18(iTRAQ4plex	0.592		45		
4502163	APOD	apolipoprotein D precursor	15.87	3	3	13	0.677	21.3								
									NILTSNNIDVK	4	N-Term(iTRAQ4plex); K11(iTRAQ4plex	0.78	5.25	83	2	759.94
									NPNLPPETVDSLK	8	N-Term(iTRAQ4plex); K13(iTRAQ4plex	0.65	3.2	61	2	856.48
									WYEIEK	1	N-Term(iTRAQ4plex); K6(iTRAQ4plex	0.73		36		
226437599	VSTM2B	V-set and transmembrane domain-containing protein 2B precursor	15.79	3	3	18	0.953	30.3								
									FAPPNMQAAEAVSHIQSSGPR	3	N-Term(iTRAQ4plex	1.417	5.08	81	3	780.39
									ELLHELALSVPGAR	12	N-Term(iTRAQ4plex	0.953	4.49	97	2	824.98
									LQDEGVYECR	3	N-Term(iTRAQ4plex); C9(Methylthio	0.905	3.82	43	2	701.32
4502133	APCS	serum amyloid P-component precursor	15.7	3	3	13	0.86	25.4								
									IVLGQEQDSYGGK	6	N-Term(iTRAQ4plex); K13(iTRAQ4plex	0.86	5.23	99	2	841.45
									VGEYSLYIGR	4	N-Term(iTRAQ4plex	0.747	4.66	60	2	650.85
									AYSLFSYNTQGR	3	N-Term(iTRAQ4plex	0.873	3.74	64	2	775.89
157427673	NRXN3	neurexin-3-beta isoform 3 precursor	15.69	1	5	18	1.06	50.7								
									VLNMAAENNPNIK	8	N-Term(iTRAQ4plex); K13(iTRAQ4plex		5.72	100	2	858.47
									IDSAPGLGDFLQLHIEQGK	4	N-Term(iTRAQ4plex); K19(iTRAQ4plex		5.37	85	3	776.10
									YNRPVEEWLQEK	2	N-Term(iTRAQ4plex); K12(iTRAQ4plex	1.06	5.05	49	2	940.00
									SGGLILYTWPANDRPSTR	3	N-Term(iTRAQ4plex		4.75	36	3	716.72
									LAVGFSTTVK	1	N-Term(iTRAQ4plex); K10(iTRAQ4plex			39		
62526026	CUTA	protein CutA isoform 3	15.64	0	3	15		19.1								
									IEEDSEVLMMIK	2	N-Term(iTRAQ4plex); K12(iTRAQ4plex		5.39	37	2	862.96
									TQSSLVPALTDFVR	8	N-Term(iTRAQ4plex		5.17	88	2	839.47
									GKIEEDSEVLMMIK	5	N-Term(iTRAQ4plex); K2(iTRAQ4plex); K14(iTRAQ4plex)		3.95	74	2	1027.56
4557581	FABP5	fatty acid-binding protein, epidermal	15.56	1	1	2	0.913	15.2								
									TQTVCNFTDGALVQHQEWDGK	2	N-Term(iTRAQ4plex); C5(Methylthio); K21(iTRAQ4plex)	0.913	3.94	90	3	904.44
194097382	ART3	ecto-ADP-ribosyltransferase 3 isoform c	15.53	0	5	11		41.5								
									ASHQQLDTVWENAK	2	N-Term(iTRAQ4plex); K14(iTRAQ4plex		6.59	67	3	639.00
									TSQGTSFTFGGLNQAR	4	N-Term(iTRAQ4plex		5.56	114	2	908.45
									EDYIYGFQFK	2	N-Term(iTRAQ4plex); K10(iTRAQ4plex		4.54	63	2	799.41
									TQIFLPMNFK	1	N-Term(iTRAQ4plex); K10(iTRAQ4plex			57		
									YVPQLLK	2	N-Term(iTRAQ4plex); K7(iTRAQ4plex			50		

164419739	GRIA4	glutamate receptor 4 isoform 3 precursor	15.47	0	6	17		49.1								
									IQGLTGNVQFDHYGF	6	N-Term(iTRAQ4plex		5.68	97	3	616.99
									LQNILEQIVSVGK	3	N-Term(iTRAQ4plex); K13(iTRAQ4plex		4.73	71	2	865.03
									GVFAIFGLYDK	4	N-Term(iTRAQ4plex); K11(iTRAQ4plex		3.2	74	2	759.44
									GYSILQAIMEK	2	N-Term(iTRAQ4plex); K11(iTRAQ4plex			50		
									QLLEELDR	1	N-Term(iTRAQ4plex			41		
									FVIDCEIER	1	N-Term(iTRAQ4plex); C5(Methylthio			41		
4507509	TIMP1	metalloproteinase inhibitor 1 precursor	15.46	3	3	29	0.957	23.2								
									GFQALGDAADIR	11	N-Term(iTRAQ4plex	1.02	5.11	61	2	689.37
									SEEFLIAGK	16	N-Term(iTRAQ4plex); K9(iTRAQ4plex	0.936	4.44	62	2	641.37
									EPGLCTWQSLR	2	N-Term(iTRAQ4plex); C5(Methylthio	0.99	3.82	43	2	740.36
11321593	IGFBP6	insulin-like growth factor-binding protein 6 precursor	15.42	4	4	31	0.905	25.3								
									HLDSVLQQLQTEVYR	13	N-Term(iTRAQ4plex	1.353	5.13	108	3	658.36
									RGPCWCVDR	8	N-Term(iTRAQ4plex); C4(Methylthio); C6(Methylthio)	0.791	4	76	3	443.19
									GAQTLYVPNCDHR	3	N-Term(iTRAQ4plex); C10(Methylthio	1.093	3.9	43	3	555.26
									GPCWCVDR	7	N-Term(iTRAQ4plex); C3(Methylthio); C5(Methylthio)	0.898		37		
38016947	C5	complement C5 preproprotein	15.39	25	25	74	0.768	188.2								
									ITHYNYLILSK	4	N-Term(iTRAQ4plex); K11(iTRAQ4plex	0.878	5.77	89	2	826.99
									GGSASTWLTAFALR	2	N-Term(iTRAQ4plex	0.634	5.7	77	2	791.43
									DINYVNPVIK	8	N-Term(iTRAQ4plex); K10(iTRAQ4plex	0.686	5.04	65	2	731.93
									ALLVGEHLNIIVTPK	4	N-Term(iTRAQ4plex); K15(iTRAQ4plex	0.871	4.83	72	3	635.73
									IDTQDIEASHYR	8	N-Term(iTRAQ4plex	0.838	4.82	72	2	796.40
									FQNSAILTIQPK	2	N-Term(iTRAQ4plex); K12(iTRAQ4plex	0.786	4.58	87	2	824.49
									LSMDIDVSYK	4	N-Term(iTRAQ4plex); K10(iTRAQ4plex	0.651	4.53	72	2	729.89
									NADYSYSVWK	4	N-Term(iTRAQ4plex); K10(iTRAQ4plex	0.772	4.46	87	2	760.89
									EGMLSIMSYR	2	N-Term(iTRAQ4plex	0.646	4.33	63	2	665.84
									TLLPVSKPEIR	3	N-Term(iTRAQ4plex); K7(iTRAQ4plex	0.784	3.93	42	2	770.99
									ATLLDIYK	8	N-Term(iTRAQ4plex); K8(iTRAQ4plex	0.751	3.79	52	2	612.88
									TDAPDLPEENQAR	4	N-Term(iTRAQ4plex	0.745	3.56	78	2	800.39
									ELSYYSLEDLNNK	2	N-Term(iTRAQ4plex); K13(iTRAQ4plex	0.615	3.56	66	2	938.49
									KVTCTNAELVK	2	N-Term(iTRAQ4plex); K1(iTRAQ4plex); C4(Methylthio); K11(iTRAQ4plex)	0.774	3.55	48	3	561.99
									VFQFLEK	2	N-Term(iTRAQ4plex); K7(iTRAQ4plex	0.833	3.38	41	2	599.86
									VTCTNAELVK	2	N-Term(iTRAQ4plex); C3(Methylthio); K10(iTRAQ4plex)	0.671	3.31	42	2	706.39
									IDTALIK	2	N-Term(iTRAQ4plex); K7(iTRAQ4plex	0.585	3.27	34	2	531.35
									TSGMQFCVK	2	N-Term(iTRAQ4plex); C7(Methylthio); K9(iTRAQ4plex)	0.713	3.14	51	2	667.83
									YVLSPYK	1	N-Term(iTRAQ4plex); K7(iTRAQ4plex	0.59		49		
									NFEITIK	1	N-Term(iTRAQ4plex); K7(iTRAQ4plex	0.788		49		
									IPLDLVPK	3	N-Term(iTRAQ4plex); K8(iTRAQ4plex	0.768		41		
									SYFPESWLWEVHLVPR	1	N-Term(iTRAQ4plex	0.481		41		
									QYLIMGK	1	N-Term(iTRAQ4plex); K7(iTRAQ4plex	0.769		36		
									LNLVATPLFLKPGIPYPIK	1	N-Term(iTRAQ4plex); K11(iTRAQ4plex); K19(iTRAQ4plex)	0.767		27		
5453876	PENK	proenkephalin-A	15.36	3	3	27	0.95	30.8								
									DAEEDDSLANSSDLLK	21	N-Term(iTRAQ4plex); K16(iTRAQ4plex	0.889	6.93	120	2	1005.49
									FAEALPSDEEGESYSK	5	N-Term(iTRAQ4plex); K16(iTRAQ4plex	1.238	4.2	96	2	1023.99
									ELLETGDNR	1	N-Term(iTRAQ4plex	1.085		40		
122937309	LRRC4B	leucine-rich repeat-containing protein 4B precursor	15.29	8	11	33	0.948	76.4								
									NNPIESIPSYAFNR	6	N-Term(iTRAQ4plex		4.89	57	2	883.45
									DLAEVPASIPVNTR	6	N-Term(iTRAQ4plex	0.948	4.46	65	2	813.45
									LEELELSGNR	4	N-Term(iTRAQ4plex	0.91	4.34	43	2	652.35
									RLDLGELK	4	N-Term(iTRAQ4plex); K8(iTRAQ4plex		4.16	47	3	411.26
									RDLAEVPASIPVNTR	2	N-Term(iTRAQ4plex	0.921	3.97	38	2	891.51
									LTTVPTQAFEYLSK	2	N-Term(iTRAQ4plex); K14(iTRAQ4plex	1.007	3.97	50	2	943.53
									LDLIRPGSFQGLTSLR	2	N-Term(iTRAQ4plex	0.992	3.44	35	3	639.71
									LWLMHAQVATIER	3	N-Term(iTRAQ4plex	0.937	3.37	66	3	571.32
									LDLGELK	1	N-Term(iTRAQ4plex); K7(iTRAQ4plex			43		
									HLEILQLSK	2	N-Term(iTRAQ4plex); K9(iTRAQ4plex	0.949		36		
4501889	ACTG2	actin, gamma-enteric smooth muscle isoform 1 precursor	15.16	0	5	17		41.8								
									EITALAPSTMK	3	N-Term(iTRAQ4plex); K11(iTRAQ4plex		4.19	66	2	725.42
									DLTDYLMK	5	N-Term(iTRAQ4plex); K8(iTRAQ4plex		3.98	48	2	643.85
									YPIEHGIITNWDDMEK	4	N-Term(iTRAQ4plex); K16(iTRAQ4plex		3.37	54	3	750.38
									SYELPDGQVITIGNER	3	N-Term(iTRAQ4plex		3.27	49	2	968.00
									EITALAPSTMK	1	N-Term(iTRAQ4plex); M10(Oxidation); K11(iTRAQ4plex)			43		
									GILTLK	1	N-Term(iTRAQ4plex); K6(iTRAQ4plex			31		
213688375	ACTA2	actin, aortic smooth muscle	15.12	0	5	17		42								
									EITALAPSTMK	3	N-Term(iTRAQ4plex); K11(iTRAQ4plex		4.19	66	2	725.42
									DLTDYLMK	5	N-Term(iTRAQ4plex); K8(iTRAQ4plex		3.98	48	2	643.85
									YPIEHGIITNWDDMEK	4	N-Term(iTRAQ4plex); K16(iTRAQ4plex		3.37	54	3	750.38
									SYELPDGQVITIGNER	3	N-Term(iTRAQ4plex		3.27	49	2	968.00
									EITALAPSTMK	1	N-Term(iTRAQ4plex); M10(Oxidation); K11(iTRAQ4plex)			43		
									GILTLK	1	N-Term(iTRAQ4plex); K6(iTRAQ4plex			31		
312283703	MDH1	malate dehydrogenase, cytoplasmic isoform 3	15.1	0	3	6		27								
									VIVVGNPANTNCLTASK	2	N-Term(iTRAQ4plex); C12(Methylthio); K17(iTRAQ4plex)		5.01	68	2	1018.05
									FVEGLPINDFSR	3	N-Term(iTRAQ4plex		4.07	68	2	769.41
									ENFSCLTR	1	N-Term(iTRAQ4plex); C5(Methylthio			31		
21361167	ART3	ecto-ADP-ribosyltransferase 3 isoform b	15.08	0	5	11		42.7								
									ASHQQLDTVWENAK	2	N-Term(iTRAQ4plex); K14(iTRAQ4plex		6.59	67	3	639.00
									TSQGTSFTFGGLNQAR	4	N-Term(iTRAQ4plex		5.56	114	2	908.45
									EDYIYGFQFK	2	N-Term(iTRAQ4plex); K10(iTRAQ4plex		4.54	63	2	799.41
									TQIFLPMNFK	1	N-Term(iTRAQ4plex); K10(iTRAQ4plex			57		
									YVPQLLK	2	N-Term(iTRAQ4plex); K7(iTRAQ4plex			50		
87298828	C1QB	complement C1q subcomponent subunit B precursor	15.02	3	3	17	0.77	26.7								
									FDHVITNMNNNYEPR	3	N-Term(iTRAQ4plex); M8(Oxidation	0.95	6.28	75	3	675.32
									LEQGENVFLQATDK	2	N-Term(iTRAQ4plex); K14(iTRAQ4plex	0.77	5.84	72	2	940.51
									FDHVITNMNNNYEPR	8	N-Term(iTRAQ4plex	0.761	5.51	71	3	669.99
									GNLCVNLMR	4	N-Term(iTRAQ4plex); C4(Methylthio	0.661	4.02	56	2	605.31

5803011	ENO2	gamma-enolase	14.98	5	5	10	0.822	47.2								
									LGAEVYHTLK	2	N-Term(iTRAQ4plex); K10(iTRAQ4plex	0.879	5.14	58	2	709.92
									LAQENGWGVMVSHR	2	N-Term(iTRAQ4plex	0.593	4.81	36	3	576.63
									IEEELGDEAR	2	N-Term(iTRAQ4plex	0.822	3.64	52	2	652.82
									VNQIGSVTEAIQACK	2	N-Term(iTRAQ4plex); C14(Methylthio); K15(iTRAQ4plex)	0.857	3.25	75	2	948.01
									YITGDQLGALYQDFVR	2	N-Term(iTRAQ4plex	0.784	3.18	63	2	1002.03
5174539	MDH1	malate dehydrogenase, cytoplasmic isoform 2	14.97	0	4	7		36.4								
									VIVVGNPANTNCLTASI	2	N-Term(iTRAQ4plex); C12(Methylthio); K17(iTRAQ4plex)		5.01	68	2	1018.05
									FVEGLPINDFSR	3	N-Term(iTRAQ4plex		4.07	68	2	769.41
									ENFSCLTR	1	N-Term(iTRAQ4plex); C5(Methylthio			31		
5803225	YWHAE	14-3-3 protein epsilon	14.9	3	4	6	0.733	29.2								
									DSTLIMQLLR	2	N-Term(iTRAQ4plex		4.44	67	2	667.39
									YDEMVESMK	2	N-Term(iTRAQ4plex); K9(iTRAQ4plex	0.733	3.96	54	2	710.34
									EALQDVEDENQ	1	N-Term(iTRAQ4plex	0.631		50		
									IISSIEQK	1	N-Term(iTRAQ4plex); K8(iTRAQ4plex	0.756		44		
42560233	PVRL1	poliovirus receptor-related protein 1 isoform 3 precursor	14.77	0	4	13		39.1								
									VEFLRPSFTDGTIR	5	N-Term(iTRAQ4plex		4.04	43	3	594.66
									LKGEAEYQEIR	4	N-Term(iTRAQ4plex); K2(iTRAQ4plex		3.78	57	2	812.45
									QNVAIYNPSMGVSVLAPYF	2	N-Term(iTRAQ4plex		3.23	40	2	1112.09
									ITQVTWQK	2	N-Term(iTRAQ4plex); K8(iTRAQ4plex			56		
194097380	ART3	ecto-ADP-ribosyltransferase 3 isoform a	14.65	0	5	11		43.9								
									ASHQQLDTVWENAK	2	N-Term(iTRAQ4plex); K14(iTRAQ4plex		6.59	67	3	639.00
									TSQGTSFTFGGLNQAR	4	N-Term(iTRAQ4plex		5.56	114	2	908.45
									EDYIYGFQFK	2	N-Term(iTRAQ4plex); K10(iTRAQ4plex		4.54	63	2	799.41
									TQIFLPMNFK	1	N-Term(iTRAQ4plex); K10(iTRAQ4plex			57		
									YVPQLLK	2	N-Term(iTRAQ4plex); K7(iTRAQ4plex			50		
192447438	PROS1	vitamin K-dependent protein S preproprotein	14.64	9	9	35	1.061	75.1								
									IETISHEDLQR	15	N-Term(iTRAQ4plex	1.075	5.34	70	3	495.60
									IQALSLCSDQQSHLEFR	2	N-Term(iTRAQ4plex); C7(Methylthio	0.984	4.67	89	3	722.36
									NNLELSTPLK	6	N-Term(iTRAQ4plex); K10(iTRAQ4plex	1.098	4.6	57	2	708.92
									SFQTGLFTAAR	4	N-Term(iTRAQ4plex	1.016	4.52	65	2	671.87
									FSAEFDFR	3	N-Term(iTRAQ4plex	1.052	3.5	38	2	581.79
									SQDILLSVENTVIYR	2	N-Term(iTRAQ4plex	1.033	3.34	51	2	947.53
									IEVQLK	1	N-Term(iTRAQ4plex); K6(iTRAQ4plex	1.16		37		
									VYFAGFPR	1	N-Term(iTRAQ4plex	1.14		35		
51477716	MAN2A2	alpha-mannosidase 2x	14.43	12	12	19	0.815	130.5								
									VIDSGTSDFALSNR	3	N-Term(iTRAQ4plex	0.826	5.1	99	2	813.42
									GAEVLYSLAAAHAR	2	N-Term(iTRAQ4plex	0.844	4.21	61	3	524.96
									EAVVVDYGVR	2	N-Term(iTRAQ4plex	0.782	3.6	55	2	625.85
									TLQAEEDTLPSAETALILHR	2	N-Term(iTRAQ4plex	0.54	3.56	81	3	784.76
									FSMVSLLVNSPR	2	N-Term(iTRAQ4plex	0.815	3.41	67	2	747.42
									FVVLFNPLEQER	2	N-Term(iTRAQ4plex	0.877	3.2	43	2	817.96
									ETYHFDPEAPFLQVDDTR	1	N-Term(iTRAQ4plex	0.641		61		
									LTLHTAQALGVSSLK	1	N-Term(iTRAQ4plex); K15(iTRAQ4plex	0.937		54		
									YDKPQEWDAQFFNYQR	1	N-Term(iTRAQ4plex); K3(iTRAQ4plex	0.778		48		
									YPLSDFTLLTEAR	1	N-Term(iTRAQ4plex	0.857		47		
									SNVLLVPLGDDFR	1	N-Term(iTRAQ4plex	0.739		37		
4505529	ORM2	alpha-1-acid glycoprotein 2 precursor	14.43	2	3	4	0.541	23.6								
									TLMFGSYLDDEK	1	N-Term(iTRAQ4plex); K12(iTRAQ4plex	0.518		42		
4758426	GDA	guanine deaminase	14.32	6	6	16	0.537	51								
									NIEEVYVGGK	4	N-Term(iTRAQ4plex); K10(iTRAQ4plex	0.615	4.54	73	2	698.39
									EFDAILINPK	2	N-Term(iTRAQ4plex); K10(iTRAQ4plex	0.647	4.22	41	2	724.43
									FQNIDFAEEVYTR	2	N-Term(iTRAQ4plex	0.466	3.74	55	2	888.44
									AVMVSNILLINK	2	N-Term(iTRAQ4plex); K12(iTRAQ4plex	0.616	3.59	90	2	802.00
									FVSEMLQK	2	N-Term(iTRAQ4plex); K8(iTRAQ4plex	0.499	3.46	49	2	635.36
									IVFLEEASQQEK	4	N-Term(iTRAQ4plex); K12(iTRAQ4plex	0.49	3.41	93	2	854.98
21070980	PAM	peptidyl-glycine alpha-amidating monooxygenase isoform c preproprotein	14.32	0	11	49		96.2								
									DGNYWVTDVALHQVFK	7	N-Term(iTRAQ4plex); K16(iTRAQ4plex		6.34	71	3	727.39
									IPVDEEAFVIDFKPR	7	N-Term(iTRAQ4plex); K13(iTRAQ4plex		6.2	55	3	688.39
									NLFYLPHGLSIDK	4	N-Term(iTRAQ4plex); K13(iTRAQ4plex		5.72	67	3	602.35
									DKIPLLQQPK	2	N-Term(iTRAQ4plex); K2(iTRAQ4plex); K10(iTRAQ4plex)		5.69	30	3	538.01
									GDHVWDGNSFDSK	9	N-Term(iTRAQ4plex); K13(iTRAQ4plex		5.57	75	3	584.62
									DCSGVSLHLTR	3	N-Term(iTRAQ4plex); C2(Methylthio		4.61	51	2	689.34
									NYPMHVFAYR	5	N-Term(iTRAQ4plex		3.72	46	3	481.24
									EGPVLILGR	4	N-Term(iTRAQ4plex		3.71	47	2	549.35
									AGIEVQEIK	2	N-Term(iTRAQ4plex); K9(iTRAQ4plex		3.6	63	2	637.87
									IVQFSPSGK	3	N-Term(iTRAQ4plex); K9(iTRAQ4plex		3.52	48	2	625.87
									ANILYAWAR	3	N-Term(iTRAQ4plex		3.23	56	2	611.35
4504351	HBD	hemoglobin subunit delta	14.29	0	2	11		16								
									VVAGVANALAHK	6	N-Term(iTRAQ4plex); K12(iTRAQ4plex		5.99	78	3	479.97
									LHVDPENFR	5	N-Term(iTRAQ4plex		3.47	38	3	424.23
30425563	RTN4RL2	reticulon-4 receptor-like 2 precursor	14.29	5	5	15	0.753	46.1								
									VSSSDVTCATPPER	6	N-Term(iTRAQ4plex); C8(Methylthio	0.744	5.85	73	2	819.89
									SLEPDTFQGLER	4	N-Term(iTRAQ4plex	0.852	4.15	55	2	768.40
									LLTEHVFR	3	N-Term(iTRAQ4plex	0.638	3.27	42	2	579.84
13249346	NPPC	C-type natriuretic peptide precursor	14.29	1	1	4	1.376	13.2								
									TPPAEELAEPQAAGGGQK	4	N-Term(iTRAQ4plex); K18(iTRAQ4plex	1.376	5.33	105	2	1020.03
55770842	CRP	C-reactive protein precursor	14.29	3	3	7	0.291	25								
									GYSIFSYATK	3	N-Term(iTRAQ4plex); K10(iTRAQ4plex	0.439	5.02	50	2	712.89
									ESDTSYVSLK	2	N-Term(iTRAQ4plex); K10(iTRAQ4plex	0.257	4.64	64	2	708.88
									RQDNEILIFWSK	2	N-Term(iTRAQ4plex); K12(iTRAQ4plex	0.261		50		
312283701	MDH1	malate dehydrogenase, cytoplasmic isoform 1	14.2	0	4	7		38.6								
									VIVVGNPANTNCLTASI	2	N-Term(iTRAQ4plex); C12(Methylthio); K17(iTRAQ4plex)		5.01	68	2	1018.05
									FVEGLPINDFSR	3	N-Term(iTRAQ4plex		4.07	68	2	769.41
									ENFSCLTR	1	N-Term(iTRAQ4plex); C5(Methylthio			31		
30089937	TMEM132A	transmembrane protein 132A isoform b	14.17	0	10	25		110								
									AVSVEAAVTPAEPYAR	4	N-Term(iTRAQ4plex		6.11	74	2	887.98
									AEELVNTAPLTGVPQHVPVR	3	N-Term(iTRAQ4plex		5.39	49	3	757.76
									SETFLLLQPWPR	2	N-Term(iTRAQ4plex		4.86	50	2	815.96
									IELTDTTLEQVR	4	N-Term(iTRAQ4plex		4.84	67	2	781.43
									VPGPAEGPAEPAAEASDEAER	3	N-Term(iTRAQ4plex		4.35	96	2	1097.53
									SPLSDSILGEQALAVTDDK	2	N-Term(iTRAQ4plex); K19(iTRAQ4plex		4.34	78	2	1124.10

									LTVWAPLLPLR	2	N-Term(iTRAQ4plex		3.44	39	2	711.95
									FLAPFAAHPLDGGR	1	N-Term(iTRAQ4plex			63		
									VQPVMGISLTLSR	1	N-Term(iTRAQ4plex			51		
									ALIPLAK	3	N-Term(iTRAQ4plex); K7(iTRAQ4plex			32		
30089935	TMEM132A	transmembrane protein 132A isoform a	14.16	0	10	25		110.1								
									AVSVEAAVTPAEPYAR	4	N-Term(iTRAQ4plex		6.11	74	2	887.98
									AEELVNTAPLTGVPQHVPVR	3	N-Term(iTRAQ4plex		5.39	49	3	757.76
									SETFLLLQPWPR	2	N-Term(iTRAQ4plex		4.86	50	2	815.96
									IELTDTTLEQVR	4	N-Term(iTRAQ4plex		4.84	67	2	781.43
									VPGPAEGPAEPAAEASDEAER	3	N-Term(iTRAQ4plex		4.35	96	2	1097.53
									SPLSDSILGEQALAVTDDK	2	N-Term(iTRAQ4plex); K19(iTRAQ4plex		4.34	78	2	1124.10
									LTVWAPLLPLR	2	N-Term(iTRAQ4plex		3.44	39	2	711.95
									FLAPFAAHPLDGGR	1	N-Term(iTRAQ4plex			63		
									VQPVMGISLTLSR	1	N-Term(iTRAQ4plex			51		
									ALIPLAK	3	N-Term(iTRAQ4plex); K7(iTRAQ4plex			32		
260656025	CALM1	calmodulin isoform 2	14.16	0	1	2		12.9								
									VFDKDGNGYISAAELR	2	N-Term(iTRAQ4plex); K4(iTRAQ4plex		5.84	70	3	681.70
312434033	ITIH5	inter-alpha-trypsin inhibitor heavy chain H5 isoform 2	14.15	0	8	21		80.6								
									ELLSSWLQSDDEPEKER	2	N-Term(iTRAQ4plex); K15(iTRAQ4plex		6.74	47	3	783.73
									TITILINKPER	4	N-Term(iTRAQ4plex); K8(iTRAQ4plex		5.01	62	2	793.50
									DYLASHPFDTGMTLGR	2	N-Term(iTRAQ4plex		4.23	54	3	642.32
									DHLISVTPDSIR	7	N-Term(iTRAQ4plex		4.22	59	3	499.61
									SYLEITPSR	2	N-Term(iTRAQ4plex		3.5	42	2	605.33
									LWSYLTTK	2	N-Term(iTRAQ4plex); K8(iTRAQ4plex		3.4	48	2	650.38
									MDGLEEAHGMSAAMGPEPVVQSVR	1	N-Term(iTRAQ4plex			53		
									KFIILK	1	N-Term(iTRAQ4plex); K1(iTRAQ4plex); K6(iTRAQ4plex)			34		
4757900	CALR	calreticulin precursor	14.15	6	6	32	0.951	48.1								
									KVHVIFNYK	10	N-Term(iTRAQ4plex); K1(iTRAQ4plex); K9(iTRAQ4plex)	1.071	4.82	65	2	790.49
									GQTLVVQFTVK	2	N-Term(iTRAQ4plex); K11(iTRAQ4plex	1.017	4.49	73	2	754.46
									VHVIFNYK	4	N-Term(iTRAQ4plex); K8(iTRAQ4plex	0.934	4.11	47	3	436.60
									EQFLDGDGWTSR	11	N-Term(iTRAQ4plex	0.914	3.78	69	2	777.87
									HEQNIDCGGGYVK	4	N-Term(iTRAQ4plex); C7(Methylthio); K13(iTRAQ4plex)	0.809		53		
									FYALSASFEPFSNK	1	N-Term(iTRAQ4plex); K14(iTRAQ4plex	0.912		36		
62198241	CUTA	protein CutA isoform 1	14.14	0	3	15		20.9								
									IEEDSEVLMMIK	2	N-Term(iTRAQ4plex); K12(iTRAQ4plex		5.39	37	2	862.96
									TQSSLVPALTDFVR	8	N-Term(iTRAQ4plex		5.17	88	2	839.47
									GKIEEDSEVLMMIK	5	N-Term(iTRAQ4plex); K2(iTRAQ4plex); K14(iTRAQ4plex)		3.95	74	2	1027.56
34734068	FBLN1	fibulin-1 isoform A precursor	14.13	0	6	60		61.5								
									SQETGDLDVGGLQETDK	9	N-Term(iTRAQ4plex); K17(iTRAQ4plex		6.58	93	2	1040.52
									IIEVEEEQEDPYLNDR	24	N-Term(iTRAQ4plex		6.37	91	3	712.35
									CVDVDECAPPAEPCGK	2	N-Term(iTRAQ4plex); C1(Methylthio); C7(Methylthio); C14(Methylthio); K16(iTRAQ4plex)		4.79	65	2	1029.92
									TGYYFDGISR	12	N-Term(iTRAQ4plex		4.28	44	2	661.83
									DCSLPYATESK	4	N-Term(iTRAQ4plex); C2(Methylthio); K11(iTRAQ4plex)		4.16	66	2	774.37
									MCVDVNECQR	9	N-Term(iTRAQ4plex); C2(Methylthio); C8(Methylthio)		3.69	55	2	716.79
186910296	HP	haptoglobin isoform 2 preproprotein	14.12	0	5	7		38.4								
									VTSIQDWVQK	2	N-Term(iTRAQ4plex); K10(iTRAQ4plex		4.28	88	2	746.42
									VMPICLPSK	2	N-Term(iTRAQ4plex); C5(Methylthio); K9(iTRAQ4plex)		3.26	48	2	661.37
									GSFPWQAK	1	N-Term(iTRAQ4plex); K8(iTRAQ4plex			43		
									TEGDGVYTLNNEK	1	N-Term(iTRAQ4plex); K13(iTRAQ4plex			39		
4506427	RARRES2	retinoic acid receptor responder protein 2 precursor	14.11	2	2	11	1.149	18.6								
									GLQVALEEFHK	10	N-Term(iTRAQ4plex); K11(iTRAQ4plex	1.164	5.58	83	3	520.30
295986608	IGLL5	immunoglobulin lambda-like polypeptide	14.02	2	2	5	0.243	23								
									SYSCQVTHEGSTVEK	4	N-Term(iTRAQ4plex); C4(Methylthio); K15(iTRAQ4plex)	0.257	6.09	76	3	663.65
									YAASSYLSLTPEQWK	1	N-Term(iTRAQ4plex); K15(iTRAQ4plex	0.184		75		
21070982	PAM	peptidyl-glycine alpha-amidating monooxygenase isoform d preproprotein	13.98	0	11	49		98.7								
									DGNYWVTDVALHQVFK	7	N-Term(iTRAQ4plex); K16(iTRAQ4plex		6.34	71	3	727.39
									IPVDEEAFVIDFKPR	7	N-Term(iTRAQ4plex); K13(iTRAQ4plex		6.2	55	3	688.39
									NLFYLPHGLSIDK	4	N-Term(iTRAQ4plex); K13(iTRAQ4plex		5.72	67	3	602.35
									DKIPLLQQPK	2	N-Term(iTRAQ4plex); K2(iTRAQ4plex); K10(iTRAQ4plex)		5.69	30	3	538.01
									GDHVWDGNSFDSK	9	N-Term(iTRAQ4plex); K13(iTRAQ4plex		5.57	75	3	584.62
									DCSGVSLHLTR	3	N-Term(iTRAQ4plex); C2(Methylthio		4.61	51	2	689.34
									NYPMHVFAYR	5	N-Term(iTRAQ4plex		3.72	46	3	481.24
									EGPVLILGR	4	N-Term(iTRAQ4plex		3.71	47	2	549.35
									AGIEVQEIK	2	N-Term(iTRAQ4plex); K9(iTRAQ4plex		3.6	63	2	637.87
									IVQFSPSGK	3	N-Term(iTRAQ4plex); K9(iTRAQ4plex		3.52	48	2	625.87
									ANILYAWAR	3	N-Term(iTRAQ4plex		3.23	56	2	611.35
126273569	CPB2	carboxypeptidase B2 isoform a preproprotein	13.95	1	6	15	1.098	48.4								
									DTGTYGFLLPER	2	N-Term(iTRAQ4plex	1.098	5.54	68	2	756.89
									SKDHEELSLVASEAVR	4	N-Term(iTRAQ4plex); K2(iTRAQ4plex		4.18	87	2	1029.56
									IHIGSSFEK	2	N-Term(iTRAQ4plex); K9(iTRAQ4plex		3.78	54	3	435.92
									DHEELSLVASEAVR	2	N-Term(iTRAQ4plex		3.19	80	2	849.94
									YPLYVLK	4	N-Term(iTRAQ4plex); K7(iTRAQ4plex			49		
41350305	NRXN3	neurexin-3-beta isoform 2 precursor	13.89	0	4	16		47.2								
									VLNMAAENNPNIK	8	N-Term(iTRAQ4plex); K13(iTRAQ4plex		5.72	100	2	858.47
									IDSAPGLGDFLQLHIEQGK	4	N-Term(iTRAQ4plex); K19(iTRAQ4plex		5.37	85	3	776.10
									SGGLILYTWPANDRPSTR	3	N-Term(iTRAQ4plex		4.75	36	3	716.72
									LAVGFSTTVK	1	N-Term(iTRAQ4plex); K10(iTRAQ4plex			39		
16445029	IGSF8	immunoglobulin superfamily member 8	13.87	6	6	23	0.878	65								
									VLPDVLQVSAAPPGPR	4	N-Term(iTRAQ4plex	1.165	5.09	73	2	880.52
									LQAQDAGIYECHTPSTDTR	7	N-Term(iTRAQ4plex); C11(Methylthio	0.897	4.41	65	3	766.02
									LQGDAVVLK	4	N-Term(iTRAQ4plex); K9(iTRAQ4plex	0.837	4.22	52	2	615.89
									MTVHEGQELALGCLAR	2	N-Term(iTRAQ4plex); C13(Methylthio	1.017	4.15	52	3	639.99
									EVLVPEGPLYR	4	N-Term(iTRAQ4plex	0.852	3.63	50	2	708.40
									SDLAVEAGAPYAER	2	N-Term(iTRAQ4plex	0.952	3.58	64	2	796.91

156119625	ITIH1	inter-alpha-trypsin inhibitor heavy chain H1 isoform a	13.72	4	9	62	1.184	101.3								
									FAHYVVTSQVVNTANEAl	5	N-Term(iTRAQ4plex	0.988	8.07	79	3	717.37
									GSLVQASEANLQAAQDFVF	5	N-Term(iTRAQ4plex		6.6	104	2	1074.56
									QYYEGSEIVVAGR	17	N-Term(iTRAQ4plex		5.65	78	2	807.92
									QAVDTAVDGVFIF	4	N-Term(iTRAQ4plex	1.149	5.58	72	2	767.92
									TMEQFTIHLTVNPQSk	6	N-Term(iTRAQ4plex); K16(iTRAQ4plex	1.17	4.72	71	3	721.39
									LDAQASFLPK	11	N-Term(iTRAQ4plex); K10(iTRAQ4plex		4.65	61	2	689.40
									EVAFDLEIPK	7	N-Term(iTRAQ4plex); K10(iTRAQ4plex	1.355	4.37	64	2	724.91
									NHMQYEIVIK	3	N-Term(iTRAQ4plex); K10(iTRAQ4plex		4.27	46	3	521.63
									GFSLDEATNLNGGLLR	2	N-Term(iTRAQ4plex		4.21	77	2	910.99
									TMEQFTIHLTVNPQSk	2	N-Term(iTRAQ4plex); M2(Oxidation); K16(iTRAQ4plex)	0.861	3.94	39	3	726.72
21070974	PAM	peptidyl-glycine alpha-amidating monooxygenase isoform b preproprotein	13.7	0	11	49		100.8								
									DGNYWVTDVALHQVFk	7	N-Term(iTRAQ4plex); K16(iTRAQ4plex		6.34	71	3	727.39
									IPVDEEAFVIDFKPR	7	N-Term(iTRAQ4plex); K13(iTRAQ4plex		6.2	55	3	688.39
									NLFYLPHGLSIDK	4	N-Term(iTRAQ4plex); K13(iTRAQ4plex		5.72	67	3	602.35
									DKIPLLQQPK	2	N-Term(iTRAQ4plex); K2(iTRAQ4plex); K10(iTRAQ4plex)		5.69	30	3	538.01
									GDHVWDGNSFDSK	9	N-Term(iTRAQ4plex); K13(iTRAQ4plex		5.57	75	3	584.62
									DCSGVSLHLTR	3	N-Term(iTRAQ4plex); C2(Methylthio		4.61	51	2	689.34
									NYPMHVFAYF	5	N-Term(iTRAQ4plex		3.72	46	3	481.24
									EGPVLILGR	4	N-Term(iTRAQ4plex		3.71	47	2	549.35
									AGIEVQEIK	2	N-Term(iTRAQ4plex); K9(iTRAQ4plex		3.6	63	2	637.87
									IVQFSPSGK	3	N-Term(iTRAQ4plex); K9(iTRAQ4plex		3.52	48	2	625.87
									ANILYAWAR	3	N-Term(iTRAQ4plex		3.23	56	2	611.35
258679518	FXYD6	FXYD domain-containing ion transport regulator 6 precursor	13.68	1	1	1	1.206	10.5								
									EMDPFHYDYQTLR	1	N-Term(iTRAQ4plex); M2(Oxidation	1.206		31		
290656011	NEO1	neogenin isoform 2 precursor	13.64	0	16	51		154.2								
									VETQPEVQLPGPAPNLF	4	N-Term(iTRAQ4plex		6.06	71	2	995.05
									GYAIGYGIGSPHAQTIk	4	N-Term(iTRAQ4plex); K17(iTRAQ4plex		5.88	97	2	1011.06
									GMGPMSEAVQFR	6	N-Term(iTRAQ4plex		5.53	75	2	727.36
									EHNLQVLGLVK	8	N-Term(iTRAQ4plex); K11(iTRAQ4plex		5	67	2	769.47
									KDGTFLNLVSDDR	2	N-Term(iTRAQ4plex); K1(iTRAQ4plex		4.71	73	2	884.48
									ITWADNSLPK	2	N-Term(iTRAQ4plex); K10(iTRAQ4plex		4.69	48	2	716.90
									DGTFLNLVSDDRR	2	N-Term(iTRAQ4plex		4.11	65	3	551.29
									VLPDPEVISDLVFLK	4	N-Term(iTRAQ4plex); K15(iTRAQ4plex		4.1	68	2	986.58
									DVVASLVSTF	4	N-Term(iTRAQ4plex		3.99	55	2	595.85
									QLLPDGSLFISNVVHSK	3	N-Term(iTRAQ4plex); K17(iTRAQ4plex		3.97	59	3	714.75
									YSDEVELK	2	N-Term(iTRAQ4plex); K8(iTRAQ4plex		3.77	44	2	635.84
									TLSDVPSAAPQNLSLEVF	3	N-Term(iTRAQ4plex		3.46	42	2	1021.06
									NGDMVIPSDYFK	1	N-Term(iTRAQ4plex); K12(iTRAQ4plex			58		
									LTHQIQELTLDTPYYFK	2	N-Term(iTRAQ4plex); K17(iTRAQ4plex			45		
									LIVAGLPR	3	N-Term(iTRAQ4plex			40		
									LYYMEK	1	N-Term(iTRAQ4plex); K6(iTRAQ4plex			36		
4505449	NPY	neuropeptide Y preproprotein	13.4	1	1	5	1.267	10.8								
									SSPETLISDLLMR	5	N-Term(iTRAQ4plex	1.267	5.39	81	2	803.44
315139020	FKBP1A	peptidyl-prolyl cis-trans isomerase FKBP1A isoform b	13.4	0	1	2		10.8								
									GVQVETISPGDGF	2	N-Term(iTRAQ4plex			81		
33188454	PRDX2	peroxiredoxin-2 isoform c	13.38	0	2	4		15.8								
									ATAVVDGAFk	3	N-Term(iTRAQ4plex); K10(iTRAQ4plex		4.35	67	2	633.87
34734064	FBLN1	fibulin-1 isoform B precursor	13.31	0	6	60		65.4								
									SQETGDLDVGGLQETDK	9	N-Term(iTRAQ4plex); K17(iTRAQ4plex		6.58	93	2	1040.52
									IIEVEEEQEDPYLNDR	24	N-Term(iTRAQ4plex		6.37	91	3	712.35
									CVDVDECAPPAEPCGK	2	N-Term(iTRAQ4plex); C1(Methylthio); C7(Methylthio); C14(Methylthio); K16(iTRAQ4plex)		4.79	65	2	1029.92
									TGYYFDGISR	12	N-Term(iTRAQ4plex		4.28	44	2	661.83
									DCSLPYATESK	4	N-Term(iTRAQ4plex); C2(Methylthio); K11(iTRAQ4plex)		4.16	66	2	774.37
									MCVDVNECQF	9	N-Term(iTRAQ4plex); C2(Methylthio); C8(Methylthio)		3.69	55	2	716.79
256985102	TMED7-TICAM2	TRAM adaptor with GOLD domain isoform 2	13.3	0	2	3		21.2								
									QCFYEDIAQGTK	2	N-Term(iTRAQ4plex); C2(Methylthio); K12(iTRAQ4plex)		4.15	98	2	868.91
									FCFSNEFSTFTHK	1	N-Term(iTRAQ4plex); C2(Methylthio); K13(iTRAQ4plex)			50		
290655729	NEO1	neogenin isoform 3 precursor	13.24	0	16	51		158.7								
									VETQPEVQLPGPAPNLF	4	N-Term(iTRAQ4plex		6.06	71	2	995.05
									GYAIGYGIGSPHAQTIk	4	N-Term(iTRAQ4plex); K17(iTRAQ4plex		5.88	97	2	1011.06
									GMGPMSEAVQFR	6	N-Term(iTRAQ4plex		5.53	75	2	727.36
									EHNLQVLGLVK	8	N-Term(iTRAQ4plex); K11(iTRAQ4plex		5	67	2	769.47
									KDGTFLNLVSDDR	2	N-Term(iTRAQ4plex); K1(iTRAQ4plex		4.71	73	2	884.48
									ITWADNSLPK	2	N-Term(iTRAQ4plex); K10(iTRAQ4plex		4.69	48	2	716.90
									DGTFLNLVSDDRR	2	N-Term(iTRAQ4plex		4.11	65	3	551.29
									VLPDPEVISDLVFLK	4	N-Term(iTRAQ4plex); K15(iTRAQ4plex		4.1	68	2	986.58
									DVVASLVSTF	4	N-Term(iTRAQ4plex		3.99	55	2	595.85
									QLLPDGSLFISNVVHSK	3	N-Term(iTRAQ4plex); K17(iTRAQ4plex		3.97	59	3	714.75
									YSDEVELK	2	N-Term(iTRAQ4plex); K8(iTRAQ4plex		3.77	44	2	635.84
									TLSDVPSAAPQNLSLEVF	3	N-Term(iTRAQ4plex		3.46	42	2	1021.06
									NGDMVIPSDYFK	1	N-Term(iTRAQ4plex); K12(iTRAQ4plex			58		
									LTHQIQELTLDTPYYFK	2	N-Term(iTRAQ4plex); K17(iTRAQ4plex			45		
									LIVAGLPR	3	N-Term(iTRAQ4plex			40		
									LYYMEK	1	N-Term(iTRAQ4plex); K6(iTRAQ4plex			36		
4503971	GDI1	rab GDP dissociation inhibitor alpha	13.2	4	5	8	0.835	50.6								
									TFEGVDPQTTSMF	2	N-Term(iTRAQ4plex	0.709	4.61	68	2	806.90
									IICILSHPIK	2	N-Term(iTRAQ4plex); C3(Methylthio); K10(iTRAQ4plex)	0.694	4.08	40	3	490.97
									MAGTAFDFENMk	2	N-Term(iTRAQ4plex); K12(iTRAQ4plex	0.983	3.52	100	2	825.40
									TDDYLDQPCLETVNF	1	N-Term(iTRAQ4plex); C9(Methylthio	0.996		39		
54792136	FSTL4	follistatin-related protein 4 precursor	13.18	8	9	18	0.936	93								
									VYPESQAQEPGVAASLF	2	N-Term(iTRAQ4plex	0.948	5.32	80	2	973.51
									GPDVGVGESQAEEPF	4	N-Term(iTRAQ4plex	0.93	4.72	88	2	835.91
									VDLETMMPLK	2	N-Term(iTRAQ4plex); K10(iTRAQ4plex	0.821	4.52	53	2	732.91
									DSGLFGQYLLTPAR	2	N-Term(iTRAQ4plex	0.914	4.1	81	2	841.46

									YEDTGAYTCIAK	2	N-Term(iTRAQ4plex); C9(Methylthio); K12(iTRAQ4plex)		3.9	88	2	834.90
									EPPAGPAQPWGGTHR	2	N-Term(iTRAQ4plex	1.016	3.54	50	3	567.96
									EGLSSHNELLASCGK	2	N-Term(iTRAQ4plex); C13(Methylthio); K15(iTRAQ4plex)	1.057	3.48	63	3	626.98
									CHAEGIPMPR	1	N-Term(iTRAQ4plex); C1(Methylthio	0.936		35		
157311649	NEO1	neogenin isoform 1 precursor	13.14	0	16	51		159.9								
									VETQPEVQLPGPAPNLR	4	N-Term(iTRAQ4plex		6.06	71	2	995.05
									GYAIGYGIGSPHAQTIK	4	N-Term(iTRAQ4plex); K17(iTRAQ4plex		5.88	97	2	1011.06
									GMGPMSEAVQFR	6	N-Term(iTRAQ4plex		5.53	75	2	727.36
									EHNLQVLGLVK	8	N-Term(iTRAQ4plex); K11(iTRAQ4plex		5	67	2	769.47
									KDGTFLNLVSDDR	2	N-Term(iTRAQ4plex); K1(iTRAQ4plex		4.71	73	2	884.48
									ITWADNSLPK	2	N-Term(iTRAQ4plex); K10(iTRAQ4plex		4.69	48	2	716.90
									DGTFLNLVSDDRR	2	N-Term(iTRAQ4plex		4.11	65	3	551.29
									VLPDPEVISDLVFLK	4	N-Term(iTRAQ4plex); K15(iTRAQ4plex		4.1	68	2	986.58
									DVVASLVSTR	4	N-Term(iTRAQ4plex		3.99	55	2	595.85
									QLLPDGSLFISNVVHSK	3	N-Term(iTRAQ4plex); K17(iTRAQ4plex		3.97	59	3	714.75
									YSDEVELK	2	N-Term(iTRAQ4plex); K8(iTRAQ4plex		3.77	44	2	635.84
									TLSDVPSAAPQNLSLEVR	3	N-Term(iTRAQ4plex		3.46	42	2	1021.06
									NGDMVIPSDYFK	1	N-Term(iTRAQ4plex); K12(iTRAQ4plex			58		
									LTHQIQELTLDTPYYFK	2	N-Term(iTRAQ4plex); K17(iTRAQ4plex			45		
									LIVAGLPR	3	N-Term(iTRAQ4plex			40		
									LYYMEK	1	N-Term(iTRAQ4plex); K6(iTRAQ4plex			36		
4557245	ACYP1	acylphosphatase-1 isoform a	13.13	1	1	2	0.816	11.3								
									GTVQGQLQGPISK	2	N-Term(iTRAQ4plex); K13(iTRAQ4plex	0.816	5.13	79	2	800.97
310113716	LOC100505584	PREDICTED: hypothetical protein LOC100505584	13.11	0	1	2		6.2								
									CAQGCICK	2	N-Term(iTRAQ4plex); C1(Methylthio); C5(Methylthio); C7(Methylthio); K8(iTRAQ4plex)		3.26	51	2	626.26
10835085	MT1H	metallothionein-1H	13.11	0	1	2		6								
									CAQGCICK	2	N-Term(iTRAQ4plex); C1(Methylthio); C5(Methylthio); C7(Methylthio); K8(iTRAQ4plex)		3.26	51	2	626.26
5174764	MT2A	metallothionein-2	13.11	0	1	2		6								
									CAQGCICK	2	N-Term(iTRAQ4plex); C1(Methylthio); C5(Methylthio); C7(Methylthio); K8(iTRAQ4plex)		3.26	51	2	626.26
10835230	MT1G	metallothionein-1G	13.11	0	1	2		6.1								
									CAQGCICK	2	N-Term(iTRAQ4plex); C1(Methylthio); C5(Methylthio); C7(Methylthio); K8(iTRAQ4plex)		3.26	51	2	626.26
71274113	MT1A	metallothionein-1A	13.11	0	1	2		6.1								
									CAQGCICK	2	N-Term(iTRAQ4plex); C1(Methylthio); C5(Methylthio); C7(Methylthio); K8(iTRAQ4plex)		3.26	51	2	626.26
10835232	MT1X	metallothionein-1X	13.11	0	1	2		6.1								
									CAQGCICK	2	N-Term(iTRAQ4plex); C1(Methylthio); C5(Methylthio); C7(Methylthio); K8(iTRAQ4plex)		3.26	51	2	626.26
31652249	LBP	lipopolysaccharide-binding protein precursor	13.1	5	5	6	0.645	53.3								
									VQLYDLGLQIHK	2	N-Term(iTRAQ4plex); K12(iTRAQ4plex	0.698	5.83	68	3	572.34
									ITLPDFTGDLR	1	N-Term(iTRAQ4plex	0.585		60		
									ATAQMLEVMFK	1	N-Term(iTRAQ4plex); K11(iTRAQ4plex	0.645		53		
									LAEGFPLPLLK	1	N-Term(iTRAQ4plex); K11(iTRAQ4plex	0.41		46		
									SPVTLLAAVMSLPEEHNK	1	N-Term(iTRAQ4plex); K18(iTRAQ4plex	0.752		42		
126273559	CPB2	carboxypeptidase B2 isoform b	13.06	0	5	13		40.8								
									SKDHEELSLVASEAVR	4	N-Term(iTRAQ4plex); K2(iTRAQ4plex		4.18	87	2	1029.56
									IHIGSSFEK	2	N-Term(iTRAQ4plex); K9(iTRAQ4plex		3.78	54	3	435.92
									DHEELSLVASEAVR	2	N-Term(iTRAQ4plex		3.19	80	2	849.94
									YPLYVLK	4	N-Term(iTRAQ4plex); K7(iTRAQ4plex			49		
14149609	EXTL2	exostosin-like 2	13.03	3	3	8	1.008	37.4								
									LLNHYQAVPNLHK	5	N-Term(iTRAQ4plex); K13(iTRAQ4plex	0.976	6.25	63	4	459.52
									VIVVWNNIGEK	2	N-Term(iTRAQ4plex); K11(iTRAQ4plex	1.158	4.22	49	2	779.97
									APDELWNSLGPHPIPVIFK	1	N-Term(iTRAQ4plex); K19(iTRAQ4plex	1.008		59		
52426787	OMG	oligodendrocyte-myelin glycoprotein precursor	12.95	5	5	51	1.091	49.6								
									AHVIGTPCSTQISSLK	9	N-Term(iTRAQ4plex); C8(Methylthio); K16(iTRAQ4plex)	1.086	5.33	80	3	659.36
									SDTAYQWNLK	11	N-Term(iTRAQ4plex); K10(iTRAQ4plex	1.111	5.24	68	2	757.40
									ETTFGATLSK	4	N-Term(iTRAQ4plex); K10(iTRAQ4plex	0.703	4.55	69	2	671.88
									LESLPAHLPR	21	N-Term(iTRAQ4plex	1.091	4.22	52	3	426.25
									LWTVPTNMPSK	6	N-Term(iTRAQ4plex); K11(iTRAQ4plex	1.371	3.84	77	2	781.44
209413730	PVR	poliovirus receptor isoform gamma	12.91	0	3	7		39.3								
									HGESGSMAVFHQTQGPSYSESK	2	N-Term(iTRAQ4plex); K22(iTRAQ4plex		5.77	71	3	880.42
									VLAKPQNTAEVQK	2	N-Term(iTRAQ4plex); K4(iTRAQ4plex); K13(iTRAQ4plex)		3.92	54	3	620.05
									VQLTGEPVPMAR	2	N-Term(iTRAQ4plex		3.9	45	2	721.40
									VQLTGEPVPMAR	1	N-Term(iTRAQ4plex); M10(Oxidation			46		
4502719	CDH13	cadherin-13 preproprotein	12.9	7	7	33	0.846	78.2								
									TPHAEDMAELVIVGGK	3	N-Term(iTRAQ4plex); K16(iTRAQ4plex	0.749	6.79	86	2	978.03
									DIQGSLQDIFK	7	N-Term(iTRAQ4plex); K11(iTRAQ4plex	0.921	5.64	86	2	776.44
									SIVVSPILIPENQR	3	N-Term(iTRAQ4plex	0.869	4.8	60	2	855.01
									VNSDGGLVALR	2	N-Term(iTRAQ4plex	0.907	4.28	69	2	622.86
									YEVSSPYFK	12	N-Term(iTRAQ4plex); K9(iTRAQ4plex	0.846	3.98	64	2	704.37
									GDIVTVVSPALLDR	5	N-Term(iTRAQ4plex	0.845	3.69	59	2	799.97
									MTAFDADDPATDNALLR	1	N-Term(iTRAQ4plex	0.791		36		
7656955	CALY	neuron-specific vesicular protein calcyon	12.9	2	2	3	1.08	23.4								
									SILAAIGAYPLSR	2	N-Term(iTRAQ4plex	1.26	3.43	60	2	738.44
									KHGTETPAAWGDGYR	1	N-Term(iTRAQ4plex); K1(iTRAQ4plex	0.926		45		
30102944	PPIAL4A	peptidylprolyl cis-trans isomerase A-like 4B	12.8	0	2	5		18.2								
									IIPGFMCQGGDFTR	2	N-Term(iTRAQ4plex); C7(Methylthio		4.36	69	2	866.41
									TEWLDGK	3	N-Term(iTRAQ4plex); K7(iTRAQ4plex		3.28	47	2	568.82
5803187	TALDO1	transaldolase	12.76	4	4	7	0.765	37.5								
									LSSTWEGIQAGK	2	N-Term(iTRAQ4plex); K12(iTRAQ4plex	0.844	4.89	73	2	782.94
									WLHNEDQMAVEK	2	N-Term(iTRAQ4plex); K12(iTRAQ4plex	0.777	4.84	69	2	894.46
									LLGELLQDNAK	2	N-Term(iTRAQ4plex); K11(iTRAQ4plex	0.752	3.93	63	2	751.45
									LVPVLSAK	1	N-Term(iTRAQ4plex); K8(iTRAQ4plex	0.753		42		

293336314	PAM	peptidyl-glycine alpha-amidating monooxygenase isoform e preproprotein	12.74	0	11	49		108.3								
									DGNYWVTDVALHQVFK	7	N-Term(iTRAQ4plex); K16(iTRAQ4plex		6.34	71	3	727.39
									IPVDEEAFVIDFKPR	7	N-Term(iTRAQ4plex); K13(iTRAQ4plex		6.2	55	3	688.39
									NLFYLPHGLSIDK	4	N-Term(iTRAQ4plex); K13(iTRAQ4plex		5.72	67	3	602.35
									DKIPLLQQPK	2	N-Term(iTRAQ4plex); K2(iTRAQ4plex); K10(iTRAQ4plex)		5.69	30	3	538.01
									GDHVWDGNSFDSK	9	N-Term(iTRAQ4plex); K13(iTRAQ4plex		5.57	75	3	584.62
									DCSGVSLHLTR	3	N-Term(iTRAQ4plex); C2(Methylthio		4.61	51	2	689.34
									NYPMHVFAYR	5	N-Term(iTRAQ4plex		3.72	46	3	481.24
									EGPVLILGR	4	N-Term(iTRAQ4plex		3.71	47	2	549.35
									AGIEVQEIK	2	N-Term(iTRAQ4plex); K9(iTRAQ4plex		3.6	63	2	637.87
									IVQFSPSGK	3	N-Term(iTRAQ4plex); K9(iTRAQ4plex		3.52	48	2	625.87
									ANILYAWAR	3	N-Term(iTRAQ4plex		3.23	56	2	611.35
4507467	TGFBI	transforming growth factor-beta-induced protein ig-h3 precursor	12.74	8	8	17	0.881	74.6								
									YLYHGQTLETLGGK	5	N-Term(iTRAQ4plex); K14(iTRAQ4plex	0.773	6.31	91	3	623.34
									SPYQLVLQHSR	4	N-Term(iTRAQ4plex	1.066	4.6	56	3	491.28
									EGVYTVFAPTNEAFR	2	N-Term(iTRAQ4plex	0.881	4.57	68	2	922.97
									ILGDPEALR	2	N-Term(iTRAQ4plex	0.81	3.28	37	2	564.33
									GDELADSALEIFK	1	N-Term(iTRAQ4plex); K13(iTRAQ4plex	1.1		61		
									VLTDELK	1	N-Term(iTRAQ4plex); K7(iTRAQ4plex	1.025		50		
21070984	PAM	peptidyl-glycine alpha-amidating monooxygenase isoform a preproprotein	12.73	0	11	49		108.3								
									DGNYWVTDVALHQVFK	7	N-Term(iTRAQ4plex); K16(iTRAQ4plex		6.34	71	3	727.39
									IPVDEEAFVIDFKPR	7	N-Term(iTRAQ4plex); K13(iTRAQ4plex		6.2	55	3	688.39
									NLFYLPHGLSIDK	4	N-Term(iTRAQ4plex); K13(iTRAQ4plex		5.72	67	3	602.35
									DKIPLLQQPK	2	N-Term(iTRAQ4plex); K2(iTRAQ4plex); K10(iTRAQ4plex)		5.69	30	3	538.01
									GDHVWDGNSFDSK	9	N-Term(iTRAQ4plex); K13(iTRAQ4plex		5.57	75	3	584.62
									DCSGVSLHLTR	3	N-Term(iTRAQ4plex); C2(Methylthio		4.61	51	2	689.34
									NYPMHVFAYR	5	N-Term(iTRAQ4plex		3.72	46	3	481.24
									EGPVLILGR	4	N-Term(iTRAQ4plex		3.71	47	2	549.35
									AGIEVQEIK	2	N-Term(iTRAQ4plex); K9(iTRAQ4plex		3.6	63	2	637.87
									IVQFSPSGK	3	N-Term(iTRAQ4plex); K9(iTRAQ4plex		3.52	48	2	625.87
									ANILYAWAR	3	N-Term(iTRAQ4plex		3.23	56	2	611.35
209413728	PVR	poliovirus receptor isoform beta	12.63	0	3	7		40.1								
									HGESGSMAVFHQTQGPSYSESK	2	N-Term(iTRAQ4plex); K22(iTRAQ4plex		5.77	71	3	880.42
									VLAKPQNTAEVQK	2	N-Term(iTRAQ4plex); K4(iTRAQ4plex); K13(iTRAQ4plex)		3.92	54	3	620.05
									VQLTGEPVPMAR	2	N-Term(iTRAQ4plex		3.9	45	2	721.40
									VQLTGEPVPMAR	1	N-Term(iTRAQ4plex); M10(Oxidation			46		
29826291	CASC4	protein CASC4 isoform b	12.63	0	4	8		43.3								
									ELQMDPADYGK	2	N-Term(iTRAQ4plex); K11(iTRAQ4plex		4.88	70	2	777.89
									GGDAGMPGIEENDLAK	2	N-Term(iTRAQ4plex); M6(Oxidation); K16(iTRAQ4plex)		4.4	59	2	939.46
									LIPGSNLDSEPR	2	N-Term(iTRAQ4plex		3.59	55	2	721.39
									VDDLPPALR	2	N-Term(iTRAQ4plex			50		
50400081	SUMO4	small ubiquitin-related modifier 4 precursor	12.63	0	1	2		10.6								
									VAGQDGSVVQFK	2	N-Term(iTRAQ4plex); K12(iTRAQ4plex		5.61	70	2	761.93
54792069	SUMO2	small ubiquitin-related modifier 2 isoform a precursor	12.63	0	1	2		10.9								
									VAGQDGSVVQFK	2	N-Term(iTRAQ4plex); K12(iTRAQ4plex		5.61	70	2	761.93
67782307	SOD2	superoxide dismutase [Mn], mitochondrial isoform A precursor	12.61	1	2	7	0.741	24.7								
									GDVTAQIALQPALK	5	N-Term(iTRAQ4plex); K14(iTRAQ4plex	0.741	5.38	67	2	857.01
									AIWNVINWENVTER	2	N-Term(iTRAQ4plex		3.23	62	2	944.50
209693439	SNCG	gamma-synuclein	12.6	1	1	2	1.03	13.3								
									TVEEAENIAVTSGVVR	2	N-Term(iTRAQ4plex	1.03	5.86	68	2	909.49
310703621	ITIH5	inter-alpha-trypsin inhibitor heavy chain H5 isoform 1 precursor	12.53	0	9	22		104.5								
									ELLSSWLQSDDEPEKER	2	N-Term(iTRAQ4plex); K15(iTRAQ4plex		6.74	47	3	783.73
									TITILINKPER	4	N-Term(iTRAQ4plex); K8(iTRAQ4plex		5.01	62	2	793.50
									DYLASHPFDTGMTLGR	2	N-Term(iTRAQ4plex		4.23	54	3	642.32
									DHLISVTPDSIR	7	N-Term(iTRAQ4plex		4.22	59	3	499.61
									SYLEITPSR	2	N-Term(iTRAQ4plex		3.5	42	2	605.33
									LWSYLTTK	2	N-Term(iTRAQ4plex); K8(iTRAQ4plex		3.4	48	2	650.38
									MDGLEEAHGMSAAMGPEPVVQSVR	1	N-Term(iTRAQ4plex			53		
									YEHSISVRPQQLSGR	1	N-Term(iTRAQ4plex			40		
									KFIILK	1	N-Term(iTRAQ4plex); K1(iTRAQ4plex); K6(iTRAQ4plex)			34		
5031809	ISLR	immunoglobulin superfamily containing leucine rich repeat protein precursor	12.38	4	4	14	0.79	46								
									TVAAGALASLSHLK	7	N-Term(iTRAQ4plex); K14(iTRAQ4plex	0.875	9.13	108	3	543.00
									ALPGTPVASSQPR	4	N-Term(iTRAQ4plex	0.719	4.18	74	2	712.90
									LPGLPEGAFR	2	N-Term(iTRAQ4plex	0.79	3.14	42	2	600.85
									EVPLLQSLWLAHNEIR	1	N-Term(iTRAQ4plex	0.64		54		
4504183	GSTP1	glutathione S-transferase P	12.38	2	2	5	0.876	23.3								
									MLLADQGQSWK	4	N-Term(iTRAQ4plex); K11(iTRAQ4plex	0.834	4.81	69	2	782.92
									EEVVTVETWQEGSLK	1	N-Term(iTRAQ4plex); K15(iTRAQ4plex	0.997		62		
70778918	ITIH2	inter-alpha-trypsin inhibitor heavy chain H2	12.37	10	10	57	1.176	106.4								
									IQPSGGTNINEALLR	18	N-Term(iTRAQ4plex	1.154	6.28	92	2	863.98
									IYGNQDTSSQLK	2	N-Term(iTRAQ4plex); K12(iTRAQ4plex	1.173	5.22	85	2	821.44
									AEDHFSVIDFNQNIR	3	N-Term(iTRAQ4plex	1.198	5.01	69	2	974.98
									SSALDMENFR	8	N-Term(iTRAQ4plex	1.223	4.79	51	2	657.32
									VQFELHYQEVK	8	N-Term(iTRAQ4plex); K11(iTRAQ4plex	1.22	4.54	68	3	569.98
									FYNQVSTPLLR	5	N-Term(iTRAQ4plex	1.275	4.03	42	2	741.42
									TILDDLR	5	N-Term(iTRAQ4plex	1.179	3.24	41	2	495.29
									ETAVDGELVVLYDVK	6	N-Term(iTRAQ4plex); K15(iTRAQ4plex	1.031	3.23	72	2	969.54
									KFYNQVSTPLLR	1	N-Term(iTRAQ4plex); K1(iTRAQ4plex	1.311		38		
310129614	LOC100290936	PREDICTED: phosphoglycerate mutase 1-like	12.35	0	1	2		19.4								
									SYDVPPPPMEPDHPFYSNISK	2	N-Term(iTRAQ4plex); K21(iTRAQ4plex		4.76	46	3	902.45
310124843	LOC100507705	PREDICTED: class I histocompatibility antigen, Gogo-B*0103 alpha chain-like isoform 10	12.22	0	3	5		34.5								
									SWTAADTAAQITQR	2	N-Term(iTRAQ4plex		4.98	72	2	832.43
									APWVEQEGPEYWDR	2	N-Term(iTRAQ4plex		3.42	63	2	953.45
									DYIALNEDLR	2	N-Term(iTRAQ4plex		3.38	41	2	683.36

296317276	CD99L2	CD99 antigen-like protein 2 isoform 4 precursor	12.17	0	1	5		20								
									APAKPPGSGLDLADALDDQDDGF	5	N-Term(iTRAQ4plex); K4(iTRAQ4plex		4.14	78	3	861.44
56788391	MOG	myelin-oligodendrocyte glycoprotein isoform beta2 precursor	12.14	0	2	6		23.2								
									ALVGDEVELPCR	4	N-Term(iTRAQ4plex); C11(Methylthio		5.04	49	2	745.88
									DHSYQEEAAMELK	2	N-Term(iTRAQ4plex); K13(iTRAQ4plex		4.53	49	3	613.63
4826762	HP	haptoglobin isoform 1 preproprotein	12.07	0	5	7		45.2								
									VTSIQDWVQK	2	N-Term(iTRAQ4plex); K10(iTRAQ4plex		4.28	88	2	746.42
									VMPICLPSK	2	N-Term(iTRAQ4plex); C5(Methylthio); K9(iTRAQ4plex)		3.26	48	2	661.37
									GSFPWQAK	1	N-Term(iTRAQ4plex); K8(iTRAQ4plex			43		
									TEGDGVYTLNNEK	1	N-Term(iTRAQ4plex); K13(iTRAQ4plex			39		
4504383	HGFAC	hepatocyte growth factor activator preproprotein	12.06	6	6	9	1.114	70.6								
									TTDVTQTFGIEK	2	N-Term(iTRAQ4plex); K12(iTRAQ4plex	1.071	5.4	79	2	814.44
									EALVPLVADHK	2	N-Term(iTRAQ4plex); K11(iTRAQ4plex	1.196	4.89	42	3	493.96
									SQFVQPICLPEPGSTFPAGHK	2	N-Term(iTRAQ4plex); C8(Methylthio); K21(iTRAQ4plex)	0.912	3.35	45	3	858.78
									SDACQGDSGGPLACEK	1	N-Term(iTRAQ4plex); C4(Methylthio); C14(Methylthio); K16(iTRAQ4plex)	1.158		54		
									LEACESLTR	1	N-Term(iTRAQ4plex); C4(Methylthio	1.244		33		
									WCATTHNYDR	1	N-Term(iTRAQ4plex); C2(Methylthio	1.061		27		
4506041	PRELP	prolargin precursor	12.04	4	4	6	0.856	43.8								
									NLMQLNLAHNILR	2	N-Term(iTRAQ4plex	0.843	4.9	68	3	565.32
									NQLEEVPSALPR	2	N-Term(iTRAQ4plex	0.869	4.75	48	2	748.91
									IPPGVFSK	1	N-Term(iTRAQ4plex); K8(iTRAQ4plex	0.901		49		
									LENLLLLDLQHNR	1	N-Term(iTRAQ4plex	0.641		44		
4503725	FKBP1A	peptidyl-prolyl cis-trans isomerase FKBP1A isoform a	12.04	0	1	2		11.9								
									GVQVETISPGDGR	2	N-Term(iTRAQ4plex			81		
56788383	MOG	myelin-oligodendrocyte glycoprotein isoform alpha2 precursor	12.02	0	2	6		23.6								
									ALVGDEVELPCR	4	N-Term(iTRAQ4plex); C11(Methylthio		5.04	49	2	745.88
									DHSYQEEAAMELK	2	N-Term(iTRAQ4plex); K13(iTRAQ4plex		4.53	49	3	613.63
209413732	PVR	poliovirus receptor isoform delta	11.99	0	3	7		42.8								
									HGESGSMAVFHQTQGPSYSESK	2	N-Term(iTRAQ4plex); K22(iTRAQ4plex		5.77	71	3	880.42
									VLAKPQNTAEVQK	2	N-Term(iTRAQ4plex); K4(iTRAQ4plex); K13(iTRAQ4plex)		3.92	54	3	620.05
									VQLTGEPVPMAR	2	N-Term(iTRAQ4plex		3.9	45	2	721.40
									VQLTGEPVPMAR	1	N-Term(iTRAQ4plex); M10(Oxidation			46		
4885099	CA3	carbonic anhydrase 3	11.92	2	2	3	0.18	29.5								
									HDPSLQPWSVSYDGGSAK	2	N-Term(iTRAQ4plex); K18(iTRAQ4plex	0.264	4.34	62	3	740.37
									YAAELHLVHWNPK	1	N-Term(iTRAQ4plex); K13(iTRAQ4plex	0.123		41		
28610149	IL6ST	interleukin-6 receptor subunit beta isoform 2 precursor	11.85	0	3	9		37.5								
									VTSDHINFDPVYK	2	N-Term(iTRAQ4plex); K13(iTRAQ4plex		5.2	39	3	608.32
									DASTWSQIPPEDTASTR	4	N-Term(iTRAQ4plex		4.4	59	2	1003.49
									LTWTNPSIK	3	N-Term(iTRAQ4plex); K9(iTRAQ4plex		4.27	64	2	674.40
66864913	NRP1	neuropilin-1 isoform c	11.82	0	5	9		68.3								
									SFEGNNNYDTPELR	2	N-Term(iTRAQ4plex		6.17	74	2	900.42
									IMINFNPHFDLEDR	3	N-Term(iTRAQ4plex		3.47	56	3	635.65
									FVSDYETHGAGFSIR	2	N-Term(iTRAQ4plex			39		
									LEIWDGFPDVGPHIGR	1	N-Term(iTRAQ4plex			35		
223029474	NELL2	protein kinase C-binding protein NELL2 isoform c	11.78	0	9	91		91.1								
									ASTATAEQFFQK	25	N-Term(iTRAQ4plex); K12(iTRAQ4plex		6.11	104	2	808.93
									IMELQDILAK	5	N-Term(iTRAQ4plex); K10(iTRAQ4plex		4.91	69	2	731.44
									TCLDEMNVVR	2	N-Term(iTRAQ4plex); C2(Methylthio		4.2	31	2	685.32
									SALAYVDGK	2	N-Term(iTRAQ4plex); K9(iTRAQ4plex		3.94	51	2	606.35
									VVEKPSTDLPLGTTFWLGQR	5	N-Term(iTRAQ4plex); K4(iTRAQ4plex		3.92	61	3	844.81
									AFLFQDTPR	31	N-Term(iTRAQ4plex		3.78	43	2	619.83
									FTGSSWIK	16	N-Term(iTRAQ4plex); K8(iTRAQ4plex		3.64	66	2	607.35
									GYDFCSER	4	N-Term(iTRAQ4plex); C5(Methylthio		3.34	33	2	583.74
									HEFTILVTLK	1	N-Term(iTRAQ4plex); K10(iTRAQ4plex			75		
7706573	SDF4	45 kDa calcium-binding protein isoform 1 precursor	11.78	0	3	13		39.6								
									TAEHFQEAMEESK	2	N-Term(iTRAQ4plex); K13(iTRAQ4plex		6.86	60	3	608.96
									DLGGFDEDAEPR	9	N-Term(iTRAQ4plex		5.26	71	2	732.84
									AVDPDGDGHVSWDEYK	2	N-Term(iTRAQ4plex); K16(iTRAQ4plex		3.92	52	3	693.33
7657138	GOLIM4	Golgi integral membrane protein 4	11.78	4	4	8	1.226	81.8								
									ALEEEEMEQVGQAEHLEEEHDPSPEEQD R	2	N-Term(iTRAQ4plex	1.289	5.56	46	4	891.64
									AAVEDINPADDPNNQGEDEFEEAEQVI	2	N-Term(iTRAQ4plex	0.742	4.19	75	3	1049.14
									LAVQQVEEAQQLR	2	N-Term(iTRAQ4plex	1.707	3.99	67	2	828.46
									FQSPYEEQLEQQR	2	N-Term(iTRAQ4plex	1.167	3.55	97	2	913.45
310124849	LOC100507705	PREDICTED: class I histocompatibility antigen, Gogo-B*0103 alpha chain-like isoform 9, partial	11.76	0	3	5		36.7								
									SWTAADTAAQITQR	2	N-Term(iTRAQ4plex		4.98	72	2	832.43
									APWVEQEGPEYWDR	2	N-Term(iTRAQ4plex		3.42	63	2	953.45
									DYIALNEDLR	2	N-Term(iTRAQ4plex		3.38	41	2	683.36
5453766	NELL2	protein kinase C-binding protein NELL2 isoform b precursor	11.76	0	9	91		91.3								
									ASTATAEQFFQK	25	N-Term(iTRAQ4plex); K12(iTRAQ4plex		6.11	104	2	808.93
									IMELQDILAK	5	N-Term(iTRAQ4plex); K10(iTRAQ4plex		4.91	69	2	731.44
									TCLDEMNVVR	2	N-Term(iTRAQ4plex); C2(Methylthio		4.2	31	2	685.32
									SALAYVDGK	2	N-Term(iTRAQ4plex); K9(iTRAQ4plex		3.94	51	2	606.35
									VVEKPSTDLPLGTTFWLGQR	5	N-Term(iTRAQ4plex); K4(iTRAQ4plex		3.92	61	3	844.81
									AFLFQDTPR	31	N-Term(iTRAQ4plex		3.78	43	2	619.83
									FTGSSWIK	16	N-Term(iTRAQ4plex); K8(iTRAQ4plex		3.64	66	2	607.35
									GYDFCSER	4	N-Term(iTRAQ4plex); C5(Methylthio		3.34	33	2	583.74
									HEFTILVTLK	1	N-Term(iTRAQ4plex); K10(iTRAQ4plex			75		
51873067	QSOX1	sulfhydryl oxidase 1 isoform b	11.75	0	5	12		66.8								
									EVALDLSQHK	3	N-Term(iTRAQ4plex); K10(iTRAQ4plex		5.01	66	3	476.61
									LAGAPSEDPQFPK	4	N-Term(iTRAQ4plex); K13(iTRAQ4plex		4.89	44	2	822.95
									LEEIDGFFAR	2	N-Term(iTRAQ4plex		3.47	40	2	670.85
									VGSPNAAVLWLWSSHNR	2	N-Term(iTRAQ4plex		3.36	50	3	680.03

									DVQNVAAAPELAMGALELESF	1	N-Term(iTRAQ4plex			36		
56788393	MOG	myelin-oligodendrocyte glycoprotein isoform beta5 precursor	11.74	0	2	6		24.1								
									ALVGDEVELPCR	4	N-Term(iTRAQ4plex); C11(Methylthio		5.04	49	2	745.88
									DHSYQEEAAMELK	2	N-Term(iTRAQ4plex); K13(iTRAQ4plex		4.53	49	3	613.63
21071010	TCN2	transcobalamin-2 isoform 1 precursor	11.71	1	4	6	0.923	47.5								
									DPNTPLLQGIADYRPK	2	N-Term(iTRAQ4plex); K16(iTRAQ4plex		7.56	42	3	696.05
									LGQHLLPWMDR	2	N-Term(iTRAQ4plex		3.84	43	2	755.41
									LSLEHLNPSIYVGLR	1	N-Term(iTRAQ4plex			36		
49355778	ITIH5	inter-alpha-trypsin inhibitor heavy chain H5 isoform 3 precursor	11.68	0	6	14		78.4								
									ELLSSWLQSDDEPEKER	2	N-Term(iTRAQ4plex); K15(iTRAQ4plex		6.74	47	3	783.73
									DHLISVTPDSIR	7	N-Term(iTRAQ4plex		4.22	59	3	499.61
									LWSYLTTK	2	N-Term(iTRAQ4plex); K8(iTRAQ4plex		3.4	48	2	650.38
									MDGLEEAHGMSAAMGPEPVVQSVF	1	N-Term(iTRAQ4plex			53		
									YEHSISVRPQQLSGR	1	N-Term(iTRAQ4plex			40		
									KFIILK	1	N-Term(iTRAQ4plex); K1(iTRAQ4plex); K6(iTRAQ4plex)			34		
21361657	PDIA3	protein disulfide-isomerase A3 precursor	11.68	5	5	10	0.852	56.7								
									TADGIVSHLK	3	N-Term(iTRAQ4plex); K10(iTRAQ4plex	0.933	4.21	56	3	443.60
									FVMQEEFSR	2	N-Term(iTRAQ4plex	0.794	3.88	48	2	658.82
									EATNPPVIQEEKPK	2	N-Term(iTRAQ4plex); K12(iTRAQ4plex); K14(iTRAQ4plex)	0.938	3.37	47	3	671.39
									MDATANDVPSPYEVF	2	N-Term(iTRAQ4plex	0.846	3.15	43	2	904.94
310124837	LOC100507705	PREDICTED: class I histocompatibility antigen, Gogo-B*0103 alpha chain-like isoform 5	11.66	0	3	5		37								
									SWTAADTAAQITQF	2	N-Term(iTRAQ4plex		4.98	72	2	832.43
									APWVEQEGPEYWDR	2	N-Term(iTRAQ4plex		3.42	63	2	953.45
									DYIALNEDLR	2	N-Term(iTRAQ4plex		3.38	41	2	683.36
48928058	SUMO3	small ubiquitin-related modifier 3 precursor	11.65	0	1	2		11.6								
									VAGQDGSVVQFK	2	N-Term(iTRAQ4plex); K12(iTRAQ4plex		5.61	70	2	761.93
13325075	QSOX1	sulfhydryl oxidase 1 isoform a	11.65	1	6	18	0.947	82.5								
									EVALDLSQHK	3	N-Term(iTRAQ4plex); K10(iTRAQ4plex		5.01	66	3	476.61
									LAGAPSEDPQFPK	4	N-Term(iTRAQ4plex); K13(iTRAQ4plex		4.89	44	2	822.95
									AAPGQEPPEHMAELQF	6	N-Term(iTRAQ4plex	0.947	4.3	62	3	635.65
									LEEIDGFFAR	2	N-Term(iTRAQ4plex		3.47	40	2	670.85
									VGSPNAAVLWLWSSHNF	2	N-Term(iTRAQ4plex		3.36	50	3	680.03
									DVQNVAAAPELAMGALELESF	1	N-Term(iTRAQ4plex			36		
194097471	PLEKHB1	pleckstrin homology domain-containing family B member 1 isoform d	11.64	0	2	6		21.2								
									IGPECHDVQPPEGR	5	N-Term(iTRAQ4plex); C5(Methylthio		5.14	45	3	575.27
									VLIHFNVR	1	N-Term(iTRAQ4plex			36		
300360532	GSTO1	glutathione S-transferase omega-1 isoform 2	11.54	0	2	3		23.7								
									GSAPPGPVPEGSIR	2	N-Term(iTRAQ4plex			46		
									LNECVDHTPK	1	N-Term(iTRAQ4plex); C4(Methylthio); K10(iTRAQ4plex)			35		
261878461	RGMA	repulsive guidance molecule A isoform 2	11.52	0	3	7		47.5								
									VYQAEMDELPAAFVDGSK	2	N-Term(iTRAQ4plex); K18(iTRAQ4plex		4.84	84	2	1129.56
									GCPLNQQIDFQAFHTNAEGTGAF	3	N-Term(iTRAQ4plex); C2(Methylthio		4.18	73	3	889.09
									YIGTTIVVR	2	N-Term(iTRAQ4plex		3.66	51	2	583.36
74048573	PLGLB1	plasminogen-related protein B precursor	11.46	0	1	9		11								
									EQQCVIMAENR	9	N-Term(iTRAQ4plex); C4(Methylthio		5.04	57	2	755.85
14211536	NRXN2	neurexin-2-beta isoform alpha-1 precursor	11.45	0	17	47		184.9								
									VLALAAESDPNVF	2	N-Term(iTRAQ4plex		5.22	43	2	749.92
									LGERPPALLGSQGLR	7	N-Term(iTRAQ4plex		5.18	56	3	570.00
									LEFHNIETGIMTER	4	N-Term(iTRAQ4plex		5.01	70	3	611.98
									SADYVNLSLK	4	N-Term(iTRAQ4plex); K10(iTRAQ4plex		4.65	71	2	699.40
									AIVADPVTFK	4	N-Term(iTRAQ4plex); K10(iTRAQ4plex		4.31	55	2	674.91
									VVDEWLLDK	4	N-Term(iTRAQ4plex); K9(iTRAQ4plex		4.3	64	2	702.91
									GELYIGGLSK	2	N-Term(iTRAQ4plex); K10(iTRAQ4plex		3.8	42	2	662.88
									LSALTLSTVK	2	N-Term(iTRAQ4plex); K10(iTRAQ4plex		3.54	42	2	660.92
									LPDLIADALHR	2	N-Term(iTRAQ4plex		3.48	65	3	459.94
									GLLANLK	5	N-Term(iTRAQ4plex); K7(iTRAQ4plex		3.46	37	2	508.83
									QLTIFNSQAAIK	2	N-Term(iTRAQ4plex); K12(iTRAQ4plex		3.19	63	2	811.48
									WHMVLLTR	4	N-Term(iTRAQ4plex		3.16	43	2	600.35
									VDLPLPPEVWTAALR	1	N-Term(iTRAQ4plex			81		
									TGSISLDFR	1	N-Term(iTRAQ4plex			50		
									GATADPLCAPAF	1	N-Term(iTRAQ4plex); C8(Methylthio			43		
									FTLSCAEPATLQLDTPVADDF	1	N-Term(iTRAQ4plex); C5(Methylthio			41		
									FNDNAWHDVF	1	N-Term(iTRAQ4plex			31		
223029476	NELL2	protein kinase C-binding protein NELL2 isoform d	11.44	0	9	91		94.1								
									ASTATAEQFFQK	25	N-Term(iTRAQ4plex); K12(iTRAQ4plex		6.11	104	2	808.93
									IMELQDILAK	5	N-Term(iTRAQ4plex); K10(iTRAQ4plex		4.91	69	2	731.44
									TCLDEMNVVF	2	N-Term(iTRAQ4plex); C2(Methylthio		4.2	31	2	685.32
									SALAYVDGK	2	N-Term(iTRAQ4plex); K9(iTRAQ4plex		3.94	51	2	606.35
									VVEKPSTDLPLGTTFWLGQR	5	N-Term(iTRAQ4plex); K4(iTRAQ4plex		3.92	61	3	844.81
									AFLFQDTPR	31	N-Term(iTRAQ4plex		3.78	43	2	619.83
									FTGSSWIK	16	N-Term(iTRAQ4plex); K8(iTRAQ4plex		3.64	66	2	607.35
									GYDFCSER	4	N-Term(iTRAQ4plex); C5(Methylthio		3.34	33	2	583.74
									HEFTILVTLK	1	N-Term(iTRAQ4plex); K10(iTRAQ4plex			75		
18201909	VCAM1	vascular cell adhesion protein 1 isoform b precursor	11.44	0	7	17		71.2								
									LHIDEMDSVPTVR	2	N-Term(iTRAQ4plex		4.69	73	2	828.43
									GIQVEIYSFPK	2	N-Term(iTRAQ4plex); K11(iTRAQ4plex		4.68	61	2	784.95
									SLEMTFIPTIEDTGK	2	N-Term(iTRAQ4plex); K15(iTRAQ4plex		4.1	51	2	985.52
									LHIDDMEFEPK	2	N-Term(iTRAQ4plex); K11(iTRAQ4plex		3.65	73	2	831.43
									SQEFLEDADR	2	N-Term(iTRAQ4plex		3.64	38	2	677.32
									LEIELLK	4	N-Term(iTRAQ4plex); K7(iTRAQ4plex		3.49	54	2	573.37
									LEIDLLK	3	N-Term(iTRAQ4plex); K7(iTRAQ4plex		3.28	39	2	566.36
51317358	PIK3IP1	phosphoinositide-3-kinase-interacting protein 1 isoform 1	11.41	0	2	10		28.2								
									GPWCYVSGEAGVPEK	6	N-Term(iTRAQ4plex); C4(Methylthio); K15(iTRAQ4plex)		5.42	68	2	956.96
									SEAAAVQPVIGISQF	4	N-Term(iTRAQ4plex		4.57	65	2	835.47
21166380	NRXN2	neurexin-2-beta isoform alpha-2 precursor	11.39	0	16	43		177.2								
									VLALAAESDPNVF	2	N-Term(iTRAQ4plex		5.22	43	2	749.92
									LGERPPALLGSQGLR	7	N-Term(iTRAQ4plex		5.18	56	3	570.00

									LEFHNIETGIMTER	4	N-Term(iTRAQ4plex		5.01	70	3	611.98
									SADYVNLSLK	4	N-Term(iTRAQ4plex); K10(iTRAQ4plex		4.65	71	2	699.40
									AIVADPVTFK	4	N-Term(iTRAQ4plex); K10(iTRAQ4plex		4.31	55	2	674.91
									GELYIGGLSK	2	N-Term(iTRAQ4plex); K10(iTRAQ4plex		3.8	42	2	662.88
									LSALTLSTVK	2	N-Term(iTRAQ4plex); K10(iTRAQ4plex		3.54	42	2	660.92
									LPDLIADALHR	2	N-Term(iTRAQ4plex		3.48	65	3	459.94
									GLLANLK	5	N-Term(iTRAQ4plex); K7(iTRAQ4plex		3.46	37	2	508.83
									QLTIFNSQAAIK	2	N-Term(iTRAQ4plex); K12(iTRAQ4plex		3.19	63	2	811.48
									WHMVLLTR	4	N-Term(iTRAQ4plex		3.16	43	2	600.35
									VDLPLPPEVWTAALR	1	N-Term(iTRAQ4plex			81		
									TGSISLDFR	1	N-Term(iTRAQ4plex			50		
									GATADPLCAPAF	1	N-Term(iTRAQ4plex); C8(Methylthio			43		
									FTLSCAEPATLQLDTPVADDF	1	N-Term(iTRAQ4plex); C5(Methylthio			41		
									FNDNAWHDVF	1	N-Term(iTRAQ4plex			31		
42560231	PVRL1	poliovirus receptor-related protein 1 isoform 2 precursor	11.35	0	4	13		50.7								
									VEFLRPSFTDGTIR	5	N-Term(iTRAQ4plex		4.04	43	3	594.66
									LKGEAEYQEIR	4	N-Term(iTRAQ4plex); K2(iTRAQ4plex		3.78	57	2	812.45
									QNVAIYNPSMGVSVLAPYF	2	N-Term(iTRAQ4plex		3.23	40	2	1112.09
									ITQVTWQK	2	N-Term(iTRAQ4plex); K8(iTRAQ4plex			56		
23110955	CTSH	cathepsin H preproprotein	11.34	3	3	7	1.196	37.4								
									GIMGEDTYPYQGK	4	N-Term(iTRAQ4plex); K13(iTRAQ4plex	1.196	6.21	84	2	873.93
									VNHAVLAVGYGEK	2	N-Term(iTRAQ4plex); K13(iTRAQ4plex	1.387	6.04	87	2	822.97
									GTGPYPPSVDWR	1	N-Term(iTRAQ4plex	0.936		49		
18699732	SDF4	45 kDa calcium-binding protein isoform 2 precursor	11.33	0	3	13		41.8								
									TAEHFQEAMEESK	2	N-Term(iTRAQ4plex); K13(iTRAQ4plex		6.86	60	3	608.96
									DLGGFDEDAEPR	9	N-Term(iTRAQ4plex		5.26	71	2	732.84
									AVDPDGDGHVSWDEYK	2	N-Term(iTRAQ4plex); K16(iTRAQ4plex		3.92	52	3	693.33
289547636	C1RL	complement C1r subcomponent-like protein precursor	11.29	4	4	15	0.969	53.5								
									WILTAAHTIYPK	9	N-Term(iTRAQ4plex); K12(iTRAQ4plex	0.951	4.52	51	3	568.00
									LGNFPWQAFTSIHGR	2	N-Term(iTRAQ4plex	0.989	3.34	56	3	625.66
									VLSYVDWIK	3	N-Term(iTRAQ4plex); K9(iTRAQ4plex	1.22	3.16	77	2	705.91
									QRPEVFSDNMFCVGDETQF	1	N-Term(iTRAQ4plex); C12(Methylthio	0.768		46		
17136148	ATP6AP1	V-type proton ATPase subunit S1 precursor	11.28	4	4	13	0.886	52								
									LGASPLHVDLATLF	5	N-Term(iTRAQ4plex	1.53	5.23	58	3	536.32
									LPYTASSGLMAPR	4	N-Term(iTRAQ4plex	0.836	4.26	60	2	754.41
									NVLLFLQDK	2	N-Term(iTRAQ4plex); K9(iTRAQ4plex	0.347		71		
									LPYTASSGLMAPR	1	N-Term(iTRAQ4plex); M10(Oxidation	0.769		56		
209413726	PVR	poliovirus receptor isoform alpha	11.27	0	3	7		45.3								
									HGESGSMAVFHQTQGPSYSESK	2	N-Term(iTRAQ4plex); K22(iTRAQ4plex		5.77	71	3	880.42
									VLAKPQNTAEVQK	2	N-Term(iTRAQ4plex); K4(iTRAQ4plex); K13(iTRAQ4plex)		3.92	54	3	620.05
									VQLTGEPVPMAF	2	N-Term(iTRAQ4plex		3.9	45	2	721.40
									VQLTGEPVPMAF	1	N-Term(iTRAQ4plex); M10(Oxidation			46		
12383056	MAP1LC3B	microtubule-associated proteins 1A/1B light chain 3B	11.2	0	1	1		14.7								
									FLVPDHVNMSELIK	1	N-Term(iTRAQ4plex); K14(iTRAQ4plex			62		
148277548	MAP1LC3B2	microtubule-associated proteins 1A/1B light chain 3 beta 2 precursor	11.2	0	1	1		14.6								
									FLVPDHVNMSELIK	1	N-Term(iTRAQ4plex); K14(iTRAQ4plex			62		
68160947	SCG2	secretogranin-2 precursor	11.18	7	7	49	0.953	70.9								
									LYTDDEDDIYK	14	N-Term(iTRAQ4plex); K11(iTRAQ4plex	0.861	5.84	75	2	839.41
									QYWDEDLLMK	5	N-Term(iTRAQ4plex); K10(iTRAQ4plex	1.174	5.07	63	2	814.92
									DSLSEEDWMR	16	N-Term(iTRAQ4plex	0.866	5.05	64	2	706.32
									VLEYLNQEK	4	N-Term(iTRAQ4plex); K9(iTRAQ4plex	1.062	4.19	51	2	712.40
									ALEYIENLR	2	N-Term(iTRAQ4plex	1.315	3.87	47	2	632.85
									IILEALR	6	N-Term(iTRAQ4plex	0.999		39		
									QYWDEDLLMK	1	N-Term(iTRAQ4plex); M9(Oxidation); K10(iTRAQ4plex)	0.974		38		
									HMQFPPMYEENSR	1	N-Term(iTRAQ4plex	1.698		29		
66912178	NRP1	neuropilin-1 isoform b	11.18	0	5	9		71.9								
									SFEGNNNYDTPELR	2	N-Term(iTRAQ4plex		6.17	74	2	900.42
									IMINFNPHFDLEDR	3	N-Term(iTRAQ4plex		3.47	56	3	635.65
									FVSDYETHGAGFSIF	2	N-Term(iTRAQ4plex			39		
									LEIWDGFPDVGPHIGR	1	N-Term(iTRAQ4plex			35		
148664242	CNTNAP4	contactin-associated protein-like 4 isoform 2	11.17	0	12	34		137.4								
									NMADGQLHHIMINF	8	N-Term(iTRAQ4plex		6.74	72	4	449.23
									YQEPDVVNFDFK	2	N-Term(iTRAQ4plex); K12(iTRAQ4plex		5.58	67	2	894.96
									DGAGGWSPLVSNK	2	N-Term(iTRAQ4plex); K13(iTRAQ4plex		5.01	43	2	788.42
									TMQSDGILLHR	2	N-Term(iTRAQ4plex		4.94	56	3	472.26
									EYLSVIIAK	2	N-Term(iTRAQ4plex); K9(iTRAQ4plex		4.19	47	2	662.41
									AQVTPEVQPGCF	2	N-Term(iTRAQ4plex); C11(Methylthio		4.18	52	2	737.87
									NMADGQLHHIMINF	3	N-Term(iTRAQ4plex); M11(Oxidation		4.04	52	3	603.97
									YQWLQIDLGER	2	N-Term(iTRAQ4plex		4.04	44	2	782.91
									QDGTPLSWWVGR	2	N-Term(iTRAQ4plex		3.58	55	2	773.40
									SPLGGFQGCMR	2	N-Term(iTRAQ4plex); C9(Methylthio		3.42	50	2	671.81
									LFLLINSGEAK	1	N-Term(iTRAQ4plex); K11(iTRAQ4plex			80		
									FIPLEWNPK	5	N-Term(iTRAQ4plex); K9(iTRAQ4plex			58		
									EGPNGDHITLQLF	1	N-Term(iTRAQ4plex			37		
56788389	MOG	myelin-oligodendrocyte glycoprotein isoform alpha3 precursor	11.16	0	2	6		25.3								
									ALVGDEVELPCR	4	N-Term(iTRAQ4plex); C11(Methylthio		5.04	49	2	745.88
									DHSYQEEAAMELK	2	N-Term(iTRAQ4plex); K13(iTRAQ4plex		4.53	49	3	613.63
32996709	TMED7	transmembrane emp24 domain-containing protein 7 precursor	11.16	0	2	3		25.2								
									QCFYEDIAQGTK	2	N-Term(iTRAQ4plex); C2(Methylthio); K12(iTRAQ4plex)		4.15	98	2	868.91
									FCFSNEFSTFTHK	1	N-Term(iTRAQ4plex); C2(Methylthio); K13(iTRAQ4plex)			50		
14249738	GNPTG	N-acetylglucosamine-1-phosphotransferase subunit gamma precursor	11.15	4	4	11	0.914	34								
									RDPSPVSGPVHLFR	2	N-Term(iTRAQ4plex	0.838	5.88	73	3	569.99
									TLFEDAGYLK	6	N-Term(iTRAQ4plex); K10(iTRAQ4plex	0.797	4.35	61	2	722.90
									CFSLVESTYK	2	N-Term(iTRAQ4plex); C1(Methylthio); K10(iTRAQ4plex)	1.081	3.85	73	2	755.88

29029550	CECR1	adenosine deaminase CECR1 isoform a precursor	11.15	1	5	8	1.102	58.9								
									DVAVIAESIR	2	N-Term(iTRAQ4plex		4.24	41	2	608.85
									LPYFFHAGETDWQGTSIDR	2	N-Term(iTRAQ4plex		3.59	45	3	795.39
									FIADVATK	2	N-Term(iTRAQ4plex); K8(iTRAQ4plex		3.25	55	2	576.85
									FVETHPEFIGIK	1	N-Term(iTRAQ4plex); K12(iTRAQ4plex			39		
261878452	RGMA	repulsive guidance molecule A isoform 3	11.11	0	3	7		49.3								
									VYQAEMDELPAAFVDGSK	2	N-Term(iTRAQ4plex); K18(iTRAQ4plex		4.84	84	2	1129.56
									GCPLNQQIDFQAFHTNAEGTGAR	3	N-Term(iTRAQ4plex); C2(Methylthio		4.18	73	3	889.09
									YIGTTIVVR	2	N-Term(iTRAQ4plex		3.66	51	2	583.36
223029470	NELL2	protein kinase C-binding protein NELL2 isoform a	11.09	0	9	91		96.8								
									ASTATAEQFFQK	25	N-Term(iTRAQ4plex); K12(iTRAQ4plex		6.11	104	2	808.93
									IMELQDILAK	5	N-Term(iTRAQ4plex); K10(iTRAQ4plex		4.91	69	2	731.44
									TCLDEMNVVR	2	N-Term(iTRAQ4plex); C2(Methylthio		4.2	31	2	685.32
									SALAYVDGK	2	N-Term(iTRAQ4plex); K9(iTRAQ4plex		3.94	51	2	606.35
									VVEKPSTDLPLGTTFWLGQR	5	N-Term(iTRAQ4plex); K4(iTRAQ4plex		3.92	61	3	844.81
									AFLFQDTPR	31	N-Term(iTRAQ4plex		3.78	43	2	619.83
									FTGSSWIK	16	N-Term(iTRAQ4plex); K8(iTRAQ4plex		3.64	66	2	607.35
									GYDFCSER	4	N-Term(iTRAQ4plex); C5(Methylthio		3.34	33	2	583.74
									HEFTILVTLK	1	N-Term(iTRAQ4plex); K10(iTRAQ4plex			75		
310124825	LOC100507705	PREDICTED: class I histocompatibility antigen, Gogo-B*0103 alpha chain-like isoform 8	11.08	0	3	5		38.9								
									SWTAADTAAQITQR	2	N-Term(iTRAQ4plex		4.98	72	2	832.43
									APWVEQEGPEYWDR	2	N-Term(iTRAQ4plex		3.42	63	2	953.45
									DYIALNEDLR	2	N-Term(iTRAQ4plex		3.38	41	2	683.36
24497519	MAN1A1	mannosyl-oligosaccharide 1,2-alpha-mannosidase IA	11.03	5	5	18	0.923	72.9								
									EEGAPGDPEAALEDNLAR	3	N-Term(iTRAQ4plex	1.01	4.98	95	2	999.48
									FDGGVEAIATR	5	N-Term(iTRAQ4plex	0.885	4.49	66	2	640.34
									GLPPVDFVPPIGVESR	3	N-Term(iTRAQ4plex	0.954	4.3	47	2	912.02
									SSSGLTYIAEWK	3	N-Term(iTRAQ4plex); K12(iTRAQ4plex	0.872	3.92	86	2	815.44
									MYFDAVQAIETHLIR	4	N-Term(iTRAQ4plex	1.044	3.83	53	3	651.01
20070125	P4HB	protein disulfide-isomerase precursor	11.02	5	5	7	0.891	57.1								
									VDATEESDLAQQYGVR	2	N-Term(iTRAQ4plex	0.932	5.52	103	2	962.97
									YKPESEELTAER	2	N-Term(iTRAQ4plex); K2(iTRAQ4plex	0.827	3.69	57	3	580.64
									LITLEEEMTK	1	N-Term(iTRAQ4plex); K10(iTRAQ4plex	0.978		79		
									ENLLDFIK	1	N-Term(iTRAQ4plex); K8(iTRAQ4plex	0.868		41		
									EADDIVNWLK	1	N-Term(iTRAQ4plex); K10(iTRAQ4plex	0.891		36		
29826289	CASC4	protein CASC4 isoform a	11.01	0	4	8		49.5								
									ELQMDPADYGK	2	N-Term(iTRAQ4plex); K11(iTRAQ4plex		4.88	70	2	777.89
									GGDAGMPGIEENDLAK	2	N-Term(iTRAQ4plex); M6(Oxidation); K16(iTRAQ4plex)		4.4	59	2	939.46
									LIPGSNLDSEPR	2	N-Term(iTRAQ4plex		3.59	55	2	721.39
									VDDLPPALR	2	N-Term(iTRAQ4plex			50		
118600983	NCAN	neurocan core protein precursor	10.98	11	11	35	0.803	143								
									KGTVLCGPPPAVENASLIGAR	2	N-Term(iTRAQ4plex); K1(iTRAQ4plex); C6(Methylthio)	0.736	5.99	83	3	795.44
									ELGGEVFYVGPAR	2	N-Term(iTRAQ4plex	0.78	5.91	84	2	769.41
									YQCNEGFAQHHVATIR	9	N-Term(iTRAQ4plex); C3(Methylthio	0.842	5.46	108	4	516.75
									GIEDEQDLVPLEVTGVVFHYR	4	N-Term(iTRAQ4plex	0.979	4.28	63	3	853.78
									LSSAIIAAPR	5	N-Term(iTRAQ4plex	0.803	4.16	71	2	571.85
									YALTFAEAQEACR	2	N-Term(iTRAQ4plex); C12(Methylthio	0.824	4.04	46	2	831.89
									APVLELEK	4	N-Term(iTRAQ4plex); K8(iTRAQ4plex	1.124	3.74	51	2	593.87
									GTVLCGPPPAVENASLIGAR	4	N-Term(iTRAQ4plex); C5(Methylthio	0.635	3.4	62	2	1056.56
									HLQAAFEDGFDNCDAGWLSDR	1	N-Term(iTRAQ4plex); C13(Methylthio	0.439		50		
									RNPQELYDVYCFAR	1	N-Term(iTRAQ4plex); C11(Methylthio	0.835		49		
									VSLPSYPR	1	N-Term(iTRAQ4plex	0.802		38		
13787217	FAT2	protocadherin Fat 2 precursor	10.97	36	36	80	1.046	479								
									LDQANHAPHTLTVH	2	N-Term(iTRAQ4plex); K14(iTRAQ4plex	1.177	5.87	55	3	611.68
									VVVGILDVNDNPPIFSHK	2	N-Term(iTRAQ4plex); K18(iTRAQ4plex	1.134	5.69	91	3	751.10
									AQDGGPPHWNSLVPVR	4	N-Term(iTRAQ4plex	0.889	5.68	58	3	625.33
									TGVLTVTGPLDYESK	2	N-Term(iTRAQ4plex); K15(iTRAQ4plex	1.154	5.43	106	2	934.52
									VSIEDVNDNPPK	2	N-Term(iTRAQ4plex); K12(iTRAQ4plex	1.058	5.42	69	2	807.93
									ASDGLYQDTALVK	2	N-Term(iTRAQ4plex); K13(iTRAQ4plex	1.005	5.29	38	2	834.95
									AVAAQDPVIYSLVR	2	N-Term(iTRAQ4plex	1.029	5.23	83	2	823.47
									SNEFSLVSVK	2	N-Term(iTRAQ4plex); K10(iTRAQ4plex	1.173	5.03	77	2	699.40
									VTPDGWLVTAEGLSR	2	N-Term(iTRAQ4plex	1.196	4.99	76	2	872.97
									ASDEDSEVNYSIK	2	N-Term(iTRAQ4plex); K13(iTRAQ4plex	1.057	4.96	71	2	872.93
									LDPHTGELTTLTALDR	2	N-Term(iTRAQ4plex	1.104	4.92	76	3	633.01
									VQLSEFSPPGSR	4	N-Term(iTRAQ4plex	1.04	4.7	46	2	724.39
									VTISEDMPLK	2	N-Term(iTRAQ4plex); K10(iTRAQ4plex	1.191	4.36	52	2	710.90
									DGVFSLDPDTGVIK	2	N-Term(iTRAQ4plex); K14(iTRAQ4plex	1.078	4.16	70	2	875.98
									YVLMDGAHGTFR	2	N-Term(iTRAQ4plex	1.048	4.1	47	2	755.88
									VDLMTGALILER	2	N-Term(iTRAQ4plex	0.93	4.08	60	2	737.93
									SEMFAIHPTSGVVTVAGH	2	N-Term(iTRAQ4plex); K18(iTRAQ4plex	0.919	4.03	40	3	707.05
									SGSGPYFYSQIR	2	N-Term(iTRAQ4plex	1.062	3.82	80	2	753.38
									VHEDVFPGHFILK	5	N-Term(iTRAQ4plex); K13(iTRAQ4plex	1.031	3.81	52	3	609.34
									YTLLSPTEK	4	N-Term(iTRAQ4plex); K9(iTRAQ4plex	0.976	3.69	46	2	670.40
									ISLTQVLDK	4	N-Term(iTRAQ4plex); K9(iTRAQ4plex	0.801	3.68	60	2	652.90
									SCQADITLHVEDVNDNAPF	2	N-Term(iTRAQ4plex); C2(Methylthio	1.145	3.58	46	3	763.03
									VIDLLAIDK	2	N-Term(iTRAQ4plex); K9(iTRAQ4plex	1.078	3.54	56	2	644.41
									ILEPEALK	6	N-Term(iTRAQ4plex); K8(iTRAQ4plex	0.927	3.53	54	2	600.87
									DVSYQIVEDGSDVSK	2	N-Term(iTRAQ4plex); K15(iTRAQ4plex	0.806	3.47	66	2	964.99
									LAATDPDAGFNGK	2	N-Term(iTRAQ4plex); K13(iTRAQ4plex	1.047	3.27	112	2	782.91
									LLTVNVK	2	N-Term(iTRAQ4plex); K7(iTRAQ4plex	1.016	3.22	50	2	537.85
									DVFNLVAK	2	N-Term(iTRAQ4plex); K8(iTRAQ4plex	1.099	3.17	46	2	597.36
									IHATDRDPQDTLTYSLAEEETLGR	1	N-Term(iTRAQ4plex	1.011		74		
									IDPYLGDISLK	1	N-Term(iTRAQ4plex); K11(iTRAQ4plex	1.168		71		
									VSATDLDTDTNAQITYSLHGPGAHEF	1	N-Term(iTRAQ4plex); K27(iTRAQ4plex	1.167		52		
									VAFCTVK	2	N-Term(iTRAQ4plex); C4(Methylthio); K7(iTRAQ4plex)	1.026		48		
									LDLGSGPSQHTLTVMVR	1	N-Term(iTRAQ4plex	1.191		43		
									EQQDTHELAVEVR	1	N-Term(iTRAQ4plex	1.222		41		
7661948	MLEC	malectin precursor	10.96	3	3	8	0.924	32.2								
									LSVQGEVSTFTGK	2	N-Term(iTRAQ4plex); K13(iTRAQ4plex	0.962	5.13	79	2	820.96
									FAEVYFAQSQQK	2	N-Term(iTRAQ4plex); K12(iTRAQ4plex	1.031	4.94	63	2	867.46
									LYIEFVK	4	N-Term(iTRAQ4plex); K7(iTRAQ4plex	0.863	3.49	53	2	600.36

261878463	RGMA	repulsive guidance molecule A isoform 1	10.92	0	3	7		50								
									VYQAEMDELPAAFVDGSK	2	N-Term(iTRAQ4plex); K18(iTRAQ4plex		4.84	84	2	1129.56
									GCPLNQQIDFQAFHTNAEGTGAR	3	N-Term(iTRAQ4plex); C2(Methylthio		4.18	73	3	889.09
									YIGTTIVVR	2	N-Term(iTRAQ4plex		3.66	51	2	583.36
56788387	MOG	myelin-oligodendrocyte glycoprotein isoform beta3 precursor	10.92	0	2	6		25.8								
									ALVGDEVELPCR	4	N-Term(iTRAQ4plex); C11(Methylthio		5.04	49	2	745.88
									DHSYQEEAAMELK	2	N-Term(iTRAQ4plex); K13(iTRAQ4plex		4.53	49	3	613.63
115298657	S100A7	protein S100-A7	10.89	1	1	2	0.783	11.5								
									GTNYLADVFEK	2	N-Term(iTRAQ4plex); K11(iTRAQ4plex	0.783	3.33	45	2	772.92
4506765	S100A4	protein S100-A4	10.89	1	1	1	1.011	11.7								
									ALDVMVSTFHK	1	N-Term(iTRAQ4plex); K11(iTRAQ4plex	1.011		46		
310124833	LOC100507705	PREDICTED: class I histocompatibility antigen, Gogo-B*0103 alpha chain-like isoform 7	10.86	0	3	5		39.4								
									SWTAADTAAQITQR	2	N-Term(iTRAQ4plex		4.98	72	2	832.43
									APWVEQEGPEYWDR	2	N-Term(iTRAQ4plex		3.42	63	2	953.45
									DYIALNEDLR	2	N-Term(iTRAQ4plex		3.38	41	2	683.36
4506115	PROC	vitamin K-dependent protein C preproprotein	10.85	4	4	7	1.73	52								
									RGDSPWQVVLLDSK	2	N-Term(iTRAQ4plex); K14(iTRAQ4plex	1.843	4.47	61	3	630.02
									ELNQAGQETLVTGWGYHSSR	2	N-Term(iTRAQ4plex	0.928	4.17	72	3	793.06
									WELDLDIK	2	N-Term(iTRAQ4plex); K8(iTRAQ4plex	1.623	3.48	50	2	660.37
									TFVLNFIK	1	N-Term(iTRAQ4plex); K8(iTRAQ4plex	2.278		48		
307611943	C16orf89	hypothetical protein LOC146556 isoform 2 precursor	10.8	0	3	9		40.6								
									ATIADLILSALER	5	N-Term(iTRAQ4plex		4.87	59	2	765.46
									WAQEPLLQPLSLR	2	N-Term(iTRAQ4plex		4.68	51	2	847.99
									LPEINLDGMVGVR	2	N-Term(iTRAQ4plex			39		
104487002	PTPRD	receptor-type tyrosine-protein phosphatase delta isoform 5 precursor	10.79	0	11	24		169.2								
									SDTIANYELVYK	4	N-Term(iTRAQ4plex); K12(iTRAQ4plex		5.58	74	2	852.46
									SPQGLGASTAEISAR	4	N-Term(iTRAQ4plex		5.34	96	2	794.93
									YSVAGLSPYSDYEFR	2	N-Term(iTRAQ4plex		5.01	65	2	949.46
									GFPTIDMGPQLK	2	N-Term(iTRAQ4plex); K12(iTRAQ4plex		4.89	87	2	796.44
									GALQIEQSEESDQGK	2	N-Term(iTRAQ4plex); K15(iTRAQ4plex		4.87	85	2	953.98
									YECVATNSAGTR	2	N-Term(iTRAQ4plex); C3(Methylthio		3.82	58	2	731.34
									WMLGAEDLTPEDDMPIGR	2	N-Term(iTRAQ4plex		3.43	45	2	1095.52
									FIKPWESPDEMELDELLK	2	N-Term(iTRAQ4plex); K3(iTRAQ4plex); K18(iTRAQ4plex)		3.43	65	3	884.47
									FEVIEFDDGSGSVLR	2	N-Term(iTRAQ4plex		3.19	41	2	907.46
									GPPSEPVLTQTSEQAPSSAPR	1	N-Term(iTRAQ4plex			44		
133925809	ITIH3	inter-alpha-trypsin inhibitor heavy chain H3 preproprotein	10.79	7	7	25	1.09	99.8								
									EHLVQATPENLQEAR	11	N-Term(iTRAQ4plex	1.214	6.07	71	3	627.00
									STSIVIMLTDGDANVGESRPEK	2	N-Term(iTRAQ4plex); K22(iTRAQ4plex	1.014	5.51	80	3	869.79
									LVDEDMNSFK	6	N-Term(iTRAQ4plex); K10(iTRAQ4plex	1.105	4.73	69	2	743.38
									EVSFDVELPK	2	N-Term(iTRAQ4plex); K10(iTRAQ4plex	1.161	4.46	42	2	725.91
									FTVSVNVAAGSK	2	N-Term(iTRAQ4plex); K12(iTRAQ4plex	1.074	3.41	91	2	734.43
4557469	AP2B1	AP-2 complex subunit beta isoform b	10.78	0	9	19		104.5								
									MEPLNNLQVAVK	2	N-Term(iTRAQ4plex); K12(iTRAQ4plex		5.46	78	2	822.48
									NVEGQDMLYQSLK	2	N-Term(iTRAQ4plex); K13(iTRAQ4plex		5.37	85	2	906.98
									DIPNENELQFQIK	2	N-Term(iTRAQ4plex); K13(iTRAQ4plex		5.29	66	2	938.51
									LQNNNVYTIAK	2	N-Term(iTRAQ4plex); K11(iTRAQ4plex		4.69	64	2	783.45
									IQPGNPNYTLSLK	2	N-Term(iTRAQ4plex); K13(iTRAQ4plex		4.31	67	2	867.00
									QVFLATWK	2	N-Term(iTRAQ4plex); K8(iTRAQ4plex		4.07	47	2	640.89
									GLEISGTFTHR	4	N-Term(iTRAQ4plex		3.73	53	3	454.58
									AVWLPAVK	2	N-Term(iTRAQ4plex); K8(iTRAQ4plex		3.46	38	2	586.38
									ECHLNADTVSSK	1	N-Term(iTRAQ4plex); C2(Methylthio); K12(iTRAQ4plex)			51		
62420888	DPP7	dipeptidyl peptidase 2 preproprotein	10.77	5	5	11	0.809	54.3								
									DVTADFEGQSPK	2	N-Term(iTRAQ4plex); K12(iTRAQ4plex	0.856	5.54	63	2	791.40
									GALLVFAEHR	5	N-Term(iTRAQ4plex	0.83	4.04	53	2	628.87
									SLPFGAQSTQR	2	N-Term(iTRAQ4plex	0.751	3.14	41	2	668.36
									DLFLQGAYDTVR	1	N-Term(iTRAQ4plex	0.743		39		
									LDHFNFER	1	N-Term(iTRAQ4plex	0.928		35		
283484020	PTPRD	receptor-type tyrosine-protein phosphatase delta isoform 3 precursor	10.76	0	11	24		169.6								
									SDTIANYELVYK	4	N-Term(iTRAQ4plex); K12(iTRAQ4plex		5.58	74	2	852.46
									SPQGLGASTAEISAR	4	N-Term(iTRAQ4plex		5.34	96	2	794.93
									YSVAGLSPYSDYEFR	2	N-Term(iTRAQ4plex		5.01	65	2	949.46
									GFPTIDMGPQLK	2	N-Term(iTRAQ4plex); K12(iTRAQ4plex		4.89	87	2	796.44
									GALQIEQSEESDQGK	2	N-Term(iTRAQ4plex); K15(iTRAQ4plex		4.87	85	2	953.98
									YECVATNSAGTR	2	N-Term(iTRAQ4plex); C3(Methylthio		3.82	58	2	731.34
									WMLGAEDLTPEDDMPIGR	2	N-Term(iTRAQ4plex		3.43	45	2	1095.52
									FIKPWESPDEMELDELLK	2	N-Term(iTRAQ4plex); K3(iTRAQ4plex); K18(iTRAQ4plex)		3.43	65	3	884.47
									FEVIEFDDGSGSVLR	2	N-Term(iTRAQ4plex		3.19	41	2	907.46
									GPPSEPVLTQTSEQAPSSAPR	1	N-Term(iTRAQ4plex			44		
289547551	PTPRD	receptor-type tyrosine-protein phosphatase delta isoform 2 precursor	10.76	0	11	24		169.5								
									SDTIANYELVYK	4	N-Term(iTRAQ4plex); K12(iTRAQ4plex		5.58	74	2	852.46
									SPQGLGASTAEISAR	4	N-Term(iTRAQ4plex		5.34	96	2	794.93
									YSVAGLSPYSDYEFR	2	N-Term(iTRAQ4plex		5.01	65	2	949.46
									GFPTIDMGPQLK	2	N-Term(iTRAQ4plex); K12(iTRAQ4plex		4.89	87	2	796.44
									GALQIEQSEESDQGK	2	N-Term(iTRAQ4plex); K15(iTRAQ4plex		4.87	85	2	953.98
									YECVATNSAGTR	2	N-Term(iTRAQ4plex); C3(Methylthio		3.82	58	2	731.34
									WMLGAEDLTPEDDMPIGR	2	N-Term(iTRAQ4plex		3.43	45	2	1095.52
									FIKPWESPDEMELDELLK	2	N-Term(iTRAQ4plex); K3(iTRAQ4plex); K18(iTRAQ4plex)		3.43	65	3	884.47
									FEVIEFDDGSGSVLR	2	N-Term(iTRAQ4plex		3.19	41	2	907.46
									GPPSEPVLTQTSEQAPSSAPR	1	N-Term(iTRAQ4plex			44		
10864029	JAM2	junctional adhesion molecule B precursor	10.74	3	3	6	0.909	33.2								
									TGTLQFNTVSK	2	N-Term(iTRAQ4plex); K11(iTRAQ4plex	0.903	4.98	51	2	742.42
									AEMIDFNIR	2	N-Term(iTRAQ4plex	0.935	3.69	44	2	626.83
									EGNPAPEYTWFK	2	N-Term(iTRAQ4plex); K12(iTRAQ4plex	0.909	3.23	47	2	863.94
73486658	GOT2	aspartate aminotransferase, mitochondrial precursor	10.7	4	4	7	0.608	47.5								
									VGAFTMVCK	2	N-Term(iTRAQ4plex); C8(Methylthio); K9(iTRAQ4plex)	0.544	4.13	64	2	645.34

									KQWLQEVK	2	N-Term(iTRAQ4plex); K1(iTRAQ4plex); K8(iTRAQ4plex)	0.726	3.51	53	3	497.64
									FVTVQTISGTGALF	2	N-Term(iTRAQ4plex	0.626	3.36	62	2	797.46
									ASAELALGENSEVLK	1	N-Term(iTRAQ4plex); K15(iTRAQ4plex	0.577		52		
110347423	SPON1	spondin-1 precursor	10.66	7	7	15	0.977	90.9								
									QQSDEVLTVIK	2	N-Term(iTRAQ4plex); K11(iTRAQ4plex	1.096	5.09	62	2	774.45
									VEGDPDFYKPGTSYR	2	N-Term(iTRAQ4plex); K9(iTRAQ4plex	0.939	4.83	52	3	673.67
									VTLSAAPPSYFR	2	N-Term(iTRAQ4plex	0.957	4.46	47	2	726.90
									AAPSAEFSVDR	4	N-Term(iTRAQ4plex	1.011	4.4	61	2	647.33
									SEQLKEESEGEQFPGCR	2	N-Term(iTRAQ4plex); K5(iTRAQ4plex); C16(Methylthio)	1.185	3.99	60	3	763.02
									GFTLIALR	2	N-Term(iTRAQ4plex	0.947	3.39	47	2	517.83
									AQWPAWQPLNVR	1	N-Term(iTRAQ4plex	0.998		57		
71773106	AP2B1	AP-2 complex subunit beta isoform a	10.62	0	9	19		105.6								
									MEPLNNLQVAVK	2	N-Term(iTRAQ4plex); K12(iTRAQ4plex		5.46	78	2	822.48
									NVEGQDMLYQSLK	2	N-Term(iTRAQ4plex); K13(iTRAQ4plex		5.37	85	2	906.98
									DIPNENELQFQIK	2	N-Term(iTRAQ4plex); K13(iTRAQ4plex		5.29	66	2	938.51
									LQNNNVYTIAK	2	N-Term(iTRAQ4plex); K11(iTRAQ4plex		4.69	64	2	783.45
									IQPGNPNYTLSLK	2	N-Term(iTRAQ4plex); K13(iTRAQ4plex		4.31	67	2	867.00
									QVFLATWK	2	N-Term(iTRAQ4plex); K8(iTRAQ4plex		4.07	47	2	640.89
									GLEISGTFTHR	4	N-Term(iTRAQ4plex		3.73	53	3	454.58
									AVWLPAVK	2	N-Term(iTRAQ4plex); K8(iTRAQ4plex		3.46	38	2	586.38
									ECHLNADTVSSK	1	N-Term(iTRAQ4plex); C2(Methylthio); K12(iTRAQ4plex)			51		
310119066	LOC100506055	PREDICTED: hypothetical protein LOC100506055	10.59	0	1	2		17.5								
									GPSSEGPEEEDGEGFSFK	2	N-Term(iTRAQ4plex); K18(iTRAQ4plex		3.24	67	2	1086.99
56549129	MXRA7	matrix-remodeling-associated protein 7 isoform 3	10.59	0	1	2		17.5								
									GPSSEGPEEEDGEGFSFK	2	N-Term(iTRAQ4plex); K18(iTRAQ4plex		3.24	67	2	1086.99
194097467	PLEKHB1	pleckstrin homology domain-containing family B member 1 isoform c	10.58	0	2	6		23.2								
									IGPECHDVQPPEGR	5	N-Term(iTRAQ4plex); C5(Methylthio		5.14	45	3	575.27
									VLIHFNVR	1	N-Term(iTRAQ4plex			36		
188528648	TNXB	tenascin-X isoform 1 precursor	10.58	14	19	43	0.795	457.9								
									VPGHEDGVTISGLEPDHK	5	N-Term(iTRAQ4plex); K18(iTRAQ4plex	1.035	7.37	63	4	544.54
									VGGEESEVTVGGLEPGR	5	N-Term(iTRAQ4plex	0.731	6.97	103	2	908.47
									GEESEVTVGGLEPGR	2	N-Term(iTRAQ4plex	0.748	6.04	95	2	830.42
									VPEGPGAHEEVLPGDVR	2	N-Term(iTRAQ4plex	0.735	5.5	68	3	634.66
									LGPLSAEGTTGLAPAGQTSEESRPR	2	N-Term(iTRAQ4plex		5.49	59	3	876.12
									LGVLTVTDTTPDSMR	4	N-Term(iTRAQ4plex	0.963	5.43	74	2	875.46
									FDSFTVQYK	2	N-Term(iTRAQ4plex); K9(iTRAQ4plex	0.889	4.31	66	2	711.88
									FLLYGLHEGK	2	N-Term(iTRAQ4plex); K10(iTRAQ4plex		4.01	53	2	732.93
									EVSVPGLDPAHR	3	N-Term(iTRAQ4plex	0.946	3.8	57	3	474.26
									YGPLTADGTTAPER	2	N-Term(iTRAQ4plex	0.72	3.74	55	2	796.91
									VGPISAVAITAGR	2	N-Term(iTRAQ4plex	0.698	3.5	83	2	678.41
									ILISGLEPSTPYR	2	N-Term(iTRAQ4plex		3.48	51	2	795.45
									MNLYGFHGGQR	4	N-Term(iTRAQ4plex	0.793	3.29	50	2	712.35
									GFEFSVPFTEMK	1	N-Term(iTRAQ4plex); K12(iTRAQ4plex			55		
									FLLFGIQDGK	1	N-Term(iTRAQ4plex); K10(iTRAQ4plex	0.688		51		
									AVAVSGLDPAR	1	N-Term(iTRAQ4plex	0.971		49		
									NDITLSGLESDHR	1	N-Term(iTRAQ4plex	0.967		42		
									FLLYGLLGGK	1	N-Term(iTRAQ4plex); K10(iTRAQ4plex	0.928		35		
5729772	CLN5	ceroid-lipofuscinosis neuronal protein 5	10.57	5	5	13	1.064	46.3								
									LAEFGAEFK	4	N-Term(iTRAQ4plex); K9(iTRAQ4plex	1.189	4.13	60	2	650.36
									YGDLLGHLK	2	N-Term(iTRAQ4plex); K9(iTRAQ4plex	1.064	3.94	43	2	652.39
									LQAPVWEFK	4	N-Term(iTRAQ4plex); K9(iTRAQ4plex	1.102	3.76	64	2	703.41
									IMHDAIGFR	2	N-Term(iTRAQ4plex	0.969	3.56	40	2	602.32
									HWPVPYK	1	N-Term(iTRAQ4plex); K7(iTRAQ4plex	1.004		49		
116284404	CNTNAP4	contactin-associated protein-like 4 isoform 1	10.55	0	12	34		145.2								
									NMADGQLHHIMINR	8	N-Term(iTRAQ4plex		6.74	72	4	449.23
									YQEPDVVNFDFK	2	N-Term(iTRAQ4plex); K12(iTRAQ4plex		5.58	67	2	894.96
									DGAGGWSPLVSNK	2	N-Term(iTRAQ4plex); K13(iTRAQ4plex		5.01	43	2	788.42
									TMQSDGILLHR	2	N-Term(iTRAQ4plex		4.94	56	3	472.26
									EYLSVIIAK	2	N-Term(iTRAQ4plex); K9(iTRAQ4plex		4.19	47	2	662.41
									AQVTPEVQPGCR	2	N-Term(iTRAQ4plex); C11(Methylthio		4.18	52	2	737.87
									NMADGQLHHIMINR	3	N-Term(iTRAQ4plex); M11(Oxidation		4.04	52	3	603.97
									YQWLQIDLGER	2	N-Term(iTRAQ4plex		4.04	44	2	782.91
									QDGTPLSWWVGR	2	N-Term(iTRAQ4plex		3.58	55	2	773.40
									SPLGGFQGCMR	2	N-Term(iTRAQ4plex); C9(Methylthio		3.42	50	2	671.81
									LFLLINSGEAK	1	N-Term(iTRAQ4plex); K11(iTRAQ4plex			80		
									FIPLEWNPK	5	N-Term(iTRAQ4plex); K9(iTRAQ4plex			58		
									EGPNGDHITLQLR	1	N-Term(iTRAQ4plex			37		
269914185	C4orf48	hypothetical protein LOC401115 isoform 2 precursor	10.53	0	1	6		10.2								
									TETLLLQAER	6	N-Term(iTRAQ4plex		4.3	53	2	659.38
315570273	ACTG2	actin, gamma-enteric smooth muscle isoform 2 precursor	10.51	0	3	12		37.1								
									EITALAPSTMK	3	N-Term(iTRAQ4plex); K11(iTRAQ4plex		4.19	66	2	725.42
									DLTDYLMK	5	N-Term(iTRAQ4plex); K8(iTRAQ4plex		3.98	48	2	643.85
									SYELPDGQVITIGNER	3	N-Term(iTRAQ4plex		3.27	49	2	968.00
									EITALAPSTMK	1	N-Term(iTRAQ4plex); M10(Oxidation); K11(iTRAQ4plex)			43		
296080704	TCN2	transcobalamin-2 isoform 2 precursor	10.5	0	3	5		44.4								
									DPNTPLLQGIADYRPK	2	N-Term(iTRAQ4plex); K16(iTRAQ4plex		7.56	42	3	696.05
									LGQHLLPWMDR	2	N-Term(iTRAQ4plex		3.84	43	2	755.41
									LSLEHLNPSIYVGLR	1	N-Term(iTRAQ4plex			36		
310124845	LOC100507705	PREDICTED: class I histocompatibility antigen, Gogo-B*0103 alpha chain-like isoform 6, partial	10.47	0	3	5		40.7								
									SWTAADTAAQITQR	2	N-Term(iTRAQ4plex		4.98	72	2	832.43
									APWVEQEGPEYWDR	2	N-Term(iTRAQ4plex		3.42	63	2	953.45
									DYIALNEDLR	2	N-Term(iTRAQ4plex		3.38	41	2	683.36
310124827	LOC100507705	PREDICTED: class I histocompatibility antigen, Gogo-B*0103 alpha chain-like isoform 1	10.38	0	3	5		40.9								
									SWTAADTAAQITQR	2	N-Term(iTRAQ4plex		4.98	72	2	832.43
									APWVEQEGPEYWDR	2	N-Term(iTRAQ4plex		3.42	63	2	953.45
									DYIALNEDLR	2	N-Term(iTRAQ4plex		3.38	41	2	683.36
4826673	CDH6	cadherin-6 preproprotein	10.38	7	7	12	0.86	88.3								

									IVVEDVDEPPVFSK	2	N-Term(iTRAQ4plex); K14(iTRAQ4plex	0.874	6.15	86	2	931.02
									TGRPVEPESEFIIK	2	N-Term(iTRAQ4plex); K14(iTRAQ4plex	0.816	5.77	37	3	630.69
									VEASNPYVEPR	2	N-Term(iTRAQ4plex	0.846	4.16	57	2	702.87
									IHDINDNEPIFTK	2	N-Term(iTRAQ4plex); K13(iTRAQ4plex	0.906	3.64	76	2	922.49
									TPESSPPGTPIGR	1	N-Term(iTRAQ4plex	0.886		41		
									FLYLGPFK	1	N-Term(iTRAQ4plex); K8(iTRAQ4plex	0.939		40		
52630342	HLA-C	HLA class I histocompatibility antigen, Cw-1 alpha chain precursor	10.38	0	3	5		40.6								
									SWTAADTAAQITQR	2	N-Term(iTRAQ4plex		4.98	72	2	832.43
									APWVEQEGPEYWDR	2	N-Term(iTRAQ4plex		3.42	63	2	953.45
									DYIALNEDLR	2	N-Term(iTRAQ4plex		3.38	41	2	683.36
5031839	KRT6A	keratin, type II cytoskeletal 6A	10.28	1	6	15	1.641	60								
									WTLLQEQGTK	4	N-Term(iTRAQ4plex); K10(iTRAQ4plex		4.27	67	2	746.42
									TAAENEFVTLK	2	N-Term(iTRAQ4plex); K11(iTRAQ4plex		3.68	44	2	755.92
									EYQELMNVK	2	N-Term(iTRAQ4plex); K9(iTRAQ4plex		3.39	60	2	721.38
									GMQDLVEDFK	2	N-Term(iTRAQ4plex); K10(iTRAQ4plex	1.641	3.38	48	2	735.38
									FASFIDK	4	N-Term(iTRAQ4plex); K7(iTRAQ4plex		3.19	53	2	558.31
119703753	KRT6B	keratin, type II cytoskeletal 6B	10.28	0	6	29		60								
									YEELQITAGR	16	N-Term(iTRAQ4plex		4.29	67	2	662.36
									WTLLQEQGTK	4	N-Term(iTRAQ4plex); K10(iTRAQ4plex		4.27	67	2	746.42
									TAAENEFVTLK	2	N-Term(iTRAQ4plex); K11(iTRAQ4plex		3.68	44	2	755.92
									EYQELMNVK	2	N-Term(iTRAQ4plex); K9(iTRAQ4plex		3.39	60	2	721.38
									FASFIDK	4	N-Term(iTRAQ4plex); K7(iTRAQ4plex		3.19	53	2	558.31
20665034	TNXB	tenascin-X isoform 2	10.25	0	5	8		74								
									LGPLSAEGTTGLAPAGQTSEESRPR	2	N-Term(iTRAQ4plex		5.49	59	3	876.12
									FLLYGLHEGK	2	N-Term(iTRAQ4plex); K10(iTRAQ4plex		4.01	53	2	732.93
									ILISGLEPSTPYR	2	N-Term(iTRAQ4plex		3.48	51	2	795.45
									GFEFSVPFTEMK	1	N-Term(iTRAQ4plex); K12(iTRAQ4plex			55		
310124829	LOC100507705	PREDICTED: class I histocompatibility antigen, Gogo-B*0103 alpha chain-like isoform 2	10.22	0	3	5		41.6								
									SWTAADTAAQITQR	2	N-Term(iTRAQ4plex		4.98	72	2	832.43
									APWVEQEGPEYWDR	2	N-Term(iTRAQ4plex		3.42	63	2	953.45
									DYIALNEDLR	2	N-Term(iTRAQ4plex		3.38	41	2	683.36
310124831	LOC100507705	PREDICTED: class I histocompatibility antigen, Gogo-B*0103 alpha chain-like isoform 3	10.22	0	3	5		41.6								
									SWTAADTAAQITQR	2	N-Term(iTRAQ4plex		4.98	72	2	832.43
									APWVEQEGPEYWDR	2	N-Term(iTRAQ4plex		3.42	63	2	953.45
									DYIALNEDLR	2	N-Term(iTRAQ4plex		3.38	41	2	683.36
61744483	SLC3A2	4F2 cell-surface antigen heavy chain isoform f	10.21	0	5	9		57.9								
									GQSEDPGSLLSLFR	2	N-Term(iTRAQ4plex		3.83	67	2	825.44
									EDFDSLLQSAK	2	N-Term(iTRAQ4plex); K11(iTRAQ4plex		3.65	82	2	770.91
									LDYLSSLK	3	N-Term(iTRAQ4plex); K8(iTRAQ4plex		3.27	51	2	613.87
									LLTSFLPAQLLR	1	N-Term(iTRAQ4plex			44		
									GLVLGPIHK	1	N-Term(iTRAQ4plex); K9(iTRAQ4plex			32		
10863955	PSAT1	phosphoserine aminotransferase isoform 2	10.19	0	3	6		35.2								
									QVVNFGPGPAK	2	N-Term(iTRAQ4plex); K11(iTRAQ4plex		4.49	55	2	701.41
									IINNTENLVR	2	N-Term(iTRAQ4plex		3.47	52	2	665.39
									GVGISVLEMSHR	2	N-Term(iTRAQ4plex			58		
56549133	MXRA7	matrix-remodeling-associated protein 7 isoform 2	10.17	0	1	2		18.2								
									GPSSEGPEEEDGEGFSFK	2	N-Term(iTRAQ4plex); K18(iTRAQ4plex		3.24	67	2	1086.99
13194201	RTN4R	reticulon-4 receptor precursor	10.15	4	4	15	0.951	50.7								
									SVDPATFHGLGR	7	N-Term(iTRAQ4plex	0.875	5.4	72	3	467.59
									CGLQELGPGLFR	2	N-Term(iTRAQ4plex); C1(Methylthio	0.93	4.86	49	2	740.38
									GSSSEVPCSLPQR	2	N-Term(iTRAQ4plex); C8(Methylthio	1.029	4.16	46	2	768.87
									ATDEEPLGLPK	4	N-Term(iTRAQ4plex); K11(iTRAQ4plex	0.978	4	67	2	729.41
24850115	SIAE	sialate O-acetylesterase precursor	10.13	4	4	10	1.062	58.3								
									FASYINNDMVLQK	2	N-Term(iTRAQ4plex); K13(iTRAQ4plex	1.107	6.3	69	2	915.99
									QGSIPYDSVTGPSK	4	N-Term(iTRAQ4plex); K14(iTRAQ4plex	0.977	5.28	88	2	862.46
									WHQTADFGYVPNPK	3	N-Term(iTRAQ4plex); K14(iTRAQ4plex	1.066	4.26	66	3	650.00
									ELSNTAAYQSVR	1	N-Term(iTRAQ4plex	1.019		44		
38372931	BCAN	brevican core protein isoform 2	10.13	0	6	35		71.6								
									CEVQHGIDDSSDAVEVK	13	N-Term(iTRAQ4plex); C1(Methylthio); K17(iTRAQ4plex)		8.3	84	3	722.34
									EACYGDMDGFPGVR	2	N-Term(iTRAQ4plex); C3(Methylthio		5.17	77	2	853.86
									YAFSFSGAQEACAR	2	N-Term(iTRAQ4plex); C12(Methylthio		3.7	58	2	849.38
									GVVFLYR	15	N-Term(iTRAQ4plex		3.28	42	2	499.30
									LTLEEAR	1	N-Term(iTRAQ4plex			37		
									EACYGDMDGFPGVR	1	N-Term(iTRAQ4plex); C3(Methylthio); M7(Oxidation)			25		
56788381	MOG	myelin-oligodendrocyte glycoprotein isoform alpha1 precursor	10.12	0	2	6		28.2								
									ALVGDEVELPCR	4	N-Term(iTRAQ4plex); C11(Methylthio		5.04	49	2	745.88
									DHSYQEEAAMELK	2	N-Term(iTRAQ4plex); K13(iTRAQ4plex		4.53	49	3	613.63
27436938	RELN	reelin isoform a	10.12	0	27	91		388.1								
									TVMLDTFSSAPVPQHEF	2	N-Term(iTRAQ4plex		6.08	54	3	687.02
									ITIQLPDHVSSSATQFF	3	N-Term(iTRAQ4plex		6.05	44	3	682.04
									FSYSDPSIIVLYAK	2	N-Term(iTRAQ4plex); K14(iTRAQ4plex		5.12	63	2	946.03
									EHITLDTLSYSSYK	5	N-Term(iTRAQ4plex); K14(iTRAQ4plex		4.93	71	3	649.01
									QLITSFLDSSQSR	4	N-Term(iTRAQ4plex		4.76	74	2	813.44
									APDQPGEGVLLHYSYDNGITWK	2	N-Term(iTRAQ4plex); K22(iTRAQ4plex		4.68	79	3	916.80
									GENVQFQWK	10	N-Term(iTRAQ4plex); K9(iTRAQ4plex		4.47	52	2	712.38
									LCTPSMDTTGYGNLR	6	N-Term(iTRAQ4plex); C2(Methylthio		4.41	61	2	909.92
									FVYLELPAAAK	2	N-Term(iTRAQ4plex); K11(iTRAQ4plex		4.21	52	2	755.45
									ILVSDTFNK	2	N-Term(iTRAQ4plex); K9(iTRAQ4plex		4.16	52	2	662.89
									IISVELPGDAK	8	N-Term(iTRAQ4plex); K11(iTRAQ4plex		3.91	69	2	715.43
									ITYPLPESLVGNPVR	5	N-Term(iTRAQ4plex		3.82	63	2	900.02
									KLCTPSMDTTGYGNLR	2	N-Term(iTRAQ4plex); K1(iTRAQ4plex); C3(Methylthio)		3.55	46	3	697.68
									ITIPLPNAALTR	5	N-Term(iTRAQ4plex		3.54	48	2	712.44
									ITLPLPPYTR	2	N-Term(iTRAQ4plex		3.38	37	2	657.90
									ITGAQVGTGCGTLNDGK	2	N-Term(iTRAQ4plex); C10(Methylthio); K17(iTRAQ4plex)		3.32	68	2	963.48
									VSYNVPLEAR	3	N-Term(iTRAQ4plex		3.27	41	2	646.36
									VIVLLPQK	14	N-Term(iTRAQ4plex); K8(iTRAQ4plex		3.26	46	2	599.41
									YIALEIPLK	2	N-Term(iTRAQ4plex); K9(iTRAQ4plex		3.22	44	2	674.43
									GAEVSFGCGVLASGK	2	N-Term(iTRAQ4plex); C8(Methylthio); K15(iTRAQ4plex)		3.16	33	2	858.43

									ITVYLPLSTISPR	1	N-Term(iTRAQ4plex			56		
									LSPLWYK	1	N-Term(iTRAQ4plex); K7(iTRAQ4plex			53		
									LSSYHNFYSIR	1	N-Term(iTRAQ4plex			51		
									EVYAVTHDLTPTEGWIMQFK	1	N-Term(iTRAQ4plex); K20(iTRAQ4plex			48		
									HDGLDQNDWAIDNVLISGSADQF	1	N-Term(iTRAQ4plex			39		
									QAVTQDLDLR	1	N-Term(iTRAQ4plex			38		
									TSVNEHWLFHDDCTVEF	1	N-Term(iTRAQ4plex); C13(Methylthio			32		
									LCTPSMDTTGYGNLR	1	N-Term(iTRAQ4plex); C2(Methylthio); M6(Oxidation)			30		
27436940	RELN	reelin isoform b	10.12	0	27	91		388								
									TVMLDTFSSAPVPQHEF	2	N-Term(iTRAQ4plex		6.08	54	3	687.02
									ITIQLPDHVSSSATQFF	3	N-Term(iTRAQ4plex		6.05	44	3	682.04
									FSYSDPSIIVLYAK	2	N-Term(iTRAQ4plex); K14(iTRAQ4plex		5.12	63	2	946.03
									EHITLDTLSYSSYK	5	N-Term(iTRAQ4plex); K14(iTRAQ4plex		4.93	71	3	649.01
									QLITSFLDSSQSR	4	N-Term(iTRAQ4plex		4.76	74	2	813.44
									APDQPGEGVLLHYSYDNGITWK	2	N-Term(iTRAQ4plex); K22(iTRAQ4plex		4.68	79	3	916.80
									GENVQFQWK	10	N-Term(iTRAQ4plex); K9(iTRAQ4plex		4.47	52	2	712.38
									LCTPSMDTTGYGNLR	6	N-Term(iTRAQ4plex); C2(Methylthio		4.41	61	2	909.92
									FVYLELPAAAK	2	N-Term(iTRAQ4plex); K11(iTRAQ4plex		4.21	52	2	755.45
									ILVSDTFNK	2	N-Term(iTRAQ4plex); K9(iTRAQ4plex		4.16	52	2	662.89
									IISVELPGDAK	8	N-Term(iTRAQ4plex); K11(iTRAQ4plex		3.91	69	2	715.43
									ITYPLPESLVGNPVR	5	N-Term(iTRAQ4plex		3.82	63	2	900.02
									KLCTPSMDTTGYGNLR	2	N-Term(iTRAQ4plex); K1(iTRAQ4plex); C3(Methylthio)		3.55	46	3	697.68
									ITIPLPNAALTR	5	N-Term(iTRAQ4plex		3.54	48	2	712.44
									ITLPLPPYTR	2	N-Term(iTRAQ4plex		3.38	37	2	657.90
									ITGAQVGTGCGTLNDGK	2	N-Term(iTRAQ4plex); C10(Methylthio); K17(iTRAQ4plex)		3.32	68	2	963.48
									VSYNVPLEAR	3	N-Term(iTRAQ4plex		3.27	41	2	646.36
									VIVLLPQK	14	N-Term(iTRAQ4plex); K8(iTRAQ4plex		3.26	46	2	599.41
									YIALEIPLK	2	N-Term(iTRAQ4plex); K9(iTRAQ4plex		3.22	44	2	674.43
									GAEVSFGCGVLASGK	2	N-Term(iTRAQ4plex); C8(Methylthio); K15(iTRAQ4plex)		3.16	33	2	858.43
									ITVYLPLSTISPR	1	N-Term(iTRAQ4plex			56		
									LSPLWYK	1	N-Term(iTRAQ4plex); K7(iTRAQ4plex			53		
									LSSYHNFYSIR	1	N-Term(iTRAQ4plex			51		
									EVYAVTHDLTPTEGWIMQFK	1	N-Term(iTRAQ4plex); K20(iTRAQ4plex			48		
									HDGLDQNDWAIDNVLISGSADQF	1	N-Term(iTRAQ4plex			39		
									QAVTQDLDLR	1	N-Term(iTRAQ4plex			38		
									TSVNEHWLFHDDCTVEF	1	N-Term(iTRAQ4plex); C13(Methylthio			32		
									LCTPSMDTTGYGNLR	1	N-Term(iTRAQ4plex); C2(Methylthio); M6(Oxidation)			30		
71040111	FMOD	fibromodulin precursor	10.11	3	3	3	0.8	43.2								
									SLILLDLSYNHLR	1	N-Term(iTRAQ4plex	0.69		47		
									IPPVNTNLENLYLQGNF	1	N-Term(iTRAQ4plex	0.849		43		
									YLPFVPSR	1	N-Term(iTRAQ4plex	0.8		36		
256217721	CPN2	carboxypeptidase N subunit 2	10.09	5	5	8	1.064	60.5								
									LSNNALSGLPQGVFGK	2	N-Term(iTRAQ4plex); K16(iTRAQ4plex	1.06	3.74	55	2	945.54
									LFQPLTHLK	2	N-Term(iTRAQ4plex); K9(iTRAQ4plex	1.17	3.5	32	3	462.29
									LTVSIEAR	2	N-Term(iTRAQ4plex	0.657	3.4	44	2	516.81
									DHLGFQVTWPDESK	1	N-Term(iTRAQ4plex); K14(iTRAQ4plex	1.348		54		
									LELLSLSK	1	N-Term(iTRAQ4plex); K8(iTRAQ4plex	1.064		51		
9845238	B3GNT2	UDP-GlcNAc:betaGal beta-1,3-N-acetylglucosaminyltransferase 2	10.08	3	3	6	0.894	46								
									VTSVVTGFNNLPDF	3	N-Term(iTRAQ4plex	0.894	4.89	59	2	831.95
									ISTPPEAYWNR	2	N-Term(iTRAQ4plex	0.833	3.58	39	2	739.38
									DLFIGDVIHNAGPHF	1	N-Term(iTRAQ4plex	1.001		66		
62243290	IGFBP4	insulin-like growth factor-binding protein 4 precursor	10.08	3	3	5	1.031	27.9								
									GELDCHQLADSFR	2	N-Term(iTRAQ4plex); C5(Methylthio	1.031	4.92	75	2	840.88
									GELDCHQLADSFRE	2	N-Term(iTRAQ4plex); C5(Methylthio	1.264	3.51	34	3	603.94
									CRPPVGCEELVR	1	N-Term(iTRAQ4plex); C1(Methylthio); C7(Methylthio)	0.825		46		
42560237	PVRL1	poliovirus receptor-related protein 1 isoform 1 precursor	10.06	0	4	13		57.1								
									VEFLRPSFTDGTIR	5	N-Term(iTRAQ4plex		4.04	43	3	594.66
									LKGEAEYQEIR	4	N-Term(iTRAQ4plex); K2(iTRAQ4plex		3.78	57	2	812.45
									QNVAIYNPSMGVSVLAPYF	2	N-Term(iTRAQ4plex		3.23	40	2	1112.09
									ITQVTWQK	2	N-Term(iTRAQ4plex); K8(iTRAQ4plex			56		
14589889	CDH2	cadherin-2 preproprotein	10.04	9	9	57	0.956	99.7								
									DVHEGQPLLNVK	19	N-Term(iTRAQ4plex); K12(iTRAQ4plex	0.956	5.58	82	2	818.97
									LSLKPTLTEESVK	10	N-Term(iTRAQ4plex); K4(iTRAQ4plex); K13(iTRAQ4plex)	0.966	5.56	98	3	626.38
									ESAEVEEIVFPR	9	N-Term(iTRAQ4plex	0.79	5.54	69	2	774.91
									VQYESSEPADFK	2	N-Term(iTRAQ4plex); K12(iTRAQ4plex	0.879	5.06	61	2	844.43
									TGFPEDVYSAVLSK	9	N-Term(iTRAQ4plex); K14(iTRAQ4plex	0.957	3.93	72	2	900.99
									FLIYAQDK	5	N-Term(iTRAQ4plex); K8(iTRAQ4plex	1.029	3.24	51	2	643.37
									KVQYESSEPADFK	1	N-Term(iTRAQ4plex); K1(iTRAQ4plex); K13(iTRAQ4plex)	1.069		52		
									DWVIPPINLPENSR	1	N-Term(iTRAQ4plex	0.944		51		
4507875	VCAM1	vascular cell adhesion protein 1 isoform a precursor	10.01	0	7	17		81.2								
									LHIDEMDSVPTVR	2	N-Term(iTRAQ4plex		4.69	73	2	828.43
									GIQVEIYSFPK	2	N-Term(iTRAQ4plex); K11(iTRAQ4plex		4.68	61	2	784.95
									SLEMTFIPTIEDTGK	2	N-Term(iTRAQ4plex); K15(iTRAQ4plex		4.1	51	2	985.52
									LHIDDMEFEPK	2	N-Term(iTRAQ4plex); K11(iTRAQ4plex		3.65	73	2	831.43
									SQEFLEDADR	2	N-Term(iTRAQ4plex		3.64	38	2	677.32
									LEIELLK	4	N-Term(iTRAQ4plex); K7(iTRAQ4plex		3.49	54	2	573.37
									LEIDLLK	3	N-Term(iTRAQ4plex); K7(iTRAQ4plex		3.28	39	2	566.36
297374785	ARHGDIA	rho GDP-dissociation inhibitor 1 isoform b	10	0	1	2		18.2								
									SIQEIQELDKDDESLR	2	N-Term(iTRAQ4plex); K10(iTRAQ4plex		3.64	61	3	736.05
16751921	DCD	dermcidin preproprotein	10	1	1	2	1.374	11.3								
									DAVEDLESVGK	2	N-Term(iTRAQ4plex); K11(iTRAQ4plex	1.374	5.04	68	2	725.38
4502149	APOA2	apolipoprotein A-II preproprotein	10	2	2	20	0.843	11.2								
									SKEQLTPLIK	3	N-Term(iTRAQ4plex); K2(iTRAQ4plex); K10(iTRAQ4plex)	0.828	4.2	43	2	795.01
									EQLTPLIK	17	N-Term(iTRAQ4plex); K8(iTRAQ4plex	0.846	3.96	40	2	615.39

62243068	IGFBP3	insulin-like growth factor-binding protein 3 isoform b precursor	9.97	0	3	5		31.7								
									EMEDTLNHLK	2	N-Term(iTRAQ4plex); K10(iTRAQ4plex		4.02	39	3	506.60
									YGQPLPGYTTK	2	N-Term(iTRAQ4plex); K11(iTRAQ4plex		3.2	39	2	756.92
									FLNVLSPR	1	N-Term(iTRAQ4plex			37		
15431310	KRT14	keratin, type I cytoskeletal 14	9.96	1	5	13	2.007	51.6								
									ASLENSLEETK	4	N-Term(iTRAQ4plex); K11(iTRAQ4plex		5.06	82	2	754.90
									LEQEIATYR	2	N-Term(iTRAQ4plex		4.13	44	2	633.84
									DAEEWFFTK	2	N-Term(iTRAQ4plex); K9(iTRAQ4plex	2.007	3.5	56	2	730.87
									LASYLDK	4	N-Term(iTRAQ4plex); K7(iTRAQ4plex		3.35	53	2	549.32
									ISSVLAGGSCR	1	N-Term(iTRAQ4plex); C10(Methylthio			36		
4758484	GSTO1	glutathione S-transferase omega-1 isoform 1	9.96	0	2	3		27.5								
									GSAPPGPVPEGSIR	2	N-Term(iTRAQ4plex			46		
									LNECVDHTPK	1	N-Term(iTRAQ4plex); C4(Methylthio); K10(iTRAQ4plex)			35		
56788385	MOG	myelin-oligodendrocyte glycoprotein isoform beta1 precursor	9.92	0	2	6		28.6								
									ALVGDEVELPCR	4	N-Term(iTRAQ4plex); C11(Methylthio		5.04	49	2	745.88
									DHSYQEEAAMELK	2	N-Term(iTRAQ4plex); K13(iTRAQ4plex		4.53	49	3	613.63
6042196	CTSF	cathepsin F precursor	9.92	5	5	7	0.861	53.3								
									FSDLTEEEFR	2	N-Term(iTRAQ4plex	0.855	3.9	40	2	708.84
									SVGDLAPPEWDWR	1	N-Term(iTRAQ4plex	0.866		63		
									LAAWLAK	2	N-Term(iTRAQ4plex); K7(iTRAQ4plex	0.825		48		
									NSWGTDWGEK	1	N-Term(iTRAQ4plex); K10(iTRAQ4plex	1.149		43		
									FALEMFNR	1	N-Term(iTRAQ4plex	0.973		40		
4505991	PPIC	peptidyl-prolyl cis-trans isomerase C	9.91	2	2	6	0.982	22.7								
									TVENFVALATGEK	2	N-Term(iTRAQ4plex); K13(iTRAQ4plex	0.878	3.65	73	2	833.97
									IVIGLFGK	4	N-Term(iTRAQ4plex); K8(iTRAQ4plex	1.038	3.45	55	2	567.87
283484022	PTPRD	receptor-type tyrosine-protein phosphatase delta isoform 4 precursor	9.83	0	10	22		168.6								
									SDTIANYELVYK	4	N-Term(iTRAQ4plex); K12(iTRAQ4plex		5.58	74	2	852.46
									SPQGLGASTAEISAR	4	N-Term(iTRAQ4plex		5.34	96	2	794.93
									YSVAGLSPYSDYEFR	2	N-Term(iTRAQ4plex		5.01	65	2	949.46
									GFPTIDMGPQLK	2	N-Term(iTRAQ4plex); K12(iTRAQ4plex		4.89	87	2	796.44
									YECVATNSAGTR	2	N-Term(iTRAQ4plex); C3(Methylthio		3.82	58	2	731.34
									WMLGAEDLTPEDDMPIGR	2	N-Term(iTRAQ4plex		3.43	45	2	1095.52
									FIKPWESPDEMELDELLK	2	N-Term(iTRAQ4plex); K3(iTRAQ4plex); K18(iTRAQ4plex)		3.43	65	3	884.47
									FEVIEFDDGSGSVLR	2	N-Term(iTRAQ4plex		3.19	41	2	907.46
									GPPSEPVLTQTSEQAPSSAPR	1	N-Term(iTRAQ4plex			44		
194097469	PLEKHB1	pleckstrin homology domain-containing family B member 1 isoform b	9.82	0	2	6		25.2								
									IGPECHDVQPPEGR	5	N-Term(iTRAQ4plex); C5(Methylthio		5.14	45	3	575.27
									VLIHFNVR	1	N-Term(iTRAQ4plex			36		
283484024	PTPRD	receptor-type tyrosine-protein phosphatase delta isoform 6 precursor	9.77	0	10	22		169.4								
									SDTIANYELVYK	4	N-Term(iTRAQ4plex); K12(iTRAQ4plex		5.58	74	2	852.46
									SPQGLGASTAEISAR	4	N-Term(iTRAQ4plex		5.34	96	2	794.93
									YSVAGLSPYSDYEFR	2	N-Term(iTRAQ4plex		5.01	65	2	949.46
									GFPTIDMGPQLK	2	N-Term(iTRAQ4plex); K12(iTRAQ4plex		4.89	87	2	796.44
									YECVATNSAGTR	2	N-Term(iTRAQ4plex); C3(Methylthio		3.82	58	2	731.34
									WMLGAEDLTPEDDMPIGR	2	N-Term(iTRAQ4plex		3.43	45	2	1095.52
									FIKPWESPDEMELDELLK	2	N-Term(iTRAQ4plex); K3(iTRAQ4plex); K18(iTRAQ4plex)		3.43	65	3	884.47
									FEVIEFDDGSGSVLR	2	N-Term(iTRAQ4plex		3.19	41	2	907.46
									GPPSEPVLTQTSEQAPSSAPR	1	N-Term(iTRAQ4plex			44		
38372935	BCAN	brevican core protein isoform 1	9.77	1	7	37	1.072	99.1								
									CEVQHGIDDSSDAVEVK	13	N-Term(iTRAQ4plex); C1(Methylthio); K17(iTRAQ4plex)		8.3	84	3	722.34
									EACYGDMDGFPGVR	2	N-Term(iTRAQ4plex); C3(Methylthio		5.17	77	2	853.86
									MYGAHLASISTPEEQDFINNR	2	N-Term(iTRAQ4plex	1.072	5.16	50	3	846.41
									YAFSFSGAQEACAR	2	N-Term(iTRAQ4plex); C12(Methylthio		3.7	58	2	849.38
									GVVFLYR	15	N-Term(iTRAQ4plex		3.28	42	2	499.30
									LTLEEAR	1	N-Term(iTRAQ4plex			37		
									EACYGDMDGFPGVR	1	N-Term(iTRAQ4plex); C3(Methylthio); M7(Oxidation)			25		
62243248	IGFBP3	insulin-like growth factor-binding protein 3 isoform a precursor	9.76	0	3	5		32.2								
									EMEDTLNHLK	2	N-Term(iTRAQ4plex); K10(iTRAQ4plex		4.02	39	3	506.60
									YGQPLPGYTTK	2	N-Term(iTRAQ4plex); K11(iTRAQ4plex		3.2	39	2	756.92
									FLNVLSPR	1	N-Term(iTRAQ4plex			37		
294997284	DAG1	dystroglycan preproprotein	9.72	6	6	42	0.953	97.5								
									EGAMSAQLGYPVVGWHIANK	11	N-Term(iTRAQ4plex); K20(iTRAQ4plex	0.955	8.42	76	3	806.10
									LGCSLNQNSVPDIHGVEAPAR	5	N-Term(iTRAQ4plex); C3(Methylthio	0.991	6.98	78	3	789.72
									SFSEVELHNMK	15	N-Term(iTRAQ4plex); K11(iTRAQ4plex	0.942	5.83	79	2	804.92
									EGAMSAQLGYPVVGWHIANK	2	N-Term(iTRAQ4plex); M4(Oxidation); K20(iTRAQ4plex)	0.98	5.41	77	3	811.43
									VVENGALLSWK	2	N-Term(iTRAQ4plex); K11(iTRAQ4plex	1.202	5.35	65	2	752.44
									VTIPTDLIASSGDIIK	6	N-Term(iTRAQ4plex); K16(iTRAQ4plex	0.83	4.16	65	2	966.07
									LAFAFGDR	1	N-Term(iTRAQ4plex	0.834		37		
307611942	C16orf89	hypothetical protein LOC146556 isoform 1 precursor	9.7	0	3	9		45.4								
									ATIADLILSALER	5	N-Term(iTRAQ4plex		4.87	59	2	765.46
									WAQEPLLQPLSLR	2	N-Term(iTRAQ4plex		4.68	51	2	847.99
									LPEINLDGMVGVR	2	N-Term(iTRAQ4plex			39		
17975597	SELM	selenoprotein M precursor	9.66	1	1	2	0.803	16.2								
									HLPGADPELVLLGR	2	N-Term(iTRAQ4plex	0.803	3.6	50	3	544.32
24475586	EPDR1	mammalian ependymin-related protein 1 precursor	9.59	3	3	4	0.643	38.1								
									DGVMFQIDQATK	2	N-Term(iTRAQ4plex); K12(iTRAQ4plex	0.713	3.54	73	2	820.93
									FFDIQLGIK	1	N-Term(iTRAQ4plex); K9(iTRAQ4plex	0.621		39		
									SYETWIGIYTVK	1	N-Term(iTRAQ4plex); K12(iTRAQ4plex	0.643		39		
22538442	CTSZ	cathepsin Z preproprotein	9.57	2	2	9	1.055	33.8								
									NVDGVNYASITR	7	N-Term(iTRAQ4plex	1.055	5.1	65	2	726.88
310124823	LOC100507705	PREDICTED: class I histocompatibility antigen, Gogo-B*0103 alpha chain-like isoform 4	9.57	0	3	5		44.6								
									SWTAADTAAQITQR	2	N-Term(iTRAQ4plex		4.98	72	2	832.43
									APWVEQEGPEYWDR	2	N-Term(iTRAQ4plex		3.42	63	2	953.45

									DYIALNEDLR	2	N-Term(iTRAQ4plex		3.38	41	2	683.36
4507511	TIMP2	metalloproteinase inhibitor 2 precursor	9.55	3	3	5	1.068	24.4								
									EVDSGNDIYGNPIK	2	N-Term(iTRAQ4plex); K14(iTRAQ4plex	1.31	5.45	94	2	904.97
									EVDSGNDIYGNPIKR	2	N-Term(iTRAQ4plex); K14(iTRAQ4plex	1.054	4.06	83	2	983.02
									IQYEIK	1	N-Term(iTRAQ4plex); K6(iTRAQ4plex	1.068		37		
28212222	WFIKKN2	WAP, kazal, immunoglobulin, kunitz and NTR domain-containing protein 2	9.55	7	7	21	0.839	63.9								
									ALVTVDEVLKDEK	2	N-Term(iTRAQ4plex); K10(iTRAQ4plex); K13(iTRAQ4plex)	0.839	5.46	78	2	946.05
									ALVTVDEVLK	4	N-Term(iTRAQ4plex); K10(iTRAQ4plex	0.776	4.56	76	2	687.93
									VSELTEEPDSGR	2	N-Term(iTRAQ4plex	0.771	4.41	68	2	731.86
									ADFPLSVVR	4	N-Term(iTRAQ4plex	0.968	3.41	51	2	574.33
									SDFVILGR	7	N-Term(iTRAQ4plex	0.774		51		
									EFLGLH	1	N-Term(iTRAQ4plex	0.877		39		
									LVTSFCR	1	N-Term(iTRAQ4plex); C6(Methylthio	0.844		35		
42544213	NDRG2	protein NDRG2 isoform b	9.52	0	3	6		39.3								
									LDPTQTSFLK	2	N-Term(iTRAQ4plex); K10(iTRAQ4plex		3.9	80	2	719.42
									GWMDWAAHK	3	N-Term(iTRAQ4plex); K9(iTRAQ4plex		3.44	38	3	463.91
									MADSGGQPQLTQPGK	1	N-Term(iTRAQ4plex); K15(iTRAQ4plex			35		
48255891	PRKCSH	glucosidase 2 subunit beta isoform 2	9.52	0	4	10		59.1								
									LGGSPTSLGTWGSWIGPDHDK	2	N-Term(iTRAQ4plex); K21(iTRAQ4plex		5.52	87	3	819.42
									ESLQQMAEVTR	2	N-Term(iTRAQ4plex		5.15	60	2	718.37
									LWEEQLAAAK	4	N-Term(iTRAQ4plex); K10(iTRAQ4plex		4.01	73	2	723.91
									LIELQAGK	2	N-Term(iTRAQ4plex); K8(iTRAQ4plex		3.67	39	2	580.37
61744481	SLC3A2	4F2 cell-surface antigen heavy chain isoform e	9.51	0	5	9		61.8								
									GQSEDPGSLLSLFR	2	N-Term(iTRAQ4plex		3.83	67	2	825.44
									EDFDSLLQSAK	2	N-Term(iTRAQ4plex); K11(iTRAQ4plex		3.65	82	2	770.91
									LDYLSSLK	3	N-Term(iTRAQ4plex); K8(iTRAQ4plex		3.27	51	2	613.87
									LLTSFLPAQLLR	1	N-Term(iTRAQ4plex			44		
									GLVLGPIHK	1	N-Term(iTRAQ4plex); K9(iTRAQ4plex			32		
208609951	NRXN1	neurexin-1-beta isoform alpha2 precursor	9.5	0	12	55		169.8								
									LTVDDQQAMTGQMAGDHTR	6	N-Term(iTRAQ4plex		7.91	76	3	740.35
									GPETLFAGYNLNDNEWHTVR	6	N-Term(iTRAQ4plex		6.71	63	3	826.41
									LEFHNIETGIITER	8	N-Term(iTRAQ4plex		5.82	65	3	606.00
									GKEEYIATFK	3	N-Term(iTRAQ4plex); K2(iTRAQ4plex); K10(iTRAQ4plex)		5.26	46	3	539.97
									IQLPVVMHTEAEDVSLR	4	N-Term(iTRAQ4plex		4.37	54	3	694.38
									VVDEWLLDK	4	N-Term(iTRAQ4plex); K9(iTRAQ4plex		4.3	64	2	702.91
									NIIADPVTFK	10	N-Term(iTRAQ4plex); K10(iTRAQ4plex		4.03	60	2	703.42
									TGSISFDFR	2	N-Term(iTRAQ4plex		3.86	41	2	587.30
									AGGREPYPGSAEVIR	2	N-Term(iTRAQ4plex		3.67	36	2	851.96
									FNDNAWHDVK	2	N-Term(iTRAQ4plex); K10(iTRAQ4plex		3.57	54	3	511.93
									EPYPGSAEVIR	4	N-Term(iTRAQ4plex		3.52	36	2	681.36
									LTVDDQQAMTGQMAGDHTR	2	N-Term(iTRAQ4plex); M9(Oxidation		3.46	66	3	745.68
									EATVLSYDGSMFMK	2	N-Term(iTRAQ4plex); K14(iTRAQ4plex		3.28	69	2	933.97
22538440	CTSC	dipeptidyl peptidase 1 isoform b precursor	9.49	0	2	2		15.2								
									WFAFFK	1	N-Term(iTRAQ4plex); K6(iTRAQ4plex			40		
									VVVYLQK	1	N-Term(iTRAQ4plex); K7(iTRAQ4plex			39		
29337283	LYNX1	ly-6/neurotoxin-like protein 1 isoform c	9.48	1	1	16	0.99	12.6								
									CFETVYDGYSK	16	N-Term(iTRAQ4plex); C1(Methylthio); K11(iTRAQ4plex)	0.99	4.89	77	2	823.37
48255889	PRKCSH	glucosidase 2 subunit beta isoform 1	9.47	0	4	10		59.4								
									LGGSPTSLGTWGSWIGPDHDK	2	N-Term(iTRAQ4plex); K21(iTRAQ4plex		5.52	87	3	819.42
									ESLQQMAEVTR	2	N-Term(iTRAQ4plex		5.15	60	2	718.37
									LWEEQLAAAK	4	N-Term(iTRAQ4plex); K10(iTRAQ4plex		4.01	73	2	723.91
									LIELQAGK	2	N-Term(iTRAQ4plex); K8(iTRAQ4plex		3.67	39	2	580.37
10835143	CD55	complement decay-accelerating factor isoform 1 preproprotein	9.45	0	4	22		41.4								
									TSFPEDTVITYK	13	N-Term(iTRAQ4plex); K12(iTRAQ4plex		4.74	92	2	844.96
									LTCLQNLK	5	N-Term(iTRAQ4plex); C3(Methylthio); K8(iTRAQ4plex)		4.13	64	2	633.86
									WSTAVEFCK	2	N-Term(iTRAQ4plex); C8(Methylthio); K9(iTRAQ4plex)		3.91	48	2	702.85
									DSVICLK	2	N-Term(iTRAQ4plex); C5(Methylthio); K7(iTRAQ4plex)			41		
55956790	NTRK2	BDNF/NT-3 growth factors receptor isoform b precursor	9.43	0	3	5		53								
									SSPDTQDLYCLNESSK	2	N-Term(iTRAQ4plex); C10(Methylthio); K16(iTRAQ4plex)		3.28	67	2	1060.99
									RLEIINEDDVEAYVGLR	2	N-Term(iTRAQ4plex			59		
									SNEIPSTDVTDK	1	N-Term(iTRAQ4plex); K12(iTRAQ4plex			57		
166235138	SEZ6L2	seizure 6-like protein 2 isoform 3	9.4	0	6	32		90.8								
									TASDAGFPVGSHVQYR	13	N-Term(iTRAQ4plex		5.93	112	2	918.46
									EGDMLTLFDGDGPSAR	8	N-Term(iTRAQ4plex		5.06	86	2	912.93
									SGGSPLSPVIYDSDMDDVPER	4	N-Term(iTRAQ4plex		3.49	86	2	1190.56
									LLLHFQSPR	4	N-Term(iTRAQ4plex		3.48	44	2	627.87
									WVIEAAEGR	2	N-Term(iTRAQ4plex		3.39	38	2	587.82
									FEAFEEDR	1	N-Term(iTRAQ4plex			39		
258613951	ASPH	aspartyl/asparaginyl beta-hydroxylase isoform i	9.38	0	2	3		28.9								
									LGIYDADGDGDFDVDDAK	2	N-Term(iTRAQ4plex); K18(iTRAQ4plex		6.31	74	2	1095.01
									VLLGLK	1	N-Term(iTRAQ4plex); K6(iTRAQ4plex			32		
14149613	NRXN1	neurexin-1-beta isoform alpha1 precursor	9.34	0	11	51		161.8								
									LTVDDQQAMTGQMAGDHTR	6	N-Term(iTRAQ4plex		7.91	76	3	740.35
									GPETLFAGYNLNDNEWHTVR	6	N-Term(iTRAQ4plex		6.71	63	3	826.41
									LEFHNIETGIITER	8	N-Term(iTRAQ4plex		5.82	65	3	606.00
									GKEEYIATFK	3	N-Term(iTRAQ4plex); K2(iTRAQ4plex); K10(iTRAQ4plex)		5.26	46	3	539.97
									IQLPVVMHTEAEDVSLR	4	N-Term(iTRAQ4plex		4.37	54	3	694.38
									NIIADPVTFK	10	N-Term(iTRAQ4plex); K10(iTRAQ4plex		4.03	60	2	703.42
									TGSISFDFR	2	N-Term(iTRAQ4plex		3.86	41	2	587.30
									AGGREPYPGSAEVIR	2	N-Term(iTRAQ4plex		3.67	36	2	851.96
									FNDNAWHDVK	2	N-Term(iTRAQ4plex); K10(iTRAQ4plex		3.57	54	3	511.93
									EPYPGSAEVIR	4	N-Term(iTRAQ4plex		3.52	36	2	681.36
									LTVDDQQAMTGQMAGDHTR	2	N-Term(iTRAQ4plex); M9(Oxidation		3.46	66	3	745.68
									EATVLSYDGSMFMK	2	N-Term(iTRAQ4plex); K14(iTRAQ4plex		3.28	69	2	933.97
4506549	RNASE2	non-secretory ribonuclease precursor	9.32	1	1	3	1.097	18.3								
									RDPPQYPVVPVHLDR	3	N-Term(iTRAQ4plex	1.097	6.54	35	4	483.77

315434271	VCAM1	vascular cell adhesion protein 1 isoform c precursor	9.31	0	6	15		74.3								
									LHIDEMDSVPTVR	2	N-Term(iTRAQ4plex		4.69	73	2	828.43
									SLEMTFIPTIEDTGK	2	N-Term(iTRAQ4plex); K15(iTRAQ4plex		4.1	51	2	985.52
									LHIDDMEFEPK	2	N-Term(iTRAQ4plex); K11(iTRAQ4plex		3.65	73	2	831.43
									SQEFLEDADR	2	N-Term(iTRAQ4plex		3.64	38	2	677.32
									LEIELLK	4	N-Term(iTRAQ4plex); K7(iTRAQ4plex		3.49	54	2	573.37
									LEIDLLK	3	N-Term(iTRAQ4plex); K7(iTRAQ4plex		3.28	39	2	566.36
28195384	NPTX2	neuronal pentraxin-2 precursor	9.28	2	2	5	0.776	47								
									LESLEHQLR	3	N-Term(iTRAQ4plex	0.766	4.13	45	2	634.86
									LGTGENLAPWHPIKPGGVLILGQEQDTVGR	2	N-Term(iTRAQ4plex); K14(iTRAQ4plex	0.799	3.55	64	4	875.24
6912612	SEZ6L2	seizure 6-like protein 2 isoform 1	9.26	0	6	32		92.4								
									TASDAGFPVGSHVQYR	13	N-Term(iTRAQ4plex		5.93	112	2	918.46
									EGDMLTLFDGDGPSAR	8	N-Term(iTRAQ4plex		5.06	86	2	912.93
									SGGSPLSPVIYDSDMDDVPER	4	N-Term(iTRAQ4plex		3.49	86	2	1190.56
									LLLHFQSPR	4	N-Term(iTRAQ4plex		3.48	44	2	627.87
									WVIEAAEGR	2	N-Term(iTRAQ4plex		3.39	38	2	587.82
									FEAFEEDR	1	N-Term(iTRAQ4plex			39		
167000671	CTSC	dipeptidyl peptidase 1 isoform c precursor	9.22	0	2	2		15.7								
									WFAFFK	1	N-Term(iTRAQ4plex); K6(iTRAQ4plex			40		
									VVVYLQK	1	N-Term(iTRAQ4plex); K7(iTRAQ4plex			39		
42544211	NDRG2	protein NDRG2 isoform a	9.16	0	3	6		40.8								
									LDPTQTSFLK	2	N-Term(iTRAQ4plex); K10(iTRAQ4plex		3.9	80	2	719.42
									GWMDWAAHK	3	N-Term(iTRAQ4plex); K9(iTRAQ4plex		3.44	38	3	463.91
									MADSGGQPQLTQPGK	1	N-Term(iTRAQ4plex); K15(iTRAQ4plex			35		
9910270	OLFML3	olfactomedin-like protein 3 precursor	9.11	4	4	7	0.897	46								
									DFTLAMAAR	2	N-Term(iTRAQ4plex	0.875	3.61	55	2	570.30
									FGGPAGLWTK	2	N-Term(iTRAQ4plex); K10(iTRAQ4plex	0.956	3.59	74	2	661.39
									MLPLLEVAEK	2	N-Term(iTRAQ4plex); K10(iTRAQ4plex	0.919	3.17	60	2	715.93
4507065	SLPI	antileukoproteinase precursor	9.09	1	1	2	0.841	14.3								
									CLDPVDTPNPTR	2	N-Term(iTRAQ4plex); C1(Methylthio	0.841	4.1	41	2	759.37
259089453	GFRA2	GDNF family receptor alpha-2 isoform c	9.06	0	3	8		36.4								
									QTILPSCSYEDK	2	N-Term(iTRAQ4plex); C7(Methylthio); K12(iTRAQ4plex)		4.67	77	2	859.43
									DFTENPCLR	4	N-Term(iTRAQ4plex); C7(Methylthio		3.9	34	2	642.79
									SSYISICNR	2	N-Term(iTRAQ4plex); C7(Methylthio		3.42	47	2	616.80
10864013	PLEKHB1	pleckstrin homology domain-containing family B member 1 isoform a	9.05	0	2	6		27.2								
									IGPECHDVQPPEGR	5	N-Term(iTRAQ4plex); C5(Methylthio		5.14	45	3	575.27
									VLIHFNVR	1	N-Term(iTRAQ4plex			36		
4502599	CBR1	carbonyl reductase [NADPH] 1	9.03	1	2	4	0.618	30.4								
									FHQLDIDDLQSIR	2	N-Term(iTRAQ4plex		5.33	52	3	581.97
									EGWPSSAYGVTK	2	N-Term(iTRAQ4plex); K12(iTRAQ4plex	0.618	3.92	56	2	785.42
7662350	CNTNAP2	contactin-associated protein-like 2 precursor	9.02	10	10	21	0.859	148.1								
									VDNAPDQQNSHPDLAQEEIR	2	N-Term(iTRAQ4plex	0.921	6.13	83	3	807.39
									SPTPLNDDQWHR	2	N-Term(iTRAQ4plex	0.822	5.04	65	3	537.27
									KDAGFLSYK	2	N-Term(iTRAQ4plex); K1(iTRAQ4plex); K9(iTRAQ4plex)	0.952	4.46	43	3	487.62
									YSSSDWVTQYR	2	N-Term(iTRAQ4plex	0.886	4.42	59	2	768.37
									DHLPVSQVVVGDTDR	2	N-Term(iTRAQ4plex	0.912	4.22	74	2	890.97
									HYYWGGSGPGIQK	2	N-Term(iTRAQ4plex); K13(iTRAQ4plex	0.94	4.21	54	3	579.97
									DAGFLSYK	4	N-Term(iTRAQ4plex); K8(iTRAQ4plex	0.833	3.67	53	2	594.83
									MSQIDISSGSGLNDGQWHEVR	2	N-Term(iTRAQ4plex	0.615	3.5	42	3	820.73
									DVIALNFK	2	N-Term(iTRAQ4plex); K8(iTRAQ4plex	0.76	3.39	47	2	604.37
									IVPLDWNGEGR	1	N-Term(iTRAQ4plex	0.846		39		
21361905	JAM3	junctional adhesion molecule C precursor	9.01	2	2	3	1.134	39.6								
									DDSGQYYCIASNDAGSAR	2	N-Term(iTRAQ4plex); C8(Methylthio	1.012	3.5	74	2	1041.93
									IQDEQTTYVFFDNK	1	N-Term(iTRAQ4plex); K14(iTRAQ4plex	1.27		41		
119360348	FUCA1	tissue alpha-L-fucosidase precursor	9.01	4	4	14	0.807	53.7								
									ITMLGIQGDLK	4	N-Term(iTRAQ4plex); K11(iTRAQ4plex	0.845	4.91	71	2	738.94
									DLVGELGTALR	6	N-Term(iTRAQ4plex	0.795	4.11	65	2	644.37
									WEMCTSIDK	2	N-Term(iTRAQ4plex); C4(Methylthio); K9(iTRAQ4plex)	0.804	3.93	44	2	723.84
									DGLIVPIFQER	2	N-Term(iTRAQ4plex	0.874	3.57	54	2	715.91
221316760	L1CAM	neural cell adhesion molecule L1 isoform 3 precursor	8.97	0	10	31		138.8								
									VSWSPAEDHNAPIEK	6	N-Term(iTRAQ4plex); K15(iTRAQ4plex		6.6	50	3	656.67
									LVLSDLHLLTQSQVR	4	N-Term(iTRAQ4plex		4.9	89	2	933.55
									AQLLVVGSPGPVPR	7	N-Term(iTRAQ4plex		4.6	61	2	767.47
									CEASGKPEVQFR	3	N-Term(iTRAQ4plex); C1(Methylthio); K6(iTRAQ4plex)		4.55	56	2	842.93
									WRPVDLAQVK	3	N-Term(iTRAQ4plex); K10(iTRAQ4plex		4.48	46	3	500.64
									LVVFPTDDISLK	3	N-Term(iTRAQ4plex); K12(iTRAQ4plex		3.7	77	2	817.99
									INGIPVEELAK	1	N-Term(iTRAQ4plex); K11(iTRAQ4plex			54		
									LSPYVHYTFR	1	N-Term(iTRAQ4plex			38		
									WYSLGK	2	N-Term(iTRAQ4plex); K6(iTRAQ4plex			38		
10863949	ANGPTL7	angiopoietin-related protein 7 precursor	8.96	3	3	7	1	40								
									VEMEDWEGNLR	2	N-Term(iTRAQ4plex	1.037	4.4	43	2	761.36
									GDFWLGNEHIHR	3	N-Term(iTRAQ4plex	0.982	3.93	48	3	542.28
									SGLVSFYR	2	N-Term(iTRAQ4plex	1.01	3.36	48	2	536.80
13435353	L1CAM	neural cell adhesion molecule L1 isoform 2 precursor	8.94	0	10	31		139.4								
									VSWSPAEDHNAPIEK	6	N-Term(iTRAQ4plex); K15(iTRAQ4plex		6.6	50	3	656.67
									LVLSDLHLLTQSQVR	4	N-Term(iTRAQ4plex		4.9	89	2	933.55
									AQLLVVGSPGPVPR	7	N-Term(iTRAQ4plex		4.6	61	2	767.47
									CEASGKPEVQFR	3	N-Term(iTRAQ4plex); C1(Methylthio); K6(iTRAQ4plex)		4.55	56	2	842.93
									WRPVDLAQVK	3	N-Term(iTRAQ4plex); K10(iTRAQ4plex		4.48	46	3	500.64
									LVVFPTDDISLK	3	N-Term(iTRAQ4plex); K12(iTRAQ4plex		3.7	77	2	817.99
									INGIPVEELAK	1	N-Term(iTRAQ4plex); K11(iTRAQ4plex			54		
									LSPYVHYTFR	1	N-Term(iTRAQ4plex			38		
									WYSLGK	2	N-Term(iTRAQ4plex); K6(iTRAQ4plex			38		
17402893	PSAT1	phosphoserine aminotransferase isoform 1	8.92	0	3	6		40.4								
									QVVNFGPGPAK	2	N-Term(iTRAQ4plex); K11(iTRAQ4plex		4.49	55	2	701.41
									IINNTENLVR	2	N-Term(iTRAQ4plex		3.47	52	2	665.39
									GVGISVLEMSHR	2	N-Term(iTRAQ4plex			58		
32130518	APOC2	apolipoprotein C-II precursor	8.91	1	1	2	1.154	11.3								

									TYLPAVDEK	2	N-Term(iTRAQ4plex); K9(iTRAQ4plex	1.154	3.7	53	2	662.37
4557707	L1CAM	neural cell adhesion molecule L1 isoform 1 precursor	8.91	0	10	31		139.9								
									VSWSPAEDHNAPIEK	6	N-Term(iTRAQ4plex); K15(iTRAQ4plex		6.6	50	3	656.67
									LVLSDLHLLTQSQVR	4	N-Term(iTRAQ4plex		4.9	89	2	933.55
									AQLLVVGSPGPVPR	7	N-Term(iTRAQ4plex		4.6	61	2	767.47
									CEASGKPEVQFR	3	N-Term(iTRAQ4plex); C1(Methylthio); K6(iTRAQ4plex)		4.55	56	2	842.93
									WRPVDLAQVK	3	N-Term(iTRAQ4plex); K10(iTRAQ4plex		4.48	46	3	500.64
									LVVFPTDDISLK	3	N-Term(iTRAQ4plex); K12(iTRAQ4plex		3.7	77	2	817.99
									INGIPVEELAK	1	N-Term(iTRAQ4plex); K11(iTRAQ4plex			54		
									LSPYVHYTFR	1	N-Term(iTRAQ4plex			38		
									WYSLGK	2	N-Term(iTRAQ4plex); K6(iTRAQ4plex			38		
105990535	F5	coagulation factor V precursor	8.9	17	17	35	0.861	251.5								
									ADKPLSIHPQGIR	3	N-Term(iTRAQ4plex); K3(iTRAQ4plex	0.869	5.74	51	3	574.01
									VMYTQYEDESFTK	2	N-Term(iTRAQ4plex); K13(iTRAQ4plex	0.856	4.97	75	2	964.96
									SWYLEDNINK	2	N-Term(iTRAQ4plex); K10(iTRAQ4plex	0.845	4.83	49	2	785.41
									LSEGASYLDHTFPAEK	3	N-Term(iTRAQ4plex); K16(iTRAQ4plex	0.97	4.69	53	3	685.02
									SQHLDNFSNQIGK	2	N-Term(iTRAQ4plex); K13(iTRAQ4plex	0.772	4.34	60	3	592.64
									FCENPDEVK	2	N-Term(iTRAQ4plex); C2(Methylthio); K9(iTRAQ4plex)	0.855	4.11	63	2	707.84
									EDGILGPIIR	3	N-Term(iTRAQ4plex	0.832	3.95	48	2	613.86
									SSSPELSEMLEYDR	2	N-Term(iTRAQ4plex	0.991	3.68	64	2	893.92
									ASEFLGYWEPR	3	N-Term(iTRAQ4plex	0.814	3.62	47	2	749.88
									WIISSLTPK	2	N-Term(iTRAQ4plex); K9(iTRAQ4plex	0.785	3.56	43	2	666.91
									QHQLGVWPLLPGSFK	2	N-Term(iTRAQ4plex); K15(iTRAQ4plex	0.923	3.48	43	3	665.72
									TYEDDSPEWFK	2	N-Term(iTRAQ4plex); K11(iTRAQ4plex	0.836	3.4	68	2	852.90
									LAAALGIR	3	N-Term(iTRAQ4plex	1.053	3.26	40	2	464.81
									QWLEIDLLK	1	N-Term(iTRAQ4plex); K9(iTRAQ4plex	0.726		62		
									DIHSGLIGPLLICQK	1	N-Term(iTRAQ4plex); C13(Methylthio); K15(iTRAQ4plex)	1.015		49		
									EYEPYFK	1	N-Term(iTRAQ4plex); K7(iTRAQ4plex	0.811		43		
									GEYEEHLGILGPIIR	1	N-Term(iTRAQ4plex	0.897		35		
258613955	ASPH	aspartyl/asparaginyl beta-hydroxylase isoform k	8.89	0	2	3		29.7								
									LGIYDADGDGDFDVDDAK	2	N-Term(iTRAQ4plex); K18(iTRAQ4plex		6.31	74	2	1095.01
									VLLGLK	1	N-Term(iTRAQ4plex); K6(iTRAQ4plex			32		
4759164	SPOCK1	testican-1 precursor	8.88	4	4	8	1.205	49.1								
									DWFGALHEDANR	4	N-Term(iTRAQ4plex	1.08	4.58	51	3	525.59
									SLLGAFIPR	2	N-Term(iTRAQ4plex	1.233	3.7	47	2	559.35
									LEFHACSTGK	1	N-Term(iTRAQ4plex); C6(Methylthio); K10(iTRAQ4plex)	1.205		74		
									CNEEGYYK	1	N-Term(iTRAQ4plex); C1(Methylthio); K8(iTRAQ4plex)	1.792		35		
258613957	ASPH	aspartyl/asparaginyl beta-hydroxylase isoform l	8.87	0	1	2		21.9								
									LGIYDADGDGDFDVDDAK	2	N-Term(iTRAQ4plex); K18(iTRAQ4plex		6.31	74	2	1095.01
261878614	ITIH1	inter-alpha-trypsin inhibitor heavy chain H1 isoform b	8.84	0	5	38		86.1								
									GSLVQASEANLQAAQDFVR	5	N-Term(iTRAQ4plex		6.6	104	2	1074.56
									QYYEGSEIVVAGR	17	N-Term(iTRAQ4plex		5.65	78	2	807.92
									LDAQASFLPK	11	N-Term(iTRAQ4plex); K10(iTRAQ4plex		4.65	61	2	689.40
									NHMQYEIVIK	3	N-Term(iTRAQ4plex); K10(iTRAQ4plex		4.27	46	3	521.63
									GFSLDEATNLNGGLLR	2	N-Term(iTRAQ4plex		4.21	77	2	910.99
56549131	MXRA7	matrix-remodeling-associated protein 7 isoform 1	8.82	0	1	2		21.5								
									GPSSEGPEEEDGEGFSFK	2	N-Term(iTRAQ4plex); K18(iTRAQ4plex		3.24	67	2	1086.99
4758280	EPHA4	ephrin type-A receptor 4 precursor	8.82	7	7	42	0.828	109.8								
									ETFNLYYYESDNDKER	2	N-Term(iTRAQ4plex); K14(iTRAQ4plex	0.918	7.09	59	3	792.04
									NLAQFPDTITGADTSSLVEVR	13	N-Term(iTRAQ4plex	0.85	6.5	86	3	793.42
									IDTIAADESFTQVDIGDR	10	N-Term(iTRAQ4plex	0.755	5.92	114	2	1055.53
									VYPANEVTLLDSR	10	N-Term(iTRAQ4plex	0.809	5.45	78	2	810.94
									GLNPLTSYVFHVR	5	N-Term(iTRAQ4plex	1.114		68		
									ETFNLYYYESDNDK	1	N-Term(iTRAQ4plex); K14(iTRAQ4plex	0.78		51		
									VYIEIK	1	N-Term(iTRAQ4plex); K6(iTRAQ4plex	0.711		35		
119395754	KRT5	keratin, type II cytoskeletal 5	8.81	2	5	12	1.516	62.3								
									EYQELMNTK	2	N-Term(iTRAQ4plex); K9(iTRAQ4plex	1.588	4.47	52	2	722.37
									WTLLQEQGTK	4	N-Term(iTRAQ4plex); K10(iTRAQ4plex		4.27	67	2	746.42
									FASFIDK	4	N-Term(iTRAQ4plex); K7(iTRAQ4plex		3.19	53	2	558.31
									QCANLQNAIADAEQR	1	N-Term(iTRAQ4plex); C2(Methylthio	1.448		56		
209862865	KLK11	kallikrein-11 isoform 1 precursor	8.8	0	2	3		27.4								
									YIVHLGQHNLQK	2	N-Term(iTRAQ4plex); K12(iTRAQ4plex		6.76	56	3	580.00
									YVDWIQETMK	1	N-Term(iTRAQ4plex); K10(iTRAQ4plex			40		
310124897	LOC100507680	PREDICTED: HLA class I histocompatibility antigen, A-32 alpha chain-like isoform 7	8.79	0	2	3		30.3								
									DYIALNEDLR	2	N-Term(iTRAQ4plex		3.38	41	2	683.36
									FIAVGYVDDTQFVR	2	N-Term(iTRAQ4plex		3.38	70	2	887.47
310125063	LOC100507680	PREDICTED: HLA class I histocompatibility antigen, A-43 alpha chain-like isoform 10	8.79	0	2	3		30.5								
									DYIALNEDLR	2	N-Term(iTRAQ4plex		3.38	41	2	683.36
									FIAVGYVDDTQFVR	2	N-Term(iTRAQ4plex		3.38	70	2	887.47
310125172	LOC100507680	PREDICTED: HLA class I histocompatibility antigen, A-74 alpha chain-like isoform 6	8.79	0	2	3		30.5								
									DYIALNEDLR	2	N-Term(iTRAQ4plex		3.38	41	2	683.36
									FIAVGYVDDTQFVR	2	N-Term(iTRAQ4plex		3.38	70	2	887.47
310128255	LOC100510327	PREDICTED: HLA class I histocompatibility antigen, A-30 alpha chain-like isoform 6	8.79	0	2	3		30.3								
									DYIALNEDLR	2	N-Term(iTRAQ4plex		3.38	41	2	683.36
									FIAVGYVDDTQFVR	2	N-Term(iTRAQ4plex		3.38	70	2	887.47
5901956	FSTL1	follistatin-related protein 1 precursor	8.77	3	3	13	1.001	35								
									LSFQEFLK	8	N-Term(iTRAQ4plex); K8(iTRAQ4plex	0.941	4.11	60	2	650.38
									CLNPSFNPPEK	3	N-Term(iTRAQ4plex); C1(Methylthio); K11(iTRAQ4plex)	0.96	3.63	52	2	790.40
									LDSSEFLK	2	N-Term(iTRAQ4plex); K8(iTRAQ4plex	1.223	3.16	36	2	613.84
4503487	EFNA5	ephrin-A5 precursor	8.77	1	1	1	1.147	26.3								
									VENSLEPADDTVHESAEPSR	1	N-Term(iTRAQ4plex	1.147		39		
5031857	LDHA	L-lactate dehydrogenase A chain isoform 1	8.73	0	3	3		36.7								
									DQLIYNLLK	1	N-Term(iTRAQ4plex); K9(iTRAQ4plex			42		
									LVIITAGAR	1	N-Term(iTRAQ4plex			38		
									QVVESAYEVIK	1	N-Term(iTRAQ4plex); K11(iTRAQ4plex			35		

166235136	SEZ6L2	seizure 6-like protein 2 isoform 2	8.68	0	6	32		97.5								
									TASDAGFPVGSHVQYF	13	N-Term(iTRAQ4plex		5.93	112	2	918.46
									EGDMLTLFDGDGPSAR	8	N-Term(iTRAQ4plex		5.06	86	2	912.93
									SGGSPLSPVIYDSMDDVPER	4	N-Term(iTRAQ4plex		3.49	86	2	1190.56
									LLLHFQSPR	4	N-Term(iTRAQ4plex		3.48	44	2	627.87
									WVIEAAEGR	2	N-Term(iTRAQ4plex		3.39	38	2	587.82
									FEAFEEDR	1	N-Term(iTRAQ4plex			39		
4506803	CLEC11A	C-type lectin domain family 11 member A precursor	8.67	2	2	3	0.767	35.7								
									EWEGGWGGAQEEER	2	N-Term(iTRAQ4plex	0.844		43		
									LAGLDAGLHQLHVF	1	N-Term(iTRAQ4plex	0.602		36		
21264361	MASP2	mannan-binding lectin serine protease 2 isoform 2 precursor	8.65	0	1	2		20.6								
									LASPGFPGEYANDQER	2	N-Term(iTRAQ4plex		3.61	63	2	947.95
166235140	SEZ6L2	seizure 6-like protein 2 isoform 4	8.65	0	5	28		86.8								
									TASDAGFPVGSHVQYF	13	N-Term(iTRAQ4plex		5.93	112	2	918.46
									EGDMLTLFDGDGPSAR	8	N-Term(iTRAQ4plex		5.06	86	2	912.93
									SGGSPLSPVIYDSMDDVPER	4	N-Term(iTRAQ4plex		3.49	86	2	1190.56
									WVIEAAEGR	2	N-Term(iTRAQ4plex		3.39	38	2	587.82
									FEAFEEDR	1	N-Term(iTRAQ4plex			39		
23503267	ENPP6	ectonucleotide pyrophosphatase/phosphodiesterase family member 6 precursor	8.64	3	3	7	0.796	50.2								
									GWHGYDNELMDMR	2	N-Term(iTRAQ4plex	1.004	3.75	62	3	589.93
									IDVEGHHYGPASPQF	2	N-Term(iTRAQ4plex	0.745	3.44	51	3	602.97
									ADLAAIYHER	3	N-Term(iTRAQ4plex	0.796	3.28	53	2	651.85
157819143	IGLON5	igLON family member 5 precursor	8.63	2	2	4	0.932	36.8								
									LLSSGTAEGLK	2	N-Term(iTRAQ4plex); K11(iTRAQ4plex	0.889	3.66	41	2	682.41
									VLVTVNYPPTITDVTSAF	2	N-Term(iTRAQ4plex	0.978	3.4	43	2	1045.59
4757910	CARTPT	cocaine- and amphetamine-regulated transcript protein preproprotein	8.62	1	1	3	0.821	12.8								
									GTSCNSFLLK	3	N-Term(iTRAQ4plex); C4(Methylthio); K10(iTRAQ4plex)	0.821	4.41	53	2	702.37
189011548	ASAH1	acid ceramidase isoform a preproprotein	8.61	0	4	8		44.6								
									IMQVVDEK	2	N-Term(iTRAQ4plex); K8(iTRAQ4plex		3.88	53	2	625.36
									WYVVQTNYDR	2	N-Term(iTRAQ4plex		3.53	41	2	744.37
									HPFFLDDR	2	N-Term(iTRAQ4plex			42		
258613948	ASPH	aspartyl/asparaginyl beta-hydroxylase isoform h	8.6	0	2	3		31.5								
									LGIYDADGDGDFDVDDAK	2	N-Term(iTRAQ4plex); K18(iTRAQ4plex		6.31	74	2	1095.01
									VLLGLK	1	N-Term(iTRAQ4plex); K6(iTRAQ4plex			32		
48255941	CD44	CD44 antigen isoform 4 precursor	8.59	0	3	72		39.4								
									YGFIEGHVVIPR	47	N-Term(iTRAQ4plex		4.96	83	2	765.93
									ALSIGFETCR	13	N-Term(iTRAQ4plex); C9(Methylthio		4.45	53	2	643.82
									FAGVFHVEK	12	N-Term(iTRAQ4plex); K9(iTRAQ4plex		3.55	79	2	661.38
14589862	ASPH	aspartyl/asparaginyl beta-hydroxylase isoform d	8.57	0	1	2		23.8								
									LGIYDADGDGDFDVDDAK	2	N-Term(iTRAQ4plex); K18(iTRAQ4plex		6.31	74	2	1095.01
189242612	C6	complement component C6 precursor	8.57	7	7	27	1.04	104.7								
									GEVLDNSFTGGICK	4	N-Term(iTRAQ4plex); C13(Methylthio); K14(iTRAQ4plex)	1.024	6.19	96	2	887.44
									SEYGAALAWEK	2	N-Term(iTRAQ4plex); K11(iTRAQ4plex	1.226	5.54	69	2	756.90
									DLHLSDVFLK	5	N-Term(iTRAQ4plex); K10(iTRAQ4plex	1.012	4.77	60	3	492.29
									ALNHLPLEYNSALYSR	8	N-Term(iTRAQ4plex	0.994	4.14	55	3	669.02
									TFSEWLESVK	4	N-Term(iTRAQ4plex); K10(iTRAQ4plex	1.111	4.02	73	2	757.41
									IGESIELTCPK	2	N-Term(iTRAQ4plex); C9(Methylthio); K11(iTRAQ4plex)	1.04	3.53	43	2	762.41
									QLEWGLER	2	N-Term(iTRAQ4plex	1.06	3.24	37	2	587.82
65506891	SLC3A2	4F2 cell-surface antigen heavy chain isoform c	8.57	0	5	9		68								
									GQSEDPGSLLSLFR	2	N-Term(iTRAQ4plex		3.83	67	2	825.44
									EDFDSLLQSAK	2	N-Term(iTRAQ4plex); K11(iTRAQ4plex		3.65	82	2	770.91
									LDYLSSLK	3	N-Term(iTRAQ4plex); K8(iTRAQ4plex		3.27	51	2	613.87
									LLTSFLPAQLLR	1	N-Term(iTRAQ4plex			44		
									GLVLGPIHK	1	N-Term(iTRAQ4plex); K9(iTRAQ4plex			32		
40255029	CCDC126	coiled-coil domain-containing protein 126 precursor	8.57	1	1	1	0.892	15.7								
									TIAVLLDDILQR	1	N-Term(iTRAQ4plex	0.892		72		
61744477	SLC3A2	4F2 cell-surface antigen heavy chain isoform b	8.56	0	5	9		68.1								
									GQSEDPGSLLSLFR	2	N-Term(iTRAQ4plex		3.83	67	2	825.44
									EDFDSLLQSAK	2	N-Term(iTRAQ4plex); K11(iTRAQ4plex		3.65	82	2	770.91
									LDYLSSLK	3	N-Term(iTRAQ4plex); K8(iTRAQ4plex		3.27	51	2	613.87
									LLTSFLPAQLLR	1	N-Term(iTRAQ4plex			44		
									GLVLGPIHK	1	N-Term(iTRAQ4plex); K9(iTRAQ4plex			32		
21328448	YWHAB	14-3-3 protein beta/alpha	8.54	1	2	4	0.801	28.1								
									YLSEVASGDNK	2	N-Term(iTRAQ4plex); K11(iTRAQ4plex	0.801	4.55	67	2	735.89
									DSTLIMQLLR	2	N-Term(iTRAQ4plex		4.44	67	2	667.39
27262659	CSF1R	macrophage colony-stimulating factor 1 receptor precursor	8.54	6	6	35	0.861	107.9								
									LAIPQQSDFHNNF	12	N-Term(iTRAQ4plex	0.839	5.6	74	3	561.96
									VIPGPPALTLVPAELVF	11	N-Term(iTRAQ4plex	0.821	5.5	80	2	943.59
									CTEPGDPLGGSAAIHLYVK	5	N-Term(iTRAQ4plex); C1(Methylthio); K19(iTRAQ4plex)	0.888	4.72	63	3	754.72
									FIQSQDYQCSALMGGR	3	N-Term(iTRAQ4plex); C9(Methylthio	0.945	4.09	45	2	997.46
									ALTFELTLR	3	N-Term(iTRAQ4plex	0.855	3.53	51	2	604.36
									HTFTLSLPR	1	N-Term(iTRAQ4plex	0.931		35		
155969697	KRT6C	keratin, type II cytoskeletal 6C	8.51	0	5	13		60								
									WTLLQEQGTK	4	N-Term(iTRAQ4plex); K10(iTRAQ4plex		4.27	67	2	746.42
									TAAENEFVTLK	2	N-Term(iTRAQ4plex); K11(iTRAQ4plex		3.68	44	2	755.92
									EYQELMNVK	2	N-Term(iTRAQ4plex); K9(iTRAQ4plex		3.39	60	2	721.38
									FASFIDK	4	N-Term(iTRAQ4plex); K7(iTRAQ4plex		3.19	53	2	558.31
4506309	PTPRD	receptor-type tyrosine-protein phosphatase delta isoform 1 precursor	8.47	0	11	24		214.6								
									SDTIANYELVYK	4	N-Term(iTRAQ4plex); K12(iTRAQ4plex		5.58	74	2	852.46
									SPQGLGASTAEISAR	4	N-Term(iTRAQ4plex		5.34	96	2	794.93
									YSVAGLSPYSDYEFR	2	N-Term(iTRAQ4plex		5.01	65	2	949.46
									GFPTIDMGPQLK	2	N-Term(iTRAQ4plex); K12(iTRAQ4plex		4.89	87	2	796.44
									GALQIEQSEESDQGK	2	N-Term(iTRAQ4plex); K15(iTRAQ4plex		4.87	85	2	953.98
									YECVATNSAGTF	2	N-Term(iTRAQ4plex); C3(Methylthio		3.82	58	2	731.34
									WMLGAEDLTPEDDMPIGR	2	N-Term(iTRAQ4plex		3.43	45	2	1095.52

									FIKPWESPDEMELDELLK	2	N-Term(iTRAQ4plex); K3(iTRAQ4plex); K18(iTRAQ4plex)		3.43	65	3	884.47
									FEVIEFDDGSGSVLR	2	N-Term(iTRAQ4plex		3.19	41	2	907.46
									GPPSEPVLTQTSEQAPSSAPF	1	N-Term(iTRAQ4plex			44		
88702793	VASN	vasorin precursor	8.47	4	4	11	0.877	71.7								
									SLTLGIEPVSPTSLR	3	N-Term(iTRAQ4plex	0.846	5.29	59	2	857.50
									NLHDLDVSDNQLER	4	N-Term(iTRAQ4plex	0.825	5.14	77	2	906.45
									HIQPGAFDTLDR	3	N-Term(iTRAQ4plex	0.943	5	47	3	505.27
									LAGLGLQQLDEGLFSR	1	N-Term(iTRAQ4plex	0.79		76		
19923106	PON1	serum paraoxonase/arylesterase 1 precursor	8.45	2	3	7	1.111	39.7								
									IQNILTEEPK	4	N-Term(iTRAQ4plex); K10(iTRAQ4plex	1.223	4.61	53	2	736.93
									LLIGTVFHK	3	N-Term(iTRAQ4plex); K9(iTRAQ4plex		4.07	52	3	439.28
									YVYIAELLAHK	2	N-Term(iTRAQ4plex); K11(iTRAQ4plex	1.111		36		
4758540	CHST10	carbohydrate sulfotransferase 10	8.43	3	3	4	0.989	42.2								
									LSSFSDAEIQK	2	N-Term(iTRAQ4plex); K11(iTRAQ4plex	0.989	5.23	82	2	756.91
									VEHYFLGISK	1	N-Term(iTRAQ4plex); K10(iTRAQ4plex	0.877		47		
									GIQFEDFVR	1	N-Term(iTRAQ4plex	1.044		46		
84798622	MANBA	beta-mannosidase precursor	8.42	6	6	11	1.002	100.8								
									TVELIEEPIK	2	N-Term(iTRAQ4plex); K10(iTRAQ4plex	0.958	4.33	71	2	729.94
									VSSTEDWSFNSK	2	N-Term(iTRAQ4plex); K12(iTRAQ4plex	1.098	4.33	85	2	837.92
									VNLILEGVDTVSK	2	N-Term(iTRAQ4plex); K13(iTRAQ4plex	1.047	4.02	45	2	838.00
									FSDNGFLMTEK	2	N-Term(iTRAQ4plex); K11(iTRAQ4plex	0.71	3.81	53	2	788.90
									GSNWIPADSFQDR	2	N-Term(iTRAQ4plex	0.849	3.71	31	2	818.89
									LLLQSVVDANMNTLF	1	N-Term(iTRAQ4plex	1.057		69		
300244535	IL6ST	interleukin-6 receptor subunit beta isoform 3 precursor	8.4	0	7	17		96.2								
									VTSDHINFDPVYK	2	N-Term(iTRAQ4plex); K13(iTRAQ4plex		5.2	39	3	608.32
									DGPEFTFTTPK	2	N-Term(iTRAQ4plex); K11(iTRAQ4plex		4.86	47	2	764.40
									DASTWSQIPPEDTASTR	4	N-Term(iTRAQ4plex		4.4	59	2	1003.49
									LTWTNPSIK	3	N-Term(iTRAQ4plex); K9(iTRAQ4plex		4.27	64	2	674.40
									YLATLTVR	2	N-Term(iTRAQ4plex		3.28	41	2	540.83
									TVQLVWK	3	N-Term(iTRAQ4plex); K7(iTRAQ4plex		3.24	39	2	581.36
									APSFWYK	1	N-Term(iTRAQ4plex); K7(iTRAQ4plex			38		
72534766	MANEAL	glycoprotein endo-alpha-1,2-mannosidase-like protein isoform 1	8.4	0	1	1		27.4								
									HSPPDDLGSSFYPELGPYSSR	1	N-Term(iTRAQ4plex			36		
44889963	CD163	scavenger receptor cysteine-rich type 1 protein M130 isoform b	8.39	0	7	14		121.5								
									EAEFGQGTGPIWLNEVK	2	N-Term(iTRAQ4plex); K17(iTRAQ4plex		5.4	82	2	1082.07
									CAGTVEVEIQR	2	N-Term(iTRAQ4plex); C1(Methylthio		4.86	62	2	697.85
									NWQWGGLTCDHYEEAK	2	N-Term(iTRAQ4plex); C9(Methylthio); K16(iTRAQ4plex)		4.22	69	3	757.68
									LVDGVTECSGR	2	N-Term(iTRAQ4plex); C8(Methylthio		3.61	51	2	663.32
									LVGGDIPCSGR	3	N-Term(iTRAQ4plex); C8(Methylthio		3.6	43	2	632.32
									SSMSETTVGVVCR	2	N-Term(iTRAQ4plex); C12(Methylthio		3.34	54	2	773.36
									LASPSEETWITCDNK	1	N-Term(iTRAQ4plex); C12(Methylthio); K15(iTRAQ4plex)			53		
65506779	NTRK2	BDNF/NT-3 growth factors receptor isoform e precursor	8.38	0	3	5		59.1								
									SSPDTQDLYCLNESSK	2	N-Term(iTRAQ4plex); C10(Methylthio); K16(iTRAQ4plex)		3.28	67	2	1060.99
									RLEIINEDDVEAYVGLR	2	N-Term(iTRAQ4plex			59		
									SNEIPSTDVTDK	1	N-Term(iTRAQ4plex); K12(iTRAQ4plex			57		
72534684	PLD3	phospholipase D3	8.37	4	4	9	0.913	54.7								
									ALLNVVDNAR	4	N-Term(iTRAQ4plex	0.979	4.13	59	2	614.86
									LFVVPADEAQAR	3	N-Term(iTRAQ4plex	0.897	3.75	45	2	730.41
									AFLLSLAALR	1	N-Term(iTRAQ4plex	0.868		39		
259089450	GFRA2	GDNF family receptor alpha-2 isoform b	8.36	0	3	8		39.6								
									QTILPSCSYEDK	2	N-Term(iTRAQ4plex); C7(Methylthio); K12(iTRAQ4plex)		4.67	77	2	859.43
									DFTENPCLR	4	N-Term(iTRAQ4plex); C7(Methylthio		3.9	34	2	642.79
									SSYISICNR	2	N-Term(iTRAQ4plex); C7(Methylthio		3.42	47	2	616.80
310124909	LOC100507680	PREDICTED: HLA class I histocompatibility antigen, A-32 alpha chain-like isoform 12	8.3	0	2	3		32.3								
									DYIALNEDLR	2	N-Term(iTRAQ4plex		3.38	41	2	683.36
									FIAVGYVDDTQFVR	2	N-Term(iTRAQ4plex		3.38	70	2	887.47
167614506	LCP1	plastin-2	8.29	3	5	10	0.854	70.2								
									GSVSDEEMMELR	2	N-Term(iTRAQ4plex	0.863	4.37	85	2	763.85
									AECMLQQAER	2	N-Term(iTRAQ4plex); C3(Methylthio	0.812	3.82	48	2	684.81
									ISFDEFIK	2	N-Term(iTRAQ4plex); K8(iTRAQ4plex	0.854	3.67	42	2	643.87
									MINLSVPDTIDER	2	N-Term(iTRAQ4plex		3.61	39	2	823.93
									LSPEELLLR	2	N-Term(iTRAQ4plex		3.44	42	2	607.37
71274132	PGAM4	phosphoglycerate mutase 4	8.27	0	1	2		28.8								
									SYDVPPPPMEPDHPFYSNISK	2	N-Term(iTRAQ4plex); K21(iTRAQ4plex		4.76	46	3	902.45
4505753	PGAM1	phosphoglycerate mutase 1	8.27	0	1	2		28.8								
									SYDVPPPPMEPDHPFYSNISK	2	N-Term(iTRAQ4plex); K21(iTRAQ4plex		4.76	46	3	902.45
189011546	ASAH1	acid ceramidase isoform b	8.27	0	4	8		46.5								
									IMQVVDEK	2	N-Term(iTRAQ4plex); K8(iTRAQ4plex		3.88	53	2	625.36
									WYVVQTNYDR	2	N-Term(iTRAQ4plex		3.53	41	2	744.37
									HPFFLDDR	2	N-Term(iTRAQ4plex			42		
310125077	LOC100507680	PREDICTED: HLA class I histocompatibility antigen, A-43 alpha chain-like isoform 21	8.22	0	2	3		32.5								
									DYIALNEDLR	2	N-Term(iTRAQ4plex		3.38	41	2	683.36
									FIAVGYVDDTQFVR	2	N-Term(iTRAQ4plex		3.38	70	2	887.47
310125178	LOC100507680	PREDICTED: HLA class I histocompatibility antigen, A-74 alpha chain-like isoform 11	8.22	0	2	3		32.6								
									DYIALNEDLR	2	N-Term(iTRAQ4plex		3.38	41	2	683.36
									FIAVGYVDDTQFVR	2	N-Term(iTRAQ4plex		3.38	70	2	887.47
310124839	LOC100507705	PREDICTED: class I histocompatibility antigen, Gogo-B*0103 alpha chain-like isoform 12	8.19	0	2	3		32.4								
									SWTAADTAAQITQR	2	N-Term(iTRAQ4plex		4.98	72	2	832.43
									DYIALNEDLR	2	N-Term(iTRAQ4plex		3.38	41	2	683.36
168693643	CD55	complement decay-accelerating factor isoform 2 precursor	8.18	0	4	22		48.7								
									TSFPEDTVITYK	13	N-Term(iTRAQ4plex); K12(iTRAQ4plex		4.74	92	2	844.96
									LTCLQNLK	5	N-Term(iTRAQ4plex); C3(Methylthio); K8(iTRAQ4plex)		4.13	64	2	633.86

									WSTAVEFCK	2	N-Term(iTRAQ4plex); C8(Methylthio); K9(iTRAQ4plex)		3.91	48	2	702.85
									DSVICLK	2	N-Term(iTRAQ4plex); C5(Methylthio); K7(iTRAQ4plex)			41		
264681535	CADM2	cell adhesion molecule 2 isoform 2	8.17	0	3	10		44.4								
									GKPLPEPVLWTK	6	N-Term(iTRAQ4plex); K2(iTRAQ4plex); K12(iTRAQ4plex)		4.74	57	3	599.71
									TFTVSSTLDFR	2	N-Term(iTRAQ4plex		4.32	48	2	709.38
									SDDGVAVICR	2	N-Term(iTRAQ4plex); C9(Methylthio		3.23	64	2	612.80
258613953	ASPH	aspartyl/asparaginyl beta-hydroxylase isoform j	8.16	0	2	3		32.4								
									LGIYDADGDGDFDVDDAK	2	N-Term(iTRAQ4plex); K18(iTRAQ4plex		6.31	74	2	1095.01
									VLLGLK	1	N-Term(iTRAQ4plex); K6(iTRAQ4plex			32		
65506769	NTRK2	BDNF/NT-3 growth factors receptor isoform d precursor	8.14	0	3	5		61								
									SSPDTQDLYCLNESSK	2	N-Term(iTRAQ4plex); C10(Methylthio); K16(iTRAQ4plex)		3.28	67	2	1060.99
									RLEIINEDDVEAYVGLR	2	N-Term(iTRAQ4plex			59		
									SNEIPSTDVTDK	1	N-Term(iTRAQ4plex); K12(iTRAQ4plex			57		
44662834	CD163	scavenger receptor cysteine-rich type 1 protein M130 isoform a	8.13	0	7	14		125.4								
									EAEFGQGTGPIWLNEVK	2	N-Term(iTRAQ4plex); K17(iTRAQ4plex		5.4	82	2	1082.07
									CAGTVEVEIQR	2	N-Term(iTRAQ4plex); C1(Methylthio		4.86	62	2	697.85
									NWQWGGLTCDHYEEAK	2	N-Term(iTRAQ4plex); C9(Methylthio); K16(iTRAQ4plex)		4.22	69	3	757.68
									LVDGVTECSGR	2	N-Term(iTRAQ4plex); C8(Methylthio		3.61	51	2	663.32
									LVGGDIPCSGR	3	N-Term(iTRAQ4plex); C8(Methylthio		3.6	43	2	632.32
									SSMSETTVGVVCR	2	N-Term(iTRAQ4plex); C12(Methylthio		3.34	54	2	773.36
									LASPSEETWITCDNK	1	N-Term(iTRAQ4plex); C12(Methylthio); K15(iTRAQ4plex)			53		
14249348	TXNDC17	thioredoxin domain-containing protein 17	8.13	1	1	1	0.86	13.9								
									TIFAYFTGSK	1	N-Term(iTRAQ4plex); K10(iTRAQ4plex	0.86		41		
4505423	NOV	protein NOV homolog precursor	8.12	3	3	4	1.007	39.1								
									NNEAFLQELELK	2	N-Term(iTRAQ4plex); K12(iTRAQ4plex	1.01	4.26	75	2	868.48
									KVEVPGECCEK	1	N-Term(iTRAQ4plex); K1(iTRAQ4plex); C8(Methylthio); C9(Methylthio); K11(iTRAQ4plex)	1.007		38		
									FQCTCR	1	N-Term(iTRAQ4plex); C3(Methylthio); C5(Methylthio)	0.763		21		
40317628	THBS2	thrombospondin-2 precursor	8.11	5	7	12	0.938	129.9								
									QVTQTYWEDQPTR	2	N-Term(iTRAQ4plex	0.931	5.22	84	2	898.44
									LVFNPDQEDLDGDGR	2	N-Term(iTRAQ4plex	0.983	5.14	80	2	917.44
									QVMADSGPIYDQTYAGGR	2	N-Term(iTRAQ4plex	1.129	4.99	95	2	1037.00
									VCNSPEPQYGGK	2	N-Term(iTRAQ4plex); C2(Methylthio); K12(iTRAQ4plex)	0.938	4.67	74	2	806.89
									NALWHTGNTPGQVR	2	N-Term(iTRAQ4plex		4.16	46	3	565.63
									FYVVMWK	1	N-Term(iTRAQ4plex); K7(iTRAQ4plex			43		
									LGPHVTTEYVGPSSER	1	N-Term(iTRAQ4plex	0.682		40		
4506147	PRSS2	trypsin-2 preproprotein	8.1	0	1	4		26.5								
									LGEHNIEVLEGNEQFINAAK	4	N-Term(iTRAQ4plex); K20(iTRAQ4plex		6.23	62	3	838.45
4506145	PRSS1	trypsin-1 preproprotein	8.1	0	1	4		26.5								
									LGEHNIEVLEGNEQFINAAK	4	N-Term(iTRAQ4plex); K20(iTRAQ4plex		6.23	62	3	838.45
21464101	YWHAG	14-3-3 protein gamma	8.1	1	2	4	0.78	28.3								
									YLAEVATGEK	2	N-Term(iTRAQ4plex); K10(iTRAQ4plex	0.78	4.65	59	2	684.89
									DSTLIMQLLR	2	N-Term(iTRAQ4plex		4.44	67	2	667.39
21361091	UCHL1	ubiquitin carboxyl-terminal hydrolase isozyme L1	8.07	1	1	2	0.69	24.8								
									NEAIQAAHDAVAQEGQCR	2	N-Term(iTRAQ4plex); C17(Methylthio	0.69	5.87	82	3	700.99
263190474	GM2A	ganglioside GM2 activator isoform 2 precursor	8.06	0	2	24		20.1								
									EVAGLWIK	16	N-Term(iTRAQ4plex); K8(iTRAQ4plex		4.17	54	2	602.37
									VDLVLEK	8	N-Term(iTRAQ4plex); K7(iTRAQ4plex		3.33	43	2	552.35
50263044	LINGO1	leucine-rich repeat neuronal 6A	8.06	3	4	8	0.74	69.8								
									SLEVGDNDLVYISHR	3	N-Term(iTRAQ4plex		5.04	37	3	620.99
									FVAVPEGIPTETR	2	N-Term(iTRAQ4plex	0.717	3.4	43	2	780.43
									LTVFPDGTLEVR	1	N-Term(iTRAQ4plex	0.744		59		
									ATVPFPFDIK	2	N-Term(iTRAQ4plex); K10(iTRAQ4plex	0.72		47		
258613946	ASPH	aspartyl/asparaginyl beta-hydroxylase isoform g	8.05	0	2	3		33.7								
									LGIYDADGDGDFDVDDAK	2	N-Term(iTRAQ4plex); K18(iTRAQ4plex		6.31	74	2	1095.01
									VLLGLK	1	N-Term(iTRAQ4plex); K6(iTRAQ4plex			32		
4557389	C8A	complement component C8 alpha chain precursor	8.05	4	4	14	0.955	65.1								
									INVGGGLSGDHCK	2	N-Term(iTRAQ4plex); C12(Methylthio); K13(iTRAQ4plex)	0.993	6.41	51	3	530.94
									LGSLGAACEQTQTEGAK	2	N-Term(iTRAQ4plex); C8(Methylthio); K17(iTRAQ4plex)	1.218	5.77	107	2	999.50
									AMAVEDIISR	4	N-Term(iTRAQ4plex	0.936	3.8	53	2	624.84
									KPYNFLK	3	N-Term(iTRAQ4plex); K1(iTRAQ4plex); K7(iTRAQ4plex)	0.929	3.66	43	3	447.94
									AMAVEDIISR	3	N-Term(iTRAQ4plex); M2(Oxidation	0.938		45		
57242800	SMPDL3B	acid sphingomyelinase-like phosphodiesterase 3b isoform 2	8.04	0	2	2		41.7								
									AGDMVYIVGHVPPGFFEK	1	N-Term(iTRAQ4plex); K18(iTRAQ4plex			37		
									LGEAAVLEIVER	1	N-Term(iTRAQ4plex			35		
14589864	ASPH	aspartyl/asparaginyl beta-hydroxylase isoform b	8.03	0	2	3		33.8								
									LGIYDADGDGDFDVDDAK	2	N-Term(iTRAQ4plex); K18(iTRAQ4plex		6.31	74	2	1095.01
									VLLGLK	1	N-Term(iTRAQ4plex); K6(iTRAQ4plex			32		
310124907	LOC100507680	PREDICTED: HLA class I histocompatibility antigen, A-32 alpha chain-like isoform 9	8.03	0	2	3		34.1								
									DYIALNEDLR	2	N-Term(iTRAQ4plex		3.38	41	2	683.36
									FIAVGYVDDTQFVR	2	N-Term(iTRAQ4plex		3.38	70	2	887.47
310125073	LOC100507680	PREDICTED: HLA class I histocompatibility antigen, A-43 alpha chain-like isoform 13	8.03	0	2	3		34.4								
									DYIALNEDLR	2	N-Term(iTRAQ4plex		3.38	41	2	683.36
									FIAVGYVDDTQFVR	2	N-Term(iTRAQ4plex		3.38	70	2	887.47
310125184	LOC100507680	PREDICTED: HLA class I histocompatibility antigen, A-74 alpha chain-like isoform 8	8.03	0	2	3		34.3								
									DYIALNEDLR	2	N-Term(iTRAQ4plex		3.38	41	2	683.36
									FIAVGYVDDTQFVR	2	N-Term(iTRAQ4plex		3.38	70	2	887.47

310128263	LOC100510327	PREDICTED: HLA class I histocompatibility antigen, A-30 alpha chain-like isoform 11	8.03	0	2	3		34.1								
									DYIALNEDLR	2	N-Term(iTRAQ4plex		3.38	41	2	683.36
									FIAVGYVDDTQFVF	2	N-Term(iTRAQ4plex		3.38	70	2	887.47
24430192	KRT16	keratin, type I cytoskeletal 16	8.03	0	4	11		51.2								
									ASLENSLEETK	4	N-Term(iTRAQ4plex); K11(iTRAQ4plex		5.06	82	2	754.90
									LEQEIATYR	2	N-Term(iTRAQ4plex		4.13	44	2	633.84
									LASYLDK	4	N-Term(iTRAQ4plex); K7(iTRAQ4plex		3.35	53	2	549.32
									ISSVLAGGSCR	1	N-Term(iTRAQ4plex); C10(Methylthio			36		
260099723	LDHA	L-lactate dehydrogenase A chain isoform 3	8.03	0	3	3		39.8								
									DQLIYNLLK	1	N-Term(iTRAQ4plex); K9(iTRAQ4plex			42		
									LVIITAGAR	1	N-Term(iTRAQ4plex			38		
									QVVESAYEVIK	1	N-Term(iTRAQ4plex); K11(iTRAQ4plex			35		
115298674	NID1	nidogen-1 precursor	8.02	7	8	19	0.946	136.3								
									QDLGSPEGIAVDHLGR	8	N-Term(iTRAQ4plex	0.909	6.21	75	3	603.32
									VLFETDLVNPR	2	N-Term(iTRAQ4plex	0.861	4.26	51	2	723.91
									GNLYWTDWNR	2	N-Term(iTRAQ4plex		4.08	43	2	734.86
									GGADTYSVPSVLSPR	2	N-Term(iTRAQ4plex	1.02	3.8	70	2	825.43
									NIFWTDSNLDR	2	N-Term(iTRAQ4plex	0.955	3.32	58	2	762.89
									QAEVTFVGHPGNLVIK	1	N-Term(iTRAQ4plex); K16(iTRAQ4plex	1.19		70		
									ASLHGGEPTTIIR	1	N-Term(iTRAQ4plex	0.895		37		
									AFLHVPAK	1	N-Term(iTRAQ4plex); K8(iTRAQ4plex	0.957		32		
9910364	ASPH	aspartyl/asparaginyl beta-hydroxylase isoform e	8	0	1	2		25.5								
									LGIYDADGDGDFDVDDAK	2	N-Term(iTRAQ4plex); K18(iTRAQ4plex		6.31	74	2	1095.01
262527289	KLK11	kallikrein-11 isoform 3 precursor	8	0	2	3		30.1								
									YIVHLGQHNLQK	2	N-Term(iTRAQ4plex); K12(iTRAQ4plex		6.76	56	3	580.00
									YVDWIQETMK	1	N-Term(iTRAQ4plex); K10(iTRAQ4plex			40		
91984773	APOA1BP	apolipoprotein A-I-binding protein precursor	7.99	2	2	3	0.697	31.7								
									LFGYEPTIYYPK	2	N-Term(iTRAQ4plex); K12(iTRAQ4plex	0.684	4.05	57	2	889.99
									EPFHSILSVLK	1	N-Term(iTRAQ4plex); K11(iTRAQ4plex	0.711		42		
41872557	NRP2	neuropilin-2 isoform 6 precursor	7.93	0	3	5		62.4								
									IANEQISASSTYSDGR	2	N-Term(iTRAQ4plex		5.52	103	2	921.95
									VFQANNDATEVVLNK	2	N-Term(iTRAQ4plex); K15(iTRAQ4plex		3.93	77	2	975.54
									IVLNFNPHFEIEK	1	N-Term(iTRAQ4plex); K13(iTRAQ4plex			50		
238624149	NPTN	neuroplastin isoform d precursor	7.91	0	2	3		30.8								
									IVTSEEVIIR	2	N-Term(iTRAQ4plex		3.78	38	2	651.89
									SVGYPHPDWIWR	1	N-Term(iTRAQ4plex			34		
41281656	CD99L2	CD99 antigen-like protein 2 isoform 3 precursor	7.89	0	1	6		20.3								
									DLEDIVGGGEYKPDK	6	N-Term(iTRAQ4plex); K12(iTRAQ4plex); K15(iTRAQ4plex)		5.04	41	3	689.71
28373122	CNTN4	contactin-4 precursor	7.89	5	6	10	0.906	113.4								
									TVAANVIGIGEPSRPSEK	2	N-Term(iTRAQ4plex); K18(iTRAQ4plex	0.894	4.83	58	3	705.06
									FVSQETGNLYIAK	2	N-Term(iTRAQ4plex); K13(iTRAQ4plex		4.38	77	2	879.49
									IILNWDQVK	2	N-Term(iTRAQ4plex); K9(iTRAQ4plex	1.039	4	63	2	708.93
									ITISEDGNLR	2	N-Term(iTRAQ4plex	0.743	3.79	44	2	631.34
									LQFAYLDNFK	1	N-Term(iTRAQ4plex); K10(iTRAQ4plex	0.906		44		
7657361	CNTN6	contactin-6 precursor	7.88	6	7	11	0.923	113.9								
									SVQGPPTPLVQR	2	N-Term(iTRAQ4plex	0.865	4.57	54	2	711.91
									FVSQETGNLYIAK	2	N-Term(iTRAQ4plex); K13(iTRAQ4plex		4.38	77	2	879.49
									FPETIQAAK	2	N-Term(iTRAQ4plex); K9(iTRAQ4plex	0.948	3.51	61	2	646.88
									YQIIYANAELR	2	N-Term(iTRAQ4plex	0.822	3.49	38	2	749.41
									IFLLEDGSLK	1	N-Term(iTRAQ4plex); K10(iTRAQ4plex	0.986		64		
									GQLIFYAPPEWEQK	1	N-Term(iTRAQ4plex); K14(iTRAQ4plex	1.198		35		
4758412	GALNT2	polypeptide N-acetylgalactosaminyltransferase 2	7.88	3	4	11	1.01	64.7								
									GGFDWNLVFK	2	N-Term(iTRAQ4plex); K10(iTRAQ4plex	1.031	3.92	64	2	735.91
									FYFEELGK	5	N-Term(iTRAQ4plex); K8(iTRAQ4plex	1.047	3.54	63	2	660.86
									AAEVWMDEYK	2	N-Term(iTRAQ4plex); K10(iTRAQ4plex		3.52	68	2	765.37
									VDLPATSVVITFHNEAR	2	N-Term(iTRAQ4plex	0.969		50		
310124995	LOC100507680	PREDICTED: HLA class I histocompatibility antigen, A-69 alpha chain-like isoform 15	7.87	0	1	2		19.3								
									FIAVGYVDDTQFVF	2	N-Term(iTRAQ4plex		3.38	70	2	887.47
58331240	FETUB	fetuin-B precursor	7.85	3	3	10	1.16	42								
									GSVQYLPDLDDK	4	N-Term(iTRAQ4plex); K12(iTRAQ4plex	1.117	4.62	51	2	819.43
									AIFYMNNPSR	2	N-Term(iTRAQ4plex	1.182	4.1	37	2	678.85
									LVVLPFPK	4	N-Term(iTRAQ4plex); K8(iTRAQ4plex	1.151	3.27	44	2	600.90
28610147	IL6ST	interleukin-6 receptor subunit beta isoform 1 precursor	7.84	0	7	17		103.5								
									VTSDHINFDPVYK	2	N-Term(iTRAQ4plex); K13(iTRAQ4plex		5.2	39	3	608.32
									DGPEFTFTTPK	2	N-Term(iTRAQ4plex); K11(iTRAQ4plex		4.86	47	2	764.40
									DASTWSQIPPEDTASTR	4	N-Term(iTRAQ4plex		4.4	59	2	1003.49
									LTWTNPSIK	3	N-Term(iTRAQ4plex); K9(iTRAQ4plex		4.27	64	2	674.40
									YLATLTVR	2	N-Term(iTRAQ4plex		3.28	41	2	540.83
									TVQLVWK	3	N-Term(iTRAQ4plex); K7(iTRAQ4plex		3.24	39	2	581.36
									APSFWYK	1	N-Term(iTRAQ4plex); K7(iTRAQ4plex			38		
4757768	ARHGDIA	rho GDP-dissociation inhibitor 1 isoform a	7.84	0	1	2		23.2								
									SIQEIQELDKDDESLR	2	N-Term(iTRAQ4plex); K10(iTRAQ4plex		3.64	61	3	736.05
4505185	MIF	macrophage migration inhibitory factor	7.83	1	1	1	0.459	12.5								
									LLCGLLAER	1	N-Term(iTRAQ4plex); C3(Methylthio	0.459		40		
269914187	C4orf48	hypothetical protein LOC401115 isoform 1	7.81	0	1	6		13.7								
									TETLLLQAER	6	N-Term(iTRAQ4plex		4.3	53	2	659.38
21618357	KLK11	kallikrein-11 isoform 2 precursor	7.8	0	2	3		31								
									YIVHLGQHNLQK	2	N-Term(iTRAQ4plex); K12(iTRAQ4plex		6.76	56	3	580.00
									YVDWIQETMK	1	N-Term(iTRAQ4plex); K10(iTRAQ4plex			40		
9257240	NPTN	neuroplastin isoform a precursor	7.8	0	2	3		31.3								
									IVTSEEVIIR	2	N-Term(iTRAQ4plex		3.78	38	2	651.89
									SVGYPHPDWIWR	1	N-Term(iTRAQ4plex			34		
182508169	NRP1	neuropilin-1 isoform a	7.8	0	5	9		103								
									SFEGNNNYDTPELR	2	N-Term(iTRAQ4plex		6.17	74	2	900.42
									IMINFNPHFDLEDR	3	N-Term(iTRAQ4plex		3.47	56	3	635.65
									FVSDYETHGAGFSIR	2	N-Term(iTRAQ4plex			39		
									LEIWDGFPDVGPHIGR	1	N-Term(iTRAQ4plex			35		
310124903	LOC100507680	PREDICTED: HLA class I histocompatibility antigen, A-32 alpha chain-like isoform 13	7.77	0	2	3		34.3								
									DYIALNEDLR	2	N-Term(iTRAQ4plex		3.38	41	2	683.36
									FIAVGYVDDTQFVF	2	N-Term(iTRAQ4plex		3.38	70	2	887.47

310125180	LOC100507680	PREDICTED: HLA class I histocompatibility antigen, A-74 alpha chain-like isoform 12	7.77	0	2	3		34.5								
									DYIALNEDLR	2	N-Term(iTRAQ4plex		3.38	41	2	683.36
									FIAVGYVDDTQFVF	2	N-Term(iTRAQ4plex		3.38	70	2	887.47
310125109	LOC100507681	PREDICTED: HLA class I histocompatibility antigen, Cw-15 alpha chain-like isoform 10	7.72	0	2	3		34.5								
									APWVEQEGPEYWDR	2	N-Term(iTRAQ4plex		3.42	63	2	953.45
									DYIALNEDLR	2	N-Term(iTRAQ4plex		3.38	41	2	683.36
281306830	IGSF21	immunoglobulin superfamily member 21 precursor	7.71	3	3	10	0.98	51.8								
									LLDGSAEFDGK	2	N-Term(iTRAQ4plex); K11(iTRAQ4plex	0.906	4.54	75	2	720.39
									SLSLLDAENR	5	N-Term(iTRAQ4plex	1.125	4.12	64	2	631.35
									CTAQNPLGSTDTHTF	3	N-Term(iTRAQ4plex); C1(Methylthio	0.955	3.44	67	2	896.42
261878616	ITIH1	inter-alpha-trypsin inhibitor heavy chain H1 isoform c	7.7	0	3	24		69.5								
									GSLVQASEANLQAAQDFVF	5	N-Term(iTRAQ4plex		6.6	104	2	1074.56
									QYYEGSEIVVAGR	17	N-Term(iTRAQ4plex		5.65	78	2	807.92
									GFSLDEATNLNGGLLR	2	N-Term(iTRAQ4plex		4.21	77	2	910.99
310128261	LOC100510327	PREDICTED: HLA class I histocompatibility antigen, A-30 alpha chain-like isoform 12	7.69	0	2	3		34.4								
									DYIALNEDLR	2	N-Term(iTRAQ4plex		3.38	41	2	683.36
									FIAVGYVDDTQFVF	2	N-Term(iTRAQ4plex		3.38	70	2	887.47
14589860	ASPH	aspartyl/asparaginyl beta-hydroxylase isoform c	7.67	0	2	3		34.6								
									LGIYDADGDGDFDVDDAK	2	N-Term(iTRAQ4plex); K18(iTRAQ4plex		6.31	74	2	1095.01
									VLLGLK	1	N-Term(iTRAQ4plex); K6(iTRAQ4plex			32		
222144239	FGFR2	fibroblast growth factor receptor 2 isoform 7 precursor	7.66	0	5	13		79.2								
									VYSDAQPHIQWIK	3	N-Term(iTRAQ4plex); K13(iTRAQ4plex		4.36	43	3	625.01
									TVLIGEYLQIK	2	N-Term(iTRAQ4plex); K11(iTRAQ4plex		4.34	58	2	782.98
									DAAVISWTK	4	N-Term(iTRAQ4plex); K9(iTRAQ4plex		4.25	66	2	639.87
									YGPDGLPYLK	2	N-Term(iTRAQ4plex); K10(iTRAQ4plex		3.92	48	2	705.90
									DSGLYACTASR	2	N-Term(iTRAQ4plex); C7(Methylthio		3.66	69	2	667.30
67782309	SOD2	superoxide dismutase [Mn], mitochondrial isoform B precursor	7.65	0	1	2		20.7								
									AIWNVINWENVTEF	2	N-Term(iTRAQ4plex		3.23	62	2	944.50
94818891	ERAP1	endoplasmic reticulum aminopeptidase 1 isoform b precursor	7.65	0	7	12		107.2								
									ILASTQFEPTAAR	2	N-Term(iTRAQ4plex		4.92	70	2	774.93
									EMFDDVSYDK	2	N-Term(iTRAQ4plex); K10(iTRAQ4plex		4.54	67	2	768.86
									DMNEVETQFK	2	N-Term(iTRAQ4plex); K10(iTRAQ4plex		4.22	48	2	764.89
									LSEEPLQVLEHPR	3	N-Term(iTRAQ4plex		3.42	42	3	564.31
									EYLSADAFK	1	N-Term(iTRAQ4plex); K9(iTRAQ4plex			50		
									SQIEFALCR	1	N-Term(iTRAQ4plex); C8(Methylthio			49		
									VGDYFFGK	1	N-Term(iTRAQ4plex); K8(iTRAQ4plex			40		
310124847	LOC100507705	PREDICTED: class I histocompatibility antigen, Gogo-B*0103 alpha chain-like isoform 14, partial	7.62	0	2	3		34.9								
									SWTAADTAAQITQF	2	N-Term(iTRAQ4plex		4.98	72	2	832.43
									DYIALNEDLR	2	N-Term(iTRAQ4plex		3.38	41	2	683.36
98986321	GLDN	gliomedin	7.62	4	4	9	1.066	58.9								
									FEFGQETSQTLK	2	N-Term(iTRAQ4plex); K12(iTRAQ4plex	1.126	5.85	87	2	851.95
									TYFNLAVDEK	2	N-Term(iTRAQ4plex); K10(iTRAQ4plex	0.973	4.19	58	2	744.40
									LENALYFDR	4	N-Term(iTRAQ4plex	1.076	3.98	49	2	642.84
									VTFAFDLLGGK	1	N-Term(iTRAQ4plex); K11(iTRAQ4plex	1.066		47		
62530391	SEPP1	selenoprotein P isoform 1 precursor	7.61	0	3	11		43.2								
									DMPASEDLQDLQK	6	N-Term(iTRAQ4plex); K13(iTRAQ4plex		4.02	75	2	889.46
									CINQLLCK	2	N-Term(iTRAQ4plex); C1(Methylthio); C7(Methylthio); K8(iTRAQ4plex)		3.21	49	2	657.84
									DDFLIYDR	3	N-Term(iTRAQ4plex			43		
310125071	LOC100507680	PREDICTED: HLA class I histocompatibility antigen, A-43 alpha chain-like isoform 16	7.59	0	2	3		35								
									DYIALNEDLR	2	N-Term(iTRAQ4plex		3.38	41	2	683.36
									FIAVGYVDDTQFVF	2	N-Term(iTRAQ4plex		3.38	70	2	887.47
310125117	LOC100507681	PREDICTED: HLA class I histocompatibility antigen, Cw-15 alpha chain-like isoform 9, partial	7.59	0	2	3		36								
									APWVEQEGPEYWDR	2	N-Term(iTRAQ4plex		3.42	63	2	953.45
									DYIALNEDLR	2	N-Term(iTRAQ4plex		3.38	41	2	683.36
94818901	ERAP1	endoplasmic reticulum aminopeptidase 1 isoform a precursor	7.59	0	7	12		107.8								
									ILASTQFEPTAAR	2	N-Term(iTRAQ4plex		4.92	70	2	774.93
									EMFDDVSYDK	2	N-Term(iTRAQ4plex); K10(iTRAQ4plex		4.54	67	2	768.86
									DMNEVETQFK	2	N-Term(iTRAQ4plex); K10(iTRAQ4plex		4.22	48	2	764.89
									LSEEPLQVLEHPR	3	N-Term(iTRAQ4plex		3.42	42	3	564.31
									EYLSADAFK	1	N-Term(iTRAQ4plex); K9(iTRAQ4plex			50		
									SQIEFALCR	1	N-Term(iTRAQ4plex); C8(Methylthio			49		
									VGDYFFGK	1	N-Term(iTRAQ4plex); K8(iTRAQ4plex			40		
264681533	CADM2	cell adhesion molecule 2 isoform 1	7.59	0	3	10		47.5								
									GKPLPEPVLWTK	6	N-Term(iTRAQ4plex); K2(iTRAQ4plex); K12(iTRAQ4plex)		4.74	57	3	599.71
									TFTVSSTLDFR	2	N-Term(iTRAQ4plex		4.32	48	2	709.38
									SDDGVAVICR	2	N-Term(iTRAQ4plex); C9(Methylthio		3.23	64	2	612.80
164419736	GRIA4	glutamate receptor 4 isoform 2 precursor	7.58	0	6	17		99.1								
									IQGLTGNVQFDHYGF	6	N-Term(iTRAQ4plex		5.68	97	3	616.99
									LQNILEQIVSVGK	3	N-Term(iTRAQ4plex); K13(iTRAQ4plex		4.73	71	2	865.03
									GVFAIFGLYDK	4	N-Term(iTRAQ4plex); K11(iTRAQ4plex		3.2	74	2	759.44
									GYSILQAIMEK	2	N-Term(iTRAQ4plex); K11(iTRAQ4plex			50		
									QLLEELDR	1	N-Term(iTRAQ4plex			41		
									FVIDCEIER	1	N-Term(iTRAQ4plex); C5(Methylthio			41		
310124901	LOC100507680	PREDICTED: HLA class I histocompatibility antigen, A-32 alpha chain-like isoform 11	7.57	0	2	3		35.4								
									DYIALNEDLR	2	N-Term(iTRAQ4plex		3.38	41	2	683.36
									FIAVGYVDDTQFVF	2	N-Term(iTRAQ4plex		3.38	70	2	887.47
310125176	LOC100507680	PREDICTED: HLA class I histocompatibility antigen, A-74 alpha chain-like isoform 10	7.57	0	2	3		35.4								
									DYIALNEDLR	2	N-Term(iTRAQ4plex		3.38	41	2	683.36
									FIAVGYVDDTQFVF	2	N-Term(iTRAQ4plex		3.38	70	2	887.47

310124835	LOC100507705	PREDICTED: class I histocompatibility antigen, Gogo-B*0103 alpha chain-like isoform 11	7.55	0	2	3		35.1								
									SWTAADTAAQITQF	2	N-Term(iTRAQ4plex		4.98	72	2	832.43
									DYIALNEDLR	2	N-Term(iTRAQ4plex		3.38	41	2	683.36
54607031	CADM2	cell adhesion molecule 2 isoform 3	7.55	0	3	10		47.5								
									GKPLPEPVLWTK	6	N-Term(iTRAQ4plex); K2(iTRAQ4plex); K12(iTRAQ4plex)		4.74	57	3	599.71
									TFTVSSTLDFR	2	N-Term(iTRAQ4plex		4.32	48	2	709.38
									SDDGVAVICR	2	N-Term(iTRAQ4plex); C9(Methylthio		3.23	64	2	612.80
113428306	LOC390956	PREDICTED: peptidyl-prolyl cis-trans isomerase A-like	7.53	0	1	2		20.4								
									IIPGFMCQGGDFTR	2	N-Term(iTRAQ4plex); C7(Methylthio		4.36	69	2	866.41
187607300	VCAN	versican core protein isoform 2 precursor	7.48	0	4	15		74.2								
									VSVPTHPEAVGDASLTVVI	9	N-Term(iTRAQ4plex); K19(iTRAQ4plex		7.91	94	3	732.08
									LATVGELQAAWR	2	N-Term(iTRAQ4plex		5.48	55	2	729.91
									LLASDAGLYR	2	N-Term(iTRAQ4plex		4.01	45	2	611.85
									FTFEEAAK	2	N-Term(iTRAQ4plex); K8(iTRAQ4plex		3.58	59	2	615.83
260099727	LDHA	L-lactate dehydrogenase A chain isoform 5	7.47	0	2	2		26.7								
									DQLIYNLLK	1	N-Term(iTRAQ4plex); K9(iTRAQ4plex			42		
									LVIITAGAR	1	N-Term(iTRAQ4plex			38		
164419734	GRIA4	glutamate receptor 4 isoform 1 precursor	7.43	0	6	17		100.7								
									IQGLTGNVQFDHYGF	6	N-Term(iTRAQ4plex		5.68	97	3	616.99
									LQNILEQIVSVGK	3	N-Term(iTRAQ4plex); K13(iTRAQ4plex		4.73	71	2	865.03
									GVFAIFGLYDK	4	N-Term(iTRAQ4plex); K11(iTRAQ4plex		3.2	74	2	759.44
									GYSILQAIMEK	2	N-Term(iTRAQ4plex); K11(iTRAQ4plex			50		
									QLLEELDR	1	N-Term(iTRAQ4plex			41		
									FVIDCEIER	1	N-Term(iTRAQ4plex); C5(Methylthio			41		
4759212	TBCA	tubulin-specific chaperone A	7.41	1	1	1	0.85	12.8								
									MMIPDCQR	1	N-Term(iTRAQ4plex); C6(Methylthio	0.85		34		
4503649	F9	coagulation factor IX preproprotein	7.38	3	3	3	0.919	51.7								
									EYTNIFLK	1	N-Term(iTRAQ4plex); K8(iTRAQ4plex	0.953		52		
									WIVTAAHCVETGVK	1	N-Term(iTRAQ4plex); C8(Methylthio); K14(iTRAQ4plex)	0.919		38		
310124899	LOC100507680	PREDICTED: HLA class I histocompatibility antigen, A-32 alpha chain-like isoform 6	7.36	0	2	3		36.8								
									DYIALNEDLR	2	N-Term(iTRAQ4plex		3.38	41	2	683.36
									FIAVGYVDDTQFVF	2	N-Term(iTRAQ4plex		3.38	70	2	887.47
310125065	LOC100507680	PREDICTED: HLA class I histocompatibility antigen, A-43 alpha chain-like isoform 9	7.36	0	2	3		37.1								
									DYIALNEDLR	2	N-Term(iTRAQ4plex		3.38	41	2	683.36
									FIAVGYVDDTQFVF	2	N-Term(iTRAQ4plex		3.38	70	2	887.47
310125103	LOC100507681	PREDICTED: HLA class I histocompatibility antigen, Cw-15 alpha chain-like isoform 5	7.36	0	2	3		37								
									APWVEQEGPEYWDR	2	N-Term(iTRAQ4plex		3.42	63	2	953.45
									DYIALNEDLR	2	N-Term(iTRAQ4plex		3.38	41	2	683.36
310125174	LOC100507680	PREDICTED: HLA class I histocompatibility antigen, A-74 alpha chain-like isoform 5	7.36	0	2	3		37								
									DYIALNEDLR	2	N-Term(iTRAQ4plex		3.38	41	2	683.36
									FIAVGYVDDTQFVF	2	N-Term(iTRAQ4plex		3.38	70	2	887.47
310128257	LOC100510327	PREDICTED: HLA class I histocompatibility antigen, A-30 alpha chain-like isoform 7	7.36	0	2	3		36.8								
									DYIALNEDLR	2	N-Term(iTRAQ4plex		3.38	41	2	683.36
									FIAVGYVDDTQFVF	2	N-Term(iTRAQ4plex		3.38	70	2	887.47
25777713	SKP1	S-phase kinase-associated protein 1 isoform b	7.36	1	1	2	0.987	18.6								
									NDFTEEEEAQVR	2	N-Term(iTRAQ4plex	0.987	3.15	65	2	805.88
24234732	SEMA6D	semaphorin-6D isoform 6 precursor	7.35	0	3	5		54.2								
									LSTLEYDGEEISGLAR	2	N-Term(iTRAQ4plex		4.63	81	2	948.99
									QTNVALFADGK	2	N-Term(iTRAQ4plex); K11(iTRAQ4plex		4.48	78	2	726.41
									LDFQLMLK	1	N-Term(iTRAQ4plex); K8(iTRAQ4plex			36		
312434019	PI16	peptidase inhibitor 16 precursor	7.34	3	3	24	0.947	49.4								
									AQVSPTASDMLHMF	5	N-Term(iTRAQ4plex); M13(Oxidation	0.947	6.51	79	3	568.62
									AQVSPTASDMLHMF	5	N-Term(iTRAQ4plex	1.026	6.43	68	3	563.28
									LMVELHNLYR	6	N-Term(iTRAQ4plex	0.997	4.34	53	2	716.40
									IGCGSHFCEK	6	N-Term(iTRAQ4plex); C3(Methylthio); C8(Methylthio); K10(iTRAQ4plex)	0.976	4.28	41	3	487.55
									LMVELHNLYR	2	N-Term(iTRAQ4plex); M2(Oxidation	0.849	4.05	44	3	483.27
30425438	OAF	out at first protein homolog precursor	7.33	2	2	4	0.821	30.7								
									SYSFDFYVPQR	2	N-Term(iTRAQ4plex	0.882	4.89	54	2	776.88
									ALILGELEK	2	N-Term(iTRAQ4plex); K9(iTRAQ4plex	0.765	3.29	62	2	637.41
145275213	F12	coagulation factor XII precursor	7.32	3	3	5	1.058	67.7								
									VVGGLVALR	3	N-Term(iTRAQ4plex	1.046	3.18	44	2	514.34
									LHEAFSPVSYQHDLALLF	1	N-Term(iTRAQ4plex	1.061		42		
									TTLSGAPCQPWASEATYF	1	N-Term(iTRAQ4plex); C8(Methylthio	1.161		36		
91208428	PTPRZ1	receptor-type tyrosine-protein phosphatase zeta precursor	7.3	15	15	60	1.038	254.4								
									EEEEGKDIEEGAIVNPGR	3	N-Term(iTRAQ4plex); K6(iTRAQ4plex	0.818	5.55	57	3	753.72
									FAVLYQQLDGEDQTK	6	N-Term(iTRAQ4plex); K15(iTRAQ4plex	1.235	5.34	79	2	1022.04
									TSLENTFIHNTGK	6	N-Term(iTRAQ4plex); K13(iTRAQ4plex	0.939	4.97	63	3	583.98
									VSGGVSEMVFK	4	N-Term(iTRAQ4plex); K11(iTRAQ4plex	1.176	4.69	47	2	714.39
									FSSFEEAVK	12	N-Term(iTRAQ4plex); K9(iTRAQ4plex	1.084	4.49	71	2	666.36
									TVEINLTNDYF	4	N-Term(iTRAQ4plex	1.169	4.48	56	2	741.39
									AIIDGVESVSR	9	N-Term(iTRAQ4plex	1.064	4.37	75	2	645.37
									VVYDTMIEK	4	N-Term(iTRAQ4plex); K9(iTRAQ4plex	1.225	4.19	57	2	693.38
									DIEEGAIVNPGR	2	N-Term(iTRAQ4plex	0.972	3.91	57	2	707.38
									QVFSSYTGK	2	N-Term(iTRAQ4plex); K9(iTRAQ4plex	1.246	3.47	61	2	652.86
									LVEEIGWSYTGALNQK	3	N-Term(iTRAQ4plex); K16(iTRAQ4plex	1.235	3.44	60	3	699.38
									ITFHWGK	1	N-Term(iTRAQ4plex); K7(iTRAQ4plex	0.929		42		
									YEPVLLK	2	N-Term(iTRAQ4plex); K7(iTRAQ4plex	0.876		41		
									CMSCSSYR	1	N-Term(iTRAQ4plex); C1(Methylthio); C4(Methylthio)	0.989		39		
									SDAGLVGGGEDGDTDDDGDDDDDDF	1	N-Term(iTRAQ4plex	0.669		39		
4507115	FSCN1	fascin	7.3	3	3	3	0.475	54.5								
									LVARPEPATGYTLEFF	1	N-Term(iTRAQ4plex	0.514		52		
									FLIVAHDDGR	1	N-Term(iTRAQ4plex	0.475		49		
									LINRPIIVFR	1	N-Term(iTRAQ4plex	0.434		29		
207028494	LDHA	L-lactate dehydrogenase A chain isoform 2	7.3	0	2	2		30.2								
									DQLIYNLLK	1	N-Term(iTRAQ4plex); K9(iTRAQ4plex			42		
									QVVESAYEVIK	1	N-Term(iTRAQ4plex); K11(iTRAQ4plex			35		

310125067	LOC100507680	PREDICTED: HLA class I histocompatibility antigen, A-43 alpha chain-like isoform 20	7.29	0	2	3		36.6								
									DYIALNEDLR	2	N-Term(iTRAQ4plex		3.38	41	2	683.36
									FIAVGYVDDTQFVF	2	N-Term(iTRAQ4plex		3.38	70	2	887.47
30795227	DTD1	D-tyrosyl-tRNA(Tyr) deacylase 1	7.18	1	1	3	0.766	23.4								
									ASVTVGGEQISAIGF	3	N-Term(iTRAQ4plex	0.766	4.72	87	2	794.94
34335272	PROCR	endothelial protein C receptor precursor	7.14	1	1	2	0.721	26.7								
									TQSGLQSYLLQFHGLVR	2	N-Term(iTRAQ4plex	0.721	5.16	83	3	697.72
291327491	FGFR1	basic fibroblast growth factor receptor 1 isoform 11 precursor	7.14	0	6	16		90.6								
									LRDDVQSINWLR	5	N-Term(iTRAQ4plex		5.08	62	2	829.96
									IGPDNLPYVQILK	3	N-Term(iTRAQ4plex); K13(iTRAQ4plex		4.03	40	2	879.53
									VYSDPQPHIQWLK	2	N-Term(iTRAQ4plex); K13(iTRAQ4plex		3.51	44	3	633.68
									EMEVLHLR	3	N-Term(iTRAQ4plex		3.51	39	2	585.82
									MPVAPYWTSPEK	2	N-Term(iTRAQ4plex); K12(iTRAQ4plex			57		
									DDVQSINWLR	1	N-Term(iTRAQ4plex			37		
310124911	LOC100507680	PREDICTED: HLA class I histocompatibility antigen, A-32 alpha chain-like isoform 4	7.12	0	2	3		37.9								
									DYIALNEDLR	2	N-Term(iTRAQ4plex		3.38	41	2	683.36
									FIAVGYVDDTQFVF	2	N-Term(iTRAQ4plex		3.38	70	2	887.47
310125079	LOC100507680	PREDICTED: HLA class I histocompatibility antigen, A-43 alpha chain-like isoform 15	7.12	0	2	3		38.2								
									DYIALNEDLR	2	N-Term(iTRAQ4plex		3.38	41	2	683.36
									FIAVGYVDDTQFVF	2	N-Term(iTRAQ4plex		3.38	70	2	887.47
310125186	LOC100507680	PREDICTED: HLA class I histocompatibility antigen, A-74 alpha chain-like isoform 3	7.12	0	2	3		38.1								
									DYIALNEDLR	2	N-Term(iTRAQ4plex		3.38	41	2	683.36
									FIAVGYVDDTQFVF	2	N-Term(iTRAQ4plex		3.38	70	2	887.47
4506569	ROBO1	roundabout homolog 1 isoform a precursor	7.09	1	8	19	0.967	180.8								
									IVEHPSDLIVSK	4	N-Term(iTRAQ4plex); K12(iTRAQ4plex		5.2	71	3	542.32
									TQDVLPTSQGVDHK	3	N-Term(iTRAQ4plex); K14(iTRAQ4plex		4.84	72	3	605.00
									RPSNLAVTVDDSAEFK	3	N-Term(iTRAQ4plex); K16(iTRAQ4plex		4.4	62	3	679.70
									TLEEAPSAPPQGVTVSK	2	N-Term(iTRAQ4plex); K17(iTRAQ4plex		4.13	66	2	1000.05
									SRPDEGVYVCVAF	2	N-Term(iTRAQ4plex); C10(Methylthio		3.97	43	3	547.60
									AEASATLTVQEPPHFVVKPF	2	N-Term(iTRAQ4plex); K18(iTRAQ4plex	0.967	3.83	68	3	822.46
									YVCVGTNMVGEF	2	N-Term(iTRAQ4plex); C3(Methylthio		3.36	42	2	759.36
291327489	FGFR1	basic fibroblast growth factor receptor 1 isoform 10 precursor	7.07	0	6	16		91.6								
									LRDDVQSINWLR	5	N-Term(iTRAQ4plex		5.08	62	2	829.96
									IGPDNLPYVQILK	3	N-Term(iTRAQ4plex); K13(iTRAQ4plex		4.03	40	2	879.53
									VYSDPQPHIQWLK	2	N-Term(iTRAQ4plex); K13(iTRAQ4plex		3.51	44	3	633.68
									EMEVLHLR	3	N-Term(iTRAQ4plex		3.51	39	2	585.82
									MPVAPYWTSPEK	2	N-Term(iTRAQ4plex); K12(iTRAQ4plex			57		
									DDVQSINWLR	1	N-Term(iTRAQ4plex			37		
148277022	SEPP1	selenoprotein P isoform 2	7.06	0	3	11		46.2								
									DMPASEDLQDLQK	6	N-Term(iTRAQ4plex); K13(iTRAQ4plex		4.02	75	2	889.46
									CINQLLCK	2	N-Term(iTRAQ4plex); C1(Methylthio); C7(Methylthio); K8(iTRAQ4plex)		3.21	49	2	657.84
									DDFLIYDR	3	N-Term(iTRAQ4plex			43		
105990522	FGFR1	basic fibroblast growth factor receptor 1 isoform 1 precursor	7.06	0	6	16		91.8								
									LRDDVQSINWLR	5	N-Term(iTRAQ4plex		5.08	62	2	829.96
									IGPDNLPYVQILK	3	N-Term(iTRAQ4plex); K13(iTRAQ4plex		4.03	40	2	879.53
									VYSDPQPHIQWLK	2	N-Term(iTRAQ4plex); K13(iTRAQ4plex		3.51	44	3	633.68
									EMEVLHLR	3	N-Term(iTRAQ4plex		3.51	39	2	585.82
									MPVAPYWTSPEK	2	N-Term(iTRAQ4plex); K12(iTRAQ4plex			57		
									DDVQSINWLR	1	N-Term(iTRAQ4plex			37		
4501945	ADM	ADM precursor	7.03	1	1	2	1.791	20.4								
									MSSSYPTGLADVK	2	N-Term(iTRAQ4plex); K13(iTRAQ4plex	1.791	3.71	63	2	822.43
124256496	HSPA1L	heat shock 70 kDa protein 1-like	7.02	0	3	7		70.3								
									ATAGDTHLGGEDFDNF	4	N-Term(iTRAQ4plex		5.18	77	3	607.28
									IINEPTAAAIAYGLDK	2	N-Term(iTRAQ4plex); K16(iTRAQ4plex		3.25	60	2	974.56
									TTPSYVAFTDTEF	1	N-Term(iTRAQ4plex			45		
222144231	FGFR2	fibroblast growth factor receptor 2 isoform 3 precursor	7.02	0	5	13		86.2								
									VYSDAQPHIQWIK	3	N-Term(iTRAQ4plex); K13(iTRAQ4plex		4.36	43	3	625.01
									TVLIGEYLQIK	2	N-Term(iTRAQ4plex); K11(iTRAQ4plex		4.34	58	2	782.98
									DAAVISWTK	4	N-Term(iTRAQ4plex); K9(iTRAQ4plex		4.25	66	2	639.87
									YGPDGLPYLK	2	N-Term(iTRAQ4plex); K10(iTRAQ4plex		3.92	48	2	705.90
									DSGLYACTASR	2	N-Term(iTRAQ4plex); C7(Methylthio		3.66	69	2	667.30
310125091	LOC100507681	PREDICTED: HLA class I histocompatibility antigen, Cw-15 alpha chain-like isoform 7	7	0	2	3		38.9								
									APWVEQEGPEYWDR	2	N-Term(iTRAQ4plex		3.42	63	2	953.45
									DYIALNEDLR	2	N-Term(iTRAQ4plex		3.38	41	2	683.36
310128267	LOC100510327	PREDICTED: HLA class I histocompatibility antigen, A-30 alpha chain-like isoform 14	6.96	0	2	2		39								
									DYIALNEDLR	2	N-Term(iTRAQ4plex		3.38	41	2	683.36
									FIAVGYVDDTQFVF	2	N-Term(iTRAQ4plex		3.38	70	2	887.47
4506455	RCN1	reticulocalbin-1 precursor	6.95	2	2	4	0.988	38.9								
									YIFDNVAK	2	N-Term(iTRAQ4plex); K8(iTRAQ4plex	0.893	3.43	41	2	629.36
									IDNDGDGFVTTEELK	2	N-Term(iTRAQ4plex); K15(iTRAQ4plex	1.092	3.23	69	2	970.99
4758250	EFNB2	ephrin-B2 precursor	6.91	2	2	3	0.815	36.9								
									FLPGQGLVLYPQIGDK	2	N-Term(iTRAQ4plex); K16(iTRAQ4plex	0.865	4.01	50	2	1017.09
									LDIICPK	1	N-Term(iTRAQ4plex); C5(Methylthio); K7(iTRAQ4plex)	0.769		53		
5729925	MIA	melanoma-derived growth regulatory protein precursor	6.87	1	1	1	0.746	14.5								
									GQVVYVFSK	1	N-Term(iTRAQ4plex); K9(iTRAQ4plex	0.746		48		
310125099	LOC100507681	PREDICTED: HLA class I histocompatibility antigen, Cw-15 alpha chain-like isoform 6	6.86	0	2	3		39.3								
									APWVEQEGPEYWDR	2	N-Term(iTRAQ4plex		3.42	63	2	953.45
									DYIALNEDLR	2	N-Term(iTRAQ4plex		3.38	41	2	683.36
40255005	PLXDC2	plexin domain-containing protein 2 precursor	6.81	3	3	12	0.889	59.5								
									VGLSDAFVVVHF	8	N-Term(iTRAQ4plex	0.889	4.82	77	2	721.92
									DLWVNIDQMEK	2	N-Term(iTRAQ4plex); K11(iTRAQ4plex	0.861	4.16	73	2	839.94
									QDWVDSGCPEESK	2	N-Term(iTRAQ4plex); C8(Methylthio); K13(iTRAQ4plex)	0.934	3.62	90	2	907.40
4502419	BLVRB	flavin reductase	6.8	1	1	2	0.571	22.1								

									NDLSPTTVMSEGAF	2	N-Term(iTRAQ4plex	0.571	4.75	74	2	811.41
315075311	CTSS	cathepsin S isoform 2 preproprotein	6.76	0	2	3		31.6								
									GIDSDASYPYK	2	N-Term(iTRAQ4plex); K11(iTRAQ4plex		4.6	47	2	752.38
									HPSFFLYR	1	N-Term(iTRAQ4plex			37		
104487295	PTPRS	receptor-type tyrosine-protein phosphatase S isoform 3 precursor	6.73	0	7	21		168.3								
									SETSITLSWSPPR	4	N-Term(iTRAQ4plex		5.79	74	2	802.92
									SPQGLGAFTPVVF	7	N-Term(iTRAQ4plex		5.33	61	2	736.92
									WMQGAEDLTPEDDMPVGF	2	N-Term(iTRAQ4plex		4.91	109	2	1096.00
									TQQGVPGQPMNLF	4	N-Term(iTRAQ4plex		4.34	50	2	785.40
									NVLELTDVK	2	N-Term(iTRAQ4plex); K9(iTRAQ4plex		4.2	45	2	659.90
									VLAFTSVGDGPLSDPIQVK	1	N-Term(iTRAQ4plex); K19(iTRAQ4plex			50		
									VYYTMEPEHPVGNWQK	1	N-Term(iTRAQ4plex); K16(iTRAQ4plex			46		
10190664	TWSG1	twisted gastrulation protein homolog 1 precursor	6.73	1	1	2	1.469	25								
									STVEELHEPIPSLFR	2	N-Term(iTRAQ4plex	1.469	4.61	35	3	633.34
4502519	CA4	carbonic anhydrase 4 precursor	6.73	2	2	4	0.919	35								
									YLGSLTTPTCDEK	2	N-Term(iTRAQ4plex); C10(Methylthio); K13(iTRAQ4plex)	0.931	4.69	45	2	881.44
									FFFSGYDK	2	N-Term(iTRAQ4plex); K8(iTRAQ4plex	0.908	3.51	41	2	649.84
104487611	PTPRS	receptor-type tyrosine-protein phosphatase S isoform 4 precursor	6.71	0	7	21		168.8								
									SETSITLSWSPPR	4	N-Term(iTRAQ4plex		5.79	74	2	802.92
									SPQGLGAFTPVVF	7	N-Term(iTRAQ4plex		5.33	61	2	736.92
									WMQGAEDLTPEDDMPVGF	2	N-Term(iTRAQ4plex		4.91	109	2	1096.00
									TQQGVPGQPMNLF	4	N-Term(iTRAQ4plex		4.34	50	2	785.40
									NVLELTDVK	2	N-Term(iTRAQ4plex); K9(iTRAQ4plex		4.2	45	2	659.90
									VLAFTSVGDGPLSDPIQVK	1	N-Term(iTRAQ4plex); K19(iTRAQ4plex			50		
									VYYTMEPEHPVGNWQK	1	N-Term(iTRAQ4plex); K16(iTRAQ4plex			46		
310125075	LOC100507680	PREDICTED: HLA class I histocompatibility antigen, A-43 alpha chain-like isoform 14, partial	6.69	0	2	3		40.3								
									DYIALNEDLR	2	N-Term(iTRAQ4plex		3.38	41	2	683.36
									FIAVGYVDDTQFVF	2	N-Term(iTRAQ4plex		3.38	70	2	887.47
310128265	LOC100510327	PREDICTED: HLA class I histocompatibility antigen, A-30 alpha chain-like isoform 13, partial	6.69	0	2	3		40								
									DYIALNEDLR	2	N-Term(iTRAQ4plex		3.38	41	2	683.36
									FIAVGYVDDTQFVF	2	N-Term(iTRAQ4plex		3.38	70	2	887.47
189011550	ASAH1	acid ceramidase isoform c	6.68	0	3	6		44								
									WYVVQTNYDR	2	N-Term(iTRAQ4plex		3.53	41	2	744.37
									HPFFLDDR	2	N-Term(iTRAQ4plex			42		
169214050	LOC390956	PREDICTED: peptidyl-prolyl cis-trans isomerase A-like	6.67	0	1	2		23								
									IIPGFMCQGGDFTR	2	N-Term(iTRAQ4plex); C7(Methylthio		4.36	69	2	866.41
308818204	ECM2	extracellular matrix protein 2 isoform 2 precursor	6.65	0	4	10		77.4								
									ELFLDHNDLK	4	N-Term(iTRAQ4plex); K10(iTRAQ4plex		4.78	74	3	511.28
									SYSSIVLKPQNIK	2	N-Term(iTRAQ4plex); K8(iTRAQ4plex); K13(iTRAQ4plex)		4.67	35	3	637.05
									KINVIVLR	3	N-Term(iTRAQ4plex); K1(iTRAQ4plex			52		
									SLLHLVLLGNQIER	1	N-Term(iTRAQ4plex			34		
17986001	HLA-B	major histocompatibility complex, class I, B precursor	6.63	0	2	3		40.4								
									SWTAADTAAQITQF	2	N-Term(iTRAQ4plex		4.98	72	2	832.43
									DYIALNEDLR	2	N-Term(iTRAQ4plex		3.38	41	2	683.36
226371613	SHBG	sex hormone-binding globulin isoform 4 precursor	6.62	0	2	4		31.7								
									QAEISASAPTSLR	3	N-Term(iTRAQ4plex		3.94	56	2	737.90
									DSWLDK	1	N-Term(iTRAQ4plex); K6(iTRAQ4plex			37		
27477039	ICOSLG	ICOS ligand precursor	6.62	2	2	6	1.07	33.3								
									GLYDVVSVLR	4	N-Term(iTRAQ4plex	1.04	4.46	58	2	632.87
									ALMSPAGMLR	2	N-Term(iTRAQ4plex	1.101	3.65	46	2	595.83
10834982	IGFBP5	insulin-like growth factor-binding protein 5 precursor	6.62	1	1	1	0.904	30.6								
									EHEEPTTSEMAEETYSPK	1	N-Term(iTRAQ4plex); M10(Oxidation); K18(iTRAQ4plex)	0.904		54		
310125113	LOC100507681	PREDICTED: HLA class I histocompatibility antigen, Cw-15 alpha chain-like isoform 4, partial	6.61	0	2	3		40.6								
									APWVEQEGPEYWDR	2	N-Term(iTRAQ4plex		3.42	63	2	953.45
									DYIALNEDLR	2	N-Term(iTRAQ4plex		3.38	41	2	683.36
57242798	SMPDL3B	acid sphingomyelinase-like phosphodiesterase 3b isoform 1	6.59	0	2	2		50.8								
									AGDMVYIVGHVPPGFFEK	1	N-Term(iTRAQ4plex); K18(iTRAQ4plex			37		
									LGEAAVLEIVEF	1	N-Term(iTRAQ4plex			35		
310124889	LOC100507680	PREDICTED: HLA class I histocompatibility antigen, A-32 alpha chain-like isoform 1	6.58	0	2	3		40.8								
									DYIALNEDLR	2	N-Term(iTRAQ4plex		3.38	41	2	683.36
									FIAVGYVDDTQFVF	2	N-Term(iTRAQ4plex		3.38	70	2	887.47
310125051	LOC100507680	PREDICTED: HLA class I histocompatibility antigen, A-43 alpha chain-like isoform 2	6.58	0	2	3		41								
									DYIALNEDLR	2	N-Term(iTRAQ4plex		3.38	41	2	683.36
									FIAVGYVDDTQFVF	2	N-Term(iTRAQ4plex		3.38	70	2	887.47
310125166	LOC100507680	PREDICTED: HLA class I histocompatibility antigen, A-74 alpha chain-like isoform 1	6.58	0	2	3		41								
									DYIALNEDLR	2	N-Term(iTRAQ4plex		3.38	41	2	683.36
									FIAVGYVDDTQFVF	2	N-Term(iTRAQ4plex		3.38	70	2	887.47
310128247	LOC100510327	PREDICTED: HLA class I histocompatibility antigen, A-30 alpha chain-like isoform 1	6.58	0	2	3		40.8								
									DYIALNEDLR	2	N-Term(iTRAQ4plex		3.38	41	2	683.36
									FIAVGYVDDTQFVF	2	N-Term(iTRAQ4plex		3.38	70	2	887.47
221316639	FGFR2	fibroblast growth factor receptor 2 isoform 1 precursor	6.58	0	5	13		92								
									VYSDAQPHIQWIK	3	N-Term(iTRAQ4plex); K13(iTRAQ4plex		4.36	43	3	625.01
									TVLIGEYLQIK	2	N-Term(iTRAQ4plex); K11(iTRAQ4plex		4.34	58	2	782.98
									DAAVISWTK	4	N-Term(iTRAQ4plex); K9(iTRAQ4plex		4.25	66	2	639.87

									YGPDGLPYLK	2	N-Term(iTRAQ4plex); K10(iTRAQ4plex		3.92	48	2	705.90
									DSGLYACTASR	2	N-Term(iTRAQ4plex); C7(Methylthio		3.66	69	2	667.30
4557797	NME1	nucleoside diphosphate kinase A isoform b	6.58	0	1	1		17.1								
									FMQASEDLLK	1	N-Term(iTRAQ4plex); K10(iTRAQ4plex			64		
24797067	HLA-A	HLA class I histocompatibility antigen, A-1 alpha chain precursor	6.58	0	2	2		40.8								
									DYIALNEDLR	2	N-Term(iTRAQ4plex		3.38	41	2	683.36
									FIAVGYVDDTQFVF	2	N-Term(iTRAQ4plex		3.38	70	2	887.47
310110685	LOC100508085	PREDICTED: putative V-set and immunoglobulin domain-containing protein 6-like, partial	6.57	0	1	2		15.3								
									VTISVDTSK	2	N-Term(iTRAQ4plex); K9(iTRAQ4plex		3.72	54	2	619.37
221316638	FGFR2	fibroblast growth factor receptor 2 isoform 2 precursor	6.57	0	5	13		92.1								
									VYSDAQPHIQWIK	3	N-Term(iTRAQ4plex); K13(iTRAQ4plex		4.36	43	3	625.01
									TVLIGEYLQIK	2	N-Term(iTRAQ4plex); K11(iTRAQ4plex		4.34	58	2	782.98
									DAAVISWTK	4	N-Term(iTRAQ4plex); K9(iTRAQ4plex		4.25	66	2	639.87
									YGPDGLPYLK	2	N-Term(iTRAQ4plex); K10(iTRAQ4plex		3.92	48	2	705.90
									DSGLYACTASR	2	N-Term(iTRAQ4plex); C7(Methylthio		3.66	69	2	667.30
260099725	LDHA	L-lactate dehydrogenase A chain isoform 4	6.57	0	2	2		30								
									DQLIYNLLK	1	N-Term(iTRAQ4plex); K9(iTRAQ4plex			42		
									LVIITAGAR	1	N-Term(iTRAQ4plex			38		
310125093	LOC100507681	PREDICTED: HLA class I histocompatibility antigen, Cw-15 alpha chain-like isoform 1	6.56	0	2	3		40.9								
									APWVEQEGPEYWDR	2	N-Term(iTRAQ4plex		3.42	63	2	953.45
									DYIALNEDLR	2	N-Term(iTRAQ4plex		3.38	41	2	683.36
148839346	SEZ6	seizure protein 6 homolog isoform 2	6.55	0	5	20		107.4								
									RPAYGDVTVTSLHPGGSAF	8	N-Term(iTRAQ4plex		6.61	61	4	522.03
									IGPGDVLTFYDGDDLTAF	2	N-Term(iTRAQ4plex		4.01	73	2	1035.02
									LHLHFEK	8	N-Term(iTRAQ4plex); K7(iTRAQ4plex		3.45	54	3	404.58
									FHCATGYQLK	1	N-Term(iTRAQ4plex); C3(Methylthio); K10(iTRAQ4plex)			39		
148839280	SEZ6	seizure protein 6 homolog isoform 1	6.54	0	5	20		107.4								
									RPAYGDVTVTSLHPGGSAF	8	N-Term(iTRAQ4plex		6.61	61	4	522.03
									IGPGDVLTFYDGDDLTAF	2	N-Term(iTRAQ4plex		4.01	73	2	1035.02
									LHLHFEK	8	N-Term(iTRAQ4plex); K7(iTRAQ4plex		3.45	54	3	404.58
									FHCATGYQLK	1	N-Term(iTRAQ4plex); C3(Methylthio); K10(iTRAQ4plex)			39		
310125061	LOC100507680	PREDICTED: HLA class I histocompatibility antigen, A-43 alpha chain-like isoform 4	6.52	0	2	3		41.2								
									DYIALNEDLR	2	N-Term(iTRAQ4plex		3.38	41	2	683.36
									FIAVGYVDDTQFVF	2	N-Term(iTRAQ4plex		3.38	70	2	887.47
310125089	LOC100507681	PREDICTED: HLA class I histocompatibility antigen, Cw-15 alpha chain-like isoform 8	6.52	0	2	3		41.4								
									APWVEQEGPEYWDR	2	N-Term(iTRAQ4plex		3.42	63	2	953.45
									DYIALNEDLR	2	N-Term(iTRAQ4plex		3.38	41	2	683.36
310128253	LOC100510327	PREDICTED: HLA class I histocompatibility antigen, A-30 alpha chain-like isoform 2	6.52	0	2	3		40.9								
									DYIALNEDLR	2	N-Term(iTRAQ4plex		3.38	41	2	683.36
									FIAVGYVDDTQFVF	2	N-Term(iTRAQ4plex		3.38	70	2	887.47
83641874	CPVL	probable serine carboxypeptidase CPVL precursor	6.51	3	3	6	0.768	54.1								
									QAGDFHQVIIR	2	N-Term(iTRAQ4plex	0.643	3.91	39	3	476.60
									SLMGMDWK	2	N-Term(iTRAQ4plex); K8(iTRAQ4plex	0.768	3.35	43	2	628.33
									CTEPEDQLYYVK	2	N-Term(iTRAQ4plex); C1(Methylthio); K12(iTRAQ4plex)	0.783	3.14	43	2	911.44
11034849	ENPP5	ectonucleotide pyrophosphatase/phosphodiesterase family member 5 precursor	6.5	3	3	9	1.049	54.6								
									SFSLDHMNIYDSK	4	N-Term(iTRAQ4plex); K13(iTRAQ4plex	1.189	5.57	67	3	615.64
									QVTNVFITK	2	N-Term(iTRAQ4plex); K9(iTRAQ4plex	0.815	4.23	59	2	669.41
									LGYLIQMLK	3	N-Term(iTRAQ4plex); K9(iTRAQ4plex	0.88	3.42	51	2	683.92
53692189	AEBP1	adipocyte enhancer-binding protein 1 precursor	6.48	4	5	9	0.842	130.8								
									GEDEDEVSEAQETPDHAIFF	2	N-Term(iTRAQ4plex	0.784	5.31	70	3	806.70
									YTAGIHGNEVLGF	2	N-Term(iTRAQ4plex	0.89	4.96	79	3	510.94
									EALLTFMEQVHR	2	N-Term(iTRAQ4plex	0.865	3.89	54	3	539.96
									IYAMEISDNPGEHELGEPEFR	2	N-Term(iTRAQ4plex	0.819	3.8	77	3	859.74
									FTGVITQGR	1	N-Term(iTRAQ4plex			36		
226371777	SHBG	sex hormone-binding globulin isoform 3 precursor	6.48	0	2	4		32.3								
									QAEISASAPTSLR	3	N-Term(iTRAQ4plex		3.94	56	2	737.90
									DSWLDK	1	N-Term(iTRAQ4plex); K6(iTRAQ4plex			37		
310124895	LOC100507680	PREDICTED: HLA class I histocompatibility antigen, A-32 alpha chain-like isoform 5	6.47	0	2	3		41.5								
									DYIALNEDLR	2	N-Term(iTRAQ4plex		3.38	41	2	683.36
									FIAVGYVDDTQFVF	2	N-Term(iTRAQ4plex		3.38	70	2	887.47
310125057	LOC100507680	PREDICTED: HLA class I histocompatibility antigen, A-43 alpha chain-like isoform 6	6.47	0	2	3		41.7								
									DYIALNEDLR	2	N-Term(iTRAQ4plex		3.38	41	2	683.36
									FIAVGYVDDTQFVF	2	N-Term(iTRAQ4plex		3.38	70	2	887.47
310125059	LOC100507680	PREDICTED: HLA class I histocompatibility antigen, A-43 alpha chain-like isoform 5	6.47	0	2	3		41.6								
									DYIALNEDLR	2	N-Term(iTRAQ4plex		3.38	41	2	683.36
									FIAVGYVDDTQFVF	2	N-Term(iTRAQ4plex		3.38	70	2	887.47
310125170	LOC100507680	PREDICTED: HLA class I histocompatibility antigen, A-74 alpha chain-like isoform 4	6.47	0	2	3		41.7								
									DYIALNEDLR	2	N-Term(iTRAQ4plex		3.38	41	2	683.36
									FIAVGYVDDTQFVF	2	N-Term(iTRAQ4plex		3.38	70	2	887.47
310128249	LOC100510327	PREDICTED: HLA class I histocompatibility antigen, A-30 alpha chain-like isoform 4	6.47	0	2	3		41.4								
									DYIALNEDLR	2	N-Term(iTRAQ4plex		3.38	41	2	683.36
									FIAVGYVDDTQFVF	2	N-Term(iTRAQ4plex		3.38	70	2	887.47
310128251	LOC100510327	PREDICTED: HLA class I histocompatibility antigen, A-30 alpha chain-like isoform 3	6.47	0	2	3		41.3								
									DYIALNEDLR	2	N-Term(iTRAQ4plex		3.38	41	2	683.36
									FIAVGYVDDTQFVF	2	N-Term(iTRAQ4plex		3.38	70	2	887.47

47078235	GFRA2	GDNF family receptor alpha-2 isoform a preproprotein	6.47	0	3	8		51.5								
									QTILPSCSYEDK	2	N-Term(iTRAQ4plex); C7(Methylthio); K12(iTRAQ4plex)		4.67	77	2	859.43
									DFTENPCLR	4	N-Term(iTRAQ4plex); C7(Methylthio		3.9	34	2	642.79
									SSYISICNR	2	N-Term(iTRAQ4plex); C7(Methylthio		3.42	47	2	616.80
48255943	CD44	CD44 antigen isoform 5 precursor	6.47	0	1	12		15.6								
									FAGVFHVEK	12	N-Term(iTRAQ4plex); K9(iTRAQ4plex		3.55	79	2	661.38
310125095	LOC100507681	PREDICTED: HLA class I histocompatibility antigen, Cw-15 alpha chain-like isoform 3	6.45	0	2	3		41.5								
									APWVEQEGPEYWDR	2	N-Term(iTRAQ4plex		3.42	63	2	953.45
									DYIALNEDLR	2	N-Term(iTRAQ4plex		3.38	41	2	683.36
310125097	LOC100507681	PREDICTED: HLA class I histocompatibility antigen, Cw-15 alpha chain-like isoform 2	6.45	0	2	3		41.6								
									APWVEQEGPEYWDR	2	N-Term(iTRAQ4plex		3.42	63	2	953.45
									DYIALNEDLR	2	N-Term(iTRAQ4plex		3.38	41	2	683.36
4557543	ECM2	extracellular matrix protein 2 isoform 1 precursor	6.44	0	4	10		79.7								
									ELFLDHNDLK	4	N-Term(iTRAQ4plex); K10(iTRAQ4plex		4.78	74	3	511.28
									SYSSIVLKPQNIK	2	N-Term(iTRAQ4plex); K8(iTRAQ4plex); K13(iTRAQ4plex)		4.67	35	3	637.05
									KINVIVLR	3	N-Term(iTRAQ4plex); K1(iTRAQ4plex			52		
									SLLHLVLLGNQIER	1	N-Term(iTRAQ4plex			34		
150456469	EPHA10	ephrin type-A receptor 10 isoform 2	6.44	0	1	2		32.1								
									KIDTIAADESFTQGDLGEF	2	N-Term(iTRAQ4plex); K1(iTRAQ4plex		4.58	94	3	785.41
29171717	GPLD1	phosphatidylinositol-glycan-specific phospholipase D isoform 1 precursor	6.43	3	4	9	1.127	92.3								
									IADVTSGLIGGEDGR	2	N-Term(iTRAQ4plex	1.027	4.53	63	2	802.42
									AQYVLISPEASSR	3	N-Term(iTRAQ4plex	1.13	3.84	55	2	782.93
									VAFLTVTLHQGGATF	2	N-Term(iTRAQ4plex	1.16	3.39	49	3	572.33
									ALEFLQLHNGR	2	N-Term(iTRAQ4plex		3.36	46	3	481.27
310124887	LOC100507680	PREDICTED: HLA class I histocompatibility antigen, A-32 alpha chain-like isoform 8	6.42	0	2	3		41.8								
									DYIALNEDLR	2	N-Term(iTRAQ4plex		3.38	41	2	683.36
									FIAVGYVDDTQFVF	2	N-Term(iTRAQ4plex		3.38	70	2	887.47
310125043	LOC100507680	PREDICTED: HLA class I histocompatibility antigen, A-43 alpha chain-like isoform 12	6.42	0	2	3		42.1								
									DYIALNEDLR	2	N-Term(iTRAQ4plex		3.38	41	2	683.36
									FIAVGYVDDTQFVF	2	N-Term(iTRAQ4plex		3.38	70	2	887.47
310125164	LOC100507680	PREDICTED: HLA class I histocompatibility antigen, A-74 alpha chain-like isoform 7	6.42	0	2	3		42.1								
									DYIALNEDLR	2	N-Term(iTRAQ4plex		3.38	41	2	683.36
									FIAVGYVDDTQFVF	2	N-Term(iTRAQ4plex		3.38	70	2	887.47
310128241	LOC100510327	PREDICTED: HLA class I histocompatibility antigen, A-30 alpha chain-like isoform 9	6.42	0	2	3		41.8								
									DYIALNEDLR	2	N-Term(iTRAQ4plex		3.38	41	2	683.36
									FIAVGYVDDTQFVF	2	N-Term(iTRAQ4plex		3.38	70	2	887.47
41327734	EGFR	epidermal growth factor receptor isoform c precursor	6.42	0	2	3		44.6								
									NLQEILHGAVR	2	N-Term(iTRAQ4plex		4.03	47	3	465.27
									ACGADSYEMEEDGVF	1	N-Term(iTRAQ4plex); C2(Methylthio			49		
4505257	MSN	moesin	6.41	3	4	9	0.884	67.8								
									KESPLLFK	5	N-Term(iTRAQ4plex); K1(iTRAQ4plex); K8(iTRAQ4plex)	0.884	4.15	44	3	465.29
									LFFLQVK	2	N-Term(iTRAQ4plex); K7(iTRAQ4plex			49		
									TQEQLALEMAELTAF	1	N-Term(iTRAQ4plex	0.882		46		
									GFSTWLK	1	N-Term(iTRAQ4plex); K7(iTRAQ4plex	1.35		37		
144953895	NID2	nidogen-2 precursor	6.4	6	7	16	0.866	151.2								
									ESYNVQLQLPAR	4	N-Term(iTRAQ4plex	0.896	5.13	76	2	781.43
									GNLYWTDWNR	2	N-Term(iTRAQ4plex		4.08	43	2	734.86
									SEGPYFSLTSTEQSVK	2	N-Term(iTRAQ4plex); K16(iTRAQ4plex	0.866	3.95	76	2	1024.53
									HPSFPTTQQLNVDF	2	N-Term(iTRAQ4plex	0.856	3.95	49	3	595.31
									IESALLDGSER	2	N-Term(iTRAQ4plex	0.993	3.42	48	2	667.36
									HSGQFTDEYLPEQR	2	N-Term(iTRAQ4plex	0.942	3.39	37	3	617.63
									LANPLHFYEAR	2	N-Term(iTRAQ4plex	0.824		44		
104487297	PTPRS	receptor-type tyrosine-protein phosphatase S isoform 2 precursor	6.39	0	8	23		212.4								
									ITTVAHTEVGPGPESSPVVVI	2	N-Term(iTRAQ4plex		7.75	78	3	759.42
									SETSITLSWSPPR	4	N-Term(iTRAQ4plex		5.79	74	2	802.92
									SPQGLGAFTPVVR	7	N-Term(iTRAQ4plex		5.33	61	2	736.92
									WMQGAEDLTPEDDMPVGR	2	N-Term(iTRAQ4plex		4.91	109	2	1096.00
									TQQGVPGQPMNLF	4	N-Term(iTRAQ4plex		4.34	50	2	785.40
									NVLELTDVK	2	N-Term(iTRAQ4plex); K9(iTRAQ4plex		4.2	45	2	659.90
									VLAFTSVGDGPLSDPIQVK	1	N-Term(iTRAQ4plex); K19(iTRAQ4plex			50		
									VYYTMEPEHPVGNWQK	1	N-Term(iTRAQ4plex); K16(iTRAQ4plex			46		
63055057	ACTBL2	beta-actin-like protein 2	6.38	0	2	8		42								
									DLTDYLMK	5	N-Term(iTRAQ4plex); K8(iTRAQ4plex		3.98	48	2	643.85
									SYELPDGQVITIGNER	3	N-Term(iTRAQ4plex		3.27	49	2	968.00
310125045	LOC100507680	PREDICTED: HLA class I histocompatibility antigen, A-43 alpha chain-like isoform 11	6.38	0	2	3		42.2								
									DYIALNEDLR	2	N-Term(iTRAQ4plex		3.38	41	2	683.36
									FIAVGYVDDTQFVF	2	N-Term(iTRAQ4plex		3.38	70	2	887.47
310128243	LOC100510327	PREDICTED: HLA class I histocompatibility antigen, A-30 alpha chain-like isoform 8	6.38	0	2	3		41.9								
									DYIALNEDLR	2	N-Term(iTRAQ4plex		3.38	41	2	683.36
									FIAVGYVDDTQFVF	2	N-Term(iTRAQ4plex		3.38	70	2	887.47
295054188	HABP2	hyaluronan-binding protein 2 isoform 2	6.37	0	3	4		59.8								
									VVLGDQDLK	2	N-Term(iTRAQ4plex); K9(iTRAQ4plex		3.98	62	2	637.87
									DEIPHNDIALLK	1	N-Term(iTRAQ4plex); K12(iTRAQ4plex			58		
21624607	COTL1	coactosin-like protein	6.34	2	2	3	0.974	15.9								
									KELEEDFIK	2	N-Term(iTRAQ4plex); K1(iTRAQ4plex); K9(iTRAQ4plex)	0.95	3.63	38	3	528.30
4758440	GMFG	glia maturation factor gamma	6.34	0	1	4		16.8								
									LVQTAELTK	4	N-Term(iTRAQ4plex); K9(iTRAQ4plex		3.54	61	2	645.90
4758442	GMFB	glia maturation factor beta	6.34	0	1	4		16.7								
									LVQTAELTK	4	N-Term(iTRAQ4plex); K9(iTRAQ4plex		3.54	61	2	645.90
6005864	RNF13	E3 ubiquitin-protein ligase RNF13	6.3	1	1	2	0.903	42.8								
									AAIVHNVDSDDLISMGSNDIEVLI	2	N-Term(iTRAQ4plex); K24(iTRAQ4plex	0.903	3.71	89	3	948.49

48255939	CD44	CD44 antigen isoform 3 precursor	6.29	0	3	72		53.4								
									YGFIEGHVVIPR	47	N-Term(iTRAQ4plex		4.96	83	2	765.93
									ALSIGFETCR	13	N-Term(iTRAQ4plex); C9(Methylthio		4.45	53	2	643.82
									FAGVFHVEK	12	N-Term(iTRAQ4plex); K9(iTRAQ4plex		3.55	79	2	661.38
291327495	FGFR1	basic fibroblast growth factor receptor 1 isoform 3 precursor	6.28	0	4	10		82.1								
									IGPDNLPYVQILK	3	N-Term(iTRAQ4plex); K13(iTRAQ4plex		4.03	40	2	879.53
									VYSDPQPHIQWLK	2	N-Term(iTRAQ4plex); K13(iTRAQ4plex		3.51	44	3	633.68
									EMEVLHLR	3	N-Term(iTRAQ4plex		3.51	39	2	585.82
									MPVAPYWTSPEK	2	N-Term(iTRAQ4plex); K12(iTRAQ4plex			57		
21361559	VSNL1	visinin-like protein 1	6.28	1	1	1	1.022	22.1								
									NKDDQITLDEFK	1	N-Term(iTRAQ4plex); K2(iTRAQ4plex); K12(iTRAQ4plex)	1.022		52		
310125047	LOC100507680	PREDICTED: HLA class I histocompatibility antigen, A-43 alpha chain-like isoform 7	6.27	0	2	3		43.3								
									DYIALNEDLR	2	N-Term(iTRAQ4plex		3.38	41	2	683.36
									FIAVGYVDDTQFVR	2	N-Term(iTRAQ4plex		3.38	70	2	887.47
7669492	GAPDH	glyceraldehyde-3-phosphate dehydrogenase	6.27	1	1	2	0.98	36								
									VIISAPSADAPMFVMGVNHEI	2	N-Term(iTRAQ4plex); K21(iTRAQ4plex	0.98	5.09	65	3	834.45
104487006	PTPRS	receptor-type tyrosine-protein phosphatase S isoform 1 precursor	6.26	0	8	23		216.9								
									ITTVAHTEVGPGPESSPVVVI	2	N-Term(iTRAQ4plex		7.75	78	3	759.42
									SETSITLSWSPPR	4	N-Term(iTRAQ4plex		5.79	74	2	802.92
									SPQGLGAFTPVVR	7	N-Term(iTRAQ4plex		5.33	61	2	736.92
									WMQGAEDLTPEDDMPVGR	2	N-Term(iTRAQ4plex		4.91	109	2	1096.00
									TQQGVPGQPMNLR	4	N-Term(iTRAQ4plex		4.34	50	2	785.40
									NVLELTDVK	2	N-Term(iTRAQ4plex); K9(iTRAQ4plex		4.2	45	2	659.90
									VLAFTSVGDGPLSDPIQVK	1	N-Term(iTRAQ4plex); K19(iTRAQ4plex			50		
									VYYTMEPEHPVGNWQK	1	N-Term(iTRAQ4plex); K16(iTRAQ4plex			46		
224994173	ROBO1	roundabout homolog 1 isoform c	6.25	0	7	17		169.6								
									IVEHPSDLIVSK	4	N-Term(iTRAQ4plex); K12(iTRAQ4plex		5.2	71	3	542.32
									TQDVLPTSQGVDHK	3	N-Term(iTRAQ4plex); K14(iTRAQ4plex		4.84	72	3	605.00
									RPSNLAVTVDDSAEFK	3	N-Term(iTRAQ4plex); K16(iTRAQ4plex		4.4	62	3	679.70
									TLEEAPSAPPQGVTVSK	2	N-Term(iTRAQ4plex); K17(iTRAQ4plex		4.13	66	2	1000.05
									SRPDEGVYVCVAR	2	N-Term(iTRAQ4plex); C10(Methylthio		3.97	43	3	547.60
									YVCVGTNMVGER	2	N-Term(iTRAQ4plex); C3(Methylthio		3.36	42	2	759.36
29171719	GPLD1	phosphatidylinositol-glycan-specific phospholipase D isoform 2 precursor	6.25	0	1	2		19.9								
									ALEFLQLHNGR	2	N-Term(iTRAQ4plex		3.36	46	3	481.27
4826940	PRCP	lysosomal Pro-X carboxypeptidase isoform 1 preproprotein	6.25	1	3	10	0.915	55.8								
									VDHFGFNTVK	6	N-Term(iTRAQ4plex); K10(iTRAQ4plex	0.915	4.56	67	3	484.60
									AMLVFAEHR	2	N-Term(iTRAQ4plex		3.42	48	3	406.56
									NALDPMSVLLAR	2	N-Term(iTRAQ4plex		3.36	55	2	722.41
305632839	SPINK6	serine protease inhibitor Kazal-type 6 precursor	6.25	0	1	1		8.6								
									CAFCK	1	N-Term(iTRAQ4plex); C1(Methylthio); C4(Methylthio); K5(iTRAQ4plex)			27		
310125049	LOC100507680	PREDICTED: HLA class I histocompatibility antigen, A-43 alpha chain-like isoform 8	6.23	0	2	3		43.3								
									DYIALNEDLR	2	N-Term(iTRAQ4plex		3.38	41	2	683.36
									FIAVGYVDDTQFVR	2	N-Term(iTRAQ4plex		3.38	70	2	887.47
310128245	LOC100510327	PREDICTED: HLA class I histocompatibility antigen, A-30 alpha chain-like isoform 5	6.23	0	2	3		43.1								
									DYIALNEDLR	2	N-Term(iTRAQ4plex		3.38	41	2	683.36
									FIAVGYVDDTQFVR	2	N-Term(iTRAQ4plex		3.38	70	2	887.47
4503571	ENO1	alpha-enolase isoform 1	6.22	1	2	3	0.759	47.1								
									GNPTVEVDLFTSK	2	N-Term(iTRAQ4plex); K13(iTRAQ4plex	0.759	4.67	79	2	847.97
41327732	EGFR	epidermal growth factor receptor isoform b precursor	6.21	0	3	5		69.2								
									NLQEILHGAVR	2	N-Term(iTRAQ4plex		4.03	47	3	465.27
									EISDGDVIISGNK	2	N-Term(iTRAQ4plex); K13(iTRAQ4plex		3.52	67	2	817.95
									ACGADSYEMEEDGVR	1	N-Term(iTRAQ4plex); C2(Methylthio			49		
6912586	PGLS	6-phosphogluconolactonase	6.2	1	1	1	0.65	27.5								
									ELPAAVAPAGPASLAR	1	N-Term(iTRAQ4plex	0.65		56		
256985100	TMED7-TICAM2	TRAM adaptor with GOLD domain isoform 1	6.19	0	2	3		46.1								
									QCFYEDIAQGTK	2	N-Term(iTRAQ4plex); C2(Methylthio); K12(iTRAQ4plex)		4.15	98	2	868.91
									FCFSNEFSTFTHK	1	N-Term(iTRAQ4plex); C2(Methylthio); K13(iTRAQ4plex)			50		
5729877	HSPA8	heat shock cognate 71 kDa protein isoform 1	6.19	1	3	5	0.976	70.9								
									NSLESYAFNMK	2	N-Term(iTRAQ4plex); K11(iTRAQ4plex	0.976	3.44	86	2	796.40
									IINEPTAAAIAYGLDK	2	N-Term(iTRAQ4plex); K16(iTRAQ4plex		3.25	60	2	974.56
									TTPSYVAFTDTER	1	N-Term(iTRAQ4plex			45		
228480221	FHL1	four and a half LIM domains protein 1 isoform 4	6.19	0	1	2		22								
									AIVAGDQNVEYK	2	N-Term(iTRAQ4plex); K12(iTRAQ4plex		4.31	51	2	797.94
224493972	TXNDC5	thioredoxin domain-containing protein 5 isoform 3	6.17	0	2	2		36.2								
									EFPGLAGVK	1	N-Term(iTRAQ4plex); K9(iTRAQ4plex			57		
									TLAPTWEELSK	1	N-Term(iTRAQ4plex); K11(iTRAQ4plex			43		
4503625	F10	coagulation factor X preproprotein	6.15	2	2	5	1.88	54.7								
									NTEQEEGGEAVHEVEVVIK	3	N-Term(iTRAQ4plex); K19(iTRAQ4plex	1.923	5.03	69	3	795.41
									DWAESTLMTQK	2	N-Term(iTRAQ4plex); K11(iTRAQ4plex	1.561	4.73	75	2	799.41
4757760	ADIPOQ	adiponectin precursor	6.15	1	1	2	1.169	26.4								
									IFYNQQNHYDGSTGK	2	N-Term(iTRAQ4plex); K15(iTRAQ4plex	1.169	4.8	47	3	687.34
4503271	DCN	decorin isoform a preproprotein	6.13	2	2	5	0.865	39.7								
									NLHALILVNNK	3	N-Term(iTRAQ4plex); K11(iTRAQ4plex	0.868	5.39	73	2	768.98
									VSPGAFTPLVK	2	N-Term(iTRAQ4plex); K11(iTRAQ4plex	0.865	3.98	51	2	702.43
4503117	CSTB	cystatin-B	6.12	1	1	2	1.192	11.1								
									KFPVFK	2	N-Term(iTRAQ4plex); K1(iTRAQ4plex); K6(iTRAQ4plex)	1.192	3.42	38	3	399.93
4758502	HABP2	hyaluronan-binding protein 2 isoform 1 preproprotein	6.07	0	3	4		62.6								
									VVLGDQDLK	2	N-Term(iTRAQ4plex); K9(iTRAQ4plex		3.98	62	2	637.87
									DEIPHNDIALLK	1	N-Term(iTRAQ4plex); K12(iTRAQ4plex			58		
109148552	KRT3	keratin, type II cytoskeletal 3	6.05	0	4	8		64.4								
									TAAENEFVTLK	2	N-Term(iTRAQ4plex); K11(iTRAQ4plex		3.68	44	2	755.92
									FASFIDK	4	N-Term(iTRAQ4plex); K7(iTRAQ4plex		3.19	53	2	558.31
294345407	ROBO1	roundabout homolog 1 isoform b	6.04	0	7	17		175.7								

									IVEHPSDLIVSK	4	N-Term(iTRAQ4plex); K12(iTRAQ4plex		5.2	71	3	542.32
									TQDVLPTSQGVDHK	3	N-Term(iTRAQ4plex); K14(iTRAQ4plex		4.84	72	3	605.00
									RPSNLAVTVDDSAEFK	3	N-Term(iTRAQ4plex); K16(iTRAQ4plex		4.4	62	3	679.70
									TLEEAPSAPPQGVTVSK	2	N-Term(iTRAQ4plex); K17(iTRAQ4plex		4.13	66	2	1000.05
									SRPDEGVYVCVAR	2	N-Term(iTRAQ4plex); C10(Methylthio		3.97	43	3	547.60
									YVCVGTNMVGER	2	N-Term(iTRAQ4plex); C3(Methylthio		3.36	42	2	759.36
239748171	LOC642132	PREDICTED: roundabout homolog 1-like isoform 2	6.01	0	7	17		176.6								
									IVEHPSDLIVSK	4	N-Term(iTRAQ4plex); K12(iTRAQ4plex		5.2	71	3	542.32
									TQDVLPTSQGVDHK	3	N-Term(iTRAQ4plex); K14(iTRAQ4plex		4.84	72	3	605.00
									RPSNLAVTVDDSAEFK	3	N-Term(iTRAQ4plex); K16(iTRAQ4plex		4.4	62	3	679.70
									TLEEAPSAPPQGVTVSK	2	N-Term(iTRAQ4plex); K17(iTRAQ4plex		4.13	66	2	1000.05
									SRPDEGVYVCVAR	2	N-Term(iTRAQ4plex); C10(Methylthio		3.97	43	3	547.60
									YVCVGTNMVGER	2	N-Term(iTRAQ4plex); C3(Methylthio		3.36	42	2	759.36
9910362	SEMA3G	semaphorin-3G precursor	6.01	3	4	13	0.808	86.6								
									THLAQQLHQIVVDR	6	N-Term(iTRAQ4plex	0.82	6.32	82	3	601.35
									GEHVLHLEPGSVESGR	2	N-Term(iTRAQ4plex	0.795	3.97	46	2	923.98
									EVLWPPQPGQR	2	N-Term(iTRAQ4plex	0.796	3.19	37	2	725.90
									WSTFLK	3	N-Term(iTRAQ4plex); K6(iTRAQ4plex			42		
41872567	NRP2	neuropilin-2 isoform 5 precursor	5.99	0	4	9		101.3								
									IANEQISASSTYSDGR	2	N-Term(iTRAQ4plex		5.52	103	2	921.95
									VFQANNDATEVVLNK	2	N-Term(iTRAQ4plex); K15(iTRAQ4plex		3.93	77	2	975.54
									LISPPVHLPR	4	N-Term(iTRAQ4plex		3.47	54	3	424.94
									IVLNFNPHFEIEK	1	N-Term(iTRAQ4plex); K13(iTRAQ4plex			50		
4507677	HSP90B1	endoplasmin precursor	5.98	3	4	6	1.015	92.4								
									ELISNASDALDK	1	N-Term(iTRAQ4plex); K12(iTRAQ4plex			60		
									NLLHVTDTGVGMTR	3	N-Term(iTRAQ4plex	1.031		51		
									LIINSLYK	1	N-Term(iTRAQ4plex); K8(iTRAQ4plex	1.004		51		
18104944	CBLN4	cerebellin-4 precursor	5.97	1	1	2	0.694	21.8								
									GSSSSPLGISVR	2	N-Term(iTRAQ4plex	0.694	4.91	72	2	645.86
41872544	NRP2	neuropilin-2 isoform 4 precursor	5.96	0	4	9		101.9								
									IANEQISASSTYSDGR	2	N-Term(iTRAQ4plex		5.52	103	2	921.95
									VFQANNDATEVVLNK	2	N-Term(iTRAQ4plex); K15(iTRAQ4plex		3.93	77	2	975.54
									LISPPVHLPR	4	N-Term(iTRAQ4plex		3.47	54	3	424.94
									IVLNFNPHFEIEK	1	N-Term(iTRAQ4plex); K13(iTRAQ4plex			50		
41872572	NRP2	neuropilin-2 isoform 3 precursor	5.94	0	4	9		102.1								
									IANEQISASSTYSDGR	2	N-Term(iTRAQ4plex		5.52	103	2	921.95
									VFQANNDATEVVLNK	2	N-Term(iTRAQ4plex); K15(iTRAQ4plex		3.93	77	2	975.54
									LISPPVHLPR	4	N-Term(iTRAQ4plex		3.47	54	3	424.94
									IVLNFNPHFEIEK	1	N-Term(iTRAQ4plex); K13(iTRAQ4plex			50		
119466532	LAMA2	laminin subunit alpha-2 isoform b precursor	5.9	0	14	23		343.3								
									GTYFDGTGFAK	2	N-Term(iTRAQ4plex); K11(iTRAQ4plex		4.94	62	2	726.38
									INHADFATVQLR	2	N-Term(iTRAQ4plex		4.89	55	3	510.28
									EGNDILDEANR	2	N-Term(iTRAQ4plex		4.5	58	2	695.34
									MDGMGIEMIDEK	2	N-Term(iTRAQ4plex); K12(iTRAQ4plex		3.95	83	2	828.90
									NSHIAIAFDDTK	3	N-Term(iTRAQ4plex); K12(iTRAQ4plex		3.95	46	3	540.63
									EESFTIHGTHFPVR	2	N-Term(iTRAQ4plex		3.53	61	2	900.97
									TPYNILSSPDYVGVTK	2	N-Term(iTRAQ4plex); K16(iTRAQ4plex		3.25	34	2	1021.56
									NTWWQSPSIK	2	N-Term(iTRAQ4plex); K10(iTRAQ4plex		3.14	74	2	767.91
									AEQTILPLVDEALQHTTTK	1	N-Term(iTRAQ4plex); K19(iTRAQ4plex			49		
									DVLAQITELHQNLDGLK	1	N-Term(iTRAQ4plex); K17(iTRAQ4plex			49		
									EDLHLEPFYWK	1	N-Term(iTRAQ4plex); K11(iTRAQ4plex			46		
									LFVGGAPPEFQPSPLR	1	N-Term(iTRAQ4plex			40		
									LMFHVDNGAGR	1	N-Term(iTRAQ4plex			38		
28559088	LAMA2	laminin subunit alpha-2 isoform a precursor	5.89	0	14	23		343.7								
									GTYFDGTGFAK	2	N-Term(iTRAQ4plex); K11(iTRAQ4plex		4.94	62	2	726.38
									INHADFATVQLR	2	N-Term(iTRAQ4plex		4.89	55	3	510.28
									EGNDILDEANR	2	N-Term(iTRAQ4plex		4.5	58	2	695.34
									MDGMGIEMIDEK	2	N-Term(iTRAQ4plex); K12(iTRAQ4plex		3.95	83	2	828.90
									NSHIAIAFDDTK	3	N-Term(iTRAQ4plex); K12(iTRAQ4plex		3.95	46	3	540.63
									EESFTIHGTHFPVR	2	N-Term(iTRAQ4plex		3.53	61	2	900.97
									TPYNILSSPDYVGVTK	2	N-Term(iTRAQ4plex); K16(iTRAQ4plex		3.25	34	2	1021.56
									NTWWQSPSIK	2	N-Term(iTRAQ4plex); K10(iTRAQ4plex		3.14	74	2	767.91
									AEQTILPLVDEALQHTTTK	1	N-Term(iTRAQ4plex); K19(iTRAQ4plex			49		
									DVLAQITELHQNLDGLK	1	N-Term(iTRAQ4plex); K17(iTRAQ4plex			49		
									EDLHLEPFYWK	1	N-Term(iTRAQ4plex); K11(iTRAQ4plex			46		
									LFVGGAPPEFQPSPLR	1	N-Term(iTRAQ4plex			40		
									LMFHVDNGAGR	1	N-Term(iTRAQ4plex			38		
24234686	HSPA8	heat shock cognate 71 kDa protein isoform 2	5.88	0	2	3		53.5								
									IINEPTAAAIAYGLDK	2	N-Term(iTRAQ4plex); K16(iTRAQ4plex		3.25	60	2	974.56
									TTPSYVAFTDTER	1	N-Term(iTRAQ4plex			45		
24234744	SEMA6D	semaphorin-6D isoform 5 precursor	5.86	0	3	5		67.5								
									LSTLEYDGEEISGLAR	2	N-Term(iTRAQ4plex		4.63	81	2	948.99
									QTNVALFADGK	2	N-Term(iTRAQ4plex); K11(iTRAQ4plex		4.48	78	2	726.41
									LDFQLMLK	1	N-Term(iTRAQ4plex); K8(iTRAQ4plex			36		
27754771	PCDH1	protocadherin-1 isoform 1 precursor	5.85	0	5	16		114.7								
									NTGLITVQGPVDR	2	N-Term(iTRAQ4plex		4.67	74	2	757.43
									YFLQTTTPLDYEK	2	N-Term(iTRAQ4plex); K13(iTRAQ4plex		4.65	75	2	954.01
									QPQLIVMGNLDR	4	N-Term(iTRAQ4plex		4.56	58	2	764.43
									TGDIFTTETSIDR	6	N-Term(iTRAQ4plex		4.14	80	2	800.40
									EQQSTYTFQLK	2	N-Term(iTRAQ4plex); K11(iTRAQ4plex		4.06	51	2	830.94
41872533	NRP2	neuropilin-2 isoform 2 precursor	5.83	0	4	9		104.2								
									IANEQISASSTYSDGR	2	N-Term(iTRAQ4plex		5.52	103	2	921.95
									VFQANNDATEVVLNK	2	N-Term(iTRAQ4plex); K15(iTRAQ4plex		3.93	77	2	975.54
									LISPPVHLPR	4	N-Term(iTRAQ4plex		3.47	54	3	424.94
									IVLNFNPHFEIEK	1	N-Term(iTRAQ4plex); K13(iTRAQ4plex			50		
260593665	PEPD	xaa-Pro dipeptidase isoform 3	5.83	0	2	3		47.2								
									FEVNNTILHPEIVECR	2	N-Term(iTRAQ4plex); C15(Methylthio		3.68	44	3	701.69
110349772	COL1A1	collagen alpha-1(I) chain preproprotein	5.81	7	7	20	0.918	138.8								
									NSVAYMDQQTGNLK	4	N-Term(iTRAQ4plex); K14(iTRAQ4plex	0.881	5.88	74	2	928.98
									GETGPAGPAGPVGPVGAR	6	N-Term(iTRAQ4plex	0.893	5.65	90	2	845.96
									STGGISVPGPMGPSGPR	2	N-Term(iTRAQ4plex	1.128	5.32	81	2	849.44
									DLEVDTTLK	2	N-Term(iTRAQ4plex); K9(iTRAQ4plex	1.057	4.12	58	2	661.37
									SLSQQIENIR	4	N-Term(iTRAQ4plex	0.853	3.69	63	2	666.37
									SGDRGETGPAGPAGPVGPVGAR	1	N-Term(iTRAQ4plex	0.783		39		
310118013	LOC100509457	PREDICTED: HLA class II histocompatibility antigen, DQ alpha 1 chain-like isoform 4	5.8	0	1	2		23.2								
									VEHWGLDEPLLK	2	N-Term(iTRAQ4plex); K12(iTRAQ4plex		3.45	47	3	575.32

41872562	NRP2	neuropilin-2 isoform 1 precursor	5.8	0	4	9		104.8								
									IANEQISASSTYSDGF	2	N-Term(iTRAQ4plex		5.52	103	2	921.95
									VFQANNDATEVVLNI	2	N-Term(iTRAQ4plex); K15(iTRAQ4plex		3.93	77	2	975.54
									LISPPVHLPR	4	N-Term(iTRAQ4plex		3.47	54	3	424.94
									IVLNFNPHFEIEK	1	N-Term(iTRAQ4plex); K13(iTRAQ4plex			50		
310125016	LOC100507718	PREDICTED: HLA class II histocompatibility antigen, DQ alpha 1 chain-like isoform 4	5.77	0	1	2		23.4								
									VEHWGLDEPLLK	2	N-Term(iTRAQ4plex); K12(iTRAQ4plex		3.45	47	3	575.32
310125247	LOC100507686	PREDICTED: HLA class II histocompatibility antigen, DQ alpha 1 chain-like isoform 4	5.77	0	1	2		23.4								
									VEHWGLDEPLLK	2	N-Term(iTRAQ4plex); K12(iTRAQ4plex		3.45	47	3	575.32
310125111	LOC100507681	PREDICTED: HLA class I histocompatibility antigen, Cw-15 alpha chain-like isoform 14	5.76	0	1	2		26.5								
									APWVEQEGPEYWDR	2	N-Term(iTRAQ4plex		3.42	63	2	953.45
194097398	PTPRG	receptor-type tyrosine-protein phosphatase gamma precursor	5.74	7	7	23	0.953	161.9								
									DDYFVSGAGLPGR	4	N-Term(iTRAQ4plex	1.028	5.47	76	2	749.38
									ATISHVSPDSLYLFR	3	N-Term(iTRAQ4plex	0.886	5	40	3	617.34
									HQSPIDILDQYAR	2	N-Term(iTRAQ4plex	0.96	4.74	51	2	850.44
									DNSALDPIIHGLK	4	N-Term(iTRAQ4plex); K13(iTRAQ4plex	0.993	4.48	61	3	560.99
									ETFLDPFVLR	2	N-Term(iTRAQ4plex	0.866	3.68	46	2	690.89
									TVAILLK	6	N-Term(iTRAQ4plex); K7(iTRAQ4plex	0.946	3.44	45	2	523.36
									DLLPASLGSYYR	2	N-Term(iTRAQ4plex	0.81	3.37	60	2	749.91
23110962	CTSS	cathepsin S isoform 1 preproprotein	5.74	0	2	3		37.5								
									GIDSDASYPYK	2	N-Term(iTRAQ4plex); K11(iTRAQ4plex		4.6	47	2	752.38
									HPSFFLYR	1	N-Term(iTRAQ4plex			37		
10835047	SGCE	epsilon-sarcoglycan isoform 2	5.72	0	2	8		49.8								
									NVYPSAGVLFVHVLEF	2	N-Term(iTRAQ4plex		4.29	46	3	648.70
									VPLPINDLK	6	N-Term(iTRAQ4plex); K9(iTRAQ4plex		3.39	61	2	648.91
136255216	PRRT3	proline-rich transmembrane protein 3 precursor	5.71	3	3	13	1.041	102.1								
									GSVGSEPQAFDVFPENPF	3	N-Term(iTRAQ4plex	1.161	7.21	75	2	1039.01
									HAPAEEMPEKPVASPLGPALYGPK	2	N-Term(iTRAQ4plex); K10(iTRAQ4plex); K24(iTRAQ4plex)	0.93	5.94	55	3	973.53
									SLPPAEELPVETPK	8	N-Term(iTRAQ4plex); K14(iTRAQ4plex	1.04	5.52	77	2	898.01
145701025	MEGF8	multiple epidermal growth factor-like domains protein 8	5.69	11	11	40	0.81	295.7								
									APQTVELPAVAGHTLTAF	4	N-Term(iTRAQ4plex	0.852	9.09	64	3	659.37
									SFHAAAYVPAGF	6	N-Term(iTRAQ4plex	0.816	4.95	76	3	464.25
									FHVELAAPSPELYSLHCPDF	4	N-Term(iTRAQ4plex); C17(Methylthio	0.731	4.74	77	3	824.41
									SASVGPPMEESVAHAVAAVGSI	4	N-Term(iTRAQ4plex	0.816	4.66	39	3	751.72
									TWSLLAPSQGAK	5	N-Term(iTRAQ4plex); K12(iTRAQ4plex	0.819	4.58	77	2	773.95
									TPHDLFSSGLFR	7	N-Term(iTRAQ4plex	0.886	3.88	52	3	507.60
									TGVPGGSEISFFFLEPYR	4	N-Term(iTRAQ4plex	0.801	3.62	52	2	1074.05
									GDLMAYK	2	N-Term(iTRAQ4plex); K7(iTRAQ4plex	0.826	3.38	43	2	543.30
									WTQMLAGAEDGGPGPSPR	1	N-Term(iTRAQ4plex); M4(Oxidation	0.753		68		
									SASVGPPMEESVAHAVAAVGSI	1	N-Term(iTRAQ4plex); M8(Oxidation	0.763		52		
									FLDTGVVQSDR	1	N-Term(iTRAQ4plex	0.711		40		
									CEPGFLGR	1	N-Term(iTRAQ4plex); C1(Methylthio	0.685		38		
45359859	GRB2	growth factor receptor-bound protein 2 isoform 2	5.68	0	1	2		20.5								
									ATADDELSFK	2	N-Term(iTRAQ4plex); K10(iTRAQ4plex		4.83	69	2	692.86
38045913	NME1	nucleoside diphosphate kinase A isoform a	5.65	0	1	1		19.6								
									FMQASEDLLK	1	N-Term(iTRAQ4plex); K10(iTRAQ4plex			64		
291327493	FGFR1	basic fibroblast growth factor receptor 1 isoform 2 precursor	5.61	0	5	14		91.5								
									LRDDVQSINWLR	5	N-Term(iTRAQ4plex		5.08	62	2	829.96
									IGPDNLPYVQILK	3	N-Term(iTRAQ4plex); K13(iTRAQ4plex		4.03	40	2	879.53
									VYSDPQPHIQWLK	2	N-Term(iTRAQ4plex); K13(iTRAQ4plex		3.51	44	3	633.68
									EMEVLHLR	3	N-Term(iTRAQ4plex		3.51	39	2	585.82
									DDVQSINWLR	1	N-Term(iTRAQ4plex			37		
4504919	KRT8	keratin, type II cytoskeletal 8	5.59	0	3	10		53.7								
									LALDIEIATYR	5	N-Term(iTRAQ4plex		3.43	51	2	711.41
									EYQELMNVK	2	N-Term(iTRAQ4plex); K9(iTRAQ4plex		3.39	60	2	721.38
									FASFIDK	4	N-Term(iTRAQ4plex); K7(iTRAQ4plex		3.19	53	2	558.31
238624147	NPTN	neuroplastin isoform c precursor	5.58	0	2	3		43.9								
									IVTSEEVIIR	2	N-Term(iTRAQ4plex		3.78	38	2	651.89
									SVGYPHPDWIWR	1	N-Term(iTRAQ4plex			34		
62414289	VIM	vimentin	5.58	2	2	3	0.605	53.6								
									LGDLYEEEMR	2	N-Term(iTRAQ4plex	0.523	3.62	73	2	699.84
									DGQVINETSQHHDDLE	1	N-Term(iTRAQ4plex	0.699		31		
4502809	CHIT1	chitotriosidase-1 precursor	5.58	2	2	4	9.398	51.6								
									VGAPATGSGTPGPFTK	2	N-Term(iTRAQ4plex); K16(iTRAQ4plex	7.611	5.5	111	2	866.97
									LILGMPTYGR	2	N-Term(iTRAQ4plex	11.604	3.28	41	2	632.86
150378535	SGCE	epsilon-sarcoglycan isoform 3	5.54	0	2	8		51.4								
									NVYPSAGVLFVHVLEF	2	N-Term(iTRAQ4plex		4.29	46	3	648.70
									VPLPINDLK	6	N-Term(iTRAQ4plex); K9(iTRAQ4plex		3.39	61	2	648.91
145309326	LAMC1	laminin subunit gamma-1 precurso	5.53	7	7	20	0.907	177.5								
									LNEIEGTLNK	4	N-Term(iTRAQ4plex); K10(iTRAQ4plex	0.972	4.64	70	2	709.90
									NTIEETGNLAEQAF	5	N-Term(iTRAQ4plex	0.905	4.31	82	2	845.43
									LSAEDLVLEGAGLR	4	N-Term(iTRAQ4plex	0.903	4	96	2	793.95
									SYYYAISDFAVGGR	2	N-Term(iTRAQ4plex	0.78	3.6	50	2	856.92
									DIEEIMK	2	N-Term(iTRAQ4plex); K7(iTRAQ4plex	0.882	3.49	40	2	583.32
									LNTFGDEVFNDPK	2	N-Term(iTRAQ4plex); K13(iTRAQ4plex	0.789		57		
									EAQQALGSAAADATEAK	1	N-Term(iTRAQ4plex); K17(iTRAQ4plex	1.019		37		
6912646	NPTN	neuroplastin isoform b precursor	5.53	0	2	3		44.4								
									IVTSEEVIIR	2	N-Term(iTRAQ4plex		3.78	38	2	651.89
									SVGYPHPDWIWR	1	N-Term(iTRAQ4plex			34		
41327736	EGFR	epidermal growth factor receptor isoform d precursor	5.53	0	3	5		77.3								
									NLQEILHGAVR	2	N-Term(iTRAQ4plex		4.03	47	3	465.27
									EISDGDVIISGNK	2	N-Term(iTRAQ4plex); K13(iTRAQ4plex		3.52	67	2	817.95
									ACGADSYEMEEDGVF	1	N-Term(iTRAQ4plex); C2(Methylthio			49		
310113085	LOC390791	PREDICTED: peptidyl-prolyl cis-trans isomerase A-like	5.51	0	1	3		13.6								
									TEWLDGK	3	N-Term(iTRAQ4plex); K7(iTRAQ4plex		3.28	47	2	568.82
86792863	DPP6	dipeptidyl aminopeptidase-like protein 6 isoform 3	5.49	0	4	7		91.2								
									VSALEEQQFLIIHPTADEK	2	N-Term(iTRAQ4plex); K19(iTRAQ4plex		3.79	59	3	819.45

									VAVTWLNR	2	N-Term(iTRAQ4plex		3.26	47	2	551.83
									EYYITMVK	2	N-Term(iTRAQ4plex); K8(iTRAQ4plex		3.21	37	2	667.87
86792778	DPP6	dipeptidyl aminopeptidase-like protein 6 isoform 2	5.48	0	4	7		91.3								
									VSALEEQQFLIIHPTADEK	2	N-Term(iTRAQ4plex); K19(iTRAQ4plex		3.79	59	3	819.45
									VAVTWLNR	2	N-Term(iTRAQ4plex		3.26	47	2	551.83
									EYYITMVK	2	N-Term(iTRAQ4plex); K8(iTRAQ4plex		3.21	37	2	667.87
53988378	MSLN	mesothelin isoform 1 preproprotein	5.47	1	3	6	0.813	68								
									GHEMSPQVATLIDR	2	N-Term(iTRAQ4plex	0.813	3.69	56	2	849.44
									LLGPHVEGLK	2	N-Term(iTRAQ4plex); K10(iTRAQ4plex		3.61	43	3	450.95
									EIDESLIFYK	2	N-Term(iTRAQ4plex); K10(iTRAQ4plex		3.26	67	2	772.93
65506745	NTRK2	BDNF/NT-3 growth factors receptor isoform c precursor	5.47	0	3	5		91.9								
									SSPDTQDLYCLNESSK	2	N-Term(iTRAQ4plex); C10(Methylthio); K16(iTRAQ4plex)		3.28	67	2	1060.99
									RLEIINEDDVEAYVGLR	2	N-Term(iTRAQ4plex			59		
									SNEIPSTDVTDK	1	N-Term(iTRAQ4plex); K12(iTRAQ4plex			57		
262359974	NLGN3	neuroligin-3 isoform 3	5.45	0	4	9		89.5								
									FEEVAWSK	4	N-Term(iTRAQ4plex); K8(iTRAQ4plex		3.67	47	2	642.36
									ELVEQDIQPAR	2	N-Term(iTRAQ4plex		3.61	87	2	721.39
									GNYGLLDQIQALR	2	N-Term(iTRAQ4plex		3.18	52	2	802.95
262118210	IDS	iduronate 2-sulfatase isoform c	5.43	0	2	10		52.4								
									QSTEQAIQLLEK	4	N-Term(iTRAQ4plex); K12(iTRAQ4plex		5.99	75	2	838.48
									DLEEDPYLPGNPR	6	N-Term(iTRAQ4plex		4.66	68	2	829.91
308193325	PRSS3	trypsin-3 isoform 4 preproprotein	5.42	0	1	2		25.9								
									VLEGNEQFINAAK	2	N-Term(iTRAQ4plex); K13(iTRAQ4plex		6.38	67	2	860.98
150378454	SGCE	epsilon-sarcoglycan isoform 1	5.41	0	2	8		52.5								
									NVYPSAGVLFVHVLER	2	N-Term(iTRAQ4plex		4.29	46	3	648.70
									VPLPINDLK	6	N-Term(iTRAQ4plex); K9(iTRAQ4plex		3.39	61	2	648.91
189083844	CTSC	dipeptidyl peptidase 1 isoform a preproprotein	5.4	1	3	3	0.956	51.8								
									WFAFFK	1	N-Term(iTRAQ4plex); K6(iTRAQ4plex			40		
									VVVYLQK	1	N-Term(iTRAQ4plex); K7(iTRAQ4plex			39		
291327497	FGFR1	basic fibroblast growth factor receptor 1 isoform 14 precursor	5.39	0	5	14		95.3								
									LRDDVQSINWLR	5	N-Term(iTRAQ4plex		5.08	62	2	829.96
									IGPDNLPYVQILK	3	N-Term(iTRAQ4plex); K13(iTRAQ4plex		4.03	40	2	879.53
									VYSDPQPHIQWLK	2	N-Term(iTRAQ4plex); K13(iTRAQ4plex		3.51	44	3	633.68
									EMEVLHLR	3	N-Term(iTRAQ4plex		3.51	39	2	585.82
									DDVQSINWLR	1	N-Term(iTRAQ4plex			37		
4885111	CALML3	calmodulin-like protein 3	5.37	0	1	2		16.9								
									EAFSLFDK	2	N-Term(iTRAQ4plex); K8(iTRAQ4plex		4.19	47	2	622.84
21361306	NTRK2	BDNF/NT-3 growth factors receptor isoform a precursor	5.37	0	3	5		93.8								
									SSPDTQDLYCLNESSK	2	N-Term(iTRAQ4plex); C10(Methylthio); K16(iTRAQ4plex)		3.28	67	2	1060.99
									RLEIINEDDVEAYVGLR	2	N-Term(iTRAQ4plex			59		
									SNEIPSTDVTDK	1	N-Term(iTRAQ4plex); K12(iTRAQ4plex			57		
15029530	LRRC4	leucine-rich repeat-containing protein 4 precursor	5.36	1	3	8	0.848	72.7								
									NNPIESIPSYAFNR	6	N-Term(iTRAQ4plex		4.89	57	2	883.45
									GLSEVPQGIPSNTR	1	N-Term(iTRAQ4plex	0.848		47		
									LDLGELK	1	N-Term(iTRAQ4plex); K7(iTRAQ4plex			43		
310128955	LOC100510361	PREDICTED: putative V-set and immunoglobulin domain-containing protein 6-like	5.33	0	1	2		18.9								
									VTISVDTSK	2	N-Term(iTRAQ4plex); K9(iTRAQ4plex		3.72	54	2	619.37
51593088	NLGN3	neuroligin-3 isoform 2	5.31	0	4	9		91.5								
									FEEVAWSK	4	N-Term(iTRAQ4plex); K8(iTRAQ4plex		3.67	47	2	642.36
									ELVEQDIQPAR	2	N-Term(iTRAQ4plex		3.61	87	2	721.39
									GNYGLLDQIQALR	2	N-Term(iTRAQ4plex		3.18	52	2	802.95
210147552	POMGNT1	protein O-linked-mannose beta-1,2-N-acetylglucosaminyltransferase 1	5.3	3	3	5	0.836	75.2								
									LLSEAEVLDHSK	2	N-Term(iTRAQ4plex); K12(iTRAQ4plex	0.836	5.93	57	3	543.64
									SLGSQAGPALGWR	2	N-Term(iTRAQ4plex	0.96	5.33	53	2	722.39
94538337	SIRPG	signal-regulatory protein gamma isoform 2 precursor	5.29	0	1	7		18.5								
									VTTVSDLTK	7	N-Term(iTRAQ4plex); K9(iTRAQ4plex		4.15	66	2	626.37
221316738	GAS6	growth arrest-specific protein 6 isoform 3	5.28	0	2	3		42								
									IAVAGDLFQPER	2	N-Term(iTRAQ4plex		4.45	37	2	730.40
									LVAEFDFR	1	N-Term(iTRAQ4plex			43		
21536452	PRSS3	trypsin-3 isoform 2 preproprotein	5.26	0	1	2		26.7								
									VLEGNEQFINAAK	2	N-Term(iTRAQ4plex); K13(iTRAQ4plex		6.38	67	2	860.98
4826870	NUCB2	nucleobindin-2 precursor	5.24	2	2	3	1.183	50.2								
									NEEDDMVEMEEER	2	N-Term(iTRAQ4plex	1.172	4.17	92	2	899.87
									LVTLEEFLK	1	N-Term(iTRAQ4plex); K9(iTRAQ4plex	1.194		52		
209364544	CLSTN2	calsyntenin-2 precursor	5.24	4	4	7	0.879	106.9								
									DVNEFAPTFK	2	N-Term(iTRAQ4plex); K10(iTRAQ4plex	0.895	4.35	54	2	728.39
									YHFNPSQSILVMEGDDIGNINR	2	N-Term(iTRAQ4plex	1.47	3.44	67	3	888.44
									GVTLFPDIK	2	N-Term(iTRAQ4plex); K9(iTRAQ4plex	0.81	3.16	52	2	639.39
									VISCLQACK	1	N-Term(iTRAQ4plex); C4(Methylthio); C8(Methylthio); K9(iTRAQ4plex)	0.863		54		
7019545	NENF	neudesin precursor	5.23	1	1	2	1.283	18.8								
									LFTEEELAR	2	N-Term(iTRAQ4plex	1.283	3.76	55	2	626.34
33457348	C19orf10	hypothetical protein LOC56005 precursor	5.2	1	1	1	0.934	18.8								
									SYLYFTQFK	1	N-Term(iTRAQ4plex); K9(iTRAQ4plex	0.934		58		
310124981	LOC100507680	PREDICTED: HLA class I histocompatibility antigen, A-69 alpha chain-like isoform 12	5.19	0	1	2		30.1								
									FIAVGYVDDTQFVR	2	N-Term(iTRAQ4plex		3.38	70	2	887.47
310124987	LOC100507680	PREDICTED: HLA class I histocompatibility antigen, A-69 alpha chain-like isoform 14	5.19	0	1	2		29.8								
									FIAVGYVDDTQFVR	2	N-Term(iTRAQ4plex		3.38	70	2	887.47
262359971	NLGN3	neuroligin-3 isoform 1	5.19	0	4	9		93.8								
									FEEVAWSK	4	N-Term(iTRAQ4plex); K8(iTRAQ4plex		3.67	47	2	642.36
									ELVEQDIQPAR	2	N-Term(iTRAQ4plex		3.61	87	2	721.39
									GNYGLLDQIQALR	2	N-Term(iTRAQ4plex		3.18	52	2	802.95
4758092	CTBS	di-N-acetylchitobiase precursor	5.19	1	1	2	0.959	43.7								
									DPAGHFHQVWYDNPQSISLK	2	N-Term(iTRAQ4plex); K20(iTRAQ4plex	0.959	7	102	3	876.45
157738641	NEFM	neurofilament medium polypeptide isoform 2	5.19	0	1	1		59.4								

									EGSSEKEEGEQEEGETEAEAEGEEAEAK	1	N-Term(iTRAQ4plex); K6(iTRAQ4plex); K28(iTRAQ4plex)			39		
167857782	MGAT1	alpha-1,3-mannosyl-glycoprotein 2-beta-N-acetylglucosaminyltransferase	5.17	2	2	2	0.783	50.8								
									ALGVMDDLK	1	N-Term(iTRAQ4plex); K9(iTRAQ4plex	0.765		64		
									QPDLSSIAVPPDHR	1	N-Term(iTRAQ4plex	0.801		42		
310114958	LOC100507703	PREDICTED: HLA class I histocompatibility antigen, A-69 alpha chain-like isoform 13	5.13	0	1	2		30.4								
									FIAVGYVDDTQFVR	2	N-Term(iTRAQ4plex		3.38	70	2	887.47
310114964	LOC100507703	PREDICTED: HLA class I histocompatibility antigen, A-69 alpha chain-like isoform 18	5.13	0	1	2		30.1								
									FIAVGYVDDTQFVR	2	N-Term(iTRAQ4plex		3.38	70	2	887.47
310124905	LOC100507680	PREDICTED: HLA class I histocompatibility antigen, A-32 alpha chain-like isoform 14	5.13	0	1	2		30.2								
									FIAVGYVDDTQFVR	2	N-Term(iTRAQ4plex		3.38	70	2	887.47
310125069	LOC100507680	PREDICTED: HLA class I histocompatibility antigen, A-43 alpha chain-like isoform 19	5.13	0	1	2		30.2								
									FIAVGYVDDTQFVR	2	N-Term(iTRAQ4plex		3.38	70	2	887.47
310125182	LOC100507680	PREDICTED: HLA class I histocompatibility antigen, A-74 alpha chain-like isoform 13	5.13	0	1	2		30.4								
									FIAVGYVDDTQFVR	2	N-Term(iTRAQ4plex		3.38	70	2	887.47
310128259	LOC100510327	PREDICTED: HLA class I histocompatibility antigen, A-30 alpha chain-like isoform 10	5.13	0	1	2		30.1								
									FIAVGYVDDTQFVR	2	N-Term(iTRAQ4plex		3.38	70	2	887.47
154816176	CXCL16	C-X-C motif chemokine 16	5.13	1	1	5	1.296	29.5								
									ISSDSPPSVQFMNR	5	N-Term(iTRAQ4plex	1.296	5.75	90	2	854.93
310124841	LOC100507705	PREDICTED: class I histocompatibility antigen, Gogo-B*0103 alpha chain-like isoform 13	5.11	0	1	2		30.3								
									APWVEQEGPEYWDR	2	N-Term(iTRAQ4plex		3.42	63	2	953.45
310125107	LOC100507681	PREDICTED: HLA class I histocompatibility antigen, Cw-15 alpha chain-like isoform 13	5.11	0	1	2		30.2								
									APWVEQEGPEYWDR	2	N-Term(iTRAQ4plex		3.42	63	2	953.45
21166382	NRXN2	neurexin-2-beta isoform beta precursor	5.11	0	3	8		70.9								
									VLALAAESDPNVR	2	N-Term(iTRAQ4plex		5.22	43	2	749.92
									VVDEWLLDK	4	N-Term(iTRAQ4plex); K9(iTRAQ4plex		4.3	64	2	702.91
									QLTIFNSQAAIK	2	N-Term(iTRAQ4plex); K12(iTRAQ4plex		3.19	63	2	811.48
86792774	DPP6	dipeptidyl aminopeptidase-like protein 6 isoform 1	5.09	0	4	7		97.5								
									VSALEEQQFLIIHPTADEK	2	N-Term(iTRAQ4plex); K19(iTRAQ4plex		3.79	59	3	819.45
									VAVTWLNR	2	N-Term(iTRAQ4plex		3.26	47	2	551.83
									EYYITMVK	2	N-Term(iTRAQ4plex); K8(iTRAQ4plex		3.21	37	2	667.87
167830477	MFGE8	lactadherin isoform b	5.07	0	2	3		37.5								
									EFVGNWNK	2	N-Term(iTRAQ4plex); K8(iTRAQ4plex		3.42	41	2	641.35
									ILPVAWHNR	1	N-Term(iTRAQ4plex			37		
110578649	CACHD1	VWFA and cache domain-containing protein 1	5.07	5	5	10	0.906	136.6								
									EPLVANDILNHPNFVK	2	N-Term(iTRAQ4plex); K16(iTRAQ4plex	0.906	4.81	68	3	703.40
									ISVLTVADTVR	2	N-Term(iTRAQ4plex	0.888	4.47	56	2	659.40
									IYPGSLMDK	2	N-Term(iTRAQ4plex); K9(iTRAQ4plex	0.932	3.81	50	2	656.37
									DLNSVLADNLK	2	N-Term(iTRAQ4plex); K11(iTRAQ4plex	0.848	3.77	77	2	745.43
									DAAQVILSAIDEHDK	2	N-Term(iTRAQ4plex); K15(iTRAQ4plex	1.105	3.74	72	3	638.35
149589008	PEPD	xaa-Pro dipeptidase isoform 1	5.07	0	2	3		54.5								
									FEVNNTILHPEIVECR	2	N-Term(iTRAQ4plex); C15(Methylthio		3.68	44	3	701.69
32567786	KRT79	keratin, type II cytoskeletal 79	5.05	0	3	6		57.8								
									FASFIDK	4	N-Term(iTRAQ4plex); K7(iTRAQ4plex		3.19	53	2	558.31
27262667	CSF1	macrophage colony-stimulating factor 1 isoform a precursor	5.05	1	2	16	0.972	60.1								
									FNSVPLTDTGHER	14	N-Term(iTRAQ4plex	0.972	5.42	62	3	539.61
									STCQSFEPPETPVVK	2	N-Term(iTRAQ4plex); C3(Methylthio); K15(iTRAQ4plex)		3.64	45	2	991.99
27754773	PCDH1	protocadherin-1 isoform 2 precursor	5.01	0	5	16		133.6								
									NTGLITVQGPVDR	2	N-Term(iTRAQ4plex		4.67	74	2	757.43
									YFLQTTTPLDYEK	2	N-Term(iTRAQ4plex); K13(iTRAQ4plex		4.65	75	2	954.01
									QPQLIVMGNLDR	4	N-Term(iTRAQ4plex		4.56	58	2	764.43
									TGDIFTTETSIDR	6	N-Term(iTRAQ4plex		4.14	80	2	800.40
									EQQSTYTFQLK	2	N-Term(iTRAQ4plex); K11(iTRAQ4plex		4.06	51	2	830.94
158937236	NPEPPS	puromycin-sensitive aminopeptidase	5.01	4	4	7	0.646	103.2								
									AFFESHPAPSAER	3	N-Term(iTRAQ4plex	0.631	4.25	51	3	530.60
									YAAVTQFEATDAR	2	N-Term(iTRAQ4plex	0.601	4.1	51	2	793.90
									ETALLIDPK	1	N-Term(iTRAQ4plex); K9(iTRAQ4plex	0.646		47		
13489087	SERPINB1	leukocyte elastase inhibitor	5.01	1	1	2	1.167	42.7								
									TYGADLASVDFQHASEDAR	2	N-Term(iTRAQ4plex	1.167	3.62	52	3	733.01
308818206	ECM2	extracellular matrix protein 2 isoform 3 precursor	4.98	0	3	8		73.3								
									ELFLDHNDLK	4	N-Term(iTRAQ4plex); K10(iTRAQ4plex		4.78	74	3	511.28
									KINVIVLR	3	N-Term(iTRAQ4plex); K1(iTRAQ4plex			52		
									SLLHLVLLGNQIER	1	N-Term(iTRAQ4plex			34		
48255905	TAGLN	transgelin	4.98	1	1	2	0.915	22.6								
									QMEQVAQFLK	2	N-Term(iTRAQ4plex); K10(iTRAQ4plex	0.915	4.08	51	2	755.42
308193323	PRSS3	trypsin-3 isoform 3 preproprotein	4.98	0	1	2		28.3								
									VLEGNEQFINAAK	2	N-Term(iTRAQ4plex); K13(iTRAQ4plex		6.38	67	2	860.98
13435366	DSC2	desmocollin-2 isoform Dsc2b preproprotein	4.96	0	3	8		93.7								
									VTVEDKDLVNTANWR	2	N-Term(iTRAQ4plex); K6(iTRAQ4plex		4.62	47	3	683.37
									IFVFLEHQTK	4	N-Term(iTRAQ4plex); K10(iTRAQ4plex		4.48	58	2	775.46
									TNEGVLCVVKPLNYEEK	2	N-Term(iTRAQ4plex); C7(Methylthio); K10(iTRAQ4plex); K17(iTRAQ4plex)		4.37	82	3	805.10
57546917	MRC1L1	macrophage mannose receptor 1-like protein 1 precursor	4.95	0	7	12		165.9								
									SQGPEIVEVEK	2	N-Term(iTRAQ4plex); K11(iTRAQ4plex		5.41	68	2	751.92
									GEDLFFNYGNR	2	N-Term(iTRAQ4plex		4.22	47	2	738.36
									DYQYYFSK	2	N-Term(iTRAQ4plex); K8(iTRAQ4plex		3.88	43	2	701.36
									ALGGDLASINNK	2	N-Term(iTRAQ4plex); K12(iTRAQ4plex		3.79	67	2	730.92
									IFGFMEEER	2	N-Term(iTRAQ4plex		3.62	41	2	651.32
									WENLECVQK	1	N-Term(iTRAQ4plex); C6(Methylthio); K9(iTRAQ4plex)			48		
									TGIAGGLWDVLK	1	N-Term(iTRAQ4plex); K12(iTRAQ4plex			36		
4505245	MRC1	macrophage mannose receptor 1 precursor	4.95	0	7	12		165.9								
									SQGPEIVEVEK	2	N-Term(iTRAQ4plex); K11(iTRAQ4plex		5.41	68	2	751.92
									GEDLFFNYGNR	2	N-Term(iTRAQ4plex		4.22	47	2	738.36

									DYQYYFSK	2	N-Term(iTRAQ4plex); K8(iTRAQ4plex		3.88	43	2	701.36
									ALGGDLASINNK	2	N-Term(iTRAQ4plex); K12(iTRAQ4plex		3.79	67	2	730.92
									IFGFMEEER	2	N-Term(iTRAQ4plex		3.62	41	2	651.32
									WENLECVQK	1	N-Term(iTRAQ4plex); C6(Methylthio); K9(iTRAQ4plex)			48		
									TGIAGGLWDVLK	1	N-Term(iTRAQ4plex); K12(iTRAQ4plex			36		
221316735	GAS6	growth arrest-specific protein 6 isoform 2	4.94	0	2	3		44.8								
									IAVAGDLFQPER	2	N-Term(iTRAQ4plex		4.45	37	2	730.40
									LVAEFDFR	1	N-Term(iTRAQ4plex			43		
14589937	PCDH8	protocadherin-8 isoform 2 precursor	4.93	0	3	4		103.6								
									YSTFEEDAPGTVIGTLAEDLHMK	2	N-Term(iTRAQ4plex); K23(iTRAQ4plex		4.25	56	3	938.14
									LVHVEVEVR	1	N-Term(iTRAQ4plex			38		
4885397	HBZ	hemoglobin subunit zeta	4.93	0	1	1		15.6								
									VDPVNFK	1	N-Term(iTRAQ4plex); K7(iTRAQ4plex			55		
126012571	HSPG2	basement membrane-specific heparan sulfate proteoglycan core protein precursor	4.92	16	16	51	0.894	468.5								
									GSIQVDGEELVSGR	9	N-Term(iTRAQ4plex	0.829	5.9	98	2	795.42
									LVSEDPINDGEWHR	6	N-Term(iTRAQ4plex	0.839	5.21	75	2	905.95
									SIEYSPQLEDAGSR	2	N-Term(iTRAQ4plex	0.926	4.99	71	2	848.42
									FSSGITGCVK	6	N-Term(iTRAQ4plex); C8(Methylthio); K10(iTRAQ4plex)	0.894	4.6	65	2	666.85
									GSVYIGGAPDVATLTGGR	6	N-Term(iTRAQ4plex	1.059	4.5	83	2	918.00
									VGGHLRPGIVQSGGVVR	2	N-Term(iTRAQ4plex	0.917	4.18	36	2	916.54
									CLIHDGAAPISLEWK	2	N-Term(iTRAQ4plex); C1(Methylthio); K15(iTRAQ4plex)	0.774	4.15	78	3	663.02
									YELGSGLAVLR	2	N-Term(iTRAQ4plex	0.85	4.12	63	2	661.38
									FDAGSGMATIR	2	N-Term(iTRAQ4plex	0.874	4.01	70	2	635.32
									CQVSGSPPHYFYWSR	2	N-Term(iTRAQ4plex); C1(Methylthio	0.884	3.57	37	3	668.64
									SLPEVPETIELEVR	3	N-Term(iTRAQ4plex	0.86	3.17	47	2	877.99
									DFISLGLQDGHLVFR	3	N-Term(iTRAQ4plex	0.894		73		
									AELLVTEAPSKPITVTVEEQR	1	N-Term(iTRAQ4plex); K11(iTRAQ4plex	0.952		49		
									AFAHLQVPER	3	N-Term(iTRAQ4plex	0.929		47		
									FDQPDDFK	1	N-Term(iTRAQ4plex); K8(iTRAQ4plex	1.016		37		
45580723	HPR	haptoglobin-related protein precursor	4.89	0	2	3		39								
									VMPICLPSK	2	N-Term(iTRAQ4plex); C5(Methylthio); K9(iTRAQ4plex)		3.26	48	2	661.37
									GSFPWQAK	1	N-Term(iTRAQ4plex); K8(iTRAQ4plex			43		
312222733	SPON2	spondin-2 precursor	4.83	2	2	4	0.919	35.7								
									YSITFTGK	2	N-Term(iTRAQ4plex); K8(iTRAQ4plex	0.97	3.43	41	2	602.84
									GEAWALMK	2	N-Term(iTRAQ4plex); K8(iTRAQ4plex	0.871	3.21	53	2	597.33
310118019	LOC100509457	PREDICTED: HLA class II histocompatibility antigen, DQ alpha 1 chain-like isoform 2	4.82	0	1	2		27.4								
									VEHWGLDEPLLK	2	N-Term(iTRAQ4plex); K12(iTRAQ4plex		3.45	47	3	575.32
4557351	BCHE	cholinesterase precursor	4.82	3	3	4	0.899	68.4								
									TQILVGVNK	2	N-Term(iTRAQ4plex); K9(iTRAQ4plex	0.899	3.94	53	2	630.40
									IFFPGVSEFGK	1	N-Term(iTRAQ4plex); K11(iTRAQ4plex	0.824		74		
									WNNYMMDWK	1	N-Term(iTRAQ4plex); K9(iTRAQ4plex	1.328		62		
11231177	LAIR1	leukocyte-associated immunoglobulin-like receptor 1 isoform b precursor	4.81	0	1	3		29.8								
									IDSVSEGNAGPYR	3	N-Term(iTRAQ4plex		5.07	80	2	754.88
17149834	SORT1	sortilin preproprotein	4.81	3	3	6	1.018	92								
									VVLTAEVSGGSR	2	N-Term(iTRAQ4plex	1.031	4.13	54	2	659.88
									LDAPPPPAAPLPR	2	N-Term(iTRAQ4plex	1.005	3.85	54	2	728.43
									DPIYFTGLASEPGAR	2	N-Term(iTRAQ4plex	1.005		57		
310125022	LOC100507718	PREDICTED: HLA class II histocompatibility antigen, DQ alpha 1 chain-like isoform 3	4.8	0	1	2		27.7								
									VEHWGLDEPLLK	2	N-Term(iTRAQ4plex); K12(iTRAQ4plex		3.45	47	3	575.32
310125253	LOC100507686	PREDICTED: HLA class II histocompatibility antigen, DQ alpha 1 chain-like isoform 3	4.8	0	1	2		27.6								
									VEHWGLDEPLLK	2	N-Term(iTRAQ4plex); K12(iTRAQ4plex		3.45	47	3	575.32
310133348	LOC100507718	PREDICTED: HLA class II histocompatibility antigen, DQ alpha 1 chain-like isoform 3	4.8	0	1	2		27.6								
									VEHWGLDEPLLK	2	N-Term(iTRAQ4plex); K12(iTRAQ4plex		3.45	47	3	575.32
310124993	LOC100507680	PREDICTED: HLA class I histocompatibility antigen, A-69 alpha chain-like isoform 18	4.79	0	1	2		32.3								
									FIAVGYVDDTQFVR	2	N-Term(iTRAQ4plex		3.38	70	2	887.47
7657465	PODXL2	podocalyxin-like protein 2 precursor	4.79	2	2	4	1.407	65								
									DFSLTSSSQTPGATK	2	N-Term(iTRAQ4plex); K15(iTRAQ4plex	1.156	6.59	98	2	907.97
									GPQLLALVEEVLPR	2	N-Term(iTRAQ4plex	1.712	4.75	88	2	839.51
39777608	SEMA4B	semaphorin-4B precursor	4.78	3	4	6	0.921	92.7								
									CPFDPNFK	2	N-Term(iTRAQ4plex); C1(Methylthio); K8(iTRAQ4plex)	0.925	3.18	42	2	651.32
									VPGLHHTYDVLFLGTGDGR	1	N-Term(iTRAQ4plex	0.76		48		
									VFSGLYK	2	N-Term(iTRAQ4plex); K7(iTRAQ4plex	0.962		42		
									WTSFLK	1	N-Term(iTRAQ4plex); K6(iTRAQ4plex			38		
126012562	LRP1	prolow-density lipoprotein receptor-related protein 1 precursor	4.78	16	16	32	0.861	504.3								
									NAVVQGLEQPHGLVVHPLR	4	N-Term(iTRAQ4plex	0.865	8.34	71	4	552.57
									VFFTDYGQIPK	2	N-Term(iTRAQ4plex); K11(iTRAQ4plex	0.944	5.36	54	2	801.95
									VYDESIQLDHK	2	N-Term(iTRAQ4plex); K11(iTRAQ4plex	0.871	5.28	55	3	545.62
									GVGGAPPTVTLLR	4	N-Term(iTRAQ4plex	0.828	4.85	61	2	691.42
									GDYSVLVPGLR	2	N-Term(iTRAQ4plex	0.828	4.69	66	2	660.38
									CNLDGSGLEVIDAMR	2	N-Term(iTRAQ4plex); C1(Methylthio	0.797	4.55	95	2	891.92
									TTLLAGDIEHPR	4	N-Term(iTRAQ4plex	0.953	4.26	71	3	489.61
									NLNAPVQPFEDPEHMK	2	N-Term(iTRAQ4plex); K16(iTRAQ4plex	0.866	3.91	41	3	718.70
									IETAAMDGTLR	2	N-Term(iTRAQ4plex	0.805	3.84	43	2	661.35
									IFFSDIHFGNIQQINDDGSR	2	N-Term(iTRAQ4plex	0.704	3.59	59	3	823.08
									NLFWTSYDTNK	1	N-Term(iTRAQ4plex); K11(iTRAQ4plex	0.904		67		
									ITIVENVGSVEGLAYHR	1	N-Term(iTRAQ4plex	0.699		59		
									ETVITMSGDDHPR	1	N-Term(iTRAQ4plex	1.104		43		
									ILWIDAR	1	N-Term(iTRAQ4plex	0.748		42		
									IVFPHGITLDLVSR	1	N-Term(iTRAQ4plex	0.8		42		
13129000	METRN	meteorin precursor	4.78	1	1	1	1.474	31.2								
									ALFLQATPHQDISR	1	N-Term(iTRAQ4plex	1.474		50		
11991660	SEMA6A	semaphorin-6A precursor	4.76	4	4	6	0.827	114.3								
									EIAVEYNTMGK	2	N-Term(iTRAQ4plex); K11(iTRAQ4plex	0.861	4.84	66	2	771.91
									SPDSTWTPVPDER	2	N-Term(iTRAQ4plex	0.767	4.52	37	2	815.90
									MDTLEPFGDEFSGMAR	1	N-Term(iTRAQ4plex	0.795		49		

									LTFEQDIER	1	N-Term(iTRAQ4plex	1.071		47		
156564357	NQO2	ribosyldihydronicotinamide dehydrogenase [quinone]	4.76	1	1	2	0.637	25.9								
									SLASDITDEQK	2	N-Term(iTRAQ4plex); K11(iTRAQ4plex	0.637	4.95	80	2	747.90
310114970	LOC100507703	PREDICTED: HLA class I histocompatibility antigen, A-69 alpha chain-like isoform 20	4.75	0	1	2		32.6								
									FIAVGYVDDTQFVF	2	N-Term(iTRAQ4plex		3.38	70	2	887.47
310124810	LOC100507703	PREDICTED: HLA class I histocompatibility antigen, A-69 alpha chain-like isoform 17	4.75	0	1	2		32.6								
									FIAVGYVDDTQFVF	2	N-Term(iTRAQ4plex		3.38	70	2	887.47
4557014	CAT	catalase	4.74	2	2	3	0.552	59.7								
									FSTVAGESGSADTVF	2	N-Term(iTRAQ4plex	0.523	3.44	61	2	814.41
									LCENIAGHLK	1	N-Term(iTRAQ4plex); C2(Methylthio); K10(iTRAQ4plex)	0.583		38		
4506113	PRNP	major prion protein preproprotein	4.74	1	1	12	0.983	27.6								
									VVEQMCITQYEF	12	N-Term(iTRAQ4plex); C6(Methylthio	0.983	5.26	64	2	844.90
310124991	LOC100507680	PREDICTED: HLA class I histocompatibility antigen, A-69 alpha chain-like isoform 11	4.73	0	1	2		34								
									FIAVGYVDDTQFVF	2	N-Term(iTRAQ4plex		3.38	70	2	887.47
7382460	SHBG	sex hormone-binding globulin isoform 1 precursor	4.73	0	2	4		43.8								
									QAEISASAPTSLR	3	N-Term(iTRAQ4plex		3.94	56	2	737.90
									DSWLDK	1	N-Term(iTRAQ4plex); K6(iTRAQ4plex			37		
14165276	C2orf40	augurin precursor	4.73	1	1	6	1.228	17.2								
									EFLGSLK	6	N-Term(iTRAQ4plex); K7(iTRAQ4plex	1.228	3.22	39	2	541.32
4557894	LYZ	lysozyme C precursor	4.73	1	1	1	0.754	16.5								
									YWCNDGK	1	N-Term(iTRAQ4plex); C3(Methylthio); K7(iTRAQ4plex)	0.754		53		
310118015	LOC100509457	PREDICTED: HLA class II histocompatibility antigen, DQ alpha 1 chain-like isoform 1	4.72	0	1	2		27.9								
									VEHWGLDEPLLK	2	N-Term(iTRAQ4plex); K12(iTRAQ4plex		3.45	47	3	575.32
54112392	CACNA2D2	voltage-dependent calcium channel subunit alpha-2/delta-2 isoform a	4.72	0	5	10		129.2								
									LADAAENFQK	4	N-Term(iTRAQ4plex); K10(iTRAQ4plex		4.83	67	2	697.88
									KIDLYDVR	3	N-Term(iTRAQ4plex); K1(iTRAQ4plex		3.36	46	3	437.26
									ADAELDDPESEDVER	1	N-Term(iTRAQ4plex			45		
54112394	CACNA2D2	voltage-dependent calcium channel subunit alpha-2/delta-2 isoform b	4.72	0	5	10		129								
									LADAAENFQK	4	N-Term(iTRAQ4plex); K10(iTRAQ4plex		4.83	67	2	697.88
									KIDLYDVR	3	N-Term(iTRAQ4plex); K1(iTRAQ4plex		3.36	46	3	437.26
									ADAELDDPESEDVER	1	N-Term(iTRAQ4plex			45		
52426774	HLA-DRA	HLA class II histocompatibility antigen, DR alpha chain precursor	4.72	0	1	2		28.6								
									VEHWGLDEPLLK	2	N-Term(iTRAQ4plex); K12(iTRAQ4plex		3.45	47	3	575.32
310125018	LOC100507718	PREDICTED: HLA class II histocompatibility antigen, DQ alpha 1 chain-like isoform 1	4.71	0	1	2		28.2								
									VEHWGLDEPLLK	2	N-Term(iTRAQ4plex); K12(iTRAQ4plex		3.45	47	3	575.32
310125249	LOC100507686	PREDICTED: HLA class II histocompatibility antigen, DQ alpha 1 chain-like isoform 1	4.71	0	1	2		28.1								
									VEHWGLDEPLLK	2	N-Term(iTRAQ4plex); K12(iTRAQ4plex		3.45	47	3	575.32
310133344	LOC100507718	PREDICTED: HLA class II histocompatibility antigen, DQ alpha 1 chain-like isoform 1	4.71	0	1	2		28.2								
									VEHWGLDEPLLK	2	N-Term(iTRAQ4plex); K12(iTRAQ4plex		3.45	47	3	575.32
11095447	HLA-DQA2	HLA class II histocompatibility antigen, DQ alpha 2 chain precursor	4.71	0	1	2		28								
									VEHWGLDEPLLK	2	N-Term(iTRAQ4plex); K12(iTRAQ4plex		3.45	47	3	575.32
291290994	CACNA2D2	voltage-dependent calcium channel subunit alpha-2/delta-2 isoform c	4.7	0	5	10		129.7								
									LADAAENFQK	4	N-Term(iTRAQ4plex); K10(iTRAQ4plex		4.83	67	2	697.88
									KIDLYDVR	3	N-Term(iTRAQ4plex); K1(iTRAQ4plex		3.36	46	3	437.26
									ADAELDDPESEDVER	1	N-Term(iTRAQ4plex			45		
4502601	CBR3	carbonyl reductase [NADPH] 3	4.69	0	1	2		30.8								
									FHQLDIDDLQSIR	2	N-Term(iTRAQ4plex		5.33	52	3	581.97
300360534	GSTO1	glutathione S-transferase omega-1 isoform 3	4.69	0	1	1		24.8								
									LNECVDHTPK	1	N-Term(iTRAQ4plex); C4(Methylthio); K10(iTRAQ4plex)			35		
310114968	LOC100507703	PREDICTED: HLA class I histocompatibility antigen, A-69 alpha chain-like isoform 11	4.68	0	1	2		34.3								
									FIAVGYVDDTQFVF	2	N-Term(iTRAQ4plex		3.38	70	2	887.47
13435364	DSC2	desmocollin-2 isoform Dsc2a preproprotein	4.66	0	3	8		99.9								
									VTVEDKDLVNTANWF	2	N-Term(iTRAQ4plex); K6(iTRAQ4plex		4.62	47	3	683.37
									IFVFLEHQTK	4	N-Term(iTRAQ4plex); K10(iTRAQ4plex		4.48	58	2	775.46
									TNEGVLCVVKPLNYEEK	2	N-Term(iTRAQ4plex); C7(Methylthio); K10(iTRAQ4plex); K17(iTRAQ4plex)		4.37	82	3	805.10
11761629	FGA	fibrinogen alpha chain isoform alpha preproprotein	4.66	0	3	4		69.7								
									VQHIQLLQK	2	N-Term(iTRAQ4plex); K9(iTRAQ4plex		3.71	50	3	465.63
									VPPEWK	1	N-Term(iTRAQ4plex); K6(iTRAQ4plex			38		
13186236	FGFR1	basic fibroblast growth factor receptor 1 isoform 4 precursor	4.65	0	3	8		81.8								
									IGPDNLPYVQILK	3	N-Term(iTRAQ4plex); K13(iTRAQ4plex		4.03	40	2	879.53
									VYSDPQPHIQWLK	2	N-Term(iTRAQ4plex); K13(iTRAQ4plex		3.51	44	3	633.68
									EMEVLHLR	3	N-Term(iTRAQ4plex		3.51	39	2	585.82
148005039	KIT	mast/stem cell growth factor receptor isoform 2 precursor	4.63	0	4	9		109.4								
									LVVQSSIDSSAFK	2	N-Term(iTRAQ4plex); K13(iTRAQ4plex		5.23	81	2	834.98
									YVSELHLTR	4	N-Term(iTRAQ4plex		4.28	49	2	631.35
									LLCTDPGFVK	2	N-Term(iTRAQ4plex); C3(Methylthio); K10(iTRAQ4plex)		3.25	54	2	713.89
									WTFEILDETNENK	1	N-Term(iTRAQ4plex); K13(iTRAQ4plex			34		
42794771	TXNDC5	thioredoxin domain-containing protein 5 isoform 1 precursor	4.63	0	2	2		47.6								
									EFPGLAGVK	1	N-Term(iTRAQ4plex); K9(iTRAQ4plex			57		
									TLAPTWEELSK	1	N-Term(iTRAQ4plex); K11(iTRAQ4plex			43		
4504111	GRB2	growth factor receptor-bound protein 2 isoform 1	4.61	0	1	2		25.2								
									ATADDELSFK	2	N-Term(iTRAQ4plex); K10(iTRAQ4plex		4.83	69	2	692.86

57165355	LPHN1	latrophilin-1 isoform 1 precursor	4.61	1	6	13	1.055	162.6								
									FEGTWETGYDK	2	N-Term(iTRAQ4plex); K11(iTRAQ4plex		5.08	81	2	810.89
									VDYAFNTNANF	4	N-Term(iTRAQ4plex		4.36	51	2	714.85
									VFVCPGTLQK	2	N-Term(iTRAQ4plex); C4(Methylthio); K10(iTRAQ4plex)	1.055	3.9	54	2	713.40
									AGLPFGLMR	3	N-Term(iTRAQ4plex		3.27	45	2	553.32
									TDTLTEYASWEDYVAAF	1	N-Term(iTRAQ4plex			43		
4557695	KIT	mast/stem cell growth factor receptor isoform 1 precursor	4.61	0	4	9		109.8								
									LVVQSSIDSSAFK	2	N-Term(iTRAQ4plex); K13(iTRAQ4plex		5.23	81	2	834.98
									YVSELHLTR	4	N-Term(iTRAQ4plex		4.28	49	2	631.35
									LLCTDPGFVK	2	N-Term(iTRAQ4plex); C3(Methylthio); K10(iTRAQ4plex)		3.25	54	2	713.89
									WTFEILDETNENK	1	N-Term(iTRAQ4plex); K13(iTRAQ4plex			34		
164698460	MANEAL	glycoprotein endo-alpha-1,2-mannosidase-like protein isoform 3	4.6	0	1	1		51.3								
									HSPPDDLGSSFYPELGPYSSR	1	N-Term(iTRAQ4plex			36		
15011918	ATP6AP2	renin receptor	4.57	2	2	2	0.905	39								
									ILVDALQK	1	N-Term(iTRAQ4plex); K8(iTRAQ4plex	0.983		53		
121674801	HEPACAM	hepatocyte cell adhesion molecule precursor	4.57	1	1	2	0.669	46								
									SATEPGPPGYSVSPAVPGF	2	N-Term(iTRAQ4plex	0.669	3.36	71	2	985.51
4557659	IDS	iduronate 2-sulfatase isoform a precursor	4.55	0	2	10		61.8								
									QSTEQAIQLLEK	4	N-Term(iTRAQ4plex); K12(iTRAQ4plex		5.99	75	2	838.48
									DLEEDPYLPGNPR	6	N-Term(iTRAQ4plex		4.66	68	2	829.91
13676857	HSPA2	heat shock-related 70 kDa protein 2	4.54	0	2	3		70								
									IINEPTAAAIAYGLDK	2	N-Term(iTRAQ4plex); K16(iTRAQ4plex		3.25	60	2	974.56
									TTPSYVAFTDTEF	1	N-Term(iTRAQ4plex			45		
310124989	LOC100507680	PREDICTED: HLA class I histocompatibility antigen, A-69 alpha chain-like isoform 17	4.53	0	1	2		34.2								
									FIAVGYVDDTQFVF	2	N-Term(iTRAQ4plex		3.38	70	2	887.47
4504943	LAIR1	leukocyte-associated immunoglobulin-like receptor 1 isoform a precursor	4.53	0	1	3		31.4								
									IDSVSEGNAGPYR	3	N-Term(iTRAQ4plex		5.07	80	2	754.88
194248072	HSPA1A	heat shock 70 kDa protein 1A/1B	4.52	0	2	5		70								
									ATAGDTHLGGEDFDNF	4	N-Term(iTRAQ4plex		5.18	77	3	607.28
									TTPSYVAFTDTEF	1	N-Term(iTRAQ4plex			45		
34419635	HSPA6	heat shock 70 kDa protein 6	4.51	0	2	5		71								
									ATAGDTHLGGEDFDNF	4	N-Term(iTRAQ4plex		5.18	77	3	607.28
									TTPSYVAFTDTEF	1	N-Term(iTRAQ4plex			45		
5730041	SUGT1	suppressor of G2 allele of SKP1 homolog isoform SGT1A	4.5	0	1	1		37.8								
									LELLHPIIPEQSTFK	1	N-Term(iTRAQ4plex); K15(iTRAQ4plex			37		
310114966	LOC100507703	PREDICTED: HLA class I histocompatibility antigen, A-69 alpha chain-like isoform 19	4.49	0	1	2		34.5								
									FIAVGYVDDTQFVF	2	N-Term(iTRAQ4plex		3.38	70	2	887.47
310124808	LOC100507703	PREDICTED: HLA class I histocompatibility antigen, A-69 alpha chain-like isoform 15	4.49	0	1	2		34.5								
									FIAVGYVDDTQFVF	2	N-Term(iTRAQ4plex		3.38	70	2	887.47
6631102	PCDH8	protocadherin-8 isoform 1 precursor	4.49	0	3	4		112.9								
									YSTFEEDAPGTVIGTLAEDLHMK	2	N-Term(iTRAQ4plex); K23(iTRAQ4plex		4.25	56	3	938.14
									LVHVEVEVF	1	N-Term(iTRAQ4plex			38		
112382370	FOLR3	folate receptor gamma precursor	4.49	0	1	1		27.9								
									LYNFNWDHCGK	1	N-Term(iTRAQ4plex); C9(Methylthio); K11(iTRAQ4plex)			35		
320118928	PCSK2	neuroendocrine convertase 2 isoform 2 preproprotein	4.48	0	3	5		66.3								
									KEELEEELDEAVER	2	N-Term(iTRAQ4plex); K1(iTRAQ4plex		4.7	55	3	669.34
									ELTLQAMADGVNK	2	N-Term(iTRAQ4plex); K13(iTRAQ4plex		3.62	66	2	839.46
									EELEEELDEAVER	1	N-Term(iTRAQ4plex			62		
18104957	MAG	myelin-associated glycoprotein isoform b precursor	4.47	0	2	3		63.9								
									SNPEPSVAFELPSR	2	N-Term(iTRAQ4plex		5.23	75	2	837.44
									SGLVLTSILTLR	1	N-Term(iTRAQ4plex			38		
157388900	IMPAD1	inositol monophosphatase 3	4.46	2	2	3	1.152	38.7								
									VLALLDVPDK	2	N-Term(iTRAQ4plex); K10(iTRAQ4plex	1.225	3.46	74	2	685.93
									MFYLLK	1	N-Term(iTRAQ4plex); K6(iTRAQ4plex	1.083		37		
310110547	LOC100509010	PREDICTED: heparan-sulfate 6-O-sulfotransferase 3-like	4.46	2	2	4	0.607	54.8								
									FVDFNIK	2	N-Term(iTRAQ4plex); K7(iTRAQ4plex	0.734		53		
									AGEAGPPAVPGPAF	2	N-Term(iTRAQ4plex	0.554		45		
109633039	PTPRF	receptor-type tyrosine-protein phosphatase F isoform 2 precursor	4.43	0	6	13		211.6								
									AAGTEGPFQEVDGVATTF	4	N-Term(iTRAQ4plex		6.08	110	2	975.48
									SDMGVGVFTPTIEAF	2	N-Term(iTRAQ4plex		5.07	87	2	862.44
									NVLELSNVVF	2	N-Term(iTRAQ4plex		4.07	38	2	643.88
									AHTDVGPGPESSPVLVF	2	N-Term(iTRAQ4plex		3.91	60	3	621.33
									YECVATNSAGTF	2	N-Term(iTRAQ4plex); C3(Methylthio		3.82	58	2	731.34
									IQLSWLLPPQER	1	N-Term(iTRAQ4plex			61		
7657039	TNFRSF21	tumor necrosis factor receptor superfamily member 21 precursor	4.43	2	2	12	0.775	71.8								
									TLPNLQVVNHQQGPHHF	4	N-Term(iTRAQ4plex	0.868	6.73	51	4	530.54
									GTFSDVPSSVMK	8	N-Term(iTRAQ4plex); K12(iTRAQ4plex	0.756	5.2	61	2	771.90
48255937	CD44	CD44 antigen isoform 2 precursor	4.43	0	3	72		76.6								
									YGFIEGHVVIPR	47	N-Term(iTRAQ4plex		4.96	83	2	765.93
									ALSIGFETCR	13	N-Term(iTRAQ4plex); C9(Methylthio		4.45	53	2	643.82
									FAGVFHVEK	12	N-Term(iTRAQ4plex); K9(iTRAQ4plex		3.55	79	2	661.38
94721252	VAPA	vesicle-associated membrane protein-associated protein A isoform 2	4.42	0	1	2		27.9								
									EAKPDELMDSK	2	N-Term(iTRAQ4plex); K3(iTRAQ4plex); K11(iTRAQ4plex)		3.7	46	3	565.63
296434255	VSIG4	V-set and immunoglobulin domain-containing protein 4 isoform 3 precursor	4.41	0	1	2		25.4								
									GSDPVTIFLR	2	N-Term(iTRAQ4plex		3.78	45	2	624.86
21536468	AXL	tyrosine-protein kinase receptor UFO isoform 2	4.41	0	3	23		97.3								
									APLQGTLLGYR	7	N-Term(iTRAQ4plex		4.89	56	2	666.89
									TATITVLPQQPF	12	N-Term(iTRAQ4plex		4.74	71	2	734.94
									CQLQVQGEPPEVHWLR	4	N-Term(iTRAQ4plex); C1(Methylthio		3.93	41	3	703.68

109633041	PTPRF	receptor-type tyrosine-protein phosphatase F isoform 1 precursor	4.4	0	6	13		212.7								
									AAGTEGPFQEVDGVATTI	4	N-Term(iTRAQ4plex		6.08	110	2	975.48
									SDMGVGVFTPTIEAF	2	N-Term(iTRAQ4plex		5.07	87	2	862.44
									NVLELSNVVR	2	N-Term(iTRAQ4plex		4.07	38	2	643.88
									AHTDVGPGPESSPVLVF	2	N-Term(iTRAQ4plex		3.91	60	3	621.33
									YECVATNSAGTF	2	N-Term(iTRAQ4plex); C3(Methylthio		3.82	58	2	731.34
									IQLSWLLPPQER	1	N-Term(iTRAQ4plex			61		
115430223	LGALS3	galectin-3 isoform 1	4.4	1	1	1	0.799	26.1								
									IQVLVEPDHFK	1	N-Term(iTRAQ4plex); K11(iTRAQ4plex	0.799		42		
167830475	MFGE8	lactadherin isoform a	4.39	0	2	3		43.1								
									EFVGNWNK	2	N-Term(iTRAQ4plex); K8(iTRAQ4plex		3.42	41	2	641.35
									ILPVAWHNR	1	N-Term(iTRAQ4plex			37		
4502693	CD9	CD9 antigen	4.39	1	1	3	1.097	25.4								
									KDVLETFTVK	3	N-Term(iTRAQ4plex); K1(iTRAQ4plex); K10(iTRAQ4plex)	1.097	3.77	53	3	538.00
310110104	LOC100293160	PREDICTED: peptidyl-prolyl cis-trans isomerase A-like, partial	4.38	0	1	3		17.4								
									TEWLDGK	3	N-Term(iTRAQ4plex); K7(iTRAQ4plex		3.28	47	2	568.82
310120620	LOC100293160	PREDICTED: peptidyl-prolyl cis-trans isomerase A-like, partial	4.38	0	1	3		17.4								
									TEWLDGK	3	N-Term(iTRAQ4plex); K7(iTRAQ4plex		3.28	47	2	568.82
31543806	THBS4	thrombospondin-4 precursor	4.37	3	3	5	0.903	105.8								
									AFAGPSQKPETIELR	2	N-Term(iTRAQ4plex); K8(iTRAQ4plex	0.891	4.16	67	2	966.55
									KPQDFLEELK	2	N-Term(iTRAQ4plex); K1(iTRAQ4plex); K10(iTRAQ4plex)	0.938		53		
									DVDIDSYPDEELPCSAR	1	N-Term(iTRAQ4plex); C14(Methylthio	0.691		37		
222144233	FGFR2	fibroblast growth factor receptor 2 isoform 4 precursor	4.37	0	3	8		79.8								
									TVLIGEYLQIK	2	N-Term(iTRAQ4plex); K11(iTRAQ4plex		4.34	58	2	782.98
									DAAVISWTK	4	N-Term(iTRAQ4plex); K9(iTRAQ4plex		4.25	66	2	639.87
									DSGLYACTASR	2	N-Term(iTRAQ4plex); C7(Methylthio		3.66	69	2	667.30
21536466	AXL	tyrosine-protein kinase receptor UFO isoform 1	4.36	0	3	23		98.3								
									APLQGTLLGYR	7	N-Term(iTRAQ4plex		4.89	56	2	666.89
									TATITVLPQQPF	12	N-Term(iTRAQ4plex		4.74	71	2	734.94
									CQLQVQGEPPEVHWLR	4	N-Term(iTRAQ4plex); C1(Methylthio		3.93	41	3	703.68
320118926	PCSK2	neuroendocrine convertase 2 isoform 3	4.36	0	3	5		68.5								
									KEELEEELDEAVER	2	N-Term(iTRAQ4plex); K1(iTRAQ4plex		4.7	55	3	669.34
									ELTLQAMADGVNK	2	N-Term(iTRAQ4plex); K13(iTRAQ4plex		3.62	66	2	839.46
									EELEEELDEAVER	1	N-Term(iTRAQ4plex			62		
40317626	THBS1	thrombospondin-1 precursor	4.36	2	4	6	0.844	129.3								
									NALWHTGNTPGQVF	2	N-Term(iTRAQ4plex		4.16	46	3	565.63
									FTGSQPFGQGVEHATANK	2	N-Term(iTRAQ4plex); K18(iTRAQ4plex	0.886	4.11	41	3	722.04
									FYVVMWK	1	N-Term(iTRAQ4plex); K7(iTRAQ4plex			43		
									FVFGTTPEDILR	1	N-Term(iTRAQ4plex	0.804		42		
4759036	RGN	regucalcin	4.35	1	1	1	0.501	33.2								
									VTMDAPVSSVALF	1	N-Term(iTRAQ4plex	0.501		38		
310124985	LOC100507680	PREDICTED: HLA class I histocompatibility antigen, A-69 alpha chain-like isoform 7	4.33	0	1	2		36.6								
									FIAVGYVDDTQFVF	2	N-Term(iTRAQ4plex		3.38	70	2	887.47
312836851	MAG	myelin-associated glycoprotein isoform c	4.33	0	2	3		66.2								
									SNPEPSVAFELPSR	2	N-Term(iTRAQ4plex		5.23	75	2	837.44
									SGLVLTSILTLR	1	N-Term(iTRAQ4plex			38		
4505763	PGK1	phosphoglycerate kinase 1	4.32	2	2	3	0.342	44.6								
									ALMDEVVK	2	N-Term(iTRAQ4plex); K8(iTRAQ4plex	0.343	3.17	54	2	596.85
									IQLINNMLDK	1	N-Term(iTRAQ4plex); K10(iTRAQ4plex	0.341		76		
310125001	LOC100507680	PREDICTED: HLA class I histocompatibility antigen, A-69 alpha chain-like isoform 16, partial	4.31	0	1	2		36.1								
									FIAVGYVDDTQFVF	2	N-Term(iTRAQ4plex		3.38	70	2	887.47
223029449	NPDC1	neural proliferation differentiation and control protein 1 precursor	4.31	1	1	4	1.056	34.5								
									LEDEIDFLAQELAR	4	N-Term(iTRAQ4plex	1.056	5.57	88	2	903.48
310114962	LOC100507703	PREDICTED: HLA class I histocompatibility antigen, A-69 alpha chain-like isoform 9	4.29	0	1	2		36.9								
									FIAVGYVDDTQFVF	2	N-Term(iTRAQ4plex		3.38	70	2	887.47
116014339	OLFML2A	olfactomedin-like protein 2A precursor	4.29	3	3	8	0.776	73								
									LDPGDLSVHR	5	N-Term(iTRAQ4plex	0.776	3.69	57	3	418.23
									DEAQPEVIVLSR	2	N-Term(iTRAQ4plex	0.792	3.65	65	2	750.42
									WSNMYK	1	N-Term(iTRAQ4plex); K6(iTRAQ4plex	0.688		36		
228480207	FHL1	four and a half LIM domains protein 1 isoform 2	4.29	0	1	2		31.9								
									AIVAGDQNVEYK	2	N-Term(iTRAQ4plex); K12(iTRAQ4plex		4.31	51	2	797.94
28269693	XYLT1	xylosyltransferase 1	4.28	3	3	7	0.861	107.5								
									GGAAVGGGEQPPPAPAPF	4	N-Term(iTRAQ4plex	0.721	3.92	85	2	865.46
									DFENVDNSNFAPF	2	N-Term(iTRAQ4plex	0.964	3.23	50	2	834.89
									IASPPSDFGR	1	N-Term(iTRAQ4plex	0.996		58		
170296790	PRSS3	trypsin-3 isoform 1 preproprotein	4.28	0	1	2		32.5								
									VLEGNEQFINAAK	2	N-Term(iTRAQ4plex); K13(iTRAQ4plex		6.38	67	2	860.98
221139811	PPIAL4E	peptidylprolyl isomerase A (cyclophilin A)-like 4E	4.27	0	1	3		18.2								
									TEWLDGK	3	N-Term(iTRAQ4plex); K7(iTRAQ4plex		3.28	47	2	568.82
178057341	PPIAL4G	peptidylprolyl cis-trans isomerase A-like 4G	4.27	0	1	3		18.2								
									TEWLDGK	3	N-Term(iTRAQ4plex); K7(iTRAQ4plex		3.28	47	2	568.82
21361837	PITHD1	chromosome 1 open reading frame 128	4.27	1	1	3	0.795	24.2								
									GLAYGLYLR	3	N-Term(iTRAQ4plex	0.795	3.45	47	2	585.35
6598323	GDI2	rab GDP dissociation inhibitor beta isoform 1	4.27	0	2	2		50.6								
									VICILSHPIK	1	N-Term(iTRAQ4plex); C3(Methylthio); K10(iTRAQ4plex)			43		
310124806	LOC100507703	PREDICTED: HLA class I histocompatibility antigen, A-69 alpha chain-like isoform 16	4.26	0	1	2		36.5								
									FIAVGYVDDTQFVF	2	N-Term(iTRAQ4plex		3.38	70	2	887.47
4505683	PDGFRB	beta-type platelet-derived growth factor receptor precursor	4.25	3	3	6	0.786	123.9								
									TLGDSSAGEIALSTF	2	N-Term(iTRAQ4plex	0.844	5.33	51	2	811.43
									LLGEVGTLQFAELHR	2	N-Term(iTRAQ4plex	0.74	3.9	46	3	609.68
									VLELSESHPDSGEQTVF	2	N-Term(iTRAQ4plex	0.786	3.79	54	3	676.34
9966883	PCDH9	protocadherin-9 isoform 2 precursor	4.24	0	4	9		132.2								
									EQQSSYTFDVK	2	N-Term(iTRAQ4plex); K11(iTRAQ4plex		5.73	73	2	810.41

									YIFGAQVAPATK	2	N-Term(iTRAQ4plex); K12(iTRAQ4plex		4.05	86	2	777.45
									VTVLASDGSSTPAF	4	N-Term(iTRAQ4plex		3.77	78	2	752.91
153791670	KRT76	keratin, type II cytoskeletal 2 oral	4.23	0	3	6		65.8								
									FASFIDK	4	N-Term(iTRAQ4plex); K7(iTRAQ4plex		3.19	53	2	558.31
20336244	PCSK2	neuroendocrine convertase 2 isoform 1 preproprotein	4.23	0	3	5		70.5								
									KEELEEELDEAVER	2	N-Term(iTRAQ4plex); K1(iTRAQ4plex		4.7	55	3	669.34
									ELTLQAMADGVNK	2	N-Term(iTRAQ4plex); K13(iTRAQ4plex		3.62	66	2	839.46
									EELEEELDEAVER	1	N-Term(iTRAQ4plex			62		
48255935	CD44	CD44 antigen isoform 1 precursor	4.18	0	3	72		81.5								
									YGFIEGHVVIPR	47	N-Term(iTRAQ4plex		4.96	83	2	765.93
									ALSIGFETCR	13	N-Term(iTRAQ4plex); C9(Methylthio		4.45	53	2	643.82
									FAGVFHVEK	12	N-Term(iTRAQ4plex); K9(iTRAQ4plex		3.55	79	2	661.38
310114976	LOC100507703	PREDICTED: HLA class I histocompatibility antigen, A-69 alpha chain-like isoform 16	4.15	0	1	2		38								
									FIAVGYVDDTQFVF	2	N-Term(iTRAQ4plex		3.38	70	2	887.47
310124816	LOC100507703	PREDICTED: HLA class I histocompatibility antigen, A-69 alpha chain-like isoform 13	4.15	0	1	2		38								
									FIAVGYVDDTQFVF	2	N-Term(iTRAQ4plex		3.38	70	2	887.47
11225258	MAG	myelin-associated glycoprotein isoform a precursor	4.15	0	2	3		69								
									SNPEPSVAFELPSR	2	N-Term(iTRAQ4plex		5.23	75	2	837.44
									SGLVLTSILTLR	1	N-Term(iTRAQ4plex			38		
49574491	ATP1B2	sodium/potassium-transporting ATPase subunit beta-2	4.14	1	1	2	1.051	33.3								
									INAANIATDDEF	2	N-Term(iTRAQ4plex	1.051	4.28	70	2	723.87
13027602	DDRGK1	DDRGK domain-containing protein 1 precursor	4.14	1	1	1	0.905	35.6								
									VAQPGPLEPEEPR	1	N-Term(iTRAQ4plex	0.905		49		
307574691	TGFBR3	transforming growth factor beta receptor type 3 isoform b	4.12	0	3	7		93.4								
									KEYGAVTSFTELK	2	N-Term(iTRAQ4plex); K1(iTRAQ4plex); K13(iTRAQ4plex)		3.87	55	3	635.69
									SIRDDIPSTQGNLVK	2	N-Term(iTRAQ4plex); K15(iTRAQ4plex		3.87	42	3	644.36
									NLILILK	3	N-Term(iTRAQ4plex); K7(iTRAQ4plex		3.28	54	2	557.90
45243534	PCDH9	protocadherin-9 isoform 1 precursor	4.12	0	4	9		136								
									EQQSSYTFDVK	2	N-Term(iTRAQ4plex); K11(iTRAQ4plex		5.73	73	2	810.41
									YIFGAQVAPATK	2	N-Term(iTRAQ4plex); K12(iTRAQ4plex		4.05	86	2	777.45
									VTVLASDGSSTPAF	4	N-Term(iTRAQ4plex		3.77	78	2	752.91
5031873	LMAN1	protein ERGIC-53 precursor	4.12	1	1	1	1.052	57.5								
									GAGMPGQHGQITQQELDTVVI	1	N-Term(iTRAQ4plex); M4(Oxidation); K21(iTRAQ4plex)	1.052		69		
56682966	TGFBR3	transforming growth factor beta receptor type 3 isoform a	4.11	0	3	7		93.4								
									KEYGAVTSFTELK	2	N-Term(iTRAQ4plex); K1(iTRAQ4plex); K13(iTRAQ4plex)		3.87	55	3	635.69
									SIRDDIPSTQGNLVK	2	N-Term(iTRAQ4plex); K15(iTRAQ4plex		3.87	42	3	644.36
									NLILILK	3	N-Term(iTRAQ4plex); K7(iTRAQ4plex		3.28	54	2	557.90
195963398	SUGT1	suppressor of G2 allele of SKP1 homolog isoform SGT1B	4.11	0	1	1		41								
									LELLHPIIPEQSTFK	1	N-Term(iTRAQ4plex); K15(iTRAQ4plex			37		
310125083	LOC100507680	PREDICTED: HLA class I histocompatibility antigen, A-43 alpha chain-like isoform 18	4.1	0	1	1		27.3								
									DYIALNEDLR	2	N-Term(iTRAQ4plex		3.38	41	2	683.36
5803227	YWHAQ	14-3-3 protein theta	4.08	0	1	2		27.7								
									DSTLIMQLLR	2	N-Term(iTRAQ4plex		4.44	67	2	667.39
310124913	LOC100507680	PREDICTED: HLA class I histocompatibility antigen, A-32 alpha chain-like isoform 10	4.08	0	1	1		27.3								
									DYIALNEDLR	2	N-Term(iTRAQ4plex		3.38	41	2	683.36
310125188	LOC100507680	PREDICTED: HLA class I histocompatibility antigen, A-74 alpha chain-like isoform 9	4.08	0	1	1		27.3								
									DYIALNEDLR	2	N-Term(iTRAQ4plex		3.38	41	2	683.36
4505891	PLOD3	procollagen-lysine,2-oxoglutarate 5-dioxygenase 3 precursor	4.07	2	2	3	0.961	84.7								
									LVGPEEALSPGEAR	2	N-Term(iTRAQ4plex	0.928	5.74	82	2	784.93
4507951	YWHAH	14-3-3 protein eta	4.07	0	1	2		28.2								
									DSTLIMQLLR	2	N-Term(iTRAQ4plex		4.44	67	2	667.39
117306169	PRCP	lysosomal Pro-X carboxypeptidase isoform 2 preproprotein	4.06	0	2	4		58.1								
									AMLVFAEHR	2	N-Term(iTRAQ4plex		3.42	48	3	406.56
									NALDPMSVLLAR	2	N-Term(iTRAQ4plex		3.36	55	2	722.41
310124983	LOC100507680	PREDICTED: HLA class I histocompatibility antigen, A-69 alpha chain-like isoform 8	4.05	0	1	2		38.8								
									FIAVGYVDDTQFVF	2	N-Term(iTRAQ4plex		3.38	70	2	887.47
228480205	FHL1	four and a half LIM domains protein 1 isoform 5	4.05	0	1	2		33.6								
									AIVAGDQNVEYK	2	N-Term(iTRAQ4plex); K12(iTRAQ4plex		4.31	51	2	797.94
31317256	NLGN4X	neuroligin-4, X-linked precursor	4.04	0	3	7		91.9								
									FEEVAWSK	4	N-Term(iTRAQ4plex); K8(iTRAQ4plex		3.67	47	2	642.36
									GNYGLLDQIQALR	2	N-Term(iTRAQ4plex		3.18	52	2	802.95
18641360	COLEC12	collectin-12	4.04	3	3	10	1.017	81.5								
									ESHWIGLTDSER	2	N-Term(iTRAQ4plex	1.08	4.73	40	2	787.39
									WLDGTSPDYK	7	N-Term(iTRAQ4plex); K10(iTRAQ4plex	1.135	4.13	59	2	735.38
									CYYFSVEK	1	N-Term(iTRAQ4plex); C1(Methylthio); K8(iTRAQ4plex)	0.934		45		
256222771	NLGN4Y	neuroligin-4, Y-linked isoform 1	4.04	0	3	7		92								
									FEEVAWSK	4	N-Term(iTRAQ4plex); K8(iTRAQ4plex		3.67	47	2	642.36
									GNYGLLDQIQALR	2	N-Term(iTRAQ4plex		3.18	52	2	802.95
4585710	ADAM11	disintegrin and metalloproteinase domain-containing protein 11 preproprotein	4.03	2	2	3	0.902	83.4								
									IQVQDDLLETLAR	2	N-Term(iTRAQ4plex	0.798	4.43	87	2	829.47
									LGDLVGDISSVTFYHQGK	1	N-Term(iTRAQ4plex); K18(iTRAQ4plex	1.02		34		
221316575	CD58	lymphocyte function-associated antigen 3 isoform 2	4.03	0	1	2		27.9								
									VAELENSEFR	2	N-Term(iTRAQ4plex		3.84	51	2	669.35
5454052	SFN	14-3-3 protein sigma	4.03	0	1	2		27.8								
									DSTLIMQLLR	2	N-Term(iTRAQ4plex		4.44	67	2	667.39
310124999	LOC100507680	PREDICTED: HLA class I histocompatibility antigen, A-69 alpha chain-like isoform 19	4.02	0	1	2		39.3								
									FIAVGYVDDTQFVF	2	N-Term(iTRAQ4plex		3.38	70	2	887.47

255918079	VCAN	versican core protein isoform 4 precursor	4.02	0	6	20		181.9								
									VSVPTHPEAVGDASLTVVI	9	N-Term(iTRAQ4plex); K19(iTRAQ4plex		7.91	94	3	732.08
									LATVGELQAAWR	2	N-Term(iTRAQ4plex		5.48	55	2	729.91
									LLASDAGLYR	2	N-Term(iTRAQ4plex		4.01	45	2	611.85
									FTFEEAAK	2	N-Term(iTRAQ4plex); K8(iTRAQ4plex		3.58	59	2	615.83
									LDISEIK	4	N-Term(iTRAQ4plex); K7(iTRAQ4plex		3.23	56	2	553.33
									TEEEVFSGMK	1	N-Term(iTRAQ4plex); K10(iTRAQ4plex			64		
310114960	LOC100507703	PREDICTED: HLA class I histocompatibility antigen, A-69 alpha chain-like isoform 12	4.01	0	1	2		39.1								
									FIAVGYVDDTQFVF	2	N-Term(iTRAQ4plex		3.38	70	2	887.47
310124804	LOC100507703	PREDICTED: HLA class I histocompatibility antigen, A-69 alpha chain-like isoform 8	4.01	0	1	2		39.1								
									FIAVGYVDDTQFVF	2	N-Term(iTRAQ4plex		3.38	70	2	887.47
11386147	PSAP	proactivator polypeptide isoform a preproprotein	4.01	0	2	7		58.1								
									EMPMQTLVPAK	5	N-Term(iTRAQ4plex); K11(iTRAQ4plex		4.52	50	2	766.93
									GSAVWCQNVK	2	N-Term(iTRAQ4plex); C6(Methylthio); K10(iTRAQ4plex)		4.05	55	2	713.36
30520331	LAYN	layilin	4.01	1	1	1	0.848	42.3								
									DGGQLVSIESEDEQK	1	N-Term(iTRAQ4plex); K15(iTRAQ4plex	0.848		46		
4502677	CD58	lymphocyte function-associated antigen 3 isoform 1	4	0	1	2		28.1								
									VAELENSEFR	2	N-Term(iTRAQ4plex		3.84	51	2	669.35
24234699	KRT19	keratin, type I cytoskeletal 19	4	0	2	6		44.1								
									LEQEIATYR	2	N-Term(iTRAQ4plex		4.13	44	2	633.84
									LASYLDK	4	N-Term(iTRAQ4plex); K7(iTRAQ4plex		3.35	53	2	549.32
189571583	ICAM5	intercellular adhesion molecule 5 precursor	4	3	3	7	0.743	97.1								
									SGELGAVIEGLLR	3	N-Term(iTRAQ4plex	0.737	4.17	83	2	729.43
									NVAVTVEYGPF	2	N-Term(iTRAQ4plex	0.726	3.57	67	2	674.87
									SWTWPEGPEQTLR	2	N-Term(iTRAQ4plex	0.773	3.16	52	2	865.94
38327632	DDR1	epithelial discoidin domain-containing receptor 1 isoform DDR1a	4	0	3	6		97.1								
									GPAMAWEGEPMR	3	N-Term(iTRAQ4plex		4.9	39	2	738.35
									LHLVALVGTQGF	2	N-Term(iTRAQ4plex		4.32	40	2	704.43
20149498	FTL	ferritin light chain	4	1	1	1	0.687	20								
									ALFQDIK	1	N-Term(iTRAQ4plex); K7(iTRAQ4plex	0.687		40		
310124814	LOC100507703	PREDICTED: HLA class I histocompatibility antigen, A-69 alpha chain-like isoform 11	3.99	0	1	2		39.6								
									FIAVGYVDDTQFVF	2	N-Term(iTRAQ4plex		3.38	70	2	887.47
110224479	PSAP	proactivator polypeptide isoform c preproprotein	3.99	0	2	7		58.3								
									EMPMQTLVPAK	5	N-Term(iTRAQ4plex); K11(iTRAQ4plex		4.52	50	2	766.93
									GSAVWCQNVK	2	N-Term(iTRAQ4plex); C6(Methylthio); K10(iTRAQ4plex)		4.05	55	2	713.36
110224476	PSAP	proactivator polypeptide isoform b preproprotein	3.98	0	2	7		58.4								
									EMPMQTLVPAK	5	N-Term(iTRAQ4plex); K11(iTRAQ4plex		4.52	50	2	766.93
									GSAVWCQNVK	2	N-Term(iTRAQ4plex); C6(Methylthio); K10(iTRAQ4plex)		4.05	55	2	713.36
30794472	HAPLN4	hyaluronan and proteoglycan link protein 4 precursor	3.98	1	1	2	0.775	42.8								
									AELQGDGPGDASLVLR	2	N-Term(iTRAQ4plex	0.775	3.18	75	2	871.46
10863909	SPINT2	kunitz-type protease inhibitor 2 isoform a precursor	3.97	1	1	1	1.043	28.2								
									SIHDFCLVSK	1	N-Term(iTRAQ4plex); C6(Methylthio); K10(iTRAQ4plex)	1.043		36		
211938442	APOL1	apolipoprotein L1 isoform c precursor	3.95	0	1	2		42.1								
									VTEPISAESGEQVER	2	N-Term(iTRAQ4plex		4.51	84	2	887.95
41281557	LPHN1	latrophilin-1 isoform 2 precursor	3.95	0	5	11		162								
									FEGTWETGYDK	2	N-Term(iTRAQ4plex); K11(iTRAQ4plex		5.08	81	2	810.89
									VDYAFNTNANF	4	N-Term(iTRAQ4plex		4.36	51	2	714.85
									AGLPFGLMR	3	N-Term(iTRAQ4plex		3.27	45	2	553.32
									TDTLTEYASWEDYVAAF	1	N-Term(iTRAQ4plex			43		
310124997	LOC100507680	PREDICTED: HLA class I histocompatibility antigen, A-69 alpha chain-like isoform 20, partial	3.93	0	1	2		39.8								
									FIAVGYVDDTQFVF	2	N-Term(iTRAQ4plex		3.38	70	2	887.47
214830438	SQSTM1	sequestosome-1 isoform 2	3.93	0	1	3		38.6								
									LTPVSPESSSTEEK	3	N-Term(iTRAQ4plex); K14(iTRAQ4plex		4.69	97	2	889.97
171460922	TMEM132D	transmembrane protein 132D precursor	3.91	4	4	7	0.814	122.2								
									KGDVLTFPVSISR	2	N-Term(iTRAQ4plex); K1(iTRAQ4plex	0.914	4.88	46	3	569.67
									ASSPSIWDVK	2	N-Term(iTRAQ4plex); K10(iTRAQ4plex	0.846	3.71	44	2	689.39
									VESFLIYK	2	N-Term(iTRAQ4plex); K8(iTRAQ4plex	0.784	3.55	64	2	643.89
									DFSLMATSLDEK	1	N-Term(iTRAQ4plex); K12(iTRAQ4plex	0.753		36		
310124812	LOC100507703	PREDICTED: HLA class I histocompatibility antigen, A-69 alpha chain-like isoform 12, partial	3.9	0	1	2		40.1								
									FIAVGYVDDTQFVF	2	N-Term(iTRAQ4plex		3.38	70	2	887.47
312433998	LTF	lactotransferrin isoform 2	3.9	0	2	4		73.1								
									LRPVAAEVYGTEF	2	N-Term(iTRAQ4plex		5.81	44	3	535.63
									CLAENAGDVAFVK	2	N-Term(iTRAQ4plex); C1(Methylthio); K13(iTRAQ4plex)		4.36	69	2	835.93
310114540	LOC256374	PREDICTED: peptidyl-prolyl cis-trans isomerase A-like	3.89	0	1	3		19.8								
									TEWLDGK	3	N-Term(iTRAQ4plex); K7(iTRAQ4plex		3.28	47	2	568.82
4885509	NBL1	neuroblastoma suppressor of tumorigenicity 1 isoform 2 precursor	3.89	0	1	27		19.3								
									LALFPDK	27	N-Term(iTRAQ4plex); K7(iTRAQ4plex			44		
228480209	FHL1	four and a half LIM domains protein 1 isoform 3	3.88	0	1	2		35								
									AIVAGDQNVEYK	2	N-Term(iTRAQ4plex); K12(iTRAQ4plex		4.31	51	2	797.94
310124975	LOC100507680	PREDICTED: HLA class I histocompatibility antigen, A-69 alpha chain-like isoform 1	3.87	0	1	2		40.6								
									FIAVGYVDDTQFVF	2	N-Term(iTRAQ4plex		3.38	70	2	887.47
310114972	LOC100507703	PREDICTED: HLA class I histocompatibility antigen, A-69 alpha chain-like isoform 14, partial	3.86	0	1	2		40.5								
									FIAVGYVDDTQFVF	2	N-Term(iTRAQ4plex		3.38	70	2	887.47

310114948	LOC100507703	PREDICTED: HLA class I histocompatibility antigen, A-69 alpha chain-like isoform 1	3.84	0	1	2		40.9								
									FIAVGYVDDTQFVF	2	N-Term(iTRAQ4plex		3.38	70	2	887.47
310114974	LOC100507703	PREDICTED: HLA class I histocompatibility antigen, A-69 alpha chain-like isoform 15, partial	3.84	0	1	2		40.7								
									FIAVGYVDDTQFVF	2	N-Term(iTRAQ4plex		3.38	70	2	887.47
310124792	LOC100507703	PREDICTED: HLA class I histocompatibility antigen, A-69 alpha chain-like isoform 1	3.84	0	1	2		40.9								
									FIAVGYVDDTQFVF	2	N-Term(iTRAQ4plex		3.38	70	2	887.47
310124979	LOC100507680	PREDICTED: HLA class I histocompatibility antigen, A-69 alpha chain-like isoform 2	3.84	0	1	2		40.7								
									FIAVGYVDDTQFVF	2	N-Term(iTRAQ4plex		3.38	70	2	887.47
112380628	LAMP1	lysosome-associated membrane glycoprotein 1 precursor	3.84	2	2	7	1.077	44.9								
									TVESITDIR	4	N-Term(iTRAQ4plex	1.037	3.82	41	2	589.33
									VWVQAFK	3	N-Term(iTRAQ4plex); K7(iTRAQ4plex	1.077	3.29	43	2	583.34
67782365	KRT7	keratin, type II cytoskeletal 7	3.84	0	2	8		51.4								
									LALDIEIATYR	5	N-Term(iTRAQ4plex		3.43	51	2	711.41
									FASFIDK	4	N-Term(iTRAQ4plex); K7(iTRAQ4plex		3.19	53	2	558.31
83977450	DDR1	epithelial discoidin domain-containing receptor 1 isoform DDR1b	3.83	0	3	6		101.1								
									GPAMAWEGEPMR	3	N-Term(iTRAQ4plex		4.9	39	2	738.35
									LHLVALVGTQGF	2	N-Term(iTRAQ4plex		4.32	40	2	704.43
56682959	FTH1	ferritin heavy chain	3.83	1	1	1	0.749	21.2								
									IFLQDIK	1	N-Term(iTRAQ4plex); K7(iTRAQ4plex	0.749		43		
195546915	FAM69C	hypothetical protein LOC125704	3.82	2	2	3	1.314	46.4								
									AASSVFWK	2	N-Term(iTRAQ4plex); K8(iTRAQ4plex	1.48	3.42	53	2	592.33
									HWFSAPLK	1	N-Term(iTRAQ4plex); K8(iTRAQ4plex	1.166		36		
150378552	SUSD5	sushi domain-containing protein 5 precursor	3.82	2	2	3	0.854	68								
									VIYGNTDGPSGPFVGK	2	N-Term(iTRAQ4plex); K16(iTRAQ4plex	0.791	5.02	57	2	948.51
									LVSISVGR	1	N-Term(iTRAQ4plex	0.921		48		
131412228	KRT13	keratin, type I cytoskeletal 13 isoform b	3.81	0	2	3		45.8								
									LEQEIATYR	2	N-Term(iTRAQ4plex		4.13	44	2	633.84
									LASYLEK	1	N-Term(iTRAQ4plex); K7(iTRAQ4plex			46		
83977452	DDR1	epithelial discoidin domain-containing receptor 1 isoform DDR1c	3.81	0	3	6		101.7								
									GPAMAWEGEPMR	3	N-Term(iTRAQ4plex		4.9	39	2	738.35
									LHLVALVGTQGF	2	N-Term(iTRAQ4plex		4.32	40	2	704.43
5453958	PPP5C	serine/threonine-protein phosphatase 5	3.81	1	1	1	1.022	56.8								
									GNHETDNMNQIYGFEGEVI	1	N-Term(iTRAQ4plex); K19(iTRAQ4plex	1.022		48		
310114956	LOC100507703	PREDICTED: HLA class I histocompatibility antigen, A-69 alpha chain-like isoform 3	3.8	0	1	2		41								
									FIAVGYVDDTQFVF	2	N-Term(iTRAQ4plex		3.38	70	2	887.47
310124802	LOC100507703	PREDICTED: HLA class I histocompatibility antigen, A-69 alpha chain-like isoform 4	3.8	0	1	2		41								
									FIAVGYVDDTQFVF	2	N-Term(iTRAQ4plex		3.38	70	2	887.47
310124973	LOC100507680	PREDICTED: HLA class I histocompatibility antigen, A-69 alpha chain-like isoform 4	3.8	0	1	2		41.2								
									FIAVGYVDDTQFVF	2	N-Term(iTRAQ4plex		3.38	70	2	887.47
310124977	LOC100507680	PREDICTED: HLA class I histocompatibility antigen, A-69 alpha chain-like isoform 3	3.8	0	1	2		41.1								
									FIAVGYVDDTQFVF	2	N-Term(iTRAQ4plex		3.38	70	2	887.47
296179444	SEZ6L	seizure 6-like protein isoform 6 precursor	3.8	0	3	5		103.2								
									SPTNTISVYFR	2	N-Term(iTRAQ4plex		4.83	67	2	714.88
									IMYCTDPGEVDHSTF	2	N-Term(iTRAQ4plex); C4(Methylthio		4.69	52	3	638.61
									ETGTPIWTSR	1	N-Term(iTRAQ4plex			37		
5031701	FSTL3	follistatin-related protein 3 precursor	3.8	1	1	1	0.796	27.6								
									GHPDLSVMYR	1	N-Term(iTRAQ4plex	0.796		49		
310113396	LOC100133944	PREDICTED: IgGFc-binding protein-like	3.79	0	9	20		317.9								
									VTASSPVAVLSGHSCAQI	2	N-Term(iTRAQ4plex); C15(Methylthio); K18(iTRAQ4plex)		8.09	65	3	692.70
									AISGLTIDGHAVGAK	3	N-Term(iTRAQ4plex); K15(iTRAQ4plex		6.6	100	2	849.50
									VVAEVQICHGK	4	N-Term(iTRAQ4plex); C8(Methylthio); K11(iTRAQ4plex)		5.98	89	3	506.28
									GEVGFVLVDNQF	2	N-Term(iTRAQ4plex		4.95	78	2	738.90
									VAYDLVYYVR	3	N-Term(iTRAQ4plex		4.18	38	2	702.89
									AIGYATAADCGF	2	N-Term(iTRAQ4plex); C10(Methylthio		3.84	74	2	679.82
									FYPAGDVLR	2	N-Term(iTRAQ4plex		3.18	38	2	591.32
									SVPGCEGVALVVAQTK	1	N-Term(iTRAQ4plex); C5(Methylthio); K16(iTRAQ4plex)			50		
									LPVVLANGQIR	1	N-Term(iTRAQ4plex			48		
310132731	LOC100290309	PREDICTED: IgGFc-binding protein-like	3.79	0	9	20		317.8								
									VTASSPVAVLSGHSCAQI	2	N-Term(iTRAQ4plex); C15(Methylthio); K18(iTRAQ4plex)		8.09	65	3	692.70
									AISGLTIDGHAVGAK	3	N-Term(iTRAQ4plex); K15(iTRAQ4plex		6.6	100	2	849.50
									VVAEVQICHGK	4	N-Term(iTRAQ4plex); C8(Methylthio); K11(iTRAQ4plex)		5.98	89	3	506.28
									GEVGFVLVDNQF	2	N-Term(iTRAQ4plex		4.95	78	2	738.90
									VAYDLVYYVR	3	N-Term(iTRAQ4plex		4.18	38	2	702.89
									AIGYATAADCGF	2	N-Term(iTRAQ4plex); C10(Methylthio		3.84	74	2	679.82
									FYPAGDVLR	2	N-Term(iTRAQ4plex		3.18	38	2	591.32
									SVPGCEGVALVVAQTK	1	N-Term(iTRAQ4plex); C5(Methylthio); K16(iTRAQ4plex)			50		
									LPVVLANGQIR	1	N-Term(iTRAQ4plex			48		
209447070	SYT1	synaptotagmin-1	3.79	1	1	2	0.862	47.5								
									VPMNTVDFGHVTEEWF	2	N-Term(iTRAQ4plex	0.862	3.46	65	3	687.67
310114952	LOC100507703	PREDICTED: HLA class I histocompatibility antigen, A-69 alpha chain-like isoform 5	3.77	0	1	2		41.6								
									FIAVGYVDDTQFVF	2	N-Term(iTRAQ4plex		3.38	70	2	887.47
310114954	LOC100507703	PREDICTED: HLA class I histocompatibility antigen, A-69 alpha chain-like isoform 4	3.77	0	1	2		41.4								
									FIAVGYVDDTQFVF	2	N-Term(iTRAQ4plex		3.38	70	2	887.47
310124798	LOC100507703	PREDICTED: HLA class I histocompatibility antigen, A-69 alpha chain-like isoform 6	3.77	0	1	2		41.5								
									FIAVGYVDDTQFVF	2	N-Term(iTRAQ4plex		3.38	70	2	887.47

310124800	LOC100507703	PREDICTED: HLA class I histocompatibility antigen, A-69 alpha chain-like isoform 5	3.77	0	1	2		41.4								
									FIAVGYVDDTQFVF	2	N-Term(iTRAQ4plex		3.38	70	2	887.47
310124967	LOC100507680	PREDICTED: HLA class I histocompatibility antigen, A-69 alpha chain-like isoform 10	3.77	0	1	2		41.7								
									FIAVGYVDDTQFVF	2	N-Term(iTRAQ4plex		3.38	70	2	887.47
27894337	KRT20	keratin, type I cytoskeletal 20	3.77	0	2	3		48.5								
									LEQEIATYR	2	N-Term(iTRAQ4plex		4.13	44	2	633.84
									LASYLEK	1	N-Term(iTRAQ4plex); K7(iTRAQ4plex			46		
211938440	APOL1	apolipoprotein L1 isoform a precursor	3.77	0	1	2		43.9								
									VTEPISAESGEQVEF	2	N-Term(iTRAQ4plex		4.51	84	2	887.95
154146262	FCGBP	IgGFc-binding protein precursor	3.77	1	10	23	0.844	571.6								
									VTASSPVAVLSGHSCAQI	2	N-Term(iTRAQ4plex); C15(Methylthio); K18(iTRAQ4plex)		8.09	65	3	692.70
									AISGLTIDGHAVGAK	3	N-Term(iTRAQ4plex); K15(iTRAQ4plex		6.6	100	2	849.50
									VVAEVQICHGK	4	N-Term(iTRAQ4plex); C8(Methylthio); K11(iTRAQ4plex)		5.98	89	3	506.28
									GEVGFVLVDNQF	2	N-Term(iTRAQ4plex		4.95	78	2	738.90
									VNGVLTALPVSVADGF	3	N-Term(iTRAQ4plex	0.844	4.47	87	2	856.50
									VAYDLVYYVF	3	N-Term(iTRAQ4plex		4.18	38	2	702.89
									AIGYATAADCGF	2	N-Term(iTRAQ4plex); C10(Methylthio		3.84	74	2	679.82
									FYPAGDVLR	2	N-Term(iTRAQ4plex		3.18	38	2	591.32
									SVPGCEGVALVVAQTI	1	N-Term(iTRAQ4plex); C5(Methylthio); K16(iTRAQ4plex)			50		
									LPVVLANGQIF	1	N-Term(iTRAQ4plex			48		
4758248	EFNB1	ephrin-B1 precursor	3.76	1	1	2	0.968	38								
									NLEPVSWSSLNPK	2	N-Term(iTRAQ4plex); K13(iTRAQ4plex	0.968	4.81	97	2	879.99
78191798	KLKB1	plasma kallikrein precursor	3.76	2	2	3	1.11	71.3								
									EKGEIQNILQK	2	N-Term(iTRAQ4plex); K2(iTRAQ4plex); K11(iTRAQ4plex)	1.242	3.74	40	3	578.01
									VNIPLVTNEECQK	1	N-Term(iTRAQ4plex); C11(Methylthio); K13(iTRAQ4plex)	0.992		44		
310124969	LOC100507680	PREDICTED: HLA class I histocompatibility antigen, A-69 alpha chain-like isoform 9	3.75	0	1	2		41.7								
									FIAVGYVDDTQFVF	2	N-Term(iTRAQ4plex		3.38	70	2	887.47
66392203	NME1-NME2	NME1-NME2 protein	3.75	0	1	1		30.1								
									FMQASEDLLK	1	N-Term(iTRAQ4plex); K10(iTRAQ4plex			64		
310114940	LOC100507703	PREDICTED: HLA class I histocompatibility antigen, A-69 alpha chain-like isoform 10	3.74	0	1	2		42								
									FIAVGYVDDTQFVF	2	N-Term(iTRAQ4plex		3.38	70	2	887.47
94721250	VAPA	vesicle-associated membrane protein-associated protein A isoform 1	3.74	0	1	2		32.6								
									EAKPDELMDSK	2	N-Term(iTRAQ4plex); K3(iTRAQ4plex); K11(iTRAQ4plex)		3.7	46	3	565.63
310114942	LOC100507703	PREDICTED: HLA class I histocompatibility antigen, A-69 alpha chain-like isoform 8	3.72	0	1	2		42								
									FIAVGYVDDTQFVF	2	N-Term(iTRAQ4plex		3.38	70	2	887.47
262231791	CFHR3	complement factor H-related protein 3 isoform 2 precursor	3.72	0	1	13		30.7								
									RPYFPVAVGK	13	N-Term(iTRAQ4plex); K10(iTRAQ4plex		3.95	58	3	474.62
93587332	SCN3B	sodium channel subunit beta-3 precursor	3.72	1	1	2	0.784	24.7								
									DFLIYEYR	2	N-Term(iTRAQ4plex	0.784	3.41	41	2	631.83
228480211	FHL1	four and a half LIM domains protein 1 isoform 1	3.72	0	1	2		36.2								
									AIVAGDQNVEYK	2	N-Term(iTRAQ4plex); K12(iTRAQ4plex		4.31	51	2	797.94
90903247	CD200	OX-2 membrane glycoprotein isoform a precursor	3.72	0	1	2		30.3								
									EQLYTPASLK	2	N-Term(iTRAQ4plex); K10(iTRAQ4plex		3.98	65	2	719.41
4557701	KRT17	keratin, type I cytoskeletal 17	3.7	0	2	6		48.1								
									LEQEIATYR	2	N-Term(iTRAQ4plex		4.13	44	2	633.84
									LASYLDK	4	N-Term(iTRAQ4plex); K7(iTRAQ4plex		3.35	53	2	549.32
31543380	PARK7	protein DJ-1	3.7	1	1	2	1.122	19.9								
									ALVILAK	2	N-Term(iTRAQ4plex); K7(iTRAQ4plex	1.122	3.39	45	2	508.35
310114944	LOC100507703	PREDICTED: HLA class I histocompatibility antigen, A-69 alpha chain-like isoform 6	3.66	0	1	2		43.2								
									FIAVGYVDDTQFVF	2	N-Term(iTRAQ4plex		3.38	70	2	887.47
310124971	LOC100507680	PREDICTED: HLA class I histocompatibility antigen, A-69 alpha chain-like isoform 6	3.66	0	1	2		42.9								
									FIAVGYVDDTQFVF	2	N-Term(iTRAQ4plex		3.38	70	2	887.47
54607120	LTF	lactotransferrin isoform 1 precursor	3.66	0	2	4		78.1								
									LRPVAAEVYGTEF	2	N-Term(iTRAQ4plex		5.81	44	3	535.63
									CLAENAGDVAFVK	2	N-Term(iTRAQ4plex); C1(Methylthio); K13(iTRAQ4plex)		4.36	69	2	835.93
288915539	PLS3	plastin-3 isoform 2	3.65	0	2	4		67.6								
									MINLSVPDTIDER	2	N-Term(iTRAQ4plex		3.61	39	2	823.93
									LSPEELLLR	2	N-Term(iTRAQ4plex		3.44	42	2	607.37
40217823	SLITRK5	SLIT and NTRK-like protein 5 precursor	3.65	2	3	6	0.804	107.4								
									GIISLSEISPPR	2	N-Term(iTRAQ4plex	0.789	4.93	73	2	706.92
									LLPYVGLLQHMDK	1	N-Term(iTRAQ4plex); K13(iTRAQ4plex	0.819		58		
									FVPLTHLDLR	3	N-Term(iTRAQ4plex			53		
310114946	LOC100507703	PREDICTED: HLA class I histocompatibility antigen, A-69 alpha chain-like isoform 7	3.64	0	1	2		43.3								
									FIAVGYVDDTQFVF	2	N-Term(iTRAQ4plex		3.38	70	2	887.47
310124790	LOC100507703	PREDICTED: HLA class I histocompatibility antigen, A-69 alpha chain-like isoform 7	3.64	0	1	2		43.2								
									FIAVGYVDDTQFVF	2	N-Term(iTRAQ4plex		3.38	70	2	887.47
153082696	ICAM2	intercellular adhesion molecule 2 precursor	3.64	1	1	2	1.248	30.6								
									ILLDEQAQWK	2	N-Term(iTRAQ4plex); K10(iTRAQ4plex	1.248	3.55	73	2	766.44
72534716	MCUR1	coiled-coil domain-containing protein 90A, mitochondrial precursor	3.62	1	1	28	0.644	39.7								
									ELSLSAGSLQLER	28	N-Term(iTRAQ4plex	0.644	4.02	40	2	773.93
21735616	APOL1	apolipoprotein L1 isoform b precursor	3.62	0	1	2		45.9								
									VTEPISAESGEQVEF	2	N-Term(iTRAQ4plex		4.51	84	2	887.95
163792198	LPHN3	latrophilin-3 precursor	3.61	5	5	9	1.011	164.5								
									IEGTWDTAYDK	2	N-Term(iTRAQ4plex); K11(iTRAQ4plex	0.995	5.04	86	2	793.90
									IDYIYNTDQSK	2	N-Term(iTRAQ4plex); K11(iTRAQ4plex	1.028	4.57	71	2	824.43
									TDTLTEYSSK	2	N-Term(iTRAQ4plex); K10(iTRAQ4plex	0.94	3.39	69	2	716.88
									IVISQLNPYTLR	1	N-Term(iTRAQ4plex	0.95		62		

									VFLCPGLLK	2	N-Term(iTRAQ4plex); C4(Methylthio); K9(iTRAQ4plex)	1.074		49		
239757745	LOC100128355	PREDICTED: hypothetical protein LOC100128355	3.59	0	1	16		37.3								
									LIFAGKQLEDGR	16	N-Term(iTRAQ4plex); K6(iTRAQ4plex		5	73	2	817.98
239752254	LOC100128355	PREDICTED: hypothetical protein LOC100128355	3.59	0	1	16		37.3								
									LIFAGKQLEDGR	16	N-Term(iTRAQ4plex); K6(iTRAQ4plex		5	73	2	817.98
310119290	LOC256374	PREDICTED: peptidyl-prolyl cis-trans isomerase A-like	3.57	0	1	3		21.6								
									TEWLDGK	3	N-Term(iTRAQ4plex); K7(iTRAQ4plex		3.28	47	2	568.82
285002253	AGA	N(4)-(beta-N-acetylglucosaminyl)-L-asparaginase isoform 2 preproprotein	3.57	0	1	3		36								
									FLPSYQAVEYMR	3	N-Term(iTRAQ4plex		3.69	64	2	824.42
32698861	CBLN2	cerebellin-2 precursor	3.57	0	1	15		24.1								
									GNLMGGWK	15	N-Term(iTRAQ4plex); K8(iTRAQ4plex		3.64	48	2	575.81
54873613	AGRN	agrin precursor	3.57	6	6	26	0.987	214.7								
									TEATQGLVLWSGK	4	N-Term(iTRAQ4plex); K13(iTRAQ4plex	1.028	5.34	92	2	839.48
									TFVEYLNAVTESEK	2	N-Term(iTRAQ4plex); K14(iTRAQ4plex	1.149	5.14	72	2	959.51
									SAGDVDTLAFDGR	12	N-Term(iTRAQ4plex	0.971	4.9	98	2	734.36
									ALQSNHFELSLR	4	N-Term(iTRAQ4plex	1.024	4.46	55	2	779.93
									AYGTGFVGCLR	2	N-Term(iTRAQ4plex); C9(Methylthio	0.975	4.36	54	2	667.33
									QLLTPEHVLR	2	N-Term(iTRAQ4plex	0.98	3.72	52	3	450.61
296179438	SEZ6L	seizure 6-like protein isoform 4 precursor	3.56	0	3	5		110.3								
									SPTNTISVYFR	2	N-Term(iTRAQ4plex		4.83	67	2	714.88
									IMYCTDPGEVDHSTR	2	N-Term(iTRAQ4plex); C4(Methylthio		4.69	52	3	638.61
									ETGTPIWTSR	1	N-Term(iTRAQ4plex			37		
4826950	KLK7	kallikrein-7 preproprotein	3.56	1	1	2	1.689	27.5								
									WVLTAAHCK	2	N-Term(iTRAQ4plex); C8(Methylthio); K9(iTRAQ4plex)	1.689	3.45	40	3	454.91
296179440	SEZ6L	seizure 6-like protein isoform 3 precursor	3.55	0	3	5		110.4								
									SPTNTISVYFR	2	N-Term(iTRAQ4plex		4.83	67	2	714.88
									IMYCTDPGEVDHSTR	2	N-Term(iTRAQ4plex); C4(Methylthio		4.69	52	3	638.61
									ETGTPIWTSR	1	N-Term(iTRAQ4plex			37		
14277685	PCDHGC5	protocadherin gamma-C5 isoform 2 precursor	3.53	0	2	4		95.1								
									DGKPFPELVLEQQLDR	2	N-Term(iTRAQ4plex); K3(iTRAQ4plex		4.28	67	3	724.74
									HQLVLTAVDGGTPAR	2	N-Term(iTRAQ4plex		3.4	74	2	839.97
296179436	SEZ6L	seizure 6-like protein isoform 2 precursor	3.52	0	3	5		111.6								
									SPTNTISVYFR	2	N-Term(iTRAQ4plex		4.83	67	2	714.88
									IMYCTDPGEVDHSTR	2	N-Term(iTRAQ4plex); C4(Methylthio		4.69	52	3	638.61
									ETGTPIWTSR	1	N-Term(iTRAQ4plex			37		
282165714	TMEM132C	transmembrane protein 132C	3.52	3	3	3	0.879	121.7								
									SMDQPEGTPVELYYTVHPGNER	1	N-Term(iTRAQ4plex	0.848		45		
									FSFDWK	1	N-Term(iTRAQ4plex); K6(iTRAQ4plex	0.879		41		
32261332	SEZ6L	seizure 6-like protein isoform 1 precursor	3.52	0	3	5		111.7								
									SPTNTISVYFR	2	N-Term(iTRAQ4plex		4.83	67	2	714.88
									IMYCTDPGEVDHSTR	2	N-Term(iTRAQ4plex); C4(Methylthio		4.69	52	3	638.61
									ETGTPIWTSR	1	N-Term(iTRAQ4plex			37		
24234735	SEMA6D	semaphorin-6D isoform 2 precursor	3.51	0	3	5		111.7								
									LSTLEYDGEEISGLAR	2	N-Term(iTRAQ4plex		4.63	81	2	948.99
									QTNVALFADGK	2	N-Term(iTRAQ4plex); K11(iTRAQ4plex		4.48	78	2	726.41
									LDFQLMLK	1	N-Term(iTRAQ4plex); K8(iTRAQ4plex			36		
34147715	CHRDL1	chordin-like protein 1 isoform 3 precursor	3.51	0	1	4		52								
									GDGELSWEHSDGDIFR	4	N-Term(iTRAQ4plex		4.56	82	3	655.30
28372555	SCN4B	sodium channel subunit beta-4 isoform 1 precursor	3.51	1	1	2	1.071	25								
									ILIEGTVK	2	N-Term(iTRAQ4plex); K8(iTRAQ4plex	1.071	3.31	39	2	580.88
24430190	KRT15	keratin, type I cytoskeletal 15	3.51	0	2	6		49.2								
									LEQEIATYR	2	N-Term(iTRAQ4plex		4.13	44	2	633.84
									LASYLDK	4	N-Term(iTRAQ4plex); K7(iTRAQ4plex		3.35	53	2	549.32
5360208	IDS	iduronate 2-sulfatase isoform b precursor	3.5	0	1	4		38.3								
									QSTEQAIQLLEK	4	N-Term(iTRAQ4plex); K12(iTRAQ4plex		5.99	75	2	838.48
221316563	CHRDL1	chordin-like protein 1 isoform 2 precursor	3.5	0	1	4		52.1								
									GDGELSWEHSDGDIFR	4	N-Term(iTRAQ4plex		4.56	82	3	655.30
131412225	KRT13	keratin, type I cytoskeletal 13 isoform a	3.49	0	2	3		49.5								
									LEQEIATYR	2	N-Term(iTRAQ4plex		4.13	44	2	633.84
									LASYLEK	1	N-Term(iTRAQ4plex); K7(iTRAQ4plex			46		
7549809	PLS3	plastin-3 isoform 1	3.49	0	2	4		70.8								
									MINLSVPDTIDER	2	N-Term(iTRAQ4plex		3.61	39	2	823.93
									LSPEELLLR	2	N-Term(iTRAQ4plex		3.44	42	2	607.37
221316561	CHRDL1	chordin-like protein 1 isoform 1 precursor	3.49	0	1	4		52.2								
									GDGELSWEHSDGDIFR	4	N-Term(iTRAQ4plex		4.56	82	3	655.30
285002251	AGA	N(4)-(beta-N-acetylglucosaminyl)-L-asparaginase isoform 1 preproprotein	3.47	0	1	3		37.2								
									FLPSYQAVEYMR	3	N-Term(iTRAQ4plex		3.69	64	2	824.42
312222667	SEMA6D	semaphorin-6D isoform 1 precursor	3.46	0	3	5		113.2								
									LSTLEYDGEEISGLAR	2	N-Term(iTRAQ4plex		4.63	81	2	948.99
									QTNVALFADGK	2	N-Term(iTRAQ4plex); K11(iTRAQ4plex		4.48	78	2	726.41
									LDFQLMLK	1	N-Term(iTRAQ4plex); K8(iTRAQ4plex			36		
4503689	FGA	fibrinogen alpha chain isoform alpha-E preproprotein	3.46	0	3	4		94.9								
									VQHIQLLQK	2	N-Term(iTRAQ4plex); K9(iTRAQ4plex		3.71	50	3	465.63
									VPPEWK	1	N-Term(iTRAQ4plex); K6(iTRAQ4plex			38		
4557395	CA2	carbonic anhydrase 2	3.46	1	1	2	0.583	29.2								
									VVDVLDSIK	2	N-Term(iTRAQ4plex); K9(iTRAQ4plex	0.583	3.47	61	2	638.40
310110687	LOC100508605	PREDICTED: hypothetical protein LOC100508605	3.45	0	1	2		29.1								
									VTISVDTSK	2	N-Term(iTRAQ4plex); K9(iTRAQ4plex		3.72	54	2	619.37
24234738	SEMA6D	semaphorin-6D isoform 3 precursor	3.44	0	3	5		113.7								
									LSTLEYDGEEISGLAR	2	N-Term(iTRAQ4plex		4.63	81	2	948.99
									QTNVALFADGK	2	N-Term(iTRAQ4plex); K11(iTRAQ4plex		4.48	78	2	726.41
									LDFQLMLK	1	N-Term(iTRAQ4plex); K8(iTRAQ4plex			36		
51317373	LRRC4C	leucine-rich repeat-containing protein 4C precursor	3.44	0	3	11		71.9								
									NNPIESIPSYAFNR	6	N-Term(iTRAQ4plex		4.89	57	2	883.45
									RLDLGELK	4	N-Term(iTRAQ4plex); K8(iTRAQ4plex		4.16	47	3	411.26
									LDLGELK	1	N-Term(iTRAQ4plex); K7(iTRAQ4plex			43		

110611233	COL18A1	collagen alpha-1(XVIII) chain isoform 2 precursor	3.44	0	4	14		135.4								
									TEAPSATGQASSLLGGF	6	N-Term(iTRAQ4plex		6.47	118	2	873.96
									GADFQCFQQAR	2	N-Term(iTRAQ4plex); C6(Methylthio		4.31	56	2	730.83
									LQDLYSIVR	4	N-Term(iTRAQ4plex		4.03	42	2	625.87
									AVGLAGTFR	2	N-Term(iTRAQ4plex		3.48	45	2	518.31
215272347	ST6GAL2	beta-galactoside alpha-2,6-sialyltransferase 2 isoform b	3.43	0	1	2		52.9								
									AHPAGSFHAGPGDLQK	2	N-Term(iTRAQ4plex); K16(iTRAQ4plex		5.33	74	3	626.67
27262663	CSF1	macrophage colony-stimulating factor 1 isoform b precursor	3.42	0	1	2		47.8								
									STCQSFEPPETPVVK	2	N-Term(iTRAQ4plex); C3(Methylthio); K15(iTRAQ4plex)		3.64	45	2	991.99
7662150	FAM20B	glycosaminoglycan xylosylkinase precursor	3.42	1	1	5	0.82	46.4								
									DHVVEGEPYAGYDR	5	N-Term(iTRAQ4plex	0.82	4.18	55	3	584.27
310125105	LOC100507681	PREDICTED: HLA class I histocompatibility antigen, Cw-15 alpha chain-like isoform 12	3.41	0	1	1		32.4								
									DYIALNEDLR	2	N-Term(iTRAQ4plex		3.38	41	2	683.36
6912484	PLA2G15	group XV phospholipase A2 precursor	3.4	1	1	2	0.655	46.6								
									AFVSLGAPWGGVAK	2	N-Term(iTRAQ4plex); K14(iTRAQ4plex	0.655	3.19	77	2	824.48
11497043	ADAM22	disintegrin and metalloproteinase domain-containing protein 22 isoform 5 preproprotein	3.4	0	2	6		91.4								
									SDAVHLFSGSQFESSR	4	N-Term(iTRAQ4plex		5.11	50	3	633.31
									FAISENPLITLR	2	N-Term(iTRAQ4plex			49		
90903245	CD200	OX-2 membrane glycoprotein isoform b	3.4	0	1	2		33								
									EQLYTPASLK	2	N-Term(iTRAQ4plex); K10(iTRAQ4plex		3.98	65	2	719.41
21070967	NRXN1	neurexin-1-beta isoform beta precursor	3.39	0	2	6		46.8								
									AGGREPYPGSAEVIR	2	N-Term(iTRAQ4plex		3.67	36	2	851.96
									EPYPGSAEVIR	4	N-Term(iTRAQ4plex		3.52	36	2	681.36
295424145	PCSK1	neuroendocrine convertase 1 isoform 3	3.39	0	1	5		49.3								
									RDELEEGAPSQAMLR	4	N-Term(iTRAQ4plex		5.59	89	2	923.46
									RDELEEGAPSQAMLR	1	N-Term(iTRAQ4plex); M13(Oxidation			37		
214010238	MGRN1	E3 ubiquitin-protein ligase MGRN1 isoform 4	3.39	0	1	1		58.3								
									AASIENVLQDSSPEHCGR	1	N-Term(iTRAQ4plex); C16(Methylthio			35		
38455388	FRZB	secreted frizzled-related protein 3 precursor	3.38	1	1	2	0.984	36.2								
									LLLVEGSIAEK	2	N-Term(iTRAQ4plex); K11(iTRAQ4plex	0.984	4.25	47	2	730.46
222144244	FGFR2	fibroblast growth factor receptor 2 isoform 9 precursor	3.38	0	2	5		76.4								
									VYSDAQPHIQWIK	3	N-Term(iTRAQ4plex); K13(iTRAQ4plex		4.36	43	3	625.01
									YGPDGLPYLK	2	N-Term(iTRAQ4plex); K10(iTRAQ4plex		3.92	48	2	705.90
167466198	ICAM1	intercellular adhesion molecule 1 precursor	3.38	1	1	2	1.007	57.8								
									REPAVGEPAEVTTTVLVR	2	N-Term(iTRAQ4plex	1.007	4.51	76	3	690.06
149363636	PLXNB2	plexin-B2 precursor	3.37	5	5	10	0.962	205								
									EAFEAYTDHATYK	2	N-Term(iTRAQ4plex); K13(iTRAQ4plex	1.112	5.85	71	3	611.97
									LQLEQQVATGPALDNK	2	N-Term(iTRAQ4plex); K16(iTRAQ4plex	0.846	3.95	80	2	1007.06
									IQPETGPLGGGIR	4	N-Term(iTRAQ4plex	0.974	3.84	60	2	719.91
									LVECGSLFK	1	N-Term(iTRAQ4plex); C4(Methylthio); K9(iTRAQ4plex)	0.921		62		
									AEEASHWLWSR	1	N-Term(iTRAQ4plex	0.84		34		
310125081	LOC100507680	PREDICTED: HLA class I histocompatibility antigen, A-43 alpha chain-like isoform 17	3.37	0	1	1		33.5								
									DYIALNEDLR	2	N-Term(iTRAQ4plex		3.38	41	2	683.36
226437571	ABHD14A	abhydrolase domain-containing protein 14A	3.32	1	1	1	0.93	29.7								
									VEVVLLHGK	1	N-Term(iTRAQ4plex); K9(iTRAQ4plex	0.93		42		
4755125	AQP4	aquaporin-4 isoform b	3.32	0	1	1		32.3								
									DQSGEVLSSV	1	N-Term(iTRAQ4plex			44		
189217888	CHST8	carbohydrate sulfotransferase 8	3.3	1	1	4	0.905	48.8								
									NLPAPDQPQPPLQR	4	N-Term(iTRAQ4plex	0.905	4.34	74	2	857.98
258613943	ASPH	aspartyl/asparaginyl beta-hydroxylase isoform f	3.29	0	2	3		83.2								
									LGIYDADGDGDFDVDDAK	2	N-Term(iTRAQ4plex); K18(iTRAQ4plex		6.31	74	2	1095.01
									VLLGLK	1	N-Term(iTRAQ4plex); K6(iTRAQ4plex			32		
194018511	KRT77	keratin, type II cytoskeletal 1b	3.29	0	2	16		61.9								
									FLEQQNQVLQTK	12	N-Term(iTRAQ4plex); K12(iTRAQ4plex		6.7	96	2	882.50
									FASFIDK	4	N-Term(iTRAQ4plex); K7(iTRAQ4plex		3.19	53	2	558.31
19923989	CTHRC1	collagen triple helix repeat-containing protein 1 precursor	3.29	1	1	2	0.91	26.2								
									IIIEELPK	2	N-Term(iTRAQ4plex); K8(iTRAQ4plex	0.91		50		
154426280	VSIG4	V-set and immunoglobulin domain-containing protein 4 isoform 2 precursor	3.28	0	1	2		33.8								
									GSDPVTIFLR	2	N-Term(iTRAQ4plex		3.78	45	2	624.86
11128023	PCDHGC5	protocadherin gamma-C5 isoform 1 precursor	3.28	0	2	4		101.9								
									DGKPFPELVLEQQLDR	2	N-Term(iTRAQ4plex); K3(iTRAQ4plex		4.28	67	3	724.74
									HQLVLTAVDGGTPAR	2	N-Term(iTRAQ4plex		3.4	74	2	839.97
153791158	KRT75	keratin, type II cytoskeletal 75	3.27	0	2	5		59.5								
									FASFIDK	4	N-Term(iTRAQ4plex); K7(iTRAQ4plex		3.19	53	2	558.31
222144241	FGFR2	fibroblast growth factor receptor 2 isoform 8 precursor	3.27	0	2	5		79.1								
									VYSDAQPHIQWIK	3	N-Term(iTRAQ4plex); K13(iTRAQ4plex		4.36	43	3	625.01
									YGPDGLPYLK	2	N-Term(iTRAQ4plex); K10(iTRAQ4plex		3.92	48	2	705.90
16306532	CDH11	cadherin-11 preproprotein	3.27	2	2	2	1.023	87.9								
									VTITLTDVNDNPPK	1	N-Term(iTRAQ4plex); K14(iTRAQ4plex	1.022		62		
									FFTINPEDGFIK	1	N-Term(iTRAQ4plex); K12(iTRAQ4plex	1.024		37		
94538339	SIRPG	signal-regulatory protein gamma isoform 3 precursor	3.26	0	1	7		29.9								
									VTTVSDLTK	7	N-Term(iTRAQ4plex); K9(iTRAQ4plex		4.15	66	2	626.37
4757722	ADAM22	disintegrin and metalloproteinase domain-containing protein 22 isoform 4 preproprotein	3.26	0	2	6		95.2								
									SDAVHLFSGSQFESSR	4	N-Term(iTRAQ4plex		5.11	50	3	633.31
									FAISENPLITLR	2	N-Term(iTRAQ4plex			49		
24234741	SEMA6D	semaphorin-6D isoform 4 precursor	3.26	0	3	5		119.8								
									LSTLEYDGEEISGLAR	2	N-Term(iTRAQ4plex		4.63	81	2	948.99
									QTNVALFADGK	2	N-Term(iTRAQ4plex); K11(iTRAQ4plex		4.48	78	2	726.41
									LDFQLMLK	1	N-Term(iTRAQ4plex); K8(iTRAQ4plex			36		
222144237	FGFR2	fibroblast growth factor receptor 2 isoform 6 precursor	3.26	0	2	5		79.3								
									VYSDAQPHIQWIK	3	N-Term(iTRAQ4plex); K13(iTRAQ4plex		4.36	43	3	625.01
									YGPDGLPYLK	2	N-Term(iTRAQ4plex); K10(iTRAQ4plex		3.92	48	2	705.90
214010236	MGRN1	E3 ubiquitin-protein ligase MGRN1 isoform 3	3.26	0	1	1		60.7								

									AASIENVLQDSSPEHCGF	1	N-Term(iTRAQ4plex); C16(Methylthio			35		
222144235	FGFR2	fibroblast growth factor receptor 2 isoform 5 precursor	3.25	0	2	5		79.2								
									VYSDAQPHIQWIK	3	N-Term(iTRAQ4plex); K13(iTRAQ4plex		4.36	43	3	625.01
									YGPDGLPYLK	2	N-Term(iTRAQ4plex); K10(iTRAQ4plex		3.92	48	2	705.90
115387106	NBL1	neuroblastoma suppressor of tumorigenicity 1 isoform 1	3.24	0	1	27		23.2								
									LALFPDK	27	N-Term(iTRAQ4plex); K7(iTRAQ4plex			44		
214010234	MGRN1	E3 ubiquitin-protein ligase MGRN1 isoform 2	3.24	0	1	1		60.8								
									AASIENVLQDSSPEHCGF	1	N-Term(iTRAQ4plex); C16(Methylthio			35		
30840978	NLGN2	neuroligin-2 precursor	3.23	1	2	4	0.986	90.8								
									FQPPEAPASWPGVR	2	N-Term(iTRAQ4plex	0.986	4.34	38	2	841.94
									GNYGLLDQIQALR	2	N-Term(iTRAQ4plex		3.18	52	2	802.95
21536383	ADAM22	disintegrin and metalloproteinase domain-containing protein 22 isoform 3 preproprotein	3.22	0	2	6		96.6								
									SDAVHLFSGSQFESSR	4	N-Term(iTRAQ4plex		5.11	50	3	633.31
									FAISENPLITLR	2	N-Term(iTRAQ4plex			49		
29725609	EGFR	epidermal growth factor receptor isoform a precursor	3.22	0	3	5		134.2								
									NLQEILHGAVR	2	N-Term(iTRAQ4plex		4.03	47	3	465.27
									EISDGDVIISGNK	2	N-Term(iTRAQ4plex); K13(iTRAQ4plex		3.52	67	2	817.95
									ACGADSYEMEEDGVR	1	N-Term(iTRAQ4plex); C2(Methylthio			49		
4505571	SQSTM1	sequestosome-1 isoform 1	3.18	0	1	3		47.7								
									LTPVSPESSSTEEK	3	N-Term(iTRAQ4plex); K14(iTRAQ4plex		4.69	97	2	889.97
14589866	ASPH	aspartyl/asparaginyl beta-hydroxylase isoform a	3.17	0	2	3		85.8								
									LGIYDADGDGDFDVDDAK	2	N-Term(iTRAQ4plex); K18(iTRAQ4plex		6.31	74	2	1095.01
									VLLGLK	1	N-Term(iTRAQ4plex); K6(iTRAQ4plex			32		
40217843	COMP	cartilage oligomeric matrix protein precursor	3.17	2	2	3	0.854	82.8								
									QMEQTYWQANPFR	2	N-Term(iTRAQ4plex	0.901	3.48	53	2	921.94
									DTDLDGFPDEK	1	N-Term(iTRAQ4plex); K11(iTRAQ4plex	0.809		63		
53988380	MSLN	mesothelin isoform 2 preproprotein	3.17	0	2	4		68.9								
									LLGPHVEGLK	2	N-Term(iTRAQ4plex); K10(iTRAQ4plex		3.61	43	3	450.95
									EIDESLIFYK	2	N-Term(iTRAQ4plex); K10(iTRAQ4plex		3.26	67	2	772.93
4502049	AKR1B1	aldose reductase	3.16	1	1	1	0.715	35.8								
									MPILGLGTWK	1	N-Term(iTRAQ4plex); K10(iTRAQ4plex	0.715		62		
296010988	MINPP1	multiple inositol polyphosphate phosphatase 1 isoform 3	3.15	0	1	2		33.1								
									VLEYLNDLK	2	N-Term(iTRAQ4plex); K9(iTRAQ4plex		3.3	64	2	697.91
310125101	LOC100507681	PREDICTED: HLA class I histocompatibility antigen, Cw-15 alpha chain-like isoform 11	3.14	0	1	1		35.1								
									DYIALNEDLR	2	N-Term(iTRAQ4plex		3.38	41	2	683.36
19743803	FBLN5	fibulin-5 precursor	3.13	1	1	2	0.732	50.1								
									SVPADIFQMQATTF	2	N-Term(iTRAQ4plex	0.732	4.49	77	2	854.95
44917608	MGRN1	E3 ubiquitin-protein ligase MGRN1 isoform 1	3.13	0	1	1		63.2								
									AASIENVLQDSSPEHCGF	1	N-Term(iTRAQ4plex); C16(Methylthio			35		
296434251	VSIG4	V-set and immunoglobulin domain-containing protein 4 isoform 4 precursor	3.12	0	1	2		35.5								
									GSDPVTIFLR	2	N-Term(iTRAQ4plex		3.78	45	2	624.86
21536386	ADAM22	disintegrin and metalloproteinase domain-containing protein 22 isoform 2 preproprotein	3.11	0	2	6		100.2								
									SDAVHLFSGSQFESSR	4	N-Term(iTRAQ4plex		5.11	50	3	633.31
									FAISENPLITLR	2	N-Term(iTRAQ4plex			49		
4758304	PDIA4	protein disulfide-isomerase A4 precursor	3.1	2	2	3	0.983	72.9								
									IDATSASVLASR	2	N-Term(iTRAQ4plex	0.926	3.7	48	2	667.87
									SPPIPLAK	1	N-Term(iTRAQ4plex); K8(iTRAQ4plex	1.043		40		
4502181	AQP4	aquaporin-4 isoform a	3.1	0	1	1		34.8								
									DQSGEVLSSV	1	N-Term(iTRAQ4plex			44		
19882209	IL1RAP	interleukin-1 receptor accessory protein isoform 2	3.09	0	1	2		41								
									QIQVFEDEPAR	2	N-Term(iTRAQ4plex		3.91	65	2	738.38
11497047	ADAM22	disintegrin and metalloproteinase domain-containing protein 22 isoform 1 preproprotein	3.09	0	2	6		100.4								
									SDAVHLFSGSQFESSR	4	N-Term(iTRAQ4plex		5.11	50	3	633.31
									FAISENPLITLR	2	N-Term(iTRAQ4plex			49		
19923748	DLST	dihydrolipoyllysine-residue succinyltransferase component of 2-oxoglutarate dehydrogenase complex, mitochondria	3.09	1	1	2	0.863	48.7								
									TPAFAESVTEGDVF	2	N-Term(iTRAQ4plex	0.863	5.22	65	2	811.91
310125115	LOC100507681	PREDICTED: HLA class I histocompatibility antigen, Cw-15 alpha chain-like isoform 15, partial	3.09	0	1	1		35.8								
									DYIALNEDLR	2	N-Term(iTRAQ4plex)		3.38	41	2	683.36
134133226	POTEE	POTE ankyrin domain family member E	3.07	0	3	7		121.3								
									SYELPDGQVITIGNER	3	N-Term(iTRAQ4plex		3.27	49	2	968.00
									IWHHTFYNELR	3	N-Term(iTRAQ4plex			41		
									GILTLK	1	N-Term(iTRAQ4plex); K6(iTRAQ4plex			31		
153791352	POTEF	POTE ankyrin domain family member F	3.07	0	3	7		121.4								
									SYELPDGQVITIGNER	3	N-Term(iTRAQ4plex		3.27	49	2	968.00
									IWHHTFYNELR	3	N-Term(iTRAQ4plex			41		
									GILTLK	1	N-Term(iTRAQ4plex); K6(iTRAQ4plex			31		
157738649	NEFM	neurofilament medium polypeptide isoform 1	3.06	0	1	1		102.4								
									EGSSEKEEGEQEEGETEAEAEGEEAEAK	1	N-Term(iTRAQ4plex); K6(iTRAQ4plex); K28(iTRAQ4plex)			39		
167001141	GPC1	glypican-1 precursor	3.05	1	1	6	0.804	61.6								
									TPLTHALPGLSEQEGQK	6	N-Term(iTRAQ4plex); K17(iTRAQ4plex	0.804	4.96	62	3	698.72
22748757	KRT222	keratin-like protein KRT222	3.05	0	1	2		34.1								
									LEQEIATYR	2	N-Term(iTRAQ4plex		4.13	44	2	633.84
66932947	A2M	alpha-2-macroglobulin precursor	3.05	3	4	8	0.62	163.2								
									LVHVEEPHTETVF	4	N-Term(iTRAQ4plex	0.396	3.96	55	3	563.97
									SASNMAIVDVK	2	N-Term(iTRAQ4plex); K11(iTRAQ4plex	0.804		59		
									ATVLNYLPK	1	N-Term(iTRAQ4plex); K9(iTRAQ4plex			54		
									NEDSLVFVQTDK	1	N-Term(iTRAQ4plex); K12(iTRAQ4plex	0.62		50		
115529484	CD109	CD109 antigen isoform 1 precursor	3.04	0	4	6		161.6								
									ISVFIQTDK	2	N-Term(iTRAQ4plex); K9(iTRAQ4plex		3.82	39	2	669.90
									TNIQVTVTGPSSPSPVK	2	N-Term(iTRAQ4plex); K17(iTRAQ4plex		3.76	54	2	1000.57
									NNVVITVTQF	1	N-Term(iTRAQ4plex			54		
									IPVQLVFK	1	N-Term(iTRAQ4plex); K8(iTRAQ4plex			47		
109255249	KRT4	keratin, type II cytoskeletal 4	3.03	0	2	8		63.9								

									LALDIEIATYR	5	N-Term(iTRAQ4plex		3.43	51	2	711.41
									FASFIDK	4	N-Term(iTRAQ4plex); K7(iTRAQ4plex		3.19	53	2	558.31
110611235	COL18A1	collagen alpha-1(XVIII) chain isoform 1 precursor	3.03	0	4	14		153.7								
									TEAPSATGQASSLLGGF	6	N-Term(iTRAQ4plex		6.47	118	2	873.96
									GADFQCFQQAR	2	N-Term(iTRAQ4plex); C6(Methylthio		4.31	56	2	730.83
									LQDLYSIVR	4	N-Term(iTRAQ4plex		4.03	42	2	625.87
									AVGLAGTFR	2	N-Term(iTRAQ4plex		3.48	45	2	518.31
54792787	CFHR3	complement factor H-related protein 3 isoform 1 precursor	3.03	0	1	13		37.3								
									RPYFPVAVGK	13	N-Term(iTRAQ4plex); K10(iTRAQ4plex		3.95	58	3	474.62
21361621	PGM1	phosphoglucomutase-1 isoform 1	3.02	0	2	3		61.4								
									VDLGVLGK	2	N-Term(iTRAQ4plex); K8(iTRAQ4plex		3.6	43	2	544.85
									SIFDFSALK	1	N-Term(iTRAQ4plex); K9(iTRAQ4plex			42		
26190610	ST6GAL2	beta-galactoside alpha-2,6-sialyltransferase 2 isoform a	3.02	0	1	2		60.1								
									AHPAGSFHAGPGDLQK	2	N-Term(iTRAQ4plex); K16(iTRAQ4plex		5.33	74	3	626.67
9966903	MAN1C1	mannosyl-oligosaccharide 1,2-alpha-mannosidase IC	3.02	1	1	2	0.986	70.9								
									TQQPGLEVVAEIAGHAPAR	2	N-Term(iTRAQ4plex	0.986	4.71	84	3	696.71
19923129	IFNAR2	interferon alpha/beta receptor 2 isoform b precursor	3.02	0	1	1		37.4								
									SFCDLTDEWR	1	N-Term(iTRAQ4plex); C3(Methylthio			34		
48762934	COL1A2	collagen alpha-2(I) chain precursor	3	3	3	8	0.957	129.2								
									GETGPSGPVGPAGAVGPR	4	N-Term(iTRAQ4plex	0.89	5.38	115	2	853.95
									DYEVDATLK	2	N-Term(iTRAQ4plex); K9(iTRAQ4plex	1.017	4.62	47	2	671.36
									NSIAYMDEETGNLK	2	N-Term(iTRAQ4plex); K14(iTRAQ4plex	1.029	3.56	83	2	936.97
270265859	KIAA0319	KIAA0319 isoform d precursor	2.97	0	2	3		110.3								
									GSPSGIWGDSPEDIR	2	N-Term(iTRAQ4plex		4.17	64	2	858.92
5454038	LEPREL4	synaptonemal complex protein SC65	2.97	1	1	1	0.747	50.3								
									GDEAEAEPEPELA	1	N-Term(iTRAQ4plex	0.747		32		
4504415	HLA-G	HLA class I histocompatibility antigen, alpha chain G precursor	2.96	0	1	1		38.2								
									DYIALNEDLR	2	N-Term(iTRAQ4plex		3.38	41	2	683.36
116534898	DSG2	desmoglein-2 preproprotein	2.95	3	3	5	0.968	122.2								
									IVAISEDYPR	2	N-Term(iTRAQ4plex	0.825	4.17	51	2	653.86
									DTGEIYTTSVTLDR	2	N-Term(iTRAQ4plex	0.968	3.3	42	2	857.93
4557617	GAS6	growth arrest-specific protein 6 isoform 1 precursor	2.95	0	2	3		74.9								
									IAVAGDLFQPER	2	N-Term(iTRAQ4plex		4.45	37	2	730.40
									LVAEFDFR	1	N-Term(iTRAQ4plex			43		
5453549	PRDX4	peroxiredoxin-4	2.95	0	1	2		30.5								
									GLFIIDDK	2	N-Term(iTRAQ4plex); K8(iTRAQ4plex			55		
7669503	LAMP2	lysosome-associated membrane glycoprotein 2 isoform B precursor	2.93	0	1	2		44.9								
									GILTVDELLAIR	2	N-Term(iTRAQ4plex		3.32	60	2	728.95
290463102	PGM1	phosphoglucomutase-1 isoform 2	2.93	0	2	3		63.8								
									VDLGVLGK	2	N-Term(iTRAQ4plex); K8(iTRAQ4plex		3.6	43	2	544.85
									SIFDFSALK	1	N-Term(iTRAQ4plex); K9(iTRAQ4plex			42		
4504957	LAMP2	lysosome-associated membrane glycoprotein 2 isoform A precursor	2.93	0	1	2		44.9								
									GILTVDELLAIR	2	N-Term(iTRAQ4plex		3.32	60	2	728.95
169790833	LAMP2	lysosome-associated membrane glycoprotein 2 isoform C precursor	2.92	0	1	2		45.1								
									GILTVDELLAIR	2	N-Term(iTRAQ4plex		3.32	60	2	728.95
21703710	SHISA5	protein shisa-5 precursor	2.92	1	1	5	0.923	25.6								
									FVWSEER	5	N-Term(iTRAQ4plex	0.923	3.13	43	2	548.78
61743973	SORCS1	VPS10 domain-containing receptor SorCS1 isoform a	2.91	0	3	6		129.6								
									VSENPYTSGIIASK	4	N-Term(iTRAQ4plex); K14(iTRAQ4plex		3.75	104	2	877.48
									EPDLVHLEAR	1	N-Term(iTRAQ4plex			50		
									HLWLSFDEGR	1	N-Term(iTRAQ4plex			36		
4507521	TKT	transketolase	2.89	1	1	1	0.573	67.8								
									LGQSDPAPLQHQMDIYQK	1	N-Term(iTRAQ4plex); K18(iTRAQ4plex	0.573		35		
40217817	SLITRK1	SLIT and NTRK-like protein 1 precursor	2.87	2	2	16	0.672	77.7								
									DIDPGAFQDLNK	4	N-Term(iTRAQ4plex); K12(iTRAQ4plex	0.743	4.46	89	2	810.92
									EWLENIPK	12	N-Term(iTRAQ4plex); K8(iTRAQ4plex	0.672	3.89	54	2	658.88
49472828	YIPF3	protein YIPF3	2.86	1	1	27	0.917	38.2								
									DIPAMLPAAR	20	N-Term(iTRAQ4plex	0.915	3.87	50	2	599.84
									DIPAMLPAAR	7	N-Term(iTRAQ4plex); M5(Oxidation	0.945		48		
61743975	SORCS1	VPS10 domain-containing receptor SorCS1 isoform b	2.84	0	3	6		133.3								
									VSENPYTSGIIASK	4	N-Term(iTRAQ4plex); K14(iTRAQ4plex		3.75	104	2	877.48
									EPDLVHLEAR	1	N-Term(iTRAQ4plex			50		
									HLWLSFDEGR	1	N-Term(iTRAQ4plex			36		
42475534	CLSTN3	calsyntenin-3 precursor	2.82	2	2	12	0.783	106								
									ESLLLDTTSLQQR	10	N-Term(iTRAQ4plex	0.815	5.03	71	2	824.46
									VNDVNEFAPVFVER	2	N-Term(iTRAQ4plex	0.709	4.64	66	2	889.96
270265853	KIAA0319	KIAA0319 isoform b	2.82	0	2	3		117								
									GSPSGIWGDSPEDIR	2	N-Term(iTRAQ4plex		4.17	64	2	858.92
148664205	ESAM	endothelial cell-selective adhesion molecule precursor	2.82	1	1	1	0.828	41.2								
									SKPAVQYQWDR	1	N-Term(iTRAQ4plex); K2(iTRAQ4plex	0.828		84		
14589931	PCDH7	protocadherin-7 isoform a precursor	2.81	0	2	3		116								
									YELLQEPGGGGSGGESR	2	N-Term(iTRAQ4plex		3.6	58	2	918.95
									LDETSGWLSVLHR	1	N-Term(iTRAQ4plex			50		
226530908	PCMT1	protein-L-isoaspartate(D-aspartate) O-methyltransferase	2.81	1	1	3	1.192	30.3								
									VIGIDHIK	3	N-Term(iTRAQ4plex); K8(iTRAQ4plex	1.192		41		
14589933	PCDH7	protocadherin-7 isoform b precursor	2.8	0	2	3		116.4								
									YELLQEPGGGGSGGESR	2	N-Term(iTRAQ4plex		3.6	58	2	918.95
									LDETSGWLSVLHR	1	N-Term(iTRAQ4plex			50		
270265855	KIAA0319	KIAA0319 isoform a precursor	2.8	0	2	3		117.7								
									GSPSGIWGDSPEDIR	2	N-Term(iTRAQ4plex		4.17	64	2	858.92
40549451	LYVE1	lymphatic vessel endothelial hyaluronic acid receptor 1	2.8	1	1	4	0.754	35.2								
									IMGITLVSK	4	N-Term(iTRAQ4plex); K9(iTRAQ4plex	0.754	3.72	61	2	625.40
7662470	NLGN1	neurologin-1	2.79	1	2	3	0.772	91.9								

									ELVDQDIQPAR	2	N-Term(iTRAQ4plex	0.772	3.87	62	2	714.39
190358524	FSTL5	follistatin-related protein 5 isoform c	2.75	0	2	3		94.5								
									YEDTGAYTCIAK	2	N-Term(iTRAQ4plex); C9(Methylthio); K12(iTRAQ4plex)		3.9	88	2	834.90
23111005	MFAP4	microfibril-associated glycoprotein 4 isoform 2 precursor	2.75	0	1	1		28.6								
									GFYYSLK	1	N-Term(iTRAQ4plex); K7(iTRAQ4plex			36		
116174742	FAM20C	dentin matrix protein 4	2.74	1	1	2	1.102	66.2								
									SESPPGPGGDASLLAR	2	N-Term(iTRAQ4plex	1.102	4.33	78	2	827.93
89063265	LOC652614	PREDICTED: BOLA class I histocompatibility antigen, alpha chain BL3-7-like	2.73	0	1	1		41.2								
									DYIALNEDLR	2	N-Term(iTRAQ4plex		3.38	41	2	683.36
62821776	KIAA1467	hypothetical protein LOC57613	2.73	1	1	1	0.883	67								
									TTGPSSEGHPAALVVSK	1	N-Term(iTRAQ4plex); K17(iTRAQ4plex	0.883		40		
260436864	AP1B1	AP-1 complex subunit beta-1 isoform c	2.72	0	2	4		101.3								
									MEPLNNLQVAVK	2	N-Term(iTRAQ4plex); K12(iTRAQ4plex		5.46	78	2	822.48
									NVEGQDMLYQSLK	2	N-Term(iTRAQ4plex); K13(iTRAQ4plex		5.37	85	2	906.98
190358522	FSTL5	follistatin-related protein 5 isoform b	2.72	0	2	3		95.6								
									YEDTGAYTCIAK	2	N-Term(iTRAQ4plex); C9(Methylthio); K12(iTRAQ4plex)		3.9	88	2	834.90
190358520	FSTL5	follistatin-related protein 5 isoform a	2.72	0	2	3		95.7								
									YEDTGAYTCIAK	2	N-Term(iTRAQ4plex); C9(Methylthio); K12(iTRAQ4plex)		3.9	88	2	834.90
145580631	QSOX2	sulfhydryl oxidase 2 precursor	2.72	2	2	4	0.966	77.5								
									AFFSSYLK	2	N-Term(iTRAQ4plex); K8(iTRAQ4plex	1.023	3.41	38	2	625.86
									ISGIFLTNHIK	2	N-Term(iTRAQ4plex); K11(iTRAQ4plex	0.912	3.35	43	3	510.98
84452146	B3GALNT1	UDP-GalNAc:beta-1,3-N-acetylgalactosaminyltransferase 1	2.72	1	1	1	0.901	39.5								
									IHLDVCQLR	1	N-Term(iTRAQ4plex); C6(Methylthio	0.901		47		
14165431	PCDHAC2	protocadherin alpha-C2 isoform 2 precursor	2.71	0	2	3		96.1								
									VLDTNDNSPAFDQSTYR	2	N-Term(iTRAQ4plex		5.08	100	2	1043.99
									VLELVLR	1	N-Term(iTRAQ4plex			32		
50659093	MAN2B2	epididymis-specific alpha-mannosidase precursor	2.68	2	2	3	1.247	113.9								
									IEQEYQAGPLELNR	2	N-Term(iTRAQ4plex	1.094	3.65	89	2	902.47
									SLTGTWDLSMLHR	1	N-Term(iTRAQ4plex	1.421		37		
24638433	NCSTN	nicastrin precursor	2.68	2	2	3	1.002	78.4								
									ALADVATVLGR	2	N-Term(iTRAQ4plex	0.929	3.51	67	2	615.37
									LLYGFLIK	1	N-Term(iTRAQ4plex); K8(iTRAQ4plex	1.08		28		
145207994	HES7	transcription factor HES-7 isoform 2	2.67	0	1	1		24.9								
									LLLLER	1	N-Term(iTRAQ4plex			31		
260436860	AP1B1	AP-1 complex subunit beta-1 isoform b	2.66	0	2	4		103.5								
									MEPLNNLQVAVK	2	N-Term(iTRAQ4plex); K12(iTRAQ4plex		5.46	78	2	822.48
									NVEGQDMLYQSLK	2	N-Term(iTRAQ4plex); K13(iTRAQ4plex		5.37	85	2	906.98
4557791	NEU1	sialidase-1 precursor	2.65	1	1	1	1.012	45.4								
									LIVCGHGTLER	1	N-Term(iTRAQ4plex); C4(Methylthio	1.012		49		
206597445	COL18A1	collagen alpha-1(XVIII) chain isoform 3 precursor	2.63	0	4	14		177.8								
									TEAPSATGQASSLLGGR	6	N-Term(iTRAQ4plex		6.47	118	2	873.96
									GADFQCFQQAR	2	N-Term(iTRAQ4plex); C6(Methylthio		4.31	56	2	730.83
									LQDLYSIVR	4	N-Term(iTRAQ4plex		4.03	42	2	625.87
									AVGLAGTFR	2	N-Term(iTRAQ4plex		3.48	45	2	518.31
260436862	AP1B1	AP-1 complex subunit beta-1 isoform a	2.63	0	2	4		104.5								
									MEPLNNLQVAVK	2	N-Term(iTRAQ4plex); K12(iTRAQ4plex		5.46	78	2	822.48
									NVEGQDMLYQSLK	2	N-Term(iTRAQ4plex); K13(iTRAQ4plex		5.37	85	2	906.98
157427675	ADAMTS4	A disintegrin and metalloproteinase with thrombospondin motifs 4 preproprotein	2.63	2	2	3	0.945	90.1								
									SPASGQGPMCNVK	2	N-Term(iTRAQ4plex); C10(Methylthio); K13(iTRAQ4plex)	0.937	5.37	96	2	805.39
13236528	FKRP	fukutin-related protein	2.63	1	1	1	0.741	54.5								
									LVAAPVATANPAR	1	N-Term(iTRAQ4plex	0.741		41		
110815855	ST8SIA3	sia-alpha-2,3-Gal-beta-1,4-GlcNAc-R:alpha 2,8-sialyltransferase	2.63	1	1	1	0.864	43.9								
									TLVDFFVEHR	1	N-Term(iTRAQ4plex	0.864		38		
270288820	OSMR	oncostatin-M-specific receptor subunit beta isoform 2 precursor	2.63	0	1	1		39.5								
									LPLTPVSLK	1	N-Term(iTRAQ4plex); K9(iTRAQ4plex			53		
125628632	KRT80	keratin, type II cytoskeletal 80 isoform K80.1	2.61	0	1	4		47.2								
									LALDIEIATYR	5	N-Term(iTRAQ4plex		3.43	51	2	711.41
260166650	HES7	transcription factor HES-7 isoform 1	2.61	0	1	1		25.3								
									LLLLER	1	N-Term(iTRAQ4plex			31		
7662036	SPOCK2	testican-2 isoform 2	2.59	1	1	2	1.199	46.7								
									EKPPCLAELER	2	N-Term(iTRAQ4plex); K2(iTRAQ4plex); C5(Methylthio)	1.199	3.94	62	3	540.29
27502405	CD46	membrane cofactor protein isoform 14 precursor	2.58	0	1	1		39.3								
									ATVMFECDK	1	N-Term(iTRAQ4plex); C7(Methylthio); K9(iTRAQ4plex)			41		
227430303	CD109	CD109 antigen isoform 3 precursor	2.56	0	3	4		152.6								
									TNIQVTVTGPSSPSPVK	2	N-Term(iTRAQ4plex); K17(iTRAQ4plex		3.76	54	2	1000.57
									NNVVITVTQR	1	N-Term(iTRAQ4plex			54		
									IPVQLVFK	1	N-Term(iTRAQ4plex); K8(iTRAQ4plex			47		
4503979	GFAP	glial fibrillary acidic protein isoform 1	2.55	0	1	4		49.8								
									LALDIEIATYR	5	N-Term(iTRAQ4plex		3.43	51	2	711.41
196115290	GFAP	glial fibrillary acidic protein isoform 2	2.55	0	1	4		49.5								
									LALDIEIATYR	5	N-Term(iTRAQ4plex		3.43	51	2	711.41
27502427	CD46	membrane cofactor protein isoform 12 precursor	2.54	0	1	1		39.8								
									ATVMFECDK	1	N-Term(iTRAQ4plex); C7(Methylthio); K9(iTRAQ4plex)			41		
29788996	PON3	serum paraoxonase/lactonase 3	2.54	0	1	2		39.6								
									LLIGTVFHK	3	N-Term(iTRAQ4plex); K9(iTRAQ4plex		4.07	52	3	439.28
148762980	SIGLEC14	sialic acid-binding Ig-like lectin 14 precursor	2.53	0	1	2		43.9								
									MEDTGSYFFR	2	N-Term(iTRAQ4plex		3.43	45	2	698.82
6005958	VSIG4	V-set and immunoglobulin domain-containing protein 4 isoform 1 precursor	2.51	0	1	2		44								
									GSDPVTIFLR	2	N-Term(iTRAQ4plex		3.78	45	2	624.86
14589893	CDH4	cadherin-4 preproprotein	2.51	2	2	6	0.919	100.2								

									GTQYETNSMDFK	2	N-Term(iTRAQ4plex); K12(iTRAQ4plex	1.015	6.16	67	2	854.91
									VGADGTVFATR	4	N-Term(iTRAQ4plex	0.897	3.46	56	2	619.34
310923208	MFAP4	microfibril-associated glycoprotein 4 isoform 1 precursor	2.51	0	1	1		31.1								
									GFYYSLK	1	N-Term(iTRAQ4plex); K7(iTRAQ4plex			36		
157384973	TNR	tenascin-R precursor	2.5	2	2	3	0.945	149.5								
									LNPATEYEISLNSVR	2	N-Term(iTRAQ4plex	1.034	4.27	62	2	925.49
									VGFGNVEDEFWLGLDNIHR	1	N-Term(iTRAQ4plex	0.863		45		
4506141	HTRA1	serine protease HTRA1 precursor	2.5	1	1	2	0.733	51.3								
									IAPAVVHIELFR	2	N-Term(iTRAQ4plex	0.733	3.9	49	3	503.64
169646441	GDI2	rab GDP dissociation inhibitor beta isoform 2	2.5	0	1	1		45.6								
									VICILSHPIK	1	N-Term(iTRAQ4plex); C3(Methylthio); K10(iTRAQ4plex)			43		
4506121	PROZ	vitamin K-dependent protein Z precursor	2.5	1	1	1	2.578	44.7								
									DFAEHLLIPR	1	N-Term(iTRAQ4plex	2.578		40		
167466164	GSTT1	glutathione S-transferase theta-1	2.5	1	1	1	0.719	27.3								
									IVDLIK	1	N-Term(iTRAQ4plex); K6(iTRAQ4plex	0.719		44		
4503899	GALNS	N-acetylgalactosamine-6-sulfatase precursor	2.49	1	1	2	0.759	58								
									GDTLMAATLGQHK	2	N-Term(iTRAQ4plex); K13(iTRAQ4plex	0.759	5.65	64	3	544.30
27502409	CD46	membrane cofactor protein isoform 5 precursor	2.49	0	1	1		40.8								
									ATVMFECDK	1	N-Term(iTRAQ4plex); C7(Methylthio); K9(iTRAQ4plex)			41		
22749183	LINGO2	leucine-rich repeat and immunoglobulin-like domain-containing nogo receptor-interacting protein 2 precursor	2.48	0	1	3		68								
									SLEVGDNDLVYISHR	3	N-Term(iTRAQ4plex		5.04	37	3	620.99
33413400	ESD	S-formylglutathione hydrolase	2.48	1	1	1	0.346	31.4								
									KIPVVFR	1	N-Term(iTRAQ4plex); K1(iTRAQ4plex	0.346		32		
290463104	PGM1	phosphoglucomutase-1 isoform 3	2.47	0	1	1		40.3								
									SIFDFSALK	1	N-Term(iTRAQ4plex); K9(iTRAQ4plex			42		
315139010	POC1B-GALNT4	POC1B-GALNT4 protein isoform 2	2.46	0	1	2		45.7								
									AAEVWMDEYK	2	N-Term(iTRAQ4plex); K10(iTRAQ4plex		3.52	68	2	765.37
27502411	CD46	membrane cofactor protein isoform 6 precursor	2.44	0	1	1		41.3								
									ATVMFECDK	1	N-Term(iTRAQ4plex); C7(Methylthio); K9(iTRAQ4plex)			41		
218749883	MAN1B1	endoplasmic reticulum mannosyl-oligosaccharide 1,2-alpha-mannosidase	2.43	1	1	2	0.907	79.5								
									IPYSDVNIGTGVAHPPR	2	N-Term(iTRAQ4plex	0.907	6.11	53	3	646.35
125628636	KRT80	keratin, type II cytoskeletal 80 isoform K80	2.43	0	1	4		50.5								
									LALDIEIATYR	5	N-Term(iTRAQ4plex		3.43	51	2	711.41
110431348	DCC	netrin receptor DCC precursor	2.42	3	3	6	0.815	158.4								
									FLSEPSDAVTMR	2	N-Term(iTRAQ4plex	0.846	3.87	48	2	748.88
									NQQDLTPIPGDSR	2	N-Term(iTRAQ4plex	0.678	3.55	59	2	792.91
									DVVPVLVSSR	2	N-Term(iTRAQ4plex	0.815	3.14	36	2	607.87
167614504	LAMB1	laminin subunit beta-1 precursor	2.41	3	3	7	0.895	197.9								
									VESLSQVEVILQHSAADIAR	2	N-Term(iTRAQ4plex	1.203	3.83	56	3	770.43
									LHTLGDNLLDSR	3	N-Term(iTRAQ4plex	0.837	3.63	63	2	749.41
									NIGNLFEEAEK	2	N-Term(iTRAQ4plex); K11(iTRAQ4plex	0.943	3.51	69	2	776.41
139394621	CSPG5	chondroitin sulfate proteoglycan 5 precursor	2.41	1	1	4	1.028	56.9								
									EAGSAVEAEELVK	4	N-Term(iTRAQ4plex); K13(iTRAQ4plex	1.028	6.33	108	2	810.44
291084609	PCDH7	protocadherin-7 isoform c precursor	2.41	0	2	3		135.6								
									YELLQEPGGGGSGGESR	2	N-Term(iTRAQ4plex		3.6	58	2	918.95
									LDETSGWLSVLHR	1	N-Term(iTRAQ4plex			50		
4758282	EPHA7	ephrin type-A receptor 7 precursor	2.4	2	2	6	0.945	112								
									GTCVSSAEEEAENAPR	4	N-Term(iTRAQ4plex); C3(Methylthio	0.937	7.19	89	2	920.40
									EVLLLDSK	2	N-Term(iTRAQ4plex); K8(iTRAQ4plex	0.973	3.66	42	2	602.88
13899303	CRISPLD1	cysteine-rich secretory protein LCCL domain-containing 1	2.4	1	1	1	2.26	56.9								
									YMDEDGEWWIAK	1	N-Term(iTRAQ4plex); K12(iTRAQ4plex	2.26		51		
291084612	PCDH7	protocadherin-7 isoform d precursor	2.39	0	2	3		136.5								
									YELLQEPGGGGSGGESR	2	N-Term(iTRAQ4plex		3.6	58	2	918.95
									LDETSGWLSVLHR	1	N-Term(iTRAQ4plex			50		
41152086	SERPINB6	serpin B6	2.39	1	1	1	0.913	42.6								
									GNWDEQFDK	1	N-Term(iTRAQ4plex); K9(iTRAQ4plex	0.913		35		
27502407	CD46	membrane cofactor protein isoform 3 precursor	2.39	0	1	1		42.2								
									ATVMFECDK	1	N-Term(iTRAQ4plex); C7(Methylthio); K9(iTRAQ4plex)			41		
110347437	LTBP4	latent-transforming growth factor beta-binding protein 4 isoform c	2.38	0	3	4		165.6								
									VSGPWEEADAEAVAR	2	N-Term(iTRAQ4plex		5.1	80	2	865.93
									VSAPDGPCPTGFER	1	N-Term(iTRAQ4plex); C8(Methylthio			49		
									VPEGFTCR	1	N-Term(iTRAQ4plex); C7(Methylthio			32		
9256600	PCDHAC2	protocadherin alpha-C2 isoform 1 precursor	2.38	0	2	3		109.4								
									VLDTNDNSPAFDQSTYR	2	N-Term(iTRAQ4plex		5.08	100	2	1043.99
									VLELVLR	1	N-Term(iTRAQ4plex			32		
4826876	OMD	osteomodulin precursor	2.38	1	1	2	1.023	49.5								
									LLLGYNEISK	2	N-Term(iTRAQ4plex); K10(iTRAQ4plex	1.023	4.06	77	2	719.44
17136143	OLFM1	noelin isoform 1	2.36	1	1	2	0.909	54.1								
									LTGISDPVTVK	2	N-Term(iTRAQ4plex); K11(iTRAQ4plex	0.909	4.74	64	2	709.43
217330598	GLOD4	glyoxalase domain-containing protein 4	2.35	1	1	2	0.968	33.2								
									SDEWFAK	2	N-Term(iTRAQ4plex); K7(iTRAQ4plex	0.968		53		
14589914	PCDH10	protocadherin-10 isoform 2 precursor	2.34	0	2	6		96.9								
									SFDYEQLK	5	N-Term(iTRAQ4plex); K8(iTRAQ4plex		3.4	49	2	659.35
									EAGDSYTLTVVAR	1	N-Term(iTRAQ4plex			66		
24432108	CD46	membrane cofactor protein isoform 4 precursor	2.34	0	1	1		42.7								
									ATVMFECDK	1	N-Term(iTRAQ4plex); C7(Methylthio); K9(iTRAQ4plex)			41		
94538335	SIRPG	signal-regulatory protein gamma isoform 1 precursor	2.33	0	1	7		42.5								
									VTTVSDLTK	7	N-Term(iTRAQ4plex); K9(iTRAQ4plex		4.15	66	2	626.37
13124891	GALNT1	polypeptide N-acetylgalactosaminyltransferase 1	2.33	1	1	2	0.996	64.2								
									NVETNQCLDNMAR	2	N-Term(iTRAQ4plex); C7(Methylthio	0.996	3.74	56	2	849.38
110347412	LTBP4	latent-transforming growth factor beta-binding protein 4 isoform b	2.33	0	3	4		169.3								
									VSGPWEEADAEAVAR	2	N-Term(iTRAQ4plex		5.1	80	2	865.93
									VSAPDGPCPTGFER	1	N-Term(iTRAQ4plex); C8(Methylthio			49		
									VPEGFTCR	1	N-Term(iTRAQ4plex); C7(Methylthio			32		

21264363	MASP2	mannan-binding lectin serine protease 2 isoform 1 precursor	2.33	0	1	2		75.7								
									LASPGFPGEYANDQER	2	N-Term(iTRAQ4plex		3.61	63	2	947.95
21361873	RELT	tumor necrosis factor receptor superfamily member 19L precursor	2.33	1	1	1	1.027	46.1								
									LEAQVGMATR	1	N-Term(iTRAQ4plex	1.027		46		
93141003	SPOCK3	testican-3 isoform 1	2.31	0	1	4		49								
									LEYQACVLGK	4	N-Term(iTRAQ4plex); C6(Methylthio); K10(iTRAQ4plex)		4.69	70	2	729.39
5453832	HYOU1	hypoxia up-regulated protein 1 precursor	2.3	1	2	3	0.831	111.3								
									AEAGPEGVAPAPEGEK	2	N-Term(iTRAQ4plex); K16(iTRAQ4plex	0.831	4.95	84	2	898.97
78190498	SMOC1	SPARC-related modular calcium-binding protein 1 isoform 1	2.3	0	1	3		48.2								
									SYESMCEYQR	3	N-Term(iTRAQ4plex); C6(Methylthio		3.33	42	2	743.30
11545873	SMOC1	SPARC-related modular calcium-binding protein 1 isoform 2	2.3	0	1	3		48.1								
									SYESMCEYQR	3	N-Term(iTRAQ4plex); C6(Methylthio		3.33	42	2	743.30
166362713	CDH5	cadherin-5 preproprotein	2.3	2	2	4	0.965	87.5								
									YTFVVPEDTR	2	N-Term(iTRAQ4plex	0.999	3.79	43	2	685.85
									YEIVVEAR	2	N-Term(iTRAQ4plex	0.932	3.4	38	2	561.82
27502402	CD46	membrane cofactor protein isoform 1 precursor	2.3	0	1	1		43.7								
									ATVMFECDK	1	N-Term(iTRAQ4plex); C7(Methylthio); K9(iTRAQ4plex)			41		
93141001	SPOCK3	testican-3 isoform 2	2.29	0	1	4		49.4								
									LEYQACVLGK	4	N-Term(iTRAQ4plex); C6(Methylthio); K10(iTRAQ4plex)		4.69	70	2	729.39
110347431	LTBP4	latent-transforming growth factor beta-binding protein 4 isoform a	2.28	0	3	4		173.3								
									VSGPWEEADAEAVAR	2	N-Term(iTRAQ4plex		5.1	80	2	865.93
									VSAPDGPCPTGFER	1	N-Term(iTRAQ4plex); C8(Methylthio			49		
									VPEGFTCR	1	N-Term(iTRAQ4plex); C7(Methylthio			32		
295849272	DPP10	inactive dipeptidyl peptidase 10 isoform a	2.28	0	2	3		85.4								
									EYYITMVK	2	N-Term(iTRAQ4plex); K8(iTRAQ4plex		3.21	37	2	667.87
21450863	ATRN	attractin isoform 2	2.28	0	2	4		141.3								
									LTGSSGFVTDGPGNYK	2	N-Term(iTRAQ4plex); K16(iTRAQ4plex		5.72	92	2	944.49
									NHNALLASLTTQK	2	N-Term(iTRAQ4plex); K13(iTRAQ4plex		4.74	62	2	849.99
283436133	IGSF1	immunoglobulin superfamily member 1 isoform 4	2.26	0	2	3		147.9								
									EGEQEPVQQLGAVGR	2	N-Term(iTRAQ4plex		3.25	58	2	870.96
									FALLQEGAHVPLQFR	1	N-Term(iTRAQ4plex			36		
14150145	MXRA8	matrix-remodeling-associated protein 8 precursor	2.26	1	1	2	0.952	49.1								
									LLDLYASGER	2	N-Term(iTRAQ4plex	0.952	3.64	40	2	640.85
4826702	DSC1	desmocollin-1 isoform Dsc1b preproprotein	2.26	0	1	1		93.8								
									ILEDGSIYTTHDLILSSER	1	N-Term(iTRAQ4plex			53		
27502423	CD46	membrane cofactor protein isoform 2 precursor	2.26	0	1	1		44.2								
									ATVMFECDK	1	N-Term(iTRAQ4plex); C7(Methylthio); K9(iTRAQ4plex)			41		
45505167	IGSF1	immunoglobulin superfamily member 1 isoform	2.25	0	2	3		148.8								
									EGEQEPVQQLGAVGR	2	N-Term(iTRAQ4plex		3.25	58	2	870.96
									FALLQEGAHVPLQFR	1	N-Term(iTRAQ4plex			36		
4502271	ATP1A2	sodium/potassium-transporting ATPase subunit alpha-2 proprotein	2.25	1	2	2	1.3	112.2								
									VDNSSLTGESEPQTR	1	N-Term(iTRAQ4plex			52		
									LSLDELGR	1	N-Term(iTRAQ4plex	1.3		41		
283436131	IGSF1	immunoglobulin superfamily member 1 isoform 3	2.24	0	2	3		149.3								
									EGEQEPVQQLGAVGR	2	N-Term(iTRAQ4plex		3.25	58	2	870.96
									FALLQEGAHVPLQFR	1	N-Term(iTRAQ4plex			36		
83921602	SLC38A10	putative sodium-coupled neutral amino acid transporter 10 isoform a	2.23	2	2	3	0.908	119.7								
									GPEQVPVPDPAR	2	N-Term(iTRAQ4plex	0.918	4.18	64	2	703.38
									DLGPHAEGQLAPR	1	N-Term(iTRAQ4plex	0.898		36		
15451916	BMPR2	bone morphogenetic protein receptor type-2 precursor	2.22	2	2	3	0.756	115.1								
									DPYQQDLGIGESR	2	N-Term(iTRAQ4plex	0.762	3.17	53	2	811.40
									GSTCYGLWEK	1	N-Term(iTRAQ4plex); C4(Methylthio); K10(iTRAQ4plex)	0.751		43		
205277383	MST1	hepatocyte growth factor-like protein precursor	2.21	1	1	2	1.056	81.9								
									VVGGHPGNSPWTVSLR	2	N-Term(iTRAQ4plex	1.056	5.55	49	3	603.00
157426829	LINGO3	leucine-rich repeat and immunoglobulin-like domain-containing nogo receptor-interacting protein 3 precursor	2.2	1	1	2	0.981	64.8								
									LTAVPDGIPAETR	2	N-Term(iTRAQ4plex	0.981	3.89	53	2	742.42
240255535	COL6A3	collagen alpha-3(VI) chain isoform 4 precursor	2.18	0	4	7		278								
									VEFSLTDYGSK	2	N-Term(iTRAQ4plex); K11(iTRAQ4plex		4.69	68	2	767.40
									EVQVFEITENSAK	2	N-Term(iTRAQ4plex); K13(iTRAQ4plex		4.52	93	2	891.48
									IAVAQYSDDVK	2	N-Term(iTRAQ4plex); K11(iTRAQ4plex		3.88	79	2	748.92
									VAVVQHAPSESVDNASMPPVK	1	N-Term(iTRAQ4plex); K21(iTRAQ4plex			54		
86792661	DPP3	dipeptidyl peptidase 3	2.17	1	1	4	0.885	82.5								
									VILGSEAAQQHPEEVR	4	N-Term(iTRAQ4plex	0.885	5.36	78	3	636.34
85787627	DPP10	inactive dipeptidyl peptidase 10 isoform short	2.15	0	2	3		90.1								
									EYYITMVK	2	N-Term(iTRAQ4plex); K8(iTRAQ4plex		3.21	37	2	667.87
295842403	DPP10	inactive dipeptidyl peptidase 10 isoform b	2.15	0	2	3		90.3								
									EYYITMVK	2	N-Term(iTRAQ4plex); K8(iTRAQ4plex		3.21	37	2	667.87
52426756	DPP10	inactive dipeptidyl peptidase 10 isoform long	2.14	0	2	3		90.8								
									EYYITMVK	2	N-Term(iTRAQ4plex); K8(iTRAQ4plex		3.21	37	2	667.87
295842359	DPP10	inactive dipeptidyl peptidase 10 isoform c	2.13	0	2	3		91.1								
									EYYITMVK	2	N-Term(iTRAQ4plex); K8(iTRAQ4plex		3.21	37	2	667.87
4506325	PTPRR	receptor-type tyrosine-protein phosphatase R isoform 1 precursor	2.13	1	1	2	0.895	73.8								
									TGISDALPSEEVLR	2	N-Term(iTRAQ4plex	0.895	5.43	73	2	815.94
194097442	PTPRN2	receptor-type tyrosine-protein phosphatase N2 isoform 3 precursor	2.13	0	1	2		108								
									SEHPESSLSSEEETAGVENVK	2	N-Term(iTRAQ4plex); K21(iTRAQ4plex		5.2	76	2	1267.11
13435361	DSC1	desmocollin-1 isoform Dsc1a preproprotein	2.13	0	1	1		99.9								
									ILEDGSIYTTHDLILSSER	1	N-Term(iTRAQ4plex			53		

295424143	PCSK1	neuroendocrine convertase 1 isoform 2	2.12	0	1	5		79								
									RDELEEGAPSQAMLR	4	N-Term(iTRAQ4plex		5.59	89	2	923.46
									RDELEEGAPSQAMLR	1	N-Term(iTRAQ4plex); M13(Oxidation			37		
194097438	PTPRN2	receptor-type tyrosine-protein phosphatase N2 isoform 2 precursor	2.1	0	1	2		109.6								
									SEHPESSLSSEEETAGVENVK	2	N-Term(iTRAQ4plex); K21(iTRAQ4plex		5.2	76	2	1267.11
262206315	SELL	L-selectin precursor	2.08	1	1	2	1.305	43.6								
									AEIEYLEK	2	N-Term(iTRAQ4plex); K8(iTRAQ4plex	1.305	3.94	44	2	641.86
50845384	ADAMTS1	A disintegrin and metalloproteinase with thrombospondin motifs 1 preproprotein	2.07	1	1	2	0.951	105.3								
									QGDVGGTCGVVDDEPRPTGK	2	N-Term(iTRAQ4plex); C8(Methylthio); K20(iTRAQ4plex)	0.951	3.7	53	3	774.38
194097440	PTPRN2	receptor-type tyrosine-protein phosphatase N2 isoform 1 precursor	2.07	0	1	2		111.2								
									SEHPESSLSSEEETAGVENVK	2	N-Term(iTRAQ4plex); K21(iTRAQ4plex		5.2	76	2	1267.11
88703043	SNX6	sorting nexin-6 isoform a	2.07	0	1	1		33.5								
									LSDLLK	1	N-Term(iTRAQ4plex); K6(iTRAQ4plex			33		
157266300	ANPEP	aminopeptidase N precursor	2.07	2	2	2	0.851	109.5								
									VVATTQMQAADAR	1	N-Term(iTRAQ4plex	0.878		64		
									ELWILNR	1	N-Term(iTRAQ4plex	0.825		37		
255918077	VCAN	versican core protein isoform 3 precursor	2.03	0	4	15		264.9								
									VSVPTHPEAVGDASLTVVK	9	N-Term(iTRAQ4plex); K19(iTRAQ4plex		7.91	94	3	732.08
									LATVGELQAAWR	2	N-Term(iTRAQ4plex		5.48	55	2	729.91
									LLASDAGLYR	2	N-Term(iTRAQ4plex		4.01	45	2	611.85
									FTFEEAAK	2	N-Term(iTRAQ4plex); K8(iTRAQ4plex		3.58	59	2	615.83
32967597	PKLR	pyruvate kinase isozymes R/L isoform 2	2.03	0	1	9		58.5								
									GDLGIEIPAEK	9	N-Term(iTRAQ4plex); K11(iTRAQ4plex		4.18	56	2	715.41
21450861	ATRN	attractin isoform 1	2.03	0	2	4		158.4								
									LTGSSGFVTDGPGNYK	2	N-Term(iTRAQ4plex); K16(iTRAQ4plex		5.72	92	2	944.49
									NHNALLASLTTQK	2	N-Term(iTRAQ4plex); K13(iTRAQ4plex		4.74	62	2	849.99
10716563	CANX	calnexin precursor	2.03	1	1	2	0.885	67.5								
									TPELNLDQFHDK	2	N-Term(iTRAQ4plex); K12(iTRAQ4plex	0.885	5.3	56	3	582.31
14589916	PCDH10	protocadherin-10 isoform 1 precursor	2.02	0	2	6		112.9								
									SFDYEQLK	5	N-Term(iTRAQ4plex); K8(iTRAQ4plex		3.4	49	2	659.35
									EAGDSYTLTVVAR	1	N-Term(iTRAQ4plex			66		
4557251	ADAM10	disintegrin and metalloproteinase domain-containing protein 10 precursor	2.01	1	1	2	0.967	84.1								
									YGPQGGCADHSVFER	2	N-Term(iTRAQ4plex); C7(Methylthio	0.967	3.65	58	2	906.90
13929462	B4GALT1	beta-1,4-galactosyltransferase 1	2.01	1	1	1	0.746	43.9								
									VAIIIPFR	1	N-Term(iTRAQ4plex	0.746		33		
31559829	KRT25	keratin, type I cytoskeletal 25	2	0	1	9		49.3								
									DAEAWFNEK	9	N-Term(iTRAQ4plex); K9(iTRAQ4plex		4.11	65	2	699.34
26051218	CAMK2B	calcium/calmodulin-dependent protein kinase type II subunit beta isoform 8	2	0	1	1		50.9								
									FYFENLLAK	1	N-Term(iTRAQ4plex); K9(iTRAQ4plex			45		
20336242	PCSK1	neuroendocrine convertase 1 isoform 1 preproprotein	1.99	0	1	5		84.1								
									RDELEEGAPSQAMLR	4	N-Term(iTRAQ4plex		5.59	89	2	923.46
									RDELEEGAPSQAMLR	1	N-Term(iTRAQ4plex); M13(Oxidation			37		
145309313	GALNT13	polypeptide N-acetylgalactosaminyltransferase 13	1.98	1	1	4	0.969	64								
									GNQLWEYDAER	4	N-Term(iTRAQ4plex	0.969	4.31	77	2	762.86
153945736	KRT27	keratin, type I cytoskeletal 27	1.96	0	1	9		49.8								
									DAEAWFNEK	9	N-Term(iTRAQ4plex); K9(iTRAQ4plex		4.11	65	2	699.34
114431246	KRT28	keratin, type I cytoskeletal 28	1.94	0	1	9		50.5								
									DAEAWFNEK	9	N-Term(iTRAQ4plex); K9(iTRAQ4plex		4.11	65	2	699.34
21361116	VCAN	versican core protein isoform 1 precursor	1.94	0	6	20		372.6								
									VSVPTHPEAVGDASLTVVK	9	N-Term(iTRAQ4plex); K19(iTRAQ4plex		7.91	94	3	732.08
									LATVGELQAAWR	2	N-Term(iTRAQ4plex		5.48	55	2	729.91
									LLASDAGLYR	2	N-Term(iTRAQ4plex		4.01	45	2	611.85
									FTFEEAAK	2	N-Term(iTRAQ4plex); K8(iTRAQ4plex		3.58	59	2	615.83
									LDISEIK	4	N-Term(iTRAQ4plex); K7(iTRAQ4plex		3.23	56	2	553.33
									TEEEVFSGMK	1	N-Term(iTRAQ4plex); K10(iTRAQ4plex			64		
46488937	IFNAR2	interferon alpha/beta receptor 2 isoform a precursor	1.94	0	1	1		57.7								
									SFCDLTDEWR	1	N-Term(iTRAQ4plex); C3(Methylthio			34		
115527070	COL6A2	collagen alpha-2(VI) chain isoform 2C2a' precursor	1.93	0	1	2		87.2								
									DVTVTAIGIGDMFHEK	2	N-Term(iTRAQ4plex); K16(iTRAQ4plex		5.42	80	3	674.36
302058265	UNC5C	netrin receptor UNC5C precursor	1.93	2	2	3	0.848	103.1								
									VDETSGLIVR	2	N-Term(iTRAQ4plex	0.864	3.49	51	2	616.85
									EVSIEISR	1	N-Term(iTRAQ4plex	0.832		39		
4504661	IL1RAP	interleukin-1 receptor accessory protein isoform 1	1.93	0	1	2		65.4								
									QIQVFEDEPAR	2	N-Term(iTRAQ4plex		3.91	65	2	738.38
116174734	PAPPA2	pappalysin-2 isoform 2	1.93	0	1	1		92.1								
									YQPGTWTHVAATYDGR	1	N-Term(iTRAQ4plex			34		
40354205	ALDOB	fructose-bisphosphate aldolase B	1.92	0	1	2		39.4								
									VLAAVYK	2	N-Term(iTRAQ4plex); K7(iTRAQ4plex		3.15	41	2	526.34
10835121	PKLR	pyruvate kinase isozymes R/L isoform 1	1.92	0	1	9		61.8								
									GDLGIEIPAEK	9	N-Term(iTRAQ4plex); K11(iTRAQ4plex		4.18	56	2	715.41
5729999	RRAGA	ras-related GTP-binding protein A	1.92	0	1	7		36.5								
									ISNIIK	7	N-Term(iTRAQ4plex); K6(iTRAQ4plex			35		
315113881	PTPRN	receptor-type tyrosine-protein phosphatase-like N isoform 3 precursor	1.91	0	1	2		96.2								
									GEKPASPAVQPDAALQR	2	N-Term(iTRAQ4plex); K3(iTRAQ4plex		3.4	41	3	675.05
53850576	OR1K1	olfactory receptor 1K1	1.9	1	1	1	0.73	34.2								
									ALLIGR	1	N-Term(iTRAQ4plex	0.73		32		
4504169	GSS	glutathione synthetase	1.9	1	1	1	0.79	52.4								
									AIENELLAR	1	N-Term(iTRAQ4plex	0.79		48		
47419930	CSPG4	chondroitin sulfate proteoglycan 4 precursor	1.89	3	3	5	0.963	250.4								
									GSLLLGGLDAEASR	2	N-Term(iTRAQ4plex	0.844	5.16	100	2	751.92
									LSDGQGFTQDDIQAGR	2	N-Term(iTRAQ4plex	0.997	3.68	93	2	926.45
									HGELELDIPGAQAR	1	N-Term(iTRAQ4plex	0.963		58		
227430301	CD109	CD109 antigen isoform 2 precursor	1.89	0	3	4		159.6								
									ISVFIQTDK	2	N-Term(iTRAQ4plex); K9(iTRAQ4plex		3.82	39	2	669.90
									NNVVITVTQR	1	N-Term(iTRAQ4plex			54		
									IPVQLVFK	1	N-Term(iTRAQ4plex); K8(iTRAQ4plex			47		

295844837	ACE	angiotensin-converting enzyme isoform 3 precursor	1.88	0	1	2		78.6								
									AALPAQELEEYNK	2	N-Term(iTRAQ4plex); K13(iTRAQ4plex		3.31	82	2	882.47
55743106	COL6A3	collagen alpha-3(VI) chain isoform 5 precursor	1.88	0	4	7		321.2								
									VEFSLTDYGSK	2	N-Term(iTRAQ4plex); K11(iTRAQ4plex		4.69	68	2	767.40
									EVQVFEITENSAK	2	N-Term(iTRAQ4plex); K13(iTRAQ4plex		4.52	93	2	891.48
									IAVAQYSDDVK	2	N-Term(iTRAQ4plex); K11(iTRAQ4plex		3.88	79	2	748.92
									VAVVQHAPSESVDNASMPPVI	1	N-Term(iTRAQ4plex); K21(iTRAQ4plex			54		
26051216	CAMK2B	calcium/calmodulin-dependent protein kinase type II subunit beta isoform 7	1.88	0	1	1		54								
									FYFENLLAK	1	N-Term(iTRAQ4plex); K9(iTRAQ4plex			45		
150456460	EPHA10	ephrin type-A receptor 10 isofom 3	1.88	0	1	1		109.6								
									KIDTIAADESFTQGDLGEF	2	N-Term(iTRAQ4plex); K1(iTRAQ4plex		4.58	94	3	785.41
13112048	FGFR3	fibroblast growth factor receptor 3 isoform 2 precursor	1.87	0	1	1		75.6								
									VGPDGTPYVTVLK	1	N-Term(iTRAQ4plex); K13(iTRAQ4plex			63		
19923761	MINPP1	multiple inositol polyphosphate phosphatase 1 isoform 1 precursor	1.85	0	1	2		55								
									VLEYLNDLK	2	N-Term(iTRAQ4plex); K9(iTRAQ4plex		3.3	64	2	697.91
29568105	TMEFF1	tomoregulin-1 precursor	1.84	0	1	1		40.9								
									EITVIAR	1	N-Term(iTRAQ4plex			34		
94538350	PCDH17	protocadherin-17 precursor	1.81	2	2	2	1.005	126.2								
									FPELVIQK	1	N-Term(iTRAQ4plex); K8(iTRAQ4plex	0.947		41		
4757960	CDH1	cadherin-1 preproprotein	1.81	1	1	2	1.182	97.4								
									GQVPENEANVVITTLH	2	N-Term(iTRAQ4plex); K16(iTRAQ4plex	1.182	3.86	41	2	1000.57
4502659	SIGLEC5	sialic acid-binding Ig-like lectin 5 precursor	1.81	0	1	2		60.7								
									MEDTGSYFFR	2	N-Term(iTRAQ4plex		3.43	45	2	698.82
109148539	GGT7	gamma-glutamyltransferase 7	1.81	1	1	1	0.823	70.4								
									EQALHWVAETLK	1	N-Term(iTRAQ4plex); K12(iTRAQ4plex	0.823		36		
315113878	PTPRN	receptor-type tyrosine-protein phosphatase-like N isoform 2 precursor	1.79	0	1	2		102.8								
									GEKPASPAVQPDAALQF	2	N-Term(iTRAQ4plex); K3(iTRAQ4plex		3.4	41	3	675.05
26051212	CAMK2B	calcium/calmodulin-dependent protein kinase type II subunit beta isoform 5	1.79	0	1	1		56.3								
									FYFENLLAK	1	N-Term(iTRAQ4plex); K9(iTRAQ4plex			45		
23238214	ACE	angiotensin-converting enzyme isoform 2 precursor	1.78	0	1	2		83.3								
									AALPAQELEEYNK	2	N-Term(iTRAQ4plex); K13(iTRAQ4plex		3.31	82	2	882.47
16306530	CDH10	cadherin-10 preproprotein	1.78	1	1	2	0.733	88.4								
									ISIEDVDEPPVFSR	2	N-Term(iTRAQ4plex	0.733	3.32	61	2	873.96
55743098	COL6A3	collagen alpha-3(VI) chain isoform 1 precursor	1.76	0	4	7		343.5								
									VEFSLTDYGSK	2	N-Term(iTRAQ4plex); K11(iTRAQ4plex		4.69	68	2	767.40
									EVQVFEITENSAK	2	N-Term(iTRAQ4plex); K13(iTRAQ4plex		4.52	93	2	891.48
									IAVAQYSDDVK	2	N-Term(iTRAQ4plex); K11(iTRAQ4plex		3.88	79	2	748.92
									VAVVQHAPSESVDNASMPPVI	1	N-Term(iTRAQ4plex); K21(iTRAQ4plex			54		
51477714	MAN2A1	alpha-mannosidase 2	1.75	2	2	3	0.854	131.1								
									FDQTGLMK	2	N-Term(iTRAQ4plex); K8(iTRAQ4plex	0.921	3.28	45	2	614.34
									YLVVYNPLEQDR	1	N-Term(iTRAQ4plex	0.792		47		
4504329	SPINT1	kunitz-type protease inhibitor 1 isoform 2 precursor	1.75	0	1	2		56.8								
									EGFINYLTR	2	N-Term(iTRAQ4plex		3.33	35	2	628.84
4757734	AIM2	interferon-inducible protein AIM2	1.75	1	1	1	1.014	38.9								
									IIIIAR	1	N-Term(iTRAQ4plex	1.014		24		
315221121	POC1B-GALNT4	POC1B-GALNT4 protein isoform 1	1.74	0	1	2		66.2								
									AAEVWMDEYK	2	N-Term(iTRAQ4plex); K10(iTRAQ4plex		3.52	68	2	765.37
115527066	COL6A2	collagen alpha-2(VI) chain isoform 2C2a precursor	1.74	0	1	2		97.4								
									DVTVTAIGIGDMFHEK	2	N-Term(iTRAQ4plex); K16(iTRAQ4plex		5.42	80	3	674.36
4506321	PTPRN	receptor-type tyrosine-protein phosphatase-like N isoform 1 precursor	1.74	0	1	2		105.8								
									GEKPASPAVQPDAALQF	2	N-Term(iTRAQ4plex); K3(iTRAQ4plex		3.4	41	3	675.05
26051210	CAMK2B	calcium/calmodulin-dependent protein kinase type II subunit beta isoform 4	1.74	0	1	1		57.9								
									FYFENLLAK	1	N-Term(iTRAQ4plex); K9(iTRAQ4plex			45		
26051208	CAMK2B	calcium/calmodulin-dependent protein kinase type II subunit beta isoform 3	1.74	0	1	1		58								
									FYFENLLAK	1	N-Term(iTRAQ4plex); K9(iTRAQ4plex			45		
34452725	GALNT4	polypeptide N-acetylgalactosaminyltransferase 4	1.73	0	1	2		66.6								
									AAEVWMDEYK	2	N-Term(iTRAQ4plex); K10(iTRAQ4plex		3.52	68	2	765.37
46249395	RRAGB	ras-related GTP-binding protein B short isoform	1.73	0	1	7		40.1								
									ISNIIK	7	N-Term(iTRAQ4plex); K6(iTRAQ4plex			35		
10337581	KRT33B	keratin, type I cuticular Ha3-II	1.73	0	1	1		46.2								
									LASYLEK	1	N-Term(iTRAQ4plex); K7(iTRAQ4plex			46		
14917117	KRT33A	keratin, type I cuticular Ha3-I	1.73	0	1	1		45.9								
									LASYLEK	1	N-Term(iTRAQ4plex); K7(iTRAQ4plex			46		
4502951	COL3A1	collagen alpha-1(III) chain preproprotein	1.71	2	2	4	0.926	138.5								
									NSIAYMDQASGNVK	2	N-Term(iTRAQ4plex); K14(iTRAQ4plex	1.027	5.82	96	2	893.46
									INTDEIMTSLK	2	N-Term(iTRAQ4plex); K11(iTRAQ4plex	0.836	4.38	72	2	776.93
209954788	ATF6B	cyclic AMP-dependent transcription factor ATF-6 beta isoform b	1.71	0	1	1		76.4								
									AVPIQPPGPPER	1	N-Term(iTRAQ4plex			37		
20631977	ATF6B	cyclic AMP-dependent transcription factor ATF-6 beta isoform a	1.71	0	1	1		76.7								
									AVPIQPPGPPER	1	N-Term(iTRAQ4plex			37		
32313599	SPINT1	kunitz-type protease inhibitor 1 isoform 1 precursor	1.7	0	1	2		58.4								
									EGFINYLTR	2	N-Term(iTRAQ4plex		3.33	35	2	628.84
27597078	TYRO3	tyrosine-protein kinase receptor TYRO3	1.69	1	1	2	1.062	96.8								
									GLAPASAPQNLHAIF	2	N-Term(iTRAQ4plex	1.062	3.74	63	3	553.98
116325989	HCN1	potassium/sodium hyperpolarization-activated cyclic nucleotide-gated channel 1	1.69	1	1	1	0.835	98.7								
									GVPPAPPPPAAALPF	1	N-Term(iTRAQ4plex	0.835		40		
28178825	IDH1	isocitrate dehydrogenase [NADP] cytoplasmic	1.69	1	1	1	0.656	46.6								
									IIWELIK	1	N-Term(iTRAQ4plex); K7(iTRAQ4plex	0.656		45		
14917115	KRT31	keratin, type I cuticular Ha1	1.68	0	1	1		47.2								
									LASYLEK	1	N-Term(iTRAQ4plex); K7(iTRAQ4plex			46		
61888896	ROBO2	roundabout homolog 2 isoform ROBO2b	1.67	0	2	2		151.1								

									MEASATLTVF	1	N-Term(iTRAQ4plex			55		
157502212	GALNT7	N-acetylgalactosaminyltransferase 7	1.67	1	1	6	0.95	75.3								
									AKPLVLGPEFK	6	N-Term(iTRAQ4plex); K2(iTRAQ4plex); K11(iTRAQ4plex)	0.95	5.37	70	3	544.35
25952118	CAMK2A	calcium/calmodulin-dependent protein kinase type II subunit alpha isoform 2	1.67	0	1	2		54.1								
									WQIVHFHR	2	N-Term(iTRAQ4plex			46		
28373129	CNTN5	contactin-5 isoform short	1.66	0	1	2		112.6								
									GQPIDFEEEGGHFESIR	2	N-Term(iTRAQ4plex		5.05	54	3	697.67
27894339	KRT23	keratin, type I cytoskeletal 23	1.66	0	1	1		48.1								
									LASYLEK	1	N-Term(iTRAQ4plex); K7(iTRAQ4plex			46		
26051206	CAMK2B	calcium/calmodulin-dependent protein kinase type II subunit beta isoform 2	1.66	0	1	1		60.3								
									FYFENLLAK	1	N-Term(iTRAQ4plex); K9(iTRAQ4plex			45		
20149594	HSP90AB1	heat shock protein HSP 90-beta	1.66	0	1	1		83.2								
									ELISNASDALDK	1	N-Term(iTRAQ4plex); K12(iTRAQ4plex			60		
193083163	ROBO2	roundabout homolog 2 isoform ROBO2a	1.65	0	2	2		153								
									MEASATLTVF	1	N-Term(iTRAQ4plex			55		
4506975	SLC12A2	solute carrier family 12 member 2	1.65	1	1	2	0.889	131.4								
									VELPGTAVPSVPEDAAPASF	2	N-Term(iTRAQ4plex	0.889	3.33	50	2	1054.06
310117657	LOC100508206	PREDICTED: bile salt-activated lipase-like isoform 5, partial	1.64	0	1	1		47.1								
									NPLFWAK	1	N-Term(iTRAQ4plex); K7(iTRAQ4plex			47		
113413200	POTEJ	PREDICTED: POTE ankyrin domain family member J	1.64	0	2	4		117.3								
									IWHHTFYNELR	3	N-Term(iTRAQ4plex			41		
									GILTLK	1	N-Term(iTRAQ4plex); K6(iTRAQ4plex			31		
25952114	CAMK2A	calcium/calmodulin-dependent protein kinase type II subunit alpha isoform 1	1.64	0	1	2		55.3								
									WQIVHFHR	2	N-Term(iTRAQ4plex			46		
4758022	COCH	cochlin precursor	1.64	1	1	1	0.863	59.4								
									WSASFTVTK	1	N-Term(iTRAQ4plex); K9(iTRAQ4plex	0.863		64		
55956794	NTRK3	NT-3 growth factor receptor isoform c precursor	1.63	0	1	3		68.4								
									VVSLEEPELR	3	N-Term(iTRAQ4plex		4.21	65	2	657.87
16306537	CDH20	cadherin-20 preproprotein	1.62	1	1	2	0.97	88.9								
									VLDVNDNAPEFPF	2	N-Term(iTRAQ4plex	0.97	4.56	69	2	815.42
125490370	KRT40	keratin, type I cytoskeletal 40	1.62	0	1	1		48.1								
									LASYLEK	1	N-Term(iTRAQ4plex); K7(iTRAQ4plex			46		
4505405	GPNMB	transmembrane glycoprotein NMB isoform b precursor	1.61	0	1	4		62.6								
									AYVPIAQVK	4	N-Term(iTRAQ4plex); K9(iTRAQ4plex		3.6	56	2	638.89
254028242	FGFR3	fibroblast growth factor receptor 3 isoform 3 precursor	1.61	0	1	1		88.1								
									VGPDGTPYVTVLK	1	N-Term(iTRAQ4plex); K13(iTRAQ4plex			63		
14917119	KRT34	keratin, type I cuticular Ha4	1.61	0	1	1		49.4								
									LASYLEK	1	N-Term(iTRAQ4plex); K7(iTRAQ4plex			46		
4503711	FGFR3	fibroblast growth factor receptor 3 isoform 1 precursor	1.61	0	1	1		87.7								
									VGPDGTPYVTVLK	1	N-Term(iTRAQ4plex); K13(iTRAQ4plex			63		
268840382	IL1RAP	interleukin-1 receptor accessory protein isoform 3	1.6	0	1	2		78.6								
									QIQVFEDEPAR	2	N-Term(iTRAQ4plex		3.91	65	2	738.38
4759092	SEMA3B	semaphorin-3B isoform 1 precursor	1.6	0	1	4		83.1								
									ETAVEAAPALGF	4	N-Term(iTRAQ4plex		4.09	63	2	664.87
54607089	SEMA3B	semaphorin-3B isoform 2 precursor	1.6	0	1	4		83								
									ETAVEAAPALGF	4	N-Term(iTRAQ4plex		4.09	63	2	664.87
46249397	RRAGB	ras-related GTP-binding protein B long isoform	1.6	0	1	7		43.2								
									ISNIIK	7	N-Term(iTRAQ4plex); K6(iTRAQ4plex			35		
54312078	NTNG1	netrin-G1 isoform 3	1.6	0	1	2		49.3								
									IIHFEIK	2	N-Term(iTRAQ4plex); K7(iTRAQ4plex			41		
4885259	MSTN	growth/differentiation factor 8 precursor	1.6	1	1	2	1.011	42.7								
									IQILSK	2	N-Term(iTRAQ4plex); K6(iTRAQ4plex	1.011		41		
19718780	SEMA6B	semaphorin-6B precursor	1.58	1	1	2	0.934	95.2								
									LTPAEGADDLNIQF	2	N-Term(iTRAQ4plex	0.934	4.21	57	2	828.94
88953571	POTEI	PREDICTED: POTE ankyrin domain family member I isoform 2	1.58	0	2	4		121.2								
									IWHHTFYNELR	3	N-Term(iTRAQ4plex			41		
									GILTLK	1	N-Term(iTRAQ4plex); K6(iTRAQ4plex			31		
52694752	GPNMB	transmembrane glycoprotein NMB isoform a precursor	1.57	0	1	4		63.9								
									AYVPIAQVK	4	N-Term(iTRAQ4plex); K9(iTRAQ4plex		3.6	56	2	638.89
115527062	COL6A2	collagen alpha-2(VI) chain isoform 2C2 precursor	1.57	0	1	2		108.5								
									DVTVTAIGIGDMFHEK	2	N-Term(iTRAQ4plex); K16(iTRAQ4plex		5.42	80	3	674.36
30089980	CHRD	chordin precursor	1.57	1	1	1	1.749	102								
									GDGHTDFVALLTGPF	1	N-Term(iTRAQ4plex	1.749		38		
119703755	LAMB2	laminin subunit beta-2 precursor	1.56	2	2	3	0.959	195.9								
									YSEIEPSTEGEVIYR	2	N-Term(iTRAQ4plex	0.911	4.86	67	2	958.47
									LQELEGTYEENER	1	N-Term(iTRAQ4plex	1.01		72		
7657359	CNTN5	contactin-5 isoform long	1.55	0	1	2		120.6								
									GQPIDFEEEGGHFESIR	2	N-Term(iTRAQ4plex		5.05	54	3	697.67
24307911	SMPDL3A	acid sphingomyelinase-like phosphodiesterase 3a precursor	1.55	1	1	1	0.54	51.2								
									LIDIFQK	1	N-Term(iTRAQ4plex); K7(iTRAQ4plex	0.54		41		
311771742	MSANTD3-TMEFF1	C9orf30-TMEFF1 fusion protein	1.54	0	1	1		51								
									EITVIAR	1	N-Term(iTRAQ4plex			34		
237681111	ATP1A1	sodium/potassium-transporting ATPase subunit alpha-1 isoform d	1.51	0	1	1		109.5								
									VDNSSLTGESEPQTF	1	N-Term(iTRAQ4plex			52		
240255478	SULF2	extracellular sulfatase Sulf-2 isoform b precursor	1.5	0	1	12		100.1								
									VYHVGLGDAAQPF	12	N-Term(iTRAQ4plex		5.46	62	3	509.61
226246665	KRT72	keratin, type II cytoskeletal 72 isoform 2	1.49	0	1	4		51.1								
									FASFIDK	4	N-Term(iTRAQ4plex); K7(iTRAQ4plex		3.19	53	2	558.31
38195091	GALNT10	polypeptide N-acetylgalactosaminyltransferase 10	1.49	1	1	2	0.932	68.9								
									TFFLGDGQK	2	N-Term(iTRAQ4plex); K9(iTRAQ4plex	0.932	3.62	49	2	650.86

240255483	SULF2	extracellular sulfatase Sulf-2 isoform a precursor	1.49	0	1	12		100.4								
									VYHVGLGDAAQPF	12	N-Term(iTRAQ4plex		5.46	62	3	509.61
222446618	GALNT18	putative polypeptide N-acetylgalactosaminyltransferase-like protein 4	1.48	1	1	1	0.908	69.5								
									LDHLENVIK	1	N-Term(iTRAQ4plex); K9(iTRAQ4plex	0.908		40		
22748667	ATP1A3	sodium/potassium-transporting ATPase subunit alpha-3	1.48	0	1	1		111.7								
									VDNSSLTGESEPQTF	1	N-Term(iTRAQ4plex			52		
119393891	GAA	lysosomal alpha-glucosidase preproprotein	1.47	1	1	2	1.2	105.3								
									DFPAMVQELHQGGF	2	N-Term(iTRAQ4plex	1.2	4.68	60	3	576.96
237681109	ATP1A1	sodium/potassium-transporting ATPase subunit alpha-1 isoform c	1.47	0	1	1		112.9								
									VDNSSLTGESEPQTF	1	N-Term(iTRAQ4plex			52		
21361181	ATP1A1	sodium/potassium-transporting ATPase subunit alpha-1 isoform a	1.47	0	1	1		112.8								
									VDNSSLTGESEPQTF	1	N-Term(iTRAQ4plex			52		
270265857	KIAA0319	KIAA0319 isoform c	1.46	0	1	2		113								
									GSPSGIWGDSPEDIR	2	N-Term(iTRAQ4plex		4.17	64	2	858.92
164414437	NTNG1	netrin-G1 isoform 2	1.46	0	1	2		53.9								
									IIHFEIK	2	N-Term(iTRAQ4plex); K7(iTRAQ4plex			41		
51944966	ATP4A	potassium-transporting ATPase alpha chain 1	1.45	0	1	1		114								
									VDNSSLTGESEPQTF	1	N-Term(iTRAQ4plex			52		
88703041	SNX6	sorting nexin-6 isoform b	1.44	0	1	1		47.8								
									LSDLLK	1	N-Term(iTRAQ4plex); K6(iTRAQ4plex			33		
223718246	PLS1	plastin-1	1.43	0	1	2		70.2								
									LSPEELLLR	2	N-Term(iTRAQ4plex		3.44	42	2	607.37
4557699	KRT12	keratin, type I cytoskeletal 12	1.42	0	1	4		53.5								
									LASYLDK	4	N-Term(iTRAQ4plex); K7(iTRAQ4plex		3.35	53	2	549.32
18641362	PTPRC	receptor-type tyrosine-protein phosphatase C isoform 2 precursor	1.4	0	1	1		130.8								
									SEAAHQGVITWNPPQF	1	N-Term(iTRAQ4plex			56		
28372503	KRT72	keratin, type II cytoskeletal 72 isoform 1	1.37	0	1	4		55.8								
									FASFIDK	4	N-Term(iTRAQ4plex); K7(iTRAQ4plex		3.19	53	2	558.31
21264359	MASP1	mannan-binding lectin serine protease 1 isoform 2 precursor	1.37	1	1	2	1.099	81.8								
									TLSDVLQYVK	2	N-Term(iTRAQ4plex); K10(iTRAQ4plex	1.099	3.64	82	2	727.43
134244281	MFI2	melanotransferrin isoform 1 precursor	1.36	1	1	1	1.001	80.2								
									ADTDGGLIFR	1	N-Term(iTRAQ4plex	1.001		40		
26051204	CAMK2B	calcium/calmodulin-dependent protein kinase type II subunit beta isoform 1	1.35	0	1	1		72.6								
									FYFENLLAK	1	N-Term(iTRAQ4plex); K9(iTRAQ4plex			45		
15618995	KRT71	keratin, type II cytoskeletal 71	1.34	0	1	4		57.3								
									FASFIDK	4	N-Term(iTRAQ4plex); K7(iTRAQ4plex		3.19	53	2	558.31
310117655	LOC100508206	PREDICTED: bile salt-activated lipase-like isoform 3	1.34	0	1	1		56.4								
									NPLFWAK	1	N-Term(iTRAQ4plex); K7(iTRAQ4plex			47		
148612803	KRT74	keratin, type II cytoskeletal 74	1.32	0	1	4		57.8								
									FASFIDK	4	N-Term(iTRAQ4plex); K7(iTRAQ4plex		3.19	53	2	558.31
28173564	KRT73	keratin, type II cytoskeletal 73	1.3	0	1	4		58.9								
									FASFIDK	4	N-Term(iTRAQ4plex); K7(iTRAQ4plex		3.19	53	2	558.31
164414431	NTNG1	netrin-G1 isoform 1	1.3	0	1	2		60.5								
									IIHFEIK	2	N-Term(iTRAQ4plex); K7(iTRAQ4plex			41		
122937283	SNED1	sushi, nidogen and EGF-like domain-containing protein 1 precursor	1.27	1	1	2	0.945	152.1								
									TADMAEVETTTNVGVPGI	2	N-Term(iTRAQ4plex	0.945	4.22	74	2	996.50
221316695	MMRN2	multimerin-2 precursor	1.26	1	1	2	0.859	104.3								
									VWFELTQGSITK	2	N-Term(iTRAQ4plex); K12(iTRAQ4plex	0.859	3.65	79	2	848.98
224589135	RGMB	RGM domain family member E	1.26	1	1	8	0.989	51.9								
									ITIIFK	8	N-Term(iTRAQ4plex); K6(iTRAQ4plex	0.989		42		
222418615	ITGA7	integrin alpha-7 isoform 3	1.25	0	1	3		114.5								
									ELEPPEQQEPGER	3	N-Term(iTRAQ4plex		3.88	66	2	841.41
38638698	KIAA1199	KIAA1199 precursor	1.25	2	2	2	0.812	152.9								
									TLPIGQNFPIR	1	N-Term(iTRAQ4plex	0.793		38		
									FALGFK	1	N-Term(iTRAQ4plex); K6(iTRAQ4plex	0.831		35		
18201905	GPI	glucose-6-phosphate isomerase isoform 2	1.25	0	1	1		63.1								
									MLVDLAK	1	N-Term(iTRAQ4plex); K7(iTRAQ4plex			49		
310125282	LOC100507699	PREDICTED: vesicle-fusing ATPase-like	1.24	0	1	1		61.4								
									LFGLLVK	1	N-Term(iTRAQ4plex); K7(iTRAQ4plex			42		
296080693	GPI	glucose-6-phosphate isomerase isoform 1	1.23	0	1	1		64.3								
									MLVDLAK	1	N-Term(iTRAQ4plex); K7(iTRAQ4plex			49		
18641347	PTPRC	receptor-type tyrosine-protein phosphatase C isoform 1 precursor	1.23	0	1	1		147.2								
									SEAAHQGVITWNPPQF	1	N-Term(iTRAQ4plex			56		
72534658	IFIT3	interferon-induced protein with tetratricopeptide repeats 3	1.22	0	1	1		55.9								
									VLLGLK	1	N-Term(iTRAQ4plex); K6(iTRAQ4plex			32		
188497750	HK1	hexokinase-1 isoform HKI-td	1.22	0	1	1		101								
									FNTSDVSAIEK	1	N-Term(iTRAQ4plex); K11(iTRAQ4plex			69		
59889560	NTRK3	NT-3 growth factor receptor isoform b precursor	1.21	0	1	3		92.7								
									VVSLEEPELR	3	N-Term(iTRAQ4plex		4.21	65	2	657.87
105990532	APOB	apolipoprotein B-100 precursor	1.21	6	6	9	1.441	515.2								
									SEYQADYESLR	2	N-Term(iTRAQ4plex	1.417	4.8	52	2	752.86
									SPAFTDLHLR	2	N-Term(iTRAQ4plex	1.466	4.09	50	3	434.24
									YENYELTLK	2	N-Term(iTRAQ4plex); K9(iTRAQ4plex	1.272	4.06	74	2	730.90
									HINIDQFVR	1	N-Term(iTRAQ4plex	1.27		37		
									SLWDFLK	1	N-Term(iTRAQ4plex); K7(iTRAQ4plex	1.637		37		
110611237	F13B	coagulation factor XIII B chain precursor	1.21	1	1	1	0.991	75.5								
									CNEYYLLR	1	N-Term(iTRAQ4plex); C1(Methylthio	0.991		35		
66932918	MERTK	tyrosine-protein kinase Mer precursor	1.2	1	1	2	1.057	110.2								
									AIPSPPTEVSIR	2	N-Term(iTRAQ4plex	1.057	3.5	50	2	705.91
4506467	RDX	radixin	1.2	0	1	2		68.5								
									LFFLQVK	2	N-Term(iTRAQ4plex); K7(iTRAQ4plex			49		
17975768	EPHB3	ephrin type-B receptor 3 precursor	1.2	1	1	1	0.886	110.3								
									VDTIAPDESFSR	1	N-Term(iTRAQ4plex	0.886		44		
15991827	HK1	hexokinase-1 isoform HKI-R	1.2	0	1	1		102.1								
									FNTSDVSAIEK	1	N-Term(iTRAQ4plex); K11(iTRAQ4plex			69		
188497754	HK1	hexokinase-1 isoform HKI	1.2	0	1	1		102.4								

									FNTSDVSAIEK	1	N-Term(iTRAQ4plex); K11(iTRAQ4plex			69		
59889562	NTRK3	NT-3 growth factor receptor isoform a precursor	1.19	0	1	3		94.4								
									VVSLEEPELR	3	N-Term(iTRAQ4plex		4.21	65	2	657.87
148539848	DSC3	desmocollin-3 isoform Dsc3b preproprotein	1.19	0	1	1		93.4								
									EVTVLLEHQK	1	N-Term(iTRAQ4plex); K10(iTRAQ4plex			48		
40217825	SLITRK6	SLIT and NTRK-like protein 6 precursor	1.19	0	1	3		95								
									FVPLTHLDLR	3	N-Term(iTRAQ4plex			53		
223005864	CPXM2	inactive carboxypeptidase-like protein X2 precursor	1.19	0	1	1		85.8								
									FTGVITQGR	1	N-Term(iTRAQ4plex			36		
15991831	HK1	hexokinase-1 isoform HKI-ta/tb	1.19	0	1	1		102.7								
									FNTSDVSAIEK	1	N-Term(iTRAQ4plex); K11(iTRAQ4plex			69		
21614499	EZR	ezrin	1.19	0	1	2		69.4								
									LFFLQVK	2	N-Term(iTRAQ4plex); K7(iTRAQ4plex			49		
15431316	KRT84	keratin, type II cuticular Hb4	1.17	0	1	4		64.8								
									FASFIDK	4	N-Term(iTRAQ4plex); K7(iTRAQ4plex		3.19	53	2	558.31
55743096	COL14A1	collagen alpha-1(XIV) chain precursor	1.17	2	2	2	0.994	193.4								
									ETLLDAIK	1	N-Term(iTRAQ4plex); K8(iTRAQ4plex	1.134		50		
296179442	SEZ6L	seizure 6-like protein isoform 5 precursor	1.16	0	1	2		103.4								
									SPTNTISVYFR	2	N-Term(iTRAQ4plex		4.83	67	2	714.88
11968148	SLC12A5	solute carrier family 12 member 5 isoform 2	1.16	0	1	2		123.4								
									ESSPFINSTDTEK	2	N-Term(iTRAQ4plex); K13(iTRAQ4plex		4.07	77	2	871.94
119964726	IGF2R	cation-independent mannose-6-phosphate receptor precursor	1.16	2	2	4	0.986	274.1								
									TTTGDVQVLGLVHTQI	2	N-Term(iTRAQ4plex); K16(iTRAQ4plex	1.035	4.47	49	3	662.38
									FLHQDIDSGQGIR	2	N-Term(iTRAQ4plex	0.939	4.37	50	3	543.95
56711320	C14orf37	hypothetical protein LOC145407 precursor	1.16	1	1	1	0.945	84.1								
									EIAHVHAEK	1	N-Term(iTRAQ4plex); K9(iTRAQ4plex	0.945		41		
198041678	SLC12A5	solute carrier family 12 member 5 isoform 1	1.14	0	1	2		126.1								
									ESSPFINSTDTEK	2	N-Term(iTRAQ4plex); K13(iTRAQ4plex		4.07	77	2	871.94
222418611	ITGA7	integrin alpha-7 isoform 2 precursor	1.14	0	1	3		124.2								
									ELEPPEQQEPGER	3	N-Term(iTRAQ4plex		3.88	66	2	841.41
222418613	ITGA7	integrin alpha-7 isoform 1 precursor	1.14	0	1	3		124.6								
									ELEPPEQQEPGER	3	N-Term(iTRAQ4plex		3.88	66	2	841.41
148539846	DSC3	desmocollin-3 isoform Dsc3a preproprotein	1.12	0	1	1		99.9								
									EVTVLLEHQK	1	N-Term(iTRAQ4plex); K10(iTRAQ4plex			48		
310117651	LOC100508206	PREDICTED: bile salt-activated lipase-like isoform 2	1.1	0	1	1		68.7								
									NPLFWAK	1	N-Term(iTRAQ4plex); K7(iTRAQ4plex			47		
310117649	LOC100508206	PREDICTED: bile salt-activated lipase-like isoform 1	1.08	0	1	1		69.7								
									NPLFWAK	1	N-Term(iTRAQ4plex); K7(iTRAQ4plex			47		
66346698	NAGLU	alpha-N-acetylglucosaminidase precursor	1.08	1	1	1	0.93	82.2								
									SDVFEAWR	1	N-Term(iTRAQ4plex	0.93		36		
116008152	COL15A1	collagen alpha-1(XV) chain precursor	1.08	1	1	1	1.204	141.6								
									AFLSSHLQDLSTIVR	1	N-Term(iTRAQ4plex	1.204		63		
88758613	CD93	complement component C1q receptor precursor	1.07	0	1	2		68.5								
									VLAQLLR	2	N-Term(iTRAQ4plex			32		
240255542	COL6A3	collagen alpha-3(VI) chain isoform 2 precursor	1.06	0	1	2		113.2								
									IAVAQYSDDVK	2	N-Term(iTRAQ4plex); K11(iTRAQ4plex		3.88	79	2	748.92
32261318	UNC5B	netrin receptor UNC5B precursor	1.06	1	1	1	0.823	103.6								
									AFPATQIYFK	1	N-Term(iTRAQ4plex); K10(iTRAQ4plex	0.823		69		
310117653	LOC100508206	PREDICTED: bile salt-activated lipase-like isoform 4	1.04	0	1	1		72.3								
									NPLFWAK	1	N-Term(iTRAQ4plex); K7(iTRAQ4plex			47		
210147493	TMEM108	transmembrane protein 108 precursor	1.04	1	1	1	1.048	59.9								
									IFQIYK	1	N-Term(iTRAQ4plex); K6(iTRAQ4plex	1.048		34		
5803229	ZBTB33	transcriptional regulator Kaiso	1.04	1	1	1	1.03	74.4								
									LENEIPK	1	N-Term(iTRAQ4plex); K7(iTRAQ4plex	1.03		37		
40217820	SLITRK3	SLIT and NTRK-like protein 3 precursor	1.02	1	1	1	0.841	108.9								
									AVSLTHLDLR	1	N-Term(iTRAQ4plex	0.841		46		
46560555	TMEM132E	transmembrane protein 132E precursor	1.02	1	1	1	0.793	107								
									AVTELTVIQR	1	N-Term(iTRAQ4plex	0.793		40		
155969705	CLCNKB	chloride channel protein ClC-Kb isoform 1	1.02	1	1	2	0.909	75.4								
									GGLEWLK	2	N-Term(iTRAQ4plex); K7(iTRAQ4plex	0.909		38		
28916698	C5orf54	transposon-derived Buster3 transposase-like protein	1.01	1	1	1	0.973	68.3								
									VINFIK	1	N-Term(iTRAQ4plex); K6(iTRAQ4plex	0.973		42		
54112397	CACNA2D3	voltage-dependent calcium channel subunit alpha-2/delta-3	1.01	1	1	1	0.67	122.9								
									LWASAFGGEIK	1	N-Term(iTRAQ4plex); K11(iTRAQ4plex	0.67		75		
153792110	HEG1	protein HEG homolog 1 precursor	1.01	1	1	1	0.619	147.4								
									TMHVATVFTDGGPF	1	N-Term(iTRAQ4plex	0.619		33		
4503273	ACE	angiotensin-converting enzyme isoform 1 precursor	1	0	1	2		149.6								
									AALPAQELEEYNK	2	N-Term(iTRAQ4plex); K13(iTRAQ4plex		3.31	82	2	882.47
21265037	ADAMTS3	A disintegrin and metalloproteinase with thrombospondin motifs 3 preproprotein	1	1	1	4	0.755	135.5								
									SAVEQAPIDMSK	4	N-Term(iTRAQ4plex); K12(iTRAQ4plex	0.755	4.18	75	2	782.42
21264332	ST6GALNAC1	alpha-N-acetylgalactosaminide alpha-2,6-sialyltransferase 1	1	1	1	2	1.004	68.5								
									WDFEEK	2	N-Term(iTRAQ4plex); K6(iTRAQ4plex	1.004		37		
4507157	SORL1	sortilin-related receptor preproprotein	0.99	2	2	3	0.905	248.3								
									ASNLLLGFDR	2	N-Term(iTRAQ4plex	0.923	3.29	41	2	625.36
									YFANEPFADFHR	1	N-Term(iTRAQ4plex	0.887		55		
75709184	CNTN3	contactin-3 precursor	0.97	1	1	2	0.912	112.8								
									ISLLNDGGLK	2	N-Term(iTRAQ4plex); K10(iTRAQ4plex	0.912	4.25	44	2	659.40
145309328	PAPLN	papilin	0.96	1	1	4	1.039	134.8								
									VVGCDHELDSSK	4	N-Term(iTRAQ4plex); C4(Methylthio); K12(iTRAQ4plex)	1.039	4.47	69	3	541.60
51173878	TRMT2A	tRNA (uracil-5-)-methyltransferase homolog A	0.96	1	1	15	0.867	68.7								
									VILAIR	15	N-Term(iTRAQ4plex	0.867		35		
156564401	NSF	vesicle-fusing ATPase	0.94	0	1	1		82.5								
									LFGLLVK	1	N-Term(iTRAQ4plex); K7(iTRAQ4plex			42		
148536848	CEL	bile salt-activated lipase precursor	0.93	0	1	1		79.6								
									NPLFWAK	1	N-Term(iTRAQ4plex); K7(iTRAQ4plex			47		

4557040	OSMR	oncostatin-M-specific receptor subunit beta isoform 1 precursor	0.92	0	1	1		110.4								
									LPLTPVSLK	1	N-Term(iTRAQ4plex); K9(iTRAQ4plex			53		
148539860	PTPRT	receptor-type tyrosine-protein phosphatase T isoform 2 precursor	0.9	0	1	2		162								
									FSATVSVADTAQF	2	N-Term(iTRAQ4plex		4.83	78	2	748.90
240255540	COL6A3	collagen alpha-3(VI) chain isoform 3 precursor	0.89	0	1	2		134.6								
									IAVAQYSDDVK	2	N-Term(iTRAQ4plex); K11(iTRAQ4plex		3.88	79	2	748.92
148539858	PTPRT	receptor-type tyrosine-protein phosphatase T isoform 1 precursor	0.89	0	1	2		163.9								
									FSATVSVADTAQF	2	N-Term(iTRAQ4plex		4.83	78	2	748.90
29336063	PLXNB3	plexin-B3 isoform 1	0.89	0	1	2		206.7								
									GPSGAEEATVEYGVTSF	2	N-Term(iTRAQ4plex		4.15	67	2	927.45
116174738	PAPPA2	pappalysin-2 isoform 1	0.89	0	1	1		198.4								
									YQPGTWTHVAATYDGF	1	N-Term(iTRAQ4plex			34		
253795481	PLXNB3	plexin-B3 isoform 2	0.88	0	1	2		209.2								
									GPSGAEEATVEYGVTSF	2	N-Term(iTRAQ4plex		4.15	67	2	927.45
14192943	MEGF10	multiple epidermal growth factor-like domains protein 10 precursor	0.88	1	1	1	0.829	122.1								
									ICSPGFYGHR	1	N-Term(iTRAQ4plex); C2(Methylthio	0.829		42		
110624774	MRC2	C-type mannose receptor 2	0.88	1	1	1	1.032	166.6								
									TLGDQLSLLLGAR	1	N-Term(iTRAQ4plex	1.032		72		
47519929	CNTNAP3	contactin-associated protein-like 3 precursor	0.85	0	1	2		140.6								
									YQWLQIDLGER	2	N-Term(iTRAQ4plex		4.04	44	2	782.91
319084483	CNTNAP3B	contactin-associated protein-like 3B	0.85	0	1	2		140.4								
									YQWLQIDLGER	2	N-Term(iTRAQ4plex		4.04	44	2	782.91
122891870	MIA3	melanoma inhibitory activity protein 3 precursor	0.84	1	1	2	1.135	213.6								
									FGSTADALVSDDETTF	2	N-Term(iTRAQ4plex	1.135	5.11	66	2	914.94
18640734	CNTNAP5	contactin-associated protein-like 5 precursor	0.84	1	1	2	0.771	145.5								
									VEITAVATQGF	2	N-Term(iTRAQ4plex	0.771	4.14	50	2	644.87
296011008	SEMA3E	semaphorin-3E isoform 2	0.84	0	1	3		82.4								
									WSTFLK	3	N-Term(iTRAQ4plex); K6(iTRAQ4plex			42		
214010220	SEMA4D	semaphorin-4D isoform 2	0.81	0	1	1		82.1								
									WTSFLK	1	N-Term(iTRAQ4plex); K6(iTRAQ4plex			38		
116235485	DNER	delta and Notch-like epidermal growth factor-related receptor precursor	0.81	1	1	2	1.093	78.4								
									LTLVVK	2	N-Term(iTRAQ4plex); K6(iTRAQ4plex	1.093		44		
6912650	SEMA3E	semaphorin-3E isoform 1 precursor	0.77	0	1	3		89.2								
									WSTFLK	3	N-Term(iTRAQ4plex); K6(iTRAQ4plex			42		
188497703	SEMA3F	semaphorin-3F precursor	0.76	0	1	3		88.3								
									WSTFLK	3	N-Term(iTRAQ4plex); K6(iTRAQ4plex			42		
27477041	AP2A2	AP-2 complex subunit alpha-2	0.75	0	1	1		103.9								
									LINNAIK	1	N-Term(iTRAQ4plex); K7(iTRAQ4plex			33		
310129361	LOC100290337	PREDICTED: DNA damage-binding protein 1 like isoform 6	0.73	0	1	1		91.9								
									GLWPLR	1	N-Term(iTRAQ4plex			34		
6005902	THBS3	thrombospondin-3 precursor	0.73	0	1	1		104.1								
									FYVVMWK	1	N-Term(iTRAQ4plex); K7(iTRAQ4plex			43		
19913416	AP2A1	AP-2 complex subunit alpha-1 isoform 2	0.73	0	1	1		105.3								
									LINNAIK	1	N-Term(iTRAQ4plex); K7(iTRAQ4plex			33		
19913414	AP2A1	AP-2 complex subunit alpha-1 isoform 1	0.72	0	1	1		107.5								
									LINNAIK	1	N-Term(iTRAQ4plex); K7(iTRAQ4plex			33		
28872814	SEMA4G	semaphorin-4G	0.71	0	1	1		92								
									WTSFLK	1	N-Term(iTRAQ4plex); K6(iTRAQ4plex			38		
214010218	SEMA4D	semaphorin-4D isoform 1	0.7	0	1	1		96.1								
									WTSFLK	1	N-Term(iTRAQ4plex); K6(iTRAQ4plex			38		
115387099	BAI2	brain-specific angiogenesis inhibitor 2 precursor	0.69	1	1	2	0.825	172.5								
									QEQVCAHFAPF	2	N-Term(iTRAQ4plex); C5(Methylthio	0.825	4.51	39	3	492.57
261337173	LTBP1	latent-transforming growth factor beta-binding protein 1 isoform 5 precursor	0.69	0	1	1		142.6								
									EIPSLDQEK	1	N-Term(iTRAQ4plex); K9(iTRAQ4plex			40		
261337171	LTBP1	latent-transforming growth factor beta-binding protein 1 isoform 3 precursor	0.67	0	1	1		147.1								
									EIPSLDQEK	1	N-Term(iTRAQ4plex); K9(iTRAQ4plex			40		
261337169	LTBP1	latent-transforming growth factor beta-binding protein 1 isoform 4 precursor	0.67	0	1	1		148.3								
									EIPSLDQEK	1	N-Term(iTRAQ4plex); K9(iTRAQ4plex			40		
261337167	LTBP1	latent-transforming growth factor beta-binding protein 1 isoform LTBP-1S precursor	0.65	0	1	1		152.8								
									EIPSLDQEK	1	N-Term(iTRAQ4plex); K9(iTRAQ4plex			40		
54234040	VPS54	vacuolar protein sorting-associated protein 54 isoform 2	0.62	0	1	1		109.3								
									LSDLLK	1	N-Term(iTRAQ4plex); K6(iTRAQ4plex			33		
54234034	VPS54	vacuolar protein sorting-associated protein 54 isoform 1	0.61	0	1	1		110.5								
									LSDLLK	1	N-Term(iTRAQ4plex); K6(iTRAQ4plex			33		
162809334	PZP	pregnancy zone protein precursor	0.61	0	1	1		163.8								
									ATVLNYLPK	1	N-Term(iTRAQ4plex); K9(iTRAQ4plex			54		
310129363	LOC100290337	PREDICTED: DNA damage-binding protein 1 like isoform 5	0.59	0	1	1		112.1								
									GLWPLR	1	N-Term(iTRAQ4plex			34		
256017257	ACAN	aggrecan core protein isoform 2 precursor	0.59	1	1	1	0.837	261.2								
									GTVACGEPPVVEHAF	1	N-Term(iTRAQ4plex); C5(Methylthio	0.837		37		
24041035	NOTCH2	neurogenic locus notch homolog protein 2 isoform 1 preproprotein	0.57	1	1	5	0.95	265.2								
									SLPGEQEQEVAGSK	5	N-Term(iTRAQ4plex); K14(iTRAQ4plex	0.95	5.06	91	2	873.96
32130516	NEMF	serologically defined colon cancer antigen 1	0.56	1	1	1	1.011	122.9								
									AIQVVR	1	N-Term(iTRAQ4plex	1.011		34		
310129365	LOC100290337	PREDICTED: DNA damage-binding protein 1 like isoform 4	0.55	0	1	1		121.6								
									GLWPLR	1	N-Term(iTRAQ4plex			34		
310129356	LOC100290337	PREDICTED: DNA damage-binding protein 1 like isoform 2	0.53	0	1	1		126.9								
									GLWPLR	1	N-Term(iTRAQ4plex			34		
87162455	CLUH	hypothetical protein LOC23277	0.53	0	1	1		146.6								
									VLELVLR	1	N-Term(iTRAQ4plex			32		

261337165	LTBP1	latent-transforming growth factor beta-binding protein 1 isoform LTBP-1L precursor	0.52	0	1	1		186.6								
									EIPSLDQEK	1	N-Term(iTRAQ4plex); K9(iTRAQ4plex			40		
22202611	CPD	carboxypeptidase D isoform 1 precursor	0.51	1	1	1	0.922	152.8								
									ALIEWIR	1	N-Term(iTRAQ4plex	0.922		38		
30581135	SMC1A	structural maintenance of chromosomes protein 1A	0.49	1	1	1	0.938	143.1								
									IDEINK	1	N-Term(iTRAQ4plex); K6(iTRAQ4plex	0.938		38		
239582741	FRMPD1	FERM and PDZ domain-containing protein 1	0.44	0	1	19		173.3								
									DIILTVK	34	N-Term(iTRAQ4plex); K7(iTRAQ4plex		3.58	46	2	545.36
45446740	ABCA2	ATP-binding cassette sub-family A member 2 isoform a	0.37	0	1	2		269.8								
									ILTVPESQK	2	N-Term(iTRAQ4plex); K9(iTRAQ4plex		3.41	46	2	651.89
47078218	ABCA2	ATP-binding cassette sub-family A member 2 isoform b	0.36	0	1	2		272.8								
									ILTVPESQK	2	N-Term(iTRAQ4plex); K9(iTRAQ4plex		3.41	46	2	651.89
154146189	DOCK10	dedicator of cytokinesis protein 10	0.32	0	2	5		249.4								
									KYAFELK	3	N-Term(iTRAQ4plex); K1(iTRAQ4plex); K7(iTRAQ4plex)		4.1	49	3	444.28
									YAFELK	2	N-Term(iTRAQ4plex); K6(iTRAQ4plex			42		
46240864	SPAG17	sperm-associated antigen 17	0.31	1	1	1		251.6								
									VVIVERK	1	N-Term(iTRAQ4plex); K7(iTRAQ4plex			42		
93102379	LRP1B	low-density lipoprotein receptor-related protein 1B precursor	0.26	1	1	1	0.697	515.2								
									TTLIAGAMEHPF	1	N-Term(iTRAQ4plex	0.697		48		
55770834	CENPF	centromere protein F	0.22	0	1	3		357.3								
									LEIDLLK	3	N-Term(iTRAQ4plex); K7(iTRAQ4plex		3.28	39	2	566.36
222144249	DNHD1	dynein heavy chain domain 1 isoform 1	0.13	1	1	1	0.986	533.3								
									LLLALR	1	N-Term(iTRAQ4plex	0.986		25		
91718902	MLL3	histone-lysine N-methyltransferase MLL3	0.1	0	1	1		541								
									CAFCK	1	N-Term(iTRAQ4plex); C1(Methylthio); C4(Methylthio); K5(iTRAQ4plex)			27		
