# Supplementary material for: Chitotriosidase - a putative biomarker for sporadic amyotrophic lateral sclerosis
Source: Clin Proteomics. 2013 Dec 2;10(1):19. doi: 10.1186/1559-0275-10-19 (PMC4220794; doi:10.1186/1559-0275-10-19)
Supplement: Additional file 2: Table S2 — Up-regulated proteins in ALS-CSF. Description of data: Table showing 31 up-regulated proteins with more than 1.5-fold increase in ALS-CSF compared to normal CSF. [file 1559-0275-10-19-S2.pdf]

Table S2: Up-regulated proteins in ALS-CSF

SNO	Accession	GENESYMBOL	Description	ΣCoverage	Σ#	Σ# Unique Peptides	Σ# Peptides	Σ# PSMs	ALS/Normal	A5: 115/114 Count	A5: 115/114 Variability [%]	# Peptides (Sequest)	# Peptides (Mascot)	MW [kDa]	calc. pI
1	4502809	CHIT1	chitotriosidase-1 precursor [Homo sapiens]	5.58	1	2	2	4	**10.177**	2	32.0	2	2	51.6	6.96
2	4759166	SPP1	osteopontin isoform b precursor [Homo sapiens]	45.33	1	1	10	158	**2.997**	9	26.5	10	9	33.8	4.61
3	4506121	PROZ	vitamin K-dependent protein Z precursor [Homo sapiens]	2.50	1	1	1	1	**2.791**	1			1	44.7	5.97
4	47132620	KRT2	keratin, type II cytoskeletal 2 epidermal [Homo sapiens]	21.28	1 1	10	13	53	**2.534**	20	27.9	12	11	65.4	8.00
5	13899303	CRISPLD1	cysteine-rich secretory protein	2.40	1	1	1	1	**2.447**	1			1	56.9	8.12

			LCCL domain-containing 1 [Homo sapiens]												
6	195972866	KRT10	keratin, type I cytoskeletal 10 [Homo sapiens]	23.97	5	11	12	69	**2.319**	37	39.2	10	11	58.8	5.21
7	27894337	KRT20	keratin, type I cytoskeletal 20 [Homo sapiens]	5.19	10	1	3	4	**2.137**	1		1	3	48.5	5.69
8	15431310	KRT14	keratin, type I cytoskeletal 14 [Homo sapiens]	9.96	7	3	5	13	**2.124**	4	17.5	4	5	51.6	5.16
9	68533260	CHI3L2	chitinase-3-like protein 2 isoform c [Homo sapiens]	20.90	3	5	5	11	**2.113**	6	33.8	4	5	34.6	7.97
10	4503625	F10	coagulation factor X preproprotein [Homo sapien	6.15	1	2	2	5	**2.036**	3	6.7	2	2	54.7	5.94

			s]												
11	119395750	KRT1	keratin, type II cytoskeletal 1 [Homo sapiens]	29.81	5	16	17	100	**1.988**	51	21.8	13	17	66.0	8.12
12	4505405	GPNMB	transmembrane glycoprotein NMB isoform b precursor [Homo sapiens]	1.61	2	1	1	4	**1.975**	2	26.2	1	1	62.6	6.64
13	4501945	ADM	ADM precursor [Homo sapiens]	7.03	1	1	1	2	**1.939**	1		1	1	20.4	10.84
14	91206462	SPP1	osteopontin isoform a precursor [Homo sapiens]	45.54	1	1	10	144	**1.904**	1		10	9	35.4	4.58
15	30089980	CHRD	chordin precursor [Homo sapiens]	1.57	1	1	1	1	**1.894**	1			1	102.0	7.75
16	91598939	SPP1	osteopontin isoform c precursor [Homo sapien	42.16	1	1	9	136	**1.883**	1		9	8	32.3	4.55

			s]												
17	221316738	GAS6	growth arrest-specific protein 6 isoform 3 [Homo sapiens]	5.28	3	2	2	3	**1.875**	2	22.8	1	2	42.0	6.47
18	4506115	PROC	vitamin K-dependent protein C preproprotein [Homo sapiens]	10.85	1	4	4	7	**1.873**	4	25.5	3	4	52.0	6.28
19	4826950	KLK7	kallikrein-7 preproprotein [Homo sapiens]	3.56	1	1	1	2	**1.829**	1		1	1	27.5	8.47
20	118442839	CFHR1	complement factor H-related protein 1 precursor [Homo sapiens]	16.06	1	1	5	32	**1.795**	1		3	5	37.6	7.39
21	5031839	KRT6A	keratin, type II cytoskeletal 6A [Homo sapiens]	10.28	9	3	6	15	**1.777**	3	34.1	6	5	60.0	8.00
22	1442262	CHI3L1	chitina	41.78	1	12	12	125	**1.7**	72	15.8	12	12	42.6	8.46

	51		se-3-like protein 1 precursor [Homo sapiens]						**13**						
23	119395754	KRT5	keratin, type II cytoskeletal 5 [Homo sapiens]	8.81	8	2	5	12	**1.642**	2	6.8	4	4	62.3	7.74
24	4503635	F2	prothrombin preproprotein [Homo sapiens]	42.28	1	20	20	122	**1.622**	75	24.4	14	20	70.0	5.90
25	258613951	ASPH	aspartyl/asparaginyl beta-hydroxylase isoform i [Homo sapiens]	9.38	13	2	2	3	**1.618**	2	0.9	1	2	28.9	4.16
26	13129000	METRN	meteorin precursor [Homo sapiens]	4.78	1	1	1	1	**1.596**	1			1	31.2	8.09
27	10190664	TWSG1	twisted gastrulation protein homolog 1 precursor [Homo	6.73	1	1	1	2	**1.591**	1		1	1	25.0	5.34

			sapien s]												
28	312433998	LTF	lactotransferrin isoform 2 [Homo sapiens]	3.90	2	2	2	4	**1.581**	2	0.5	2	2	73.1	7.85
29	105990532	APOB	apolipoprotein B-100 precursor [Homo sapiens]	1.21	1	6	6	9	**1.561**	6	12.6	4	5	515.2	7.05
30	156523970	AHSG	alpha-2-HS-glycoprotein [Homo sapiens]	23.16	1	7	7	185	**1.533**	121	20.1	6	7	39.3	5.72
31	7657465	PODXL2	podocalyxin-like protein 2 precursor [Homo sapiens]	4.79	1	2	2	4	**1.524**	2	29.7	2	2	65.0	4.34
