# Supplementary material for: Chitotriosidase - a putative biomarker for sporadic amyotrophic lateral sclerosis
Source: Clin Proteomics. 2013 Dec 2;10(1):19. doi: 10.1186/1559-0275-10-19 (PMC4220794; doi:10.1186/1559-0275-10-19)
Supplement: Additional file 3: Table S3 — Proteins down-regulated in ALS-CSF. Description of Data: List of 17 down-regulated proteins which showed a decrease of 0.5 fold or more, in ALS-CSF samples. [file 1559-0275-10-19-S3.pdf]

Table S3: Down-regulated proteins in ALS-CSF

S N O	Accession	GENE SYMBOL	Description	ΣCoverage	Σ# Proteins	Σ# Unique Peptides	Σ# Peptides	Σ# PSMs	ALS/ Normal	A5: 115/114 Count	A5: 115/114 Variability [%]	# Peptides (Sequest)	# Peptides (Mascot)	MW [kDa]	calc. pI
1	4505185	MIF	macrophage migration inhibitory factor [Homo sapiens]	7.83	1	1	1	1	**0.498**	1			1	12.5	7.88
2	4557871	TF	serotransferrin precursor [Homo sapiens]	32.38	1	19	19	71	**0.484**	45	33.2	16	19	77.0	7.12
3	228480221	FHL1	four and a half LIM domains protein 1 isoform 4 [Homo sapiens]	6.19	5	1	1	2	**0.469**	1		1	1	22.0	8.46
4	33286420	PKM2	pyruvate kinase isozymes M1/M2 isoform M1 [Homo	27.50	7	13	13	55	**0.463**	36	51.6	9	13	58.0	7.71

			sapiens]												
5	4504345	HBA2	hemoglobin subunit alpha [Homo sapiens]	33.10	2	4	4	8	**0.422**	5	9.7	2	4	15.2	8.68
6	186910296	HP	haptoglobin isoform 2 preproprotein [Homo sapiens]	14.12	3	5	5	7	**0.412**	5	7.9	3	4	38.4	6.60
7	33413400	ESD	S-formylglutathione hydrolase [Homo sapiens]	2.48	1	1	1	1	**0.374**	1			1	31.4	7.02
8	4505763	PGK1	phosphoglycerate kinase 1 [Homo sapiens]	4.32	1	2	2	3	**0.370**	2	0.5	1	2	44.6	8.10
9	4504349	HBB	hemoglobin subunit beta [Homo sapiens]	31.29	2	4	4	25	**0.368**	15	35.2	4	4	16.0	7.28
10	310110685		PREDICTED: putative V-set and immunoglobulin domain-containing protein 6-like, partial [Homo sapiens]	6.57	3	1	1	2	**0.340**	1		1	1	15.3	9.10
11	4502027	ALB	serum albumin preproprotein [Homo	47.13	2	30	30	354	**0.322**	217	48.9	24	30	69.3	6.28

			sapiens]												
12	55770842	CRP	C-reactive protein precursor [Homo sapiens]	14.29	1	3	3	7	**0.315**	5	25.1	2	3	25.0	5.63
13	167857790	ORM1	alpha-1-acid glycoprotein 1 precursor [Homo sapiens]	36.32	1	5	6	21	**0.308**	12	16.5	5	5	23.5	5.11
14	295986608	IGLL5	immunoglobulin lambda-like polypeptide 5 [Homo sapiens]	14.02	1	2	2	5	**0.263**	3	16.2	1	2	23.0	8.84
15	44955885	MB	myoglobin [Homo sapiens]	28.57	1	3	3	17	**0.213**	11	61.4	2	3	17.2	7.68
16	4885099	CA3	carbonic anhydrase 3 [Homo sapiens]	11.92	1	2	2	3	**0.195**	2	61.2	1	2	29.5	7.34
17	308193325	PRSS3	trypsin-3 isoform 4 preproprotein [Homo sapiens]	5.42	4	1	1	2	**0.181**	1		1	1	25.9	5.83
